# Long-term outcomes of offspring from multiple gestations: a two-sample Mendelian randomization study on multi-system diseases using UK Biobank and FinnGen databases

**DOI:** 10.1186/s12967-023-04423-w

**Published:** 2023-09-08

**Authors:** Yi Jiang, Yuanyuan Du, Rui Su, Xuan Zhou, Lijie Wei, Jingyi Zhang, Shenglan Zhu, Huiting Zhang, Chenyun Fang, Yuting Chen, Peng Gao, Liangnan Zhang, Shaoshuai Wang, Jun Yu, Mengzhou He, Wencheng Ding, Ling Feng

**Affiliations:** 1 Department of Obstetrics and Gynecology, Tongji Hospital, Tongji Medical College, Huazhong University of Science and Technology, No. 1095, Jiefang Avenue, Wuhan, 430030 Hubei China; 2 Department of Obstetrics and Gynecology Ultrasound, Zhongnan Hospital of Wuhan University, Wuhan, 430071 China

**Keywords:** Multiple birth, Genetics, Twins, Long-term outcomes, Mendelian randomization, SNPs

## Abstract

**Background:**

Assisted reproductive technologies (ART) have increased the incidence of multiple births, which can have a negative impact on maternal and offspring health. The study aimed to investigate the association between genetically predicted multiple birth and the risk of 42 common diseases of the nervous, psychiatric, cardiovascular, respiratory, digestive, and endocrine systems.

**Methods:**

The study utilized two-sample Mendelian randomization (MR) analysis to explore the potential causal relationship between genetically predicted multiple birth and the genetically predicted risk of diseases. The study used the FinnGen and UK Biobank datasets for analysis.

**Results:**

The study found no significant causal relationship between multiple birth and psychiatric disorders. However, the lower limits of the 95% confidence intervals for bipolar affective disorder and anxiety disorders were not robust, indicating a need for further investigation. The study found that multiple birth may be a strong risk factor for infantile cerebral palsy, and caution is necessary in both natural and ART multiple births. The study revealed a potential causal relationship between multiple birth and coronary heart disease, ischemic heart disease, and deep vein thrombosis, which may be related to abnormal intrauterine environments in multiple pregnancies. Surprisingly, multiple birth appears to have a protective effect against some respiratory diseases, such as chronic obstructive pulmonary disease and asthma.

**Conclusions:**

The study highlights the need for caution regarding the risk of infantile cerebral palsy, cardiovascular diseases, and psychiatric disorders in multiple birth. Our study can lead to the development of preventive strategies and improved clinical management for affected infants.

**Supplementary Information:**

The online version contains supplementary material available at 10.1186/s12967-023-04423-w.

## Background

The world is currently facing a critical issue of population decline, with several countries experiencing a decline in birth rates [1]. This trend has raised concerns about potential negative impacts on society, including labor shortages, decreased economic growth, and strain on healthcare and social systems [2]. Various causes contribute to negative population growth, with social pressures being a significant contributor to the incidence of infertility [3], identified as a leading cause of fertility decline in some developed countries [4]. Assisted reproductive technologies (ART), including in vitro fertilization (IVF), have revolutionized the field of reproductive medicine and have allowed many couples to conceive who would otherwise have been unable to do so [5]. While the development and maturity of ART is good news for infertile patients who are eager for children and has a positive effect on negative population growth, it has also led to a higher incidence of multiple birth [6]. Although multiple birth can further ameliorate negative population growth, they may increase the health risks of mothers and offspring [7].

Mothers with multiple pregnancies may be more vulnerable to a range of complications during pregnancy [8], including but not limited to gestational hypertension [9], gestational diabetes mellitus [10], antepartum hemorrhage [11], preterm birth [12], and anemia [13]. Pregnancy complications can cause adverse outcomes for mother and child, and even have long-term adverse effects on offspring [14, 15]. Several population-based studies have consistently shown that offspring of multiple pregnancies have worse long-term neurodevelopmental outcomes than singleton pregnancies [16, 17]. Therefore, as the use of ART continues to rise, it is becoming increasingly important to understand the potential long-term health consequences of different types of pregnancies. In particular, investigating the health outcomes of offspring of multiple pregnancies is crucial to ensure their long-term well-being and inform strategies to mitigate the negative impact of population decline.

However, it is not clear whether the susceptibility of twin offspring to certain diseases is due to innate genetic factors or acquired environmental factors, such as epigenetic changes. Furthermore, it is unclear whether twin pregnancy itself or complications caused by twin pregnancy cause the adverse long-term outcomes in offspring. This field is largely unexplored due to the length of follow-up required for such studies and the difficulty of intervention.

Mendelian randomization (MR), as an emerging epidemiological method in recent years, utilizes genetic variants as instrumental variables (IVs) to assess the causal effects of exposure factors on outcomes [18]. Here, "exposure" represents a risk factor, an intermediate phenotype, which may or may not serve as a potential causal biomarker which affects the "outcome" (for example, a certain disease) [19]. The possibility of reverse causation is minimized because genotype is established at conception (Mendel’s law of segregation). Thus, with the robust and reliable IVs provided by genome-wide association studies (GWAS), the MR analysis can be considered to be in accordance with the normal causal order [20].

This study aims to estimate the association between genetically predicted part of a multiple birth and the genetically predicted risk of several diseases. Using the FinnGen and UK Biobank databases, we assessed the susceptibility of offspring of multiple birth to 42 common diseases of the nervous, psychiatric, cardiovascular, respiratory, digestive, and endocrine systems.

## Methods

### Data sources

**Fig. 1:**
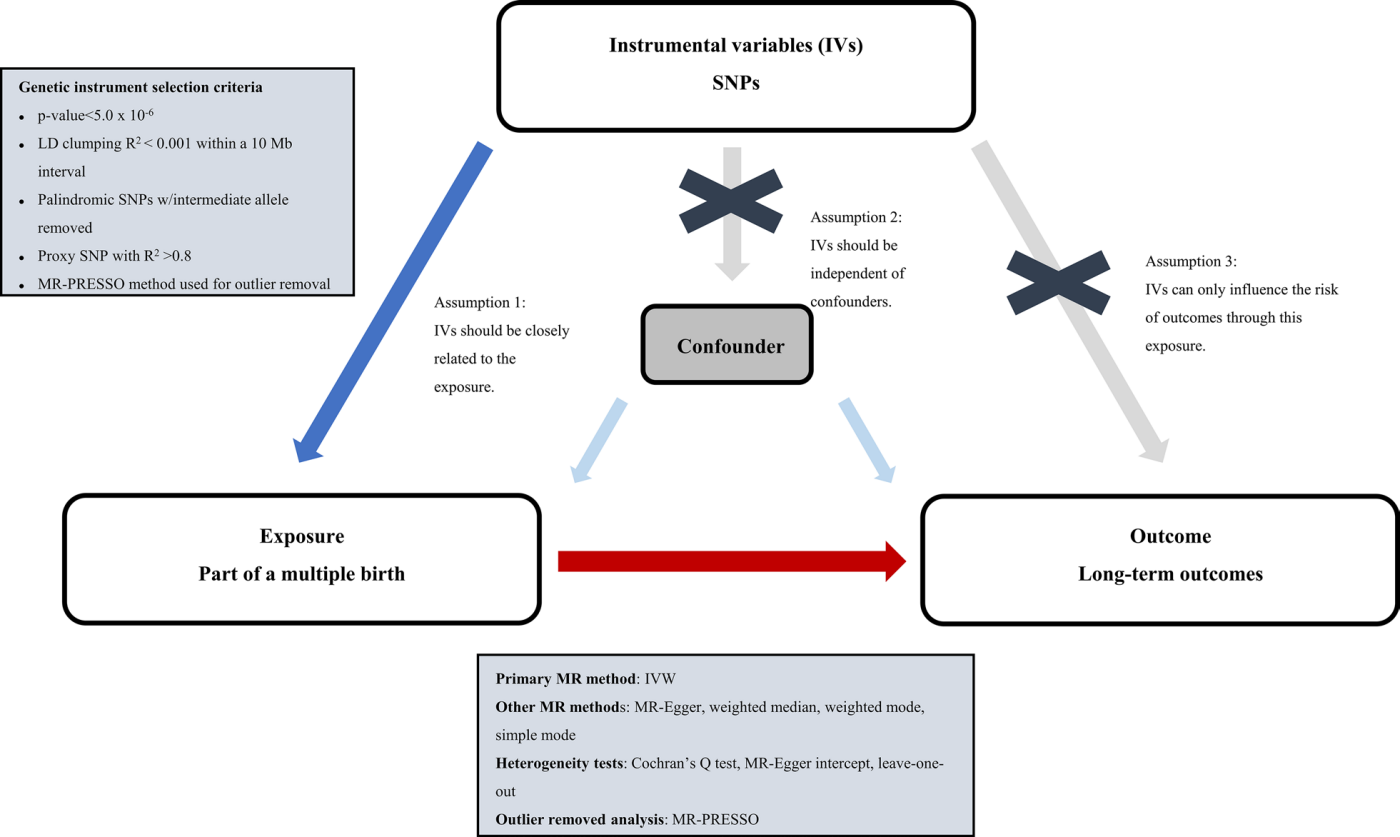
Study design overview

The study design is presented in Fig. 1 and adhered to the STROBE-MR guidelines [21]. We obtained genetic variants for multiple birth from the UK Biobank database [22] and limited out analysis to the largest European population, consisting of 414,217 individuals (including 9550 cases and 404,667 controls), in order to maintain consistency in ancestry between the exposure and outcome groups. The study collected responses to the question “Are you a twin, triplet or other multiple birth?” from all participants, except those who reported being adopted as a child. We obtained GWAS summary statistics for long-term outcomes from UK Biobank and FinnGen consortium R8 release data [23], and explored specific outcomes, including: attention deficit hyperactivity disorder, depression, autism, bipolar affective disorder, Alzheimer’s disease, cerebral palsy (or infantile cerebral palsy), epilepsy, cognitive impairment, schizophrenia, mood disorders, anxiety disorders, suicide or self-inflicted injury, migraine, mental retardation, stroke, subarachnoid hemorrhage, transient ischemic attack, pulmonary embolism, deep vein thrombosis, arterial hypertension, atrial fibrillation and flutter, ischemic heart disease, coronary heart disease (coronary atherosclerosis), cardiomyopathy, myocardial infarction, chronic obstructive pulmonary disease, asthma, bronchitis, tuberculosis, gastric ulcer, duodenal ulcer, Crohn's disease, fibrosis and cirrhosis of liver, gastritis (acute or chronic), thyrotoxicosis, hypothyroidism, thyroiditis, type 1 diabetes, type 2 diabetes, gout and obesity. Sex, age, principal components, and genotyping batch were corrected during the analysis.

### Instrumental variable (IV)

In this study, we conducted a two-sample MR analysis [24] using single nucleotide polymorphisms (SNPs) as instrumental variables (IVs) to investigate the relationship between multiple birth and long-term outcomes [25]. As depicted in Fig. 1, our analysis satisfies three principal hypotheses of classical MR analysis: (1) IVs were strongly associated with the exposure; (2) IVs were independent of confounders; and (3) IVs influenced the risk of outcomes solely through the exposure.

In this study, we employed stringent criteria to select IVs for our two-sample MR analysis. All SNPs that were significantly associated with multiple birth (p < 5 × 10^–6^) were considered as IVs, and we applied LD pruning to ensure that the selected SNPs were independent within a 10 Mb window with an r^2^ < 0.001. We also searched for secondary phenotypes in PhenoScanner [26] and GWAS Catalog [27] to rule out any potential confounding effects, and SNPs corresponding to the outcomes of interest were excluded. We evaluated the strength of the IVs using variance (R^2^) and F-statistics to assess the extent of weak instrument bias [28]. R^2^ was calculated using the formula of 2 × MAF × (1-MAF) × β^2^, and F was calculated using the formula of R^2^ × (N − K − 1)/(1 − R^2^). If F > 10, we considered the correlation between IVs and exposure to be strong enough, and the MR analysis results would be less affected by weak instrument bias.

In total, 19 SNPs were selected as IVs to represent multiple birth (see Additional file 1: Table S1). Among these SNPs, rs605765 was associated with secondary phenotypes such as waist circumference, weight and number of operations, some of which are correlated with BMI. As BMI is a confounding factor in many diseases, we performed a one-leave-out test to assess the stability of our MR analysis results after removing this SNP. In further analyses, we deleted palindromic SNPs with a moderate allele frequency.

### Statistical analysis

In this study, various MR methods, such as inverse variance weighted (IVW) [29], MR-Egger regression [30], weighted median [31], weighted mode [32], simple mode [33] and MR-PRESSO [34], were employed to investigate the potential causal relationship between multiple birth and long-term disease. The IVW was selected as the primary MR analysis, and the key features of each model were presented in Additional file 2: Table S2.

To ensure the robustness of our findings, we employed various sensitivity analyses. To assess the heterogeneity of the IVs, we conducted Cochran’s Q test, where a p value < 0.05 indicates heterogeneity [35]. We used MR-PRESSO with NbDistribution = 10,000 to identify any outliers and excluded them from the analysis. The MR-Egger intercept method was used to test for the presence of horizontal pleiotropic of IVs, and a p value < 0.05 suggests that the IVW estimate may be biased [30]. Moreover, we performed a leave-one-out sensitivity test to evaluate the impact of individual SNPs on the causal effect. Additionally, we generated funnel and forest plots to detect the presence of pleiotropy.

All statistical analyses were conducted using TwoSampleMR [36] packages in the R software (4.2.2), and all p values were two-tailed. A threshold of p < 0.05 was deemed statistically significant.

## Results

### Multiple birth and mental illness

**Fig. 2:**
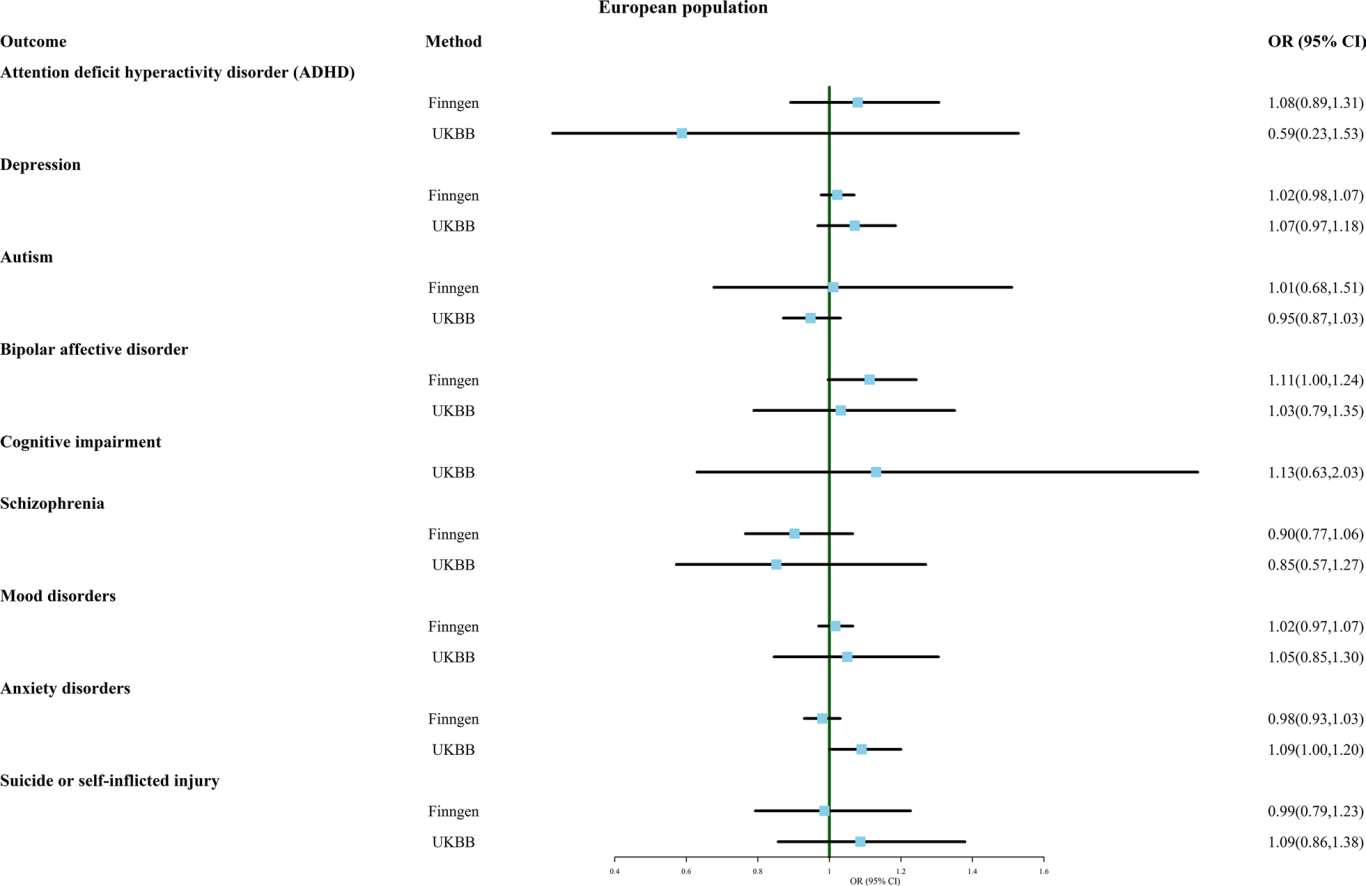
Association of genetically predicted multiple birth with risk of mental illness. Estimates were obtained from the inverse-variance weighted methods

In this part, we explored the association between multiple birth and nine common psychiatric disorders, including attention deficit hyperactivity disorder (ADHD), depression, autism, bipolar disorder, cognitive impairment, schizophrenia, mood disorders, anxiety disorders, and suicide or self-inflicted injury, using MR analysis. Figure 2 shows the results of the forest plot using the IVW method. The results suggest that there is no significant causal relationship between multiple birth and any of these psychiatric disorders, as observed in both FinnGen and UK Biobank datasets.

Additional file 3: Table S3 shows the results of other MR methods, including MR Egger, weighted median, weighted mode, simple mode, and MR-PRESSO, all of which also did not show a significant causal relationship between multiple birth and these outcomes. Sensitivity analyses showed no heterogeneity except for the analysis of depression in the UK Biobank database. After removing outliers, still no significant causal relationship was observed between multiple birth and depression. The MR Egger intercept and MR-PRESSO outcomes indicate the absence of directional pleiotropy.

Additional file 9: Material S1 provides funnel plots, scatter plots, and one-leave-out test results for all outcomes to visualize heterogeneity more clearly. However, the results for bipolar disorder in the FinnGen database (OR 1.112, 95% CI 0.996–1.243) and anxiety disorders in the UK Biobank database (OR 1.092, 95% CI 0.996–1.198) were not robust, and the exclusion of certain SNPs led to the emergence of significant results that were previously non-significant, indicating a potential causal relationship between multiple birth and these two disorders.

### Multiple birth and nervous system disease

**Fig. 3:**
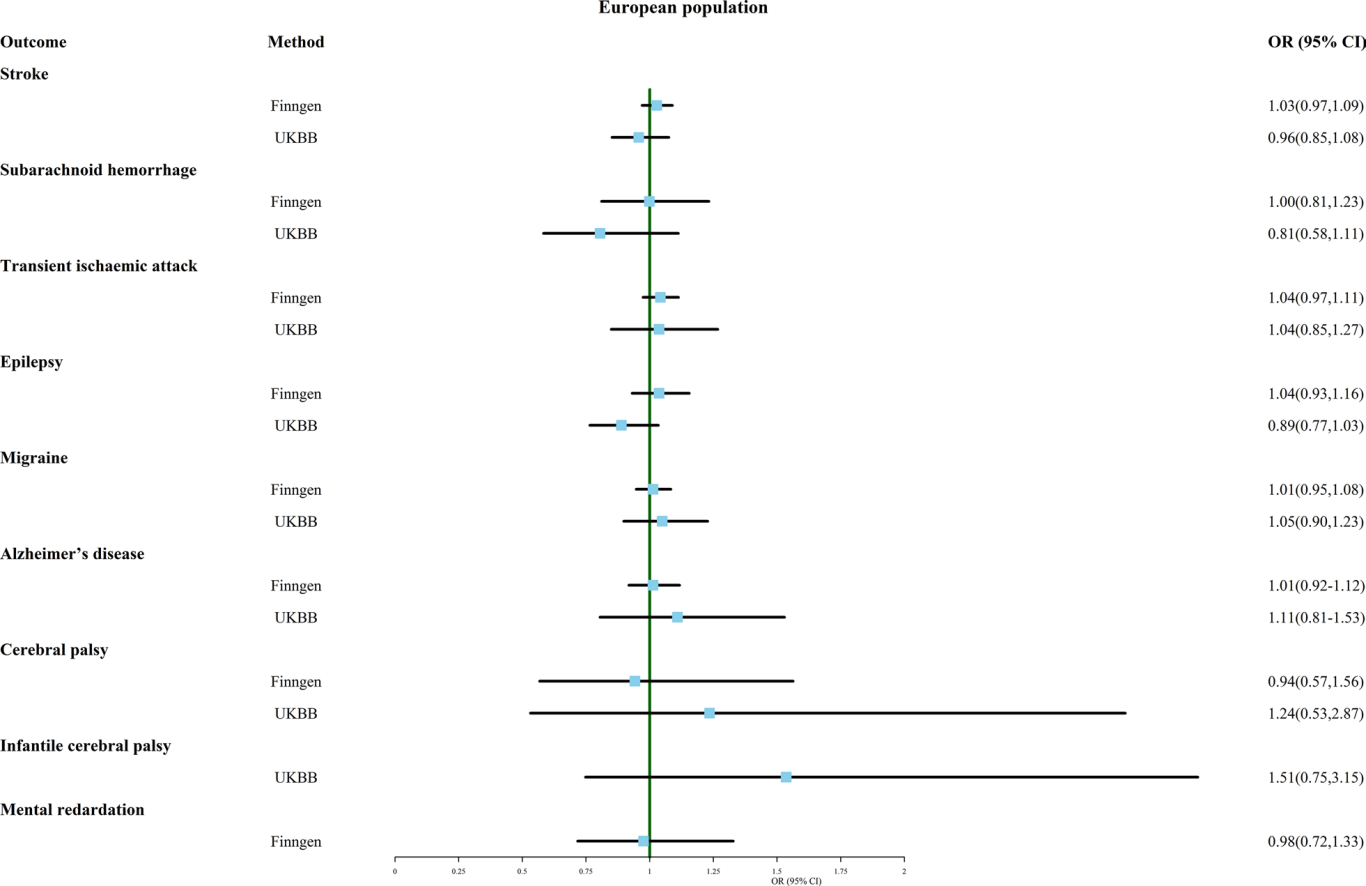
Association of genetically predicted multiple birth with risk of nervous system disease. Estimates were obtained from the inverse-variance weighted methods

In this section, we conducted an MR analysis to explore the association between multiple birth and eight neurological disorders, including stroke, subarachnoid hemorrhage, transient ischemic attack, epilepsy, migraine, Alzheimer's disease, adult and infantile cerebral palsy, and mental retardation. The IVW results, as shown in Fig. 3, suggest that there is no significant causal relationship between multiple birth and any of the neurological disorders investigated, based on outcome data from both FinnGen and UK Biobank.

However, the MR Egger analysis results, presented in Additional file 4: Table S4, suggest that multiple birth is strongly associated with infantile cerebral palsy (OR 5.191, 95% CI 1.169–23.051), even though this association was not detected by the IVW method. It is worth noting that this result should be interpreted with caution due to the small number of cases in the UK Biobank data for infantile cerebral palsy (ncase = 166).

Despite the sensitivity analysis revealing heterogeneity in the results for epilepsy and cerebral palsy in the FinnGen database, MR-PRESSO did not detect any outliers. In addition, the association between multiple birth and transient ischemic attack from the FinnGen database was not reliable due to significant pleiotropy.

Additional file 10: Material S2 provides funnel plots, scatterplots, and one-leave-out test results for all analyses. It is noteworthy that the result for the association between multiple birth and epilepsy changed when rs72892862 was excluded. Multiple birth appeared to be a protective factor for epilepsy in this case (OR 0.859, 95% CI 0.743–0.993).

Overall, these findings suggest that multiple birth is not significantly associated with any of the neurological disorders investigated, except for a potential strong association with infantile cerebral palsy that requires further investigation.

### Multiple birth and cardiovascular system disease

**Fig. 4:**
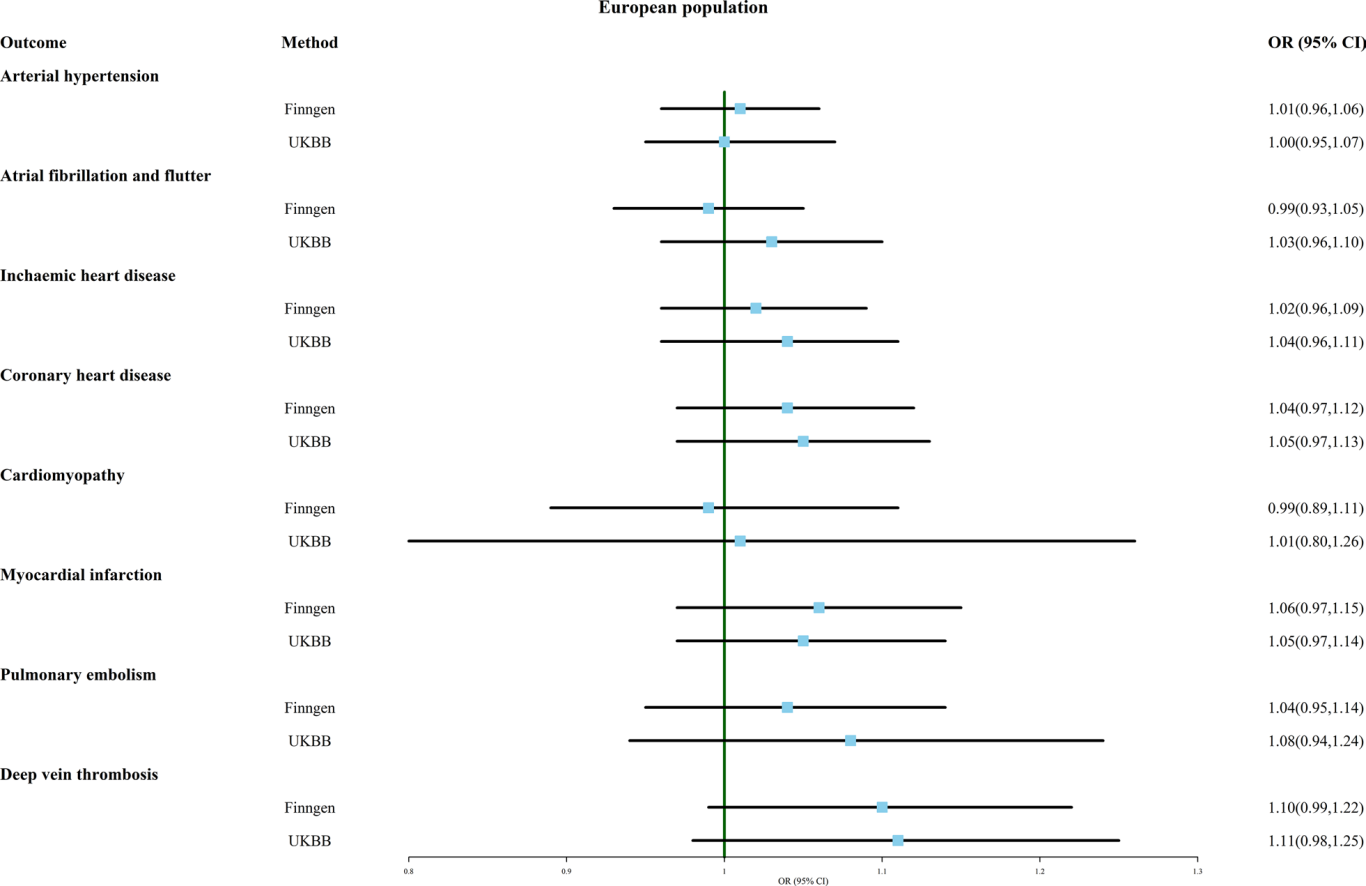
Association of genetically predicted multiple birth with risk of cardiovascular system disease. Estimates were obtained from the inverse-variance weighted methods

As shown in Fig. 4, MR analysis using IVW did not observe any significant causal relationship between multiple birth and arterial hypertension, atrial fibrillation and flutter, ischemic heart disease, coronary heart disease, cardiomyopathy, myocardial infarction, pulmonary embolism, or deep vein thrombosis.

Additional file 5: Table S5 provides the results of other analytical methods. The simple mode method suggested that multiple birth may be a risk factor for ischemic heart disease (OR 1.092, 95% CI 1.005–1.187, FinnGen). MR-PRESSO detected outliers in the results of multiple birth and coronary heart disease (FinnGen). When this outlier was removed, multiple birth appeared to be more strongly associated with coronary heart disease (OR 1.065, 95% CI 0.998–1.132) (Additional file 11: Material S3).

Although MR analysis found significant heterogeneity between multiple birth and arterial hypertension (FinnGen and UKBB), ischemic heart disease (FinnGen), and myocardial infarction (FinnGen), MR-PRESSO did not detect any single outlier. There seems to be a potential causal relationship between multiple birth and deep vein thrombosis, whether the data for deep vein thrombosis is from FinnGen (OR 1.101, 95% CI 0.992–1.223) or UKBB (OR 1.105, 95% CI 0.978–1.249). The results of the one-leave-out test also suggest that both of these results are susceptible to the influence of a single SNP, and the estimated causal relationship becomes more significant after removing a certain SNP (Additional file 11: Material S3).

### Multiple birth and respiratory system disease

**Fig. 5:**
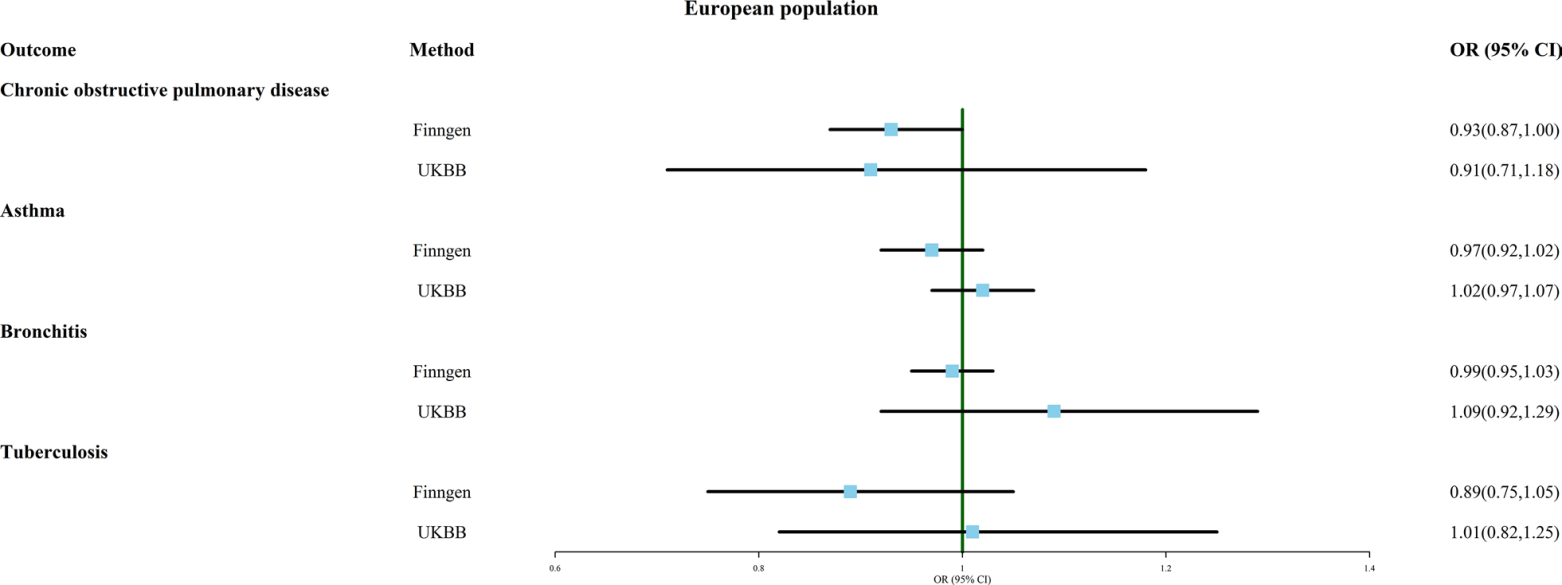
Association of genetically predicted multiple birth with risk of respiratory system disease. Estimates were obtained from the inverse-variance weighted methods

Figure 5 displays the correlation between multiple birth and four respiratory diseases: chronic obstructive pulmonary disease (COPD), asthma, bronchitis, and tuberculosis, as analyzed through MR analysis. The results suggest that multiple birth may be a protective factor against lifelong COPD (OR 0.933, 95% CI 0.873–0.998, FinnGen). Regardless of the MR analysis method used (as shown in Additional file 6: Table S6), no significant causal relationship was found between multiple birth and other respiratory diseases, and all results showed no significant heterogeneity or pleiotropy. As shown in Additional file 12: Material S4, when rs143496908 was removed, the MR analysis result showed a reduced probability of multiple birth being associated with asthma (OR 0.953, 95% CI 0.909–0.999, FinnGen).

### Multiple birth and digestive system disease

**Fig. 6:**
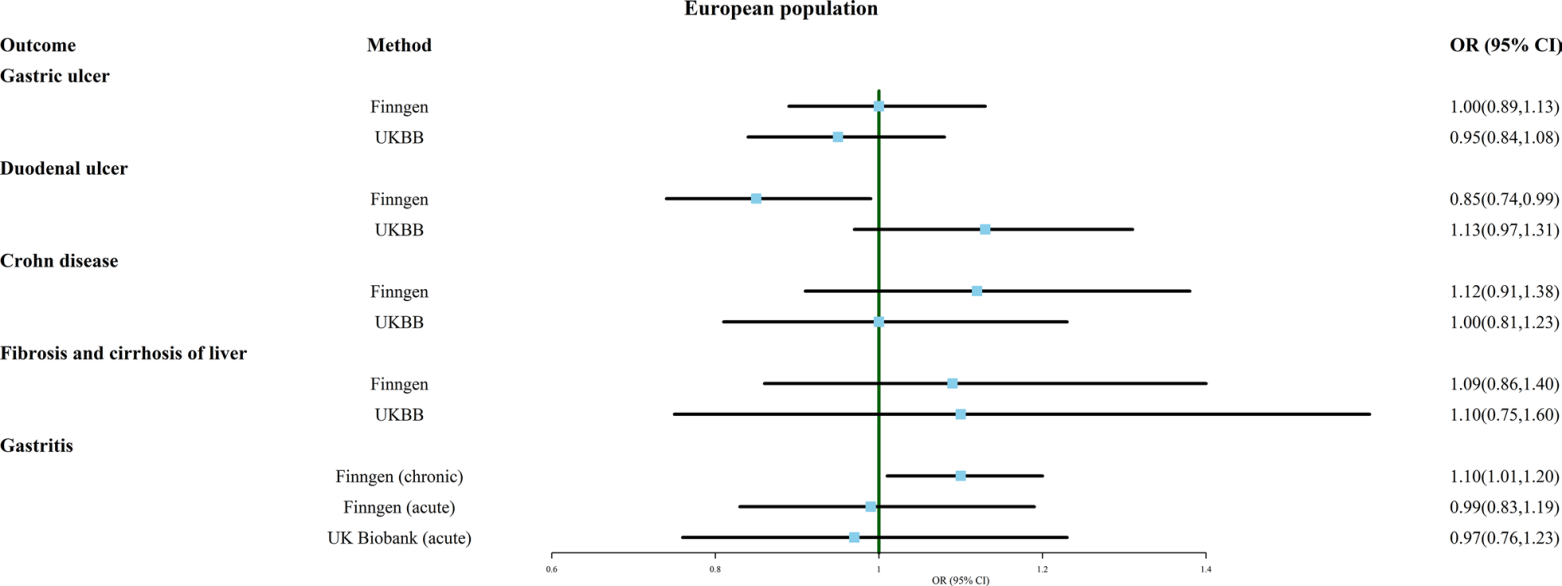
Association of genetically predicted multiple birth with risk of digestive system disease. Estimates were obtained from the inverse-variance weighted methods

Next, we investigated the association between multiple birth and several common digestive system disorders, including gastric ulcer, duodenal ulcer, Crohn's disease, fibrosis and cirrhosis of liver, and gastritis (chronic and acute). As shown in Fig. 6, the probability of multiple birth being diagnosed with duodenal ulcer was decreased (OR 0.853, 95% CI 0.738–0.986, FinnGen). However, this result should be interpreted with caution because this outcome was only observed in the FinnGen database, and the trend was actually reversed in the UK Biobank (OR 1.126, 95% CI 0.966–1.314). In addition, multiple birth seemed to be more susceptible to chronic gastritis (OR 1.101, 95% CI 1.008–1.204). The MR analysis results from other methods and sensitivity analyses are presented in Additional file 7: Table S7. Although MR Egger suggested a potential association between multiple birth and gastric ulcer (OR 0.693, 95% CI 0.530–0.906), the result was not reliable due to significant pleiotropy (p < 0.05). Furthermore, the analysis of fibrosis and cirrhosis of liver (UK Biobank) showed significant heterogeneity, and even after removing outliers using MR-PRESSO, no significant association was found between multiple birth and these diseases.

Additional file 13: Material S5 provides the funnel plot, scatter plot, and one-leave-out test results. Previously discovered associations between multiple birth and duodenal ulcer (FinnGen) and chronic gastritis (UKBB) were found to change upon removal of a single SNP, indicating that the results are not robust.

### Multiple birth and endocrine system disease

**Fig. 7:**
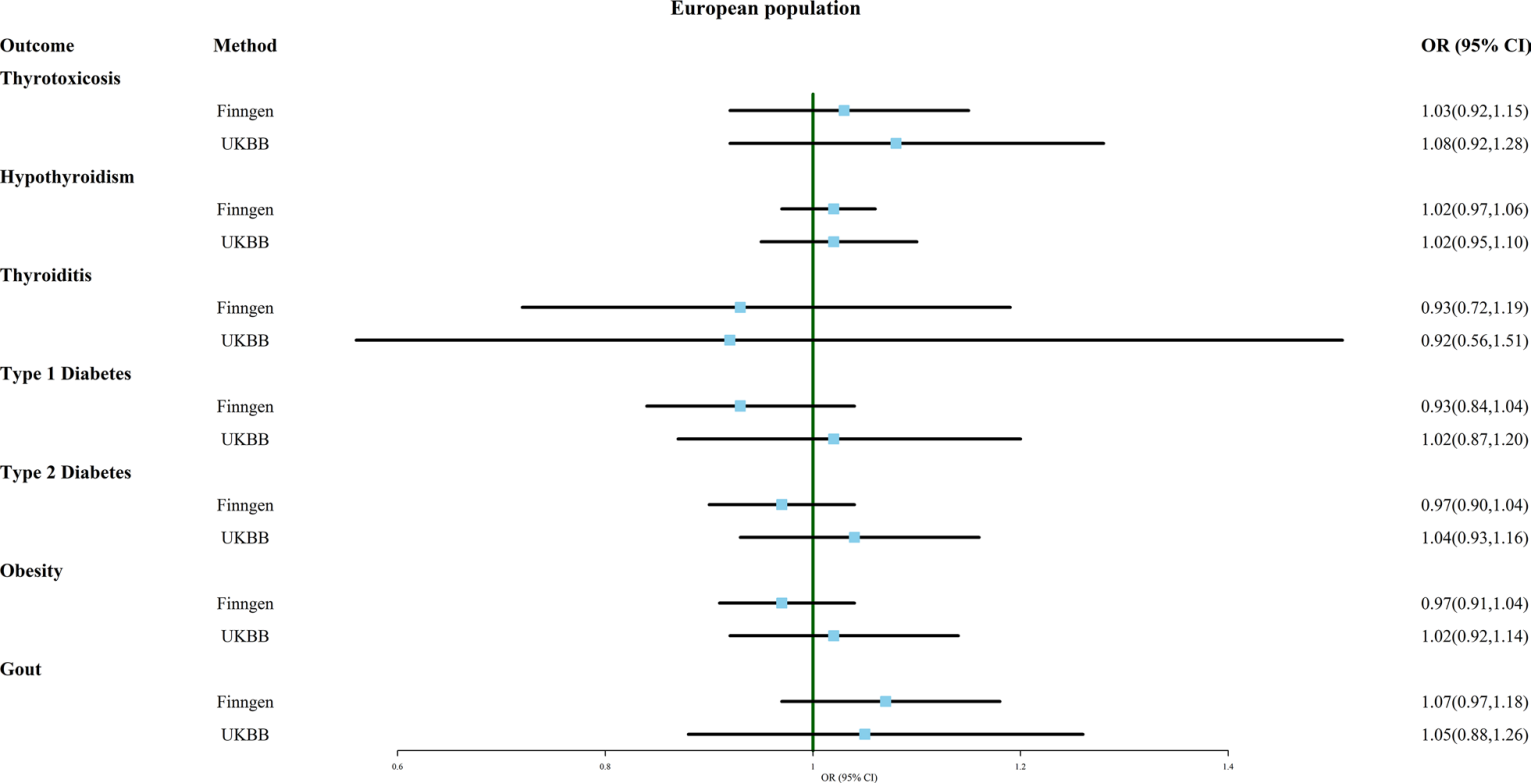
Association of genetically predicted multiple birth with risk of endocrine system disease. Estimates were obtained from the inverse-variance weighted methods

As shown in Fig. 7 and Additional file 8: Table S8, the IVW analysis indicated that the likelihood of developing seven common endocrine disorders, including thyrotoxicosis, hypothyroidism, thyroiditis, type 1 diabetes, type 2 diabetes, obesity, and gout, did not significantly differ between multiple birth and singleton birth. Only the MR Egger analysis suggested that multiple birth may be protective against thyroiditis (OR 0.631, 95% CI 0.409–0.971, FinnGen). None of the analyses showed significant pleiotropy, and some results with heterogeneity did not change after removing outliers. Moreover, all results were robust to the exclusion of individual SNPs (Additional file 14: Material S6).

## Discussion

Assisted reproductive technologies have raised multiple birth rates, potentially heightening health risks for both mothers and offspring. This study aimed to investigate the association between genetically predicted part of a multiple birth and the genetically predicted risk of 42 common diseases of the nervous, psychiatric, cardiovascular, respiratory, digestive, and endocrine systems.

Regarding psychiatric disorders, we found no significant causal relationship between multiple birth and any investigated psychiatric disorders, whether the data came from FinnGen or UK Biobank. This result may differ from some clinical studies [37, 38]. Possible explanations could be that childhood psychiatric disorders might be influenced by environmental or epigenetic factors [39]. The upbringing and surroundings of multiple offspring differ from those of singletons, and certain family and social factors, such as strict parenting, could contribute to these differences in environmental and epigenetic factors [40]. Additionally, while our study suggests that multiple pregnancies themselves may not increase the risk of psychiatric disorders in offspring, pregnant women with multiple pregnancies may be more susceptible to various pregnancy complications [41, 42], some of which, such as preterm birth [43], have been associated with psychiatric disorders in offspring [44]. Besides, one-leave-out tests revealed that the results for bipolar affective disorder (OR 1.112, 95% CI 0.996–1.243, FinnGen) and anxiety disorders (OR 1.092, 95% CI 0.996–1.198, UKBB) lacked robustness, with the lower limits of their 95% confidence intervals approaching non-significant values. Removing specific individual SNPs could render these correlations significant. In fact, in 2019, Amrhein and other statisticians cautioned researchers about avoiding the "significance trap": science researcher should never conclude that there is "no difference" or "no association" just because a p value is larger than a threshold such as 0.05 or, equivalently, because a confidence interval includes zero [45]. Therefore, we still need to be cautious about the risk of multiple birth offspring developing bipolar affective disorder and anxiety disorders in the future. It is important to acknowledge that the upbringing environment within families with multiple birth can be more intricate than anticipated. A child in a twin family may experience enhanced happiness due to having a companion during their growth, but they might also encounter dissatisfaction from the need to share their belongings. Furthermore, parental attitudes towards their children could also play a role, particularly evident in dizygotic twins (especially when of different genders). These factors could potentially increase or decrease a child's susceptibility to certain mental disorders. However, no current observational studies compare the lifetime prevalence of these two disorders between multiple birth and singleton offspring, which could be a starting point for future research.

IVW analysis found no significant causal relationship between multiple birth and several common neurological diseases. However, according to MR Egger, multiple birth may be a strong risk factor for infantile cerebral palsy (OR 5.191, 95% CI 1.169–23.051). Unlike the IVW method, which assumes that all genetic variants affecting the outcome variable come from the same causal pathway, the MR Egger method is more flexible and can draw causal conclusions from non-causal genetic correlations as long as a weak bias assumption is met [46]. Therefore, although MR Egger's analysis not being the primary approach, the substantial OR value demands careful consideration. Previous clinical studies have also shown an increased risk of infantile cerebral palsy in multiple birth [47–50]. Spangmose et al. found no significant rise in ORs of cerebral palsy in ART twins compared to naturally conceived twins, providing strong evidence against the continued use of multiple embryo transfer in most ART settings [51]. The primary risk factors for infantile cerebral palsy are prematurity and low birth weight [52, 53], occurrences more prevalent in multiple pregnancies than in singleton ones [12, 54], which may be the reason for the increased incidence of cerebral palsy in multiple births. Our study results indicate that caution should be exercised regarding the risk of infantile cerebral palsy in both natural and ART multiple births. Additionally, since the phenotype of infantile cerebral palsy is only provided in the UK Biobank and the number of cases is small (ncase = 166), the non-significant IVW results may be due to the small sample size. Besides, it is worth noting that the lifetime risk of epilepsy in multiple birth appears to be lower. Further studies are needed to explore the potential mechanisms underlying the association between multiple birth and epilepsy, which may ultimately lead to the development of preventive strategies and improved clinical management for affected infants.

After excluding outliers, IVW analysis revealed a potential causal relationship between multiple birth and coronary heart disease (OR 1.065, 95% CI 0.998–1.132, FinnGen). Simple mode also suggested an increased risk of ischemic heart disease in multiple births (OR 1.092, 95% CI 1.005–1.187, FinnGen). In addition, GWAS data from FinnGen (OR 1.101, 95% CI 0.992–1.223) and UK Biobank (OR 1.105, 95% CI 0.978–1.249) suggested a potential causal relationship between multiple birth and deep vein thrombosis, and both results could become significant after the exclusion of a single SNP. Previous studies have suggested that abnormalities in the early intrauterine environment may lead to adverse long-term effects in offspring, recognized as metabolic memory (MM). Multiple clinical and experimental studies have shown that MM may cause persistent endothelial dysfunction in offspring, amplifying the vulnerability to long-term cardiovascular complications [14, 15]. Our study suggests an augmented susceptibility to certain cardiovascular diseases in multiple birth, potentially tied to aberrations in the intrauterine environment in multiple pregnancies. Previous studies have shown that pregnant women with multiple pregnancies face a heightened risk of metabolic disorders such as gestational diabetes [55], which may lead to a high glucose environment in the uterus and result in metabolic memory in offspring during early life. Therefore, pregnant women with multiple pregnancies should diligently monitor their blood glucose levels throughout gestation, and individuals with multiple births should be vigilant about the occurrence of cardiovascular diseases in adulthood. This conclusion needs to be confirmed by large-scale clinical studies and further investigation into changes in the intrauterine environment in multiple births and their impact on the development of lifelong cardiovascular diseases in offspring.

Surprisingly, multiple birth appears to shield against some respiratory diseases, such as COPD (OR 0.933, 95% CI 0.873–0.998, FinnGen) and asthma (OR 0.953, 95% CI 0.909–0.999, FinnGen), excluding single SNP. One possible explanation is that twins could acquire early-life immune safeguards via shared placenta and fetal membranes, potentially curbing future asthma and COPD risk. Furthermore, in the Finnish population, multiple births also exhibit reduced susceptibility thyroiditis (OR 0.631, 95% CI 0.409–0.971, FinnGen). Non-identical twins sharing the uterine environment are exposed to non-identical antigens from each other, which may induce a higher level of tolerance. This partially elucidates multiple births’ conceivable role as a protective factor against asthma and thyroiditis, necessitating validation through subsequent clinical and basic research.

MR analysis regarding the link between multiple birth and duodenal ulcer, based on data from FinnGen and UK Biobank, exhibited divergent trends. FinnGen’s data suggests that multiple birth may be a protective factor for duodenal ulcer (OR 0.853, 95% CI 0.738–0.986), while UK Biobank’s data suggests a potential causal relationship between multiple birth and increased risk of duodenal ulcer (OR 1.126, 95% CI 0.966–1.314). Notably, most results obtained from the two databases do not match completely, possibly stemming from differences in population and age. UK Biobank focused on individuals aged 40–69 within the UK, while FinnGen's study population may include any Finnish residents. In addition, FinnGen's data also indicates an increased risk of chronic gastritis in multiple birth (OR 1.101, 95% CI 1.008–1.204). These results need to be interpreted with caution, and studies on the impact of twins on disease incidence rates among different populations are necessary.

Although multiple births can ameliorate negative population growth, they may increase the health risks of mothers and offspring. Pregnancy complications can cause adverse outcomes for mother and child, and even have long-term adverse effects on offspring. Thus, as the use of ART gain prominence, it is crucial to investigate the health prospects of offspring from multiple pregnancies, safeguarding their long-term well-being and guiding strategies to counterbalance the adverse consequences of population decline. Moreover, ART-conceived and naturally conceived multiple births may exhibit some differences in the genetic component. ART, encompassing in vitro fertilization and cultivation, involves additional laboratory procedures and interventions that could potentially influence genetic component. Processes like embryo screening within ART might lead to differences in the frequency of certain gene types or variants in ART-conceived individuals. Similarly, the process of embryo cultivation and implantation could also impact their genetic composition. However, it should be noted that in most genetic characteristics, ART-conceived and naturally conceived children should be similar since the genetic information primarily originates from the genetic contributions of their parents. The genetic information of ART-conceived infants still derives from their biological parents' genetic material, and the in vitro fertilization process does not involve modifications to the genome.

The study’s results have implications for clinicians and policymakers. They highlight the potential for varied disease risk among offspring of multiple pregnancies. Vigilant monitoring of the long-term health trajectories of these offspring, particularly those born from ART, becomes imperative. Further research is needed to investigate the potential associations between multiple birth and other diseases and conditions.

The study's strengths include its large sample size, utilization of MR analysis, and the availability of data from two large databases. By using "a more realistic p value", Smith et al. suggest that compared to conventional observational epidemiology, Mendelian randomization studies can capitalize on the limited associations between a specific genetic variant and other genetic variants, as well as between genetic and nongenetic variables. This enables them to provide an unbiased estimation of the relationship between the factors directly influenced by the genetic variant and disease outcomes, making them less susceptible to confounding [56].

However, this study has several limitations. Firstly, the absence of pertinent data hindered the ability to perform separate analyses for monozygotic and dizygotic multiple births. Specifically, in dizygotic multiple births, there may be significant differences in genotypes among individual fetuses. Similarly, comparing distinctions between ART and non-ART twins proved challenging, given potential genetic variations introduced by the ART process. Secondly, the study population included only individuals of European ancestry, and the results need to be validated in more diverse populations. Thirdly, while two-sample Mendelian randomization is a powerful discovery tool, one-sample MR would confirm these findings and enable a direct evaluation of potential confounding factors and covariates that may contribute to pleiotropic effects. Lastly, the results obtained through Mendelian randomization in this study lack direct support from relevant clinical randomized controlled trials, warranting cautious interpretation. Despite these limitations, the study provides valuable insights into the potential health risks associated with multiple births.

## Conclusion

In summary, this research adds to the growing body of evidence on the health risks associated with multiple birth. Although some of the findings lack robustness, the study suggests that individuals who are born from multiple pregnancies should be aware of the increased lifetime risk for bipolar affective disorder, anxiety disorders, infantile cerebral palsy, coronary heart disease, ischemic heart disease, deep vein thrombosis, and chronic gastritis. This study emphasizes the importance of investigating the health outcomes of individuals who are born from multiple pregnancies and highlights the need for further research on the long-term health consequences of various types of pregnancies. The study's findings have important clinical implications for managing multiple pregnancies and the long-term health outcomes of offspring.

### Supplementary Information

**Additional file 1: Table S1**. SNPs that were used as instrumental variables met the conditions: p value < 5 × 10–6 and F > 10. * The secondary phenotypes of rs605765 obtained by PhenoScanner include waist circumference, weight and number of operations, some of which are associated with BMI, and BMI was identified as a confounder for lots of diseases. In the subsequent one-leave-out test, we focused on whether the MR Results were stable after removing this SNP.

**Additional file 2: Table S2**. Characteristics of each method used in this study.

**Additional file 3: Table S3**. Two-sample Mendelian randomization estimations showing the effects, heterogeneity and horizontal pleiotropy of multiple birth on the risk of mental illness.

**Additional file 4: Table S4**. Two-sample Mendelian randomization estimations showing the effects, heterogeneity and horizontal pleiotropy of multiple birth on the risk of nervous system disease.

**Additional file 5: Table S5**. Two-sample Mendelian randomization estimations showing the effects, heterogeneity and horizontal pleiotropy of multiple birth on the risk of cardiovascular system disease.

**Additional file 6: Table S6**. Two-sample Mendelian randomization estimations showing the effects, heterogeneity and horizontal pleiotropy of multiple birth on the risk of respiratory system disease.

**Additional file 7: Table S7**. Two-sample Mendelian randomization estimations showing the effects, heterogeneity and horizontal pleiotropy of multiple birth on the risk of digestive system disease.

**Additional file 8: Table S8**. Two-sample Mendelian randomization estimations showing the effects, heterogeneity and horizontal pleiotropy of multiple birth on the risk of endocrine system disease.

**Additional file 9: Material S1**. The scatter plot, funnel plot and leave-one-out plot for the MR analysis of multiple birth and mental illness.

**Additional file 10: Material S2**. The scatter plot, funnel plot and leave-one-out plot for the MR analysis of multiple birth and nervous system disease.

**Additional file 11: Material S3**. The scatter plot, funnel plot and leave-one-out plot for the MR analysis of multiple birth and cardiovascular system disease.

**Additional file 12: Material S4**. The scatter plot, funnel plot and leave-one-out plot for the MR analysis of multiple birth and respiratory system disease.

**Additional file 13: Material S5**. The scatter plot, funnel plot and leave-one-out plot for the MR analysis of multiple birth and digestive system disease.

**Additional file 14: Material S6**. The scatter plot, funnel plot and leave-one-out plot for the MR analysis of multiple birth and endocrine system disease.

## Data Availability

The summary data of Pan-UK Biobank can be downloaded from the website https://pan.ukbb.broadinstitute.org/downloads/index.html. The summary data of FinnGen can be downloaded from the website https://www.finngen.fi/en/access_results. The other datasets generated and/or analysed during the current study are publicly available and included in this published article and its supplementary information files.

## Abbreviations

ADHD: Attention deficit hyperactivity disorder
ART: Assisted reproductive technologies
COPD: Chronic obstructive pulmonary disease
GWAS: Genome-wide association studies
IVF: In vitro fertilization
IVs: Instrumental variables
MM: Metabolic memory
MR: Mendelian randomization
SNP: Single nucleotide polymorphism
UKBB: UK Biobank
