# Supplementary material for: Long-term outcomes of offspring from multiple gestations: a two-sample Mendelian randomization study on multi-system diseases using UK Biobank and FinnGen databases
Source: J Transl Med. 2023 Sep 8;21:608. doi: 10.1186/s12967-023-04423-w (PMC10492369; doi:10.1186/s12967-023-04423-w)
Supplement: Supplementary file 1 — Additional file 1: Table S1. SNPs that were used as instrumental variables met the conditions: p value < 5 × 10^–6^ and F > 10. * The secondary phenotypes of rs605765 obtained by PhenoScanner include waist circumference, weight and number of operations, some of which are associated with BMI, and BMI was identified as a confounder for lots of diseases. In the subsequent one-leave-out test, we focused on whether the MR Results were stable after removing this SNP. [file 12967_2023_4423_MOESM1_ESM.docx]

| Chr | Position | SNP | Other allele | Effect allele | EAF | Beta | Se | Pvalue | R^2^ | F | Secondary phenotypes |
| --- | --- | --- | --- | --- | --- | --- | --- | --- | --- | --- | --- |
| 1 | 36822239 | rs4652908 | T | G | 0.6017 | 0.07874 | 0.01535 | 2.90E-07 | 0.002972 | 28.45877 | No data |
| 1 | 190756262 | rs513810 | G | A | 0.4099 | -0.0697 | 0.01522 | 4.66E-06 | 0.00235 | 22.49227 | No data |
| 2 | 4518155 | rs77224821 | T | A | 0.02438 | 0.2754 | 0.0567 | 1.19E-06 | 0.003608 | 34.57438 | No data |
| 3 | 23146682 | rs6783710 | C | T | 0.5486 | 0.07092 | 0.01507 | 2.53E-06 | 0.002491 | 23.84407 | No data |
| 3 | 56984055 | rs76954888 | G | A | 0.1165 | 0.1125 | 0.02459 | 4.76E-06 | 0.002605 | 24.94094 | Mean platelet volume |
| 4 | 174822669 | rs146477477 | G | A | 0.04156 | 0.2103 | 0.04316 | 1.10E-06 | 0.003523 | 33.75937 | No data |
| 5 | 72828747 | rs60326831 | A | G | 0.05622 | 0.1812 | 0.0354 | 3.08E-07 | 0.003484 | 33.38384 | No data |
| 5 | 163843131 | rs143496908 | G | A | 0.01785 | 0.3063 | 0.06604 | 3.52E-06 | 0.00329 | 31.51254 | No data |
| 6 | 110256120 | rs4484552 | C | A | 0.005759 | -0.3945 | 0.08136 | 1.24E-06 | 0.001782 | 17.04707 | No data |
| 6 | 148344581 | rs73015178 | T | C | 0.01169 | 0.4326 | 0.08734 | 7.31E-07 | 0.004324 | 41.46725 | Cause of death: without complications |
| 7 | 77717843 | rs139273536 | C | T | 0.01114 | 0.4145 | 0.08728 | 2.04E-06 | 0.003785 | 36.27927 | No data |
| 11 | 10939004 | rs117349446 | G | A | 0.01824 | 0.3152 | 0.06646 | 2.11E-06 | 0.003558 | 34.09519 | No data |
| 11 | 30217503 | rs605765^*^ | C | T | 0.5904 | -0.08786 | 0.01546 | 1.32E-08 | 0.003734 | 35.78126 | Waist circumference; Weight; Number of operations |
| 11 | 30643977 | rs72892862 | C | A | 0.07563 | -0.1292 | 0.0277 | 3.10E-06 | 0.002334 | 22.33686 | No data |
| 12 | 96553382 | rs114210843 | T | C | 0.01985 | 0.2861 | 0.06155 | 3.35E-06 | 0.003185 | 30.5082 | No data |
| 13 | 97151567 | rs1999026 | A | C | 0.8922 | -0.1179 | 0.02534 | 3.28E-06 | 0.002674 | 25.59846 | No data |
| 15 | 68240748 | rs449487 | T | C | 0.6115 | -0.07948 | 0.01563 | 3.67E-07 | 0.003001 | 28.74426 | No data |
| 18 | 42501185 | rs17726110 | G | A | 0.09124 | -0.1145 | 0.02501 | 4.69E-06 | 0.002174 | 20.80334 | No data |
| 19 | 53609534 | rs8113629 | G | T | 0.4492 | -0.0697 | 0.01511 | 3.97E-06 | 0.002404 | 23.00843 | No data |

Supplementary Table 2 SNPs that were used as instrumental variables met the conditions: Pvalue<5 x 10^-6^ and F>10. ^*^ The secondary phenotypes of rs605765 obtained by PhenoScanner include waist circumference, weight and number of operations, some of which are associated with BMI, and BMI was identified as a confounder for lots of diseases. In the subsequent one-leave-out test, we focused on whether the MR Results were stable after removing this SNP.
