# Supplementary material for: Long-term outcomes of offspring from multiple gestations: a two-sample Mendelian randomization study on multi-system diseases using UK Biobank and FinnGen databases
Source: J Transl Med. 2023 Sep 8;21:608. doi: 10.1186/s12967-023-04423-w (PMC10492369; doi:10.1186/s12967-023-04423-w)
Supplement: Supplementary file 2 — Additional file 2: Table S2. Characteristics of each method used in this study. [file 12967_2023_4423_MOESM2_ESM.docx]

| **Method** | **Characteristic** |
| --- | --- |
| Inverse-variance weighted | The IVW method was primarily employed for fundamental causal estimates, which would provide the most precise results when all selected SNPs were valid IVs. The IVW method calculates a weighted average of Wald ratio estimates. |
| MR-Egger | Under the assumption of Instrument Strength Independent of Direct Effect (InSIDE), the MR-Egger regression executes a weighted linear regression and yields a consistent causal estimate, even though the genetic IVs are all invalid. (It exhibits low precision and is susceptible to outlying genetic variants. |
| Weighted median | The Weighted Median regression method, which does not demand the InSIDE hypothesis, calculates a weighted median of the Wald ratio estimates and is robust to horizontal pleiotropic bias. It is confirmed that the Weighted Median method has some advantages over the MR-Egger regression, as it provides lower type I error and higher causal estimate power. |
| Weighted Mode | The Weighted Mode method estimates the causal effect of the subset with the largest number of SNPs by clustering the SNPs into subsets resting on the resemblance of causal effects. |
| Simple Mode | The key feature of the simple mode of Mendelian randomization is that it uses a single genetic variant as the instrumental variable (IV) to estimate the causal effect of an exposure on an outcome. This genetic variant must be strongly associated with the exposure of interest and not directly related to the outcome, except through the exposure. The simple mode is generally easier to implement than the two-sample or multivariable modes of Mendelian randomization, but it may be less powerful due to the limited amount of variation explained by a single IV. Additionally, the simple mode assumes that the genetic variant satisfies the assumptions of instrumental variable analysis, such as independence from confounding and exclusion restriction. |
| MR-PRESSO | The MR Pleiotropy REsidual Sum and Outlier (MR-PRESSO) test was additionally conducted to evaluate whether pleiotropy was present. Its functions include detecting horizontal pleiotropy, correcting for horizontal pleiotropy by removing outlier, and determining whether there are substantial variations in the causal effects before and after outlier removal. |
