# Supplementary material for: Long-term outcomes of offspring from multiple gestations: a two-sample Mendelian randomization study on multi-system diseases using UK Biobank and FinnGen databases
Source: J Transl Med. 2023 Sep 8;21:608. doi: 10.1186/s12967-023-04423-w (PMC10492369; doi:10.1186/s12967-023-04423-w)
Supplement: Supplementary file 3 — Additional file 3: Table S3. Two-sample Mendelian randomization estimations showing the effects, heterogeneity and horizontal pleiotropy of multiple birth on the risk of mental illness. [file 12967_2023_4423_MOESM3_ESM.docx]

|  | Attention deficit hyperactivity disorder | | Depression | | Autism | | Bipolar affective disorder | | Cognitive impairment | | Schizophrenia | | Mood disorders | | Anxiety disorders | | Suicide or self-inflicted injury | |
| --- | --- | --- | --- | --- | --- | --- | --- | --- | --- | --- | --- | --- | --- | --- | --- | --- | --- | --- |
|  | FinnGen | UK Biobank | FinnGen | UK Biobank | FinnGen | UK Biobank | FinnGen | UK Biobank | FinnGen | UK Biobank | FinnGen | UK Biobank | FinnGen | UK Biobank | FinnGen | UK Biobank | FinnGen | UK Biobank |
| **Main analysis** |  |  |  |  |  |  |  |  |  |  |  |  |  |  |  |  |  |  |
| IVW |  |  |  |  |  |  |  |  |  |  |  |  |  |  |  |  |  |  |
| OR (95% CI) | 1.079  (0.892-1.306) | 0.588  (0.226-1.529) | 1.022  (0.977-1.069) | 1.071  (0.967-1.185) | 1.011  (0.677-1.510) | 0.948  (0.871-1.031) | 1.112  (0.996-1.243) | 1.032  (0.788-1.350) | NA | 1.131  (0.630-2.030) | 0.903  (0.765-1.065) | 0.852  (0.572-1.270) | 1.017  (0.970-1.065) | 1.050  (0.845-1.305) | 0.978  (0.925-1.034) | 1.092  (0.996-1.198) | 0.986  (0.793-1.227) | 1.087  (0.856-1.379) |
| P value | 0.434 | 0.276 | 0.347 | 0.187 | 0.958 | 0.211 | 0.059 | 0.820 | NA | 0.681 | 0.224 | 0.431 | 0.490 | 0.659 | 0.433 | 0.062 | 0.900 | 0.494 |
| MR Egger |  |  |  |  |  |  |  |  |  |  |  |  |  |  |  |  |  |  |
| OR (95% CI) | 1.134  (0.791-1.627) | 0.978  (0.126-7.563) | 1.056  (0.968-1.151) | 1.023  (0.823-1.271) | 0.783  (0.367-1.672) | 1.144  (0.983-1.331) | 1.121  (0.908-1.383) | 0.895  (0.510-1.571) | NA | 1.713  (0.497-5.898) | 0.956  (0.693-1.320) | 0.846  (0.358-2.003) | 1.053  (0.963-1.152) | 0.954  (0.606-1.501) | 1.004  (0.902-1.118) | 0.944  (0.778-1.145) | 0.941  (0.622-1.424) | 1.321  (0.799-2.185) |
| P value | 0.502 | 0.983 | 0.237 | 0.842 | 0.536 | 0.104 | 0.303 | 0.705 | NA | 0.407 | 0.789 | 0.710 | 0.275 | 0.840 | 0.942 | 0.565 | 0.777 | 0.296 |
| Weighted median |  |  |  |  |  |  |  |  |  |  |  |  |  |  |  |  |  |  |
| OR (95% CI) | 0.977  (0.743-1.285) | 0.624  (0.176-2.213) | 1.045  (0.982-1.113) | 1.039  (0.943-1.145) | 1.124  (0.626-2.019) | 0.972  (0.873-1.083) | 1.139  (0.979-1.326) | 1.079  (0.772-1.509) | NA | 1.520  (0.716-3.225) | 0.897  (0.705-1.142) | 0.951  (0.554-1.632) | 1.058  (0.991-1.131) | 1.019  (0.759-1.368) | 0.995  (0.924-1.070) | 1.094  (0.962-1.243) | 0.848  (0.625-1.150) | 1.000  (0.751-1.333) |
| P value | 0.869 | 0.624 | 0.168 | 0.435 | 0.695 | 0.612 | 0.092 | 0.655 | NA | 0.275 | 0.378 | 0.856 | 0.092 | 0.901 | 0.885 | 0.171 | 0.289 | 0.999 |
| Weighted mode |  |  |  |  |  |  |  |  |  |  |  |  |  |  |  |  |  |  |
| OR (95% CI) | 0.940  (0.669-1.320) | 0.354  (0.035-3.603) | 1.058  (0.956-1.171) | 1.047  (0.896-1.223) | 1.100  (0.511-2.371) | 0.949  (0.779-1.156) | 1.146  (0.948-1.385) | 1.057  (0.599-1.863) | NA | 2.290  (0.663-7.914) | 0.849  (0.596-1.208) | 0.916  (0.334-2.510) | 1.071  (0.978-1.173) | 0.977  (0.597-1.598) | 1.040  (0.944-1.146) | 1.098  (0.881-1.368) | 0.821  (0.530-1.272) | 0.980  (0.612-1.568) |
| P value | 0.724 | 0.394 | 0.289 | 0.569 | 0.810 | 0.610 | 0.178 | 0.852 | NA | 0.209 | 0.375 | 0.867 | 0.159 | 0.926 | 0.438 | 0.420 | 0.389 | 0.933 |
| Simple mode |  |  |  |  |  |  |  |  |  |  |  |  |  |  |  |  |  |  |
| OR (95% CI) | 0.947  (0.633-1.418) | 0.228  (0.018-2.867) | 0.960  (0.847-1.089) | 1.044  (0.874-1.247) | 0.984  (0.396-2.444) | 0.892  (0.719-1.106) | 1.059  (0.834-1.346) | 1.030  (0.553-1.920) | NA | 2.363  (0.606-9.219) | 0.902  (0.570-1.429) | 0.916  (0.361-2.323) | 1.060  (0.931-1.206) | 1.001  (0.593-1.690) | 0.898  (0.767-1.052) | 1.098  (0.875-1.376) | 0.916  (0.545-1.541) | 0.974  (0.612-1.549) |
| P value | 0.796 | 0.269 | 0.960 | 0.639 | 0.972 | 0.311 | 0.642 | 0.926 | NA | 0.234 | 0.666 | 0.856 | 0.392 | 0.998 | 0.200 | 0.432 | 0.746 | 0.913 |
| MR-PRESSO |  |  |  |  |  |  |  |  |  |  |  |  |  |  |  |  |  |  |
| OR (95% CI) | 1.079  (0.892-1.306) | 0.588  (0.226-1.529) | 1.022  (0.977-1.069) | 1.072  (0.981-1.163) | 1.011  (0.677-1.510) | 0.948  (0.871-1.031) | 1.112  (0.996-1.243) | 1.032  (0.788-1.350) | NA | 1.131  (0.630-2.030) | 0.903  (0.765-1.065) | 0.852  (0.572-1.270) | 1.017  (0.970-1.065) | 1.050  (0.845-1.305) | 0.978  (0.925-1.034) | 1.092  (0.996-1.198) | 0.986  (0.793-1.227) | 1.087  (0.856-1.379) |
| P value | 0.434 | 0.276 | 0.347 | 0.131 | 0.958 | 0.211 | 0.059 | 0.820 | NA | 0.681 | 0.224 | 0.431 | 0.490 | 0.659 | 0.433 | 0.062 | 0.900 | 0.494 |
| **Sensitivity analysis** |  |  |  |  |  |  |  |  |  |  |  |  |  |  |  |  |  |  |
| Cochran’s Q |  |  |  |  |  |  |  |  |  |  |  |  |  |  |  |  |  |  |
| Q-statistics | 16.005 | 17.270 | 17.080 | 41.856 | 12.221 | 21.723 | 11.170 | 4.219 | NA | 20.952 | 19.191 | 18.769 | 18.460 | 9.278 | 22.114 | 14.264 | 13.946 | 19.966 |
| Q_df | 17 | 16 | 17 | 16 | 17 | 16 | 17 | 16 | NA | 16 | 17 | 16 | 17 | 16 | 17 | 16 | 17 | 16 |
| P value | 0.523 | 0.368 | 0.449 | 4.15E-4 | 0.787 | 0.152 | 0.848 | 0.998 | NA | 0.180 | 0.318 | 0.281 | 0.360 | 0.902 | 0.180 | 0.579 | 0.671 | 0.222 |
| MR-Egger |  |  |  |  |  |  |  |  |  |  |  |  |  |  |  |  |  |  |
| Q-statistics | 15.902 | 16.922 | 16.315 | 41.248 | 11.616 | 14.080 | 11.164 | 3.902 | NA | 20.195 | 18.989 | 18.769 | 17.563 | 9.053 | 21.686 | 11.414 | 13.877 | 19.018 |
| Q_df | 16 | 15 | 16 | 15 | 16 | 15 | 16 | 15 | NA | 15 | 16 | 15 | 16 | 15 | 16 | 15 | 16 | 15 |
| P value | 0.460 | 0.324 | 0.431 | 2.93E-4 | 0.770 | 0.519 | 0.799 | 0.998 | NA | 0.165 | 0.269 | 0.224 | 0.350 | 0.875 | 0.154 | 0.723 | 0.608 | 0.213 |
| Egger intercept |  |  |  |  |  |  |  |  |  |  |  |  |  |  |  |  |  |  |
| Intercept | -7.21E-3 | -6.31E-2 | 4.64E-3 | 5.67E-3 | 3.67E-2 | 2.33E-2 | -1.07E-3 | 1.76E-2 | NA | -5.16E-2 | -8.22E-3 | 8.19E-4 | -5.00E-3 | 1.20E-2 | -3.74E-3 | 1.81E-2 | 6.70E-3 | -2.41E-2 |
| P value | 0.752 | 0.587 | 0.399 | 0.645 | 0.448 | 0.145 | 0.935 | 0.582 | NA | 0.465 | 0.685 | 0.987 | 0.380 | 0.642 | 0.582 | 0.112 | 0.797 | 0.401 |
| MR-PRESSO |  |  |  |  |  |  |  |  |  |  |  |  |  |  |  |  |  |  |
| P value | 0.554 | 0.386 | 0.433 | 8E-4 | 0.798 | 0.160 | 0.872 | 0.998 | NA | 0.189 | 0.310 | 0.282 | 0.324 | 0.903 | 0.171 | 0.597 | 0.670 | 0.226 |

Supplementary Table 4. Two-sample Mendelian randomization estimations showing the effects, heterogeneity and horizontal pleiotropy of multiple birth on the risk of mental illness.
