# Supplementary material for: Long-term outcomes of offspring from multiple gestations: a two-sample Mendelian randomization study on multi-system diseases using UK Biobank and FinnGen databases
Source: J Transl Med. 2023 Sep 8;21:608. doi: 10.1186/s12967-023-04423-w (PMC10492369; doi:10.1186/s12967-023-04423-w)
Supplement: Supplementary file 4 — Additional file 4: Table S4. Two-sample Mendelian randomization estimations showing the effects, heterogeneity and horizontal pleiotropy of multiple birth on the risk of nervous system disease. [file 12967_2023_4423_MOESM4_ESM.docx]

|  | Stroke | | Subarachnoid hemorrhage | | Transient ischaemic attack | | Epilepsy | | Migraine | | Alzheimer’s disease | | Cerebral palsy | | | Mental redardation |
| --- | --- | --- | --- | --- | --- | --- | --- | --- | --- | --- | --- | --- | --- | --- | --- | --- |
|  | FinnGen | UK Biobank | FinnGen | UK Biobank | FinnGen | UK Biobank | FinnGen | UK Biobank | FinnGen | UK Biobank | FinnGen | UK Biobank | FinnGen | UK Biobank | UK Biobank (Infantile) | FinnGen |
| **Main analysis** |  |  |  |  |  |  |  |  |  |  |  |  |  |  |  |  |
| IVW |  |  |  |  |  |  |  |  |  |  |  |  |  |  |  |  |
| OR (95% CI) | 1.028  (0.970-1.089) | 0.957  (0.852-1.075) | 0.999  (0.811-1.232) | 0.805  (0.583-1.112) | 1.042  (0.974-1.113) | 1.037  (0.849-1.267) | 1.037  (0.931-1.155) | 0.889  (0.765-1.034) | 1.013  (0.947-1.083) | 1.049  (0.898-1.227) | 1.013  (0.918-1.117) | 1.109  (0.805-1.529) | 0.942  (0.568-1.563) | 1.235  (0.532-2.867) | 1.536  (0.748-3.152) | 0.976  (0.717-1.328) |
| P value | 0.349 | 0.458 | 0.995 | 0.188 | 0.231 | 0.719 | 0.505 | 0.128 | 0.708 | 0.544 | 0.802 | 0.525 | 0.817 | 0.624 | 0.242 | 0.877 |
| MR Egger |  |  |  |  |  |  |  |  |  |  |  |  |  |  |  |  |
| OR (95% CI) | 0.980  (0.879-1.094) | 0.930  (0.729-1.186) | 0.921  (0.614-1.383) | 0.961  (0.483-1.911) | 0.926  (0.816-1.051) | 1.330  (0.885-2.000) | 1.123  (0.914-1.380) | 0.911  (0.658-1.262) | 1.019  (0.897-1.157) | 1.022  (0.738-1.416) | 0.922  (0.765-1.112) | 0.842  (0.431-1.643) | 0.565  (0.219-1.456) | 1.454  (0.236-8.941) | 5.191  (1.169-23.051) | 1.257  (0.701-2.253) |
| P value | 0.727 | 0.569 | 0.698 | 0.910 | 0.250 | 0.190 | 0.286 | 0.583 | 0.773 | 0.895 | 0.408 | 0.621 | 0.254 | 0.692 | 0.047 | 0.454 |
| Weighted median |  |  |  |  |  |  |  |  |  |  |  |  |  |  |  |  |
| OR (95% CI) | 1.054  (0.978-1.136) | 0.972  (0.830-1.139) | 0.919  (0.711-1.188) | 0.861  (0.566-1.309) | 1.036  (0.946-1.133) | 1.065  (0.838-1.354) | 1.059  (0.940-1.193) | 1.001  (0.824-1.216) | 0.988  (0.894-1.091) | 0.987  (0.794-1.226) | 0.999  (0.868-1.149) | 1.109  (0.718-1.715) | 1.191  (0.641-2.212) | 1.266  (0.411-3.899) | 1.187  (0.423-3.330) | 1.069  (0.721-1.587) |
| P value | 0.171 | 0.728 | 0.919 | 0.483 | 0.449 | 0.607 | 0.346 | 0.990 | 0.810 | 0.905 | 0.985 | 0.641 | 0.581 | 0.681 | 0.745 | 0.739 |
| Weighted mode |  |  |  |  |  |  |  |  |  |  |  |  |  |  |  |  |
| OR (95% CI) | 1.043  (0.954-1.139) | 0.996  (0.758-1.309) | 0.855  (0.649-1.125) | 0.906  (0.433-1.895) | 0.995  (0.886-1.118) | 1.158  (0.750-1.787) | 1.064  (0.927-1.221) | 0.996  (0.670-1.483) | 0.982  (0.875-1.103) | 0.975  (0.642-1.480) | 1.013  (0.840-1.222) | 1.205  (0.585-2.480) | 1.792  (0.788-4.080) | 1.843  (0.244-13.934) | 1.035  (0.200-5.371) | 1.230  (0.747-2.024) |
| P value | 0.367 | 0.976 | 0.278 | 0.797 | 0.935 | 0.518 | 0.390 | 0.986 | 0.764 | 0.906 | 0.895 | 0.620 | 0.182 | 0.562 | 0.968 | 0.427 |
| Simple mode |  |  |  |  |  |  |  |  |  |  |  |  |  |  |  |  |
| OR (95% CI) | 1.021  (0.902-1.155) | 0.977  (0.735-1.299) | 0.765  (0.518-1.132) | 0.892  (0.421-1.890) | 1.014  (0.866-1.188) | 1.164  (0.746-1.818) | 1.069  (0.884-1.292) | 0.996  (0.674-1.473) | 0.971  (0.835-1.128) | 0.971  (0.656-1.438) | 1.117  (0.874-1.426) | 1.259  (0.564-2.808) | 2.057  (0.670-6.314) | 2.069  (0.275-15.570) | 1.077  (0.198-5.852) | 0.921  (0.425-1.997) |
| P value | 0.751 | 0.876 | 0.198 | 0.770 | 0.863 | 0.513 | 0.502 | 0.986 | 0.704 | 0.887 | 0.389 | 0.582 | 0.224 | 0.490 | 0.932 | 0.837 |
| MR-PRESSO |  |  |  |  |  |  |  |  |  |  |  |  |  |  |  |  |
| OR (95% CI) | 1.028  (0.970-1.089) | 0.957  (0.852-1.075) | 0.999  (0.811-1.232) | 0.805  (0.583-1.112) | 1.042  (0.974-1.113) | 1.037  (0.849-1.267) | 1.037  (0.931-1.155) | 0.889  (0.765-1.034) | 1.013  (0.947-1.083) | 1.049  (0.898-1.227) | 1.013  (0.918-1.117) | 1.109  (0.805-1.529) | 0.942  (0.568-1.563) | 1.235  (0.532-2.867) | 1.536  (0.748-3.152) | 0.976  (0.717-1.328) |
| P value | 0.349 | 0.458 | 0.995 | 0.188 | 0.231 | 0.719 | 0.505 | 0.128 | 0.708 | 0.544 | 0.802 | 0.525 | 0.817 | 0.624 | 0.242 | 0.877 |
| **Sensitivity analysis** |  |  |  |  |  |  |  |  |  |  |  |  |  |  |  |  |
| Cochran’s Q |  |  |  |  |  |  |  |  |  |  |  |  |  |  |  |  |
| Q-statistics | 21.607 | 14.909 | 25.795 | 19.249 | 16.395 | 23.711 | 30.248 | 18.513 | 14.327 | 12.657 | 12.882 | 10.441 | 29.918 | 19.283 | 16.263 | 19.762 |
| Q_df | 17 | 16 | 17 | 16 | 17 | 16 | 17 | 16 | 17 | 16 | 17 | 16 | 17 | 16 | 16 | 17 |
| P value | 0.200 | 0.531 | 0.078 | 0.256 | 0.496 | 0.096 | 0.025 | 0.295 | 0.644 | 0.698 | 0.744 | 0.843 | 0.027 | 0.254 | 0.435 | 0.287 |
| MR-Egger |  |  |  |  |  |  |  |  |  |  |  |  |  |  |  |  |
| Q-statistics | 20.339 | 14.842 | 25.457 | 18.837 | 11.784 | 21.100 | 28.825 | 18.479 | 14.314 | 12.625 | 11.549 | 9.592 | 27.274 | 19.232 | 12.937 | 18.600 |
| Q_df | 16 | 15 | 16 | 15 | 16 | 15 | 16 | 15 | 16 | 15 | 16 | 15 | 16 | 15 | 15 | 16 |
| P value | 0.205 | 0.463 | 0.062 | 0.221 | 0.759 | 0.134 | 0.025 | 0.238 | 0.575 | 0.631 | 0.774 | 0.845 | 0.039 | 0.203 | 0.607 | 0.290 |
| Egger intercept |  |  |  |  |  |  |  |  |  |  |  |  |  |  |  |  |
| Intercept | 6.76E-3 | 3.50E-3 | 1.16E-2 | -2.19E-2 | 1.68E-2 | -3.09E-2 | -1.13E-2 | -3.00E-3 | -8.92E-4 | 3.24E-3 | 1.34E-2 | 3.43E-2 | 7.29E-2 | -2.02E-2 | -1.51E-1 | -3.62E-2 |
| P value | 0.333 | 0.799 | 0.651 | 0.575 | 0.047 | 0.193 | 0.387 | 0.871 | 0.911 | 0.860 | 0.265 | 0.371 | 0.231 | 0.844 | 0.088 | 0.332 |
| MR-PRESSO |  |  |  |  |  |  |  |  |  |  |  |  |  |  |  |  |
| P value | 0.222 | 0.549 | 0.103 | 0.257 | 0.493 | 0.109 | 0.034 | 0.309 | 0.676 | 0.692 | 0.750 | 0.852 | 0.035 | 0.267 | 0.431 | 0.292 |

Supplementary Table 5. Two-sample Mendelian randomization estimations showing the effects, heterogeneity and horizontal pleiotropy of multiple birth on the risk of nervous system disease.
