# Supplementary material for: Long-term outcomes of offspring from multiple gestations: a two-sample Mendelian randomization study on multi-system diseases using UK Biobank and FinnGen databases
Source: J Transl Med. 2023 Sep 8;21:608. doi: 10.1186/s12967-023-04423-w (PMC10492369; doi:10.1186/s12967-023-04423-w)
Supplement: Supplementary file 5 — Additional file 5: Table S5. Two-sample Mendelian randomization estimations showing the effects, heterogeneity and horizontal pleiotropy of multiple birth on the risk of cardiovascular system disease. [file 12967_2023_4423_MOESM5_ESM.docx]

|  | Arterial hypertension | | Atrial fibrillation and flutter | | Ichaemic heart disease | | Coronary heart disease | | Cardiomyopathy | | Myocardial infarction | | Pulmonary embolism | | Deep vein thrombosis | |
| --- | --- | --- | --- | --- | --- | --- | --- | --- | --- | --- | --- | --- | --- | --- | --- | --- |
|  | FinnGen | UK Biobank | FinnGen | UK Biobank | FinnGen | UK Biobank | FinnGen | UK Biobank | FinnGen | UK Biobank | FinnGen | UK Biobank | FinnGen | UK Biobank | FinnGen | UK Biobank |
| **Main analysis** |  |  |  |  |  |  |  |  |  |  |  |  |  |  |  |  |
| IVW |  |  |  |  |  |  |  |  |  |  |  |  |  |  |  |  |
| OR (95% CI) | 1.007  (0.958-1.058) | 1.004  (0.947-1.065) | 0.990  (0.933-1.051) | 1.027  (0.956-1.103) | 1.020  (0.957-1.087) | 1.036  (0.964-1.113) | 1.044  (0.972-1.122) | 1.045  (0.967-1.129) | 0.994  (0.888-1.112) | 1.007  (0.803-1.264) | 1.056  (0.970-1.151) | 1.049  (0.966-1.139) | 1.039  (0.949-1.137) | 1.081  (0.944-1.239) | 1.101  (0.992-1.223) | 1.105  (0.978-1.249) |
| P value | 0.783 | 0.890 | 0.740 | 0.470 | 0.545 | 0.342 | 0.234 | 0.269 | 0.912 | 0.950 | 0.209 | 0.255 | 0.406 | 0.260 | 0.071 | 0.111 |
| MR Egger |  |  |  |  |  |  |  |  |  |  |  |  |  |  |  |  |
| OR (95% CI) | 1.016  (0.922-1.119) | 0.948  (0.838-1.072) | 0.911  (0.813-1.019) | 1.078  (0.927-1.252) | 1.006  (0.888-1.139) | 0.997  (0.855-1.162) | 1.003  (0.874-1.152) | 1.019  (0.862-1.204) | 1.067  (0.861-1.321) | 1.331  (0.829-2.137) | 1.010  (0.856-1.192) | 0.989  (0.831-1.177) | 1.090  (0.918-1.294) | 1.079  (0.806-1.444) | 1.166  (0.954-1.426) | 0.849  (0.684-1.053) |
| P value | 0.756 | 0.409 | 0.123 | 0.345 | 0.927 | 0.967 | 0.965 | 0.832 | 0.562 | 0.256 | 0.906 | 0.904 | 0.340 | 0.617 | 0.154 | 0.157 |
| Weighted median |  |  |  |  |  |  |  |  |  |  |  |  |  |  |  |  |
| OR (95% CI) | 1.039  (0.982-1.098) | 1.018  (0.960-1.079) | 0.998  (0.920-1.082) | 1.023  (0.924-1.132) | 1.048  (0.985-1.115) | 1.043  (0.956-1.138) | 1.074  (0.998-1.155) | 1.088  (0.989-1.204) | 0.988  (0.840-1.161) | 0.934  (0.691-1.262) | 1.077  (0.981-1.182) | 1.094  (0.980-1.222) | 1.051  (0.926-1.192) | 1.094  (0.906-1.320) | 1.037  (0.907-1.187) | 1.046  (0.901-1.215) |
| P value | 0.184 | 0.560 | 0.956 | 0.665 | 0.141 | 0.342 | 0.056 | 0.082 | 0.879 | 0.657 | 0.118 | 0.111 | 0.441 | 0.352 | 0.154 | 0.553 |
| Weighted mode |  |  |  |  |  |  |  |  |  |  |  |  |  |  |  |  |
| OR (95% CI) | 1.055  (0.978-1.139) | 1.026  (0.934-1.126) | 1.004  (0.907-1.111) | 1.017  (0.849-1.217) | 1.069  (0.996-1.147) | 0.913  (0.756-1.102) | 1.087  (1.005-1.176) | 1.122  (0.948-1.328) | 0.945  (0.776-1.150) | 0.895  (0.524-1.529) | 1.086  (0.975-1.209) | 1.095  (0.905-1.326) | 1.058  (0.914-1.225) | 1.095  (0.770-1.557) | 1.023  (0.863-1.212) | 0.998  (0.775-1.286) |
| P value | 0.184 | 0.601 | 0.939 | 0.860 | 0.081 | 0.355 | 0.053 | 0.198 | 0.580 | 0.691 | 0.151 | 0.364 | 0.457 | 0.622 | 0.798 | 0.990 |
| Simple mode |  |  |  |  |  |  |  |  |  |  |  |  |  |  |  |  |
| OR (95% CI) | 1.048  (0.940-1.169) | 1.026  (0.928-1.133) | 1.019  (0.899-1.154) | 1.020  (0.839-1.239) | 1.092  (1.005-1.187) | 0.909  (0.743-1.112) | 1.107  (0.993-1.234) | 1.126  (0.933-1.360) | 1.191  (0.907-1.565) | 0.871  (0.476-1.595) | 1.060  (0.928-1.210) | 1.091  (0.903-1.317) | 1.062  (0.887-1.271) | 1.095  (0.779-1.538) | 1.023  (0.843-1.241) | 0.992  (0.764-1.287) |
| P value | 0.412 | 0.624 | 0.775 | 0.846 | 0.054 | 0.367 | 0.084 | 0.234 | 0.226 | 0.871 | 0.403 | 0.380 | 0.523 | 0.609 | 0.822 | 0.951 |
| MR-PRESSO |  |  |  |  |  |  |  |  |  |  |  |  |  |  |  |  |
| OR (95% CI) | 1.007  (0.958-1.058) | 0.987  (0.938-1.037) | 0.990  (0.933-1.051) | 1.027  (0.956-1.103) | 1.038  (0.979-1.096) | 1.036  (0.964-1.113) | 1.065  (0.998-1.132) | 1.045  (0.967-1.129) | 0.994  (0.888-1.112) | 1.007  (0.803-1.264) | 1.056  (0.970-1.151) | 1.049  (0.966-1.139) | 1.039  (0.949-1.137) | 1.081  (0.944-1.239) | 1.101  (0.992-1.223) | 1.105  (0.978-1.249) |
| P value | 0.783 | 0.630 | 0.740 | 0.470 | 0.217 | 0.342 | 0.068 | 0.269 | 0.912 | 0.950 | 0.209 | 0.255 | 0.406 | 0.260 | 0.071 | 0.111 |
| **Sensitivity analysis** |  |  |  |  |  |  |  |  |  |  |  |  |  |  |  |  |
| Cochran’s Q |  |  |  |  |  |  |  |  |  |  |  |  |  |  |  |  |
| Q-statistics | 35.135 | 38.842 | 11.788 | 12.173 | 37.976 | 24.395 | 36.871 | 22.134 | 12.530 | 11.076 | 30.449 | 16.766 | 12.529 | 15.751 | 22.567 | 22.879 |
| Q_df | 17 | 16 | 17 | 16 | 17 | 16 | 17 | 16 | 17 | 16 | 17 | 16 | 17 | 16 | 17 | 16 |
| P value | 0.006 | 0.001 | 0.813 | 0.732 | 0.002 | 0.081 | 0.004 | 0.139 | 0.767 | 0.805 | 0.023 | 0.401 | 0.767 | 0.470 | 0.164 | 0.117 |
| MR-Egger |  |  |  |  |  |  |  |  |  |  |  |  |  |  |  |  |
| Q-statistics | 35.041 | 36.211 | 8.868 | 11.660 | 37.823 | 23.904 | 35.855 | 21.964 | 11.946 | 9.353 | 29.731 | 16.157 | 12.117 | 15.751 | 21.969 | 15.293 |
| Q_df | 16 | 15 | 16 | 15 | 16 | 15 | 16 | 15 | 16 | 15 | 16 | 15 | 16 | 15 | 16 | 15 |
| P value | 0.004 | 0.002 | 0.919 | 0.705 | 0.002 | 0.067 | 0.003 | 0.109 | 0.748 | 0.858 | 0.019 | 0.372 | 0.736 | 0.399 | 0.144 | 0.430 |
| Egger intercept |  |  |  |  |  |  |  |  |  |  |  |  |  |  |  |  |
| Intercept | -1.24E-3 | 7.13E-3 | 1.19E-2 | -5.98E-3 | 1.96E-3 | 4.75E-3 | 5.76E-3 | 3.17E-3 | -1.01E-2 | -3.46E-2 | 6.37E-3 | 7.23E-3 | -6.81E-3 | 2.97E-4 | -8.25E-3 | 3.27E-2 |
| P value | 0.839 | 0.313 | 0.107 | 0.485 | 0.802 | 0.587 | 0.510 | 0.738 | 0.456 | 0.209 | 0.543 | 0.464 | 0.530 | 0.986 | 0.519 | 0.160 |
| MR-PRESSO |  |  |  |  |  |  |  |  |  |  |  |  |  |  |  |  |
| P value | 0.007 | 0.002 | 0.831 | 0.739 | 0.003 | 0.084 | 0.005 | 0.144 | 0.780 | 0.806 | 0.038 | 0.401 | 0.800 | 0.488 | 0.202 | 0.127 |

Supplementary Table 6. Two-sample Mendelian randomization estimations showing the effects, heterogeneity and horizontal pleiotropy of multiple birth on the risk of cardiovascular system disease.
