# Supplementary material for: Long-term outcomes of offspring from multiple gestations: a two-sample Mendelian randomization study on multi-system diseases using UK Biobank and FinnGen databases
Source: J Transl Med. 2023 Sep 8;21:608. doi: 10.1186/s12967-023-04423-w (PMC10492369; doi:10.1186/s12967-023-04423-w)
Supplement: Supplementary file 6 — Additional file 6: Table S6. Two-sample Mendelian randomization estimations showing the effects, heterogeneity and horizontal pleiotropy of multiple birth on the risk of respiratory system disease. [file 12967_2023_4423_MOESM6_ESM.docx]

|  | Chronic obstructive pulmonary disease | | Asthma | | Bronchitis | | Tuberculosis | |
| --- | --- | --- | --- | --- | --- | --- | --- | --- |
|  | FinnGen | UK Biobank | FinnGen | UK Biobank | FinnGen | UK Biobank | FinnGen | UK Biobank |
| **Main analysis** |  |  |  |  |  |  |  |  |
| IVW |  |  |  |  |  |  |  |  |
| OR (95% CI) | 0.933  (0.873-0.998) | 0.914  (0.710-1.176) | 0.969  (0.919-1.023) | 1.018  (0.967-1.071) | 0.991  (0.952-1.031) | 1.090  (0.924-1.286) | 0.887  (0.747-1.054) | 1.012  (0.822-1.245) |
| P value | 0.045 | 0.484 | 0.258 | 0.504 | 0.646 | 0.308 | 0.172 | 0.914 |
| MR Egger |  |  |  |  |  |  |  |  |
| OR (95% CI) | 0.848  (0.747-0.964) | 1.270  (0.763-2.116) | 0.973  (0.876-1.081) | 1.071  (0.964-1.191) | 1.014  (0.941-1.093) | 1.254  (0.888-1.773) | 0.965  (0.696-1.337) | 0.996  (0.636-1.560) |
| P value | 0.022 | 0.373 | 0.613 | 0.222 | 0.715 | 0.219 | 0.832 | 0.988 |
| Weighted median |  |  |  |  |  |  |  |  |
| OR (95% CI) | 0.920  (0.835-1.014) | 0.954  (0.688-1.324) | 0.961  (0.897-1.030) | 1.047  (0.978-1.120) | 0.992  (0.937-1.049) | 1.029  (0.819-1.292) | 1.001  (0.784-1.278) | 0.918  (0.687-1.227) |
| P value | 0.092 | 0.779 | 0.263 | 0.187 | 0.773 | 0.806 | 0.993 | 0.563 |
| Weighted mode |  |  |  |  |  |  |  |  |
| OR (95% CI) | 0.888  (0.773-1.020) | 0.914  (0.511-1.635) | 0.970  (0.887-1.060) | 1.058  (0.949-1.180) | 0.999  (0.928-1.075) | 1.004  (0.708-1.424) | 1.078  (0.778-1.494) | 0.903  (0.544-1.498) |
| P value | 0.110 | 0.765 | 0.509 | 0.322 | 0.969 | 0.982 | 0.658 | 0.698 |
| Simple mode |  |  |  |  |  |  |  |  |
| OR (95% CI) | 0.866  (0.725-1.034) | 0.882  (0.511-1.525) | 0.992  (0.889-1.106) | 1.060  (0.945-1.188) | 1.015  (0.927-1.112) | 1.002  (0.699-1.434) | 0.714  (0.440-1.157) | 0.908  (0.549-1.502) |
| P value | 0.130 | 0.660 | 0.882 | 0.336 | 0.751 | 0.993 | 0.189 | 0.712 |
| MR-PRESSO |  |  |  |  |  |  |  |  |
| OR (95% CI) | 0.933  (0.873-0.998) | 0.914  (0.710-1.176) | 0.969  (0.919-1.023) | 1.018  (0.967-1.071) | 0.991  (0.952-1.031) | 1.090  (0.924-1.286) | 0.887  (0.747-1.054) | 1.012  (0.822-1.245) |
| P value | 0.045 | 0.484 | 0.258 | 0.504 | 0.646 | 0.308 | 0.172 | 0.914 |
| **Sensitivity analysis** |  |  |  |  |  |  |  |  |
| Cochran’s Q |  |  |  |  |  |  |  |  |
| Q-statistics | 14.419 | 18.067 | 23.112 | 18.322 | 10.292 | 5.536 | 15.396 | 16.564 |
| Q_df | 17 | 16 | 17 | 16 | 17 | 16 | 17 | 16 |
| P value | 0.637 | 0.320 | 0.146 | 0.305 | 0.891 | 0.992 | 0.567 | 0.414 |
| MR-Egger |  |  |  |  |  |  |  |  |
| Q-statistics | 11.413 | 15.873 | 23.105 | 16.987 | 9.771 | 4.714 | 15.045 | 16.558 |
| Q_df | 16 | 15 | 16 | 15 | 16 | 15 | 16 | 15 |
| P value | 0.783 | 0.391 | 0.111 | 0.320 | 0.878 | 0.994 | 0.521 | 0.346 |
| Egger intercept |  |  |  |  |  |  |  |  |
| Intercept | 1.37E-2 | -4.08E-2 | -4.64E-4 | -6.39E-3 | -3.35E-3 | -1.74E-2 | -1.20E-2 | 1.87E-3 |
| P value | 0.102 | 0.170 | 0.944 | 0.295 | 0.481 | 0.379 | 0.562 | 0.941 |
| MR-PRESSO |  |  |  |  |  |  |  |  |
| P value | 0.646 | 0.331 | 0.158 | 0.331 | 0.898 | 0.993 | 0.532 | 0.420 |

Supplementary Table 7. Two-sample Mendelian randomization estimations showing the effects, heterogeneity and horizontal pleiotropy of multiple birth on the risk of respiratory system disease.
