# Supplementary material for: Long-term outcomes of offspring from multiple gestations: a two-sample Mendelian randomization study on multi-system diseases using UK Biobank and FinnGen databases
Source: J Transl Med. 2023 Sep 8;21:608. doi: 10.1186/s12967-023-04423-w (PMC10492369; doi:10.1186/s12967-023-04423-w)
Supplement: Supplementary file 7 — Additional file 7: Table S7. Two-sample Mendelian randomization estimations showing the effects, heterogeneity and horizontal pleiotropy of multiple birth on the risk of digestive system disease. [file 12967_2023_4423_MOESM7_ESM.docx]

|  | Gastric ulcer | | Duodenal ulcer | | Crohn disease | | Fibrosis and cirrhosis of liver | | Gastritis | | |
| --- | --- | --- | --- | --- | --- | --- | --- | --- | --- | --- | --- |
|  | FinnGen | UK Biobank | FinnGen | UK Biobank | FinnGen | UK Biobank | FinnGen | UK Biobank | FinnGen (chronic) | FinnGen (acute) | UK Biobank (acute) |
| **Main analysis** |  |  |  |  |  |  |  |  |  |  |  |
| IVW |  |  |  |  |  |  |  |  |  |  |  |
| OR (95% CI) | 1.003  (0.891-1.130) | 0.951  (0.837-1.081) | 0.853  (0.738-0.986) | 1.126  (0.966-1.314) | 1.119  (0.906-1.383) | 0.997  (0.809-1.228) | 1.093  (0.855-1.399) | 1.097  (0.751-1.601) | 1.101  (1.008-1.204) | 0.994  (0.834-1.185) | 0.966  (0.757-1.232) |
| P value | 0.954 | 0.443 | 0.031 | 0.130 | 0.295 | 0.977 | 0.477 | 0.632 | 0.033 | 0.946 | 0.778 |
| MR Egger |  |  |  |  |  |  |  |  |  |  |  |
| OR (95% CI) | 0.989  (0.785-1.247) | 0.693  (0.530-0.906) | 0.801  (0.609-1.054) | 1.137  (0.824-1.568) | 0.922  (0.619-1.372) | 1.053  (0.682-1.626) | 0.918  (0.573-1.472) | 0.899  (0.401-2.016) | 1.170  (0.989-1.384) | 1.037  (0.740-1.455) | 0.729  (0.438-1.211) |
| P value | 0.929 | 0.017 | 0.133 | 0.446 | 0.692 | 0.820 | 0.728 | 0.800 | 0.086 | 0.834 | 0.240 |
| Weighted median |  |  |  |  |  |  |  |  |  |  |  |
| OR (95% CI) | 0.979  (0.835-1.147) | 0.956  (0.805-1.135) | 0.853  (0.702-1.036) | 1.094  (0.885-1.353) | 1.036  (0.775-1.384) | 0.960  (0.713-1.291) | 1.113  (0.824-1.503) | 0.920  (0.622-1.361) | 1.102  (0.971-1.252) | 0.985  (0.768-1.263) | 0.942  (0.670-1.326) |
| P value | 0.791 | 0.607 | 0.109 | 0.405 | 0.812 | 0.785 | 0.486 | 0.677 | 0.132 | 0.907 | 0.733 |
| Weighted mode |  |  |  |  |  |  |  |  |  |  |  |
| OR (95% CI) | 0.966  (0.784-1.191) | 0.938  (0.677-1.300) | 0.842  (0.654-1.083) | 0.999  (0.682-1.463) | 0.982  (0.694-1.391) | 0.831  (0.472-1.462) | 1.135  (0.732-1.759) | 0.862  (0.491-1.513) | 1.100  (0.939-1.290) | 1.055  (0.767-1.452) | 0.873  (0.470-1.622) |
| P value | 0.753 | 0.705 | 0.198 | 0.995 | 0.921 | 0.529 | 0.579 | 0.612 | 0.254 | 0.746 | 0.673 |
| Simple mode |  |  |  |  |  |  |  |  |  |  |  |
| OR (95% CI) | 0.952  (0.751-1.207) | 0.839  (0.605-1.165) | 0.898  (0.653-1.234) | 1.010  (0.661-1.543) | 1.029  (0.657-1.614) | 0.819  (0.468-1.431) | 1.311  (0.748-2.297) | 0.855  (0.463-1.578) | 1.103  (0.910-1.338) | 1.009  (0.654-1.556) | 0.873  (0.457-1.666) |
| P value | 0.690 | 0.310 | 0.515 | 0.963 | 0.901 | 0.493 | 0.357 | 0.623 | 0.333 | 0.969 | 0.686 |
| MR-PRESSO |  |  |  |  |  |  |  |  |  |  |  |
| OR (95% CI) | 1.003  (0.891-1.130) | 0.951  (0.837-1.081) | 0.853  (0.738-0.986) | 1.126  (0.966-1.314) | 1.119  (0.906-1.383) | 0.997  (0.809-1.228) | 1.093  (0.855-1.399) | 1.214  (0.875-1.552) | 1.101  (1.008-1.204) | 0.994  (0.834-1.185) | 0.966  (0.757-1.232) |
| P value | 0.954 | 0.443 | 0.031 | 0.130 | 0.295 | 0.977 | 0.477 | 0.280 | 0.033 | 0.946 | 0.778 |
| **Sensitivity analysis** |  |  |  |  |  |  |  |  |  |  |  |
| Cochran’s Q |  |  |  |  |  |  |  |  |  |  |  |
| Q-statistics | 18.890 | 14.062 | 12.782 | 13.078 | 17.341 | 12.154 | 25.558 | 31.018 | 8.239 | 16.459 | 14.712 |
| Q_df | 17 | 16 | 17 | 16 | 17 | 16 | 17 | 16 | 17 | 17 | 16 |
| P value | 0.335 | 0.594 | 0.751 | 0.667 | 0.432 | 0.733 | 0.083 | 0.013 | 0.961 | 0.492 | 0.546 |
| MR-Egger |  |  |  |  |  |  |  |  |  |  |  |
| Q-statistics | 18.866 | 7.107 | 12.507 | 13.074 | 16.063 | 12.076 | 24.449 | 30.408 | 7.557 | 16.372 | 13.177 |
| Q_df | 16 | 15 | 16 | 15 | 16 | 15 | 16 | 15 | 16 | 16 | 15 |
| P value | 0.276 | 0.955 | 0.708 | 0.597 | 0.449 | 0.673 | 0.080 | 0.011 | 0.961 | 0.427 | 0.589 |
| Egger intercept |  |  |  |  |  |  |  |  |  |  |  |
| Intercept | 2.03E-3 | 3.93E-2 | 8.91E-3 | -1.16E-3 | 2.78E-2 | -6.75E-3 | 2.49E-2 | 2.46E-2 | -8.60E-3 | -6.09E-3 | 3.50E-2 |
| P value | 0.889 | 0.019 | 0.607 | 0.949 | 0.276 | 0.784 | 0.407 | 0.592 | 0.421 | 0.774 | 0.234 |
| MR-PRESSO |  |  |  |  |  |  |  |  |  |  |  |
| P value | 0.365 | 0.603 | 0.785 | 0.675 | 0.438 | 0.730 | 0.081 | 0.015 | 0.970 | 0.507 | 0.562 |

Supplementary Table 8. Two-sample Mendelian randomization estimations showing the effects, heterogeneity and horizontal pleiotropy of multiple birth on the risk of digestive system disease.
