# Supplementary material for: Long-term outcomes of offspring from multiple gestations: a two-sample Mendelian randomization study on multi-system diseases using UK Biobank and FinnGen databases
Source: J Transl Med. 2023 Sep 8;21:608. doi: 10.1186/s12967-023-04423-w (PMC10492369; doi:10.1186/s12967-023-04423-w)
Supplement: Supplementary file 8 — Additional file 8: Table S8. Two-sample Mendelian randomization estimations showing the effects, heterogeneity and horizontal pleiotropy of multiple birth on the risk of endocrine system disease. [file 12967_2023_4423_MOESM8_ESM.docx]

|  | Thyrotoxicosis | | Hypothyroidism | | Thyroiditis | | Type 1 Diabetes | | Type 2 Diabetes | | Obesity | | Gout | |
| --- | --- | --- | --- | --- | --- | --- | --- | --- | --- | --- | --- | --- | --- | --- |
|  | FinnGen | UK Biobank | FinnGen | UK Biobank | FinnGen | UK Biobank | FinnGen | UK Biobank | FinnGen | UK Biobank | FinnGen | UK Biobank | FinnGen | UK Biobank |
| **Main analysis** |  |  |  |  |  |  |  |  |  |  |  |  |  |  |
| IVW |  |  |  |  |  |  |  |  |  |  |  |  |  |  |
| OR (95% CI) | 1.028  (0.920-1.149) | 1.084  (0.917-1.281) | 1.016  (0.970-1.064) | 1.022  (0.953-1.097) | 0.927  (0.724-1.188) | 0.920  (0.560-1.511) | 0.932  (0.839-1.036) | 1.020  (0.866-1.202) | 0.967  (0.903-1.036) | 1.040  (0.930-1.162) | 0.973  (0.906-1.044) | 1.024  (0.923-1.137) | 1.069  (0.970-1.178) | 1.052  (0.880-1.257) |
| P value | 0.627 | 0.345 | 0.509 | 0.542 | 0.550 | 0.741 | 0.193 | 0.809 | 0.338 | 0.495 | 0.445 | 0.650 | 0.179 | 0.580 |
| MR Egger |  |  |  |  |  |  |  |  |  |  |  |  |  |  |
| OR (95% CI) | 0.999  (0.804-1.242) | 0.998  (0.699-1.427) | 1.032  (0.942-1.129) | 1.056  (0.911-1.224) | 0.631  (0.409-0.971) | 0.867  (0.301-2.499) | 0.994  (0.812-1.217) | 0.835  (0.593-1.175) | 0.966  (0.845-1.104) | 0.860  (0.694-1.065) | 1.047  (0.917-1.194) | 0.994  (0.795-1.244) | 1.068  (0.888-1.285) | 0.917  (0.630-1.337) |
| P value | 0.994 | 0.994 | 0.512 | 0.479 | 0.053 | 0.795 | 0.958 | 0.317 | 0.616 | 0.187 | 0.507 | 0.962 | 0.493 | 0.660 |
| Weighted median |  |  |  |  |  |  |  |  |  |  |  |  |  |  |
| OR (95% CI) | 1.084  (0.937-1.253) | 1.199  (0.947-1.519) | 1.004  (0.939-1.073) | 1.004  (0.914-1.104) | 0.820  (0.613-1.097) | 1.011  (0.497-2.057) | 0.920  (0.802-1.055) | 0.985  (0.781-1.243) | 1.015  (0.937-1.099) | 0.982  (0.884-1.090) | 1.014  (0.920-1.117) | 1.018  (0.905-1.145) | 1.065  (0.925-1.226) | 1.139  (0.877-1.481) |
| P value | 0.279 | 0.132 | 0.913 | 0.930 | 0.181 | 0.977 | 0.232 | 0.902 | 0.710 | 0.728 | 0.786 | 0.768 | 0.379 | 0.329 |
| Weighted mode |  |  |  |  |  |  |  |  |  |  |  |  |  |  |
| OR (95% CI) | 1.115  (0.935-1.330) | 1.259  (0.863-1.837) | 1.000  (0.922-1.084) | 0.976  (0.821-1.159) | 0.803  (0.572-1.127) | 1.141  (0.318-4.101) | 0.905  (0.757-1.083) | 0.968  (0.654-1.432) | 1.009  (0.918-1.110) | 0.972  (0.821-1.150) | 1.019  (0.913-1.137) | 0.986  (0.779-1.247) | 1.099  (0.898-1.344) | 1.192  (0.749-1.897) |
| P value | 0.243 | 0.249 | 0.993 | 0.782 | 0.221 | 0.842 | 0.291 | 0.872 | 0.848 | 0.743 | 0.738 | 0.906 | 0.373 | 0.470 |
| Simple mode |  |  |  |  |  |  |  |  |  |  |  |  |  |  |
| OR (95% CI) | 1.131  (0.895-1.429) | 1.265  (0.833-1.922) | 1.030  (0.921-1.151) | 0.974  (0.826-1.148) | 0.824  (0.513-1.324) | 1.057  (0.310-3.598) | 0.839  (0.664-1.060) | 0.972  (0.658-1.436) | 1.011  (0.880-1.163) | 0.961  (0.798-1.157) | 0.989  (0.852-1.148) | 0.986  (0.786-1.236) | 1.113  (0.885-1.400) | 1.180  (0.704-1.979) |
| P value | 0.318 | 0.286 | 0.613 | 0.756 | 0.435 | 0.931 | 0.159 | 0.888 | 0.874 | 0.679 | 0.888 | 0.902 | 0.374 | 0.539 |
| MR-PRESSO |  |  |  |  |  |  |  |  |  |  |  |  |  |  |
| OR (95% CI) | 1.028  (0.920-1.149) | 1.084  (0.917-1.281) | 1.016  (0.970-1.064) | 1.022  (0.953-1.097) | 0.927  (0.724-1.188) | 0.920  (0.560-1.511) | 0.932  (0.839-1.036) | 1.020  (0.866-1.202) | 0.951  (0.889-1.014) | 0.992  (0.906-1.078) | 0.973  (0.906-1.044) | 1.024  (0.923-1.137) | 1.069  (0.970-1.178) | 1.052  (0.880-1.257) |
| P value | 0.627 | 0.345 | 0.509 | 0.542 | 0.550 | 0.741 | 0.193 | 0.809 | 0.136 | 0.861 | 0.445 | 0.650 | 0.179 | 0.580 |
| **Sensitivity analysis** |  |  |  |  |  |  |  |  |  |  |  |  |  |  |
| Cochran’s Q |  |  |  |  |  |  |  |  |  |  |  |  |  |  |
| Q-statistics | 22.880 | 16.470 | 19.490 | 12.209 | 25.396 | 15.631 | 21.711 | 14.307 | 32.662 | 43.609 | 22.523 | 29.269 | 14.015 | 15.960 |
| Q_df | 17 | 16 | 17 | 16 | 17 | 16 | 17 | 16 | 17 | 16 | 17 | 16 | 17 | 16 |
| P value | 0.153 | 0.421 | 0.301 | 0.729 | 0.086 | 0.479 | 0.196 | 0.576 | 0.012 | <1E-4 | 0.165 | 0.022 | 0.666 | 0.456 |
| MR-Egger |  |  |  |  |  |  |  |  |  |  |  |  |  |  |
| Q-statistics | 22.750 | 16.186 | 19.307 | 11.966 | 20.077 | 15.615 | 20.999 | 12.586 | 32.660 | 34.572 | 20.430 | 29.099 | 14.015 | 15.291 |
| Q_df | 16 | 15 | 16 | 15 | 16 | 15 | 16 | 15 | 16 | 15 | 16 | 15 | 16 | 15 |
| P value | 0.121 | 0.370 | 0.253 | 0.682 | 0.217 | 0.408 | 0.179 | 0.634 | 0.008 | 0.003 | 0.201 | 0.016 | 0.598 | 0.431 |
| Egger intercept |  |  |  |  |  |  |  |  |  |  |  |  |  |  |
| Intercept | 4.06E-3 | 1.02E-2 | -2.19E-3 | -4.05E-3 | 5.50E-2 | 7.29E-3 | -9.24E-3 | 2.50E-2 | 2.10E-4 | 2.35E-2 | -1.04E-2 | 3.69E-3 | 7.71E-5 | 1.69E-2 |
| P value | 0.766 | 0.616 | 0.702 | 0.629 | 0.056 | 0.903 | 0.472 | 0.209 | 0.980 | 0.066 | 0.219 | 0.771 | 0.995 | 0.431 |
| MR-PRESSO |  |  |  |  |  |  |  |  |  |  |  |  |  |  |
| P value | 0.176 | 0.434 | 0.304 | 0.723 | 0.101 | 0.485 | 0.211 | 0.580 | 0.014 | <1E-4 | 0.183 | 0.025 | 0.623 | 0.450 |

Supplementary Table 9. Two-sample Mendelian randomization estimations showing the effects, heterogeneity and horizontal pleiotropy of multiple birth on the risk of endocrine system disease.
