# Supplementary material for: Long-term outcomes of offspring from multiple gestations: a two-sample Mendelian randomization study on multi-system diseases using UK Biobank and FinnGen databases
Source: J Transl Med. 2023 Sep 8;21:608. doi: 10.1186/s12967-023-04423-w (PMC10492369; doi:10.1186/s12967-023-04423-w)

**Attention deficit hyperactivity disorder (ADHD) – FinnGen**


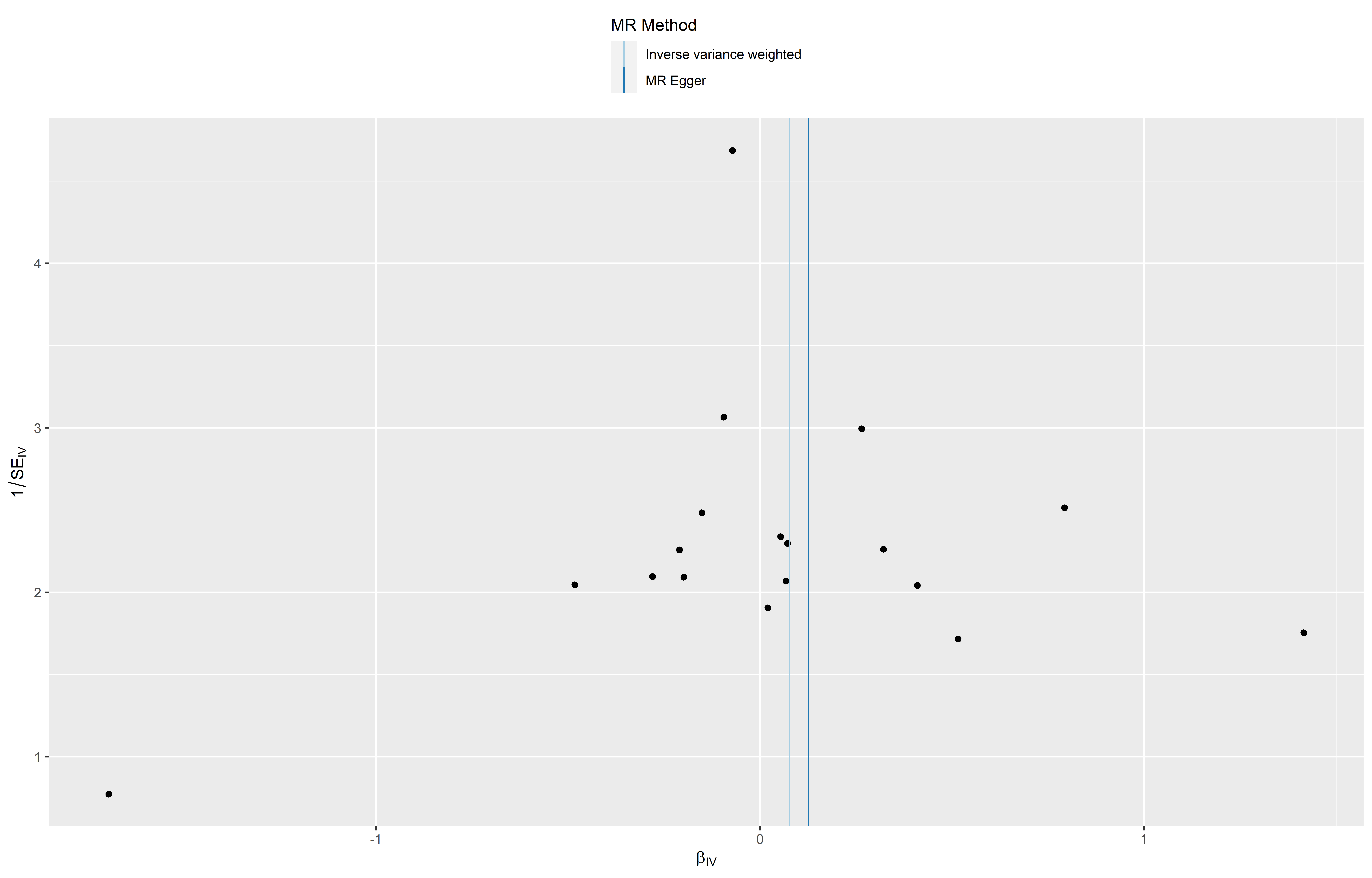

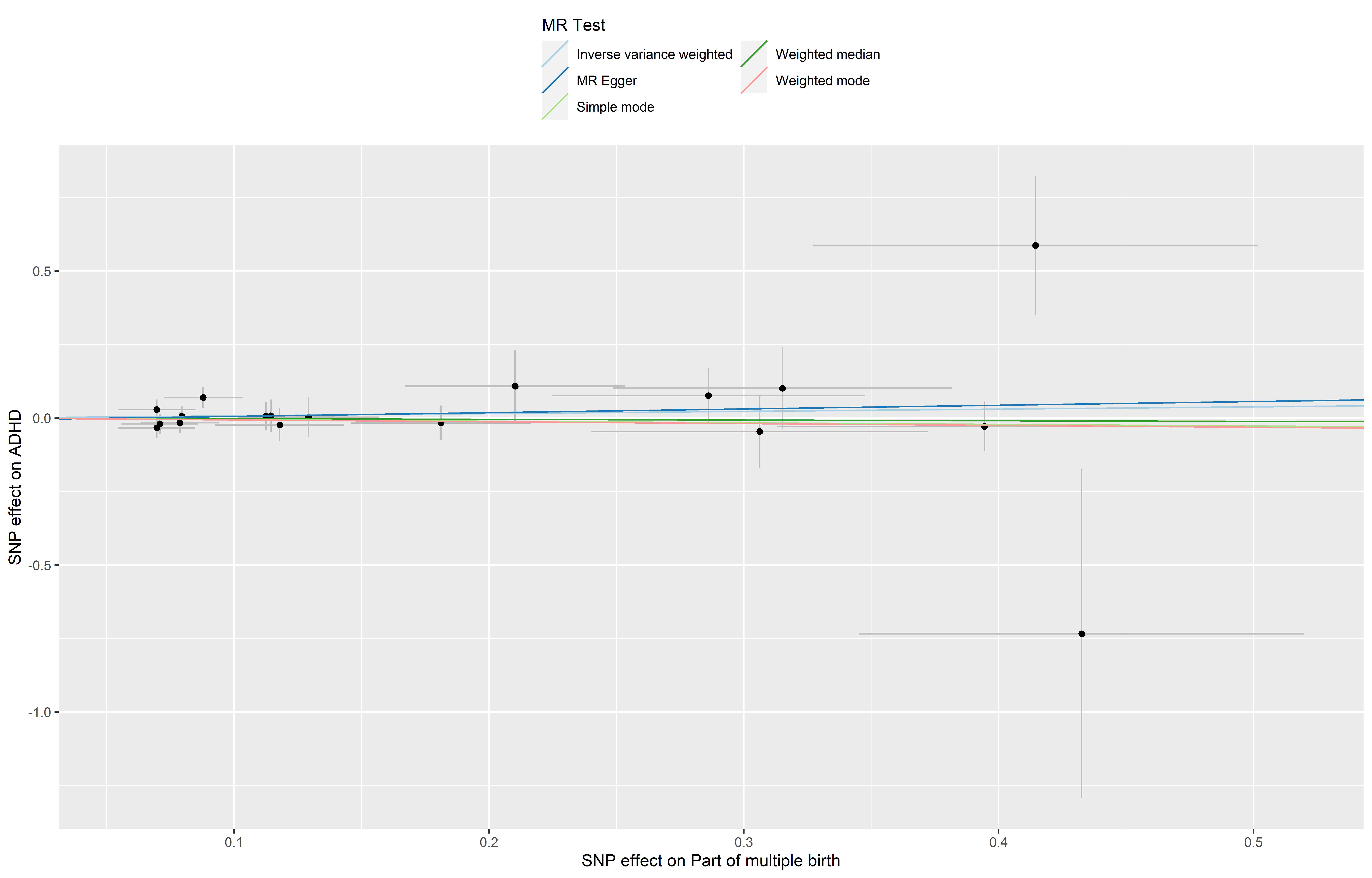


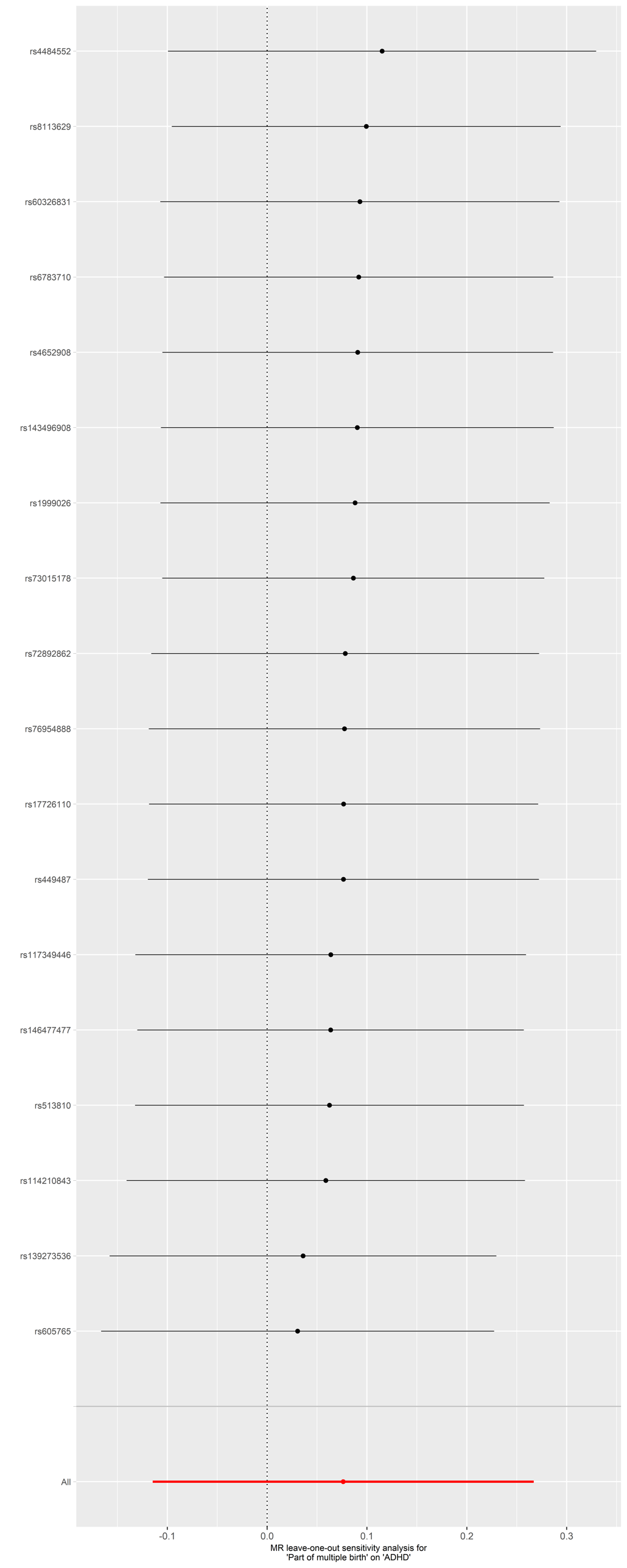


**Attention deficit hyperactivity disorder (ADHD) – UK Biobank**


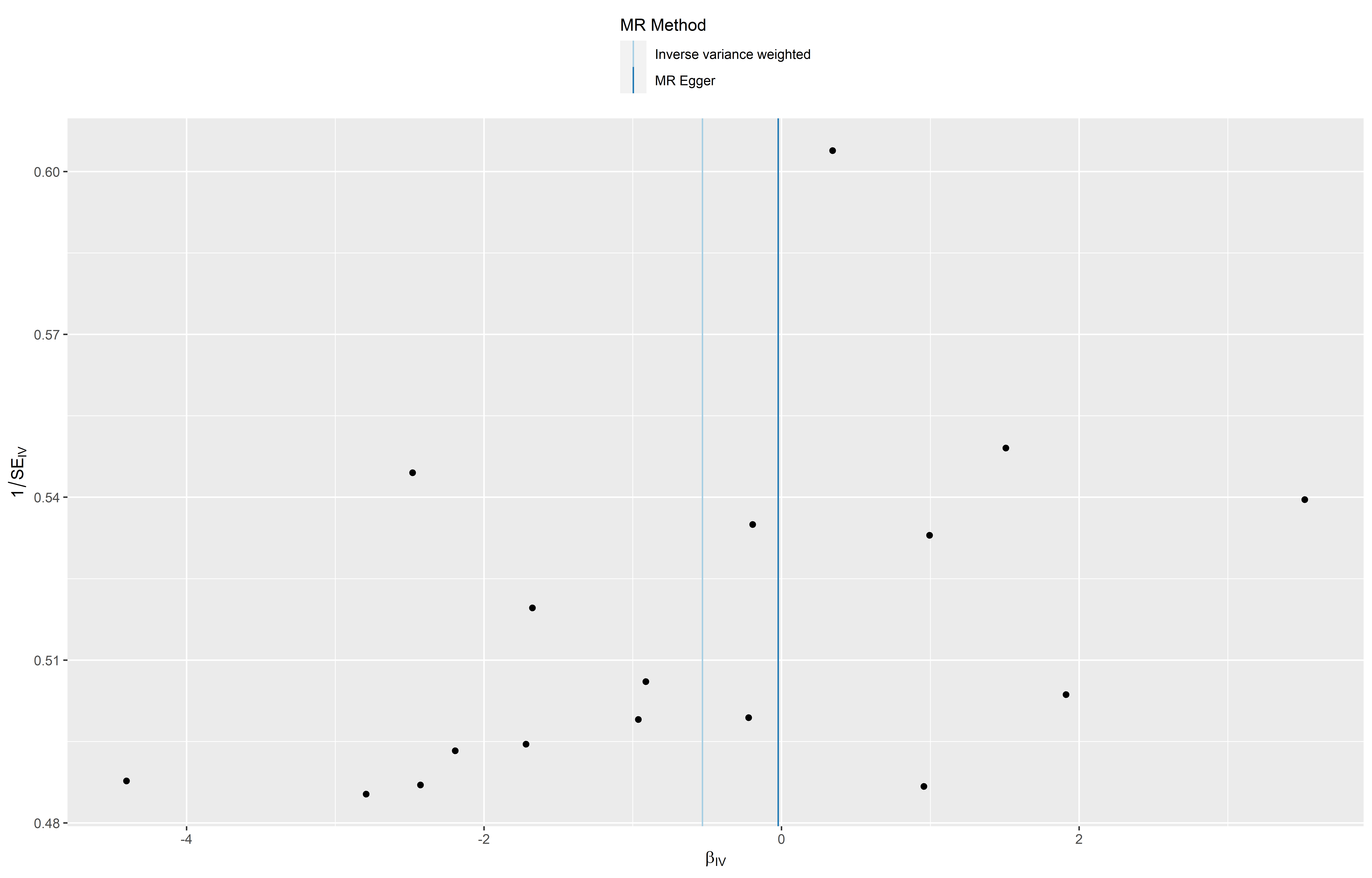

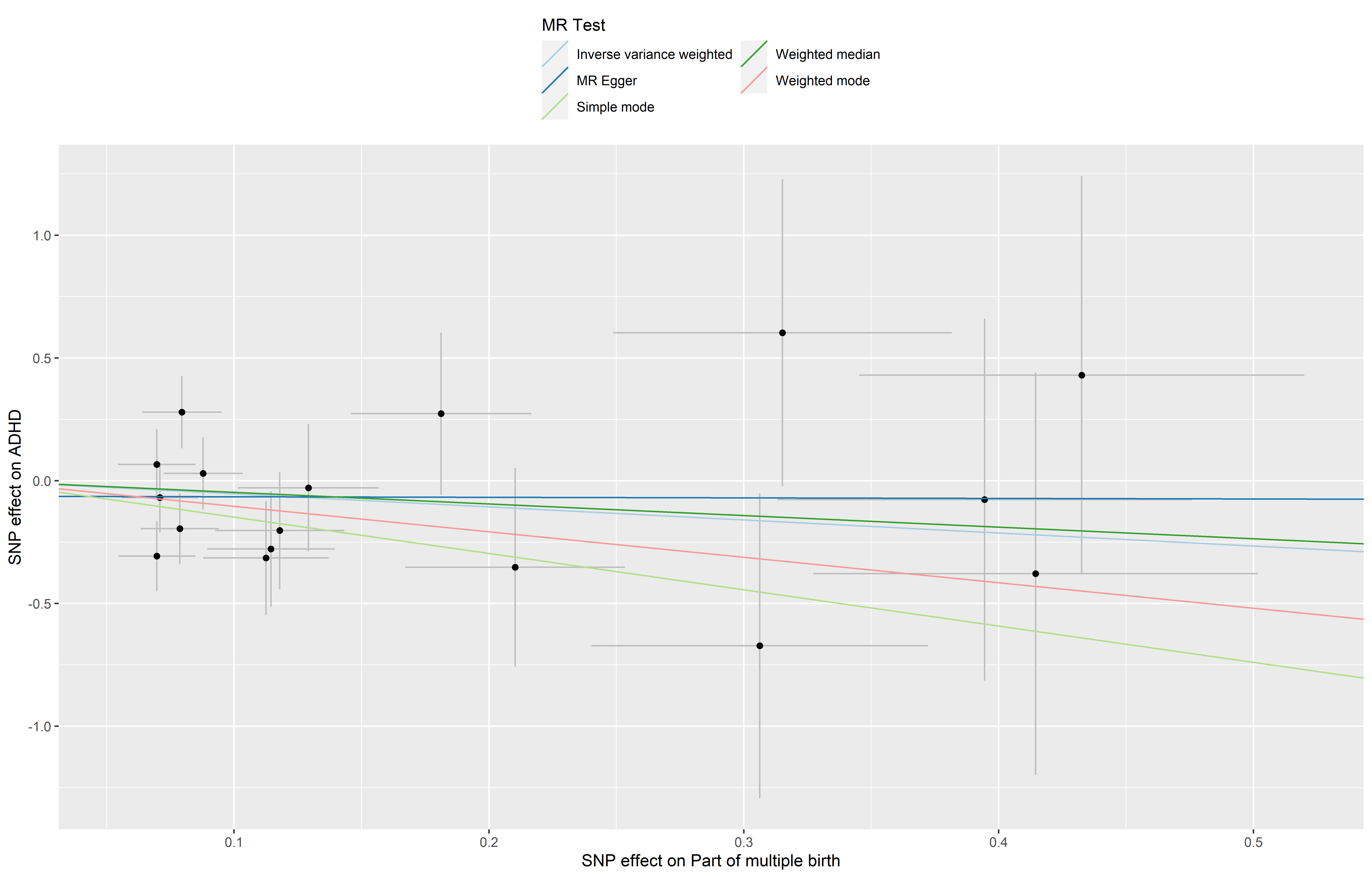


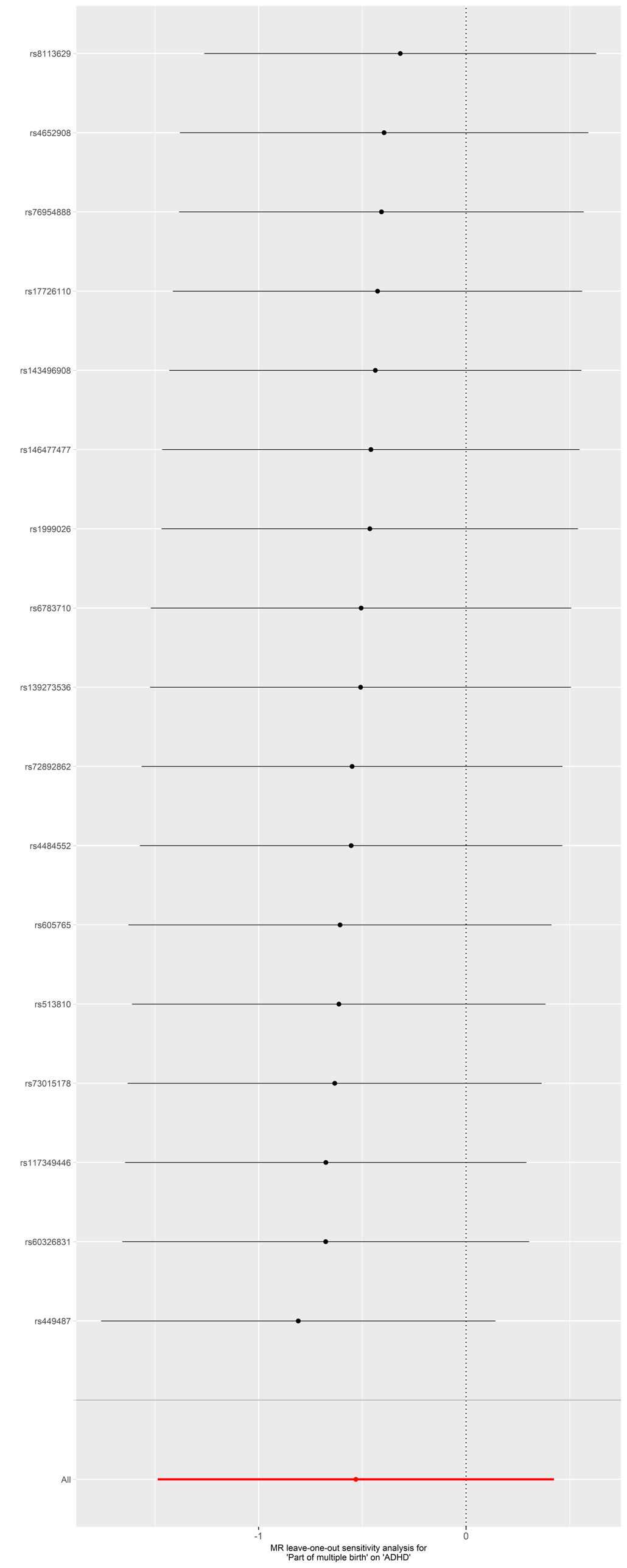


**Depression – FinnGen**


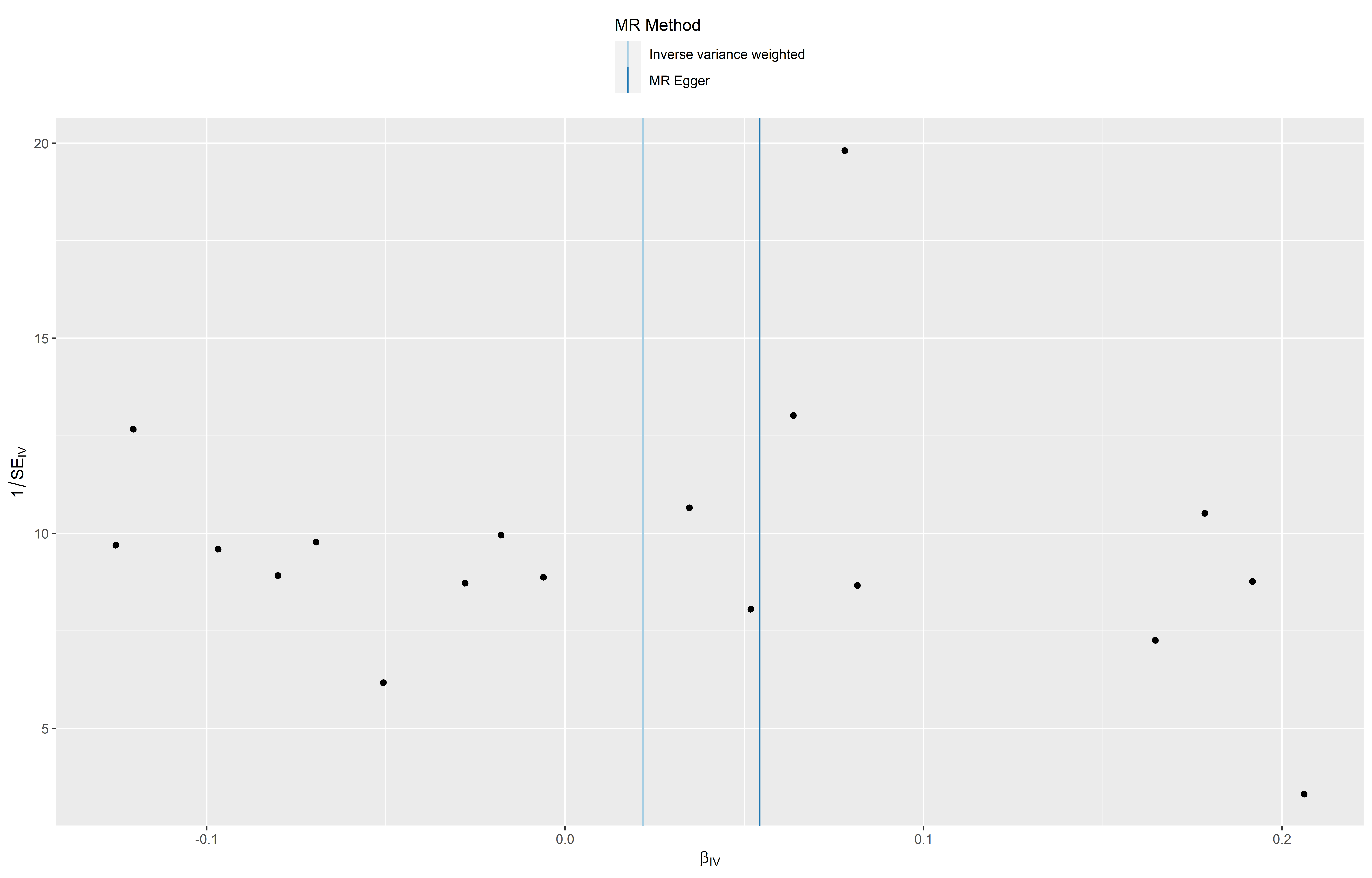

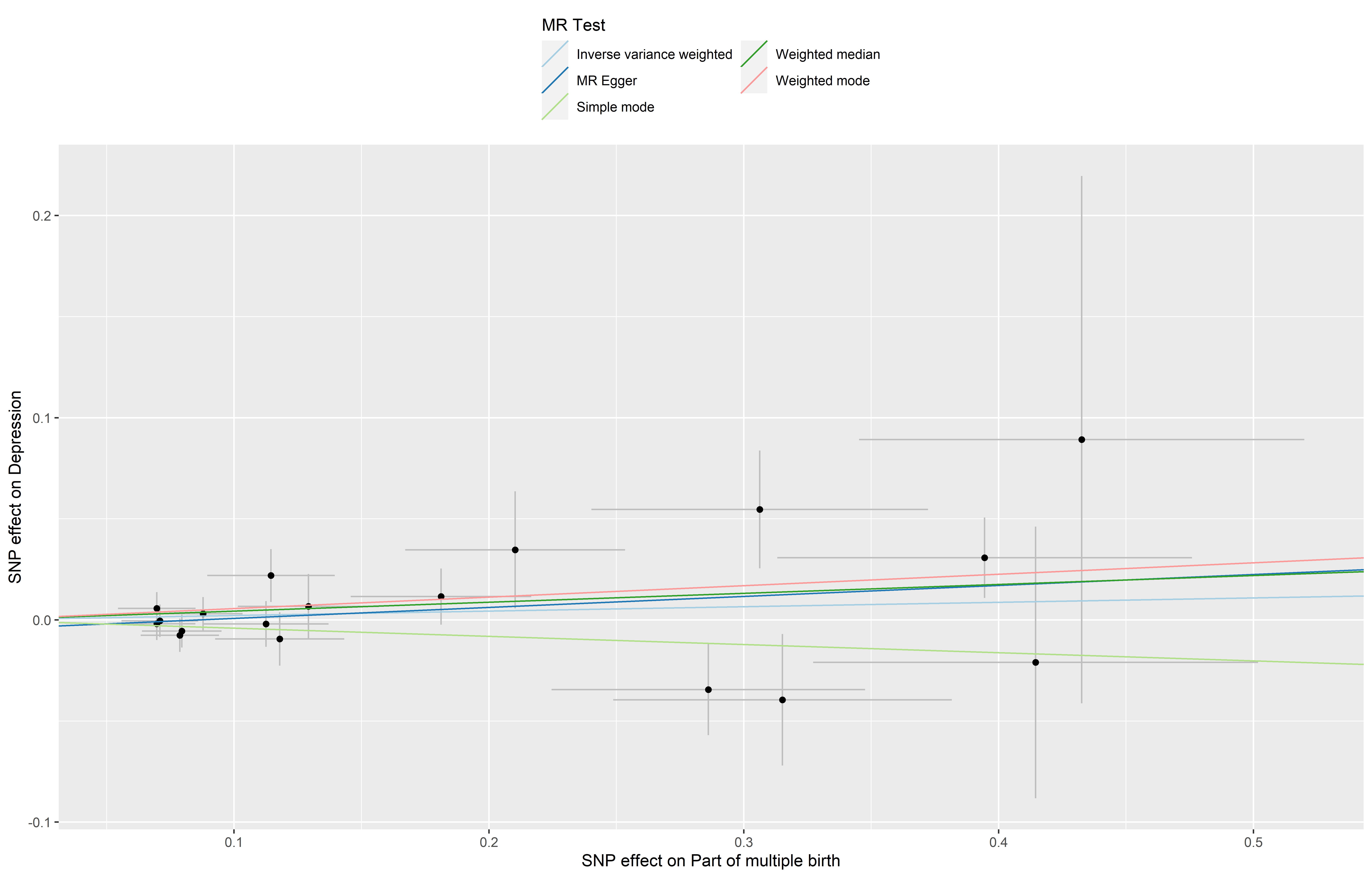


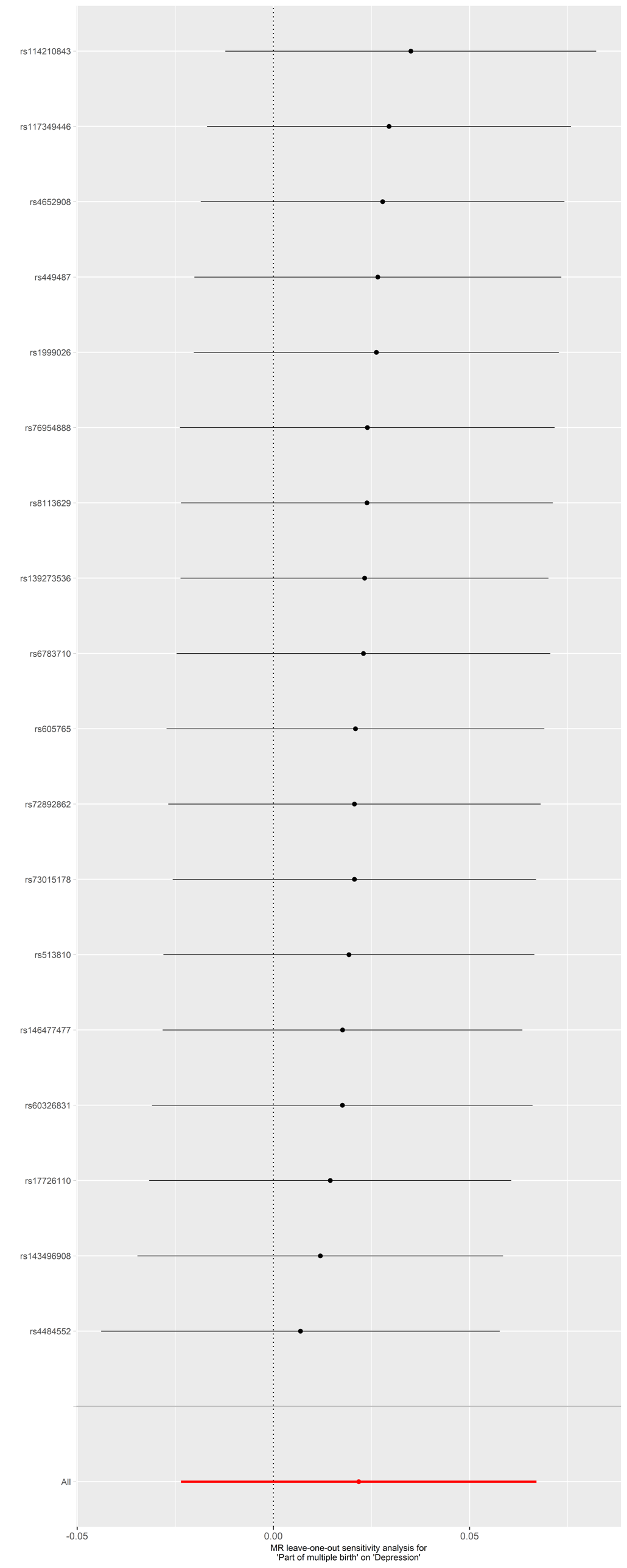


**Depression – UK Biobank**


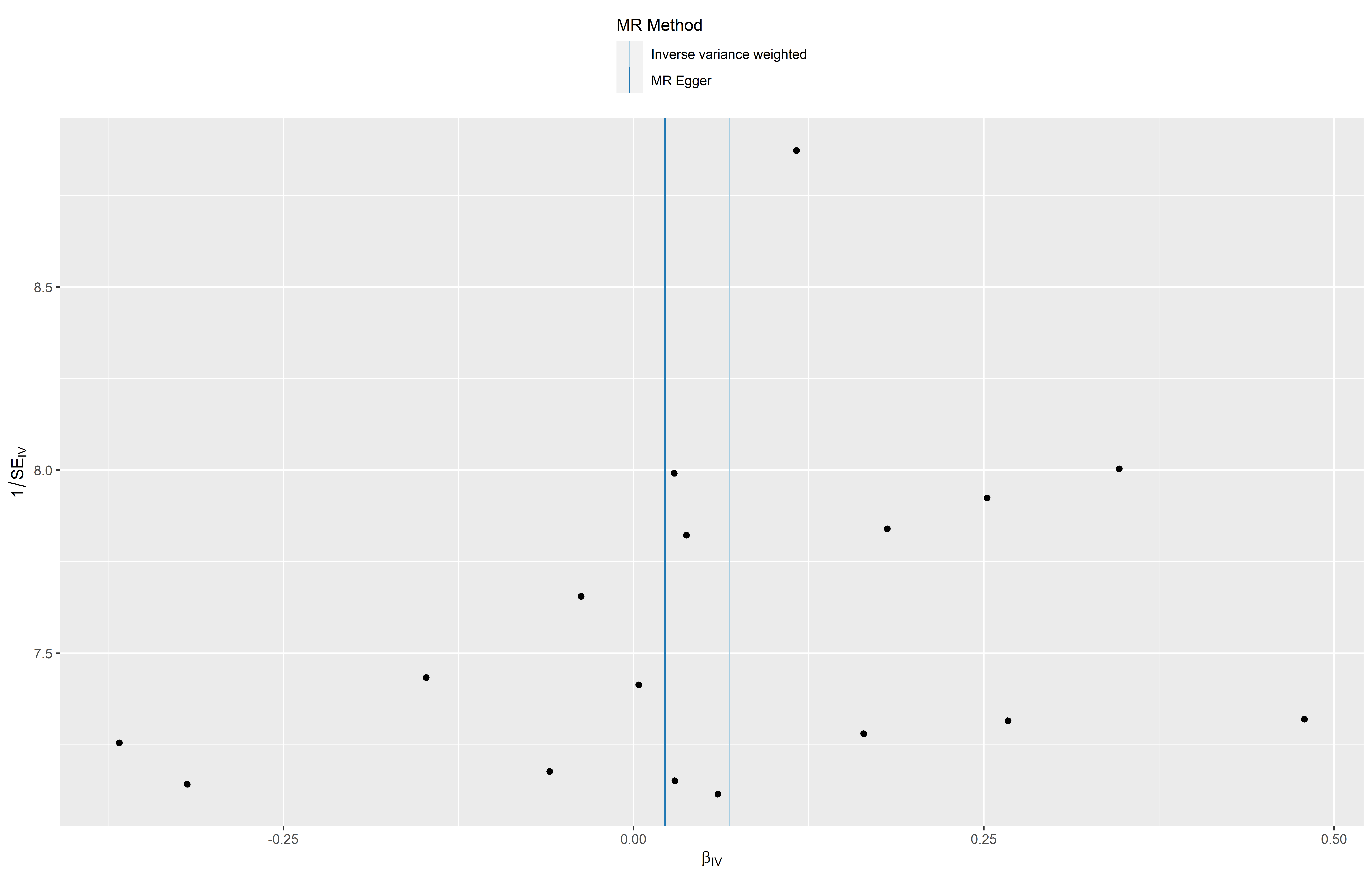

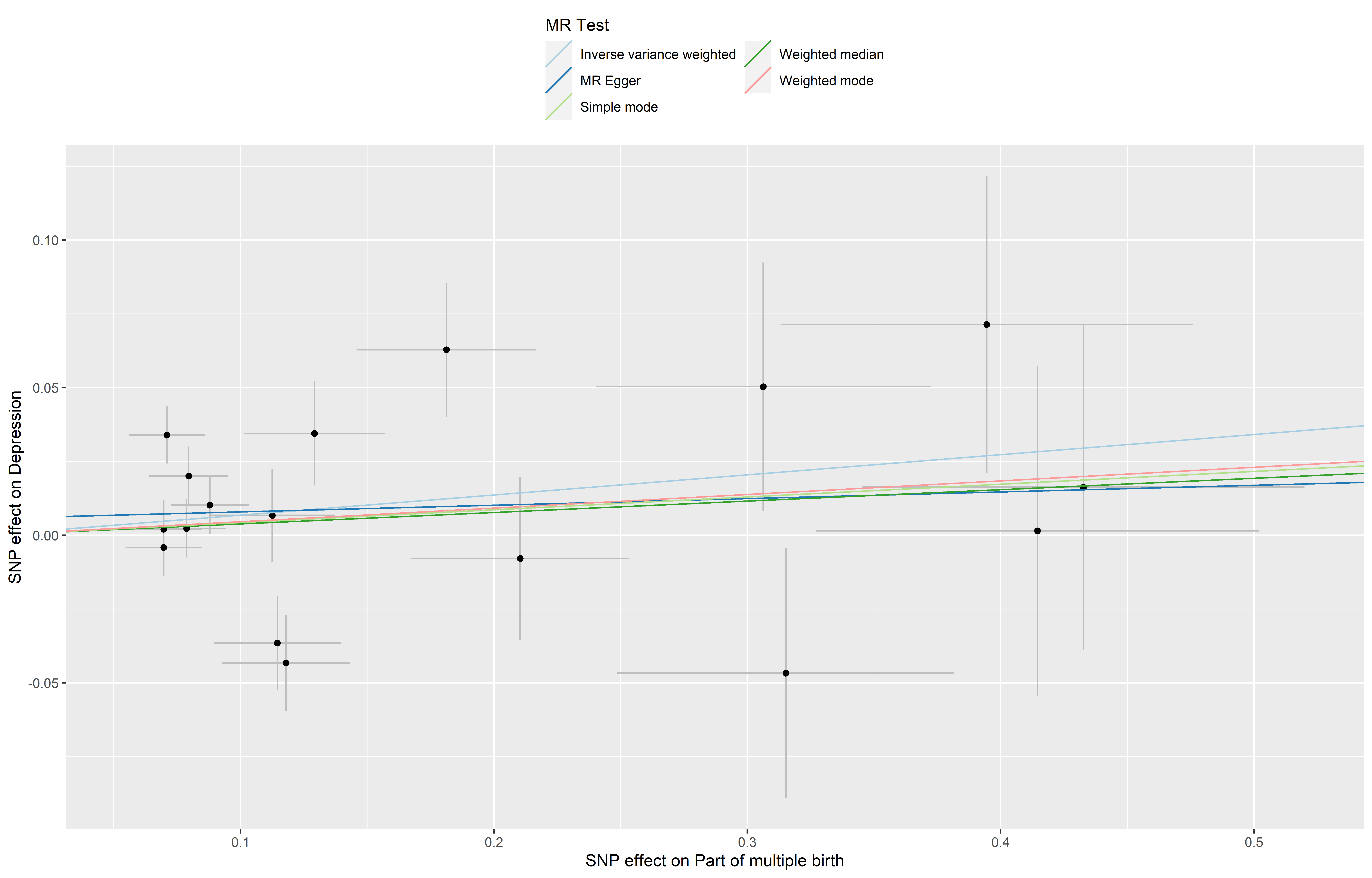


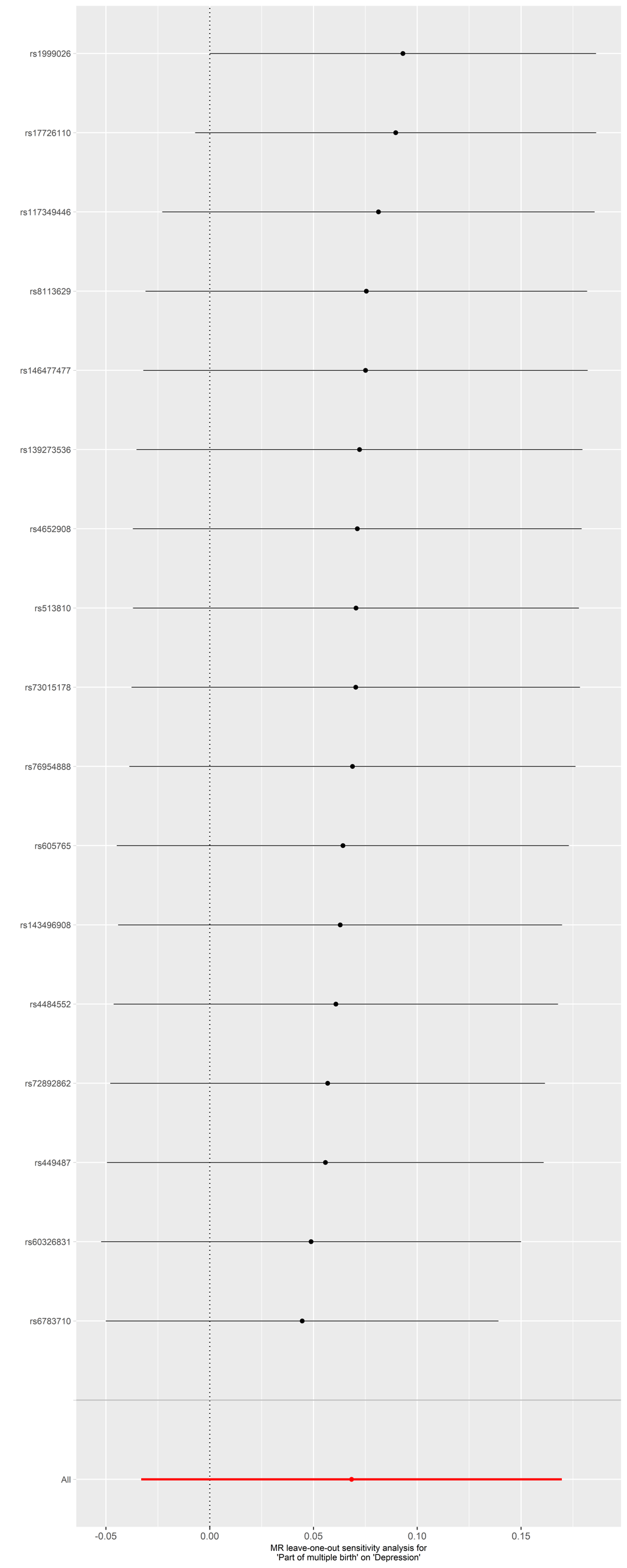


**Autism – FinnGen**


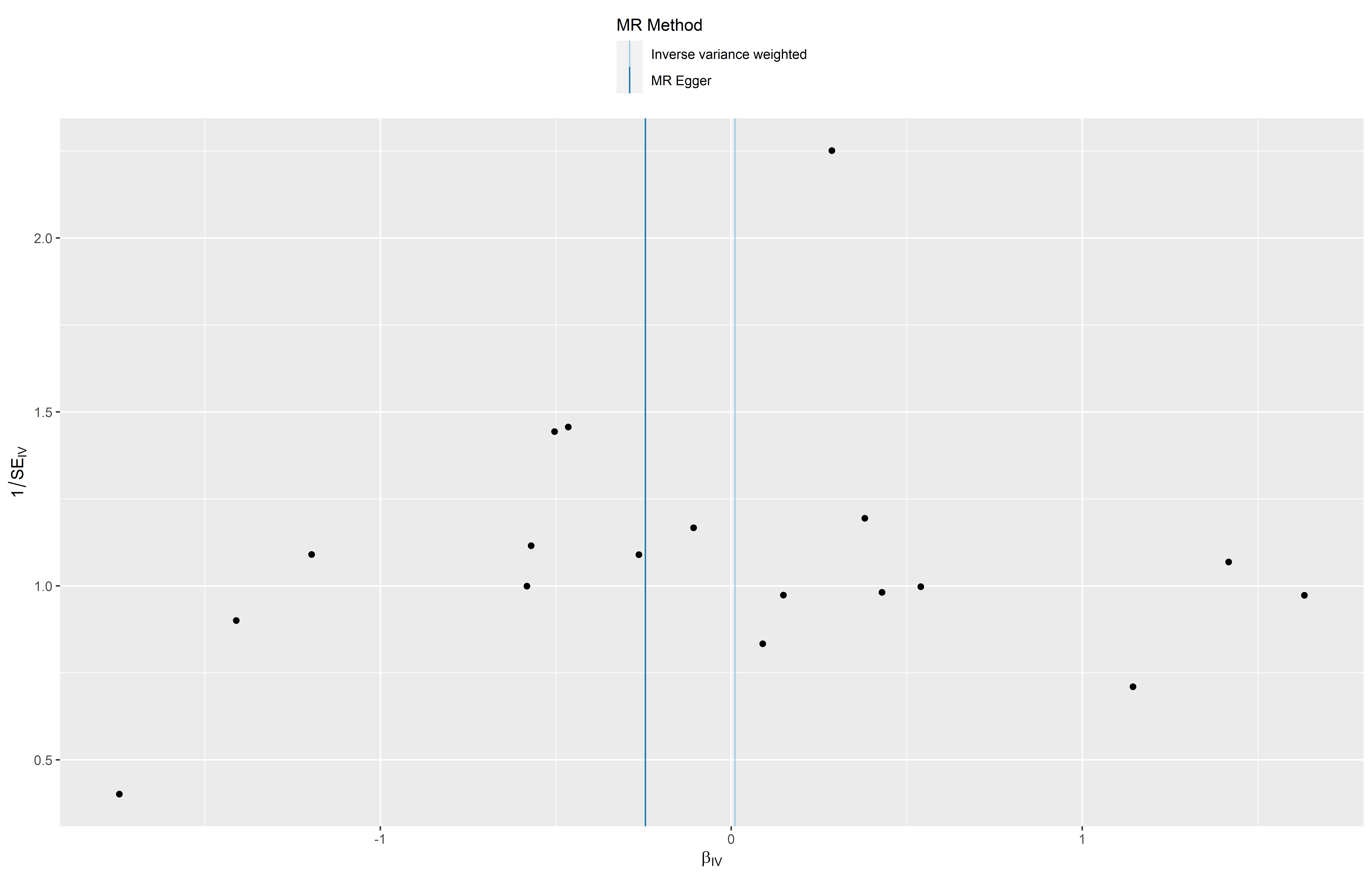

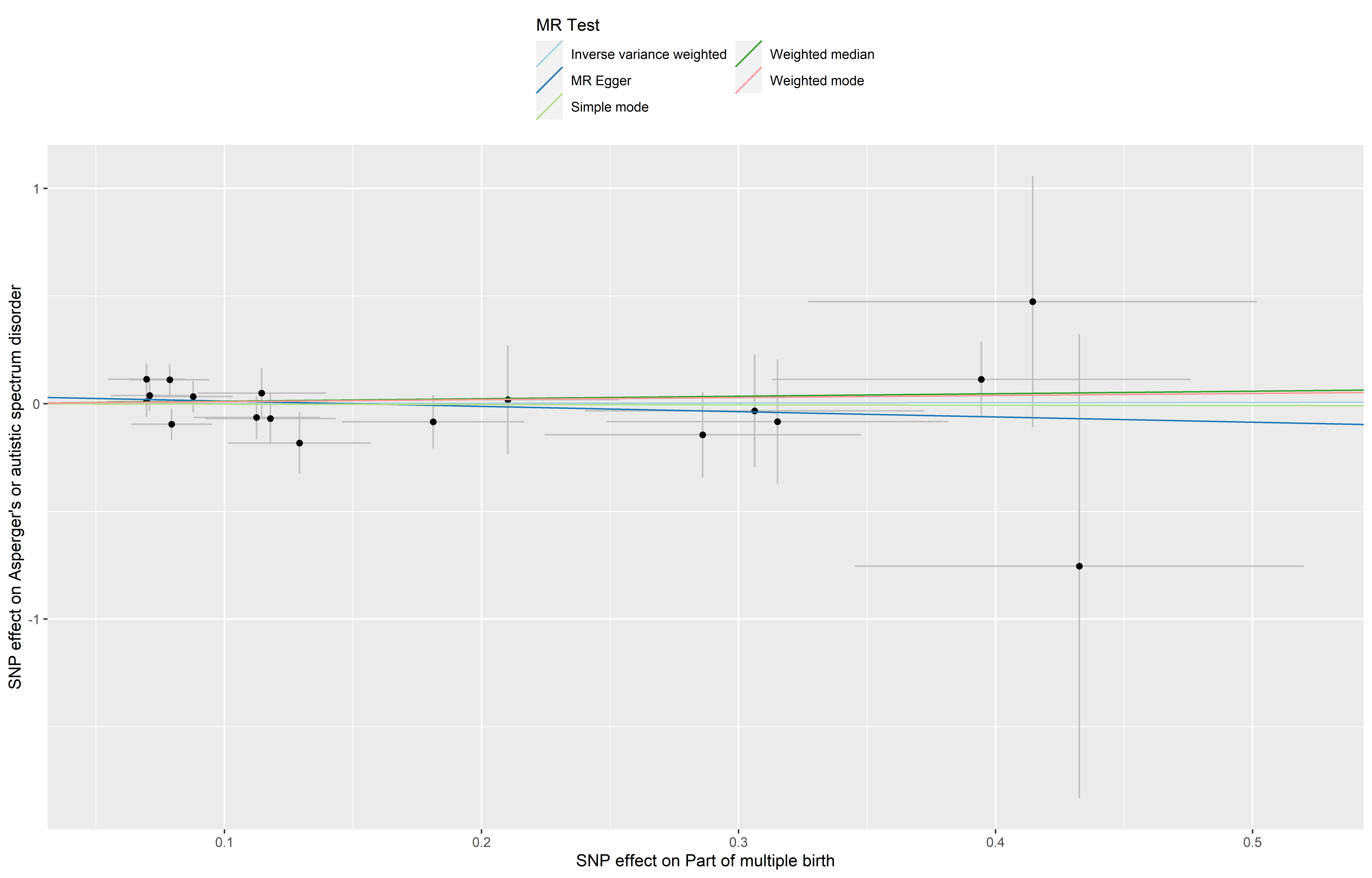


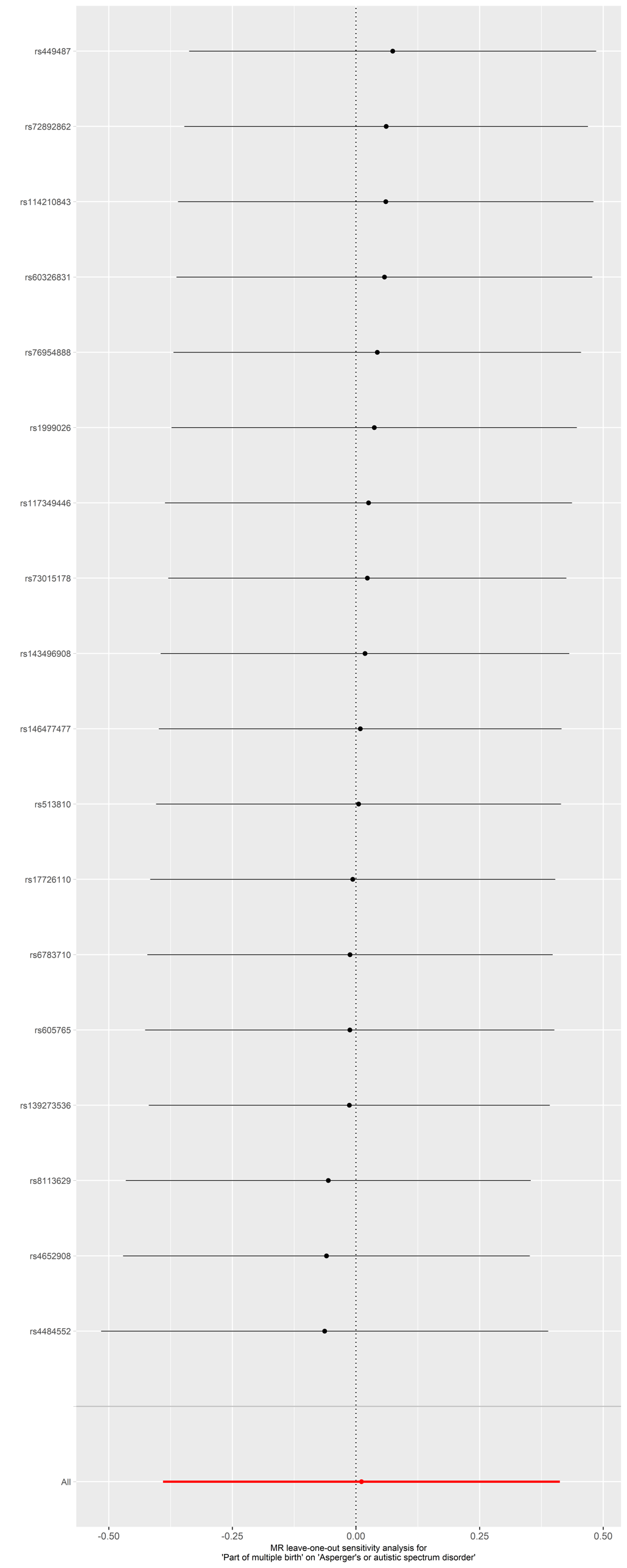


**Autism – UK Biobank**


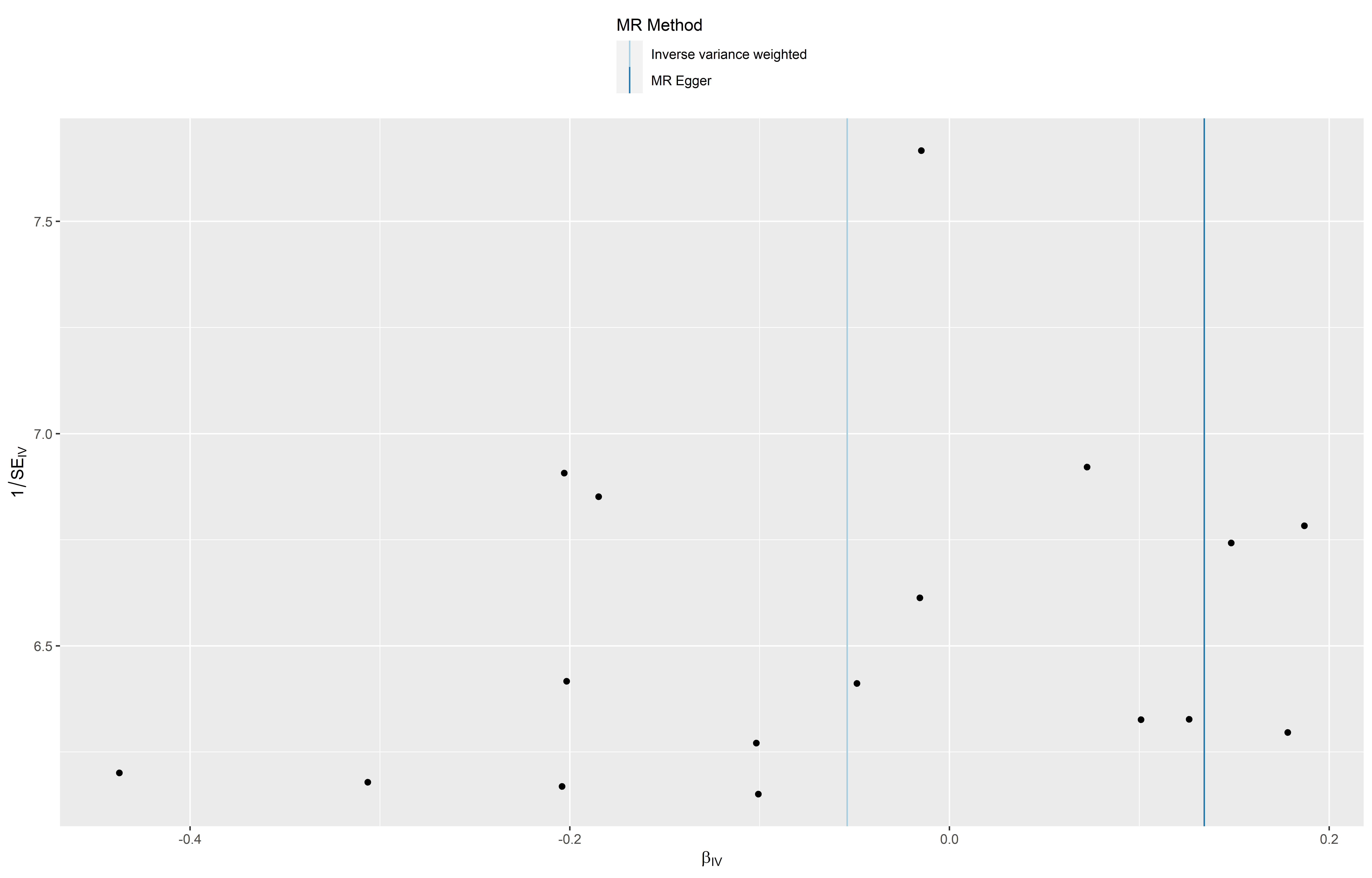

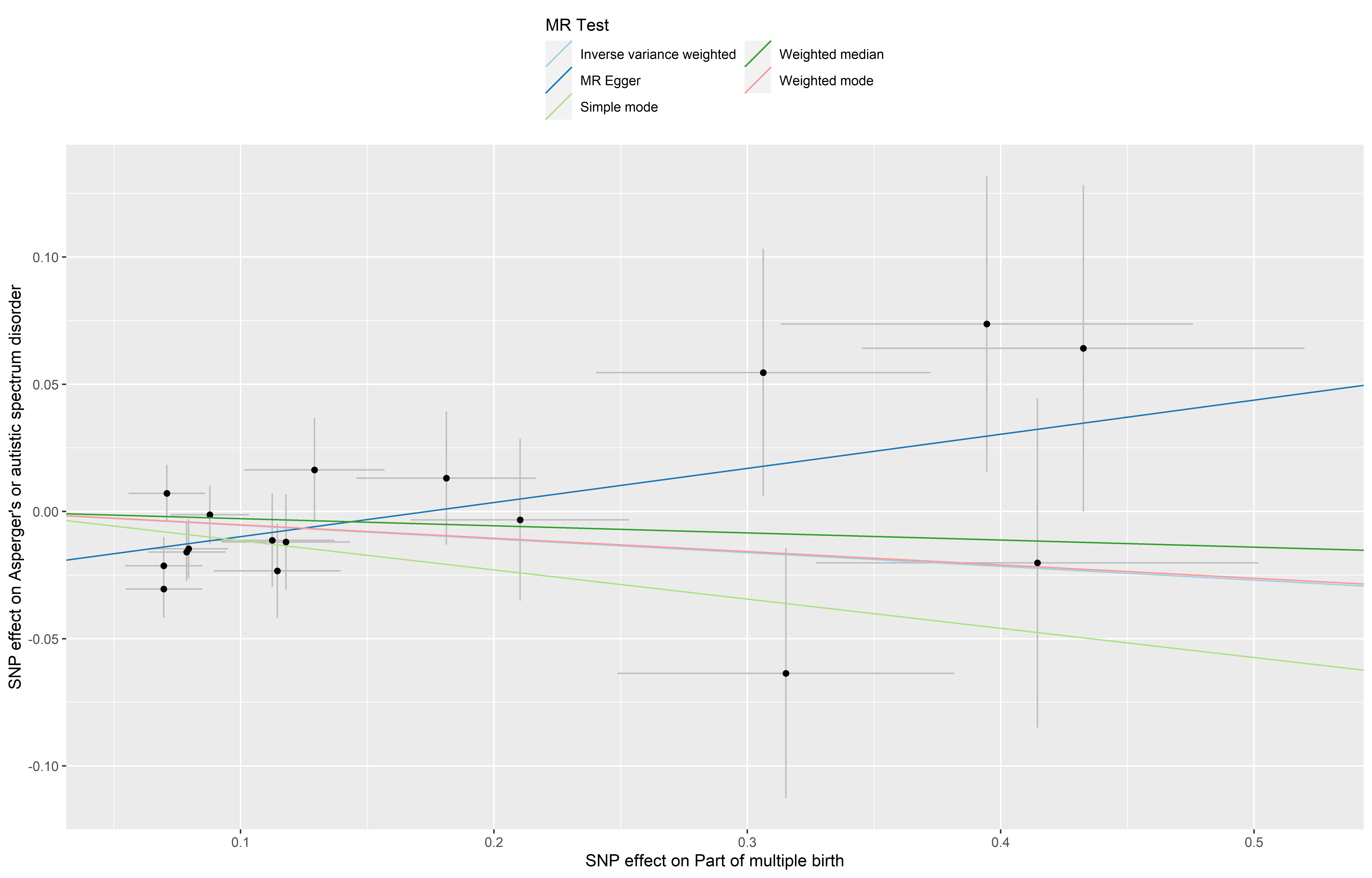


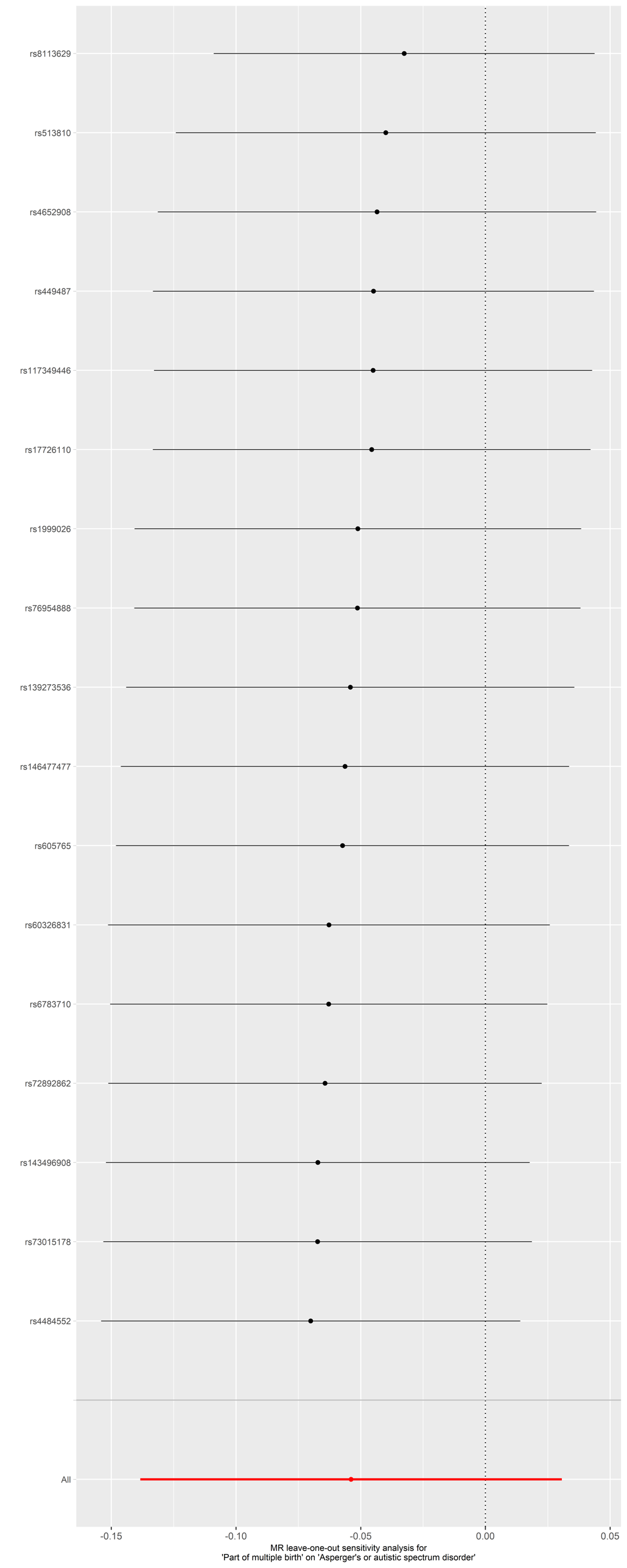


**Bipolar affective disorder – FinnGen**


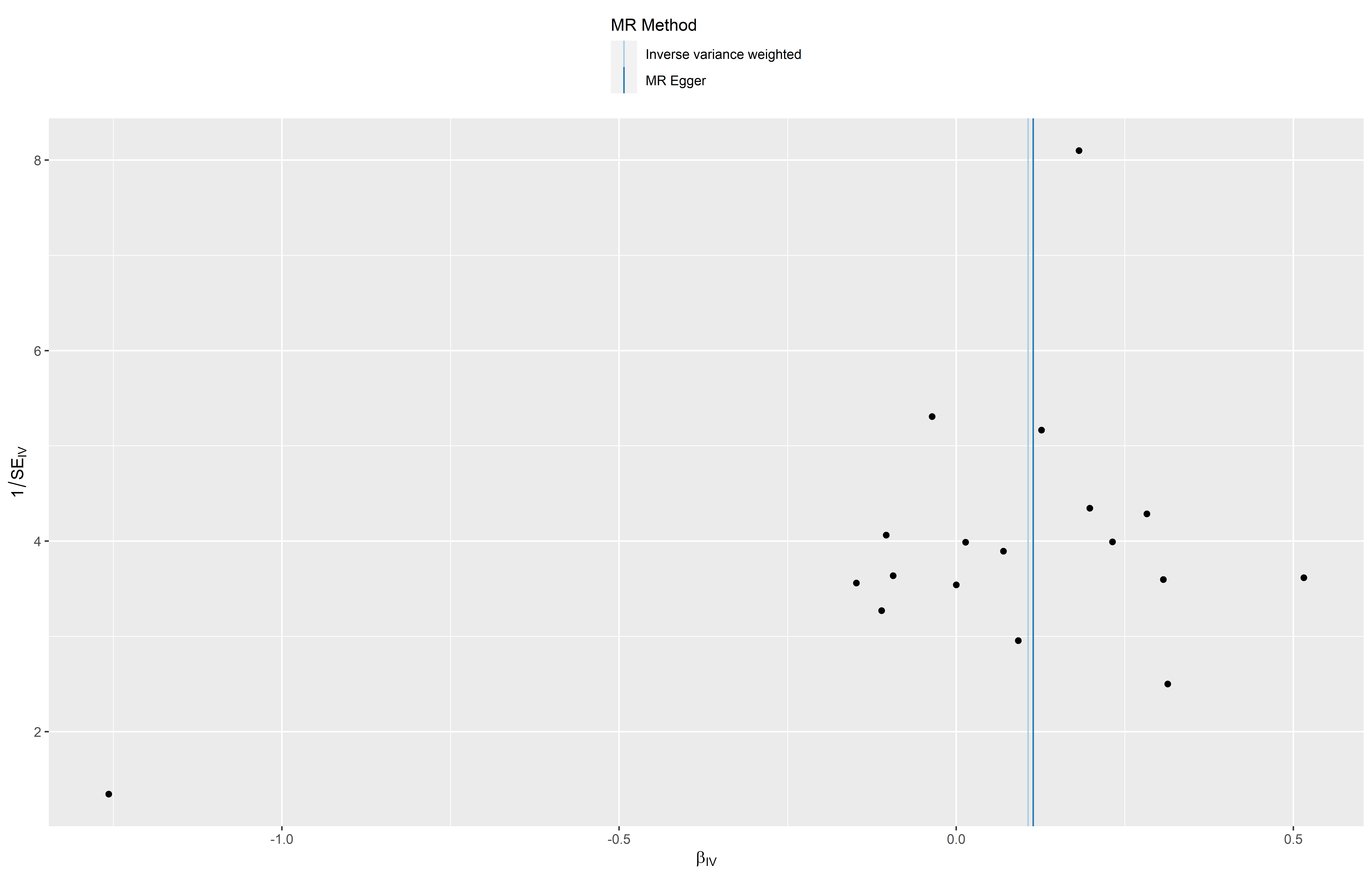

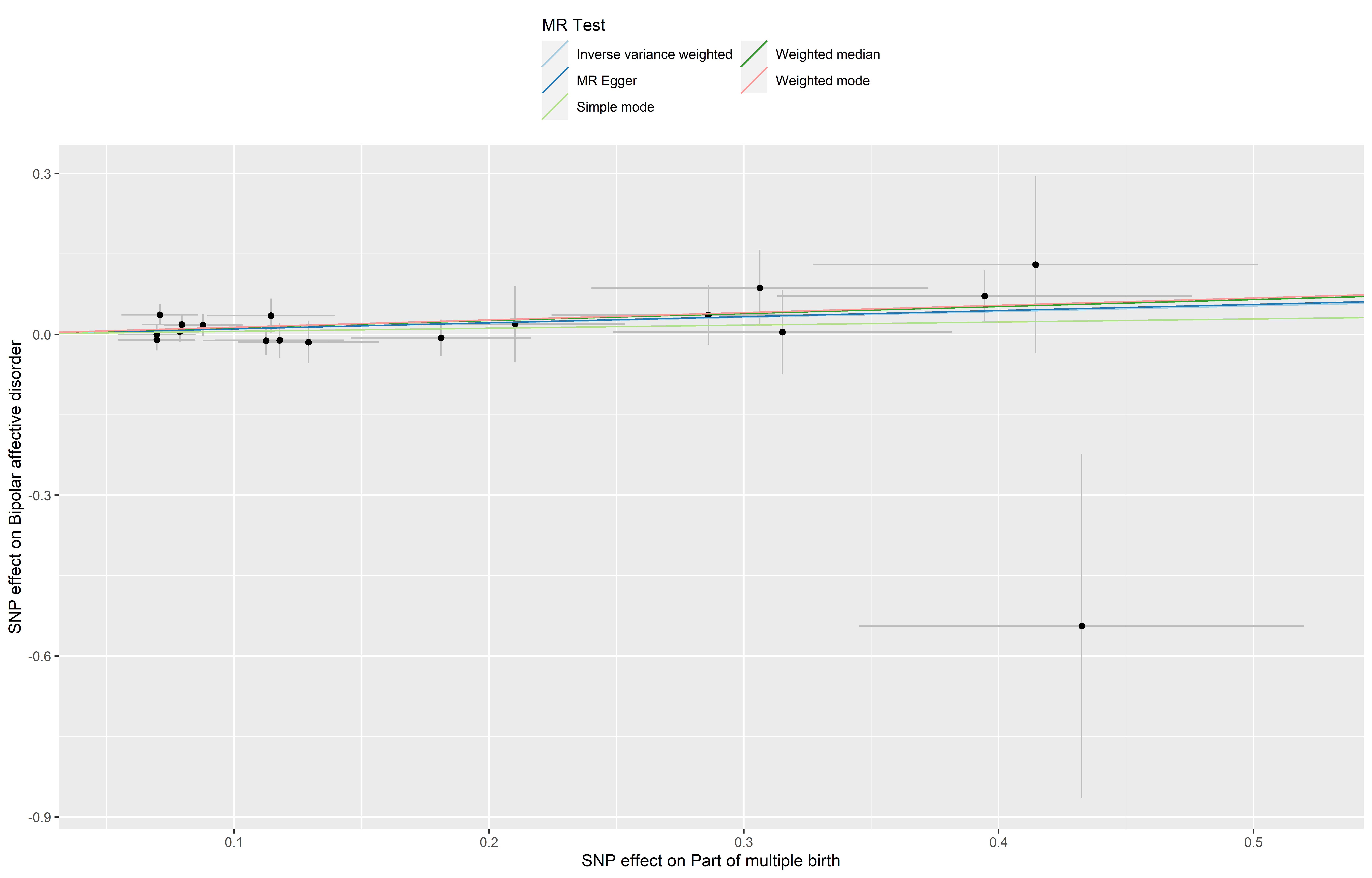


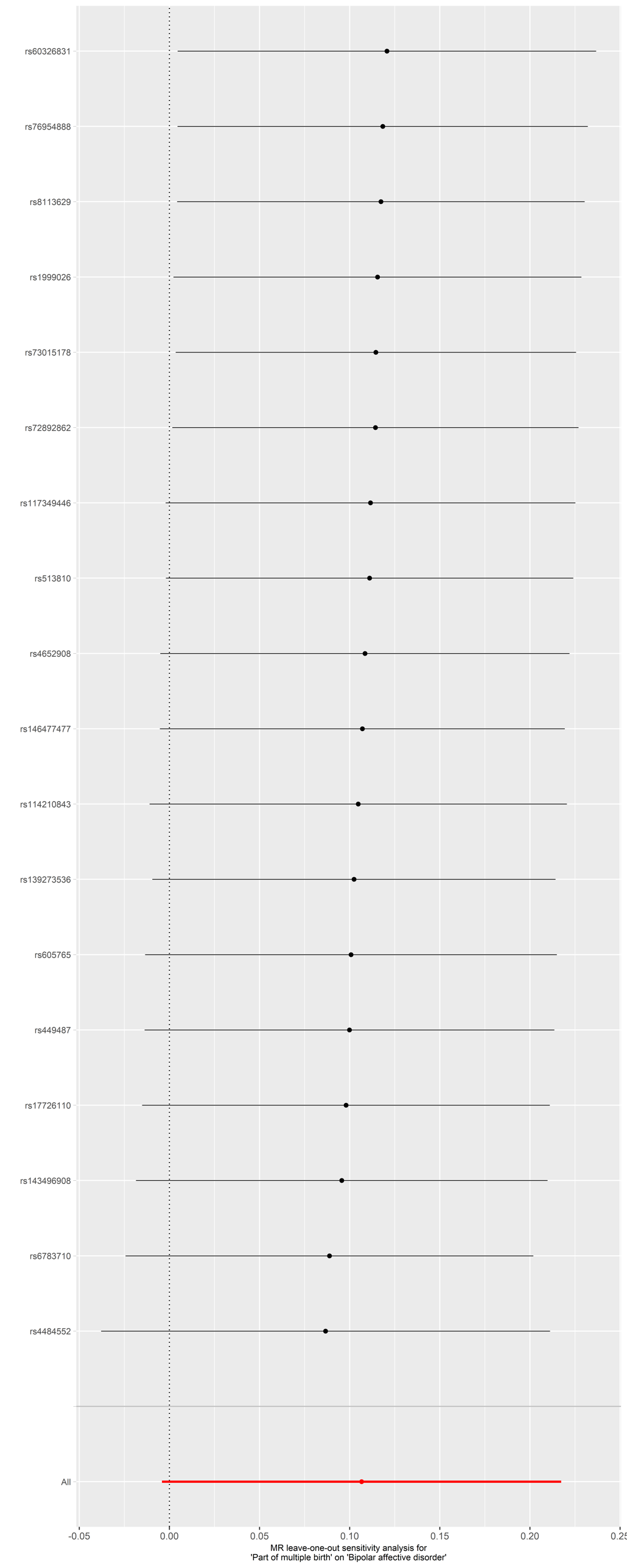


**Bipolar affective disorder – UK Biobank**


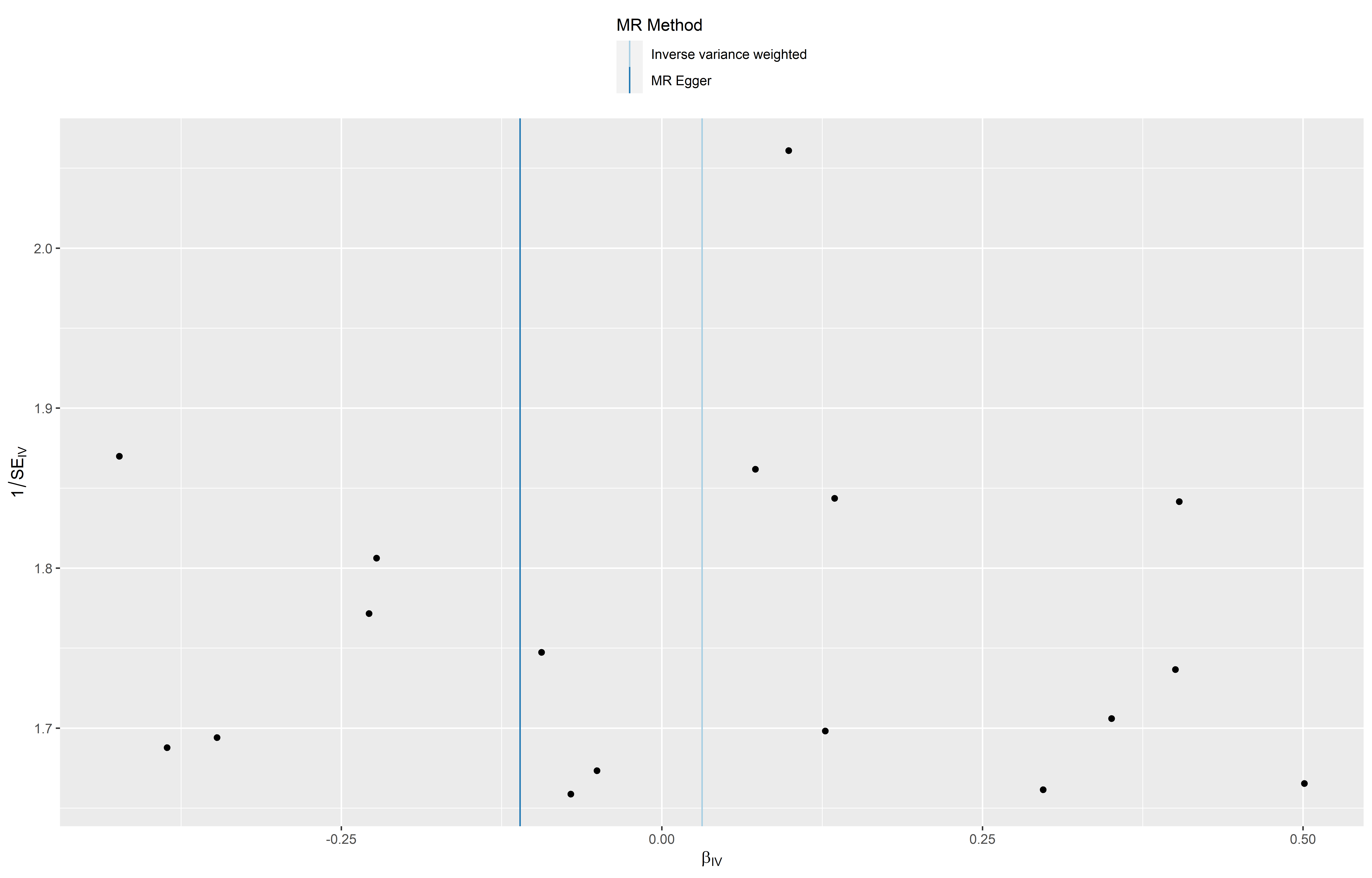

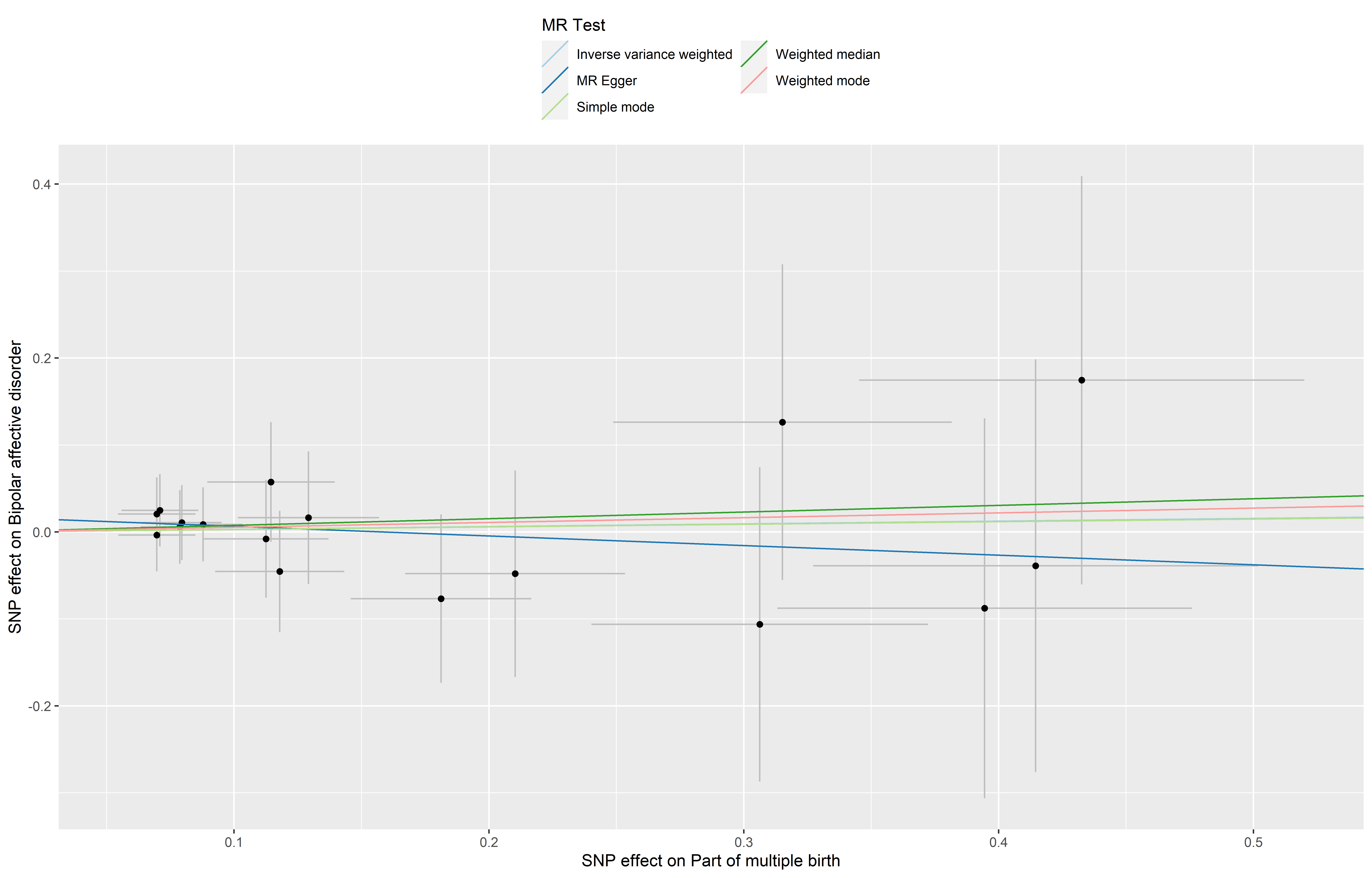


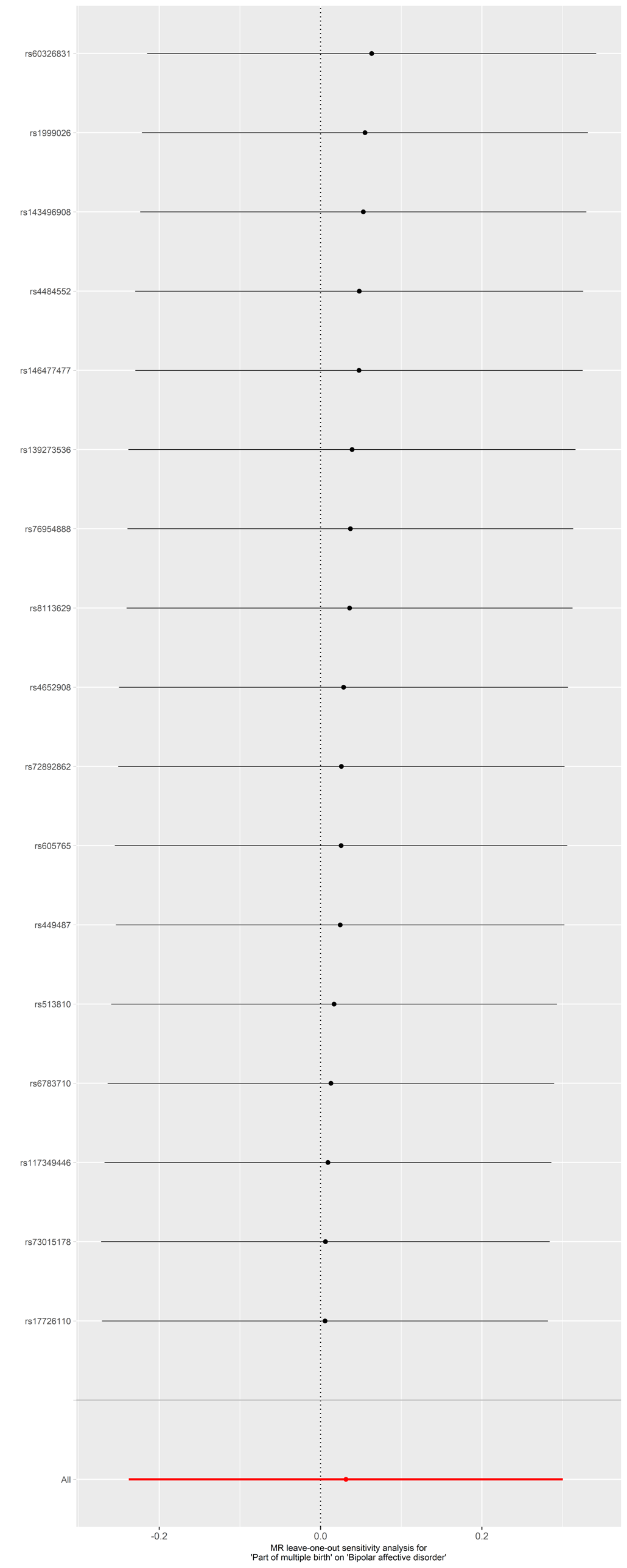


**Cognitive impairment – UK Biobank**


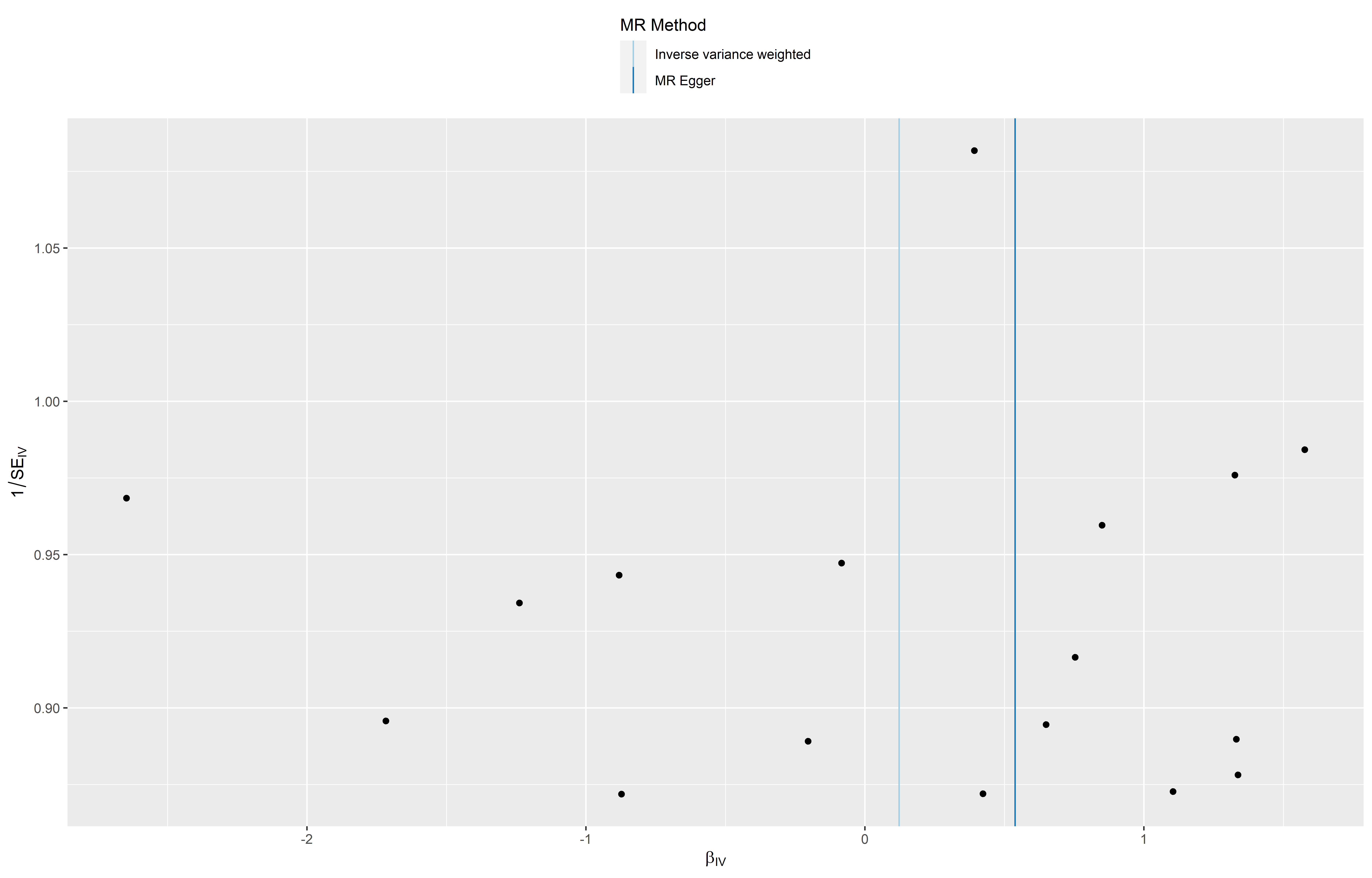

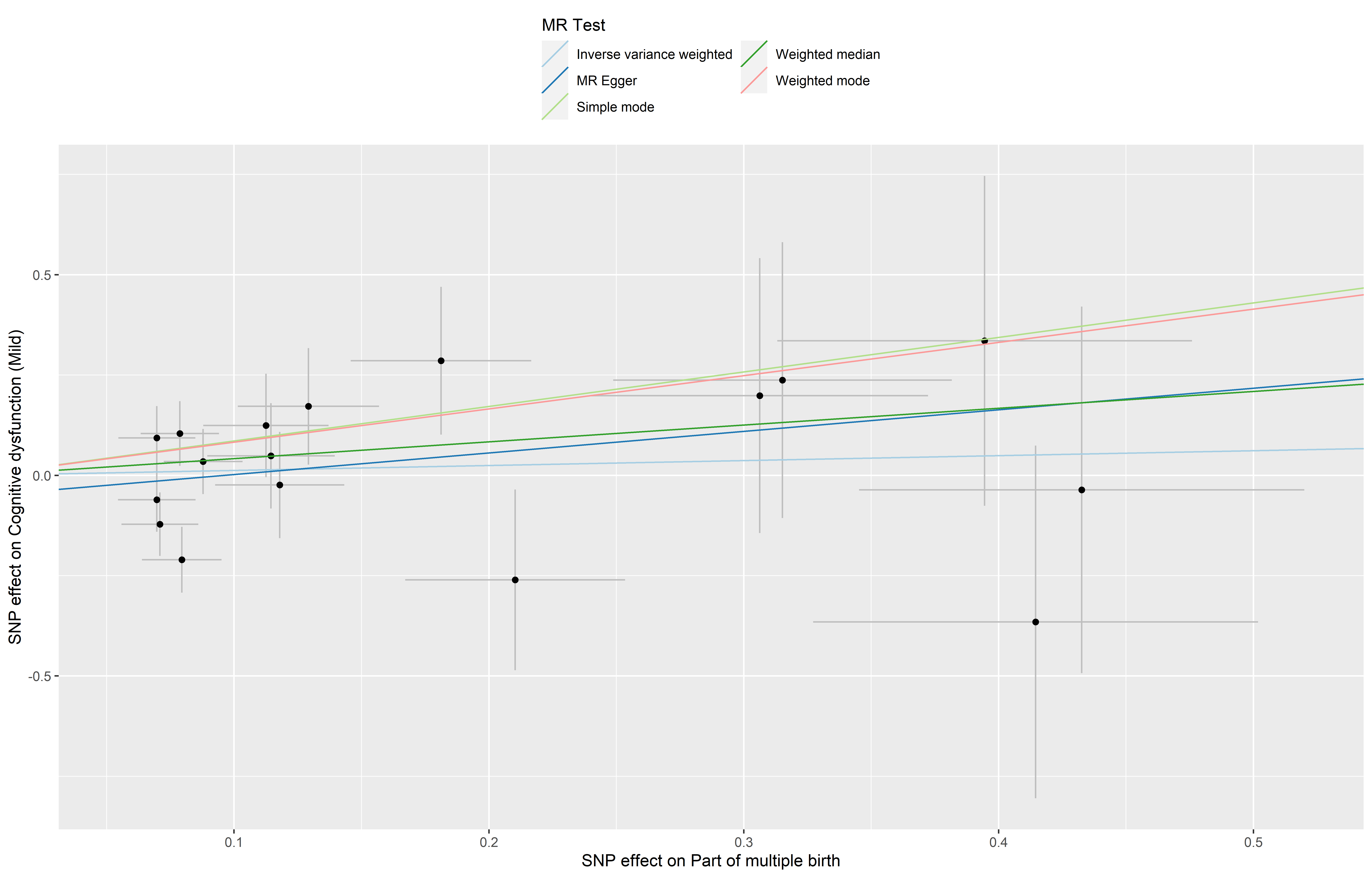


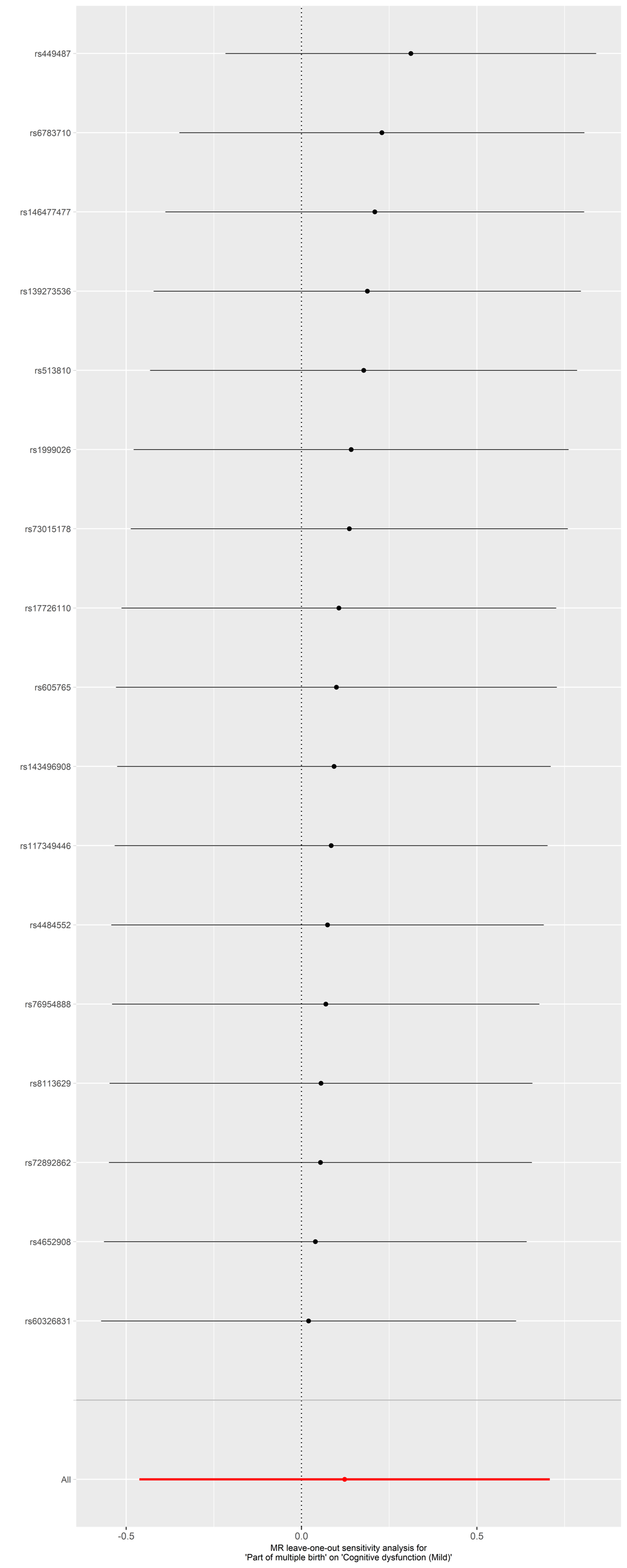


**Schizophrenia – FinnGen**


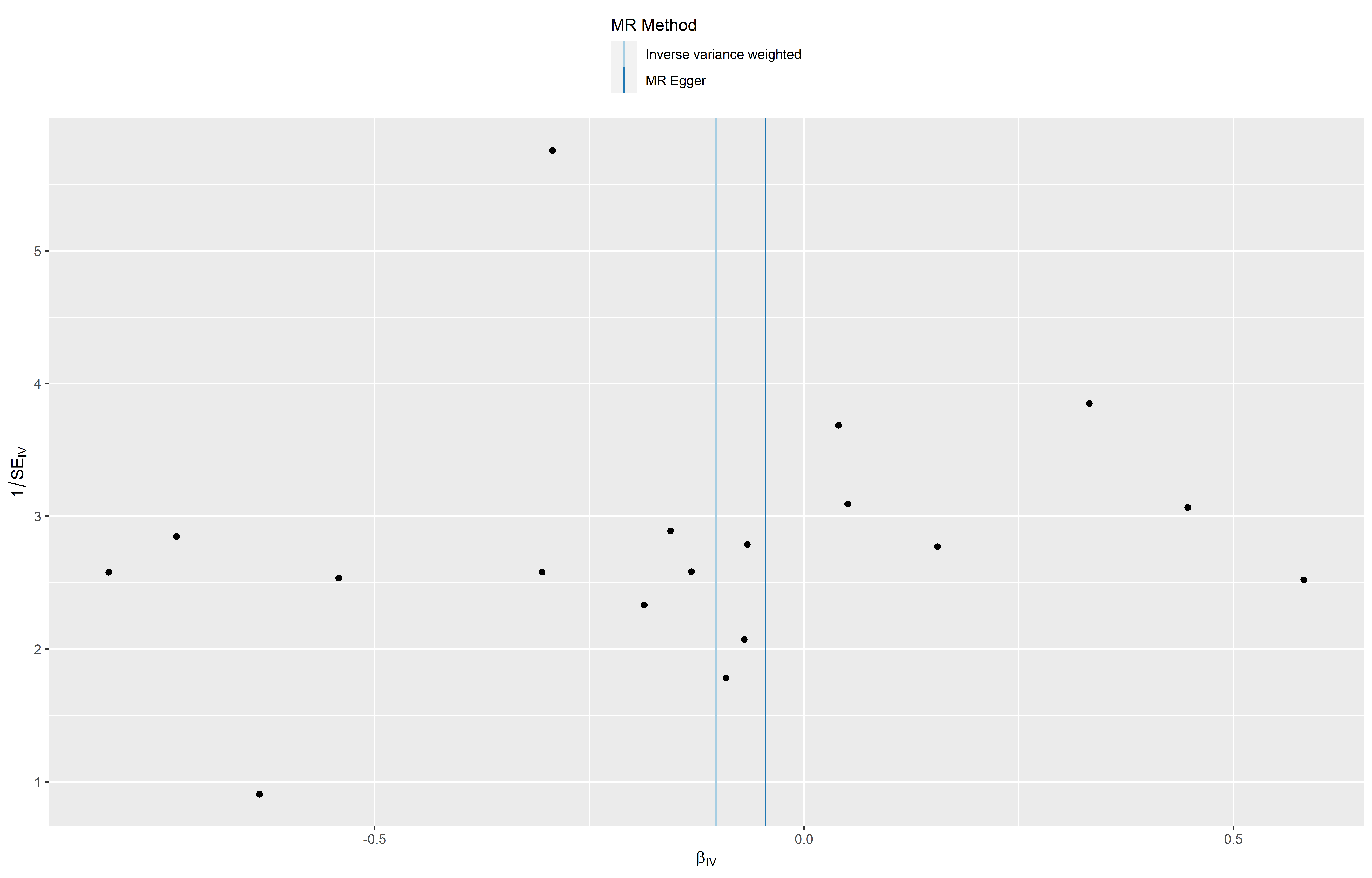

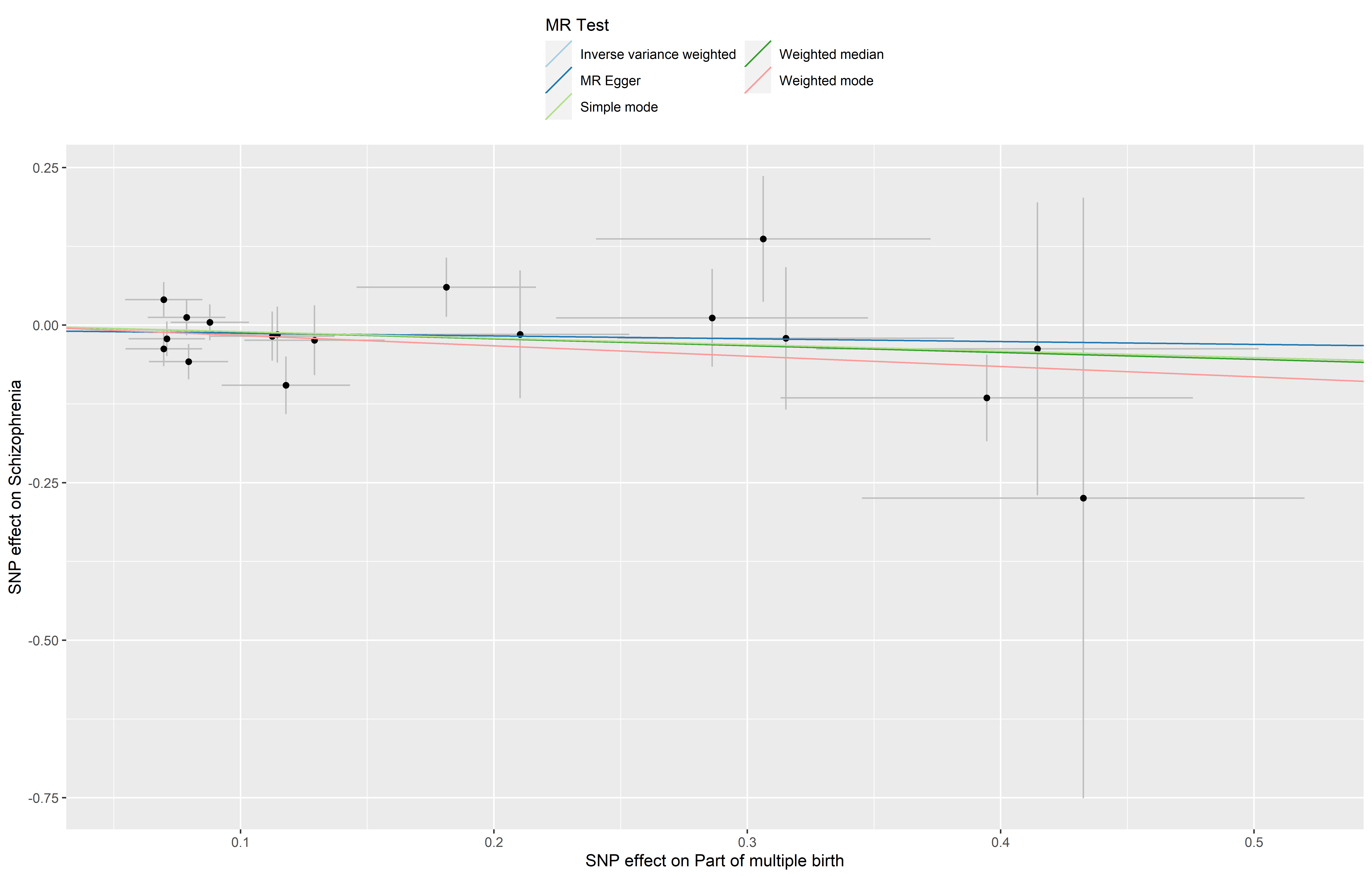


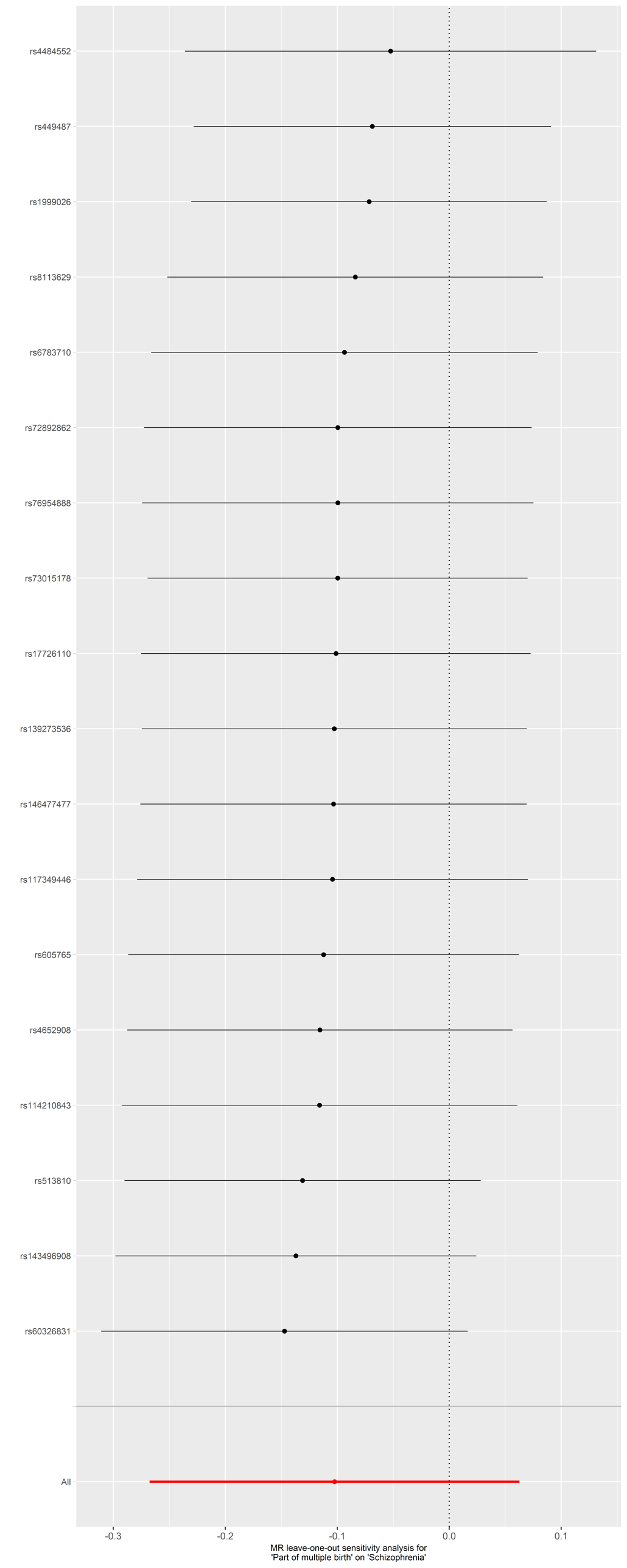


**Schizophrenia – UK Biobank**


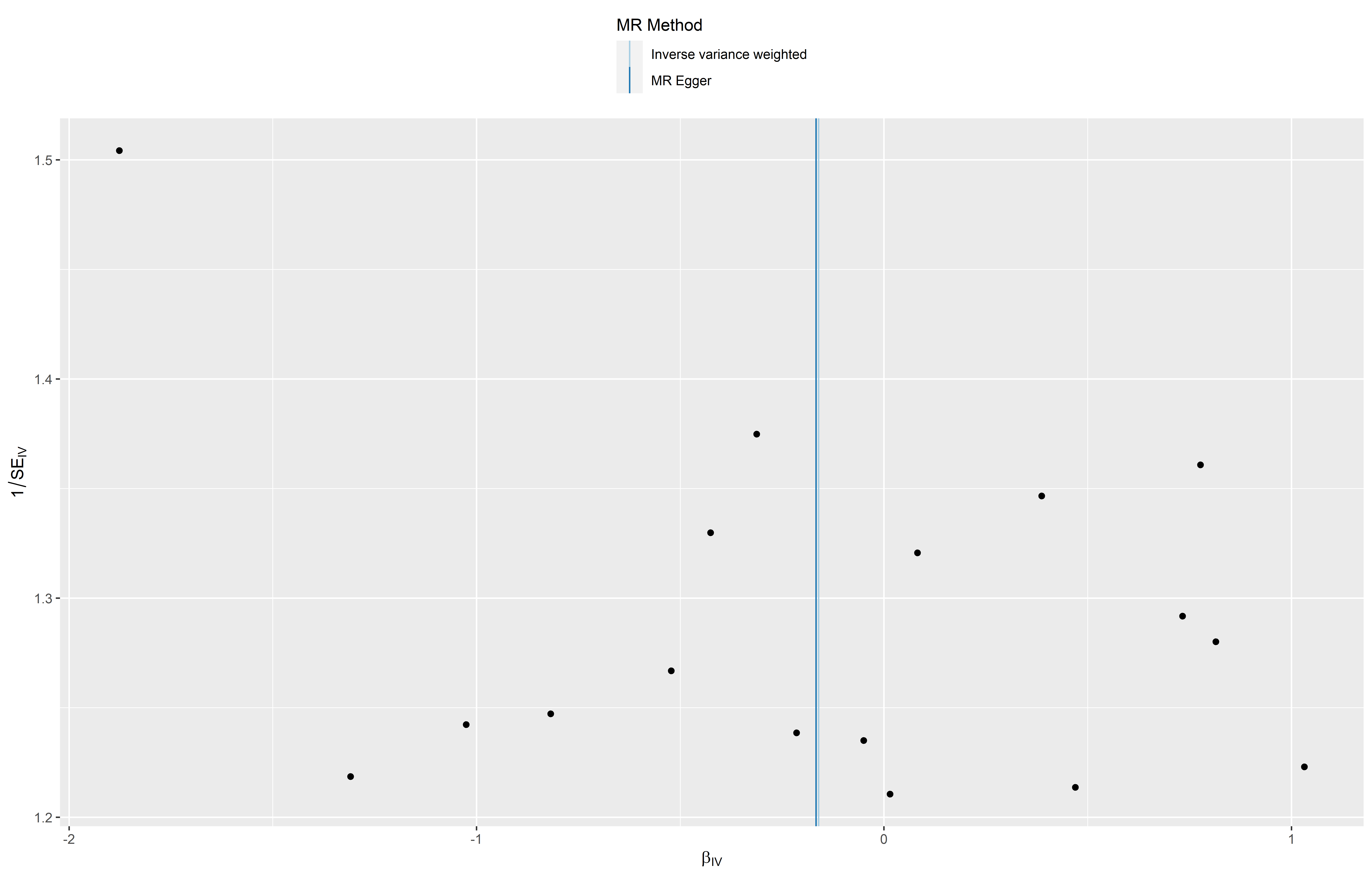

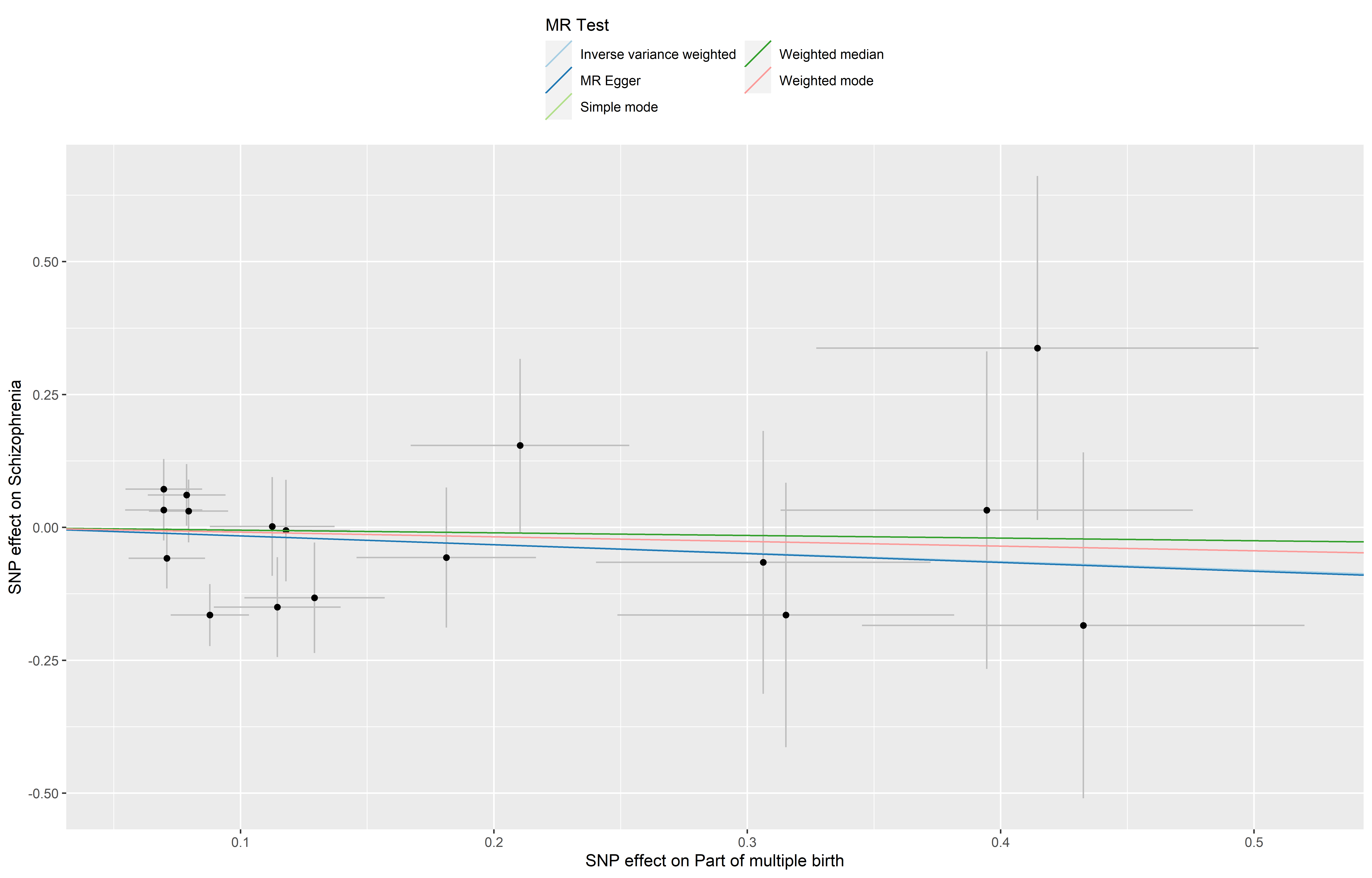


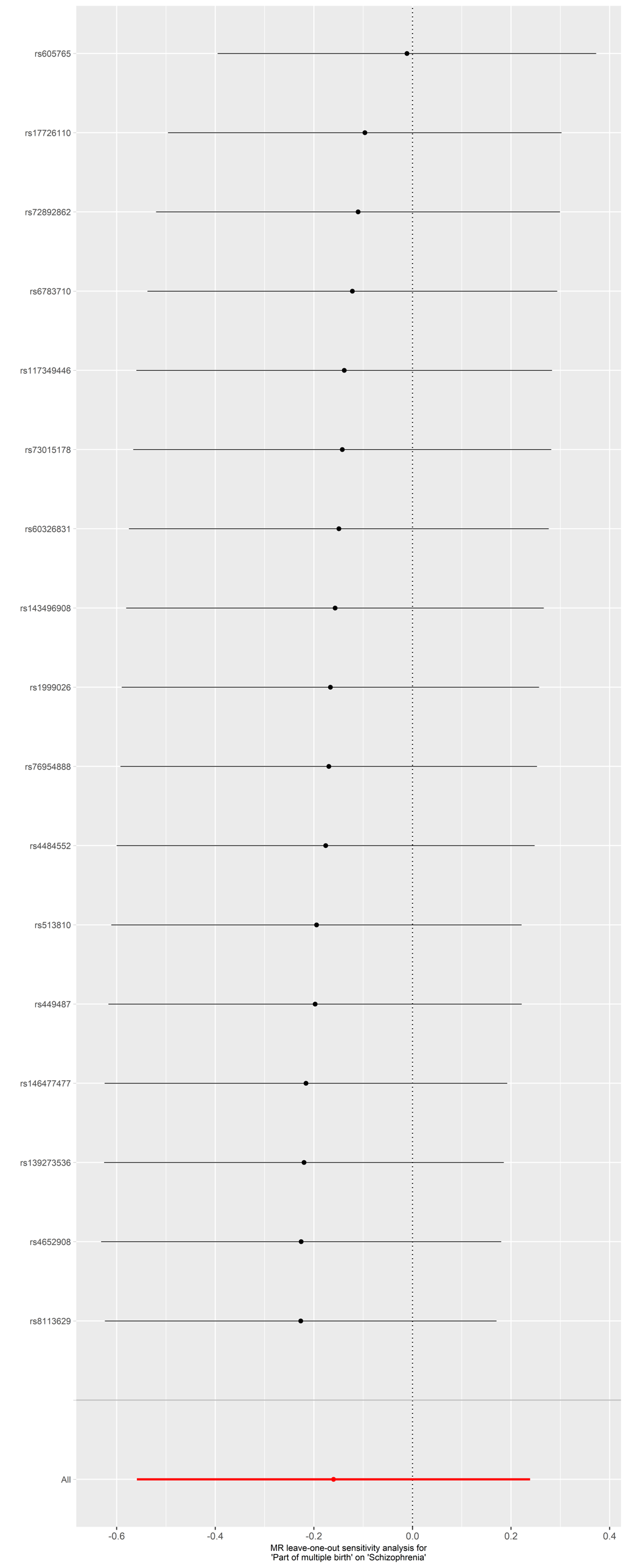


**Mood disorders – FinnGen**


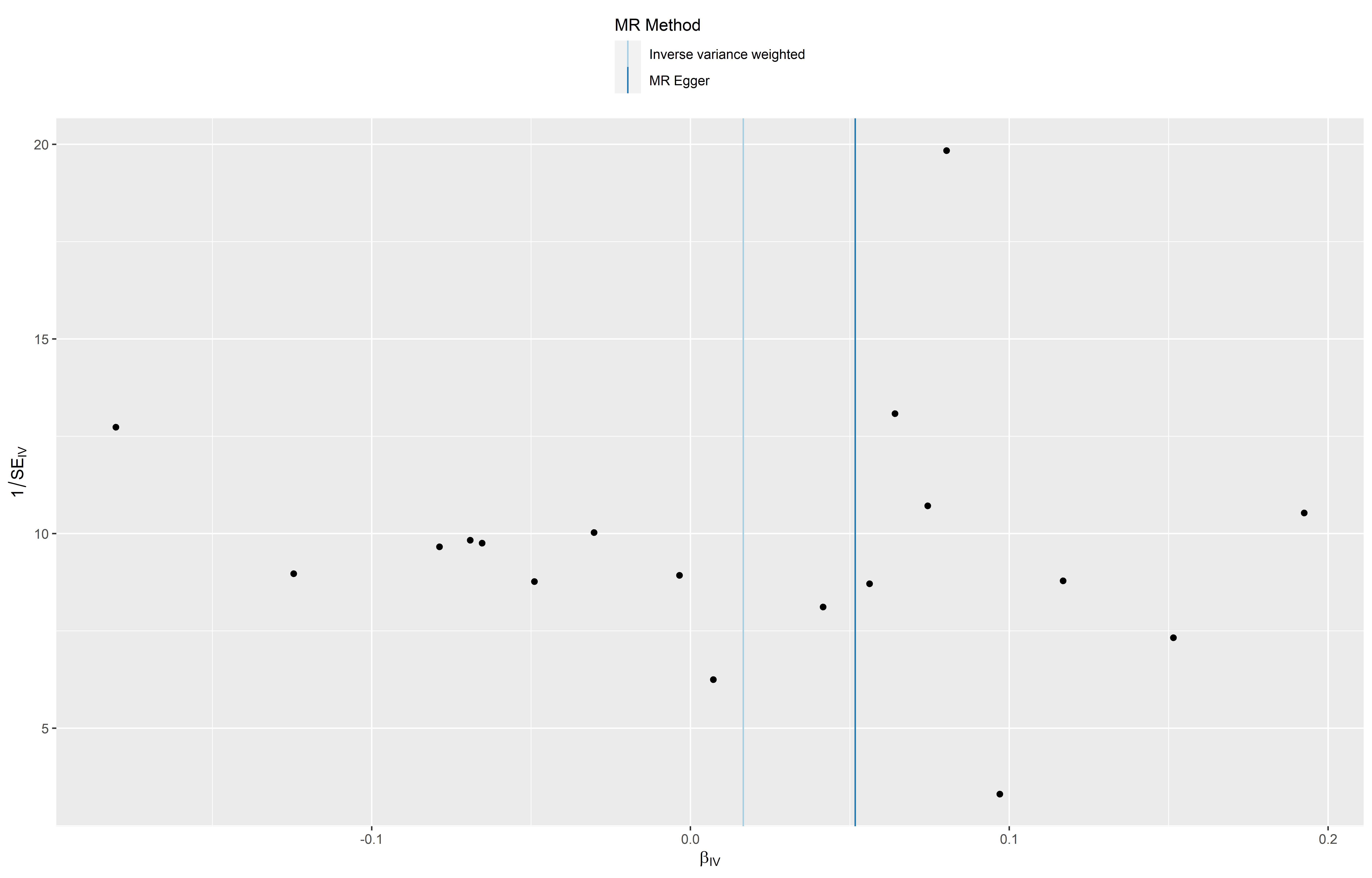

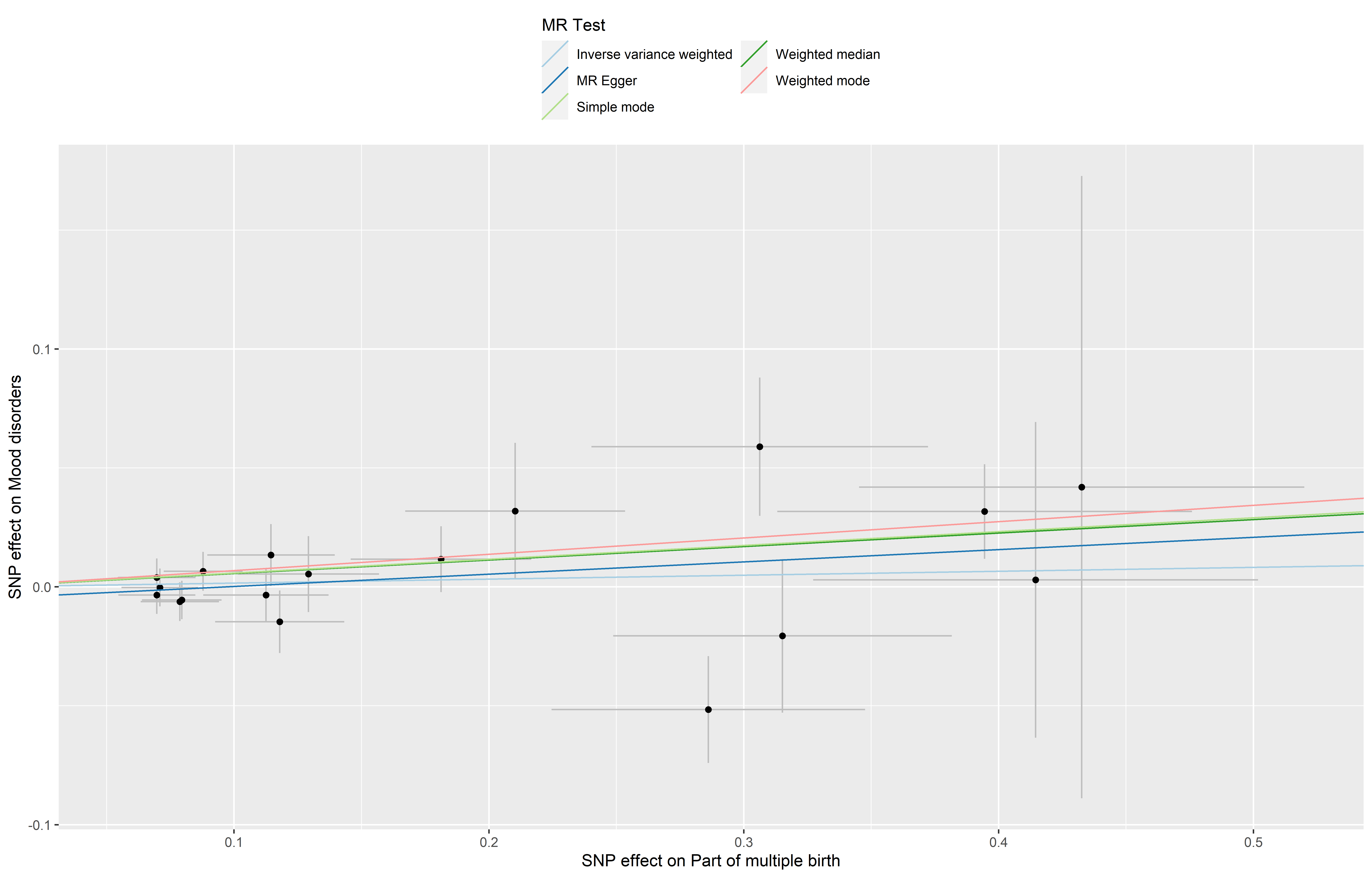


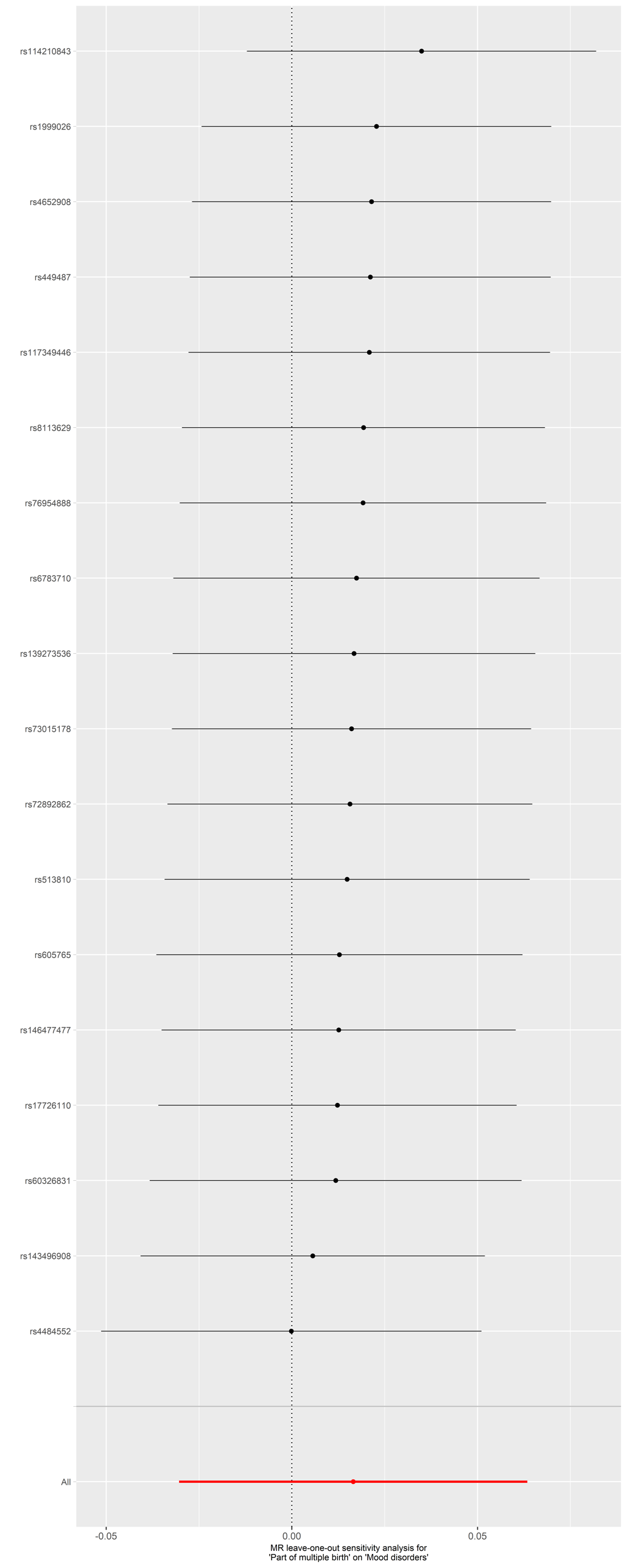


**Mood disorders – UK Biobank**


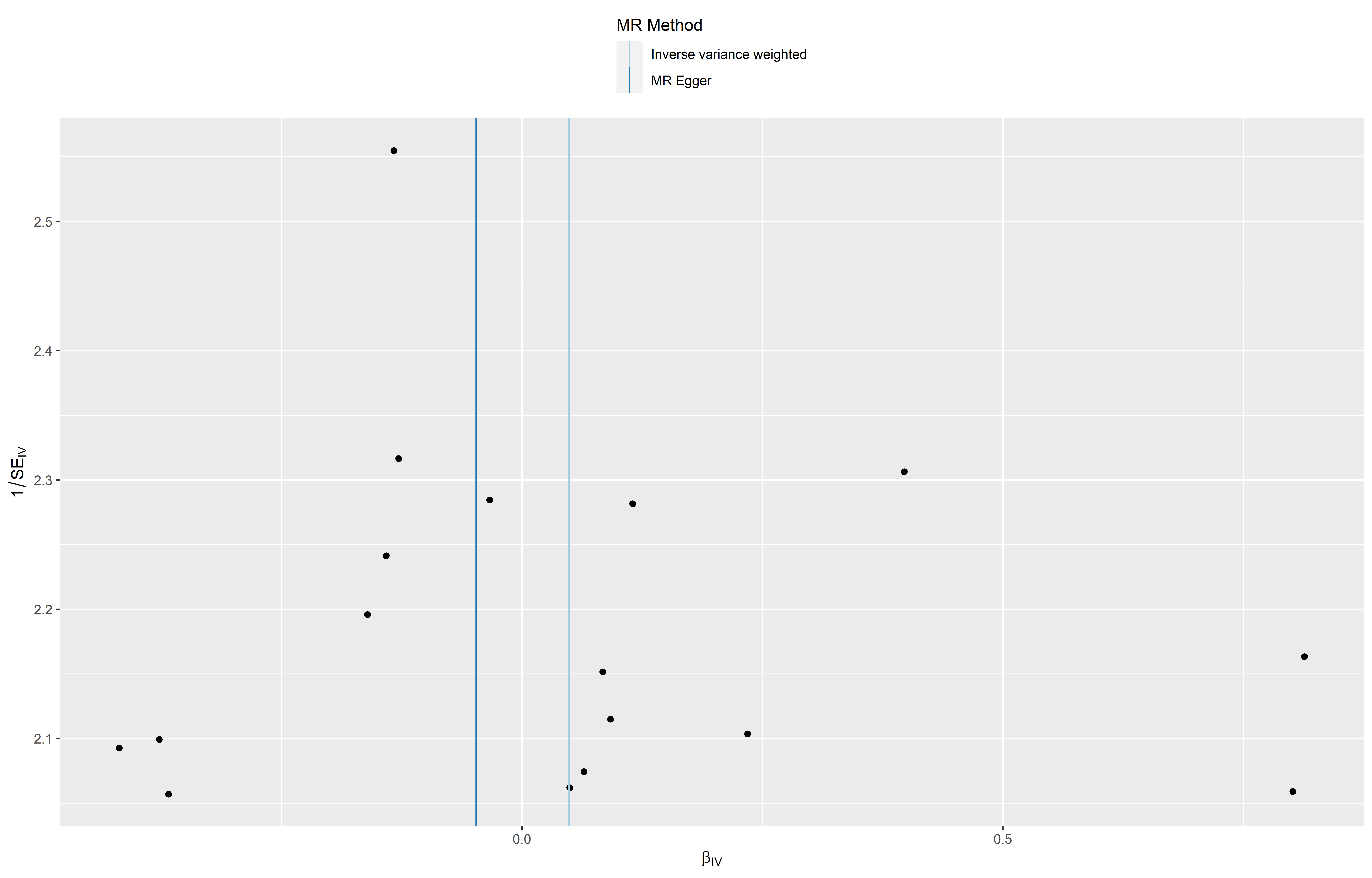

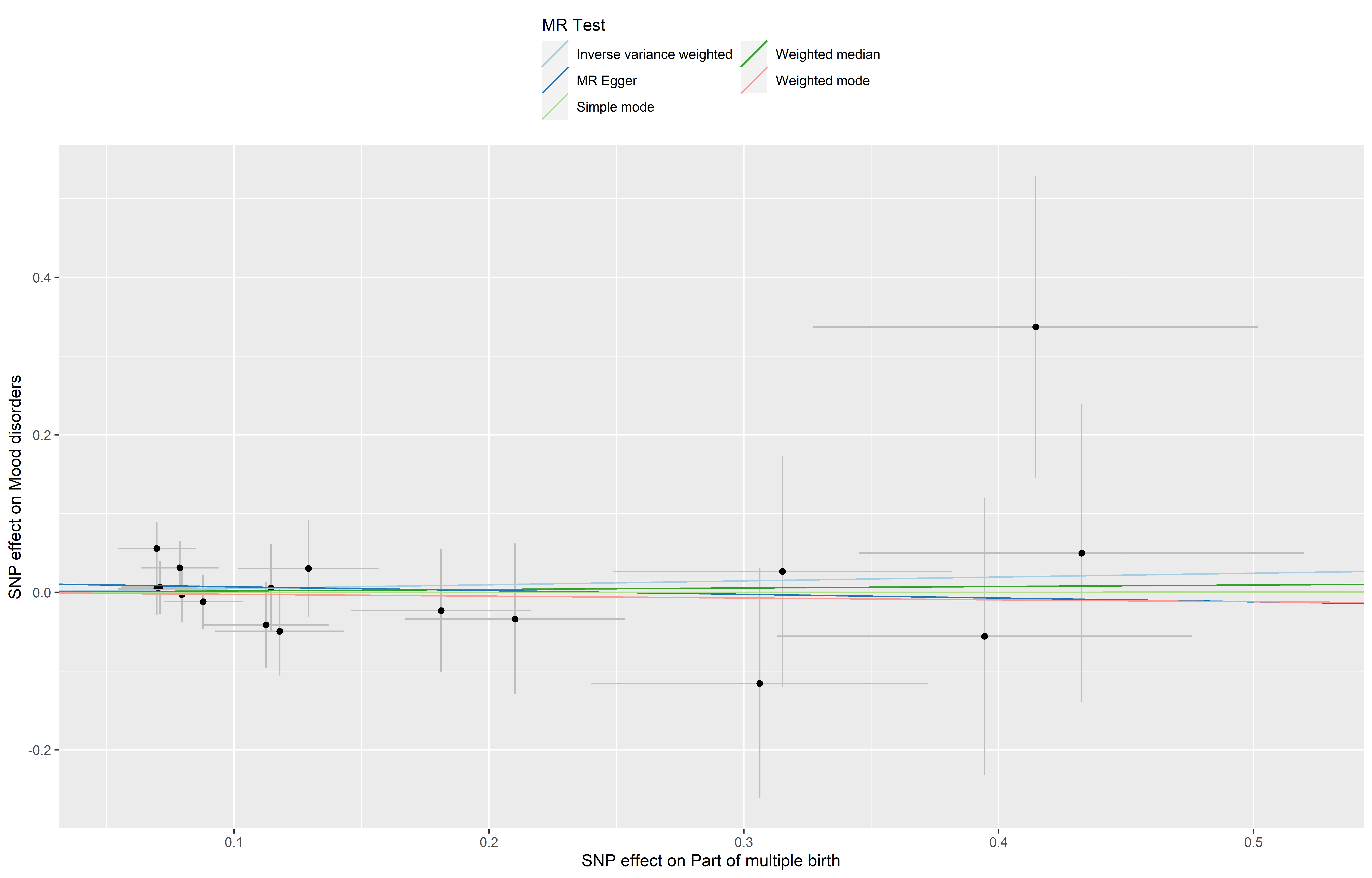


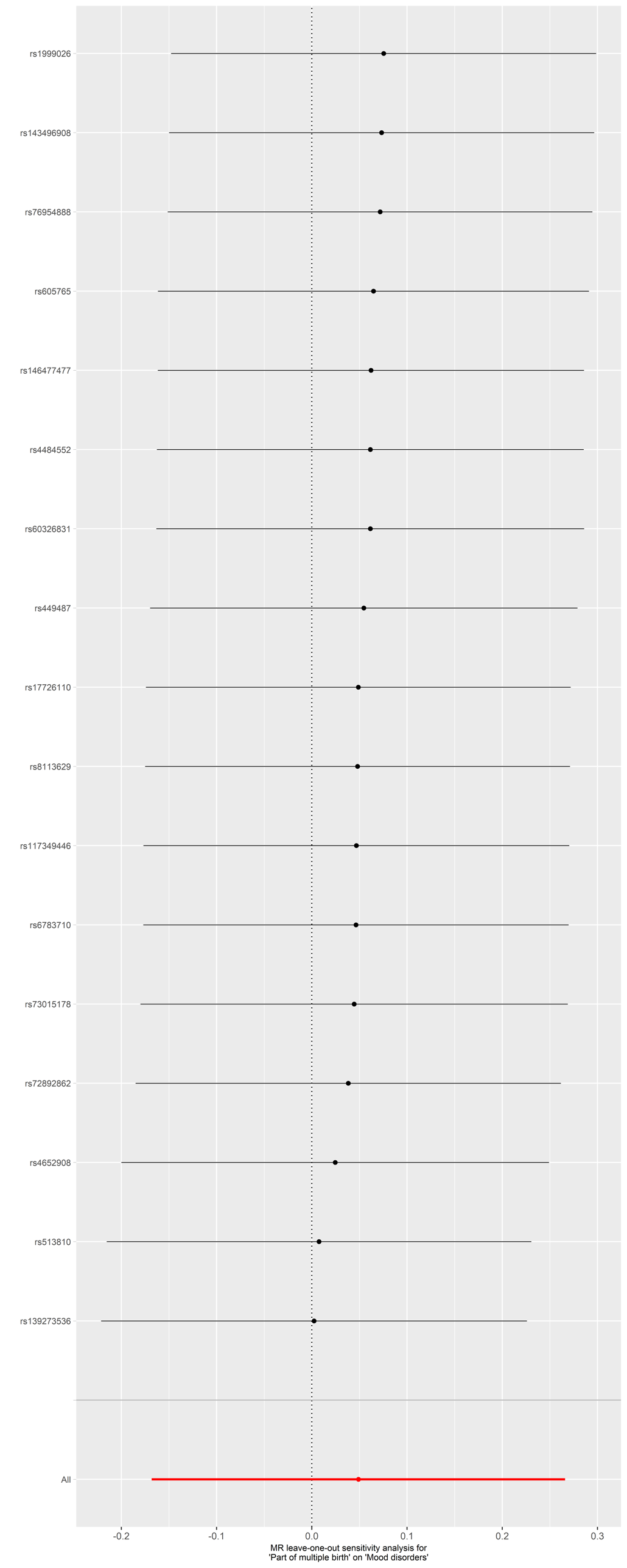


**Anxiety disorders – FinnGen**


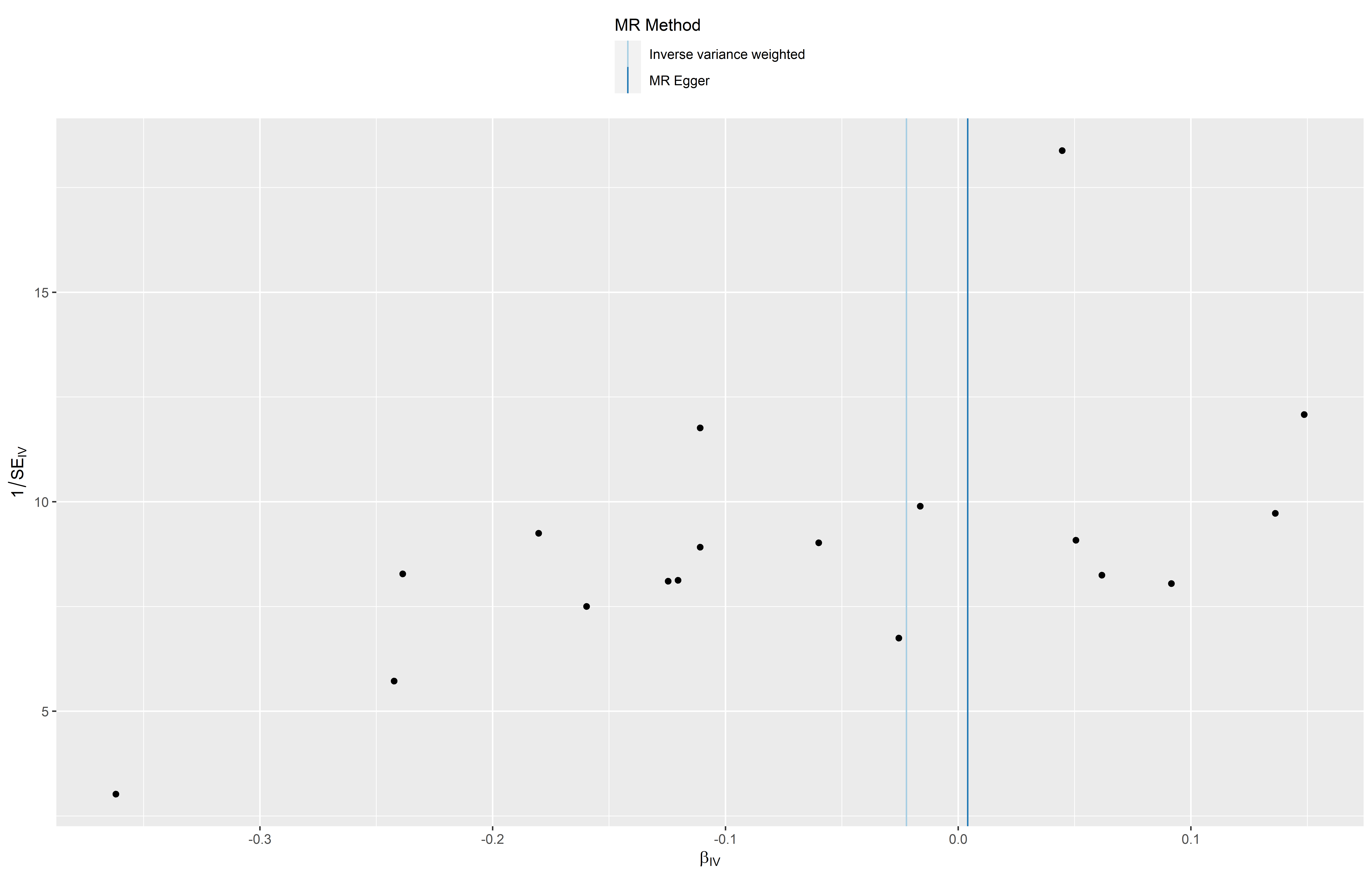

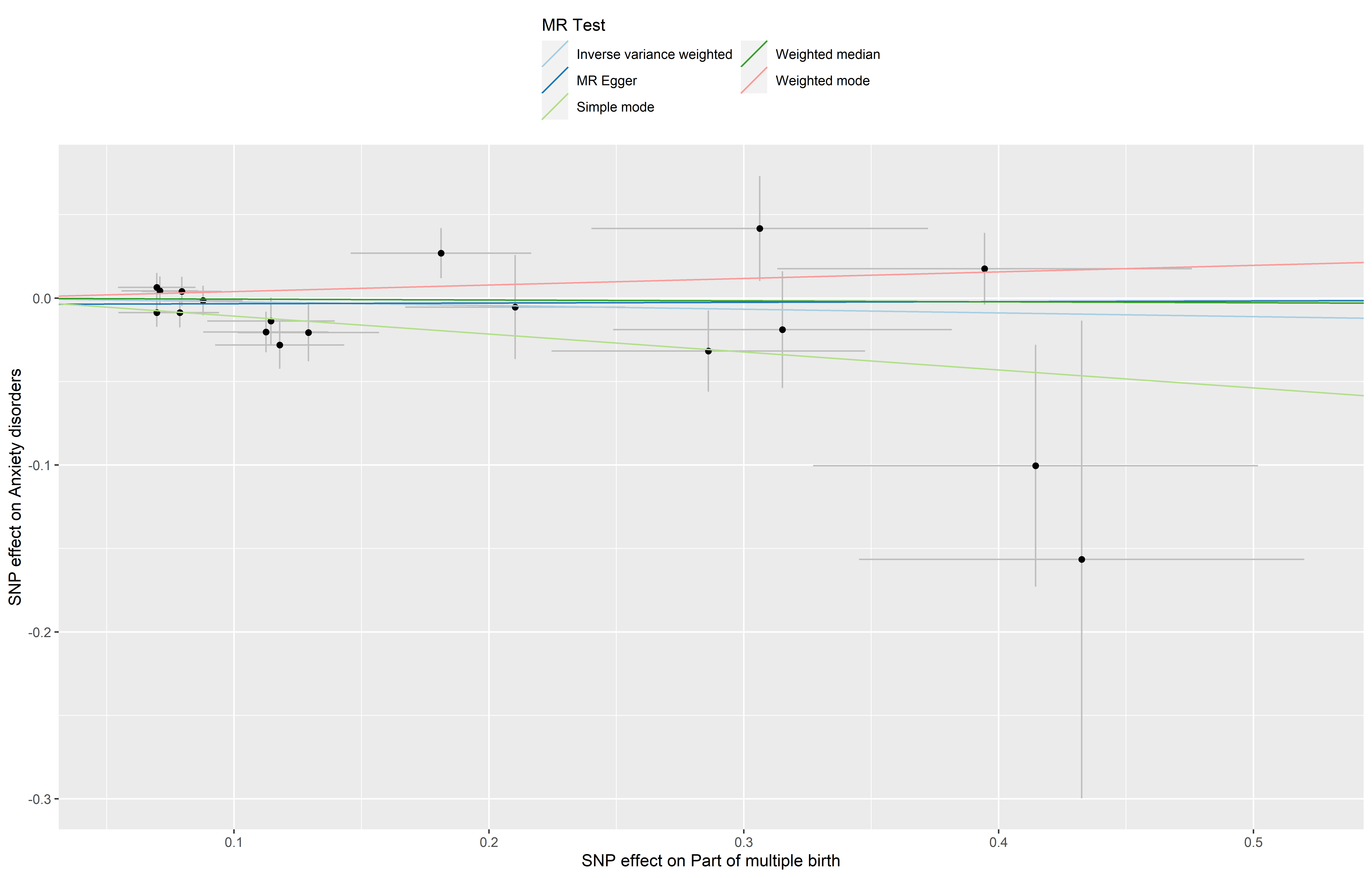


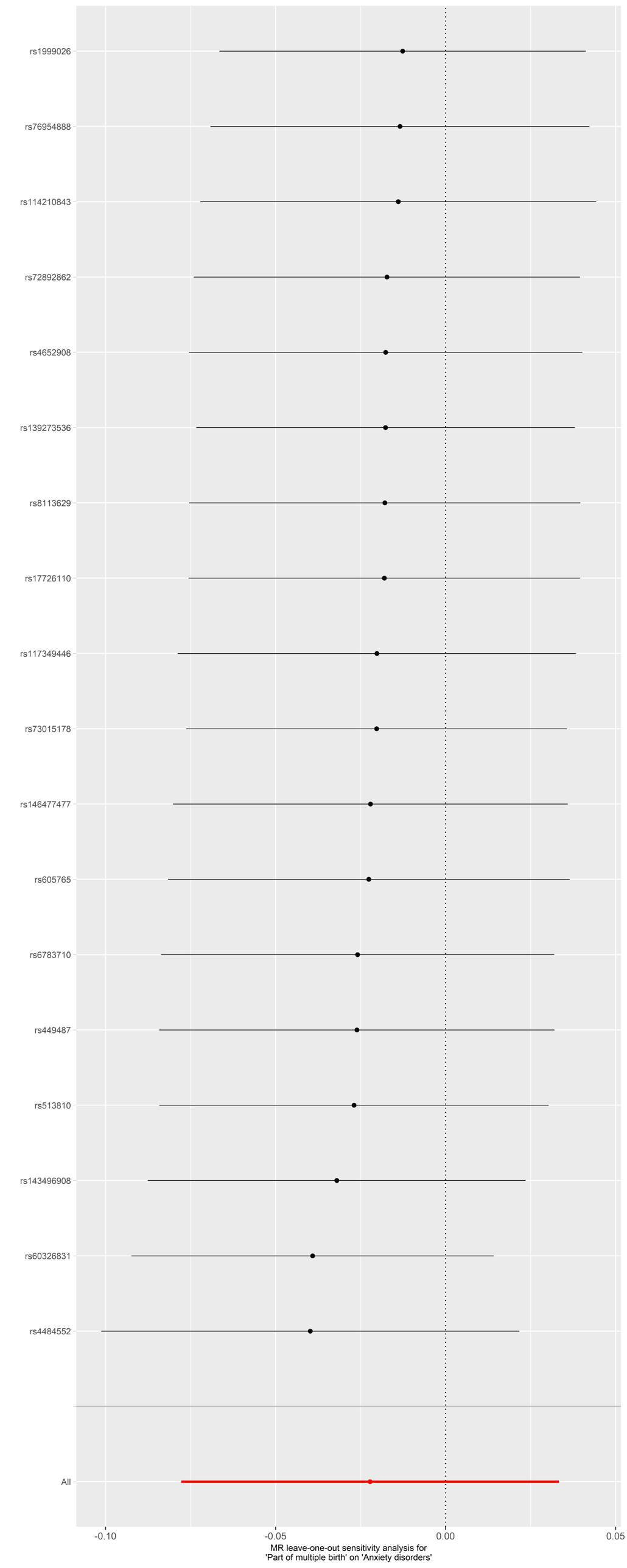


**Anxiety disorders – UK Biobank**


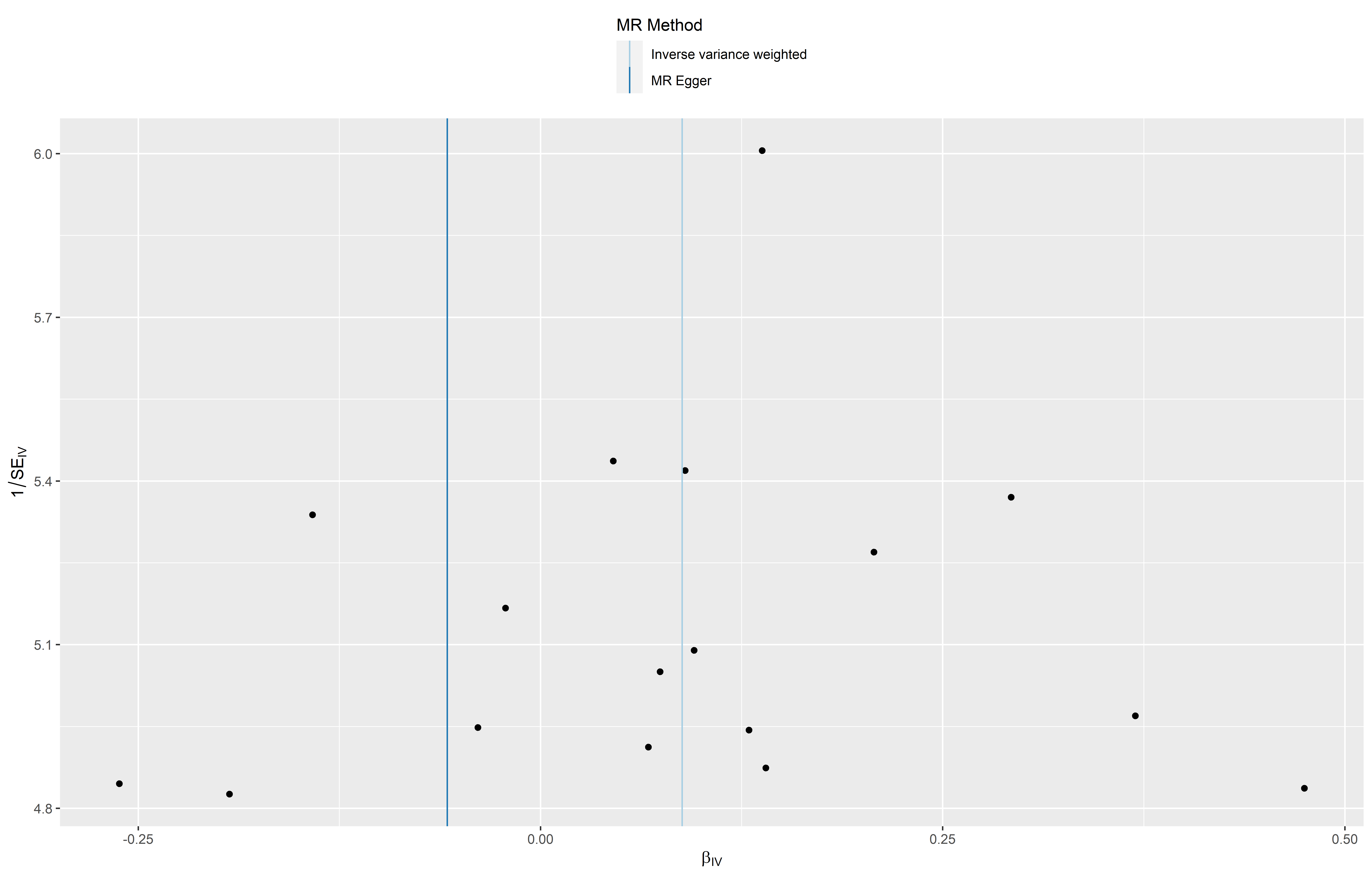

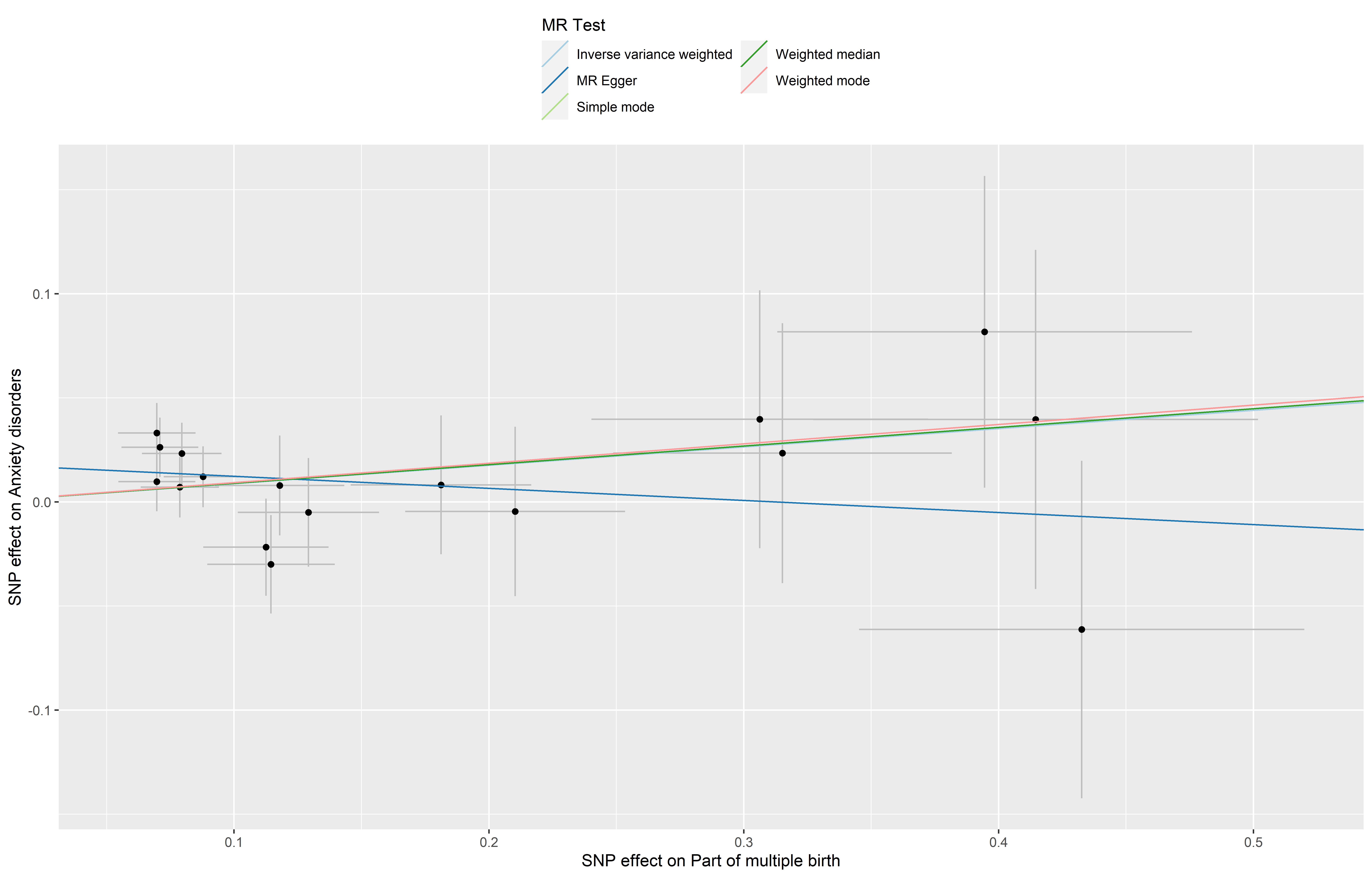


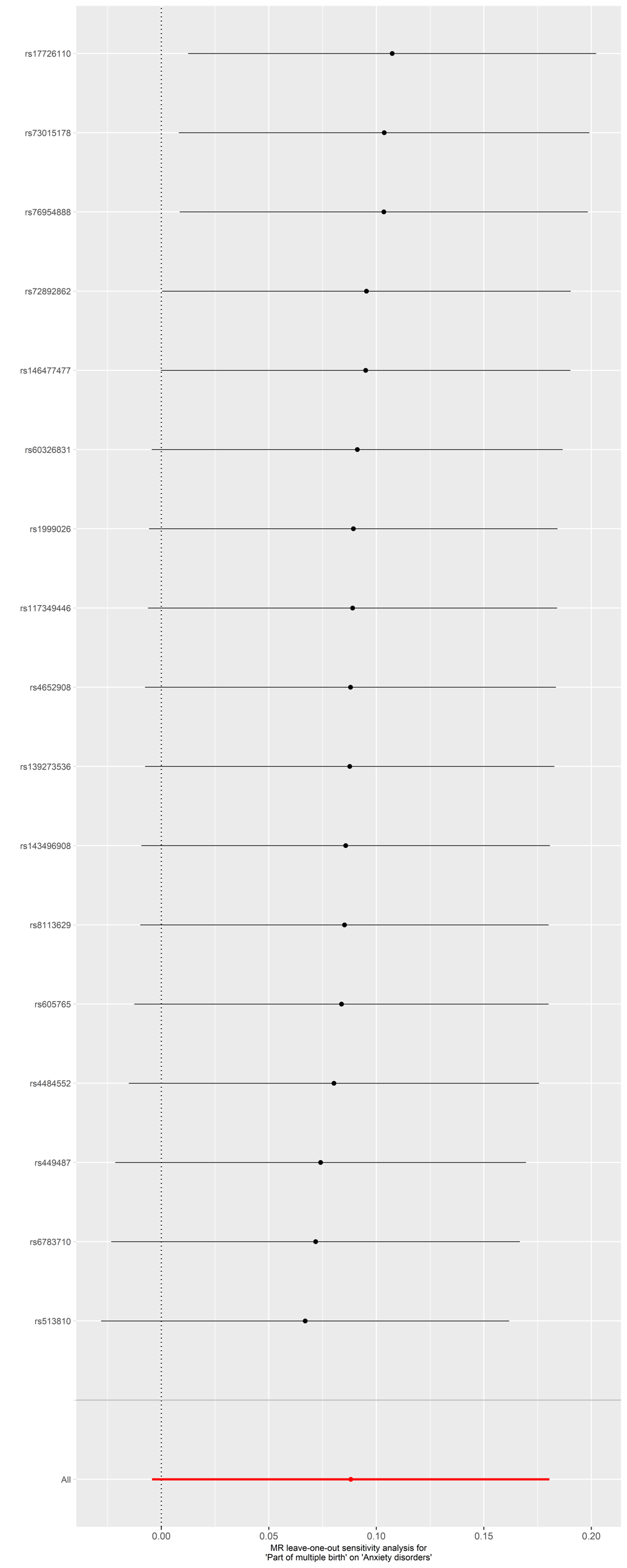


**Suicide or self-inflicted injury – FinnGen**


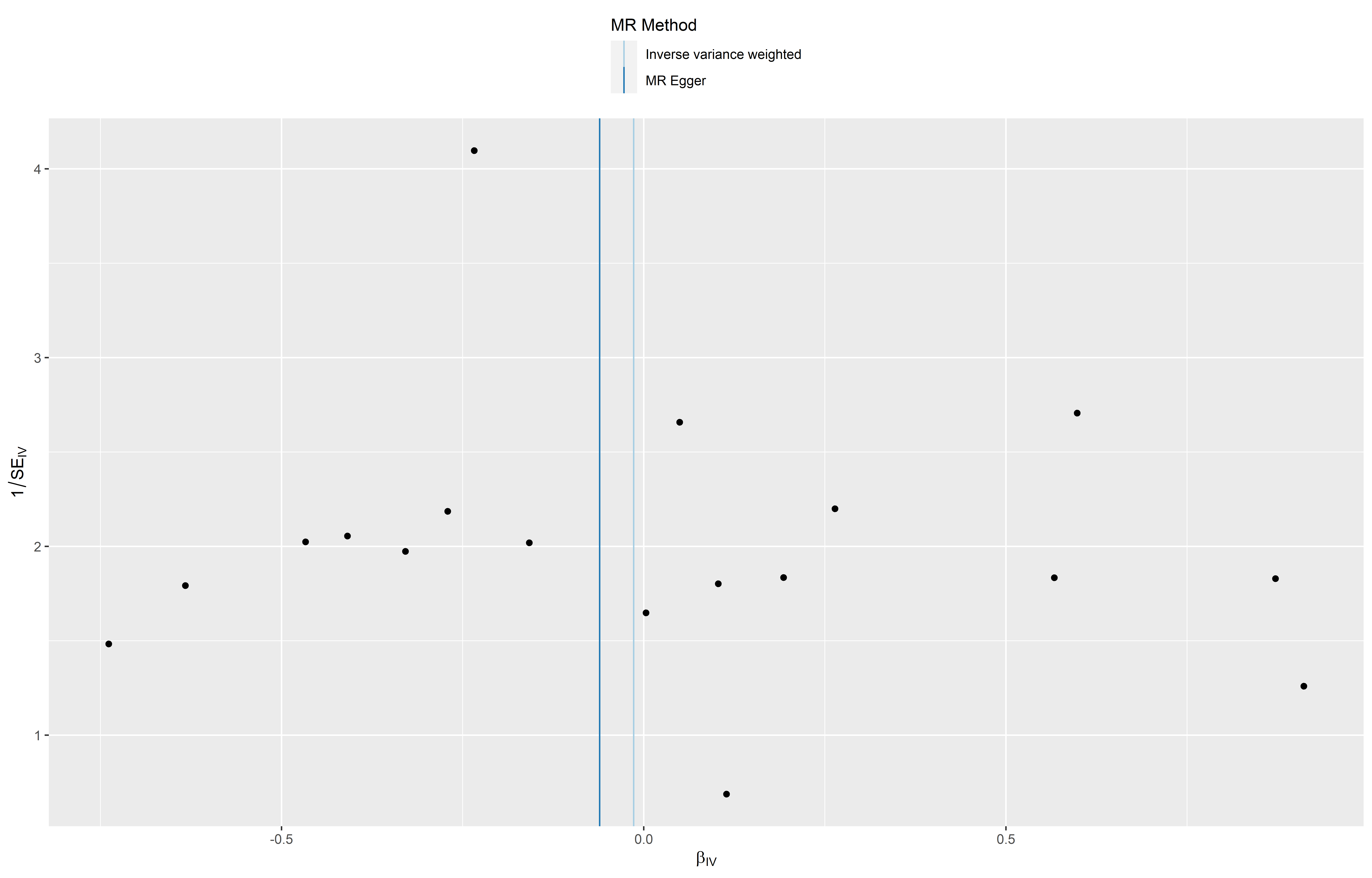

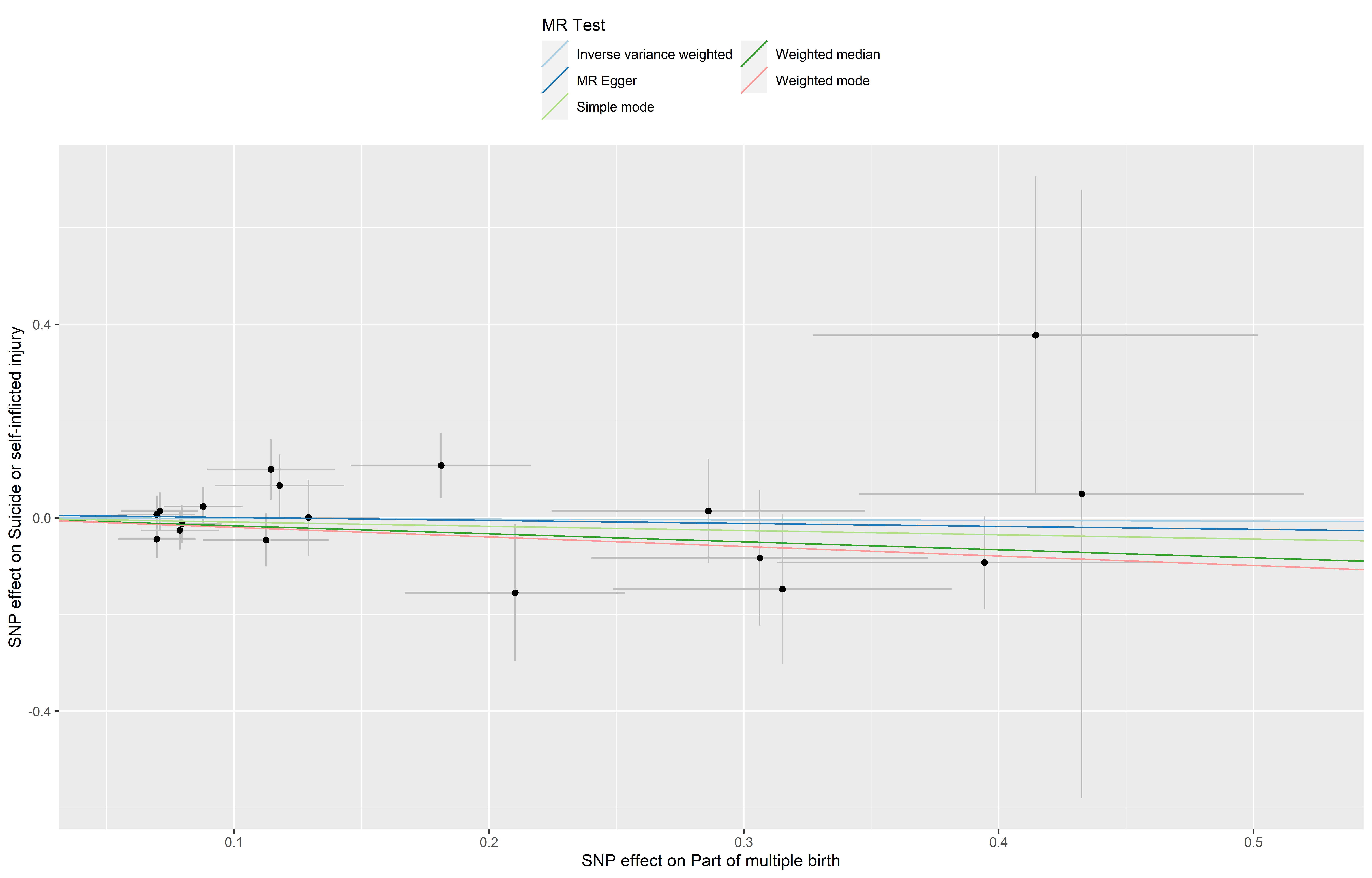


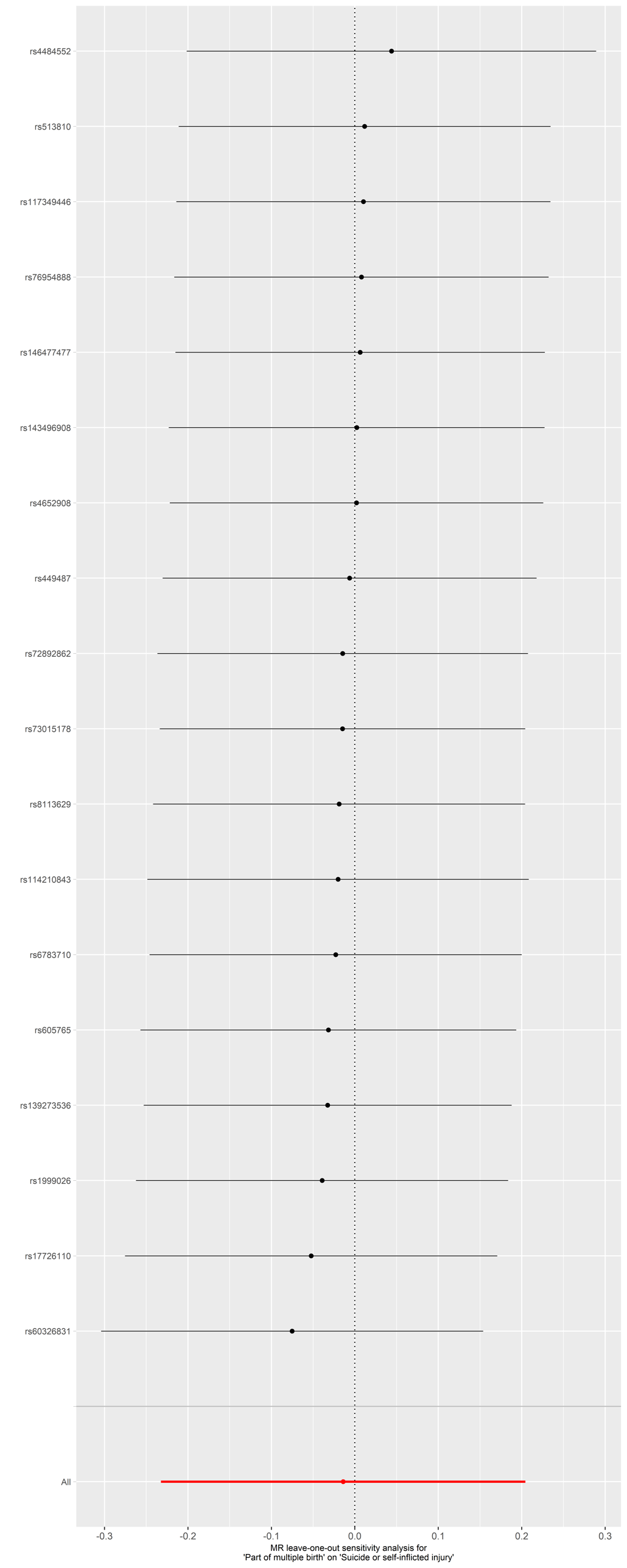


**Suicide or self-inflicted injury – UK Biobank**


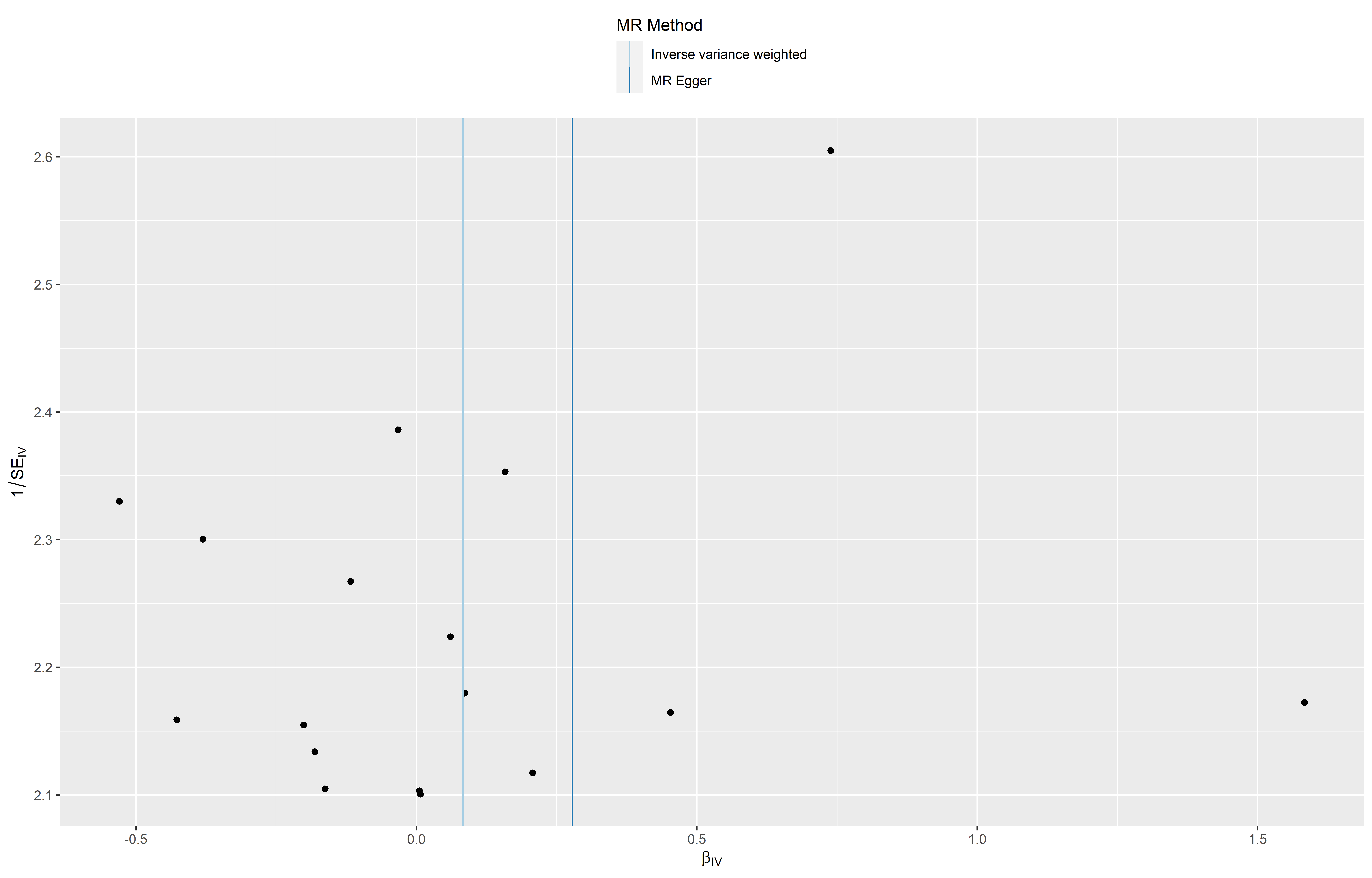

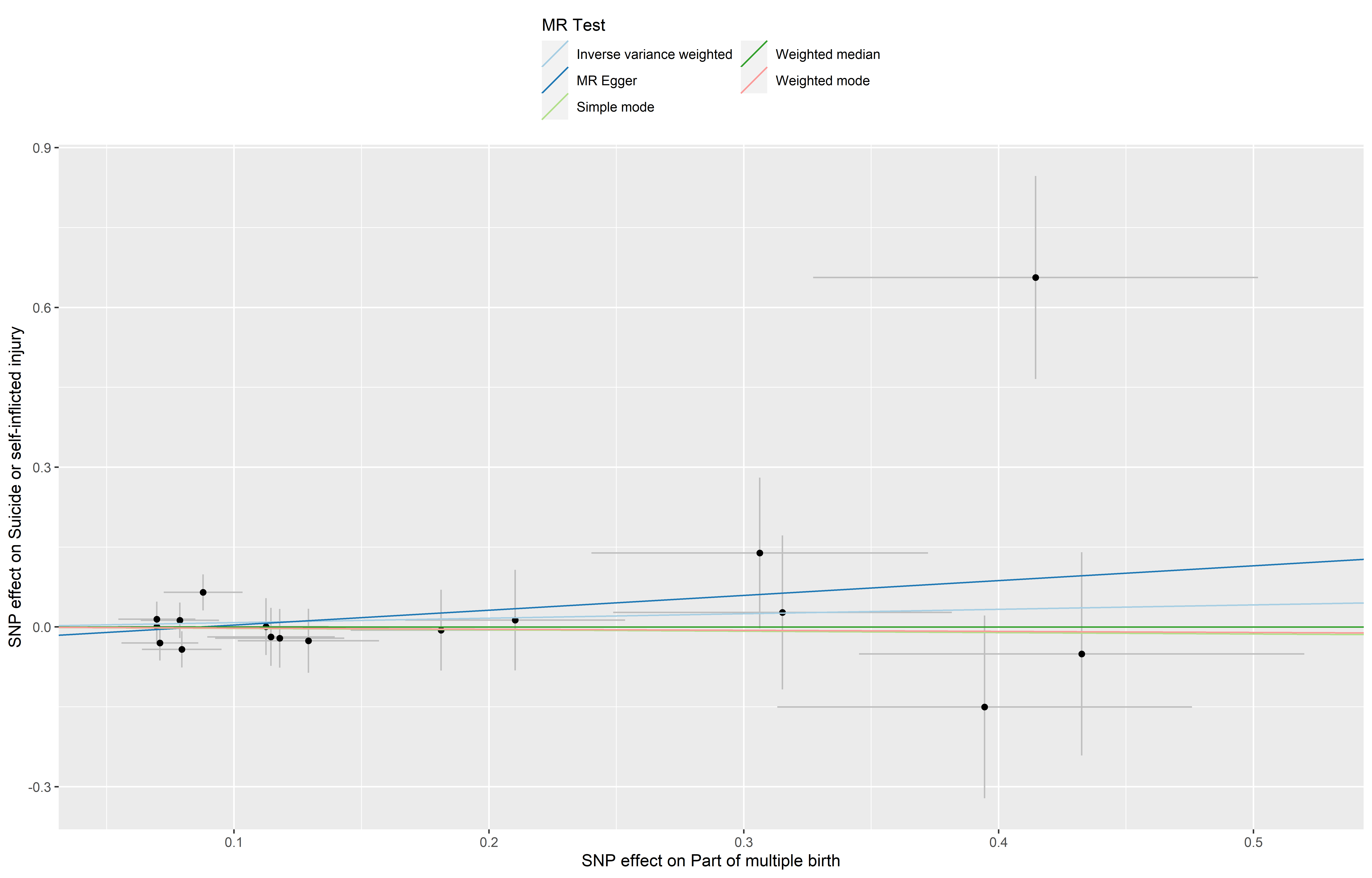


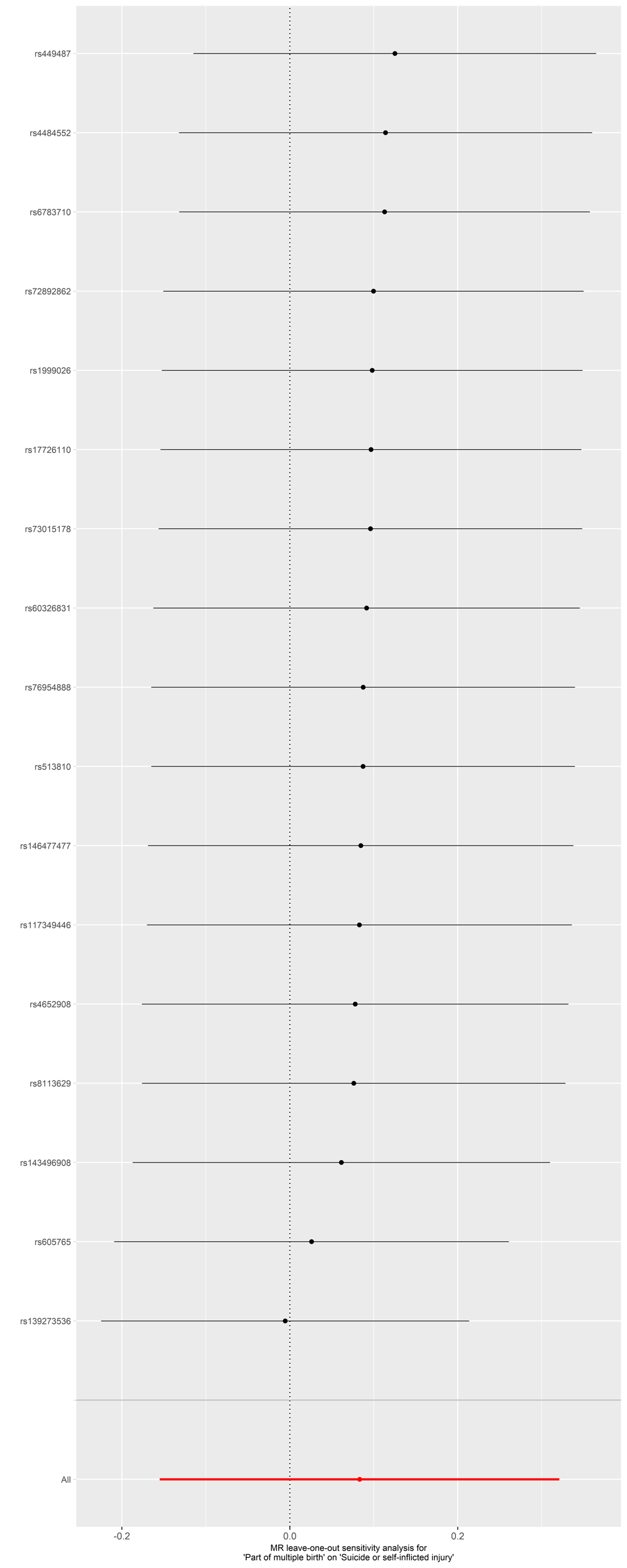

Supplement: Supplementary file 9 — Additional file 9: Material S1. The scatter plot, funnel plot and leave-one-out plot for the MR analysis of multiple birth and mental illness. [file 12967_2023_4423_MOESM9_ESM.docx]
