# Supplementary material for: Long-term outcomes of offspring from multiple gestations: a two-sample Mendelian randomization study on multi-system diseases using UK Biobank and FinnGen databases
Source: J Transl Med. 2023 Sep 8;21:608. doi: 10.1186/s12967-023-04423-w (PMC10492369; doi:10.1186/s12967-023-04423-w)

**Stroke – Finngen**


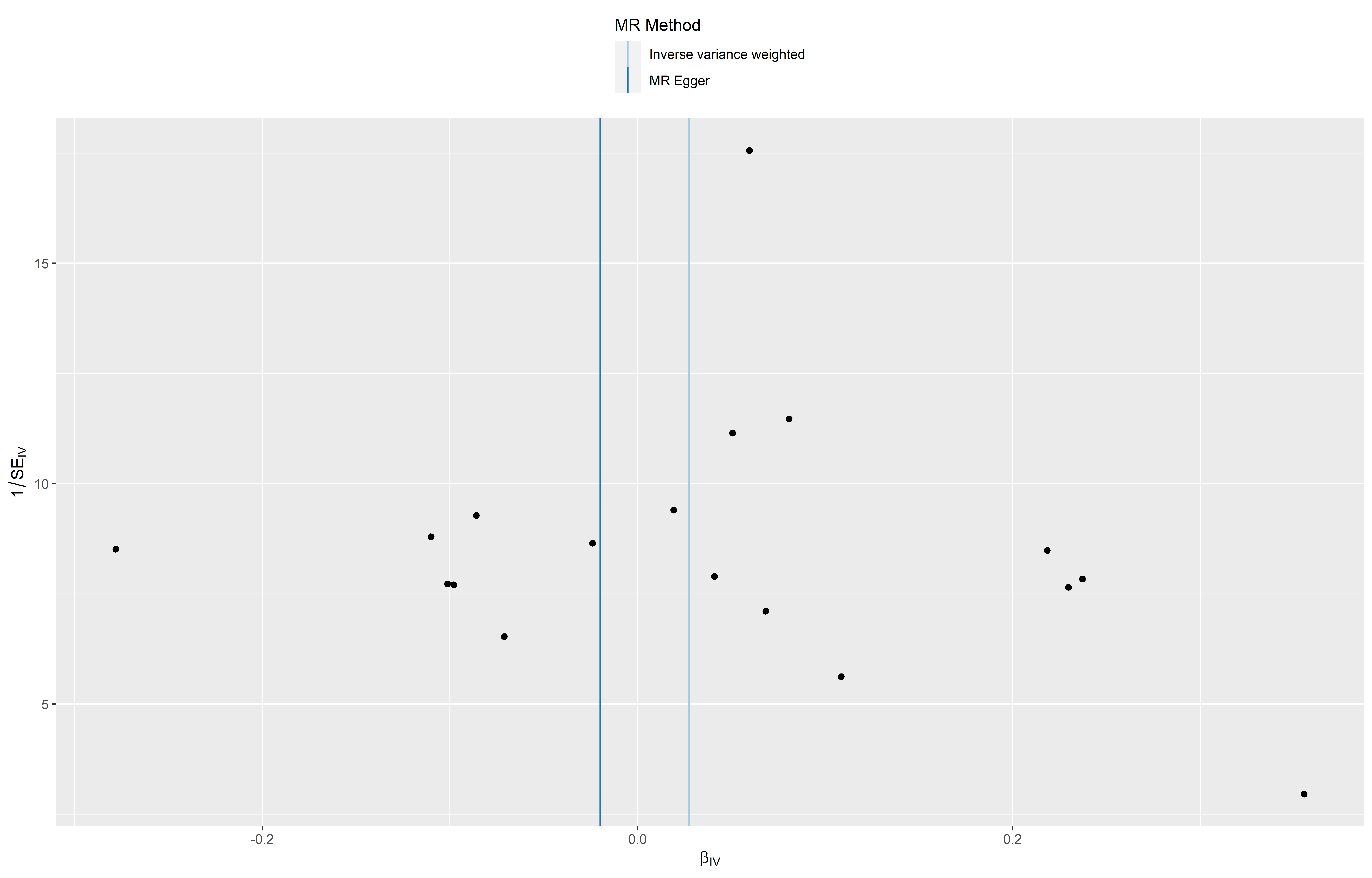

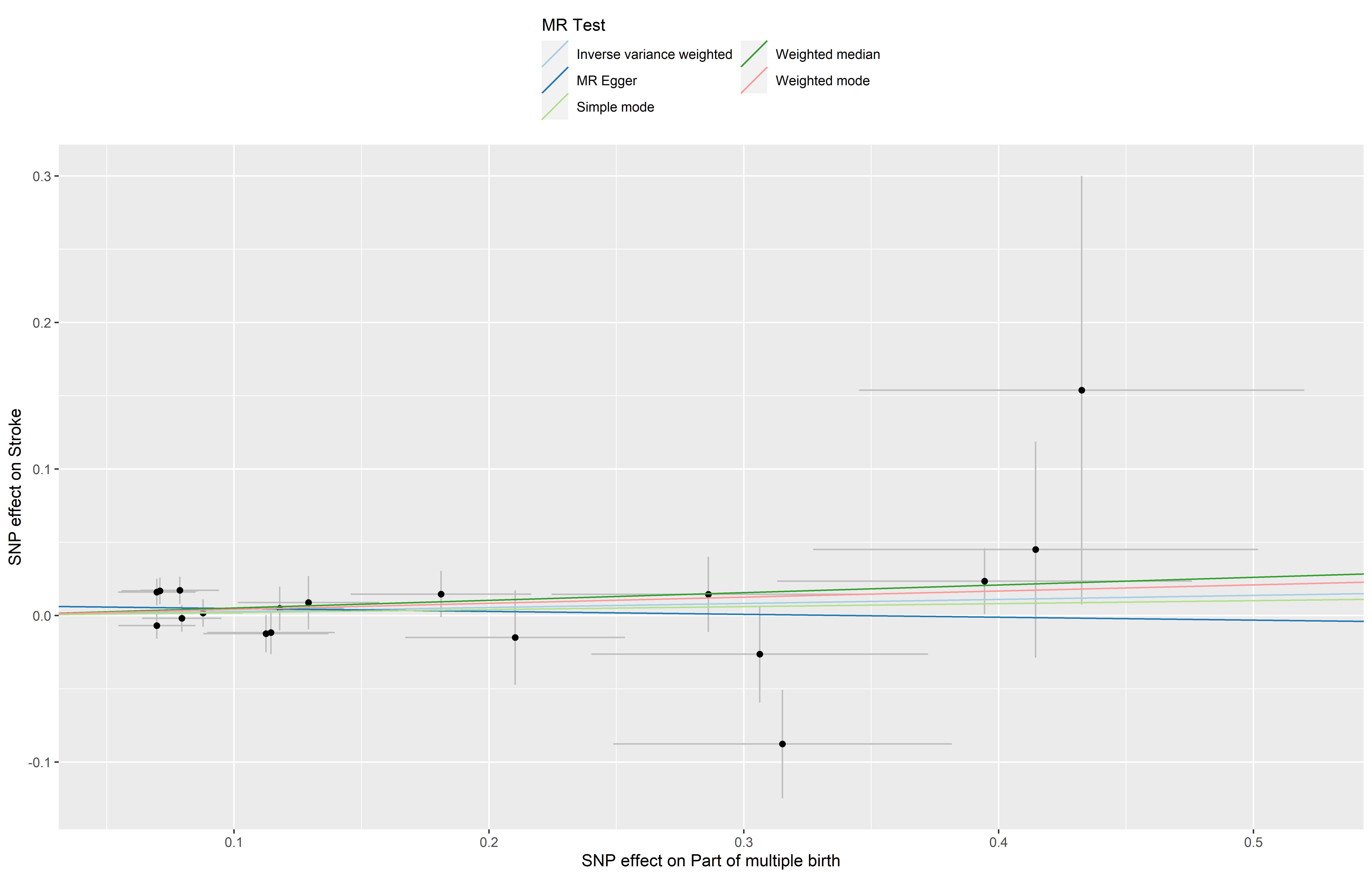


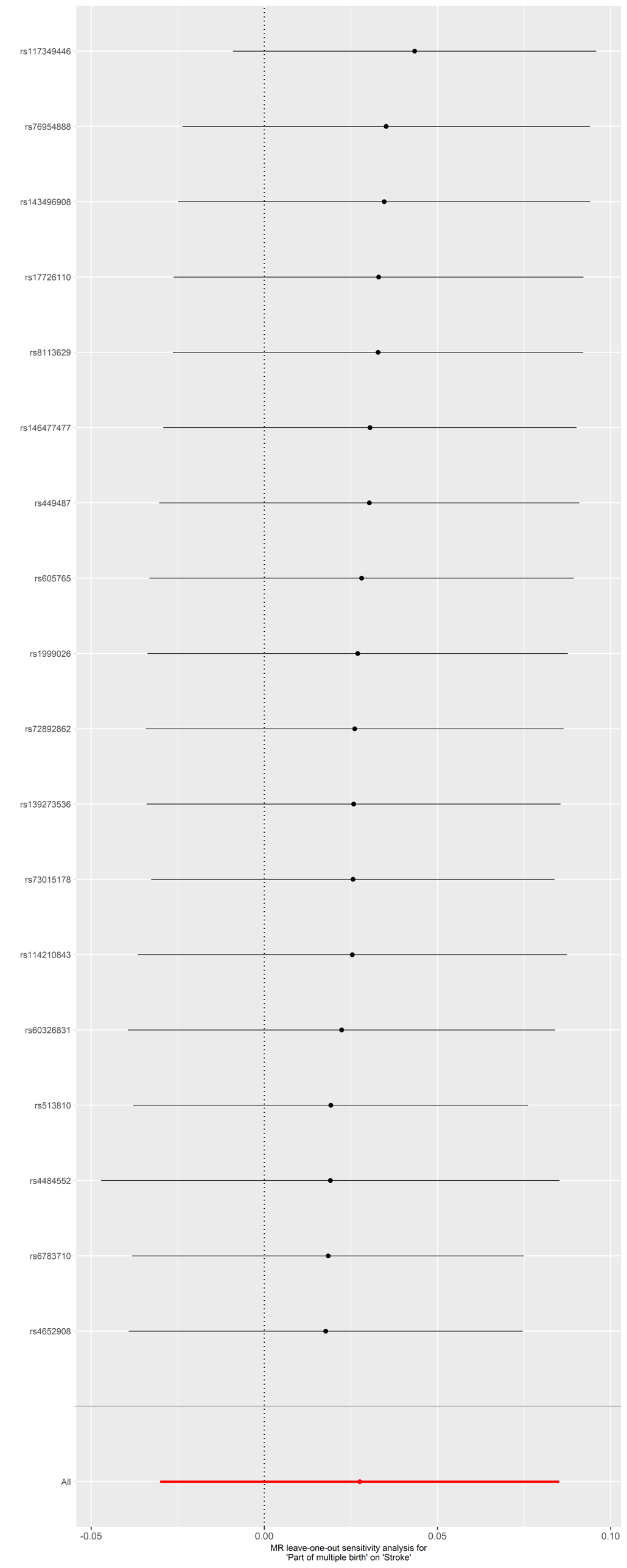


**Stroke – UK Biobank**


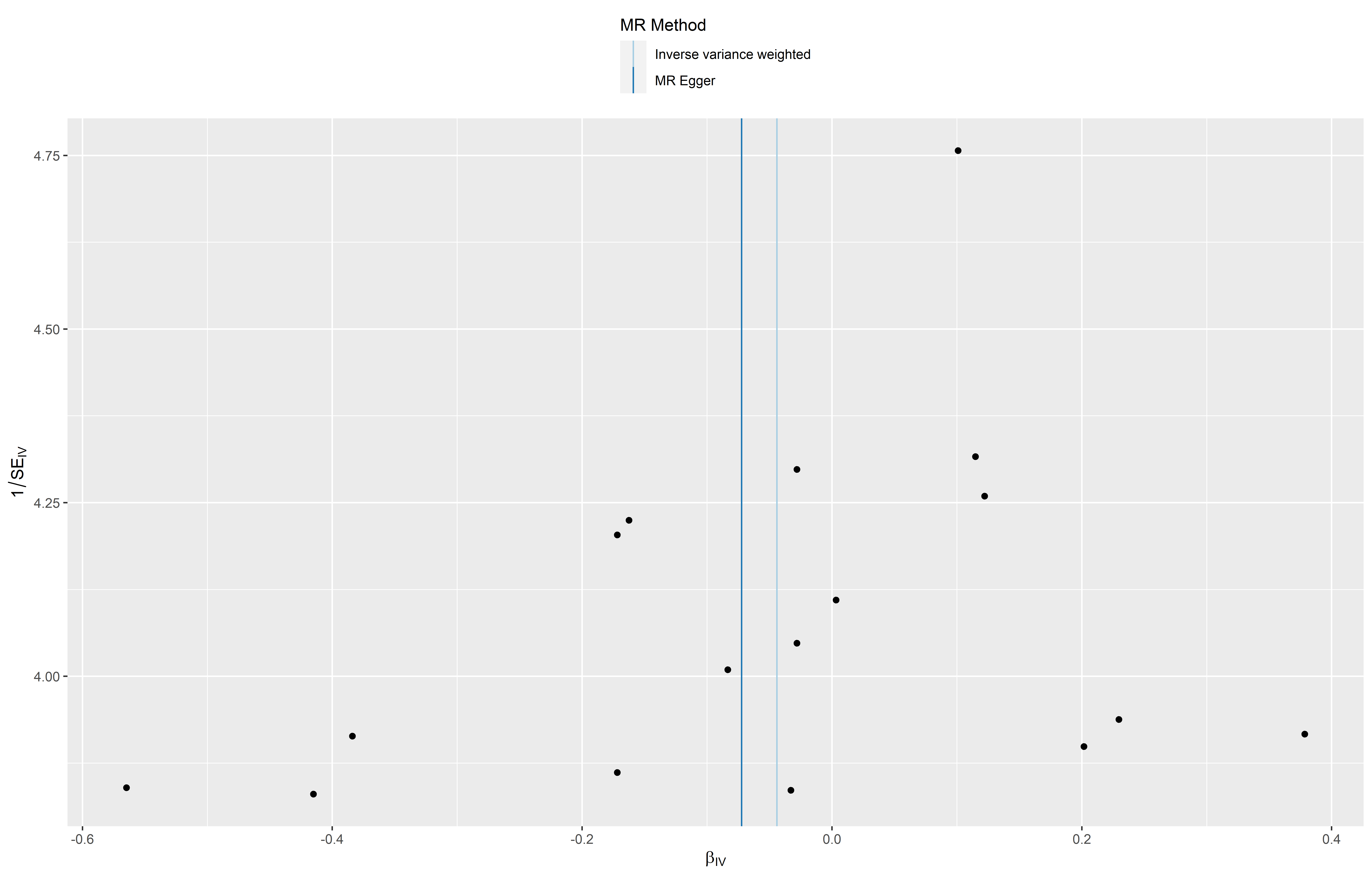

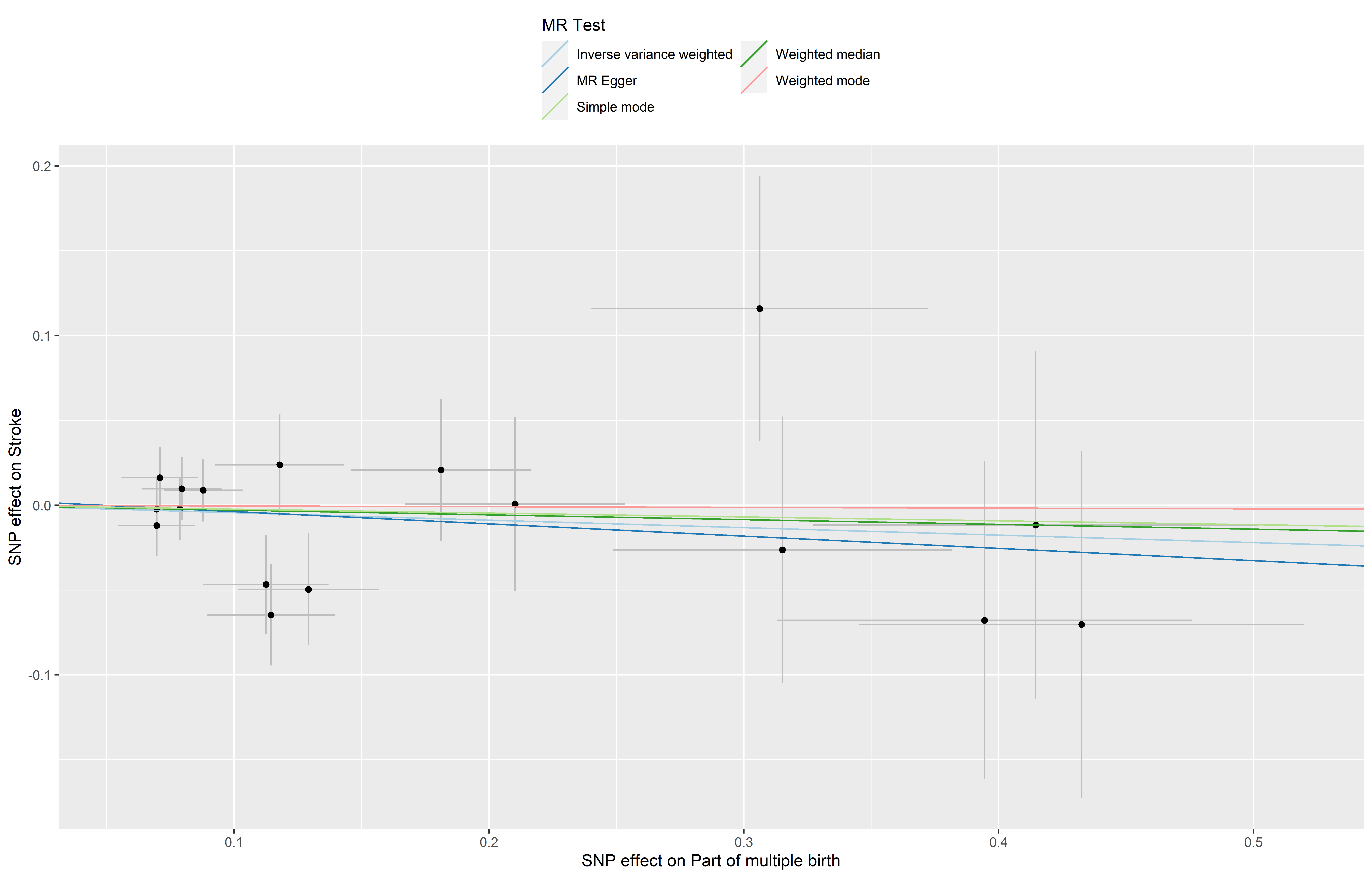


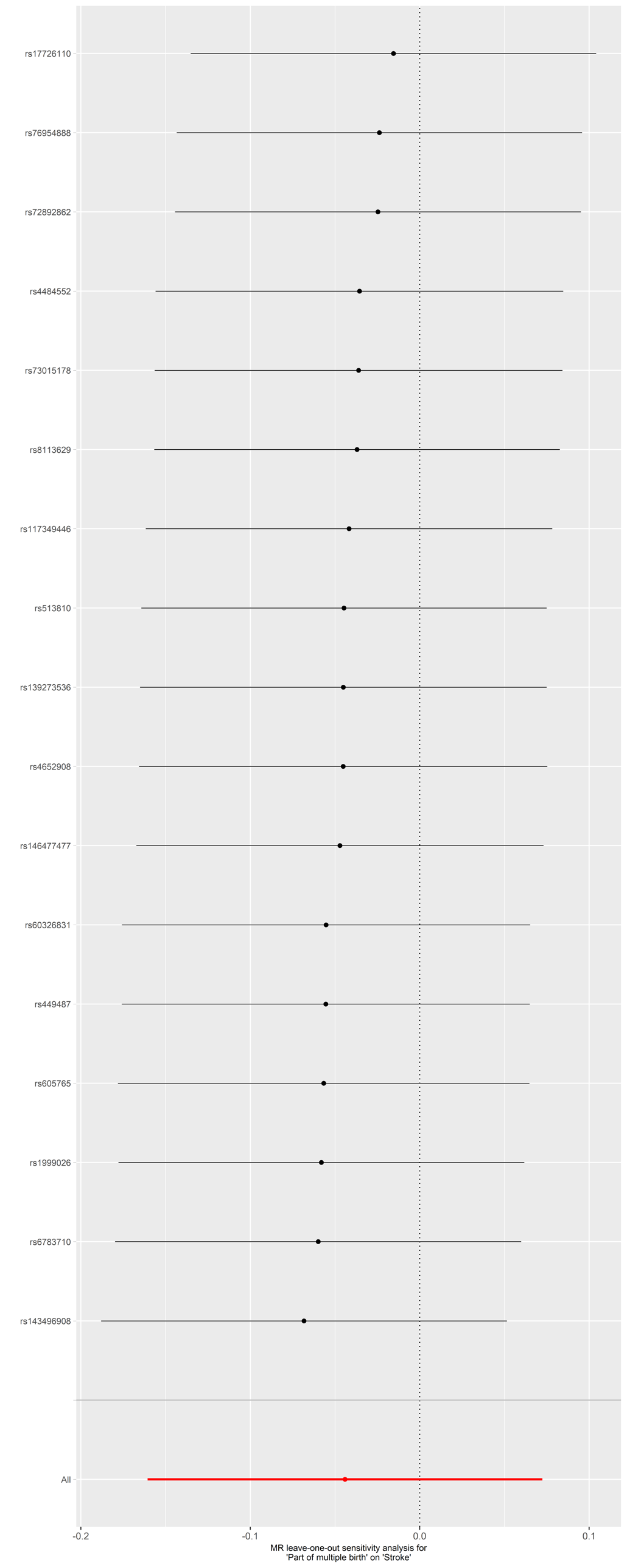


**Subarachnoid hemorrhage – Finngen**


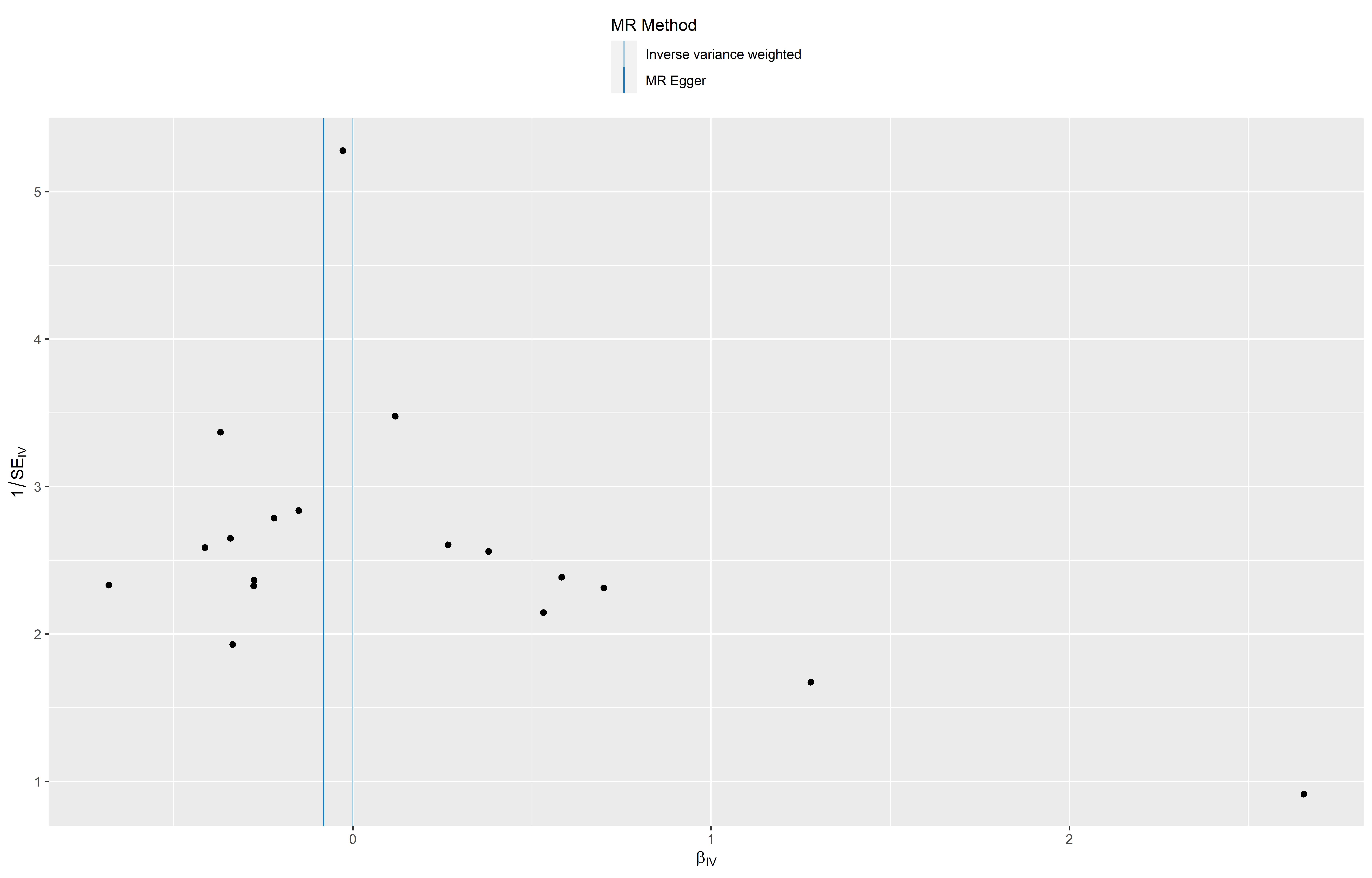

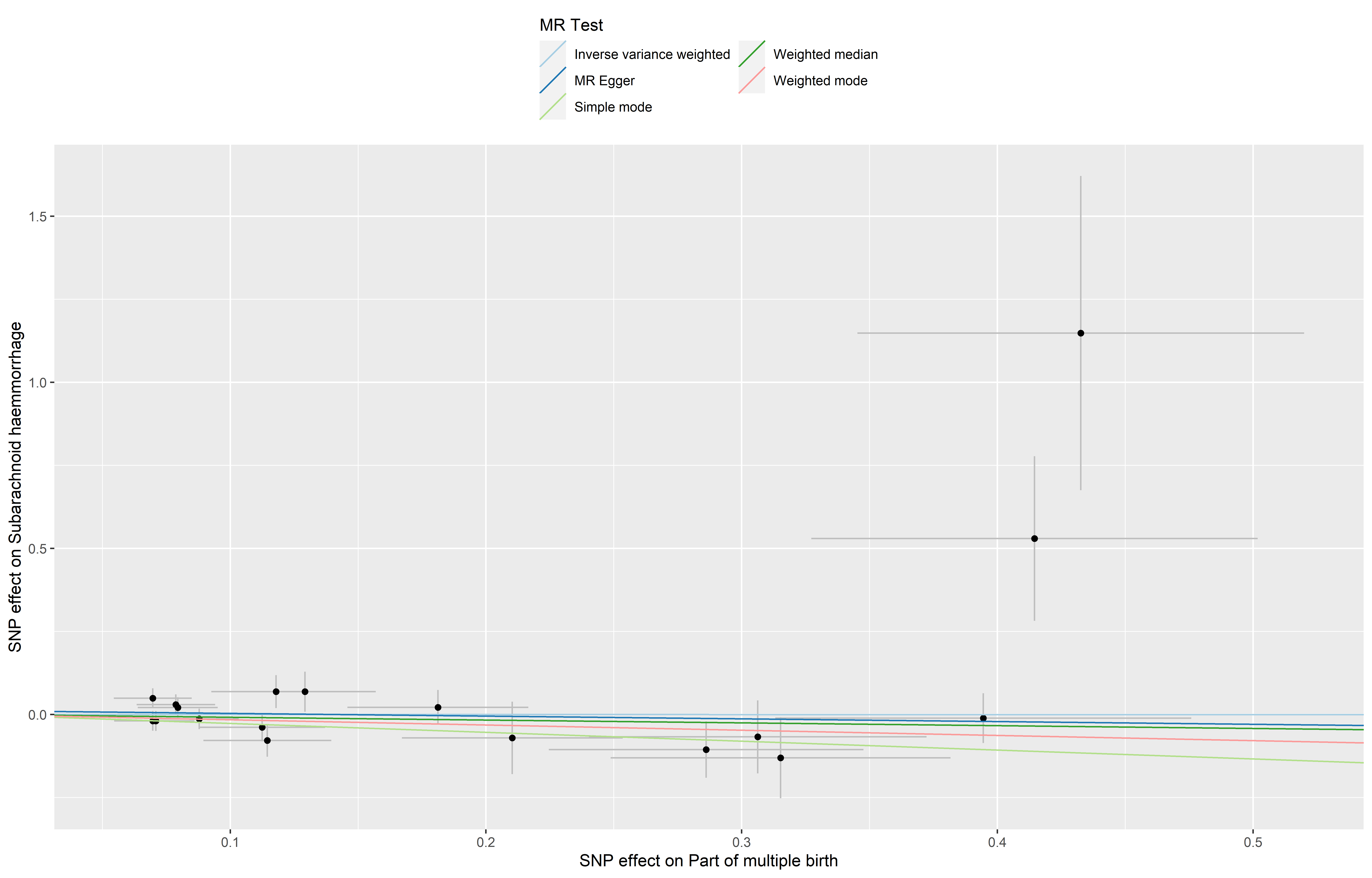


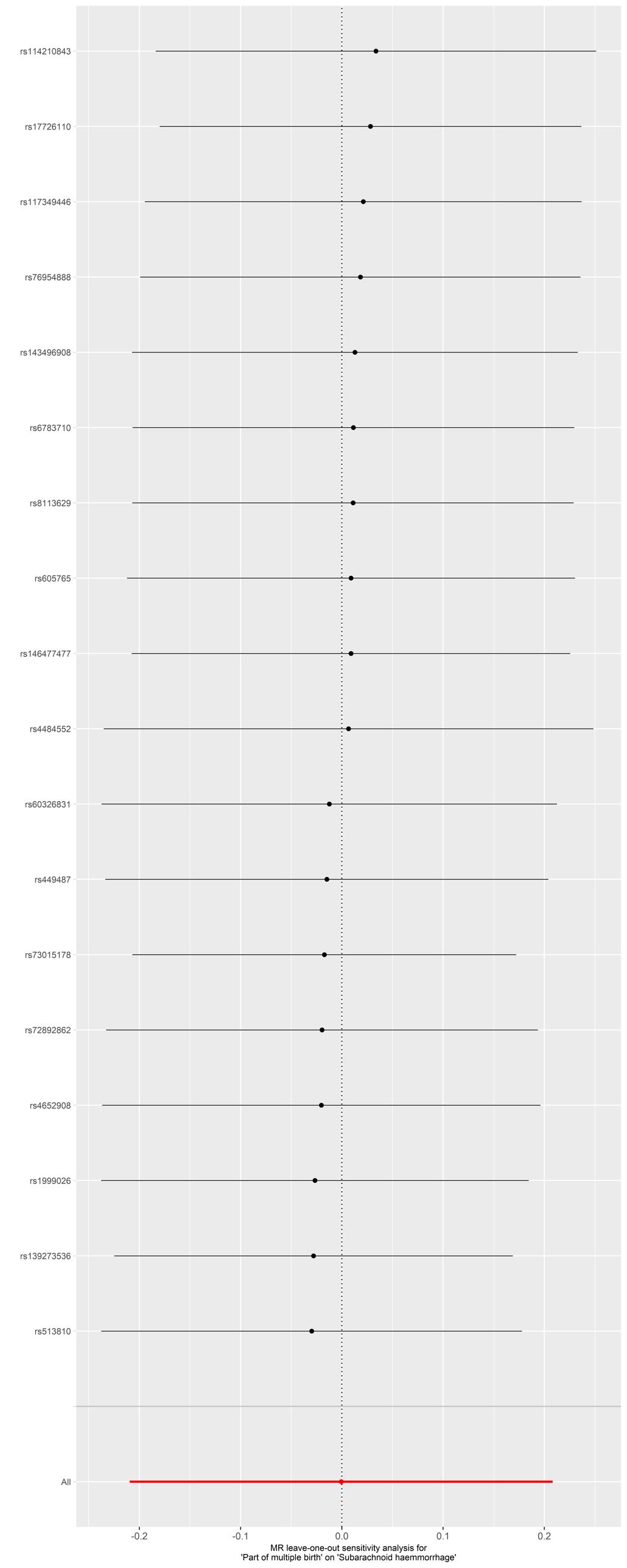


**Subarachnoid hemorrhage – UK Biobank**


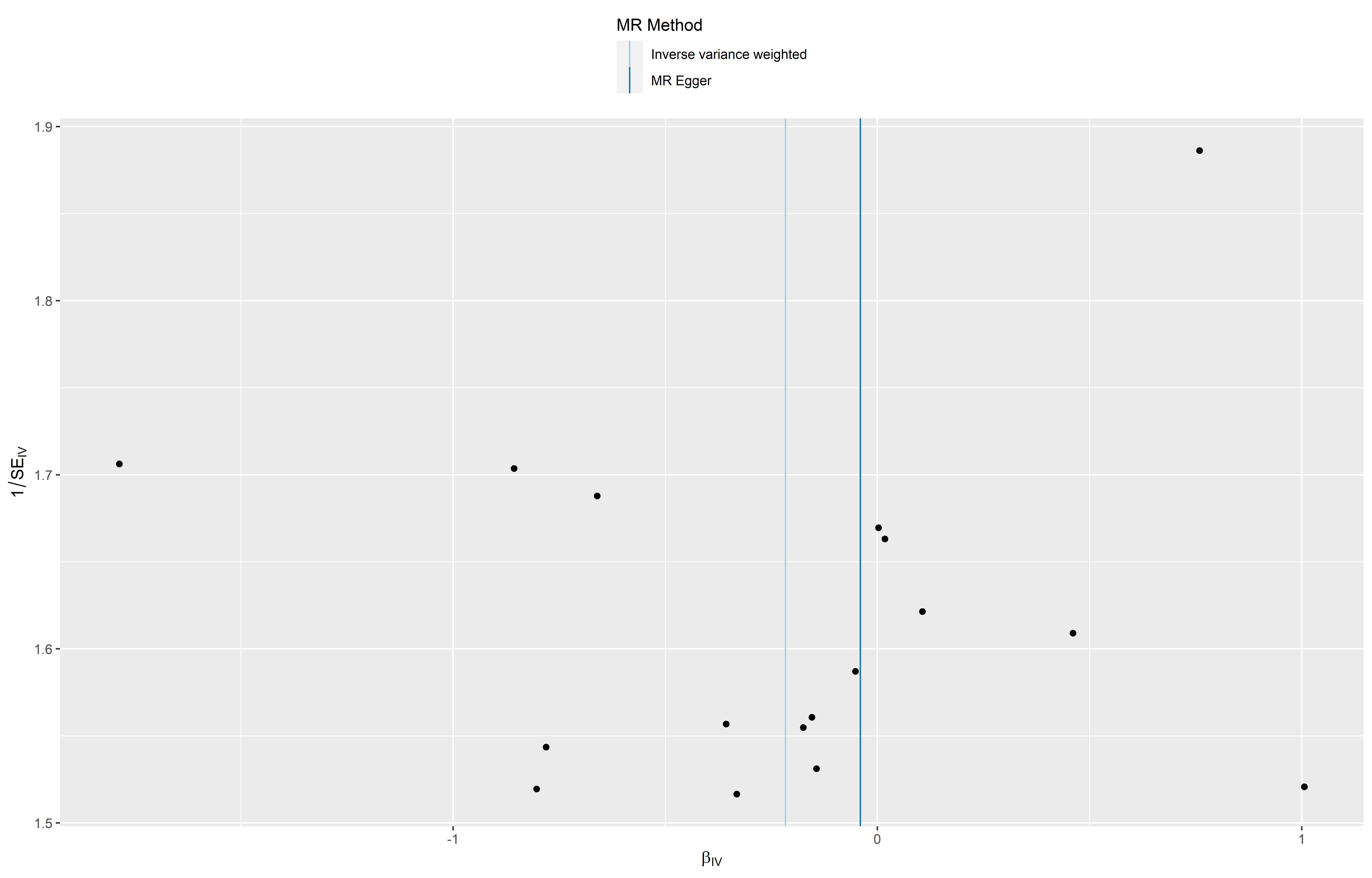

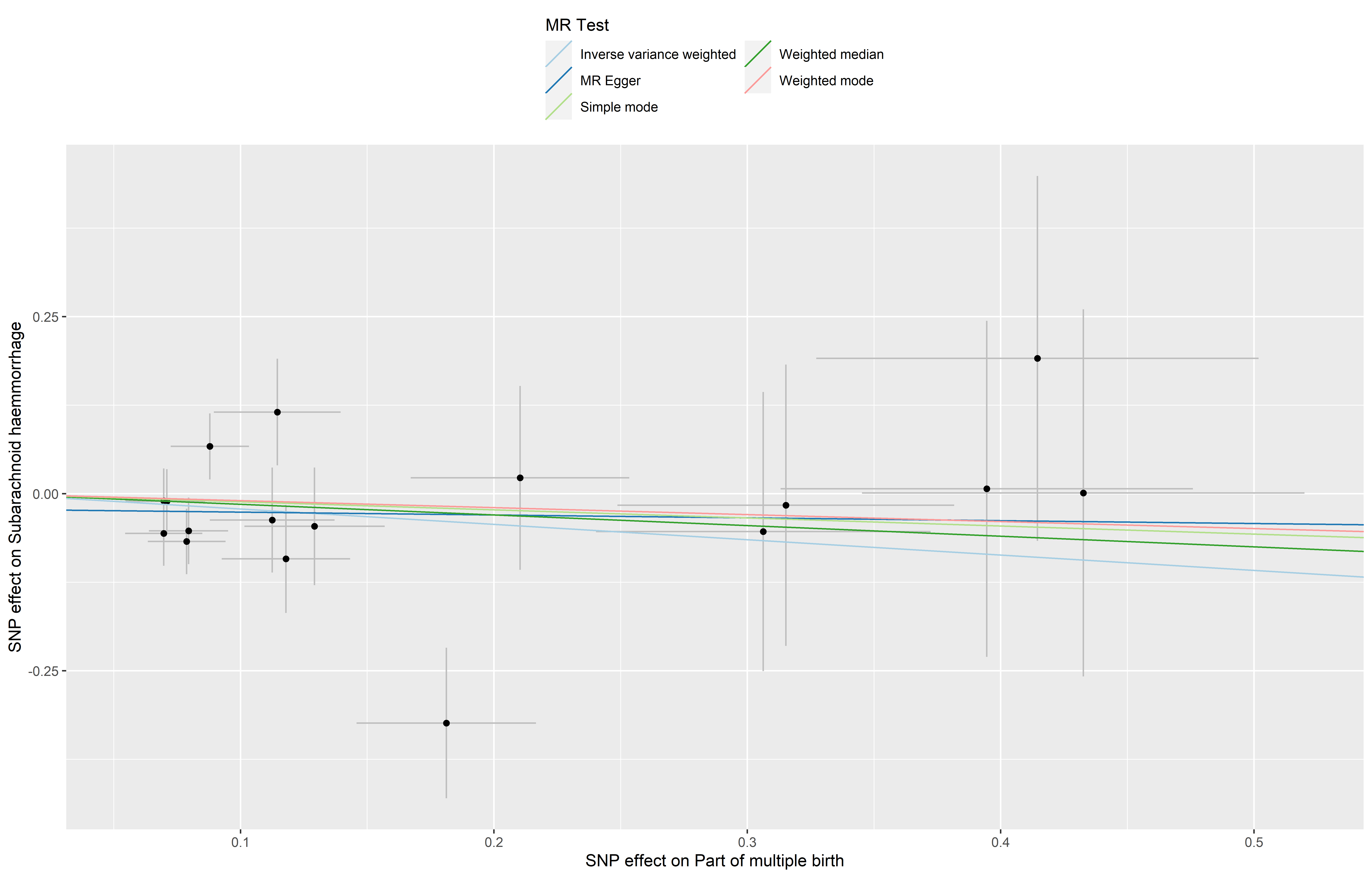


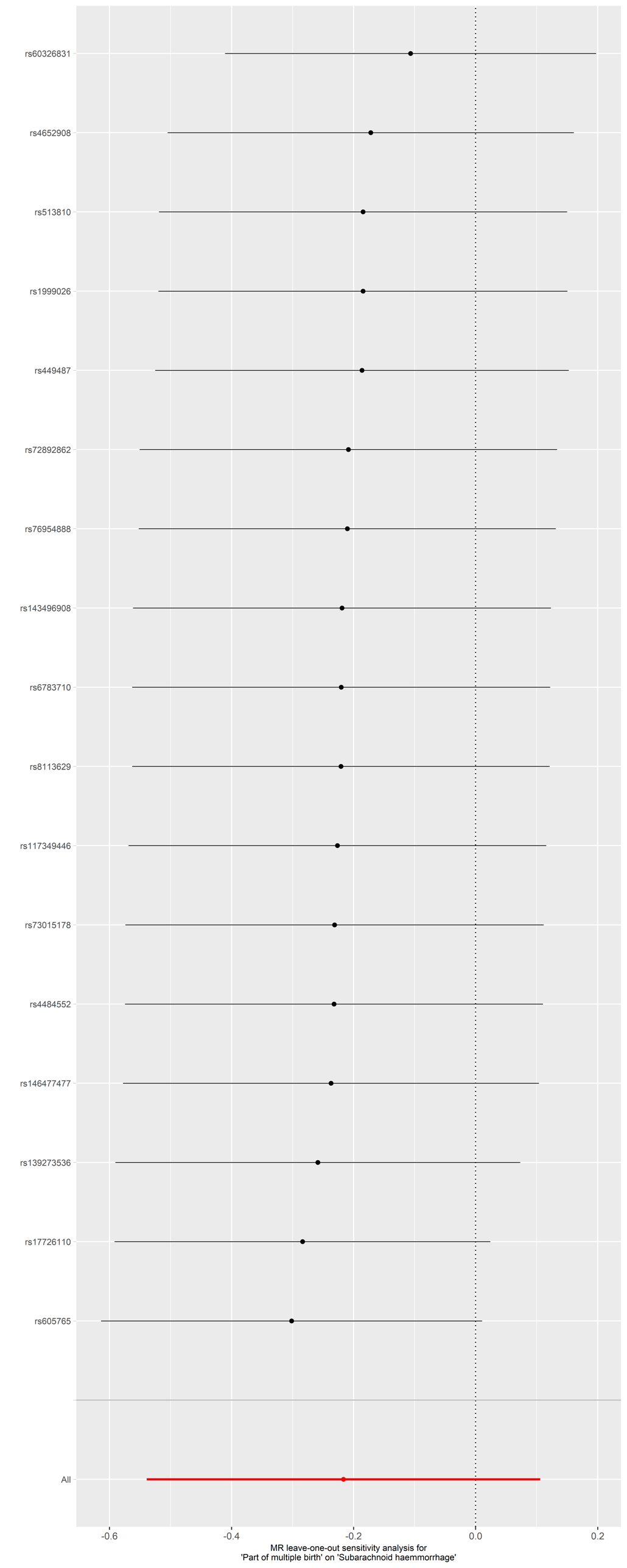


**Transient ischaemic attack – Finngen**


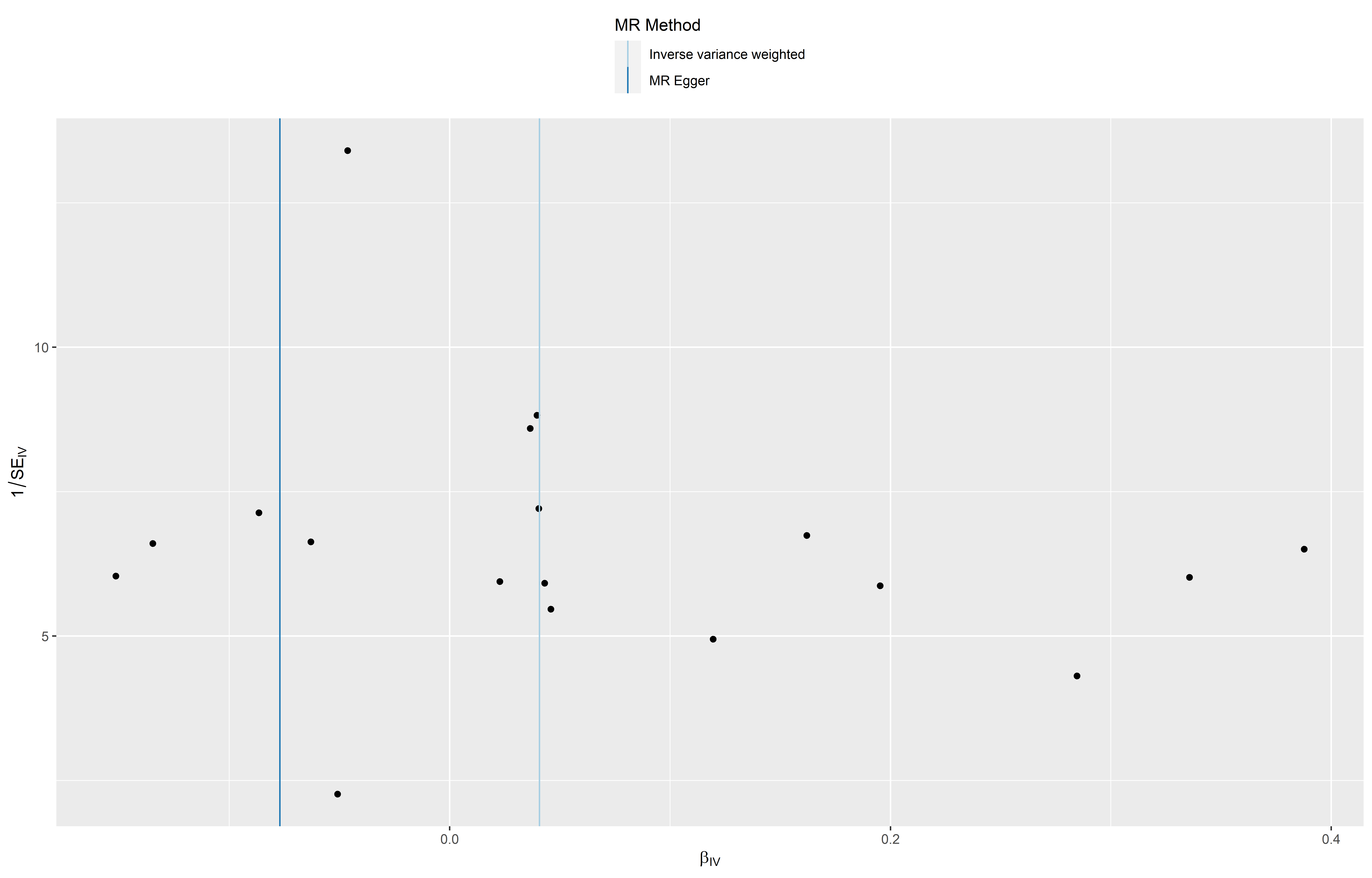

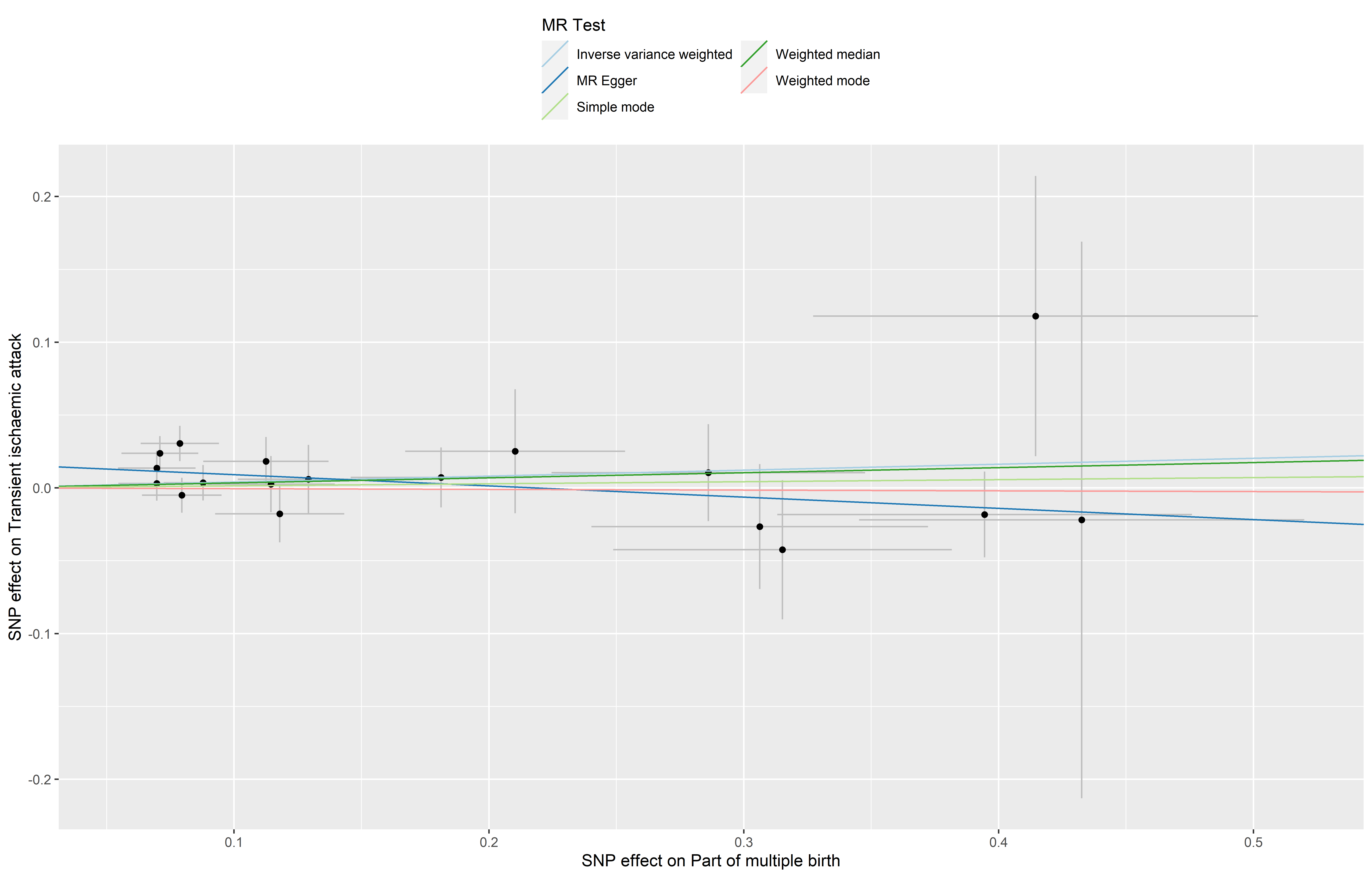


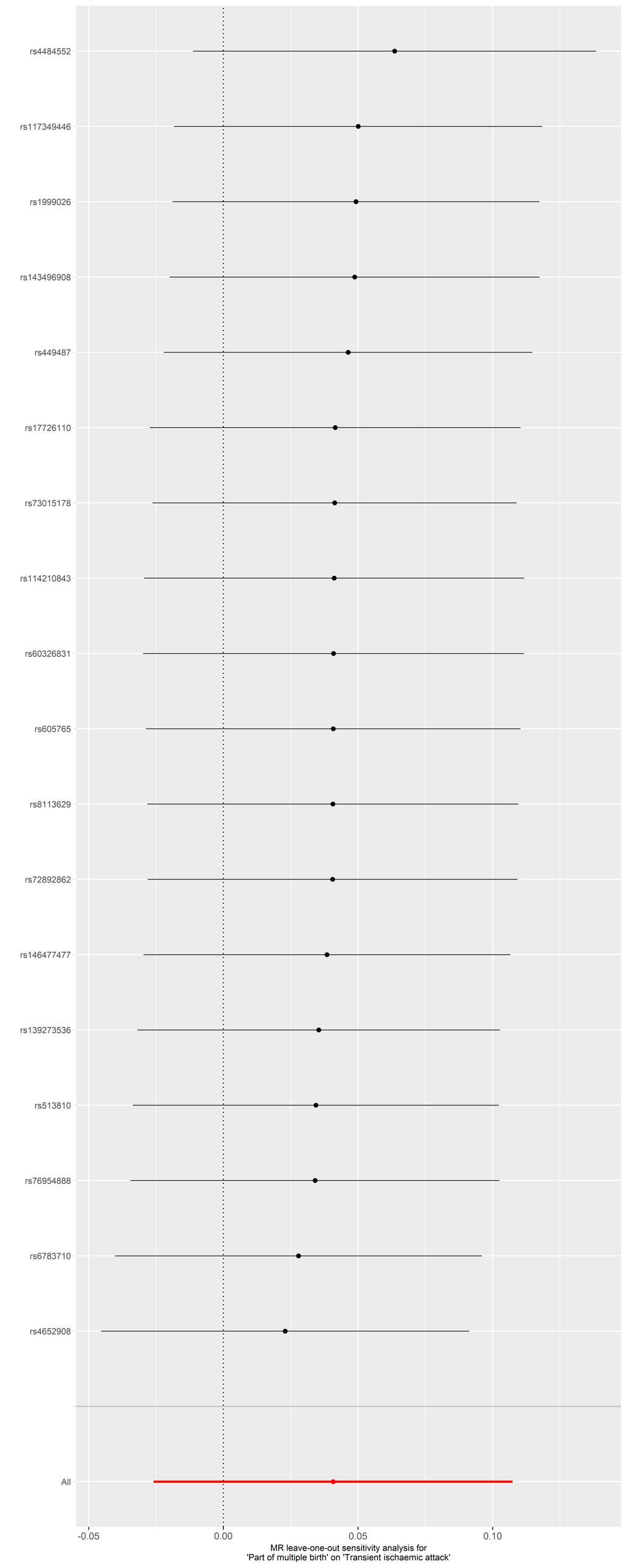


**Transient ischaemic attack – UK Biobank**


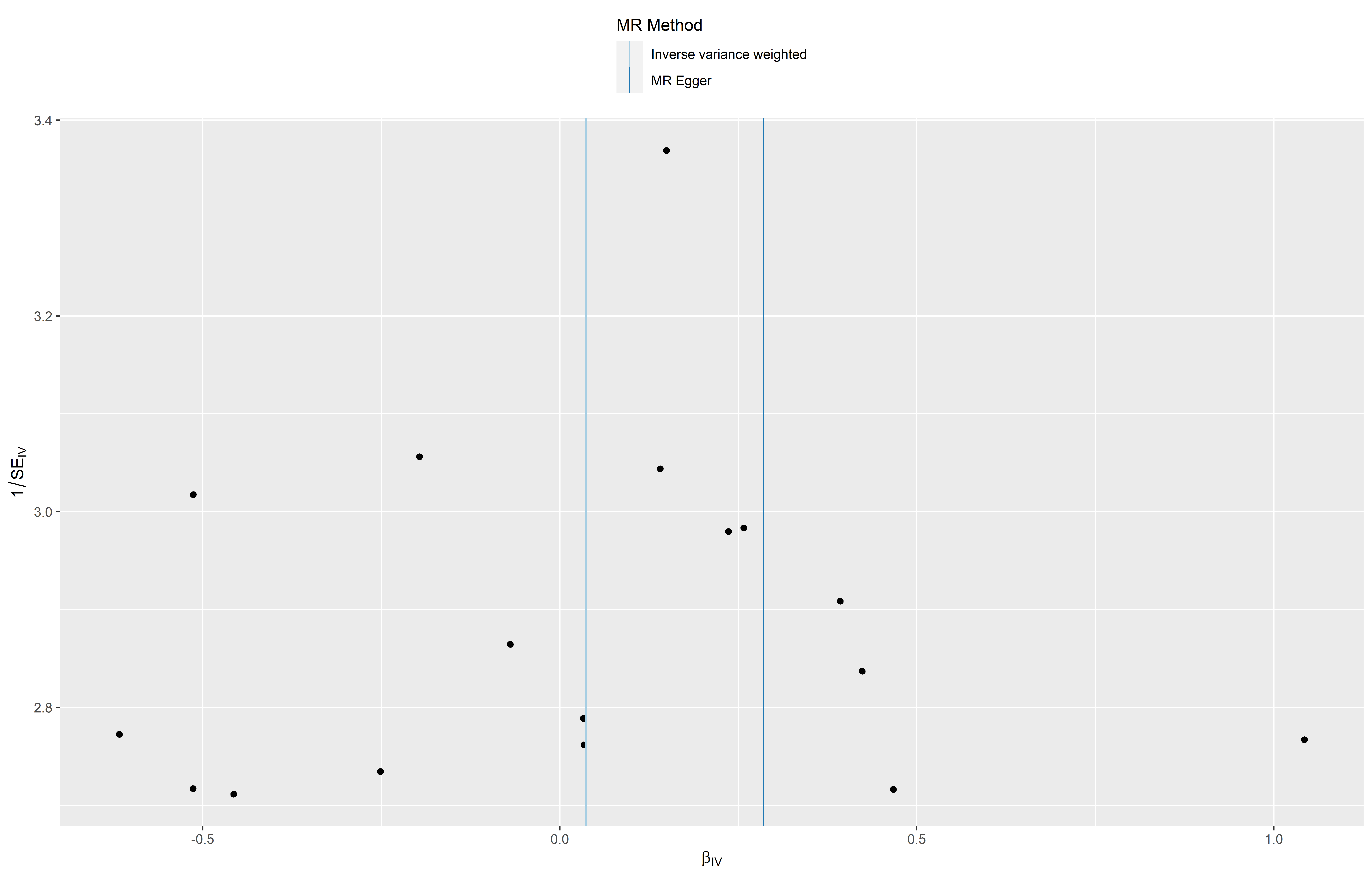

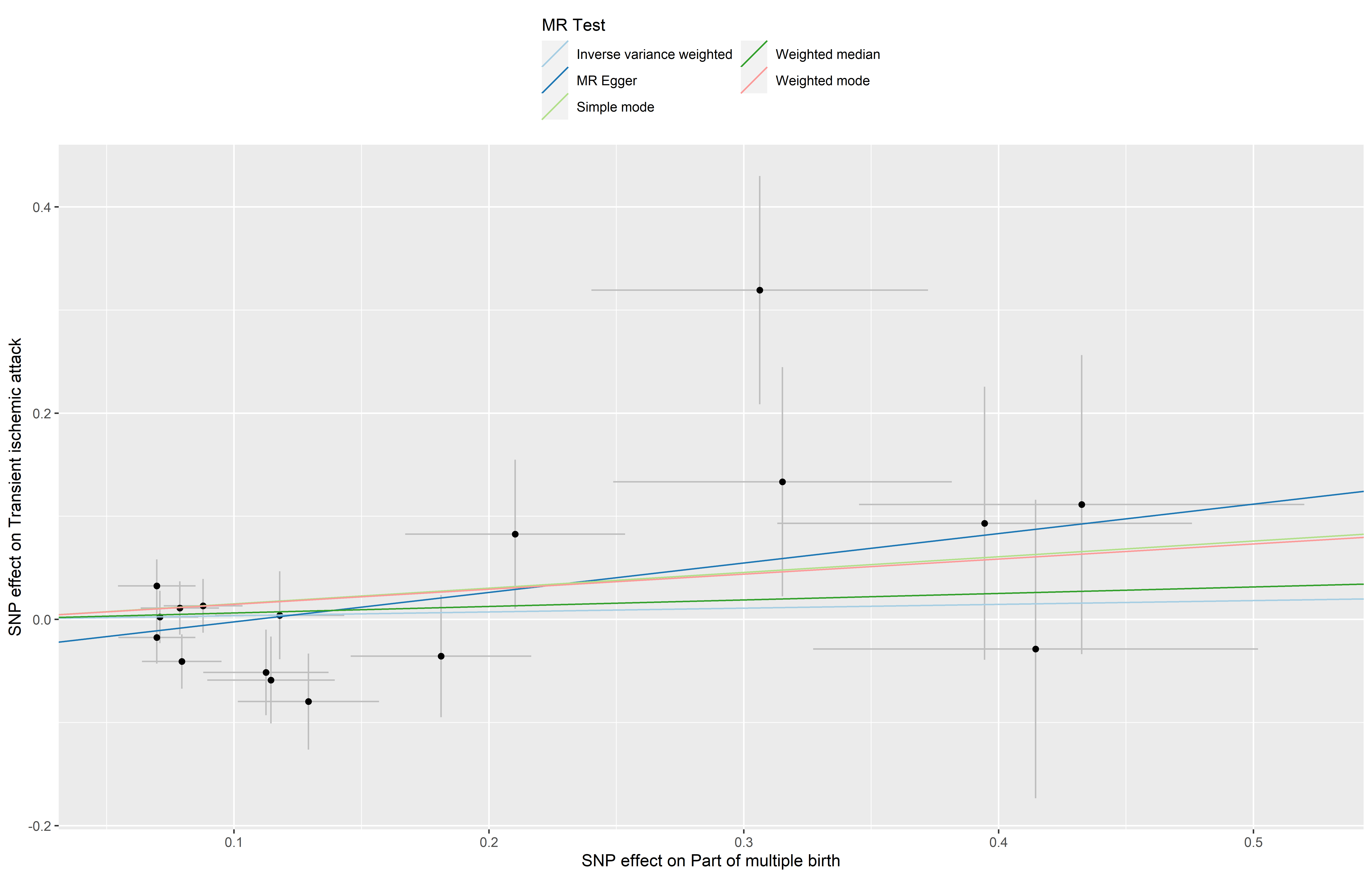


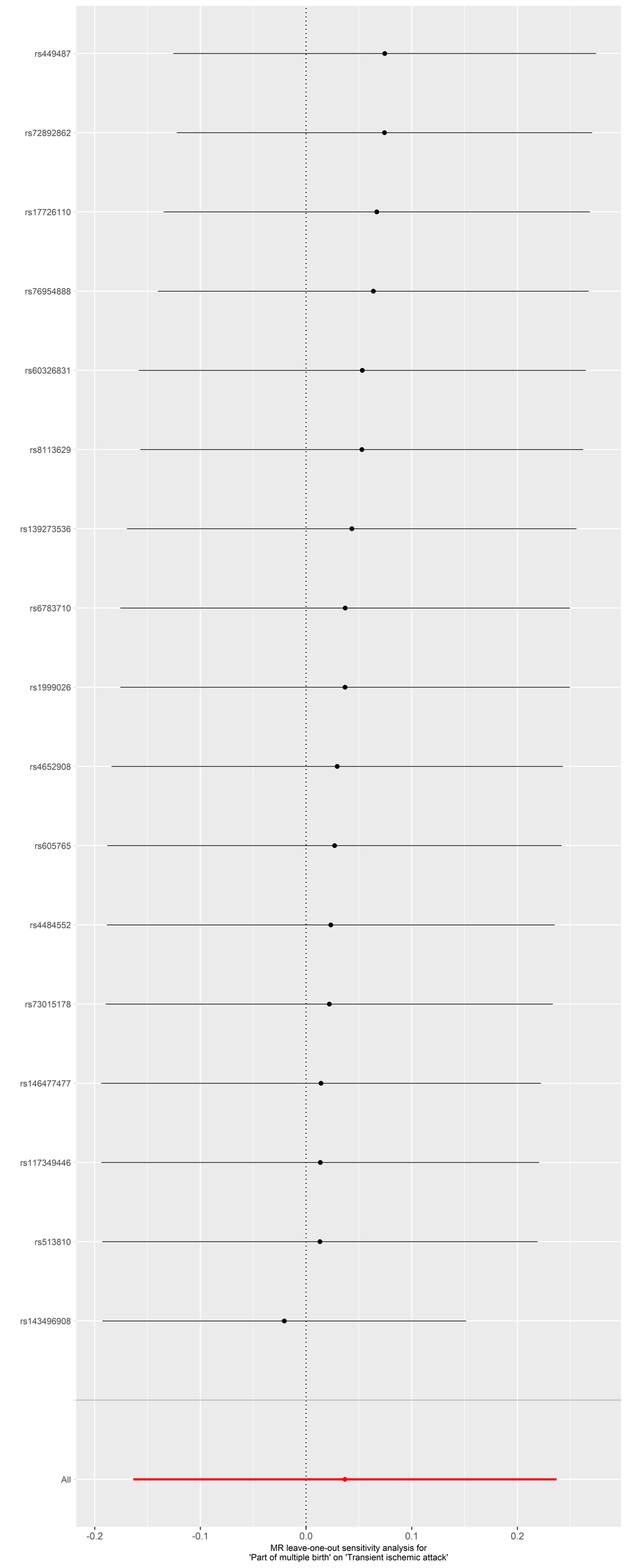


**Epilepsy – Finngen**


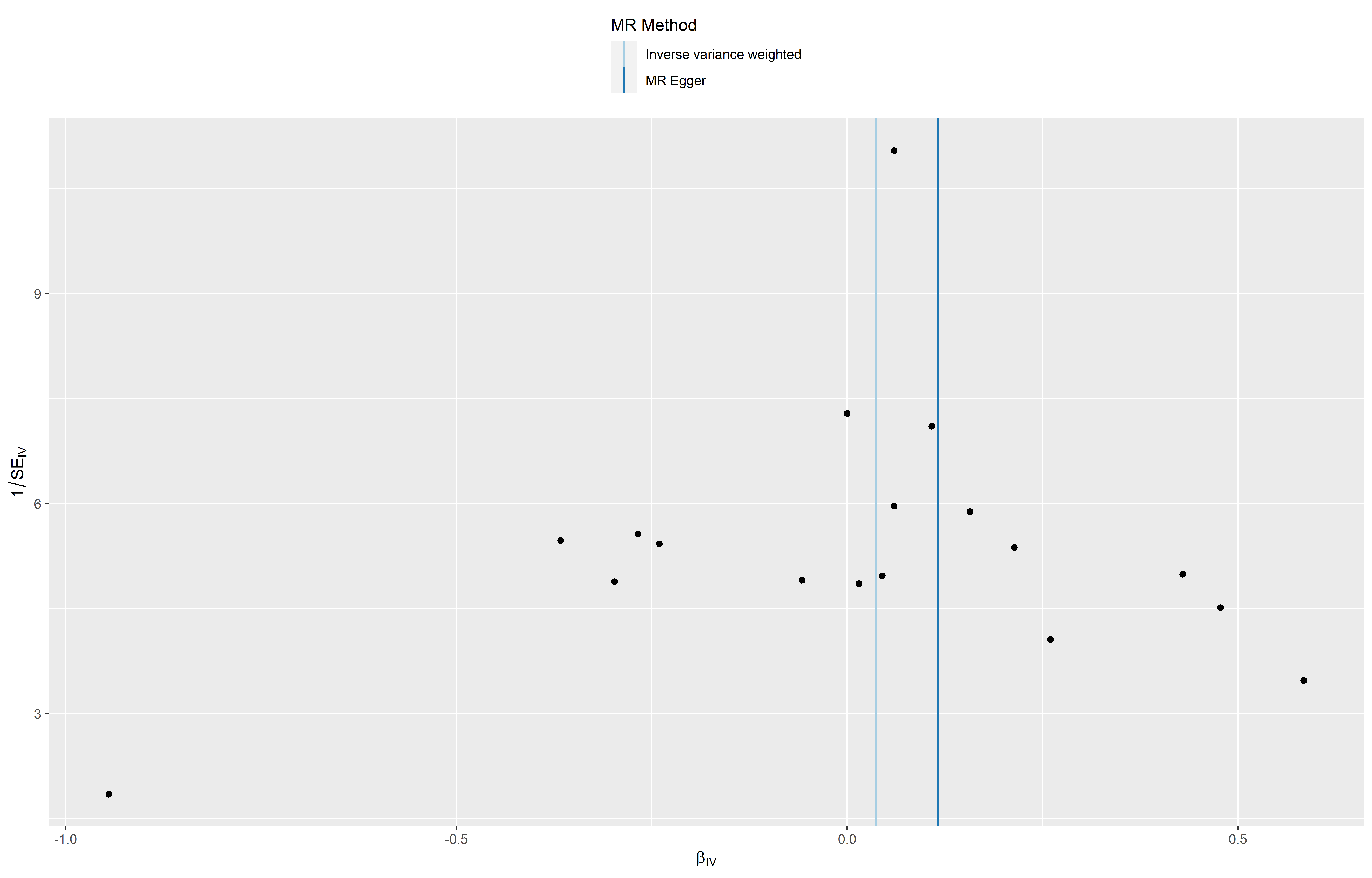

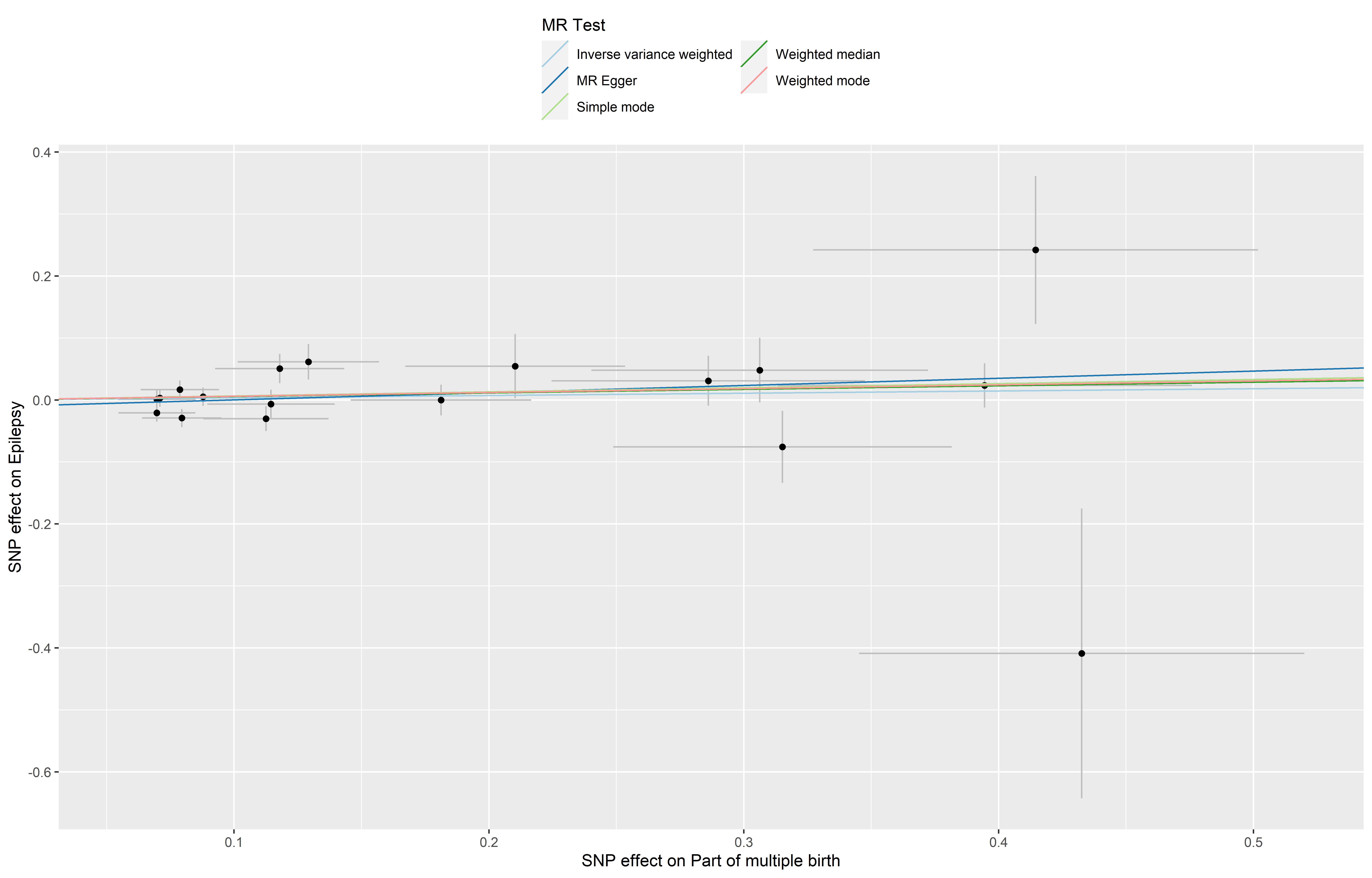


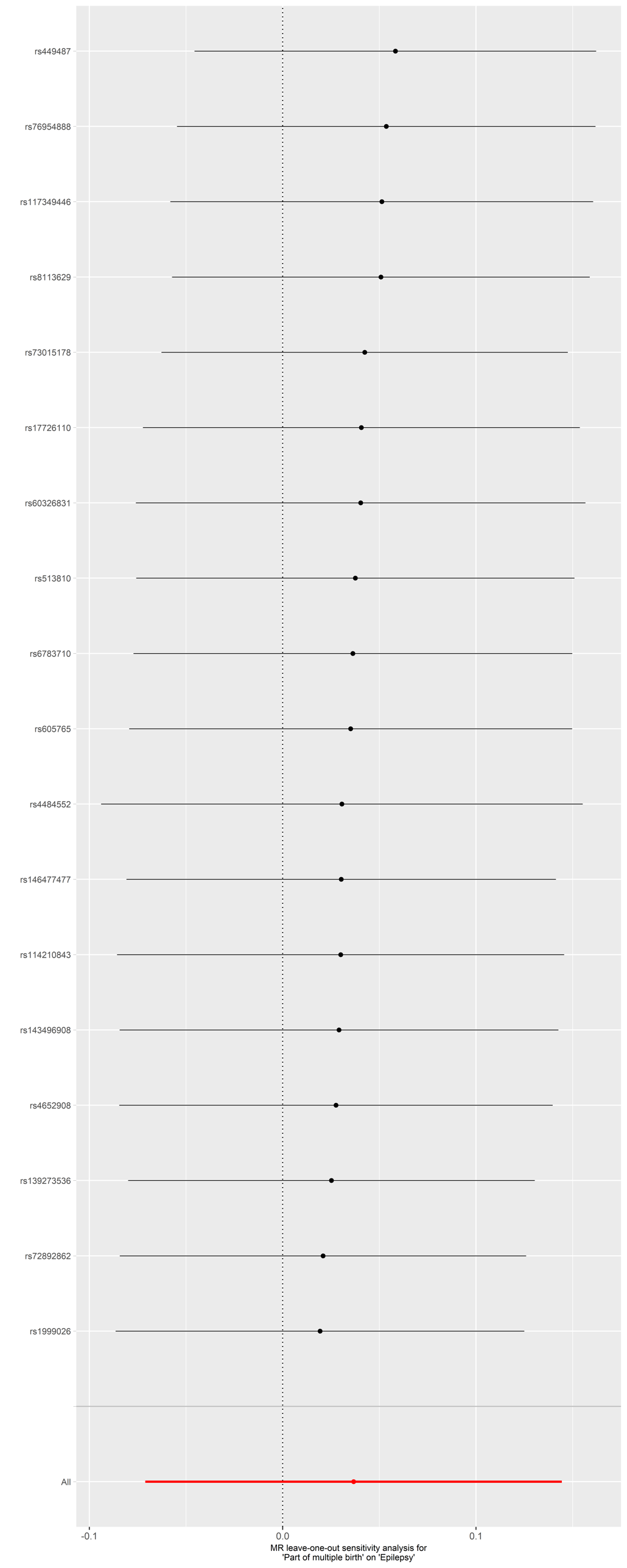


**Epilepsy – UK Biobank**


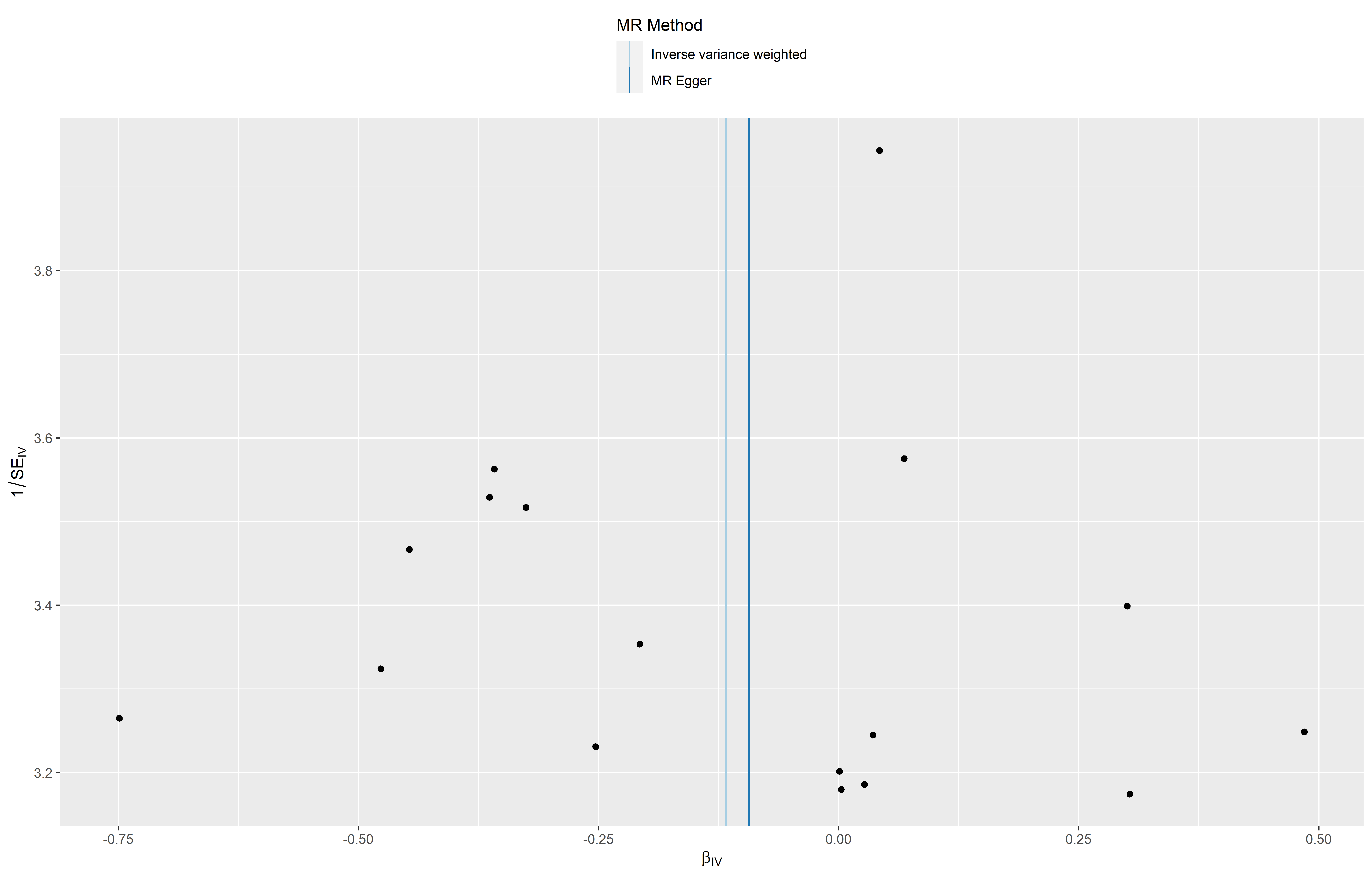

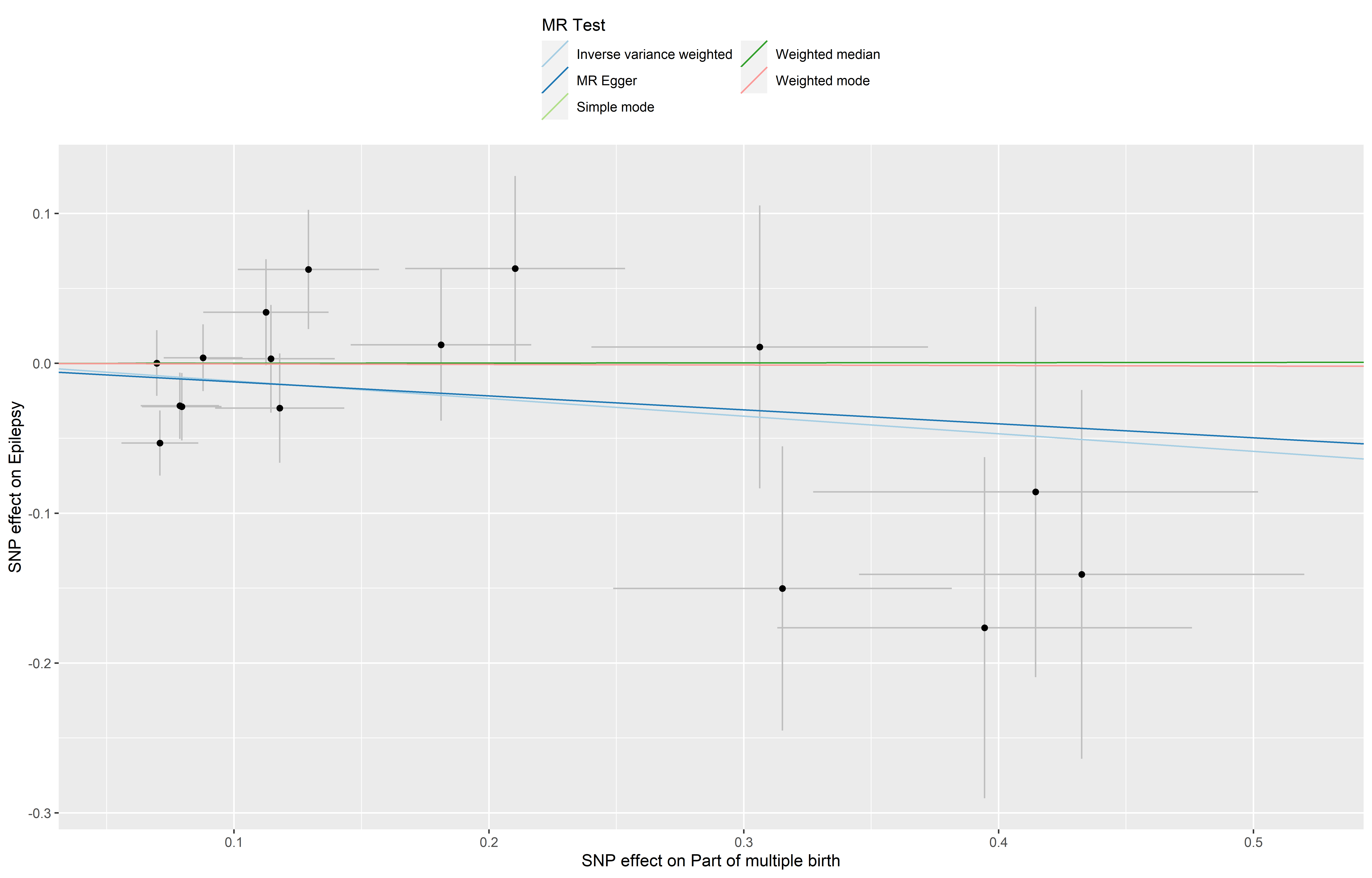


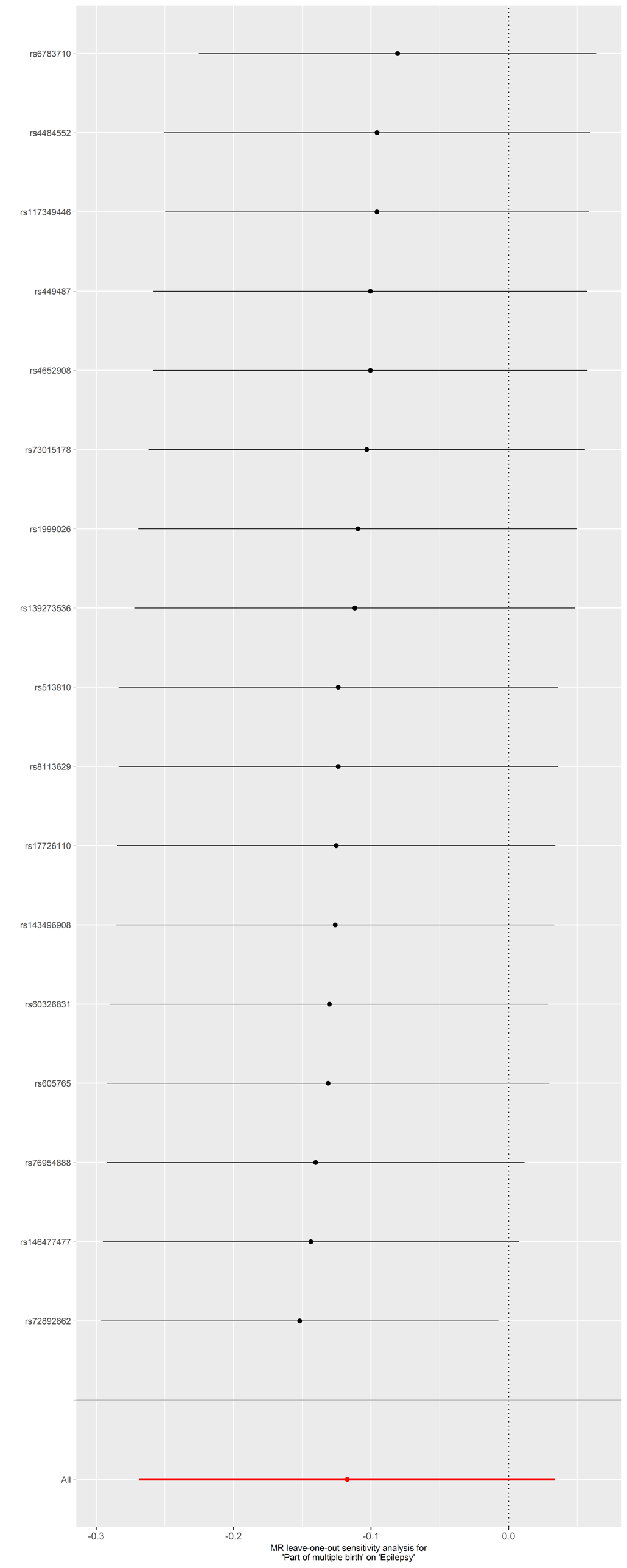


**Migraine – Finngen**


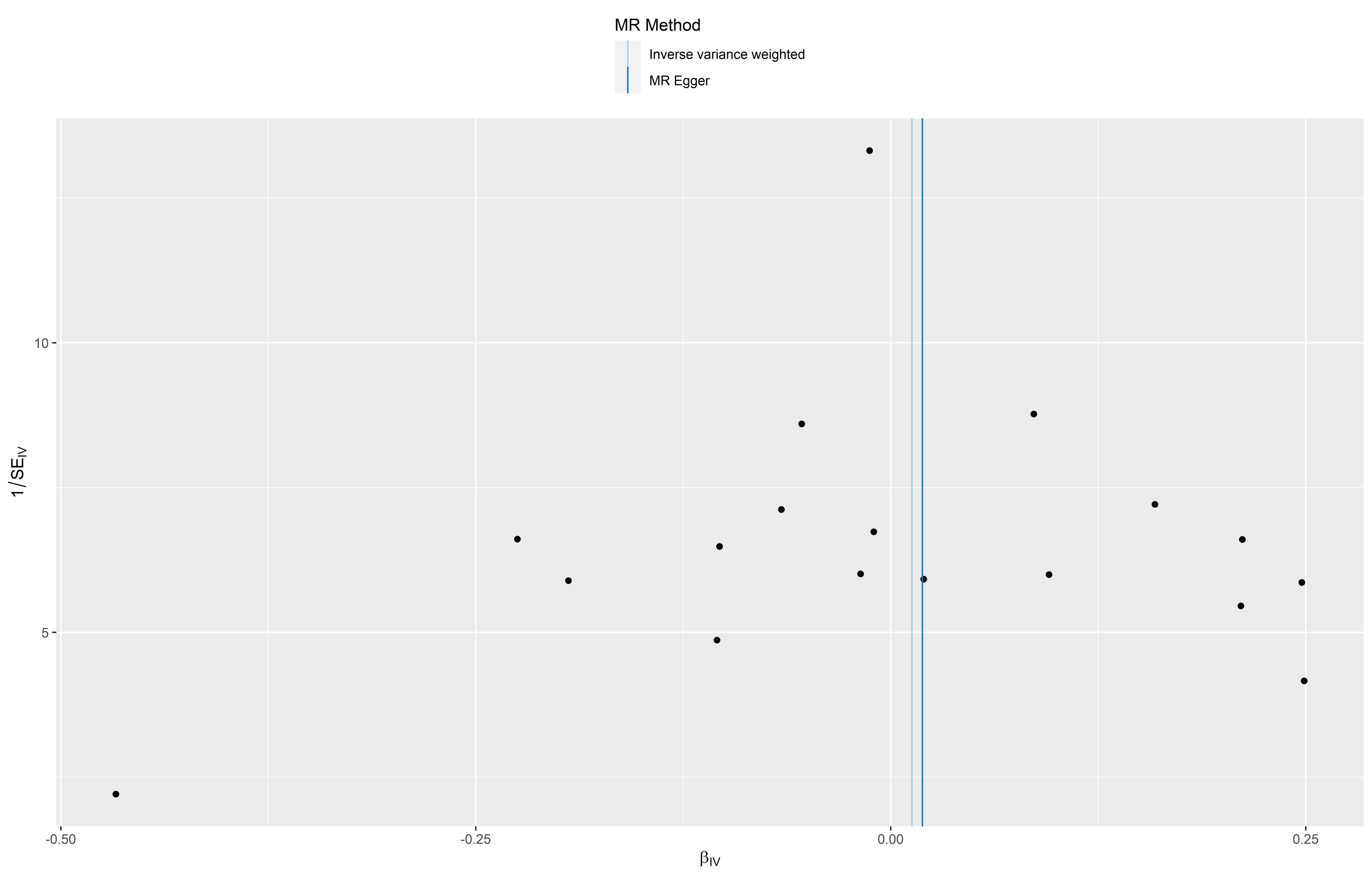

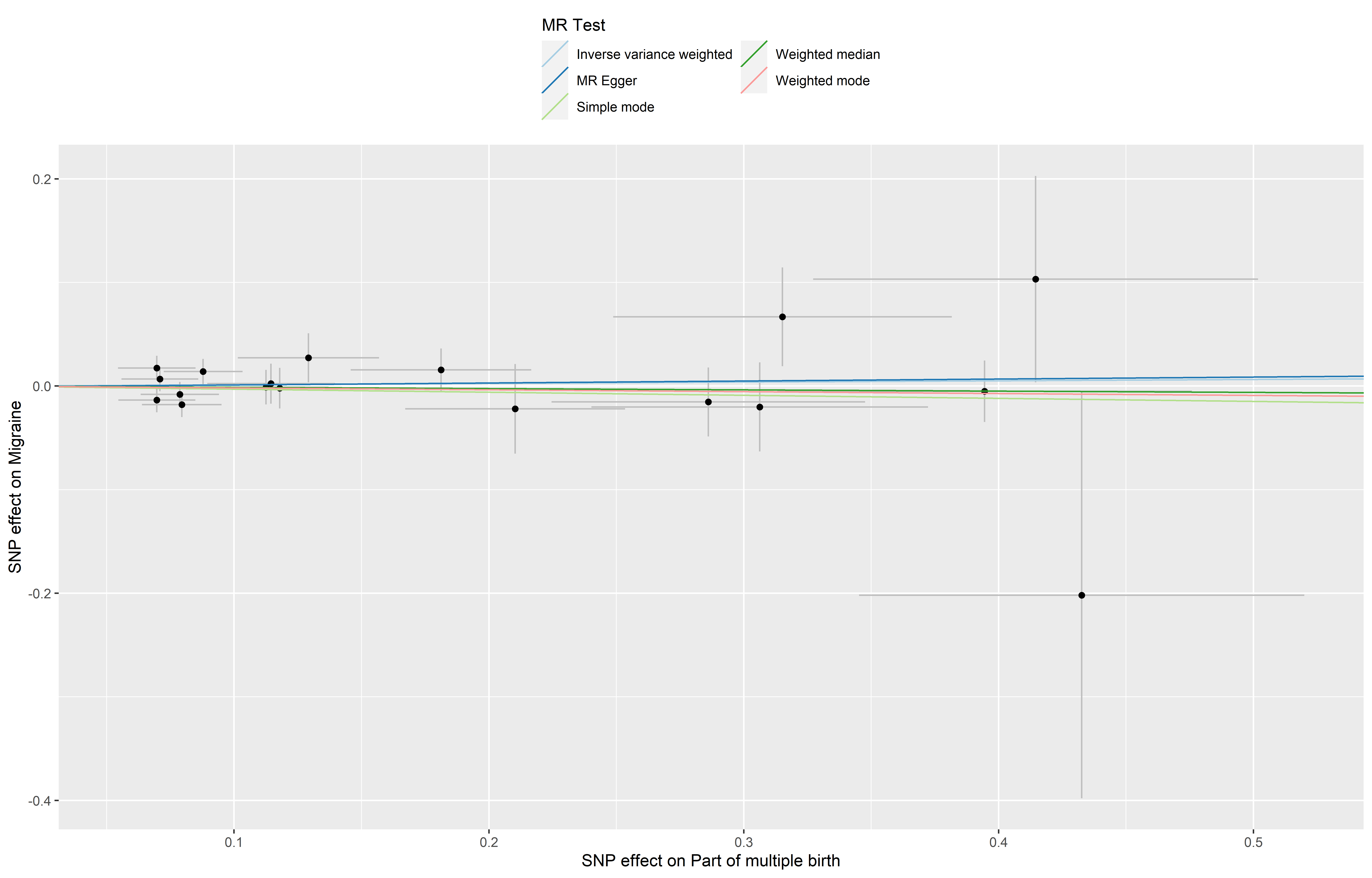


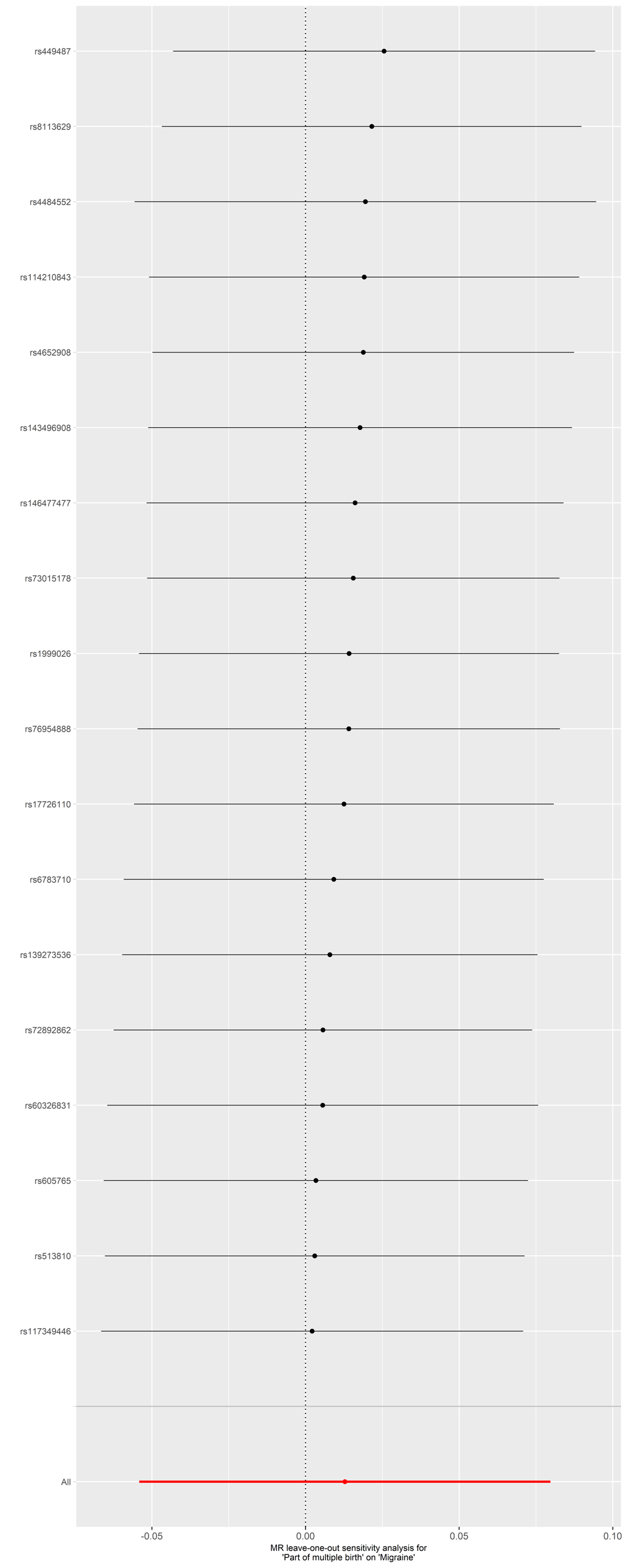


**Migraine – UK Biobank**


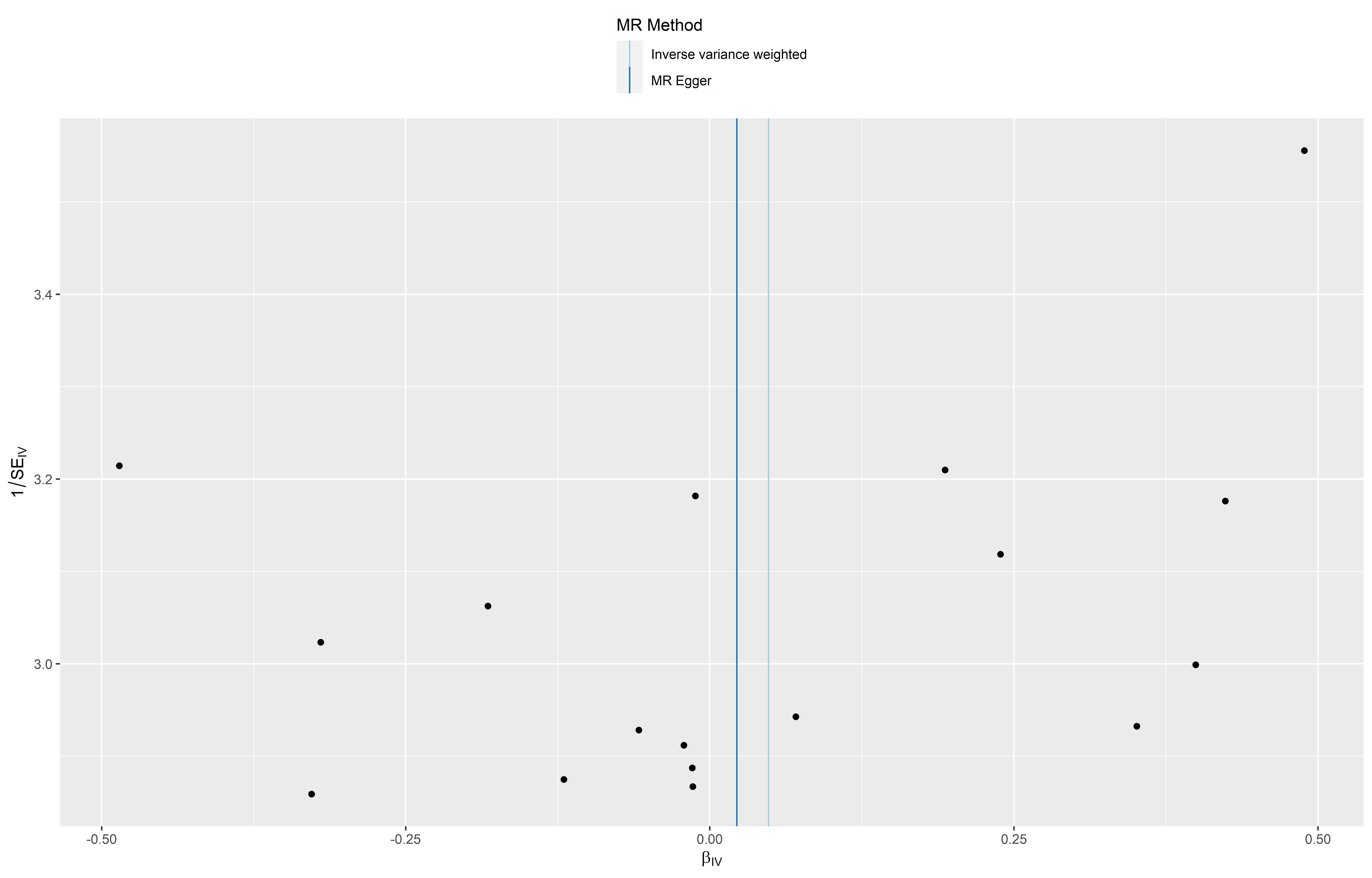

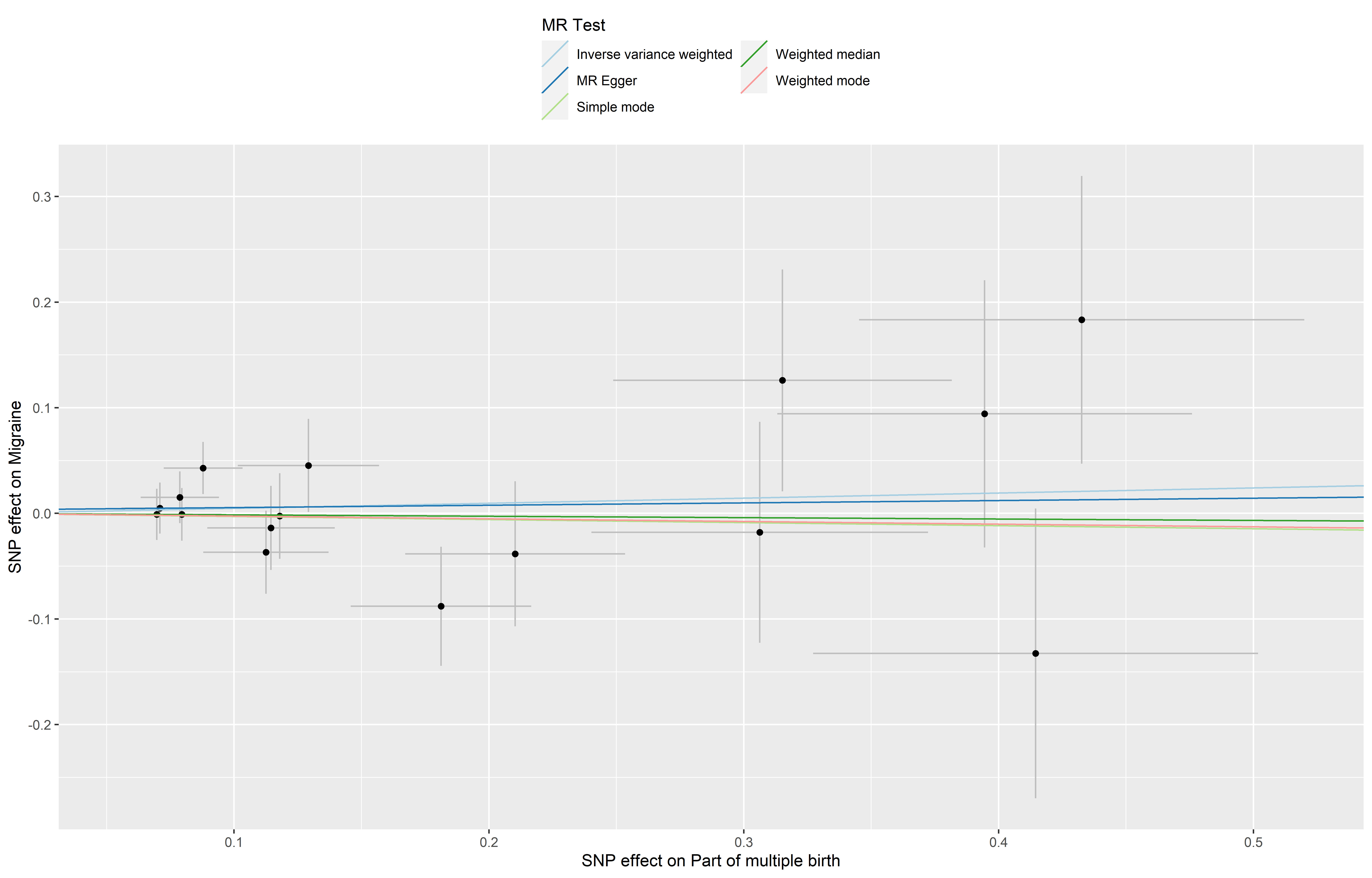


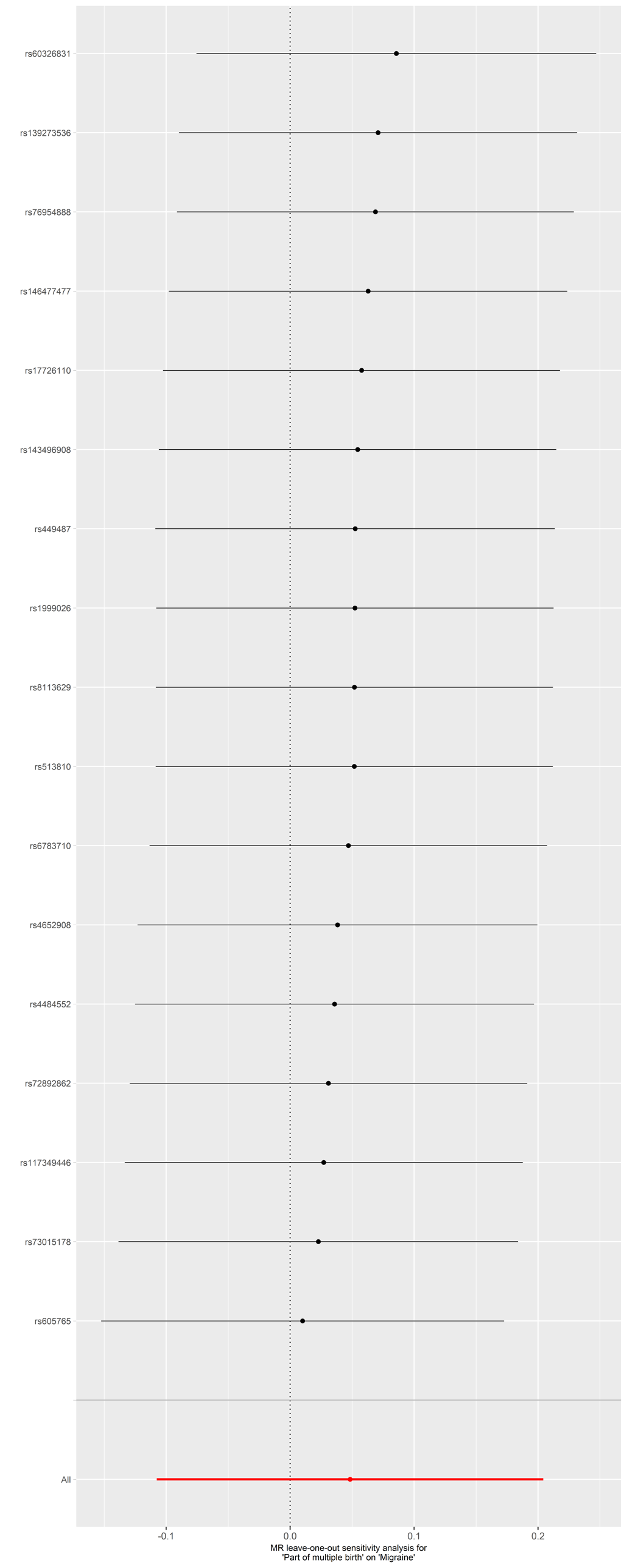


**Alzheimer's disease – Finngen**


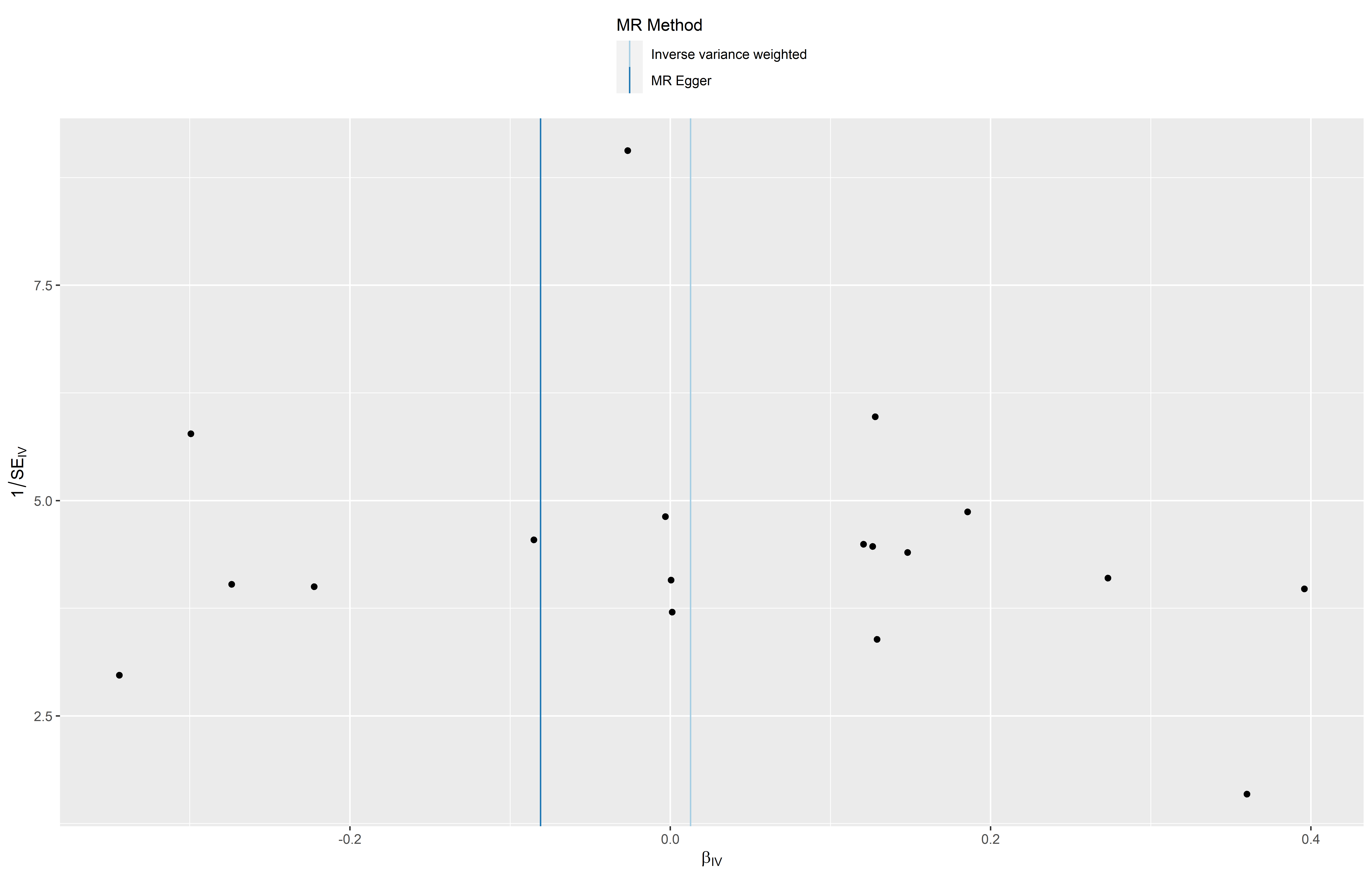

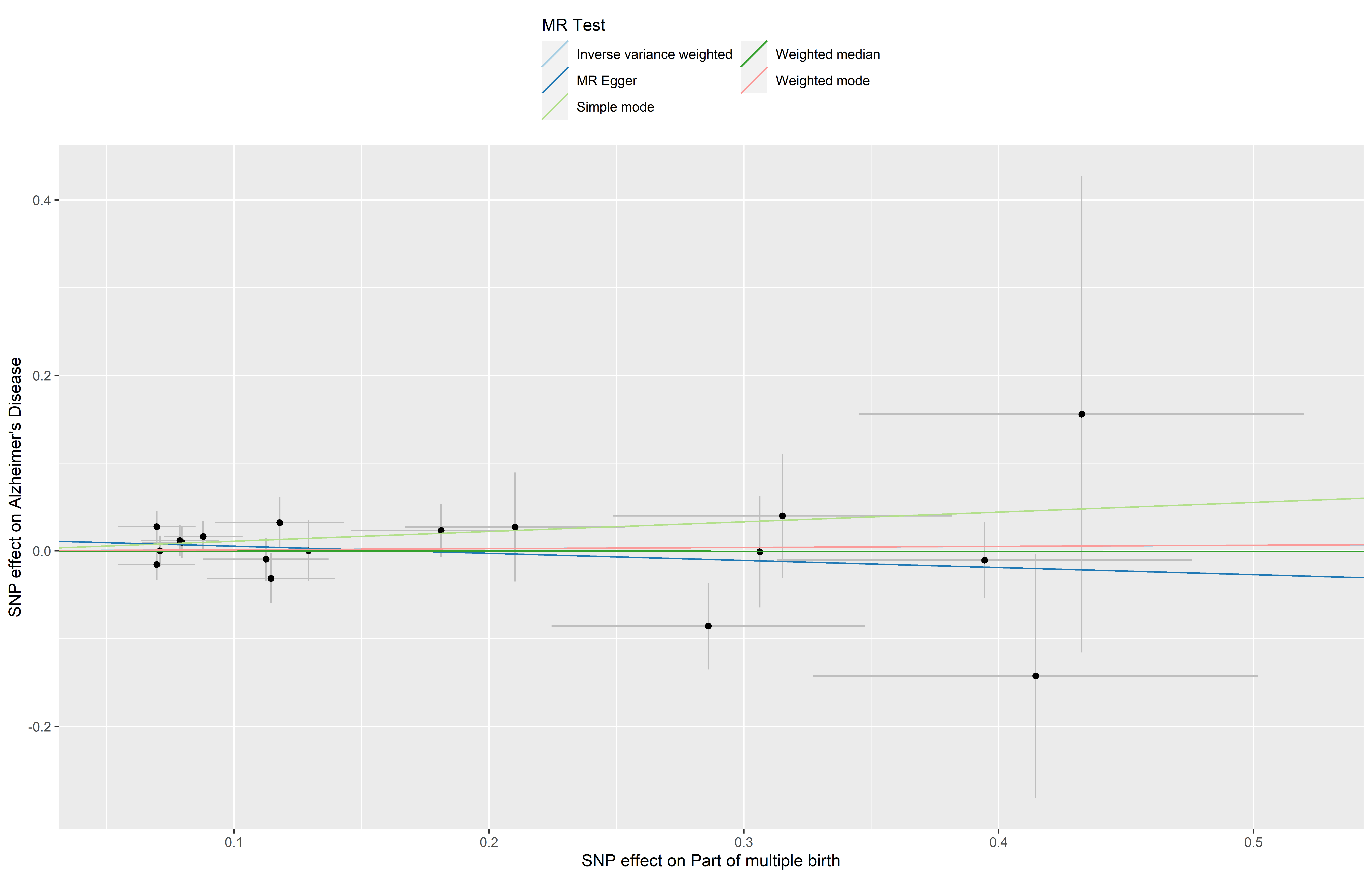

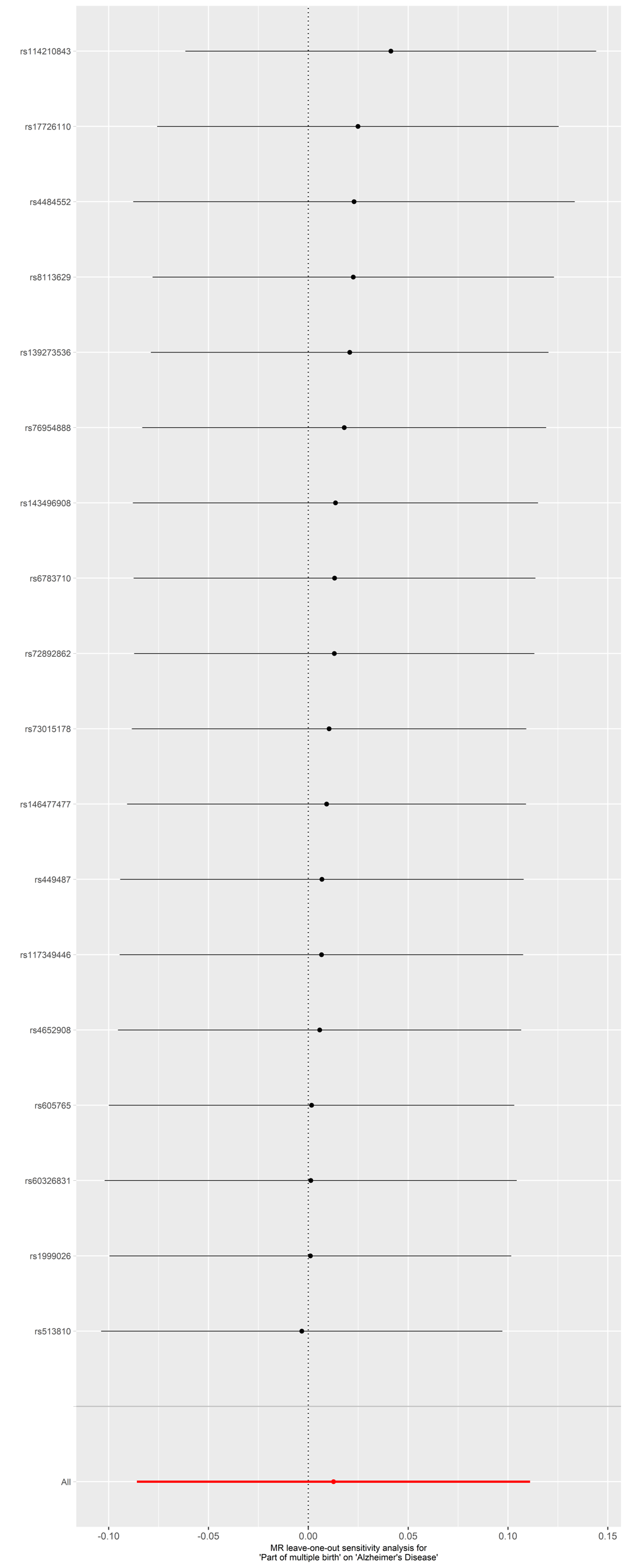


**Alzheimer's disease – UK Biobank**


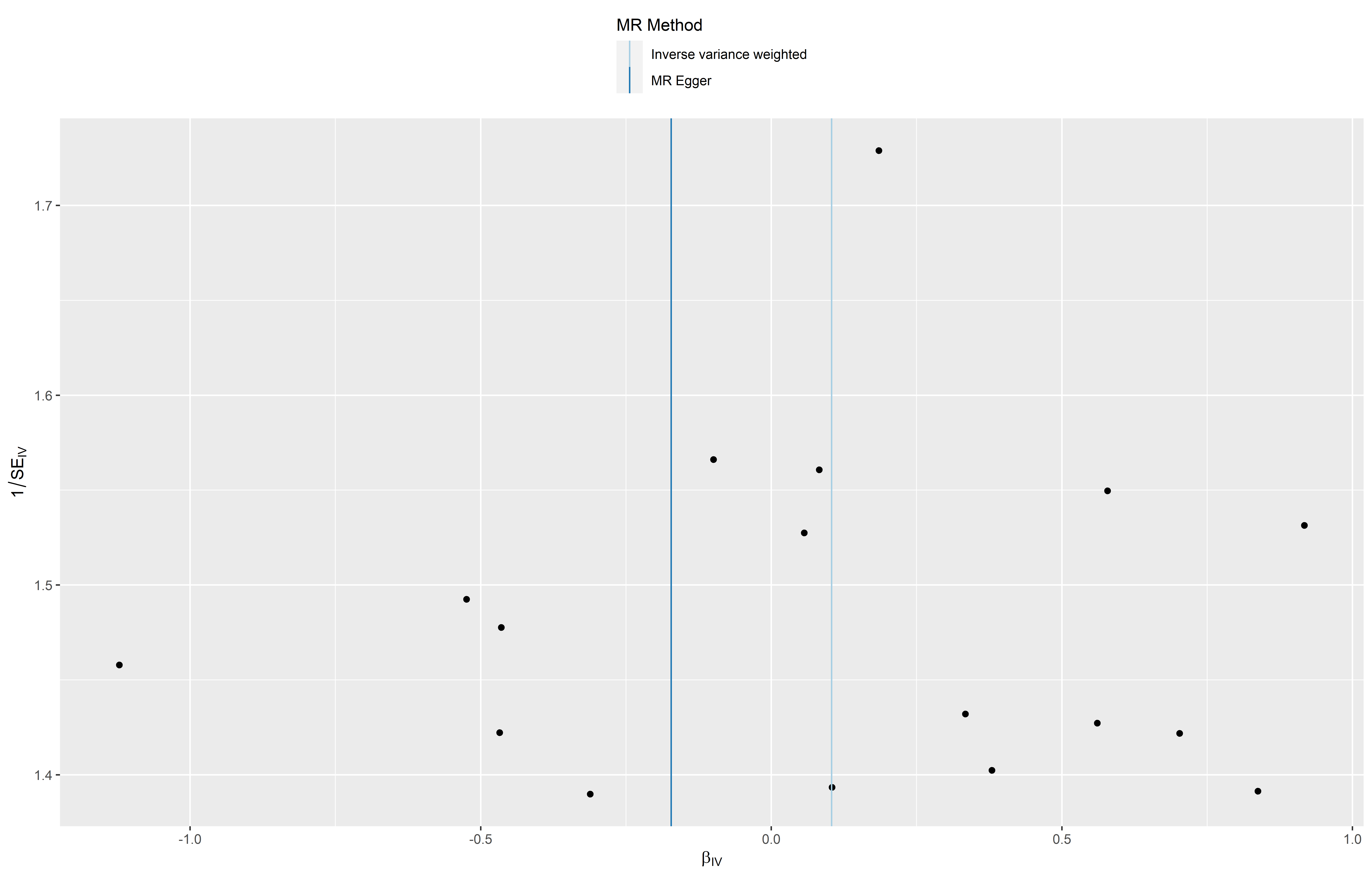

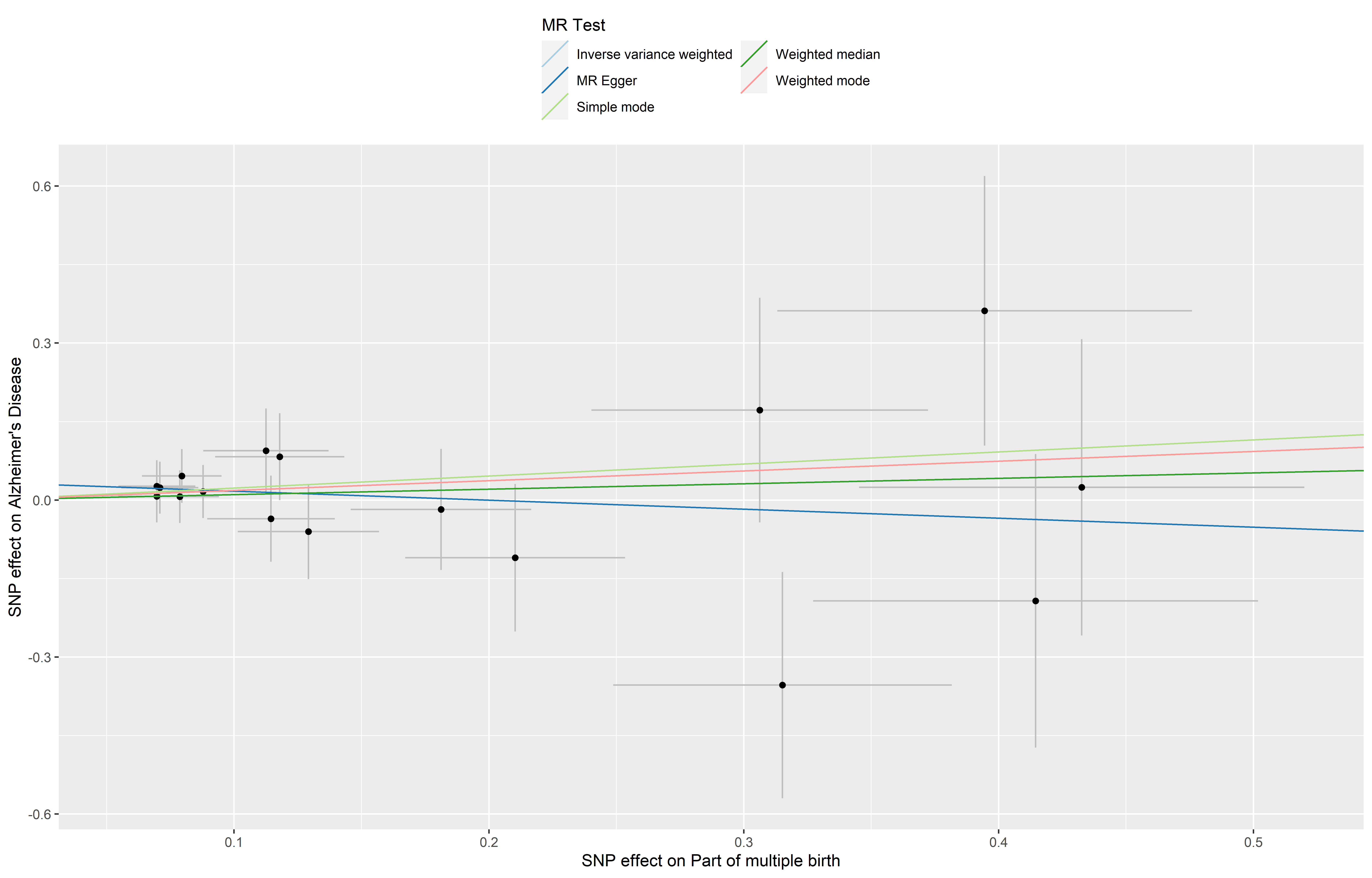


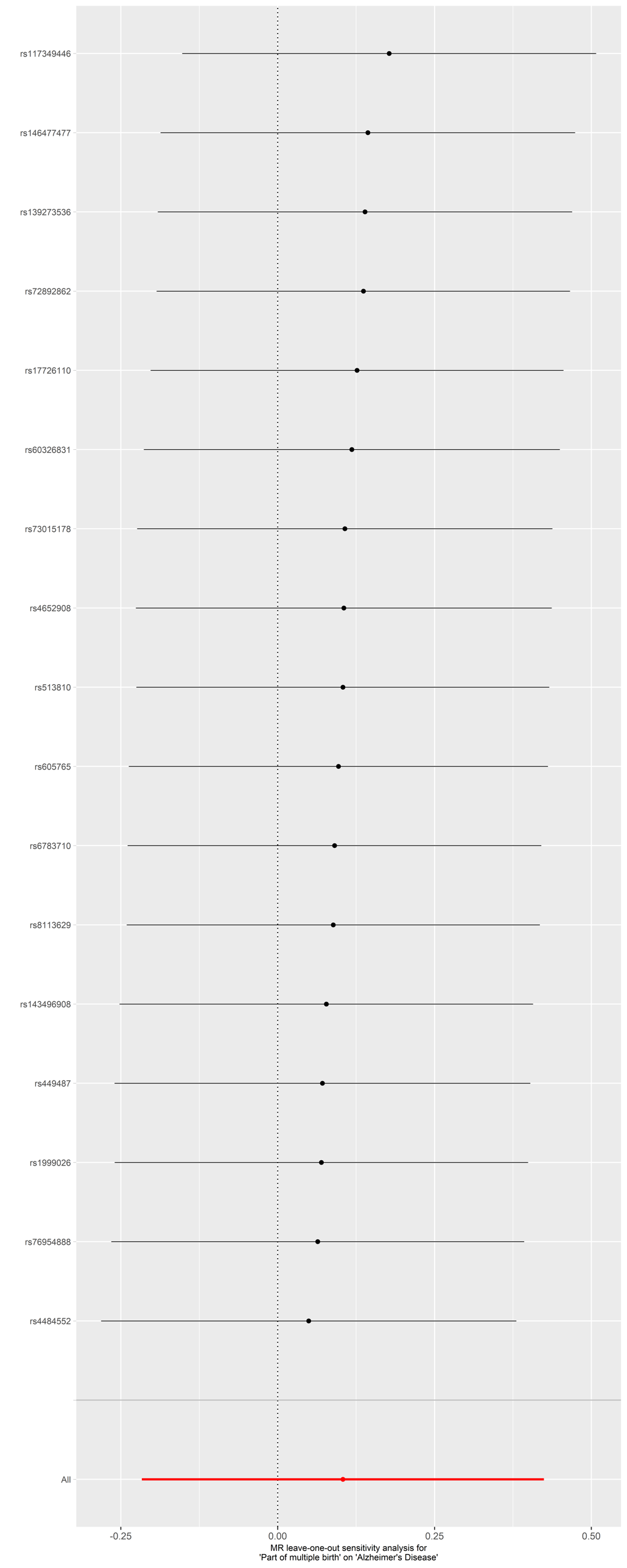


**Cerebral palsy – Finngen**


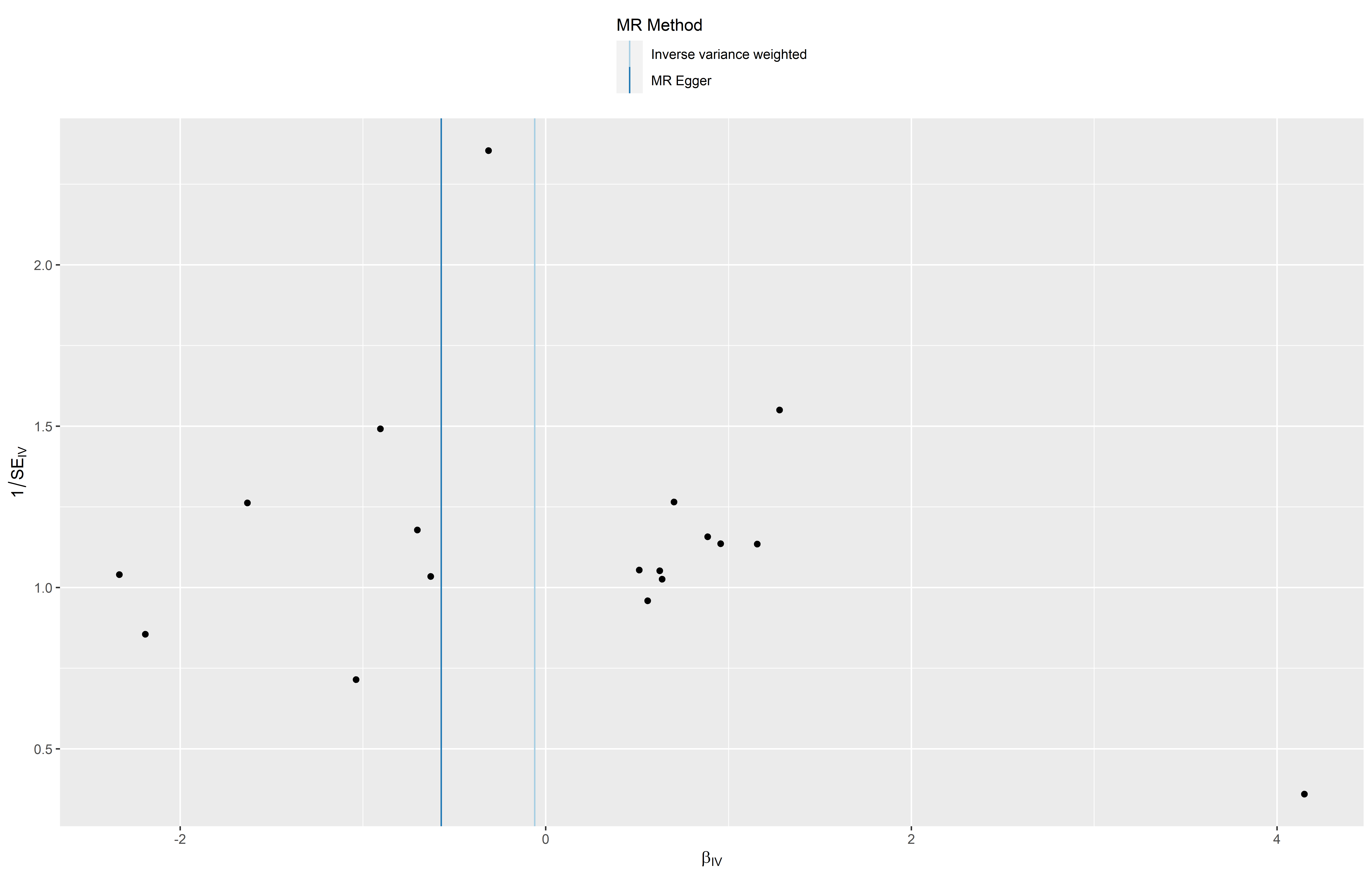

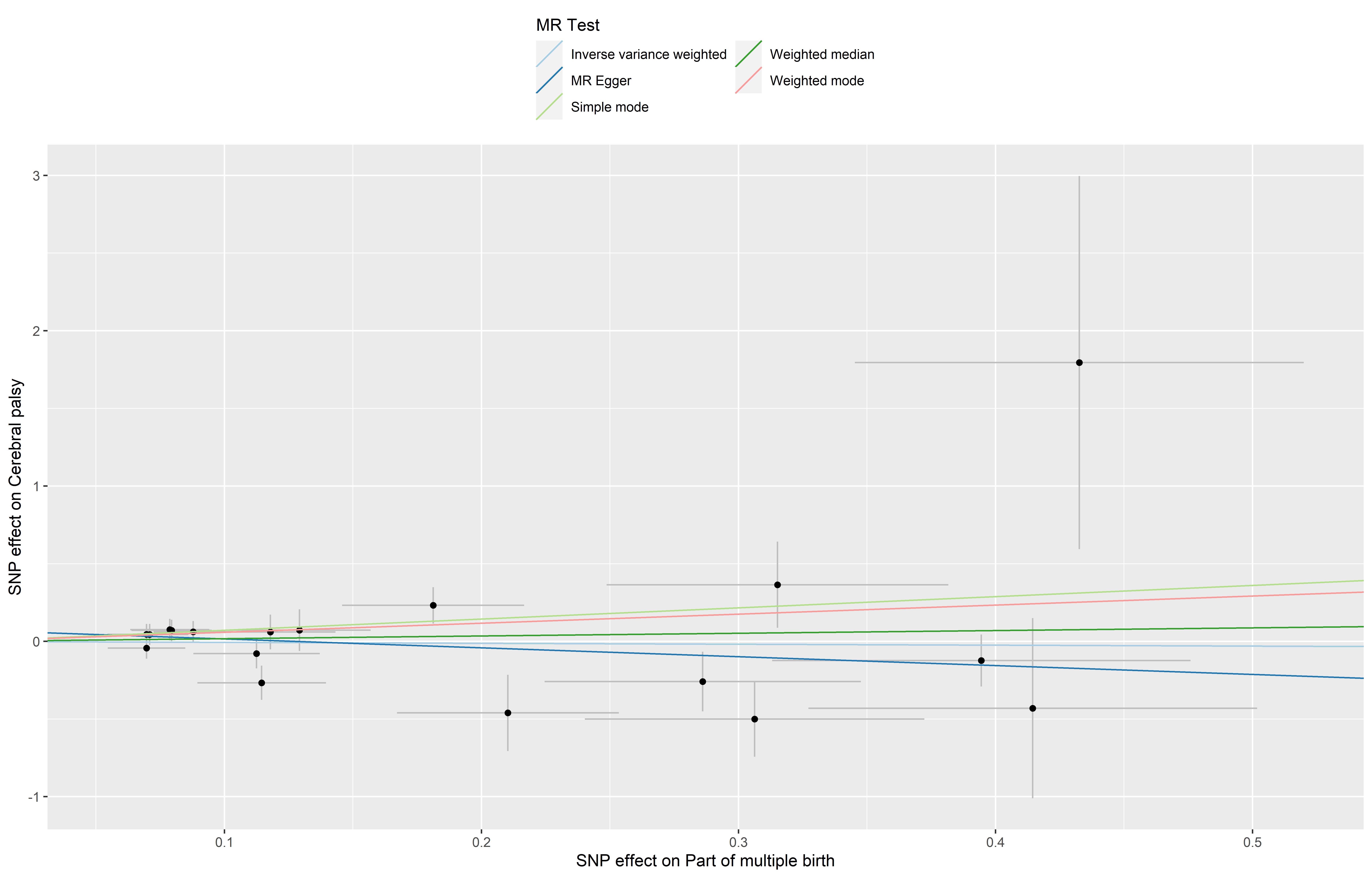


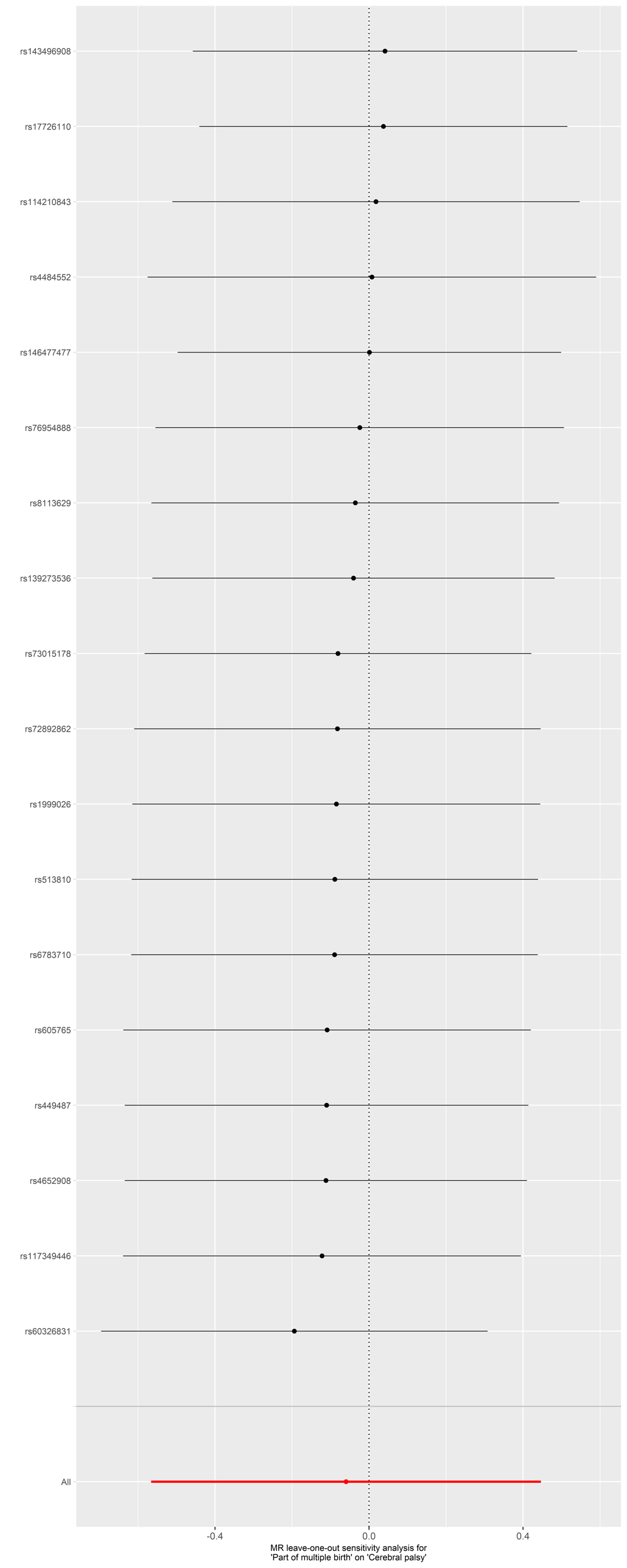


**Cerebral palsy – UK Biobank**


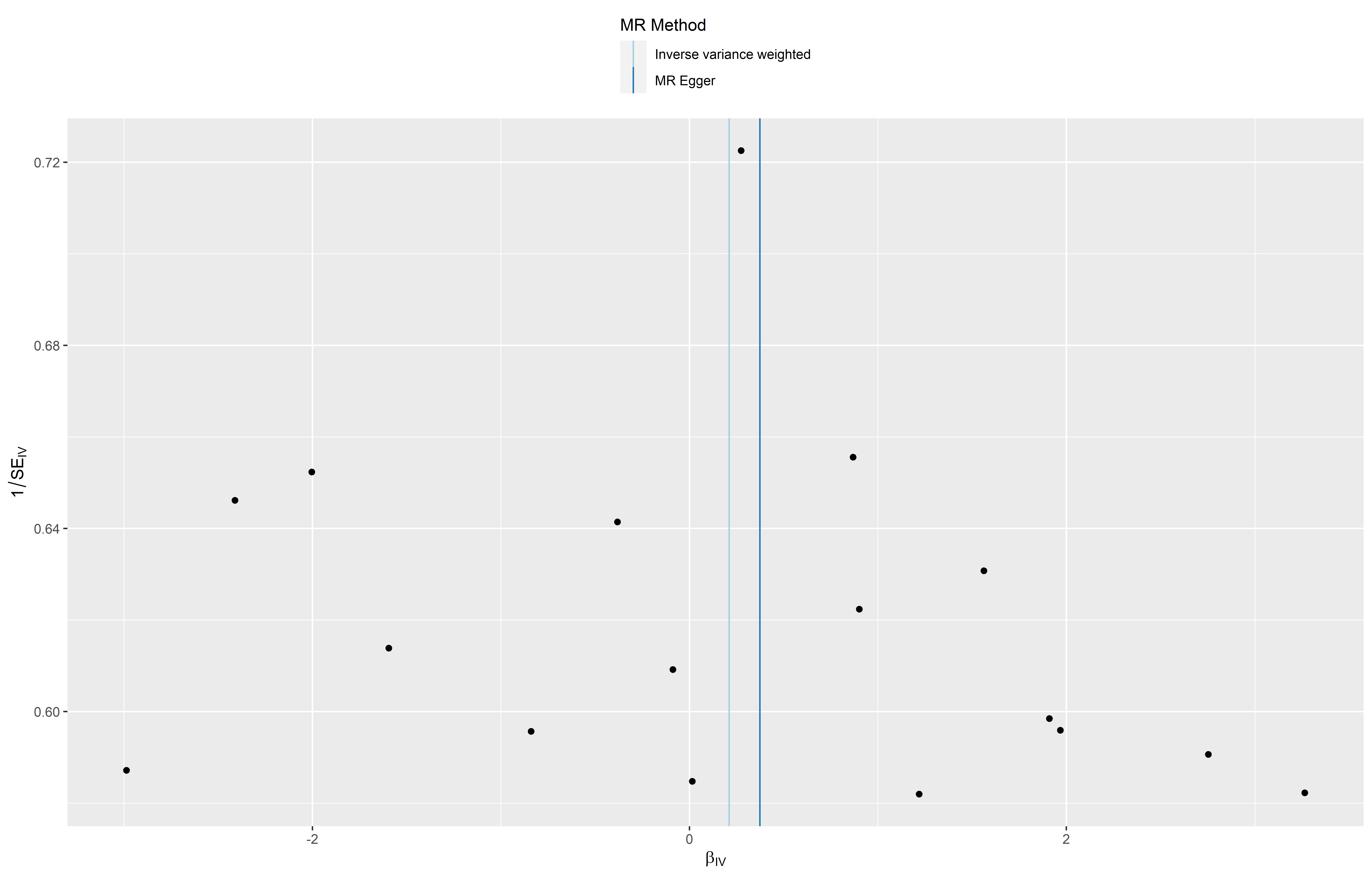

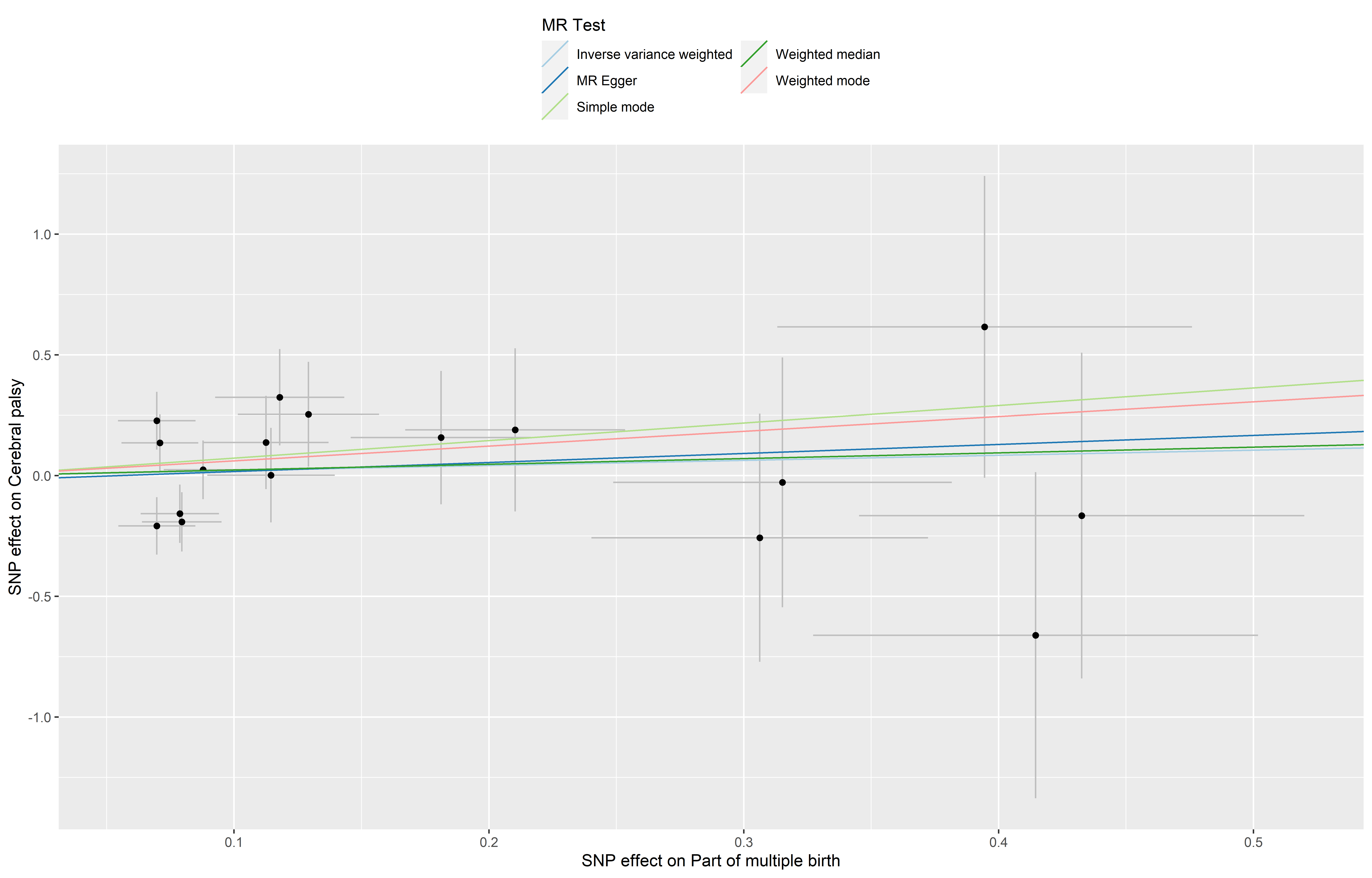


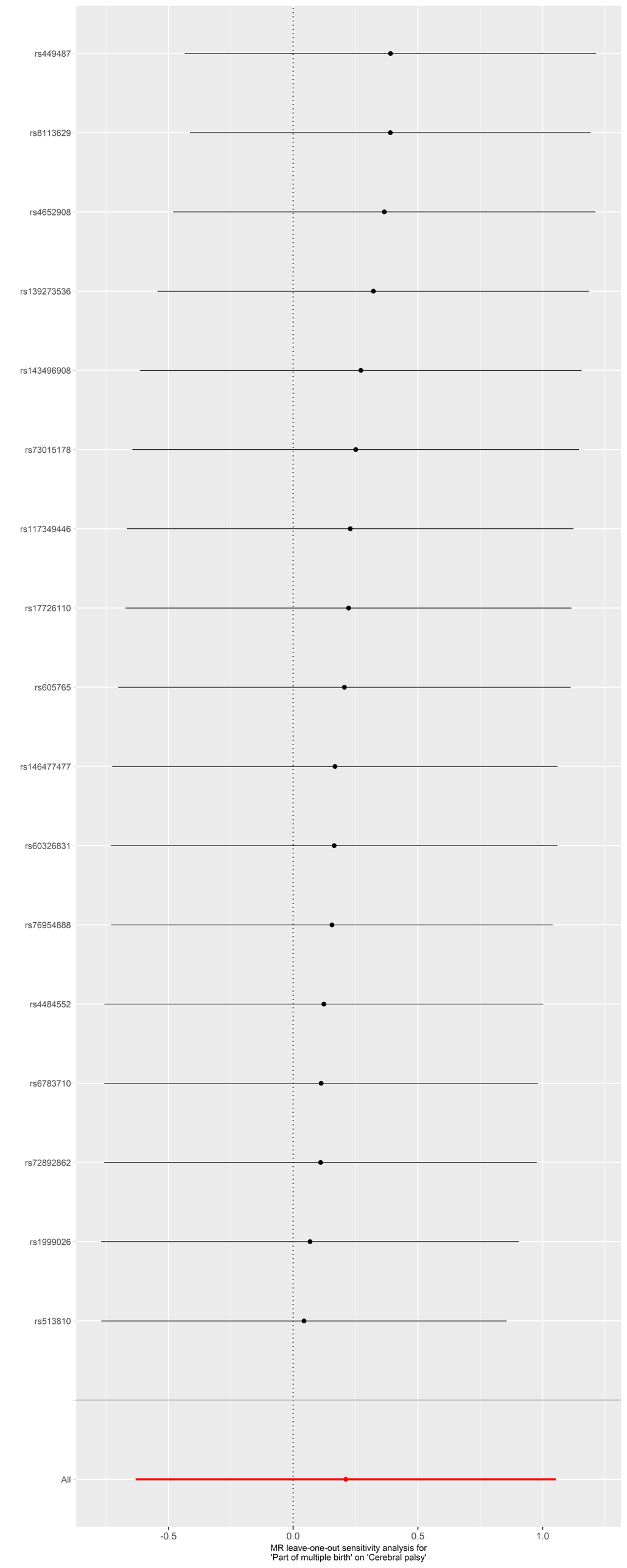


**Infantile cerebral palsy – UK Biobank**


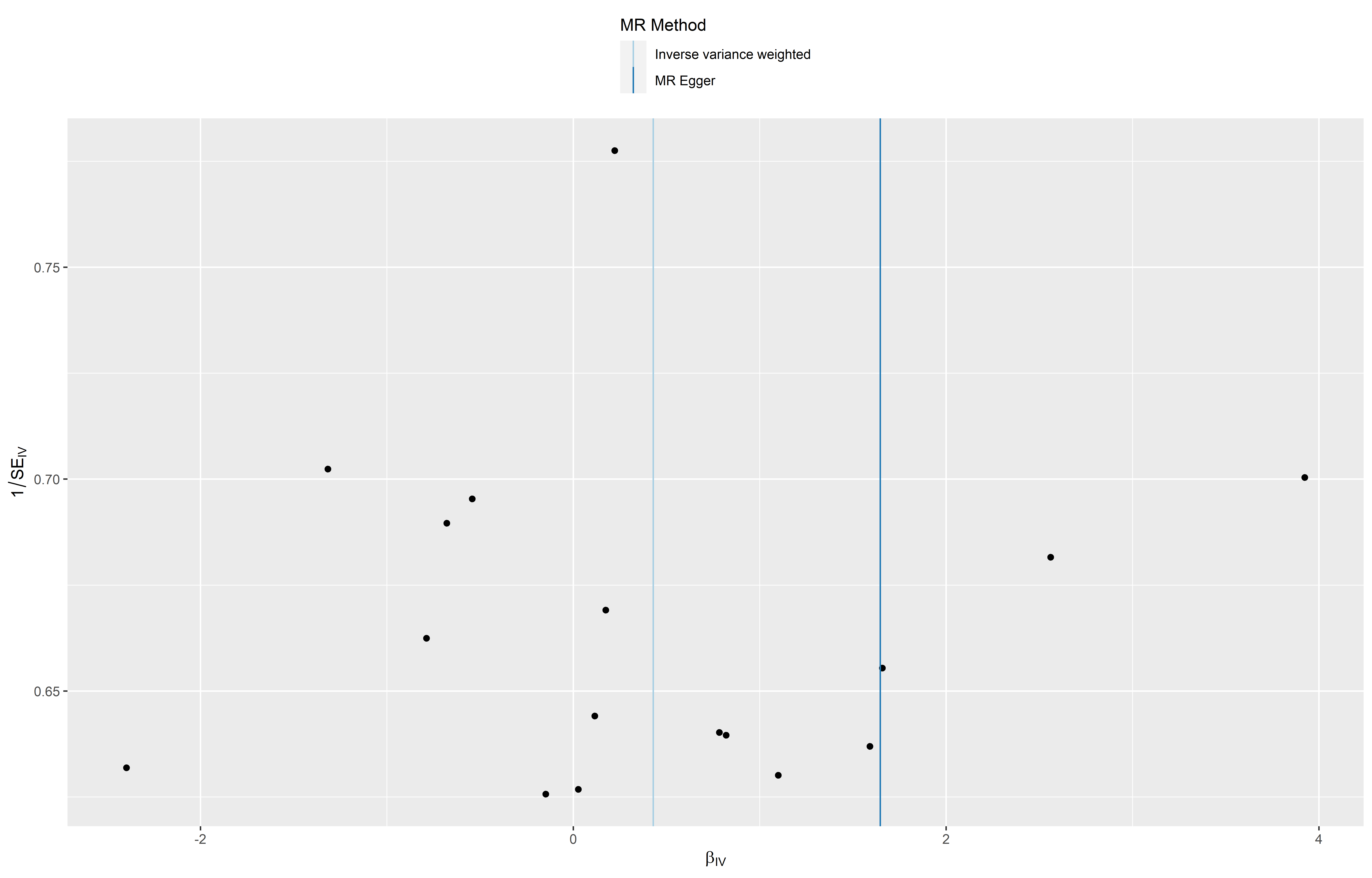

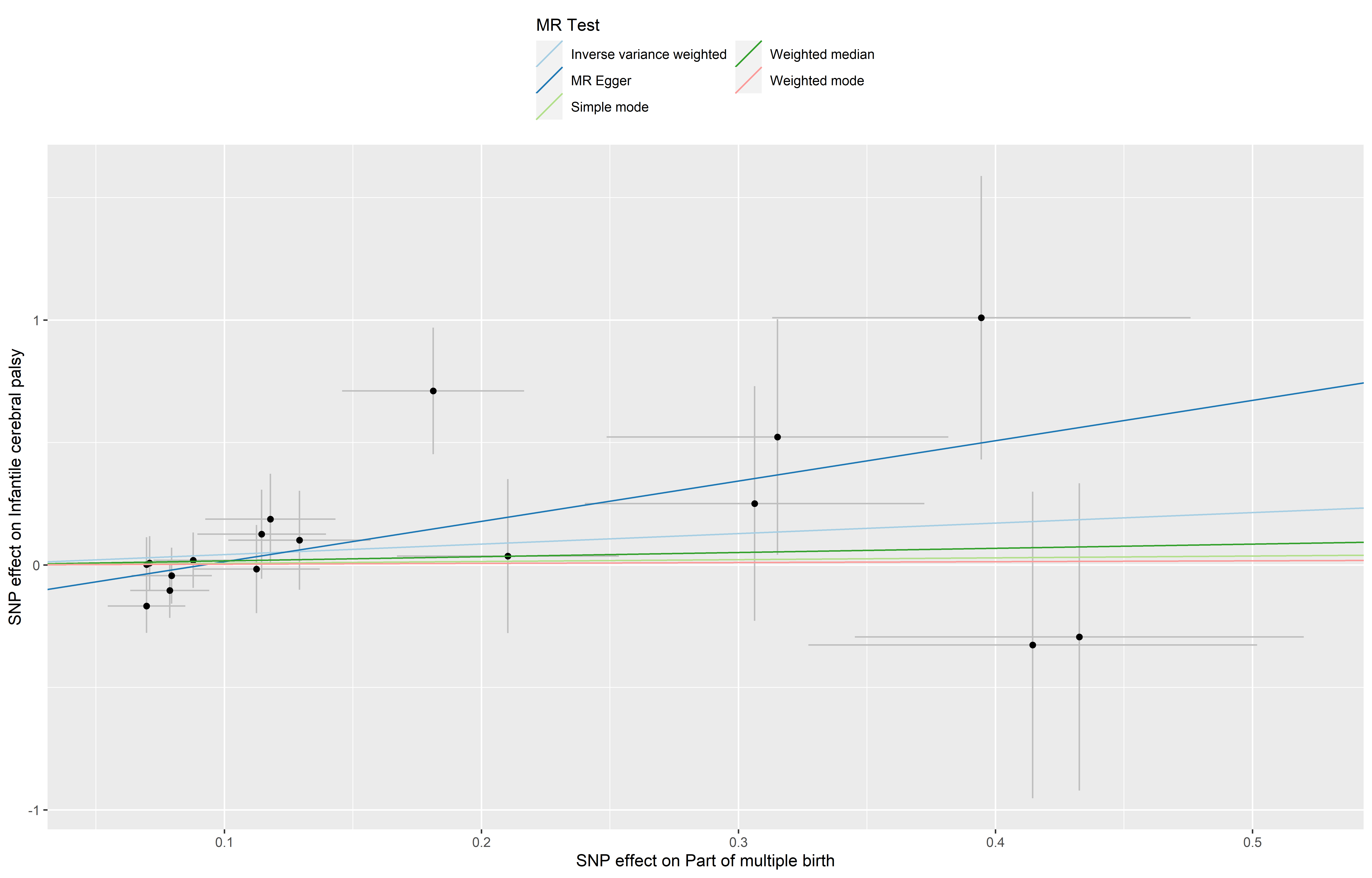


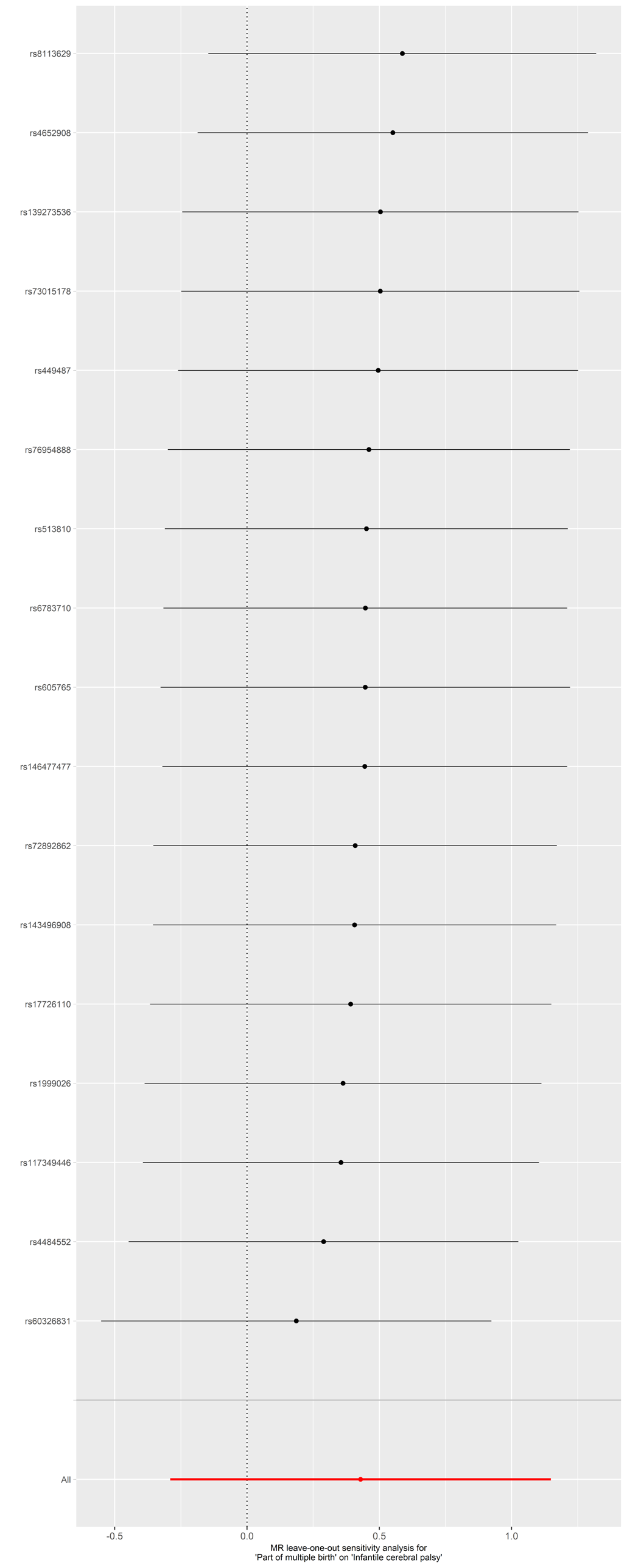


**Mental retardation – Finngen**


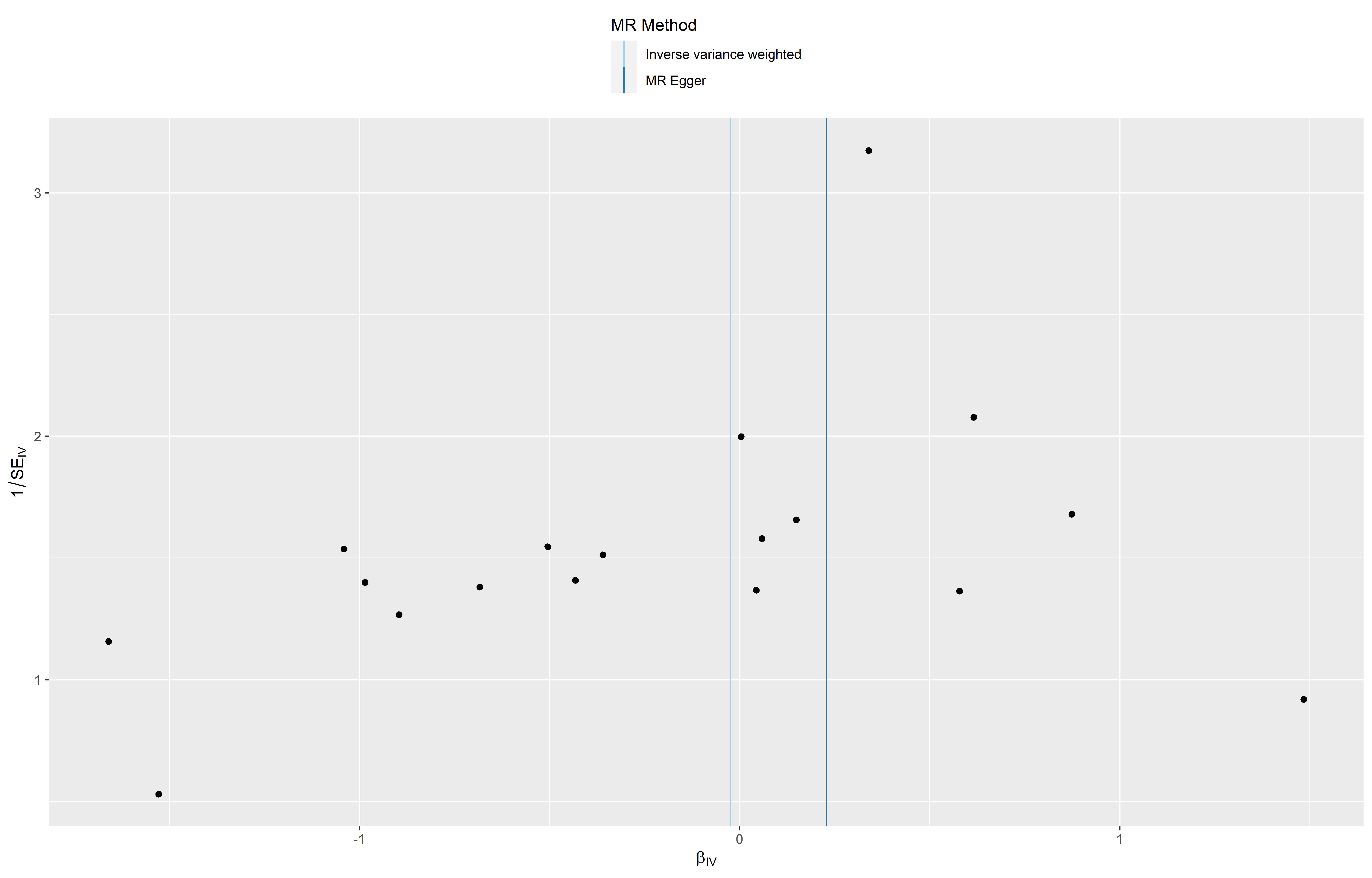

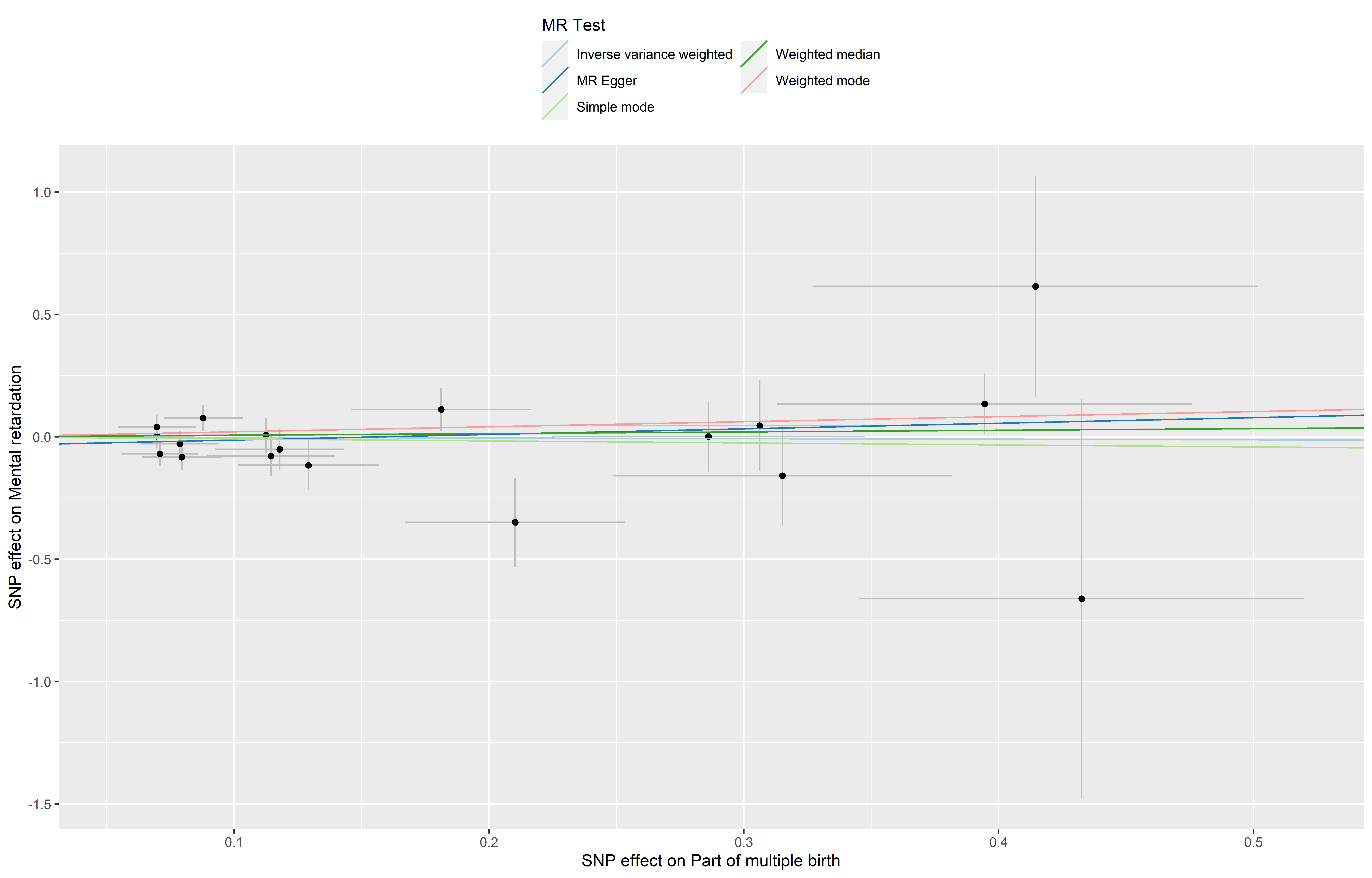


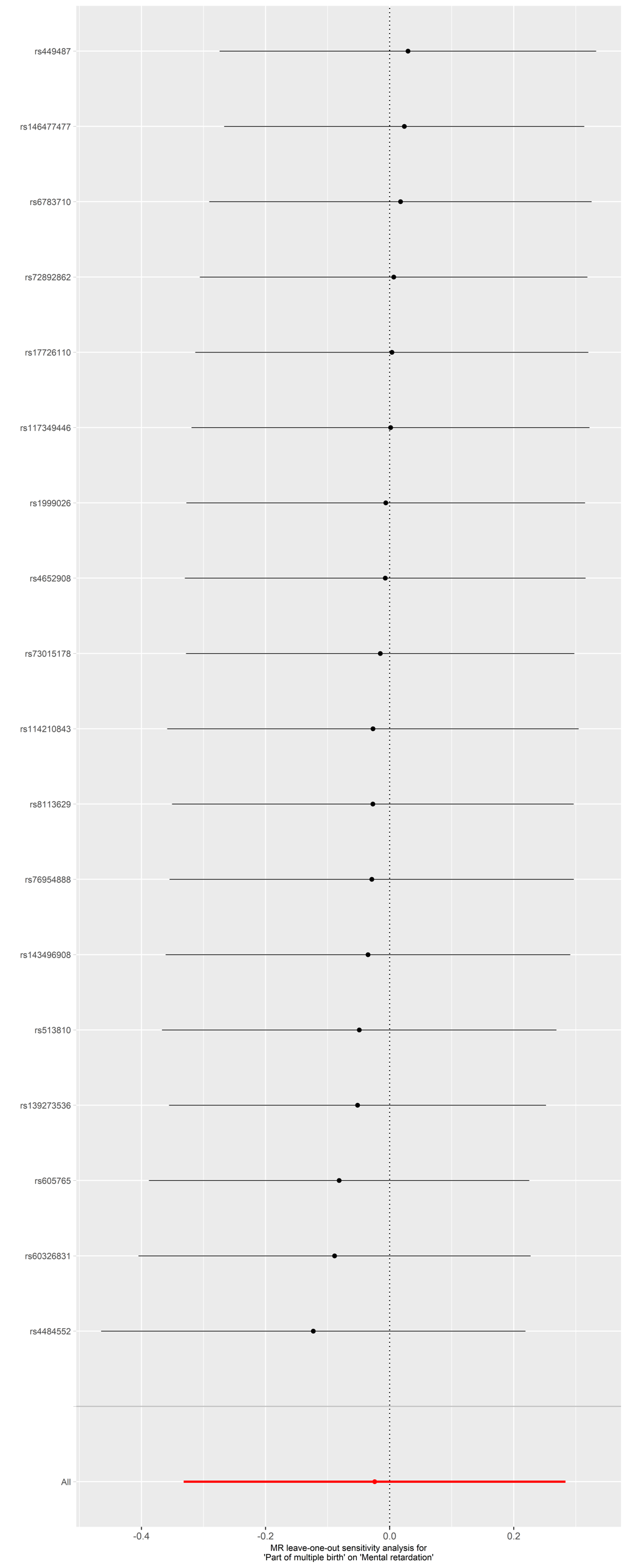

Supplement: Supplementary file 10 — Additional file 10: Material S2. The scatter plot, funnel plot and leave-one-out plot for the MR analysis of multiple birth and nervous system disease. [file 12967_2023_4423_MOESM10_ESM.docx]
