# Supplementary material for: Long-term outcomes of offspring from multiple gestations: a two-sample Mendelian randomization study on multi-system diseases using UK Biobank and FinnGen databases
Source: J Transl Med. 2023 Sep 8;21:608. doi: 10.1186/s12967-023-04423-w (PMC10492369; doi:10.1186/s12967-023-04423-w)

**Arterial hypertension – Finngen**


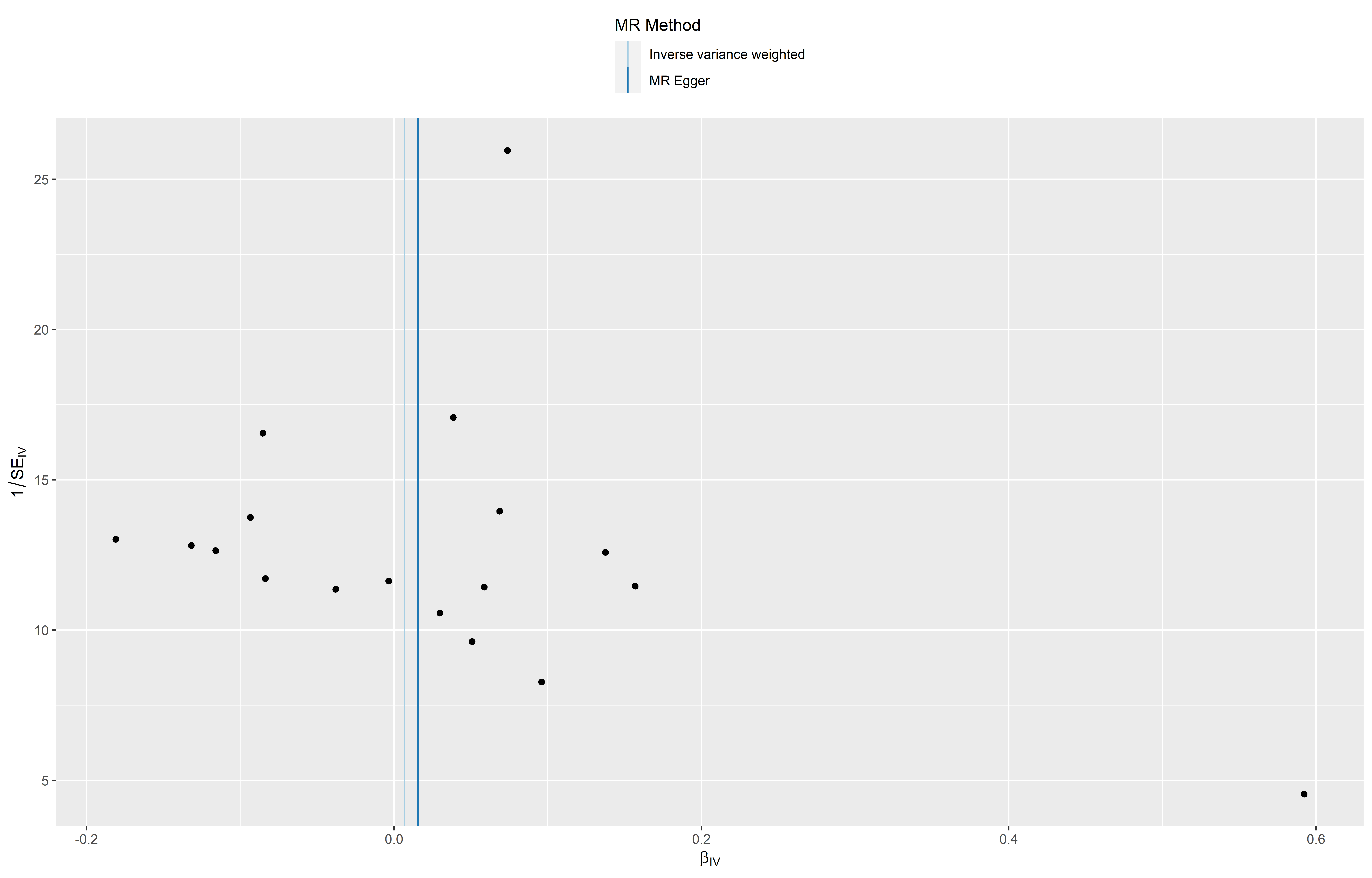

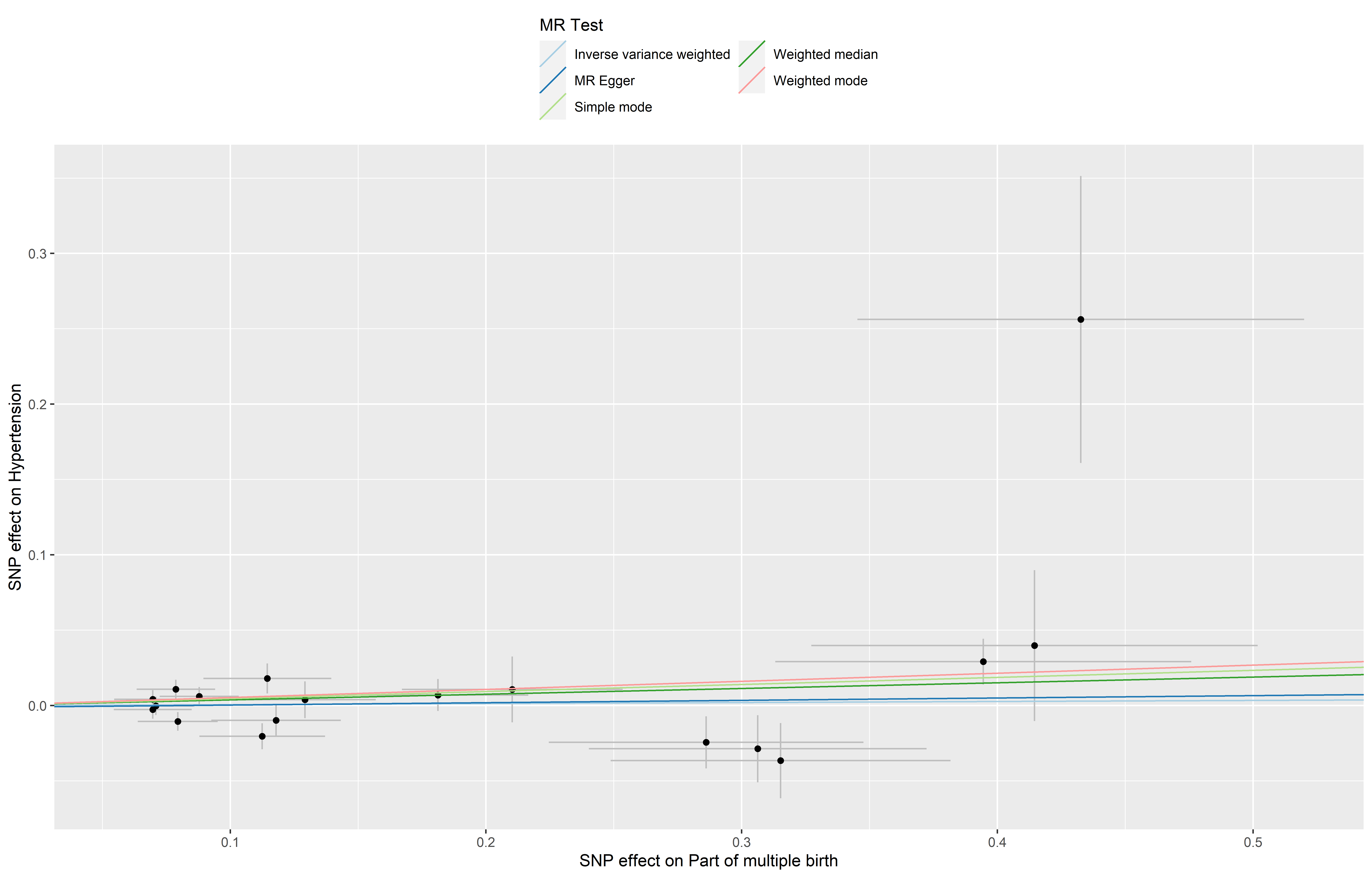


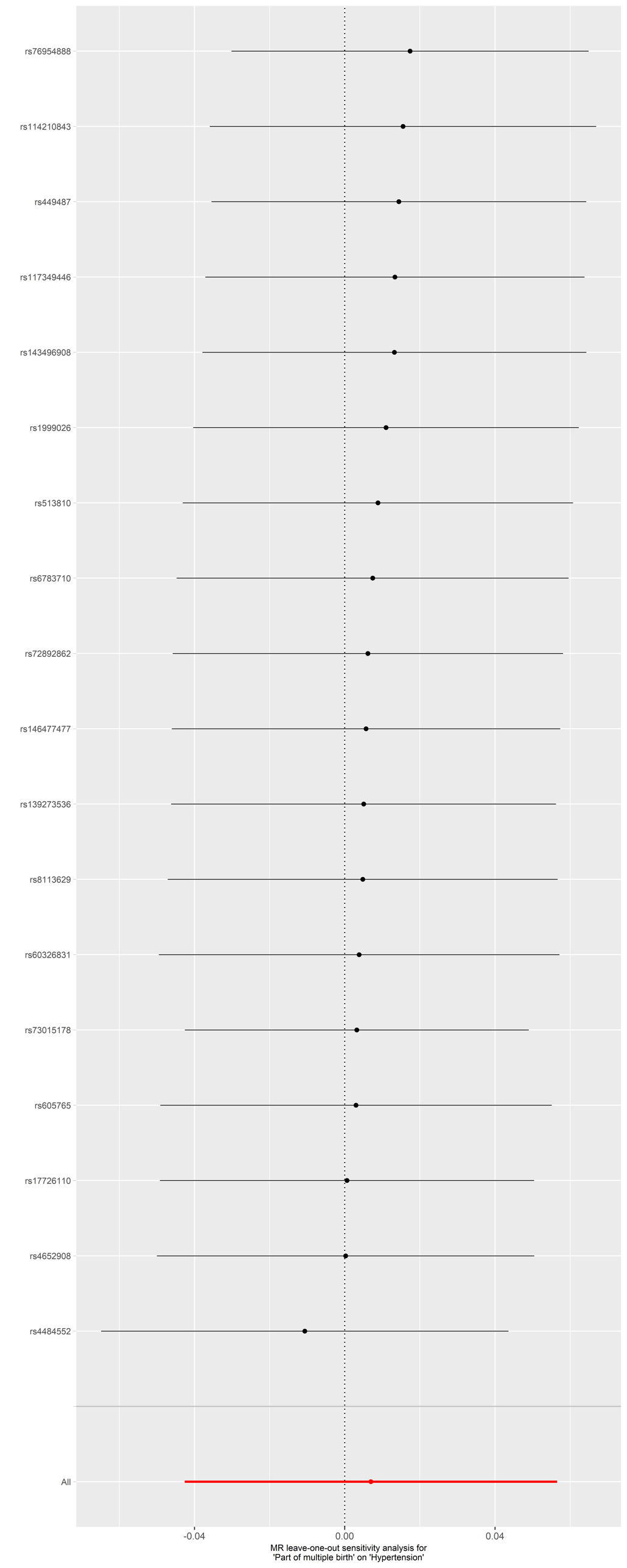


**Arterial hypertension – UK Biobank**


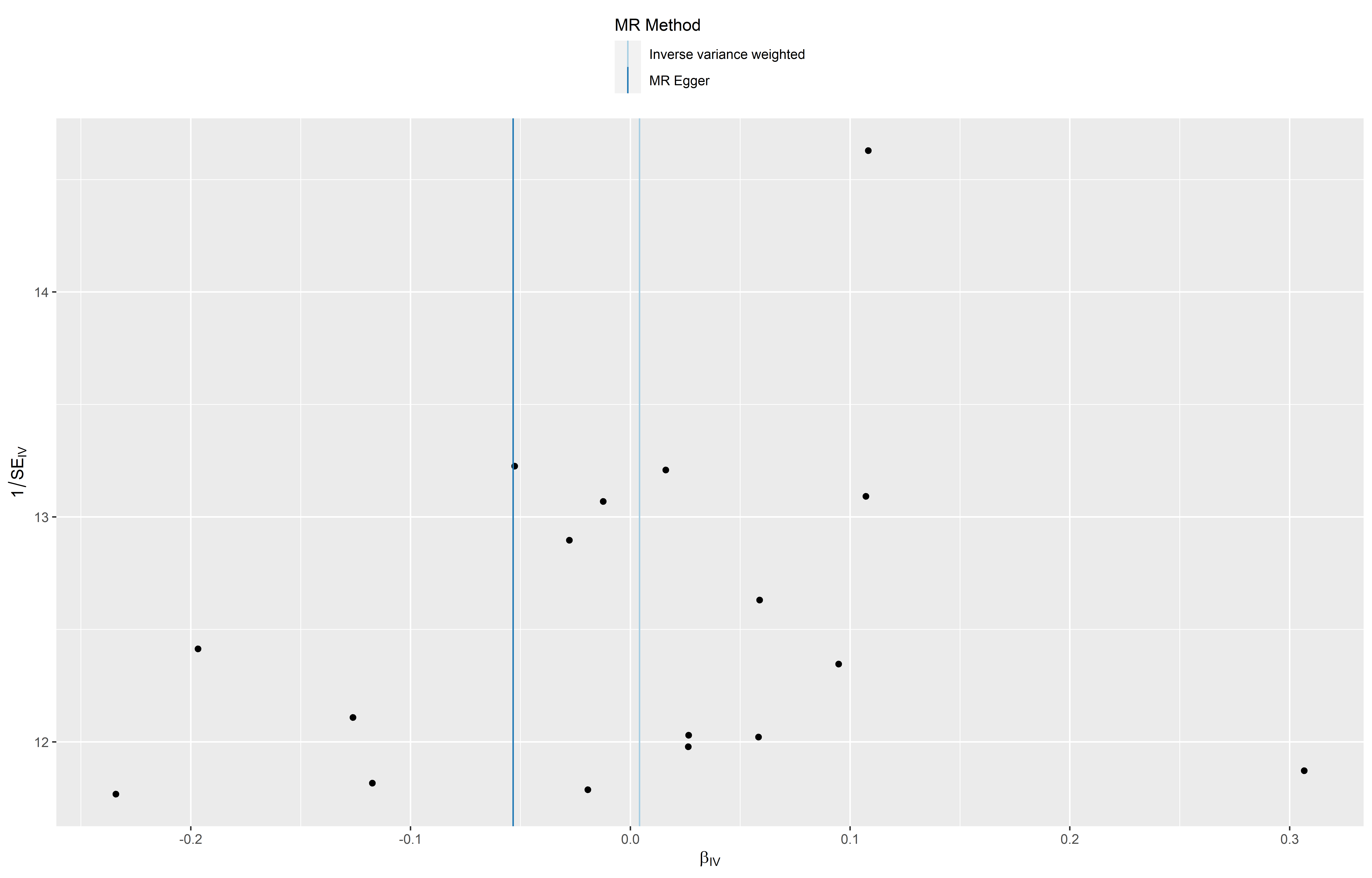

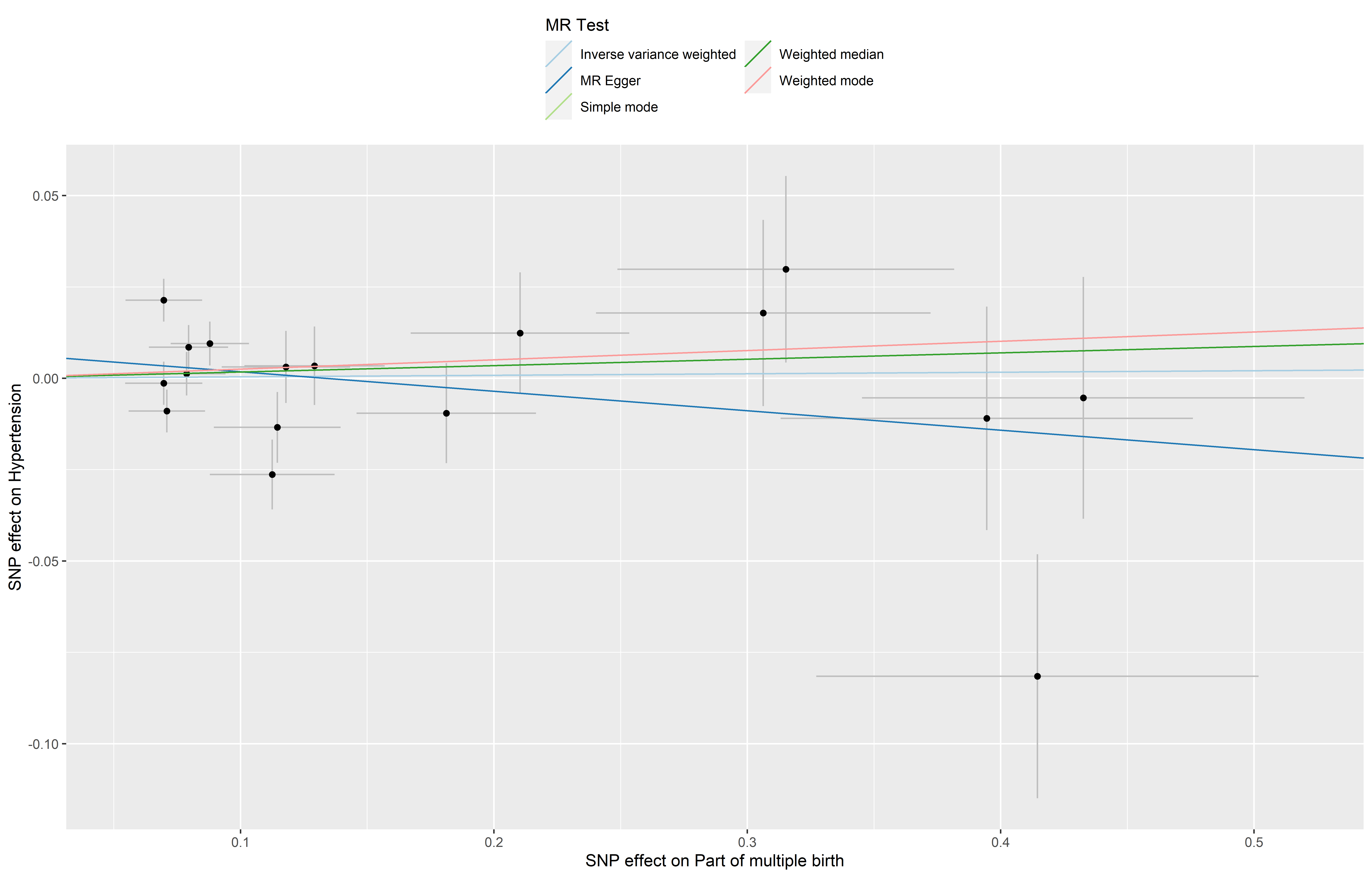


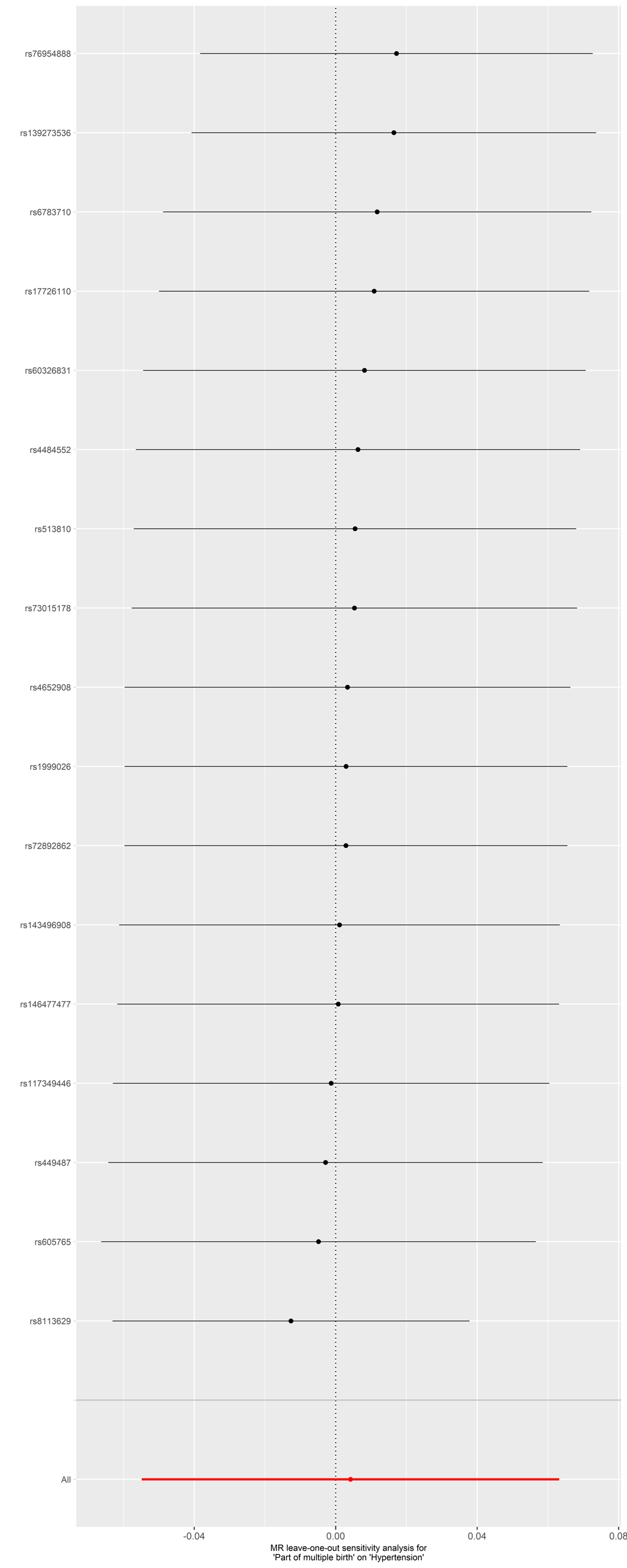


**Atrial fibrillation and flutter – Finngen**


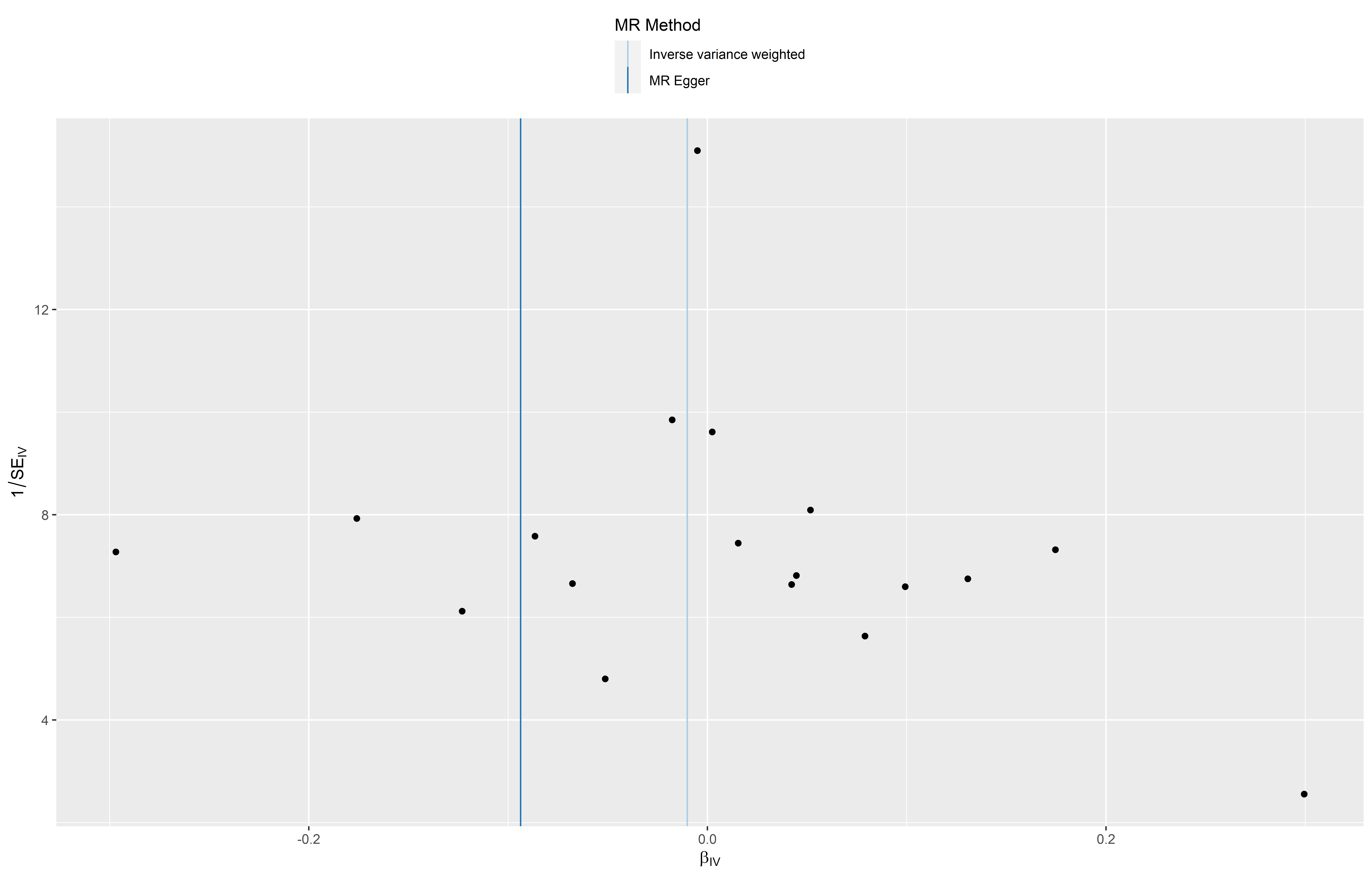

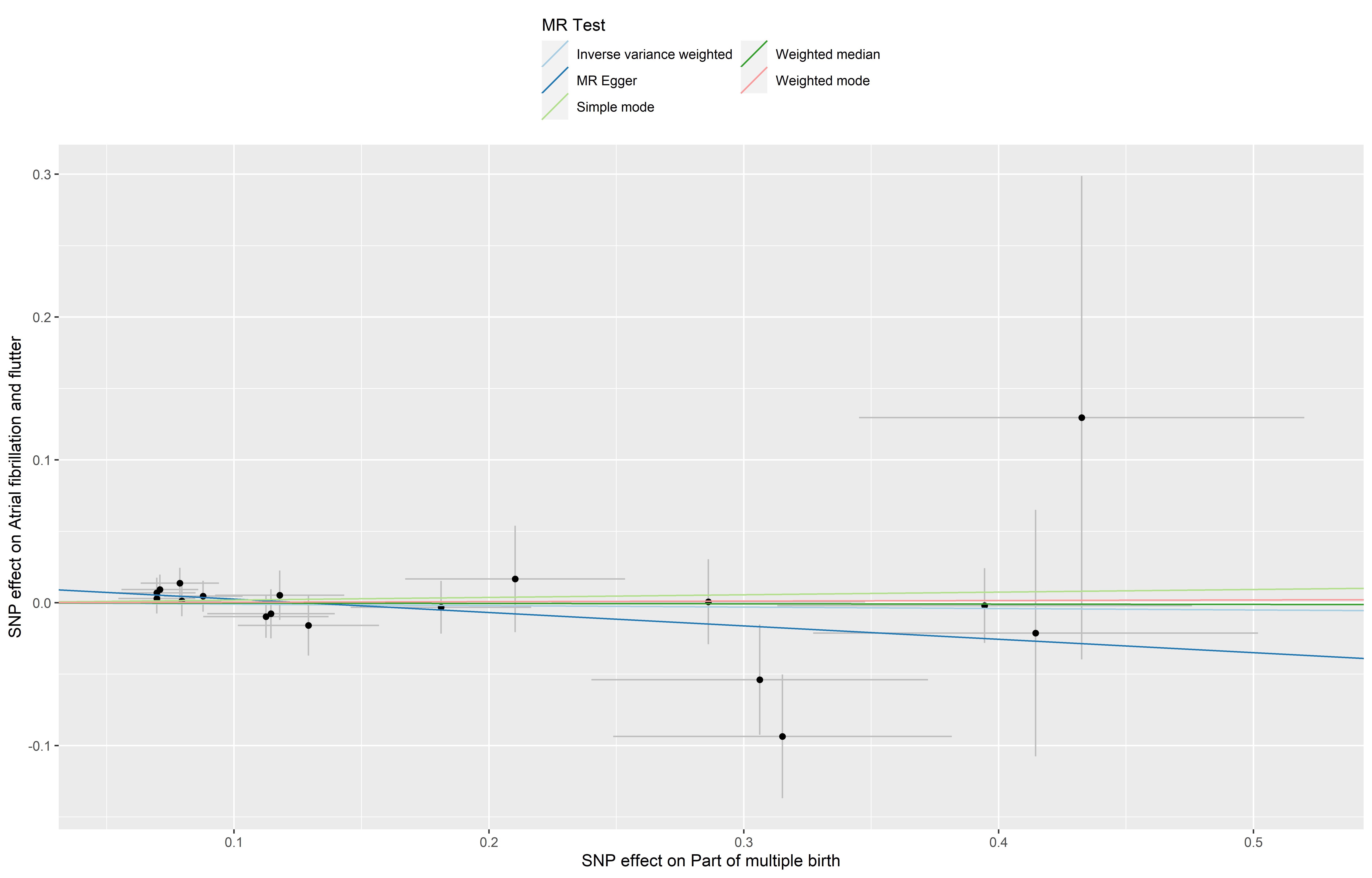


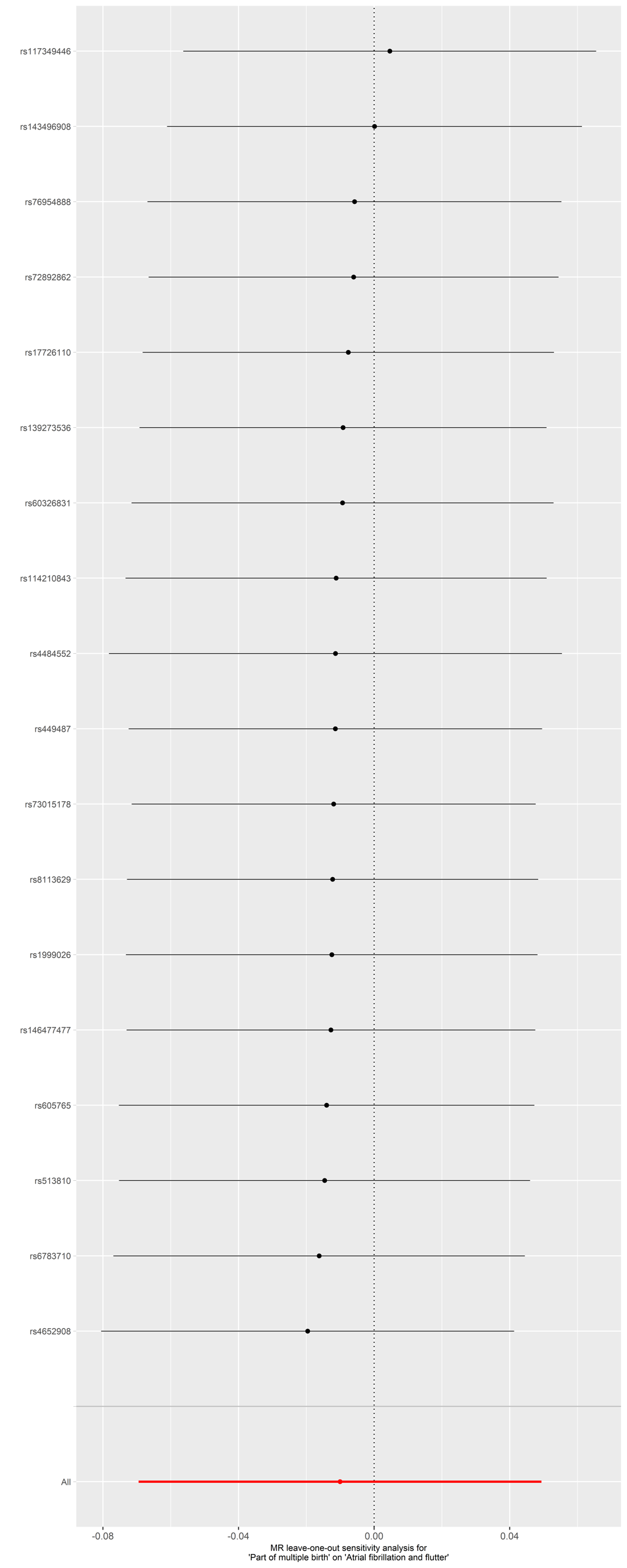


**Atrial fibrillation and flutter – UK Biobank**


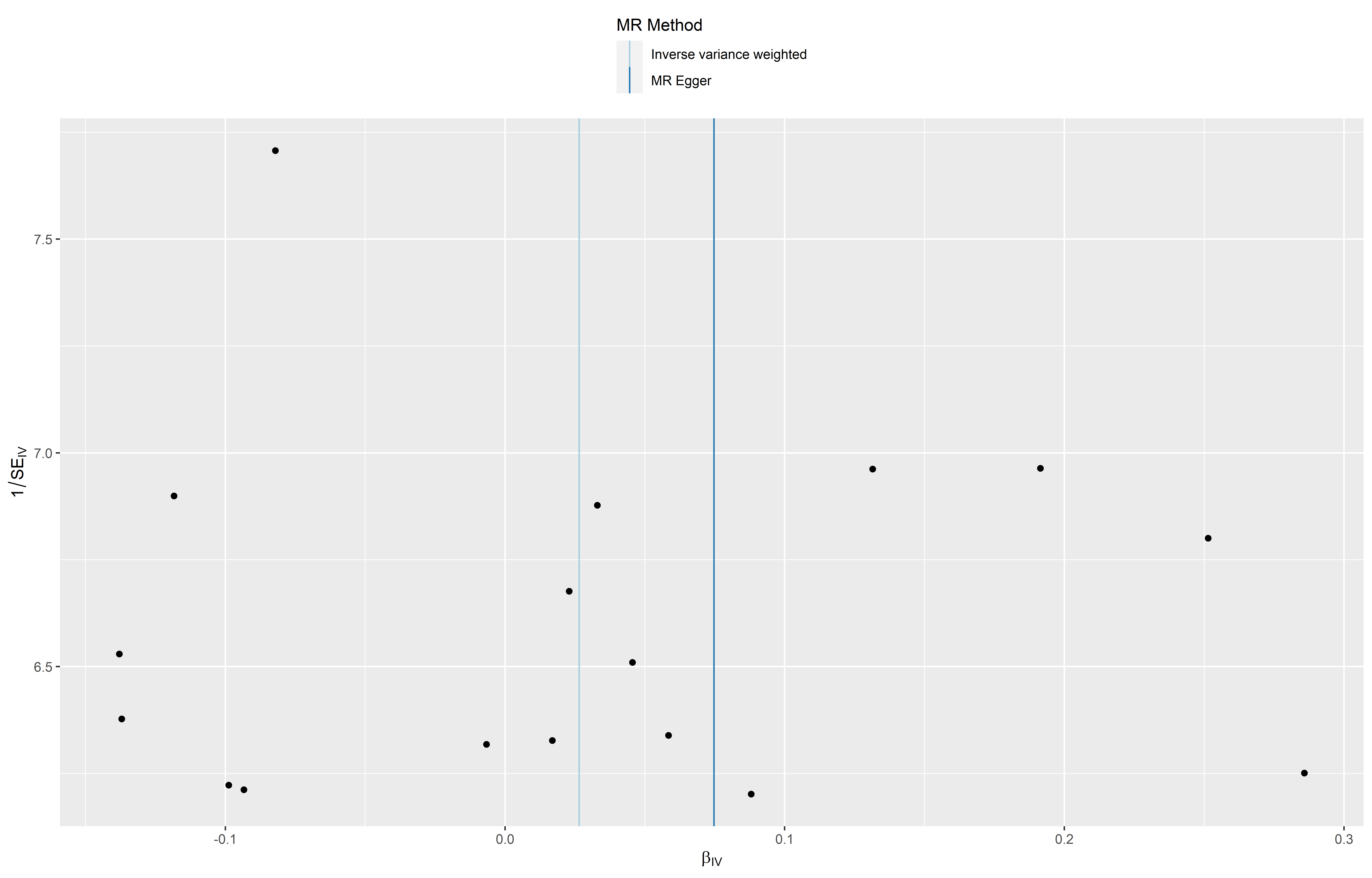

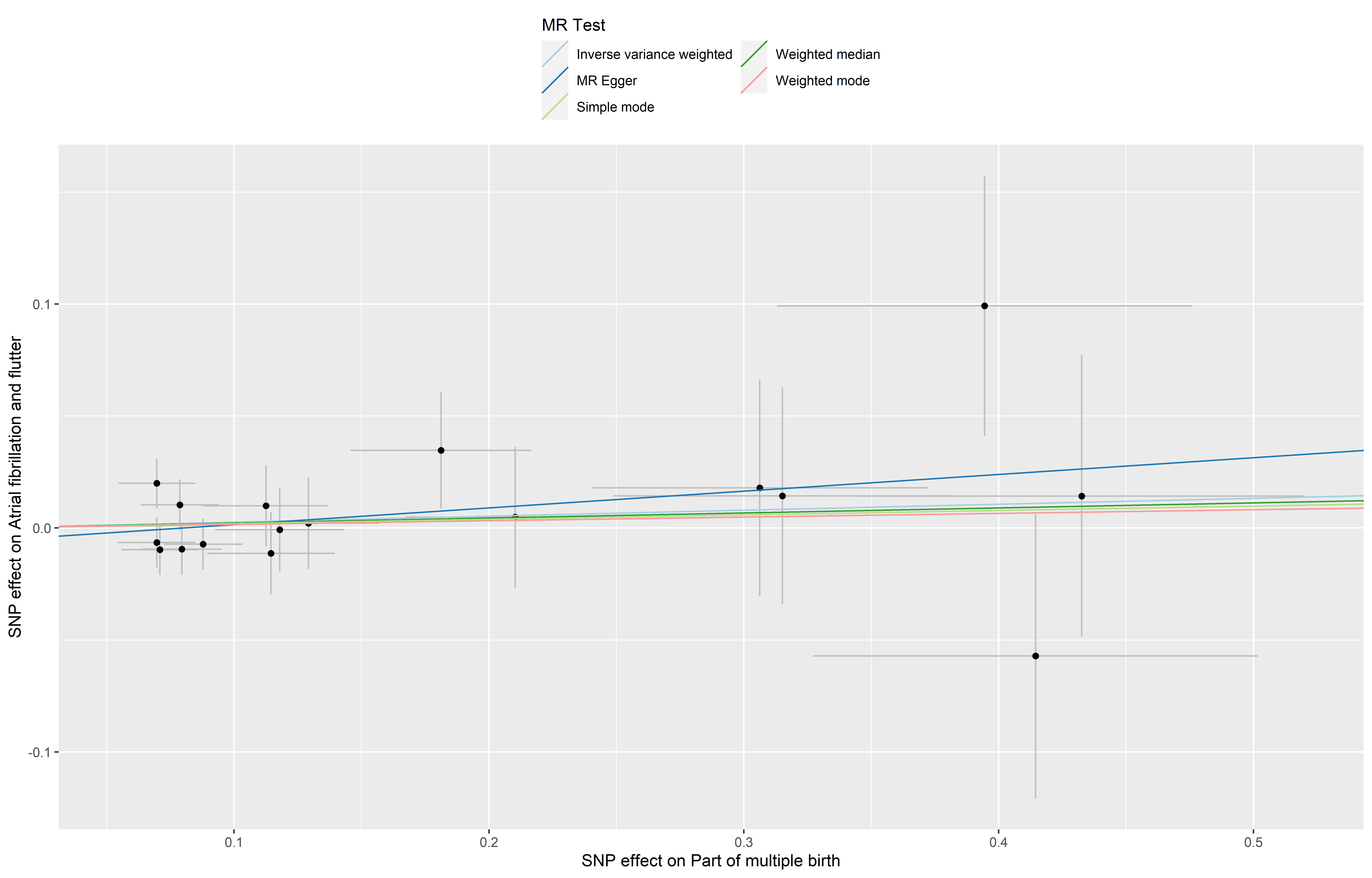


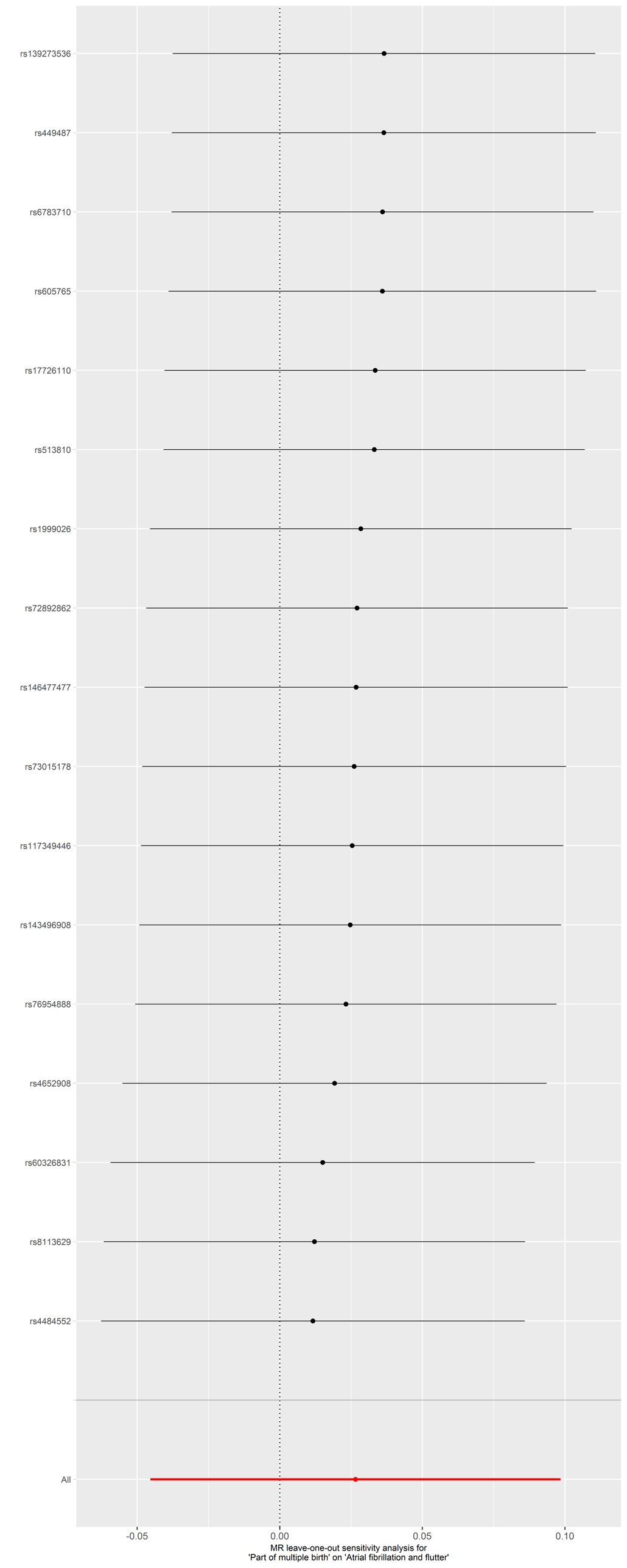


**Ischaemic heart disease – Finngen**


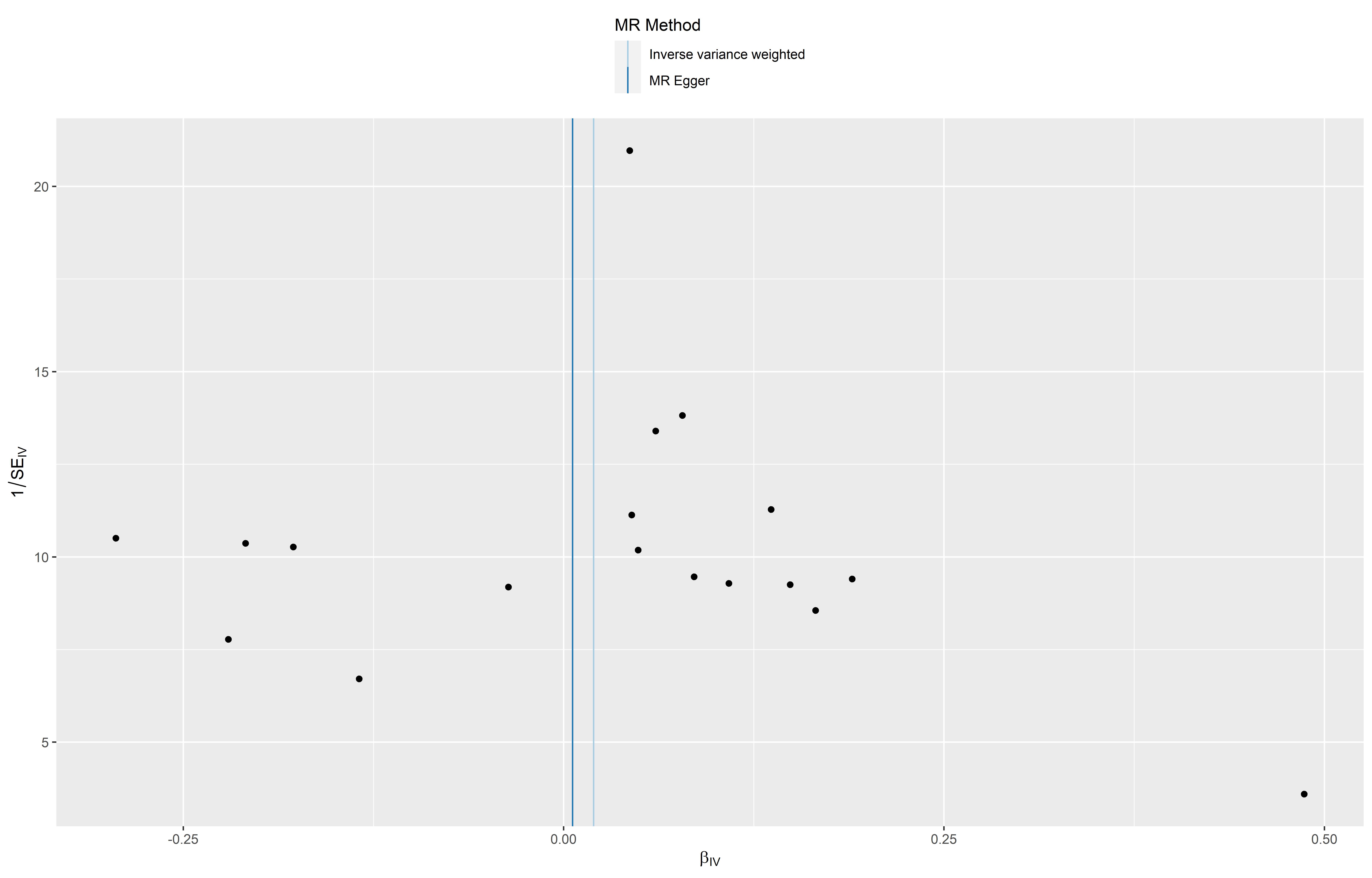

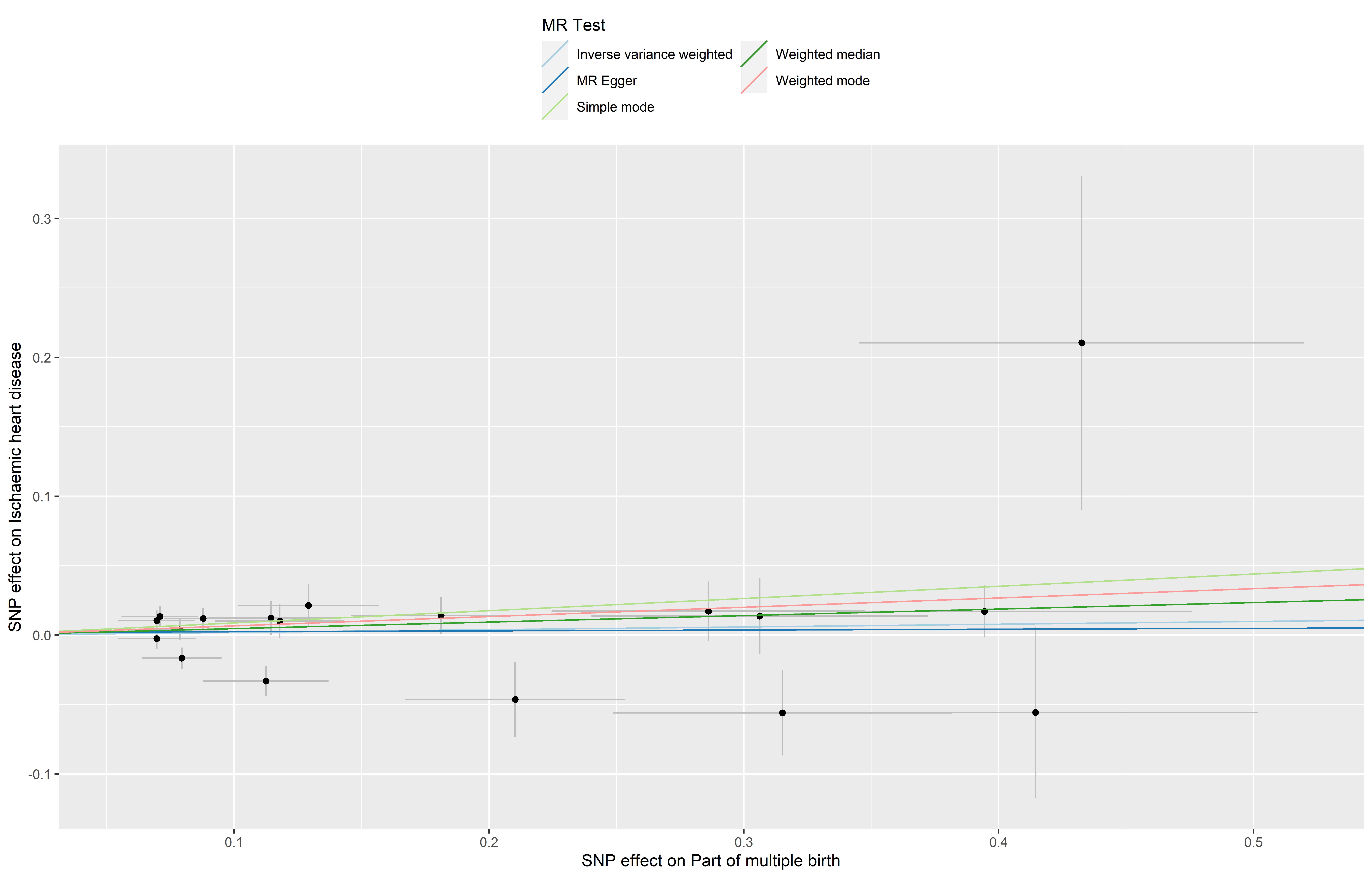


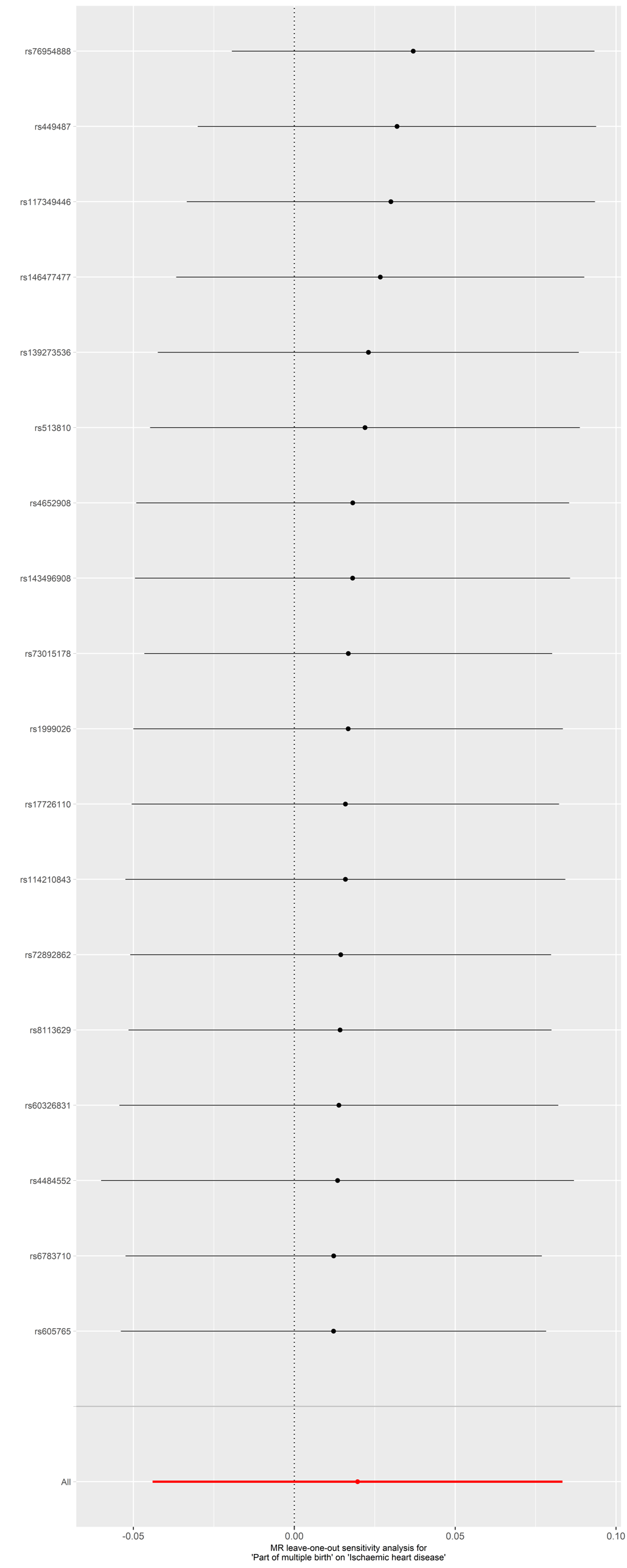


**Ischaemic heart disease – UK Biobank**


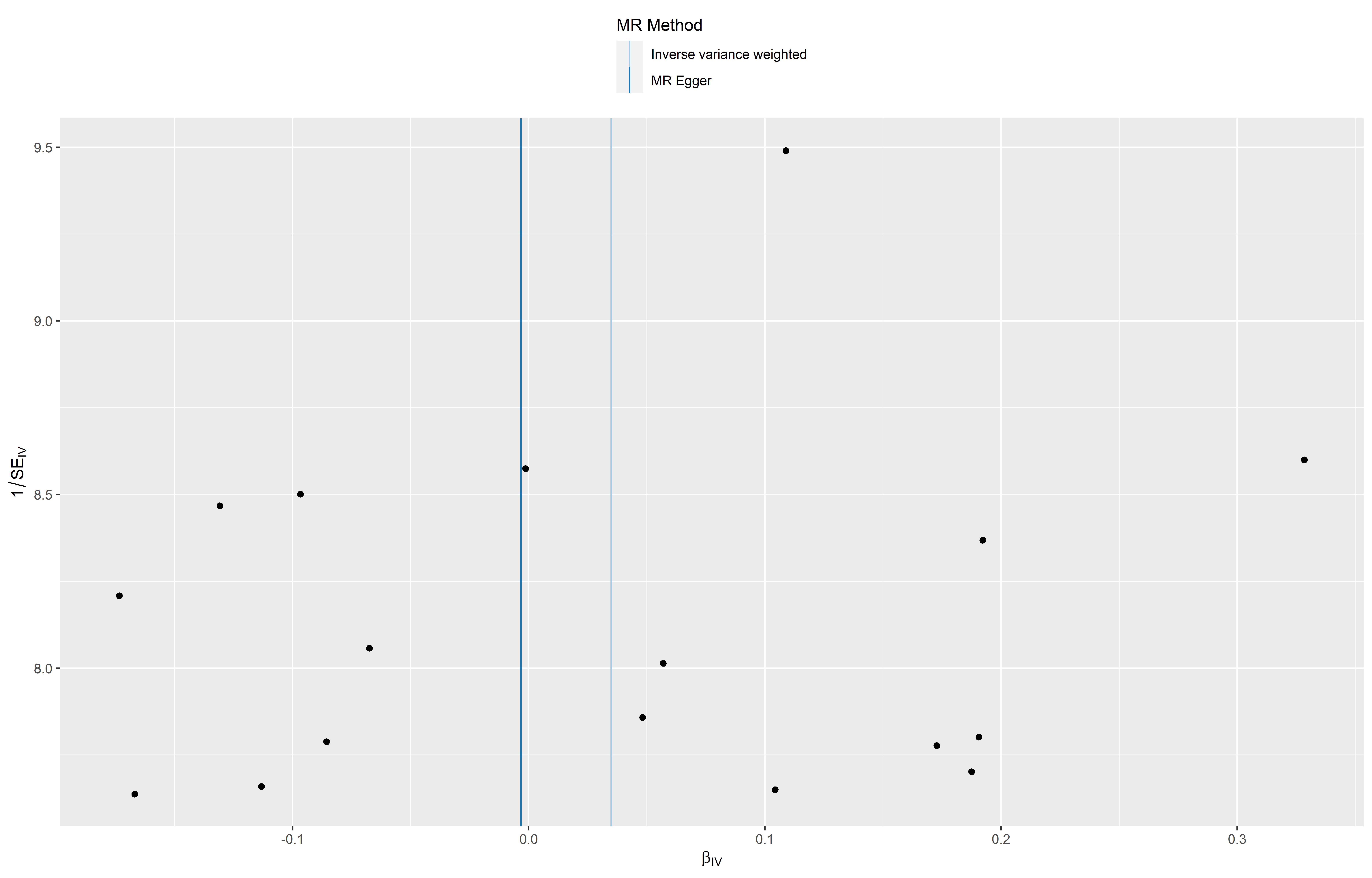

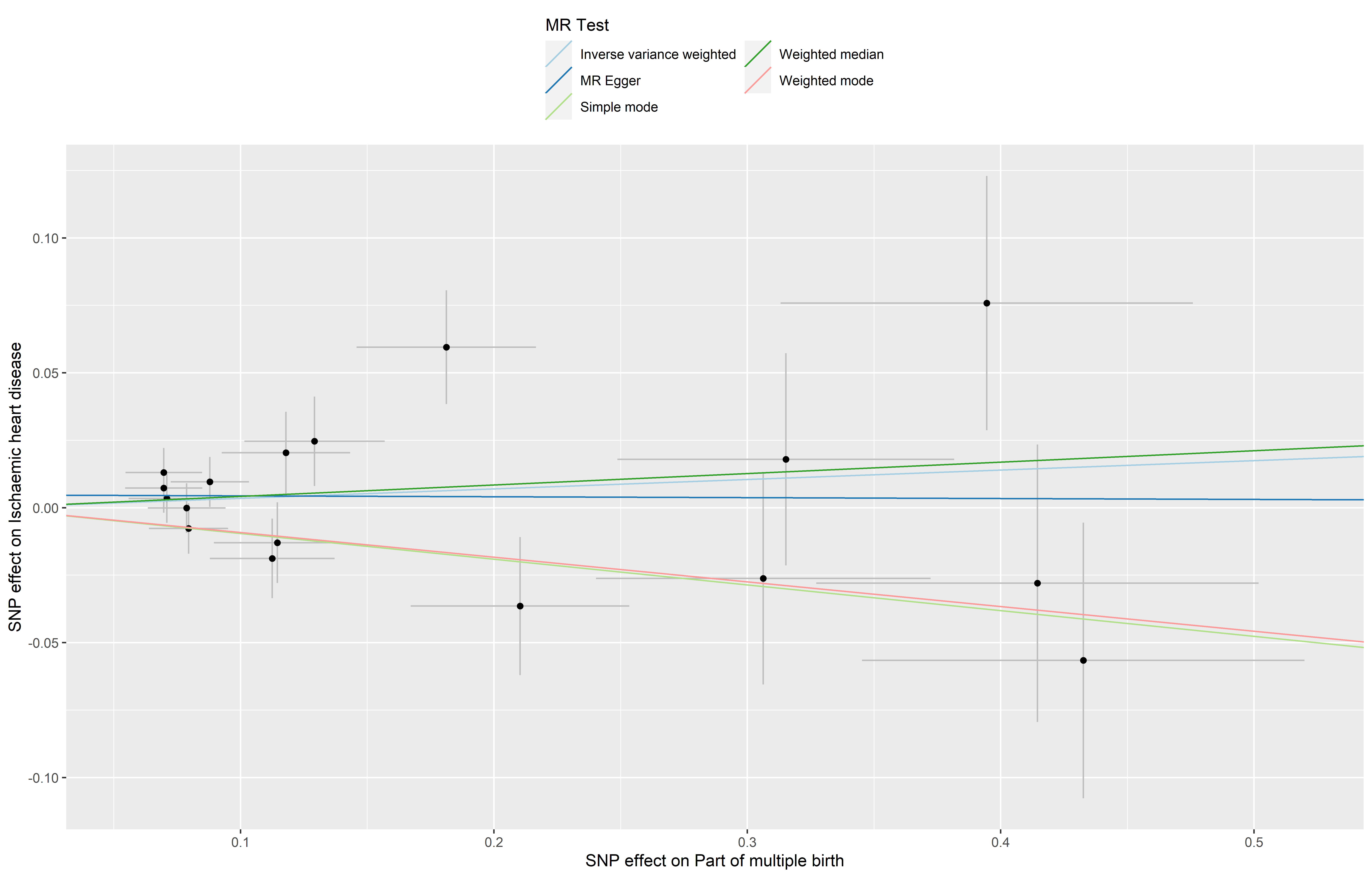


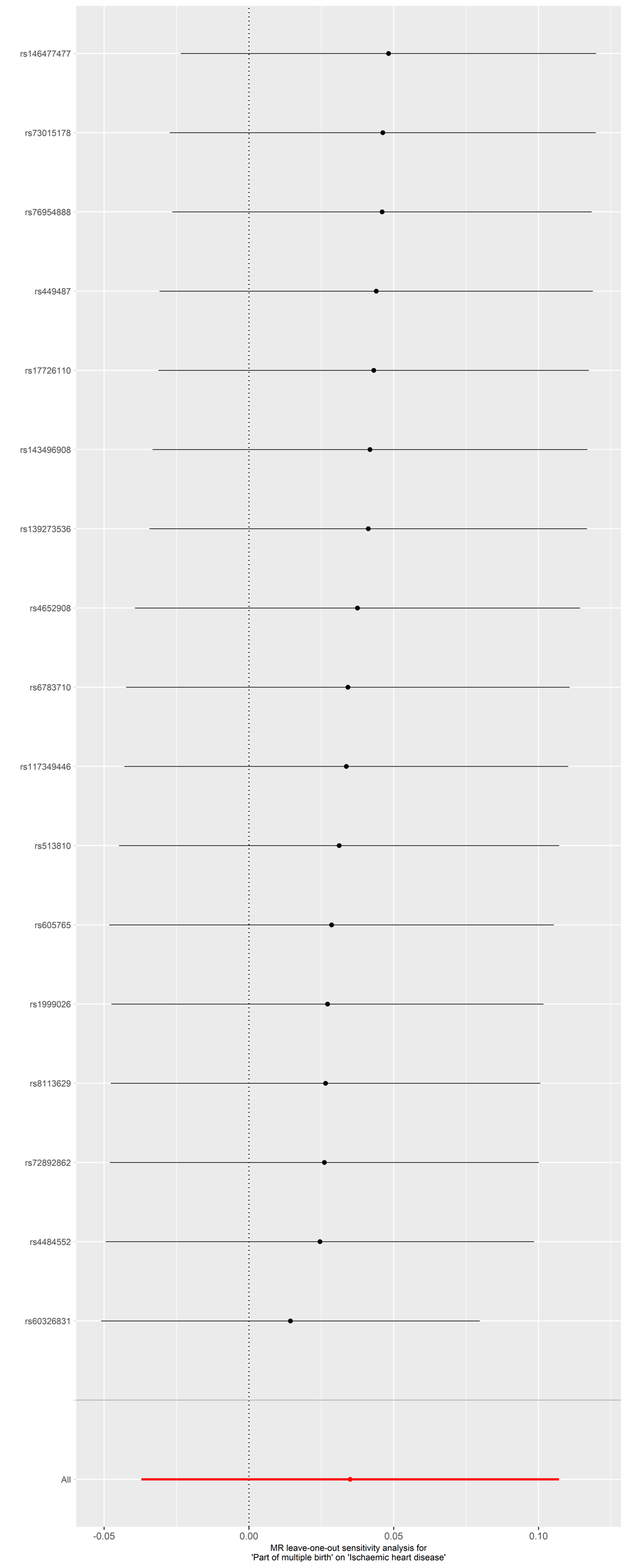


**Coronary heart disease – Finngen**


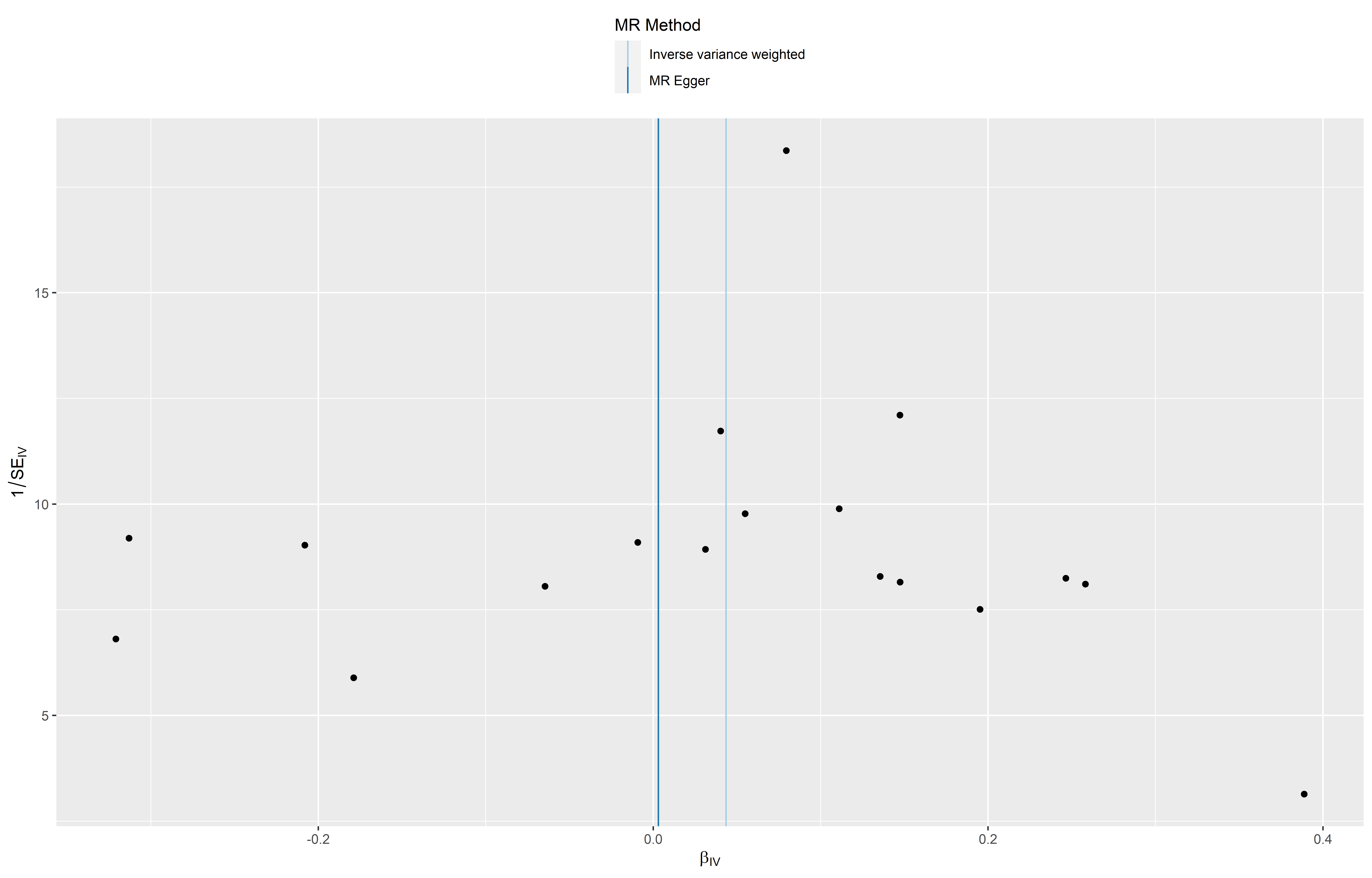

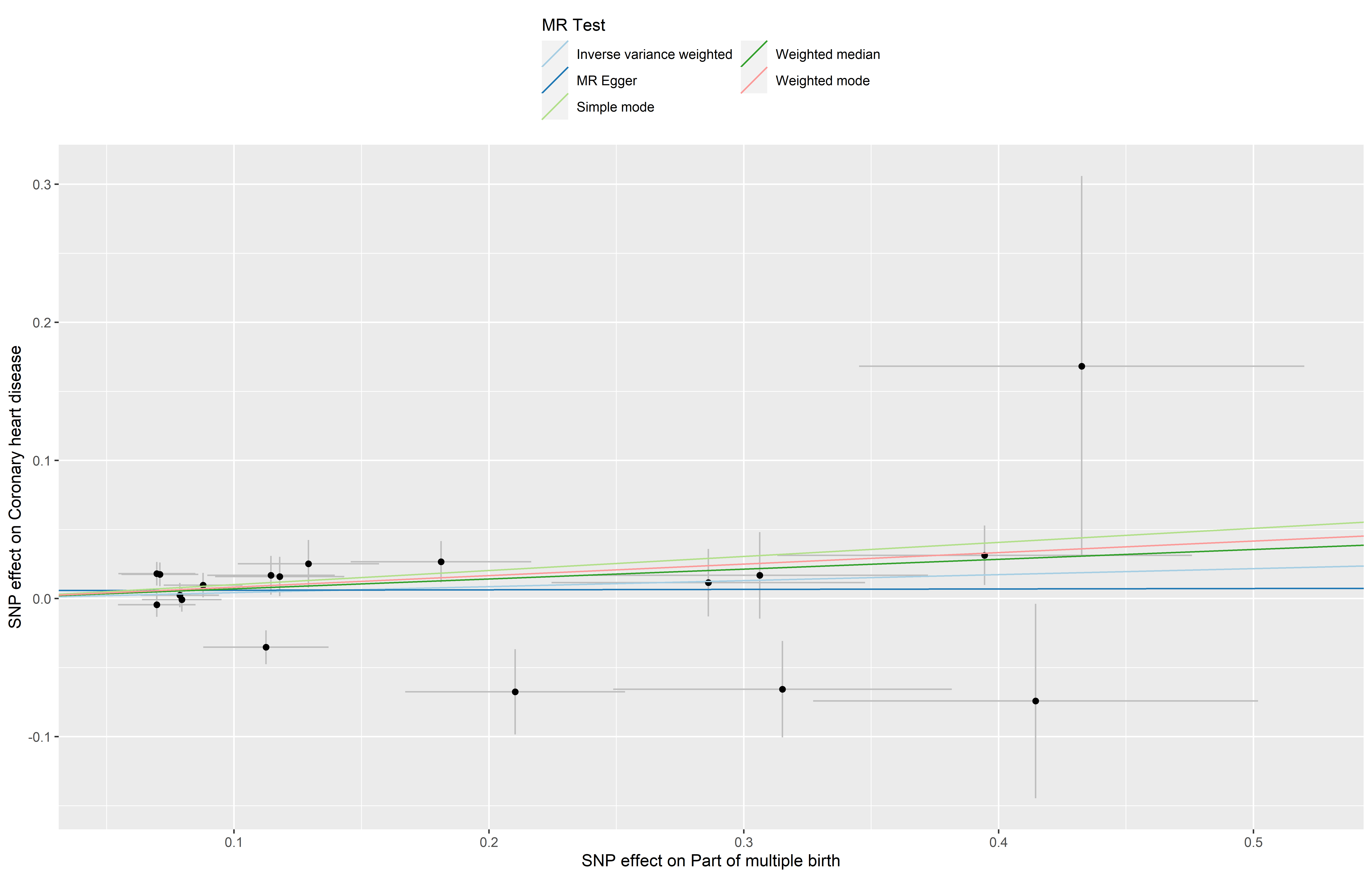


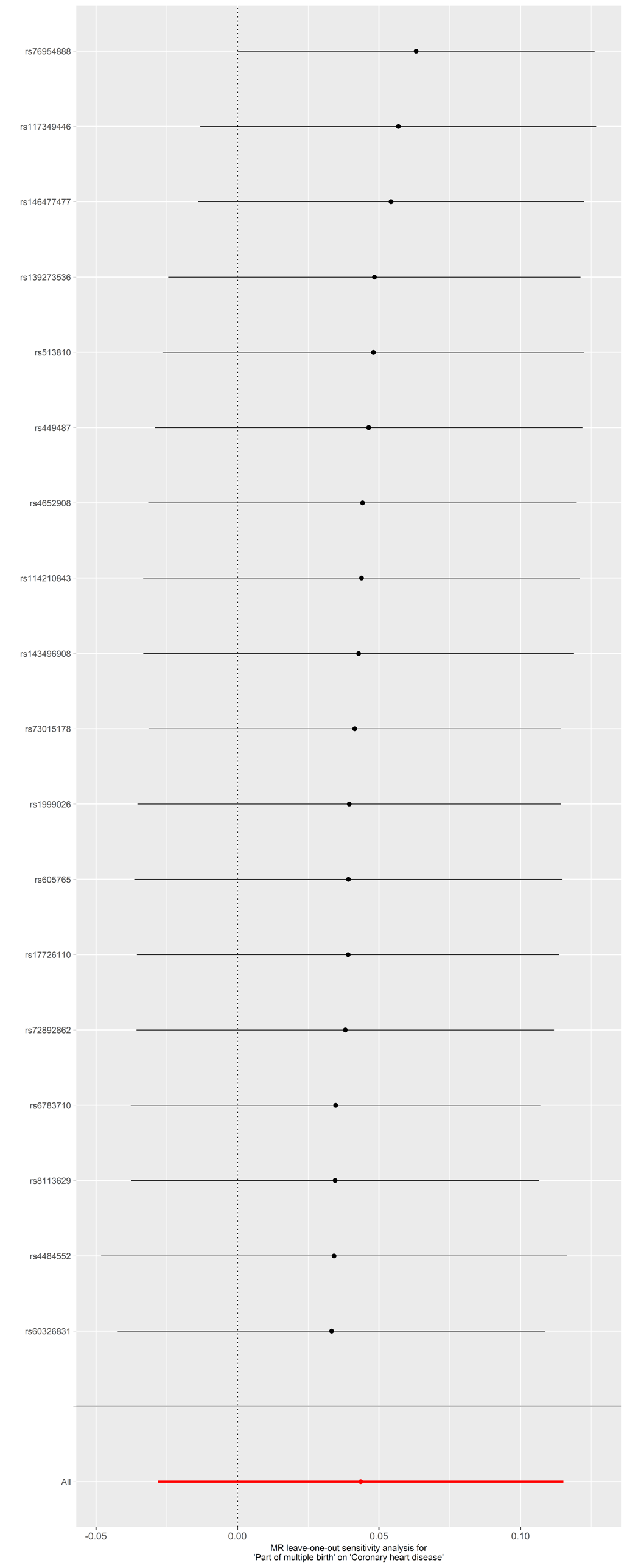


**Coronary heart disease – UK Biobank**


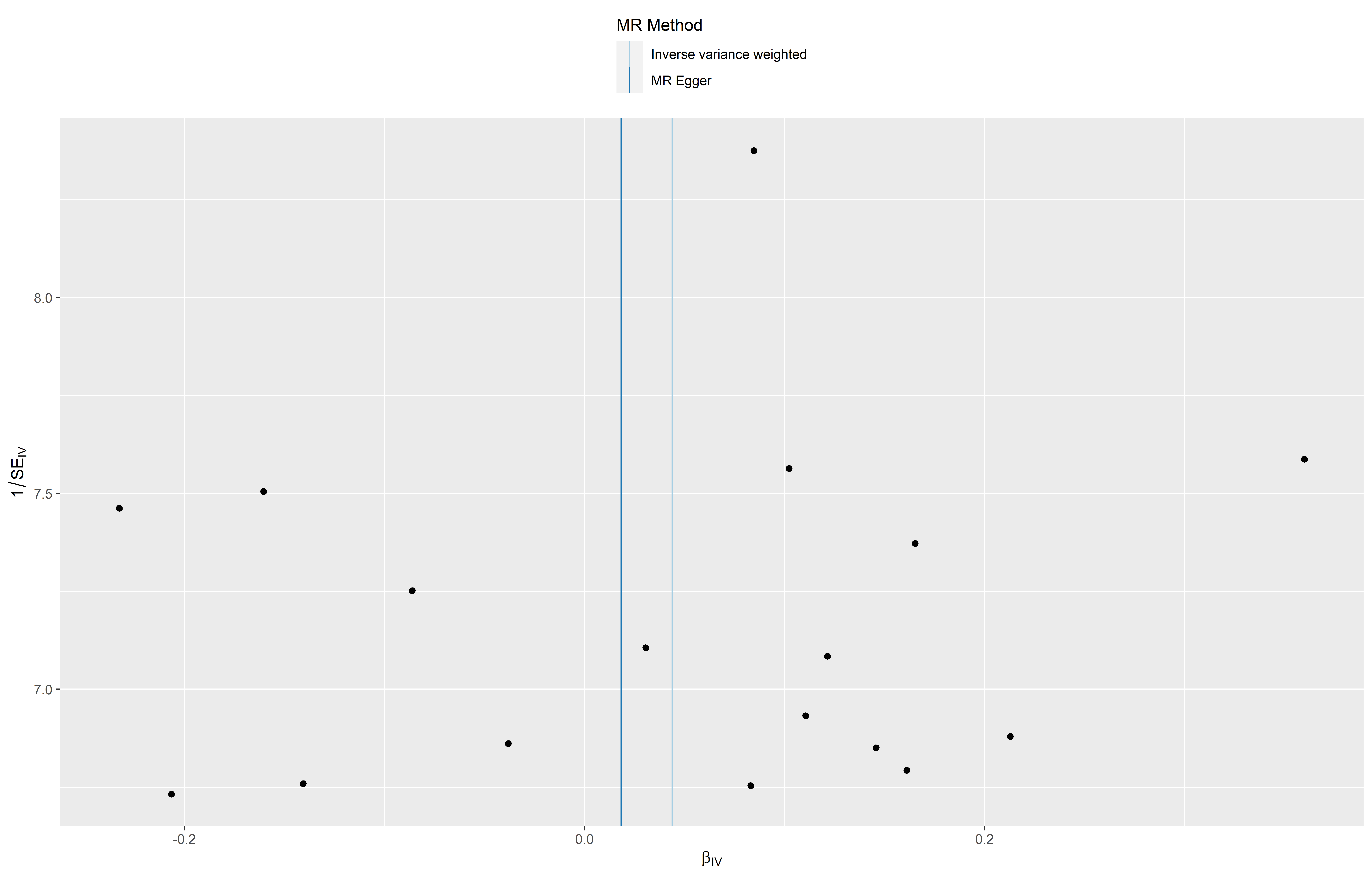

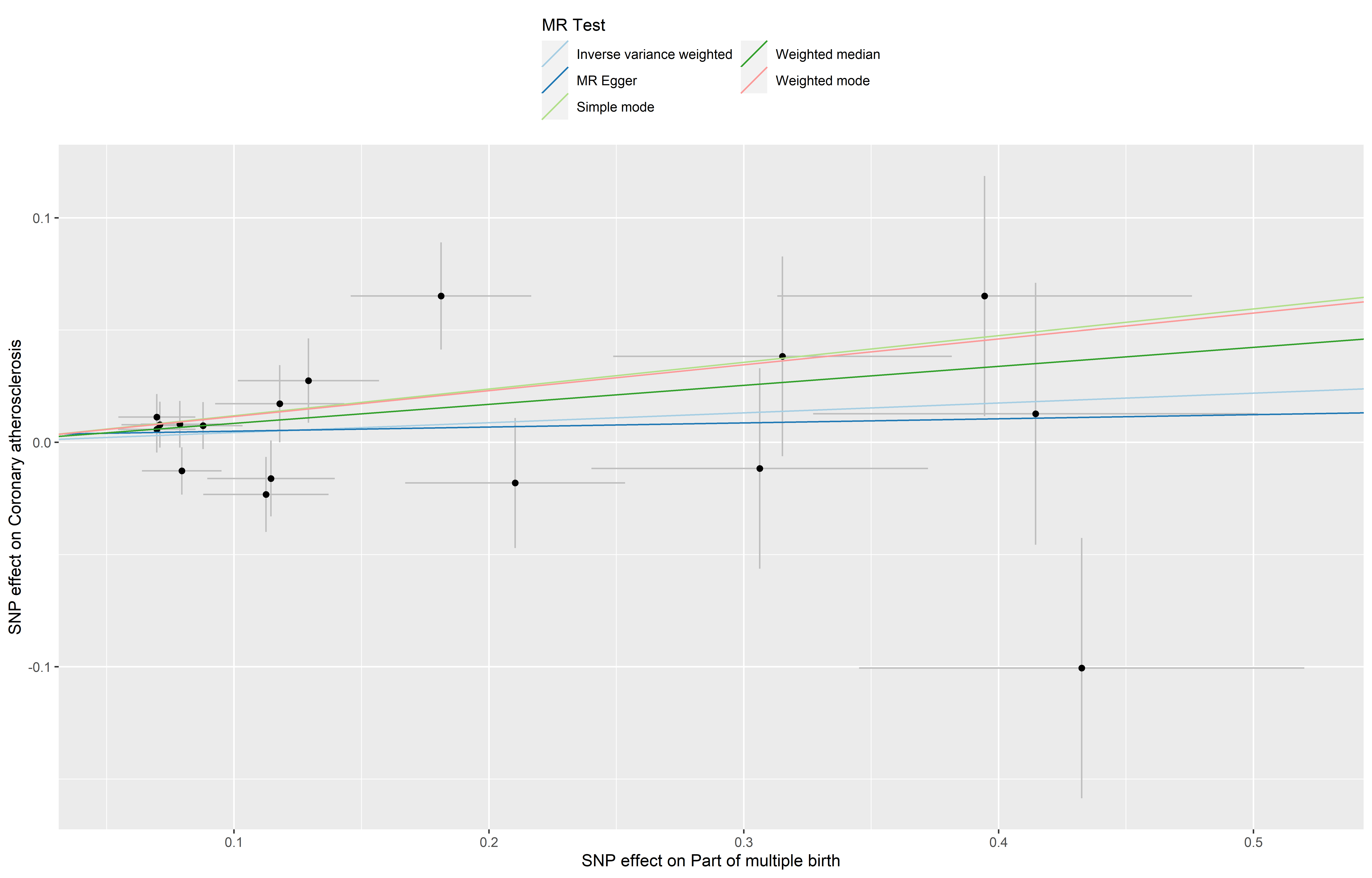


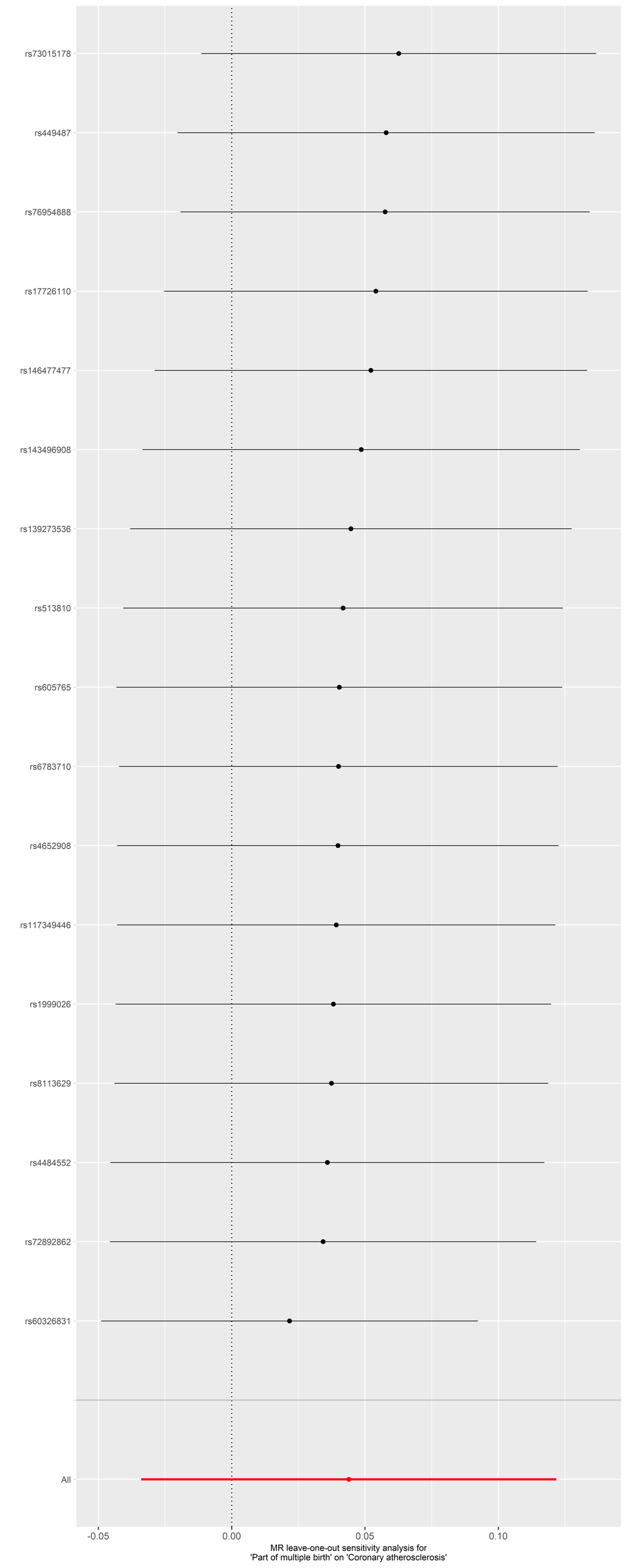


**Cardiomyopathy – Finngen**


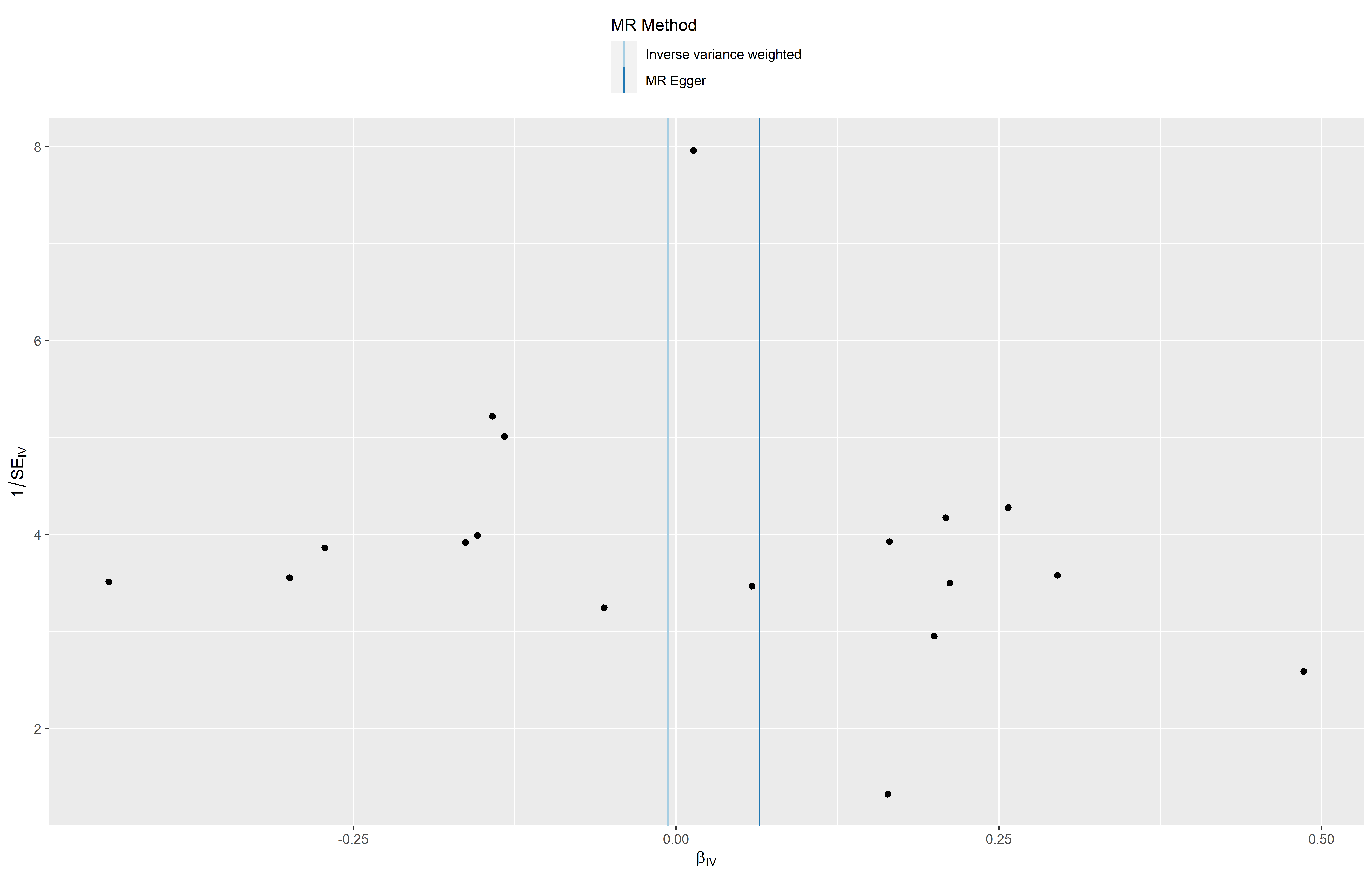

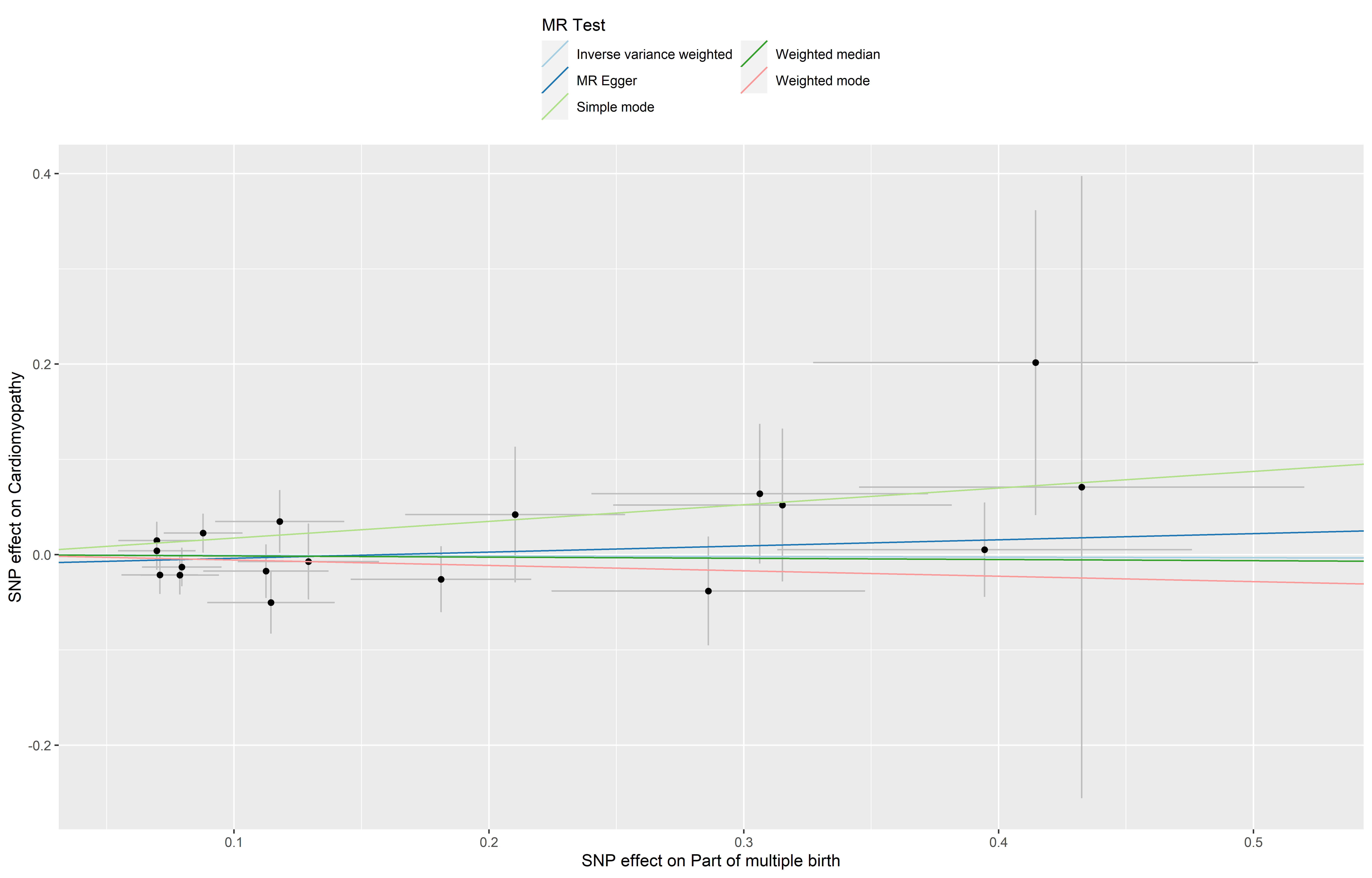


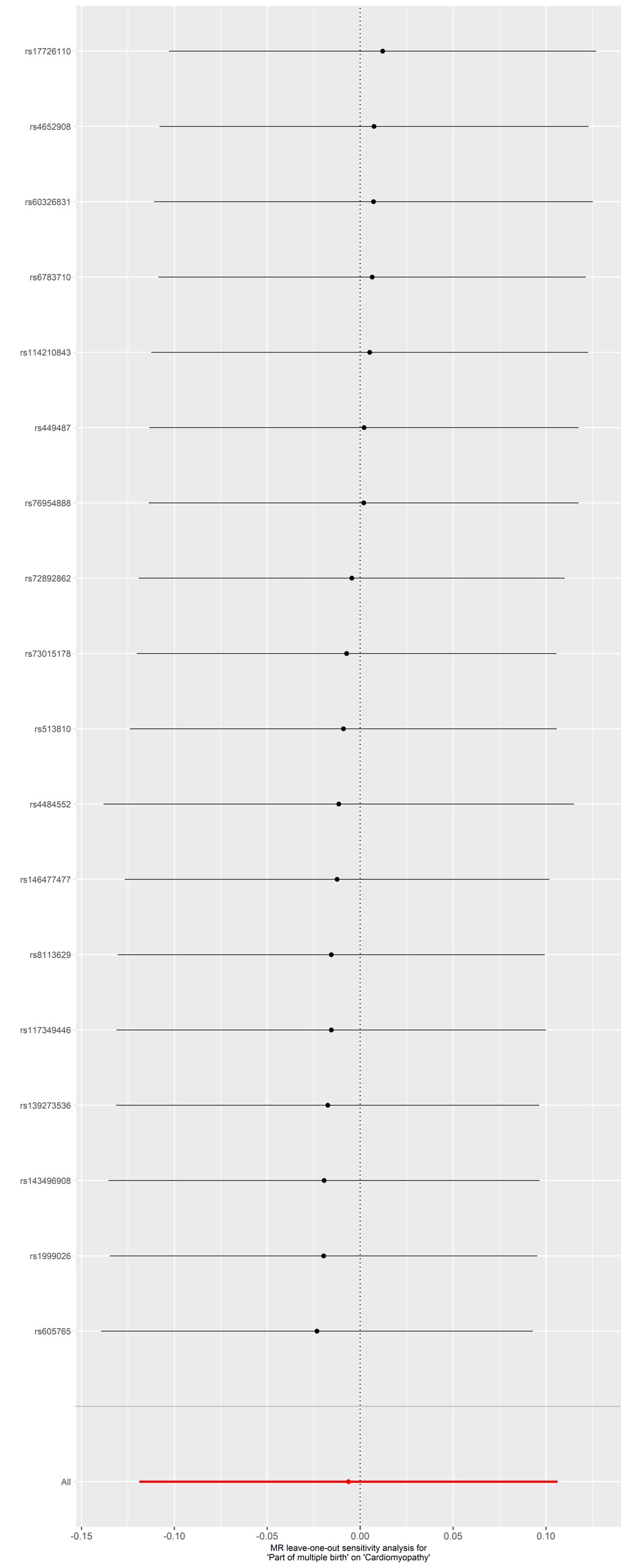


**Cardiomyopathy – UK Biobank**


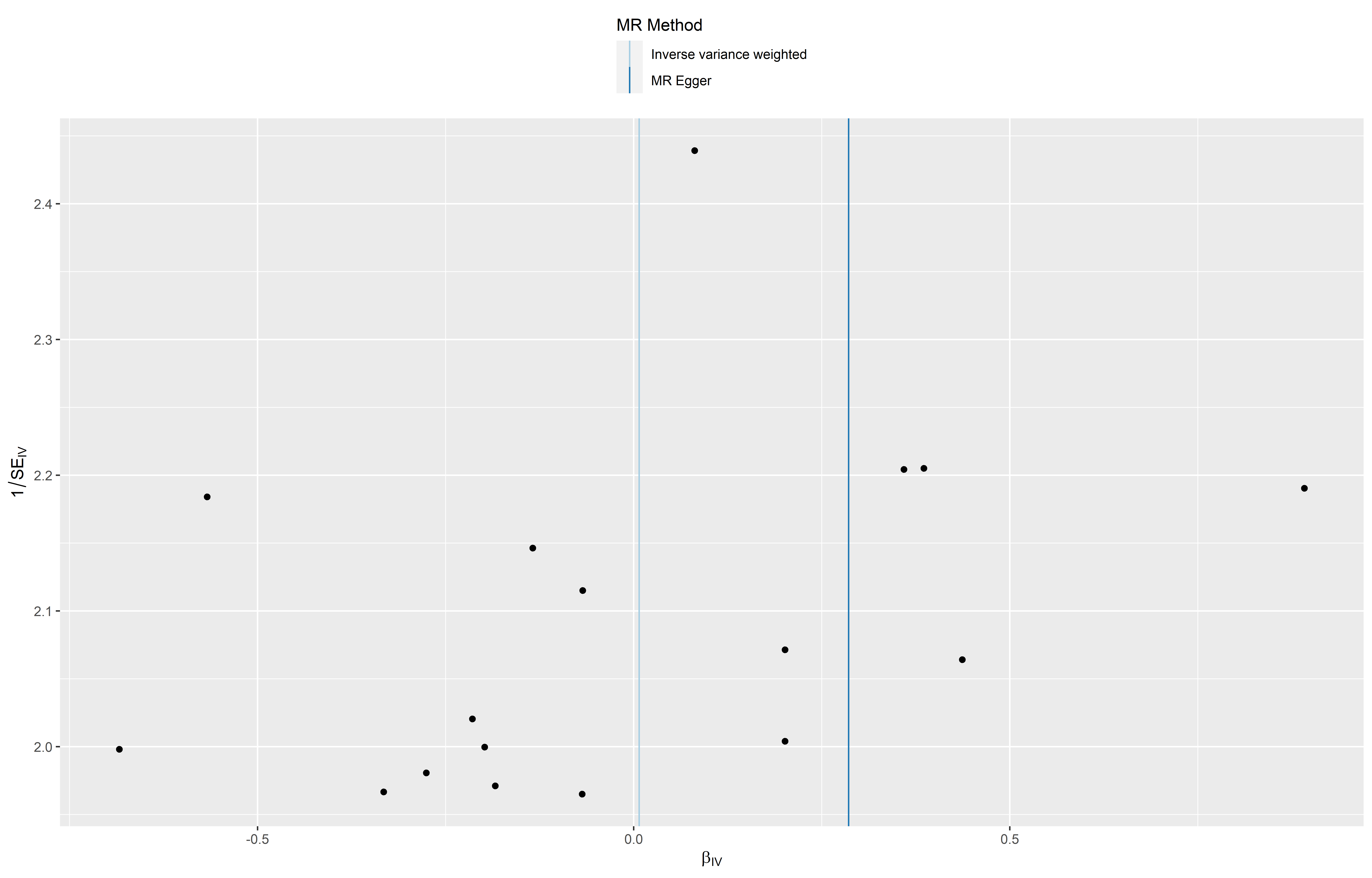

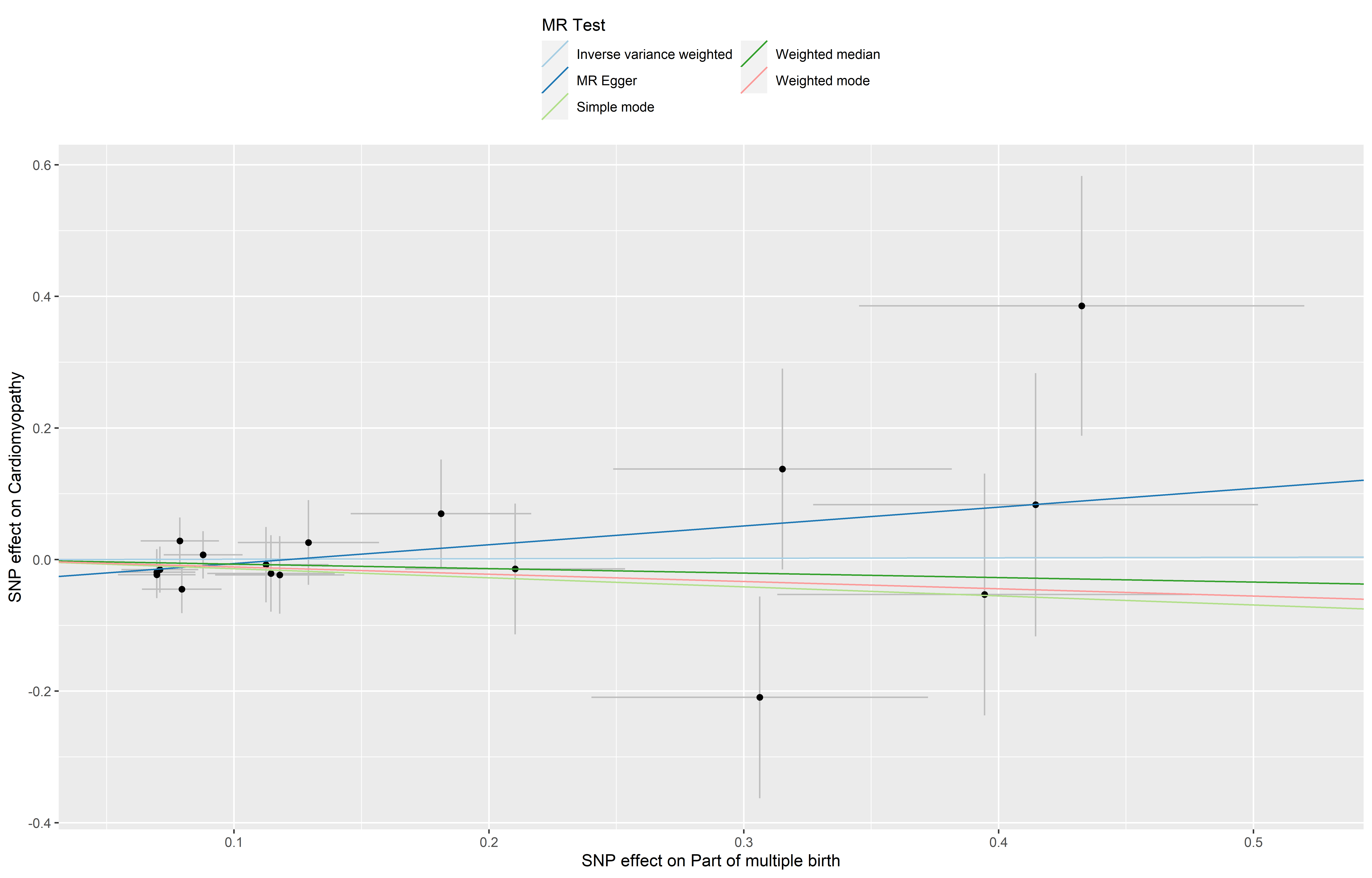


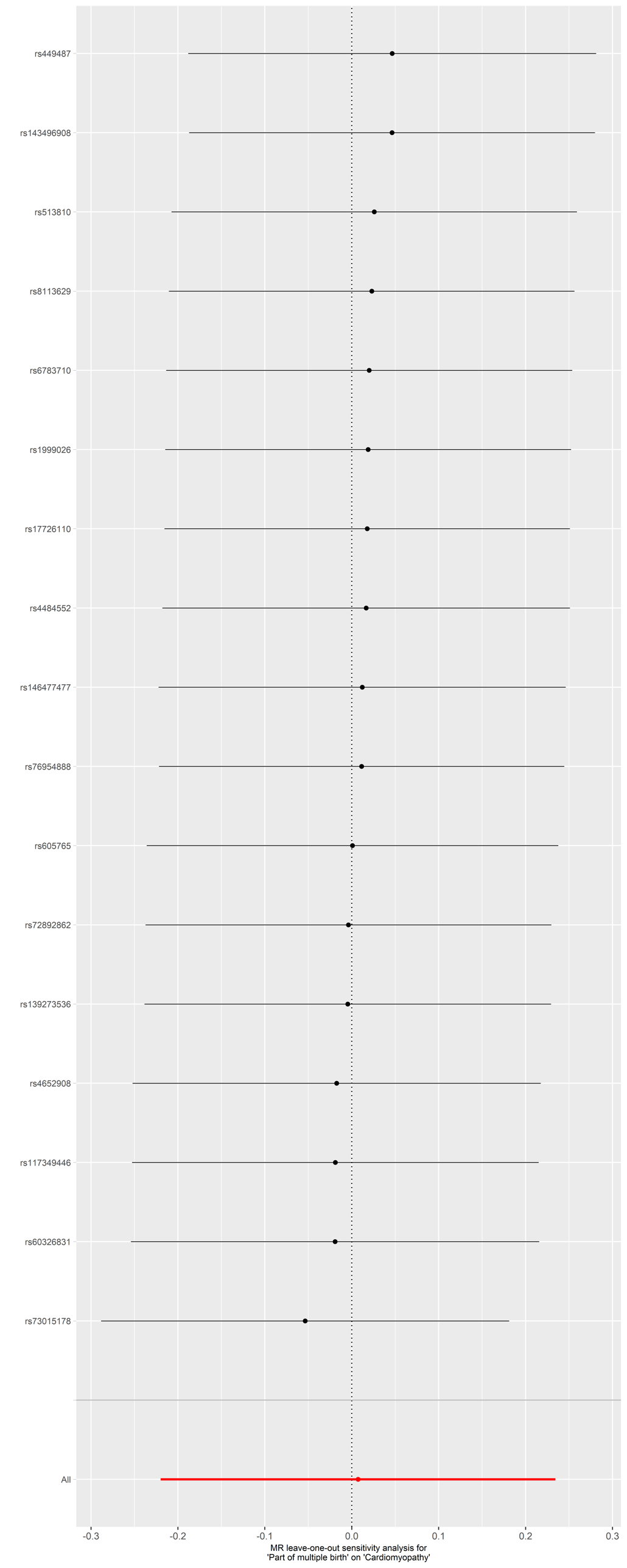


**Myocardial infarction – Finngen**


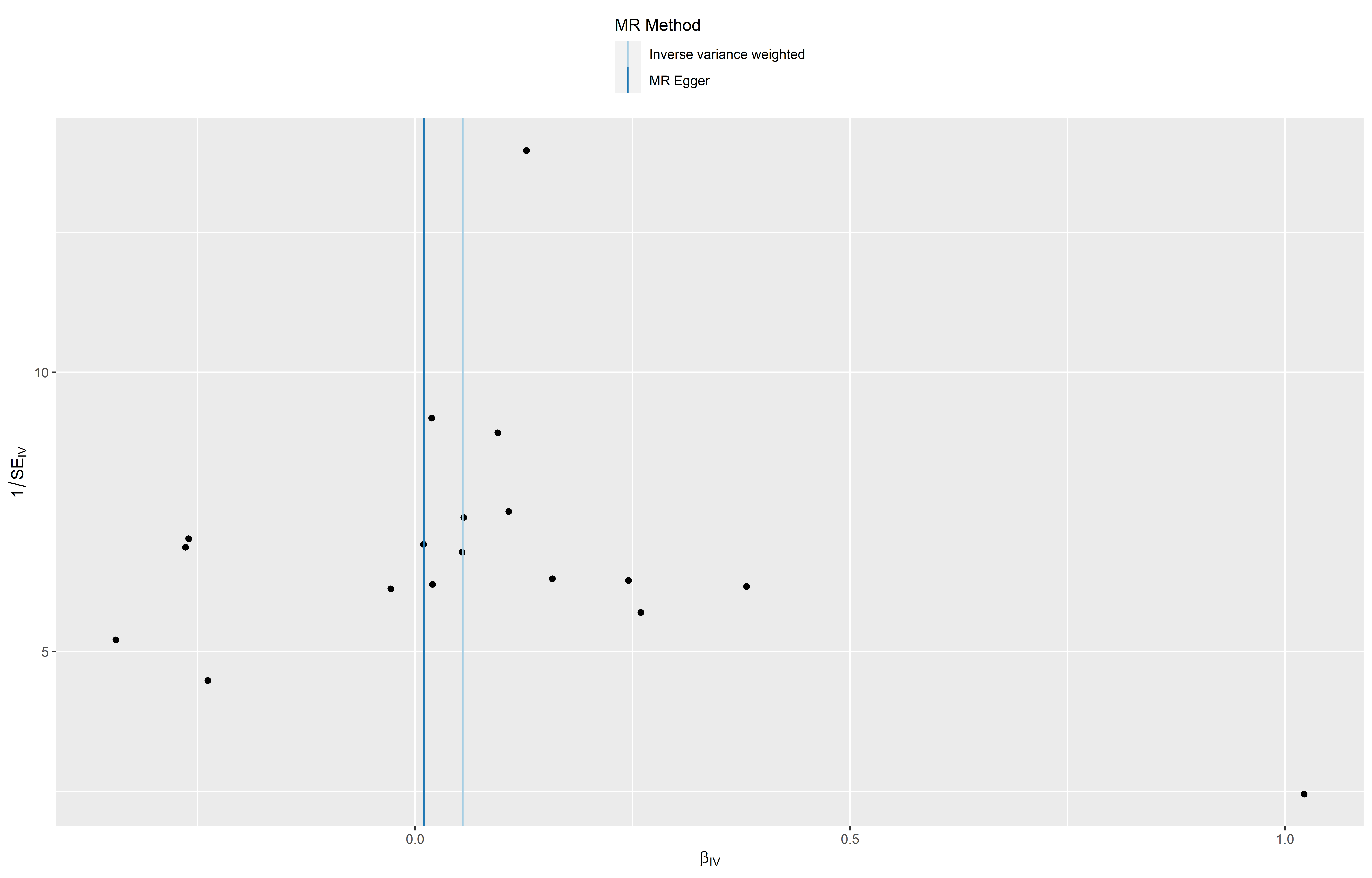

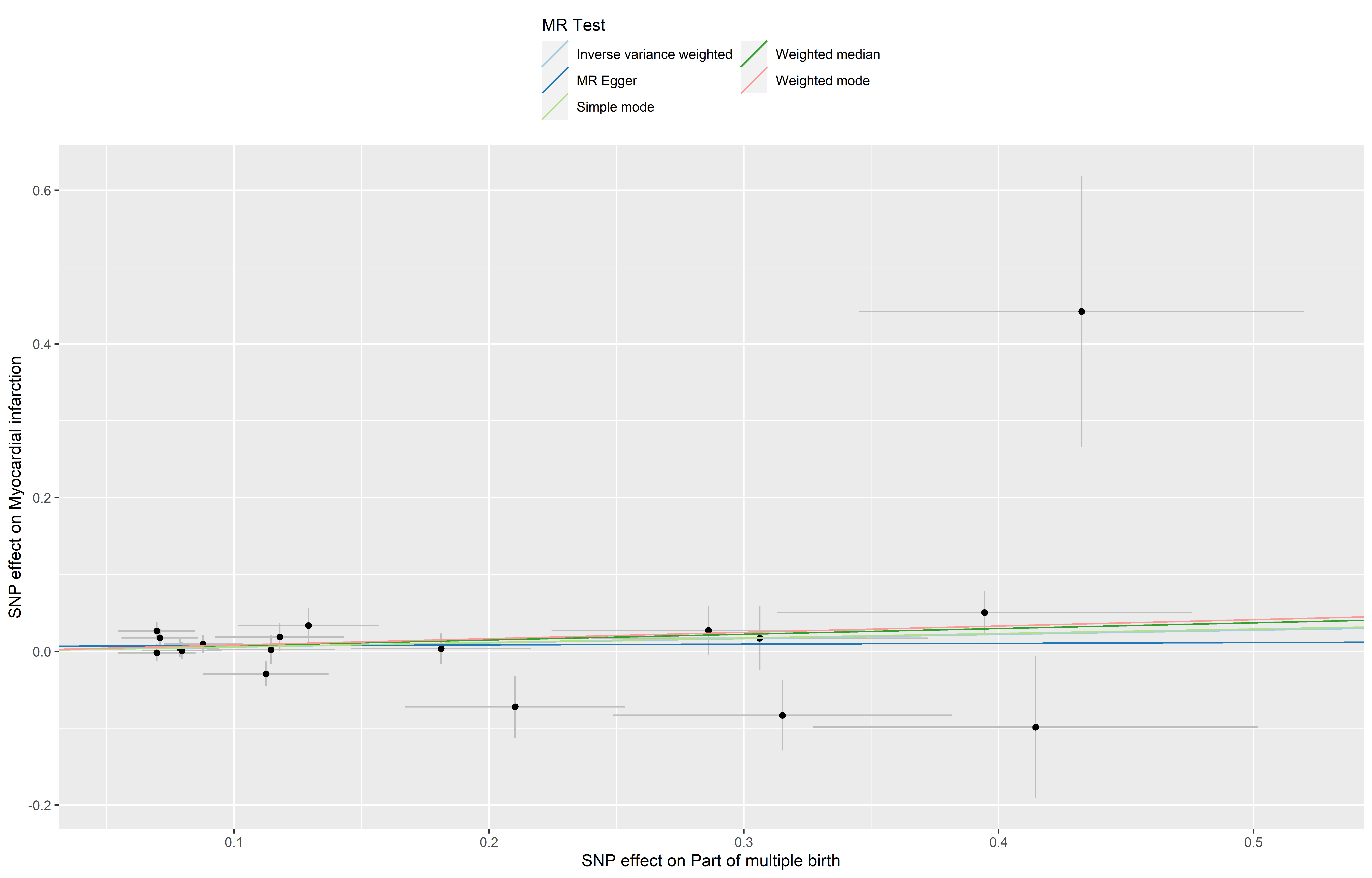


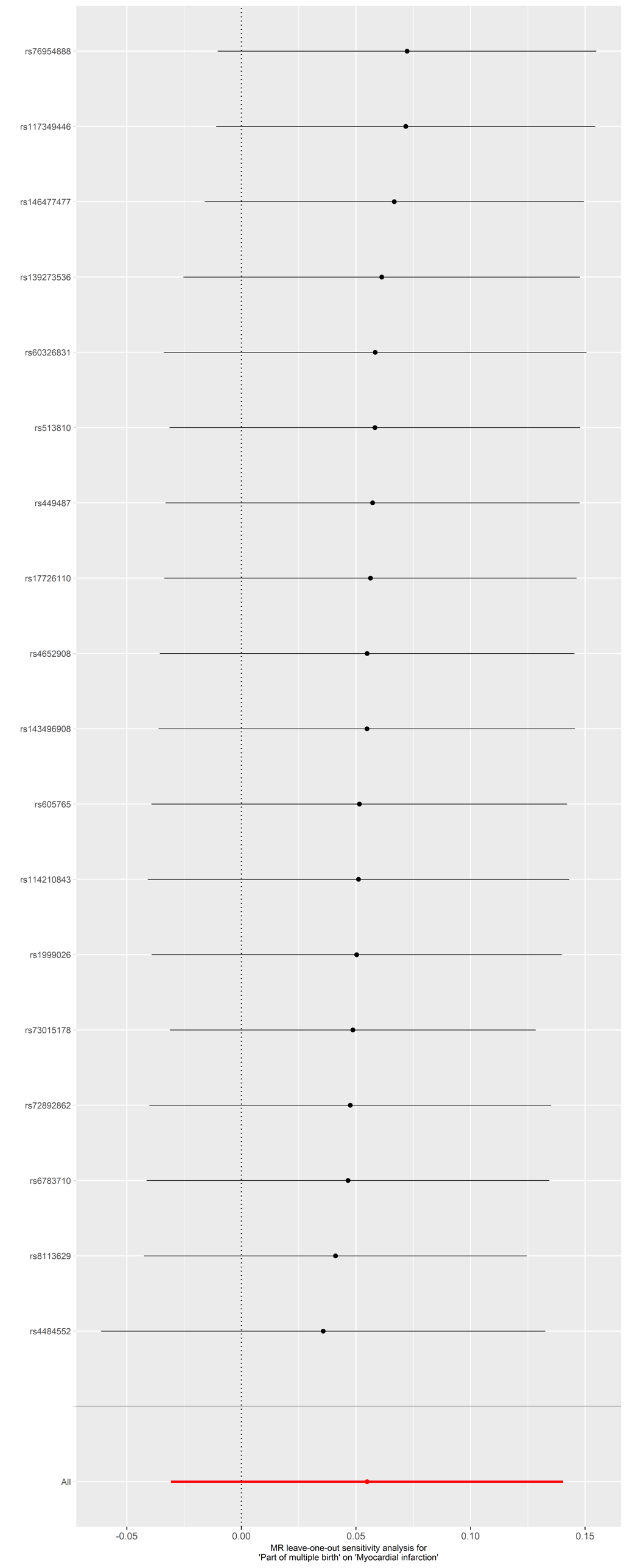


**Myocardial infarction – UK Biobank**


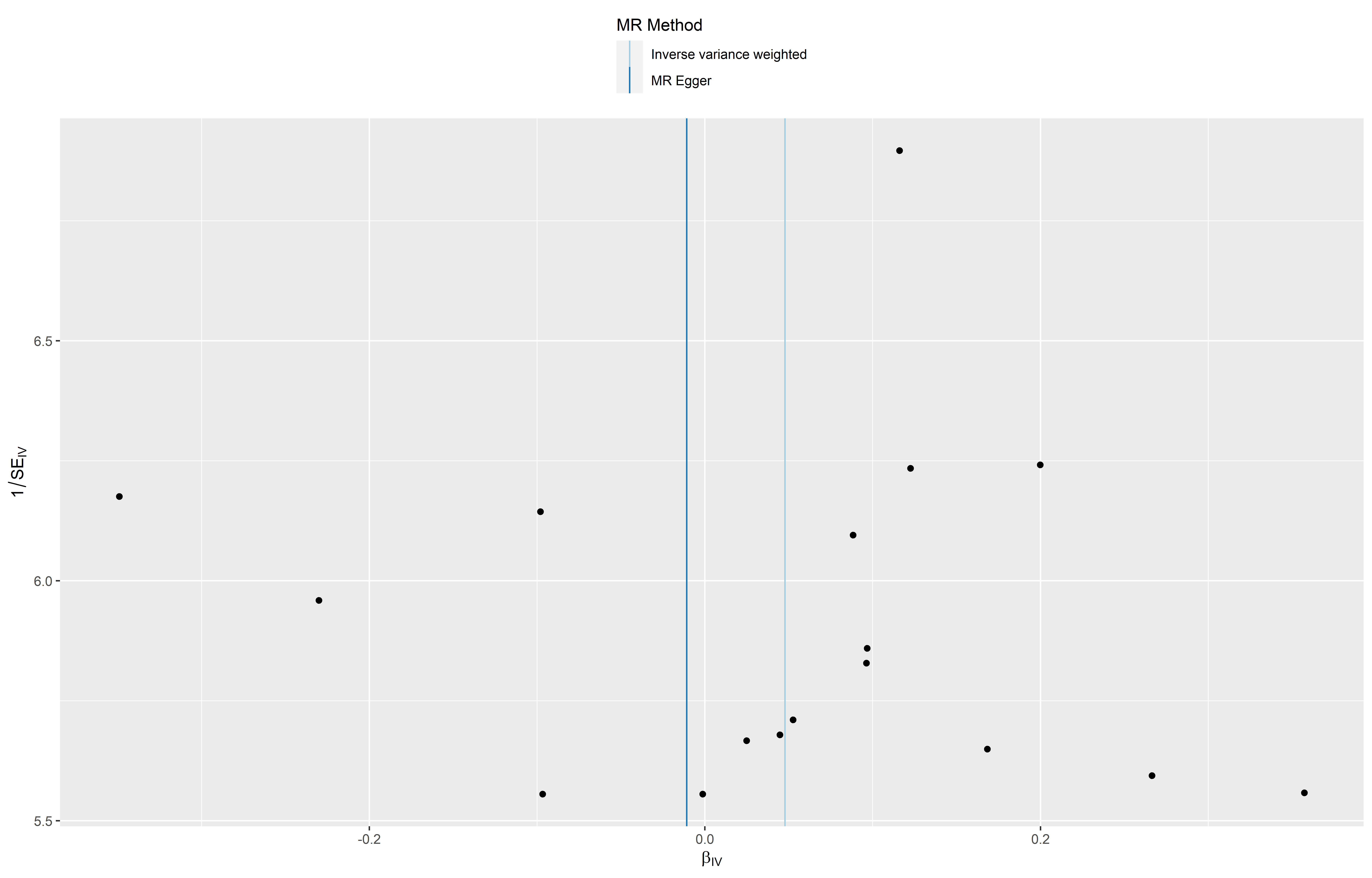

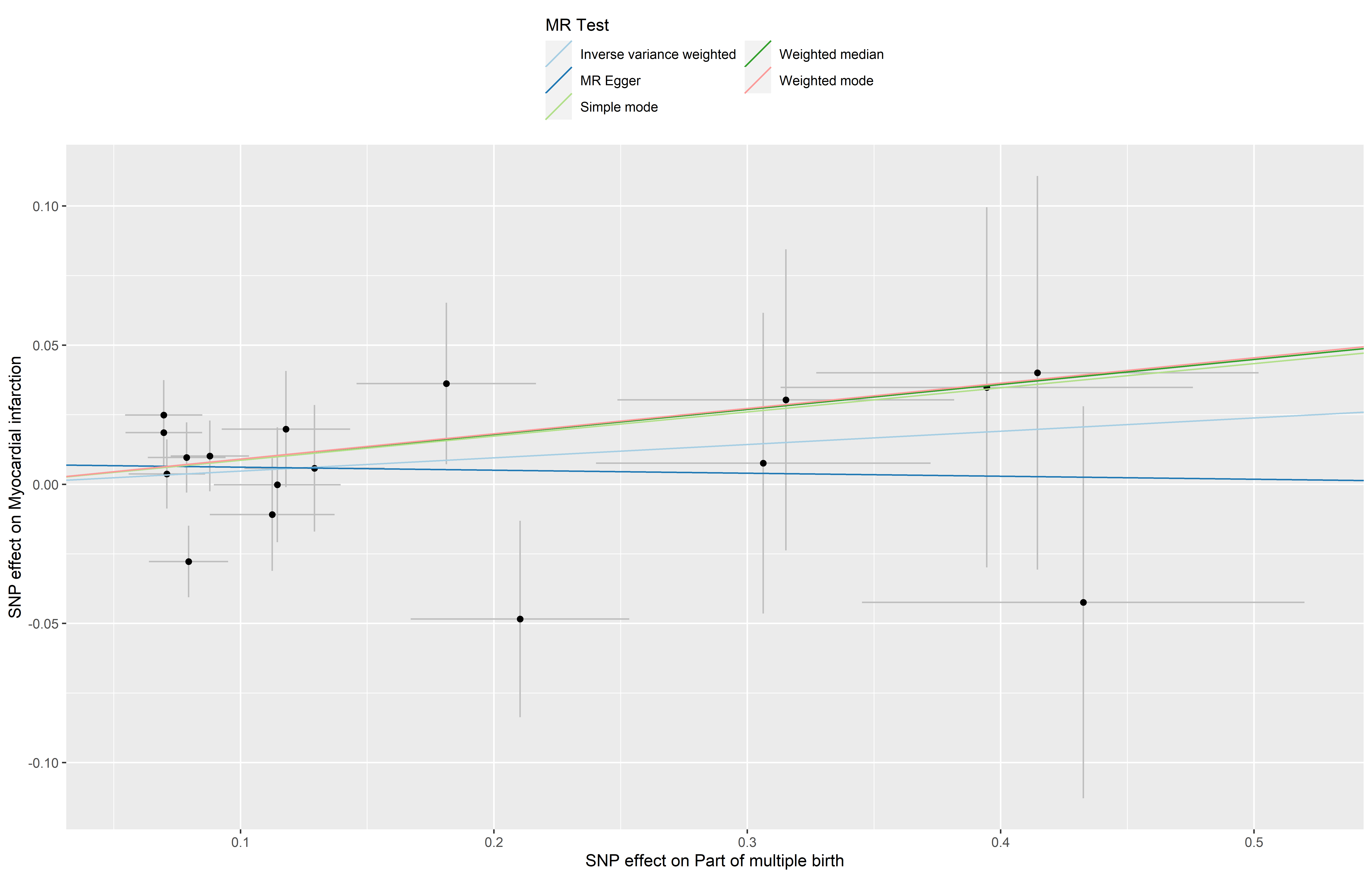


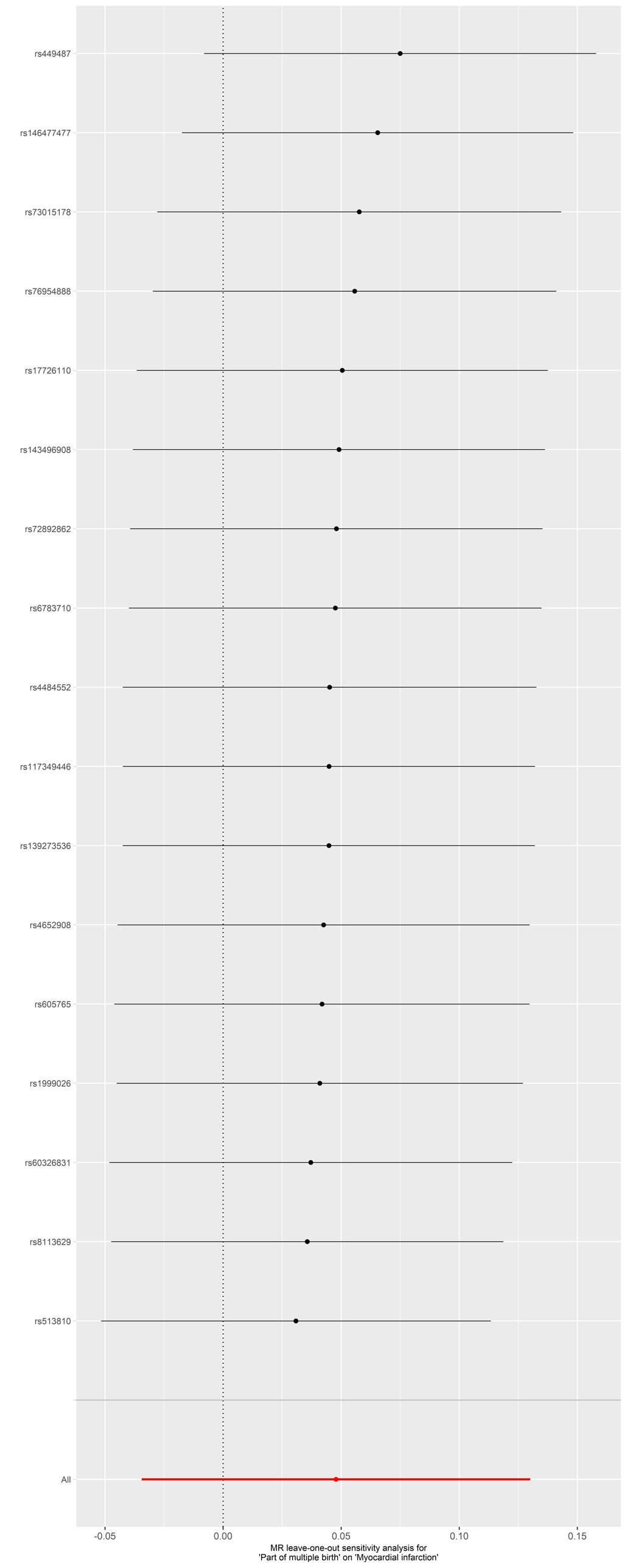


**Pulmonary embolism – Finngen**


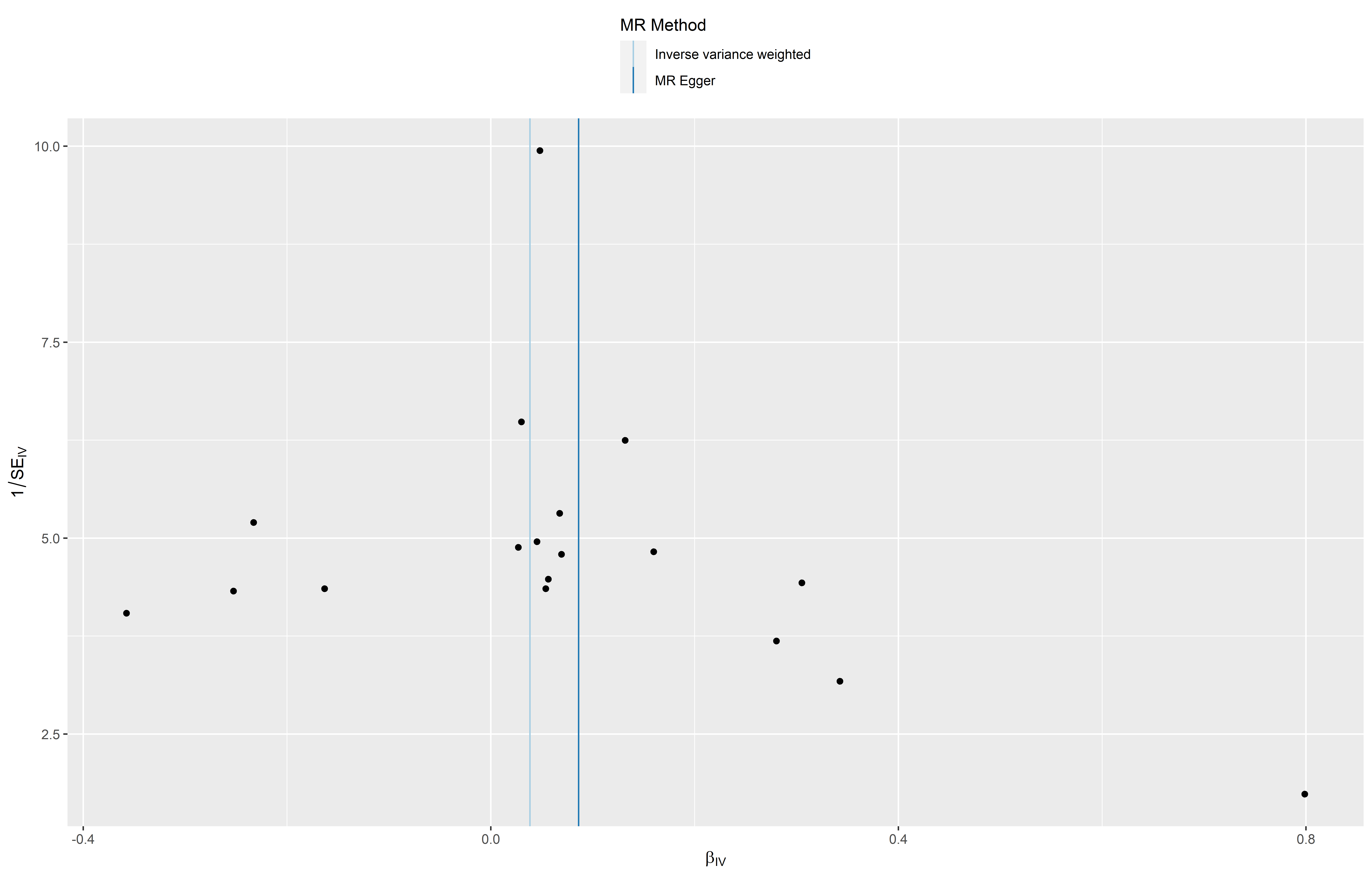

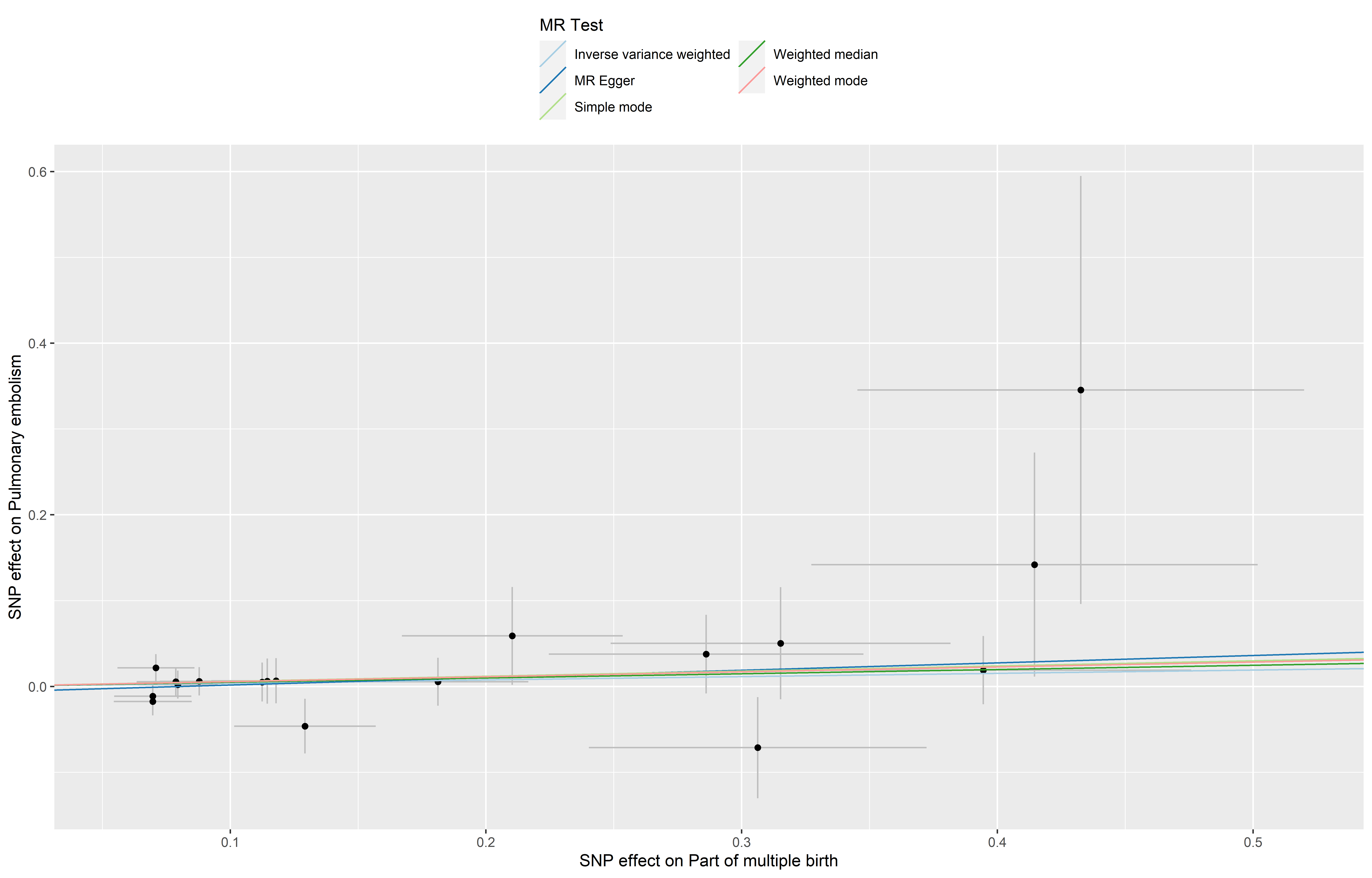


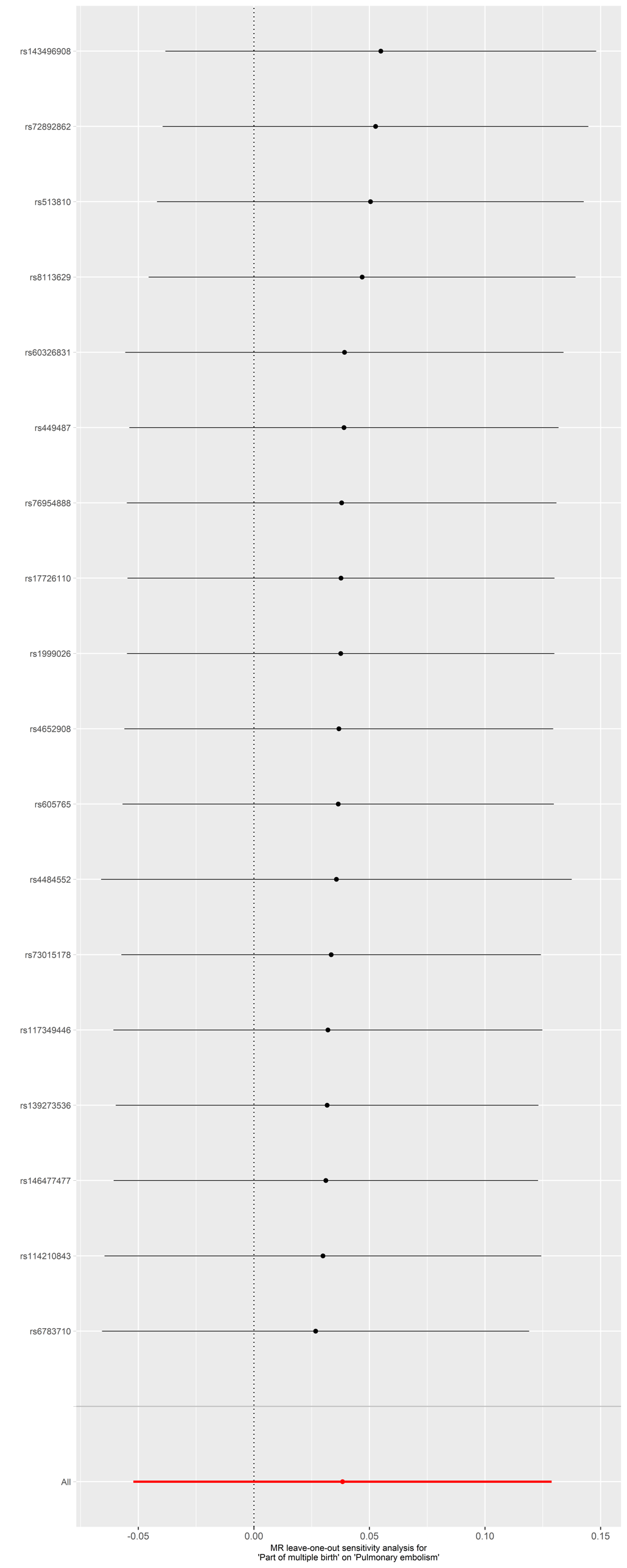


**Pulmonary embolism – UK Biobank**


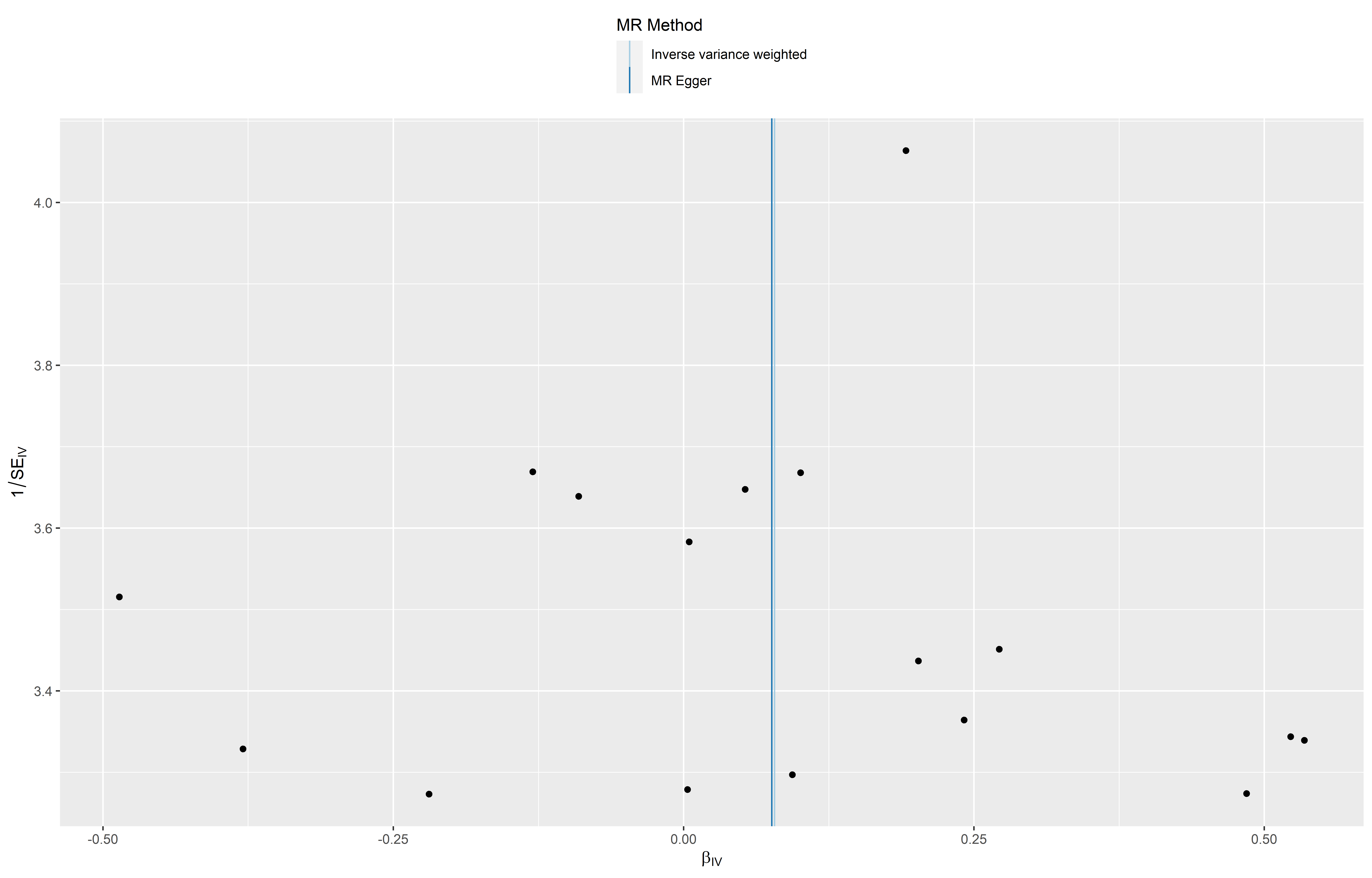

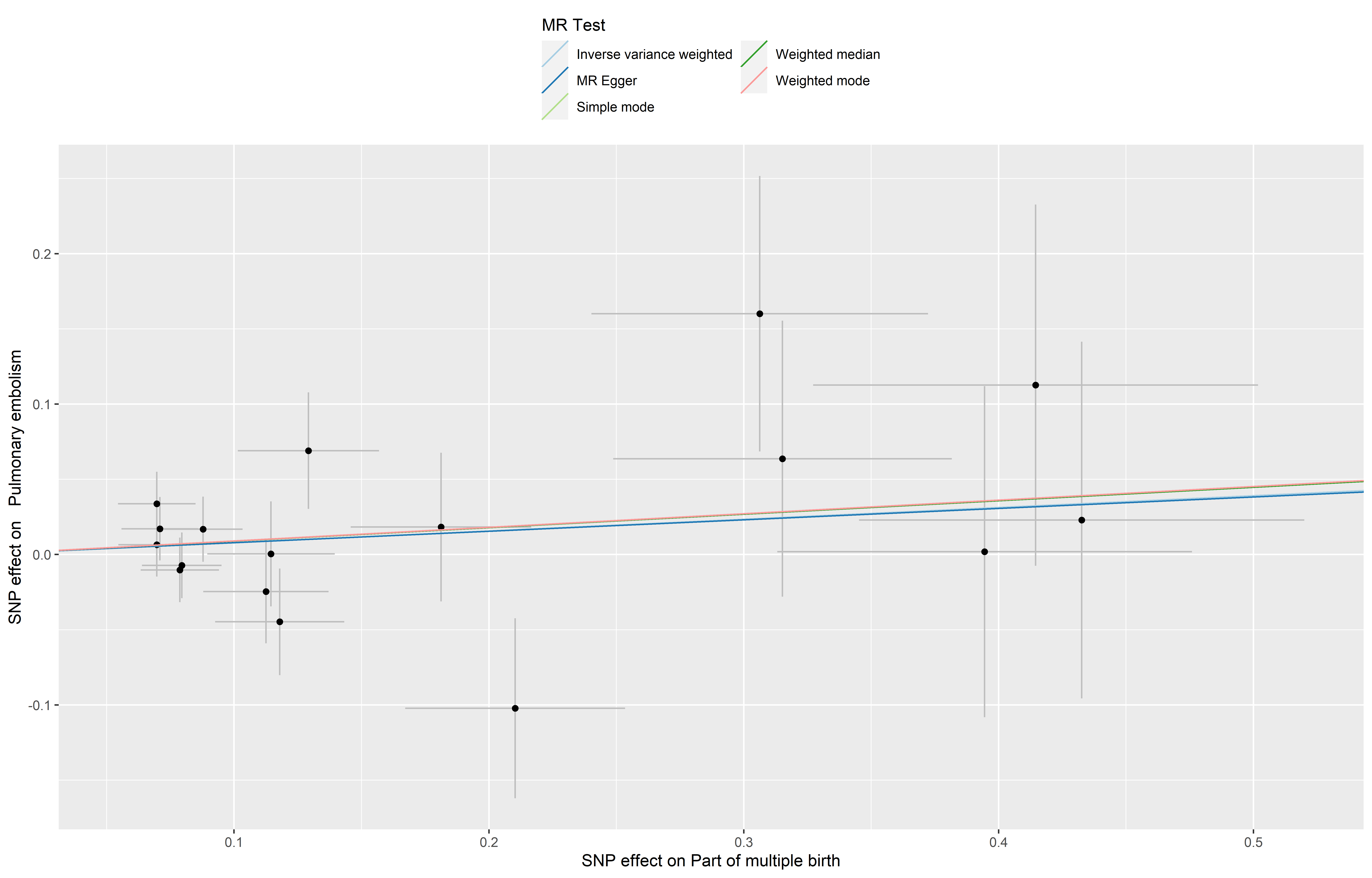


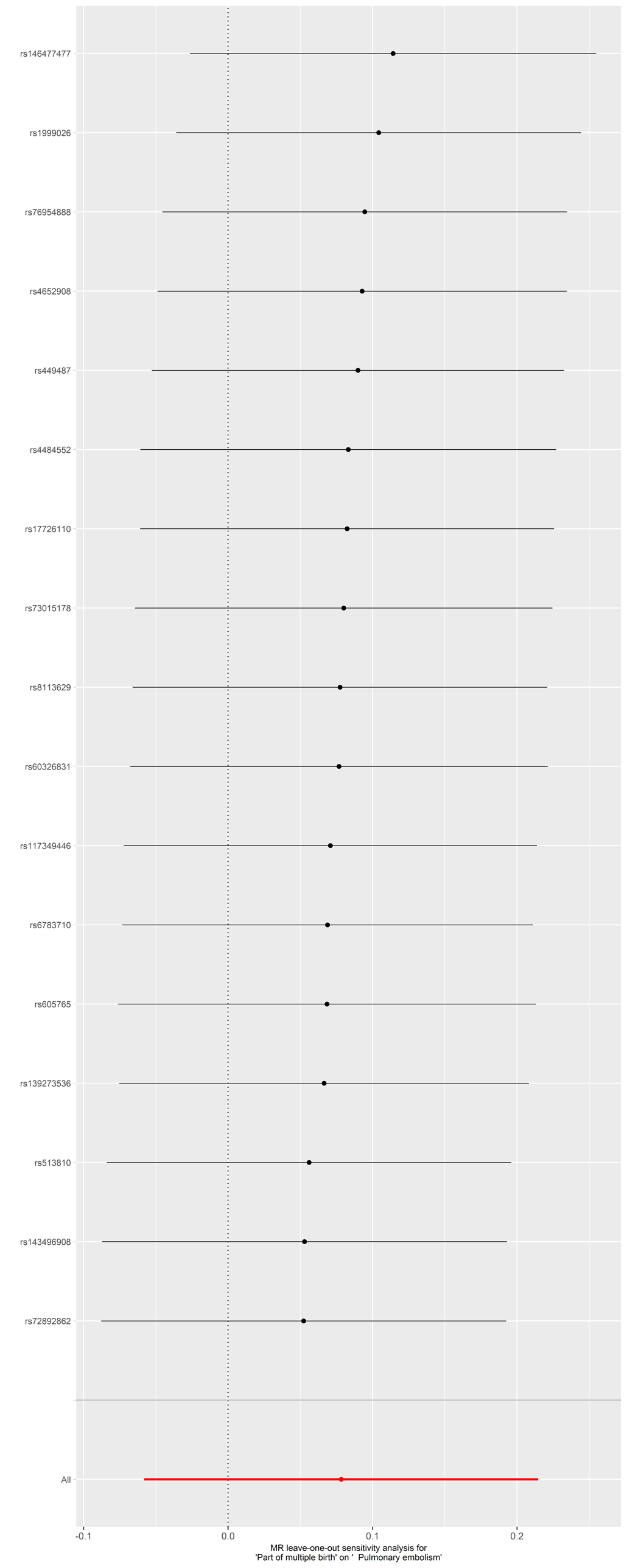


**Deep vein thrombosis – Finngen**


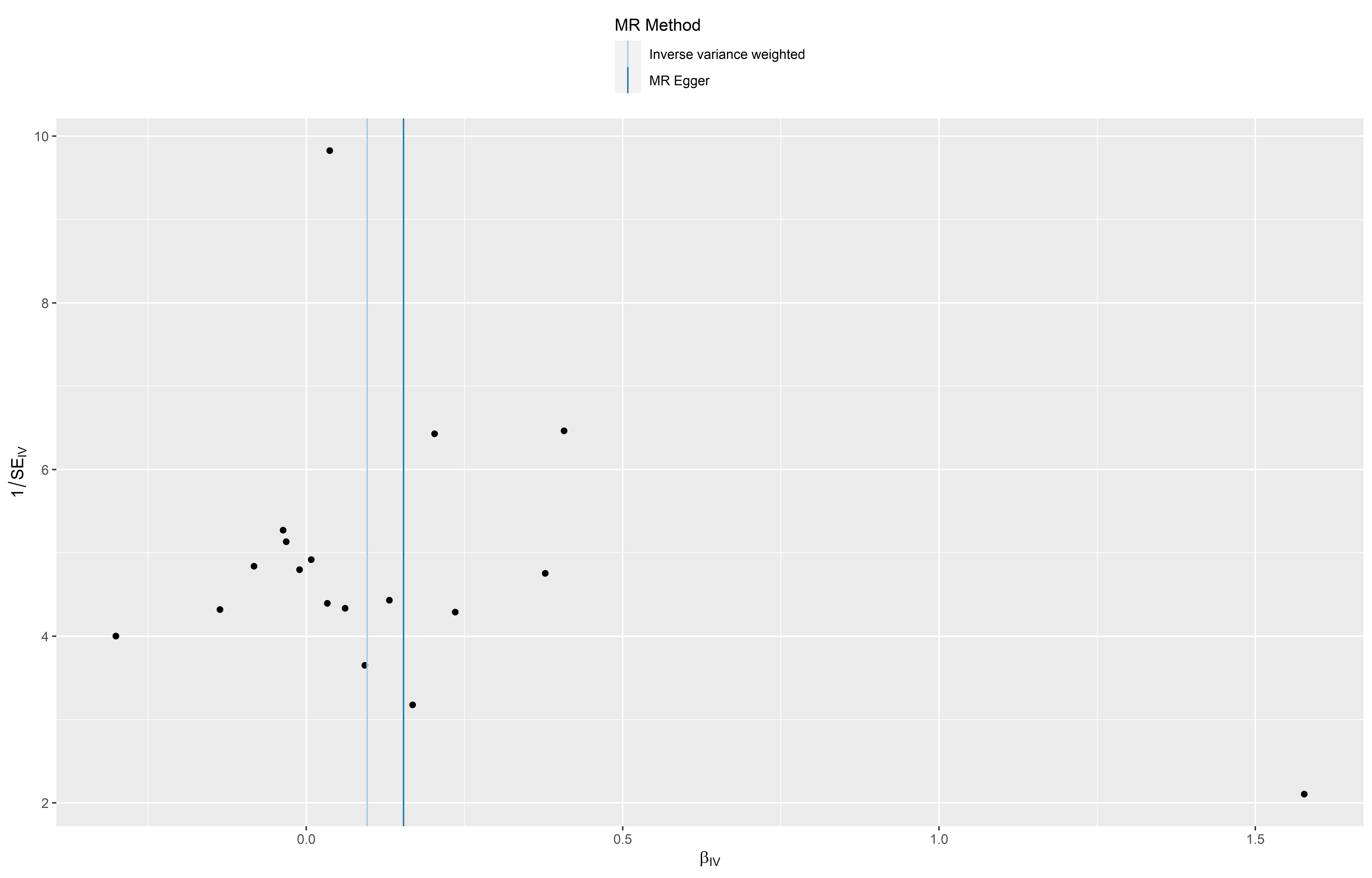

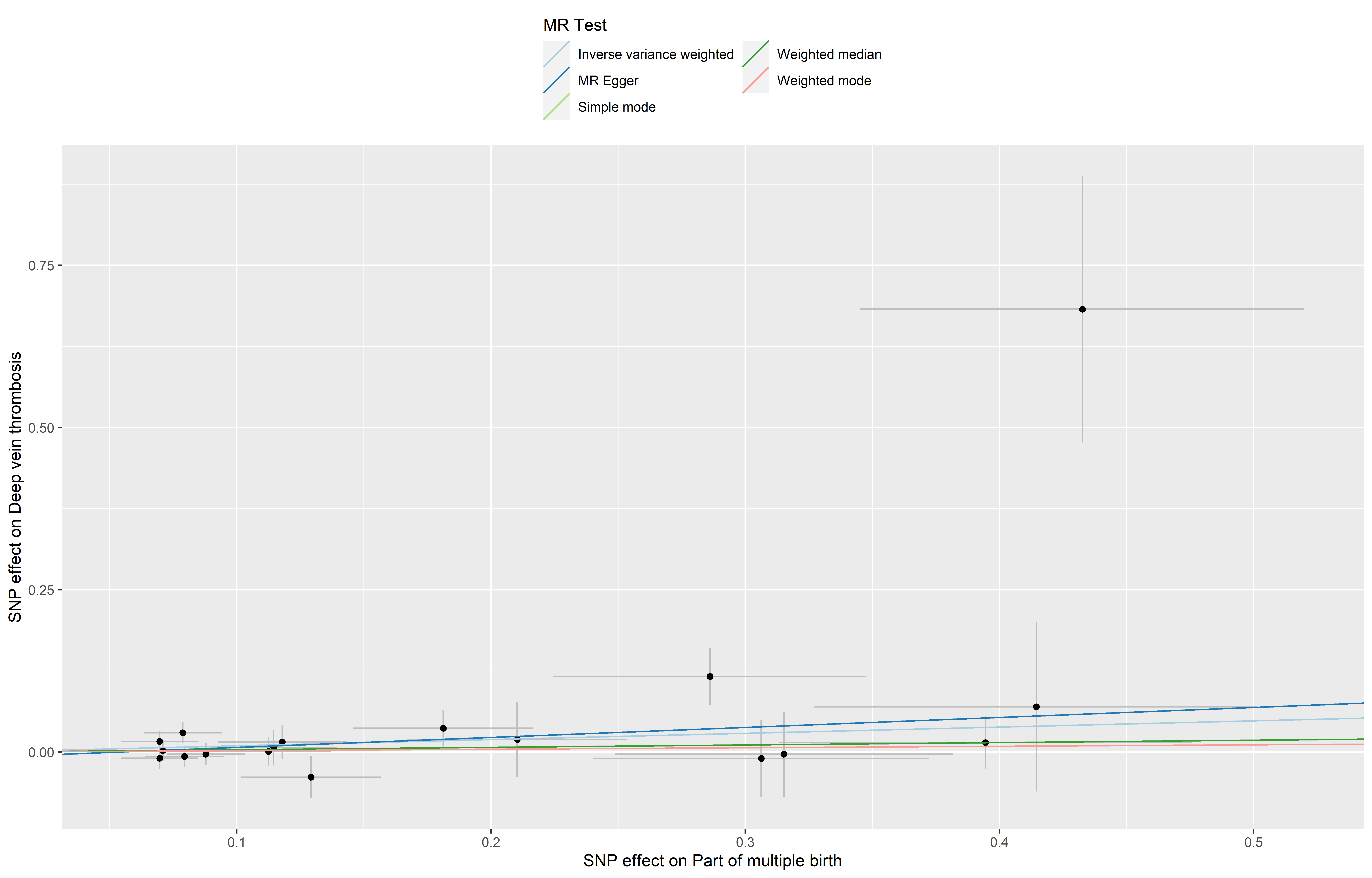


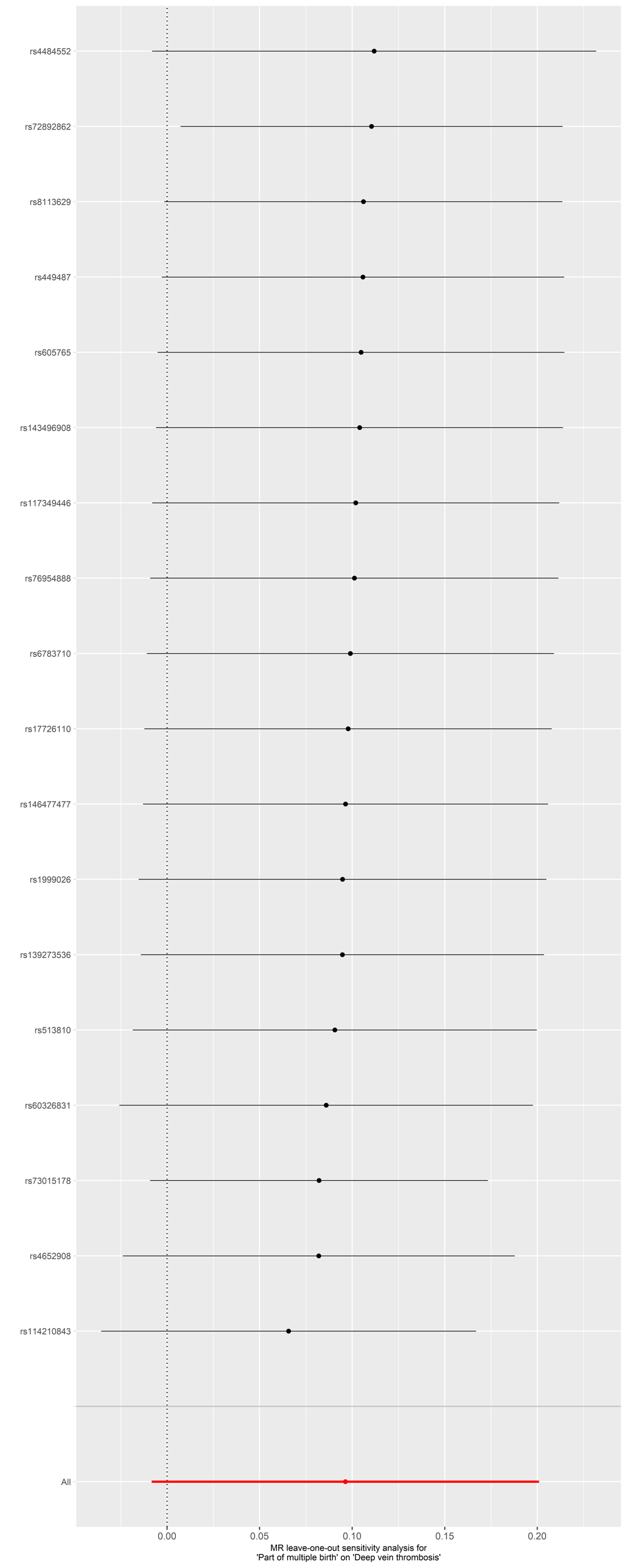


**Deep vein thrombosis – UK Biobank**


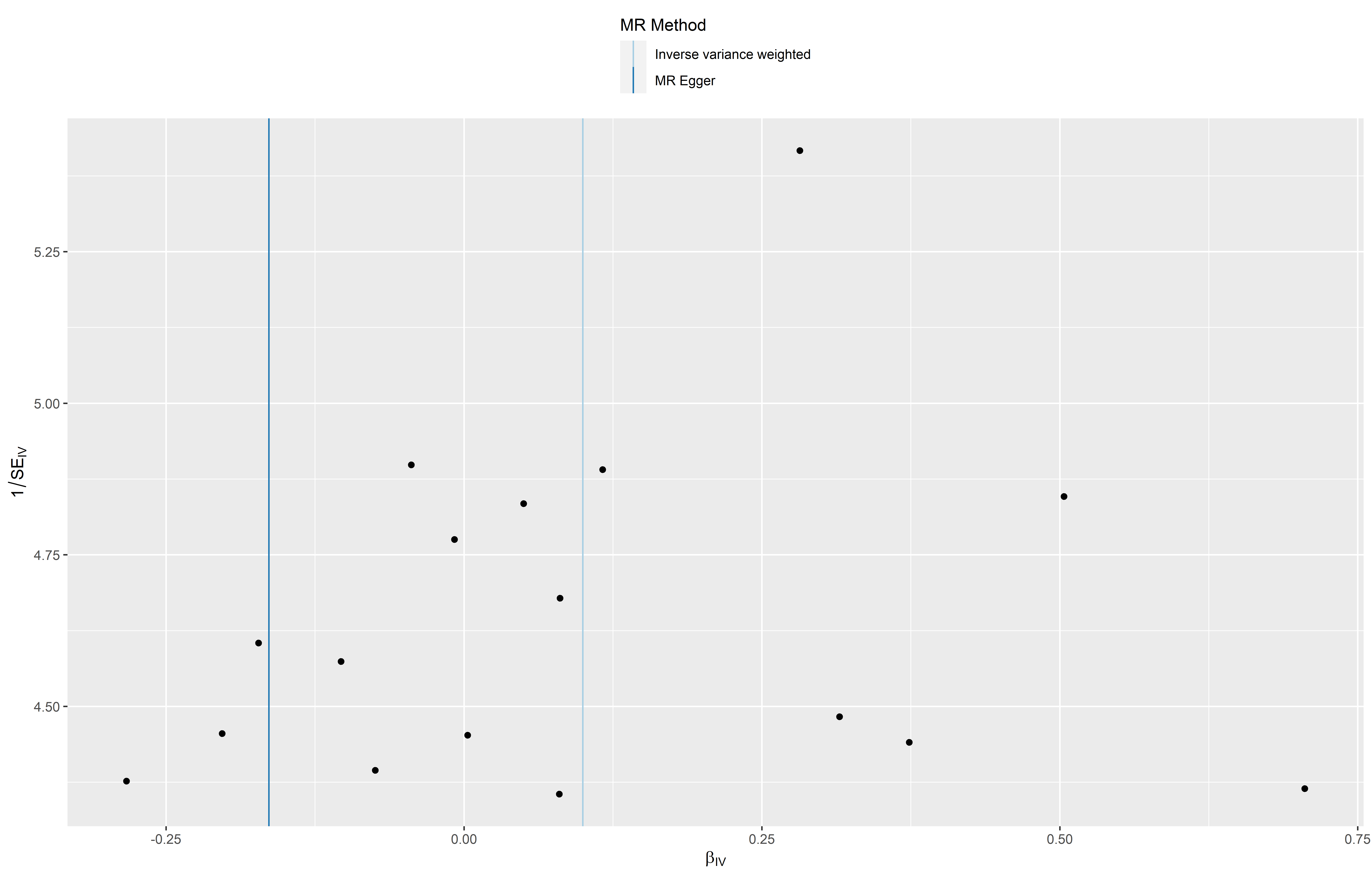

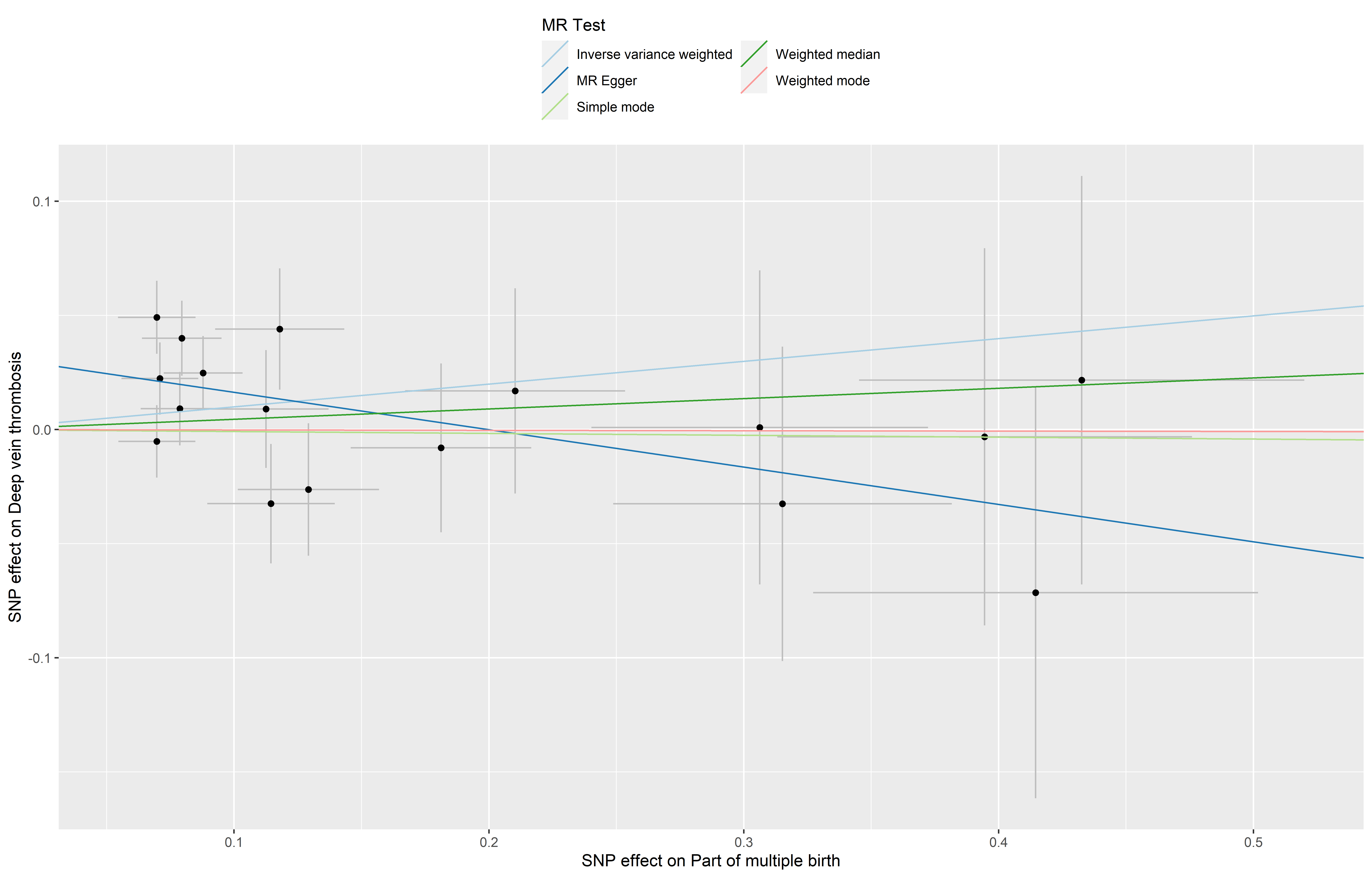


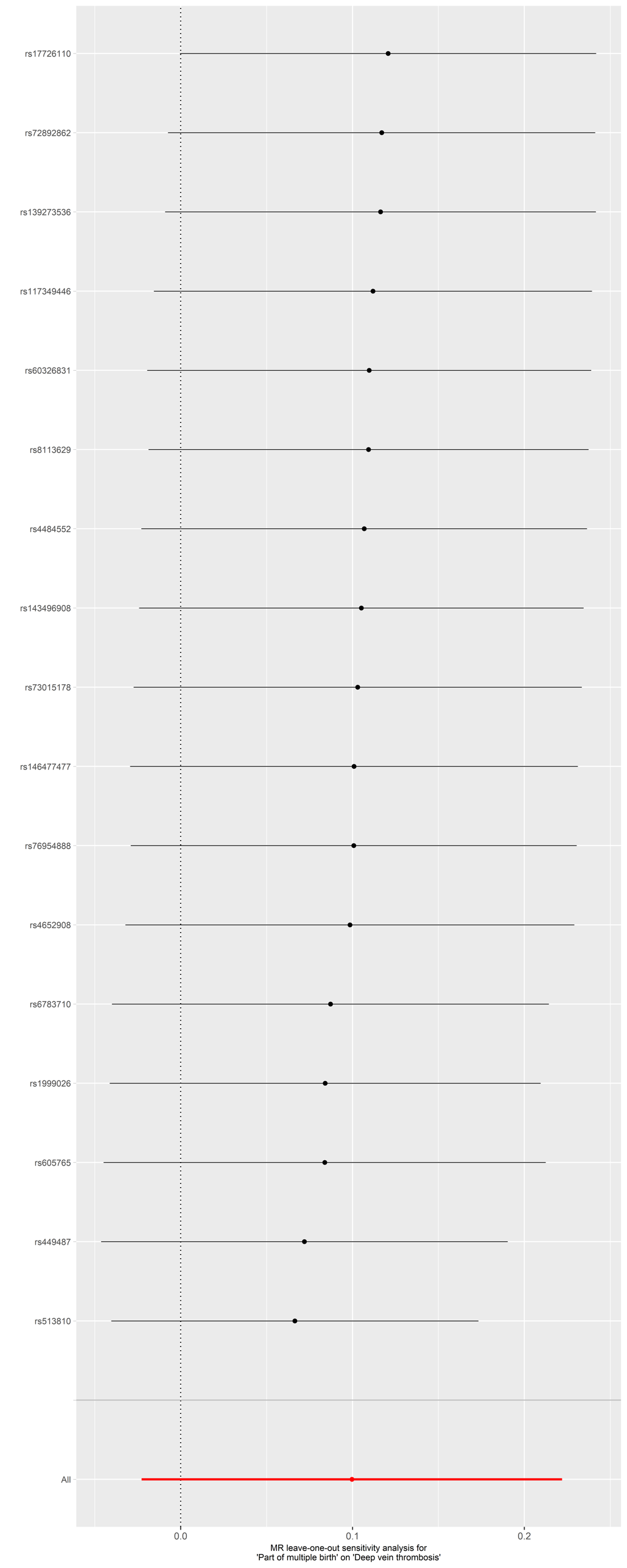

Supplement: Supplementary file 11 — Additional file 11: Material S3. The scatter plot, funnel plot and leave-one-out plot for the MR analysis of multiple birth and cardiovascular system disease. [file 12967_2023_4423_MOESM11_ESM.docx]
