# Supplementary material for: Long-term outcomes of offspring from multiple gestations: a two-sample Mendelian randomization study on multi-system diseases using UK Biobank and FinnGen databases
Source: J Transl Med. 2023 Sep 8;21:608. doi: 10.1186/s12967-023-04423-w (PMC10492369; doi:10.1186/s12967-023-04423-w)

**Chronic obstructive pulmonary disease – Finngen**


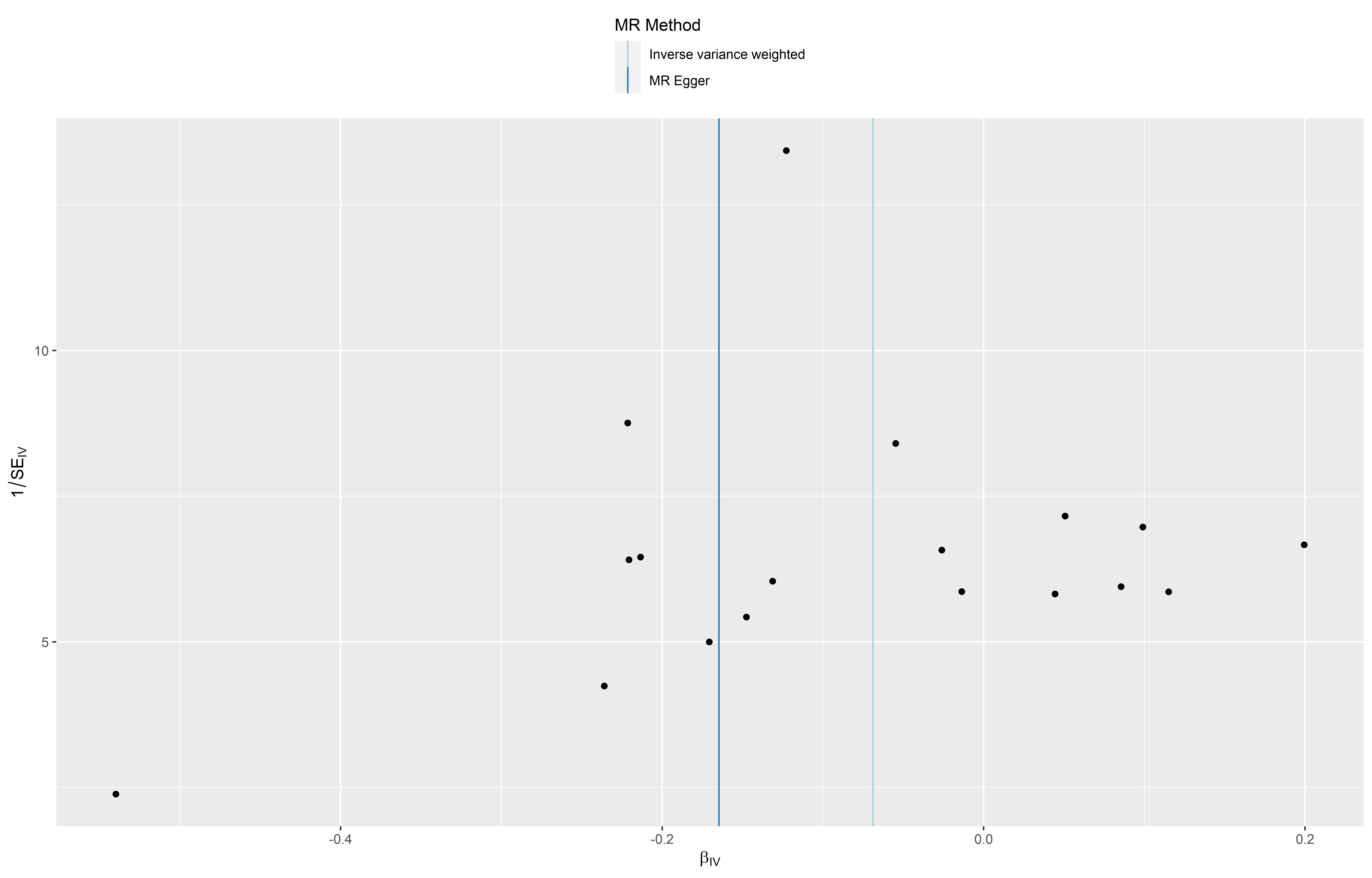

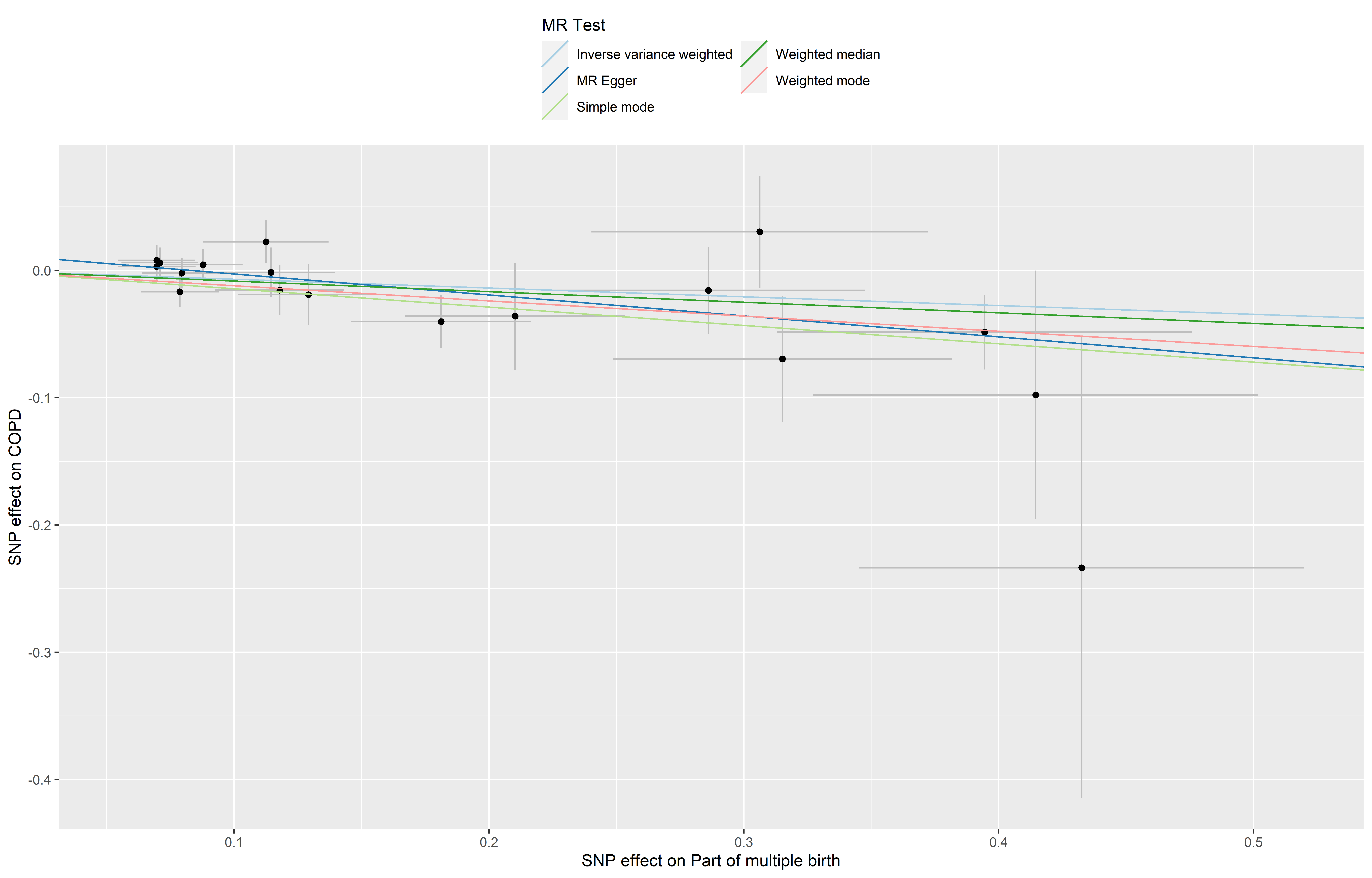


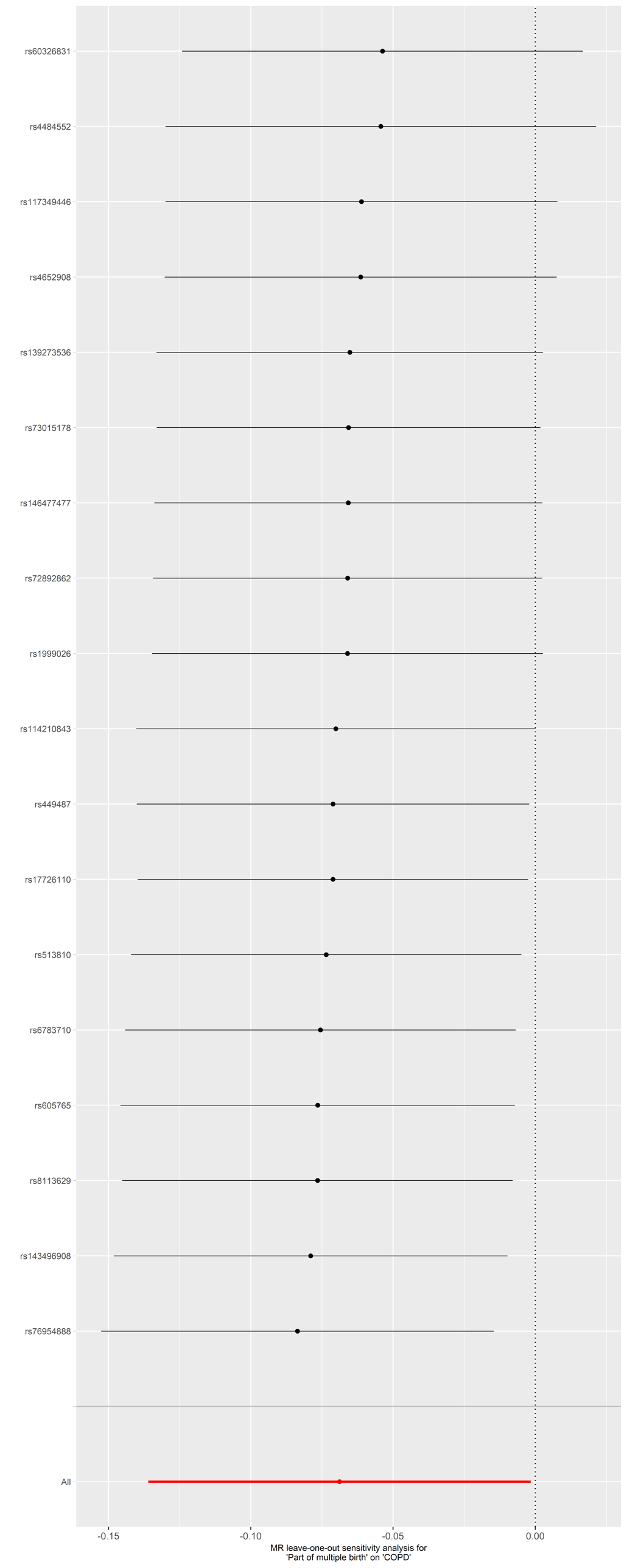


**Chronic obstructive pulmonary disease – UK Biobank**


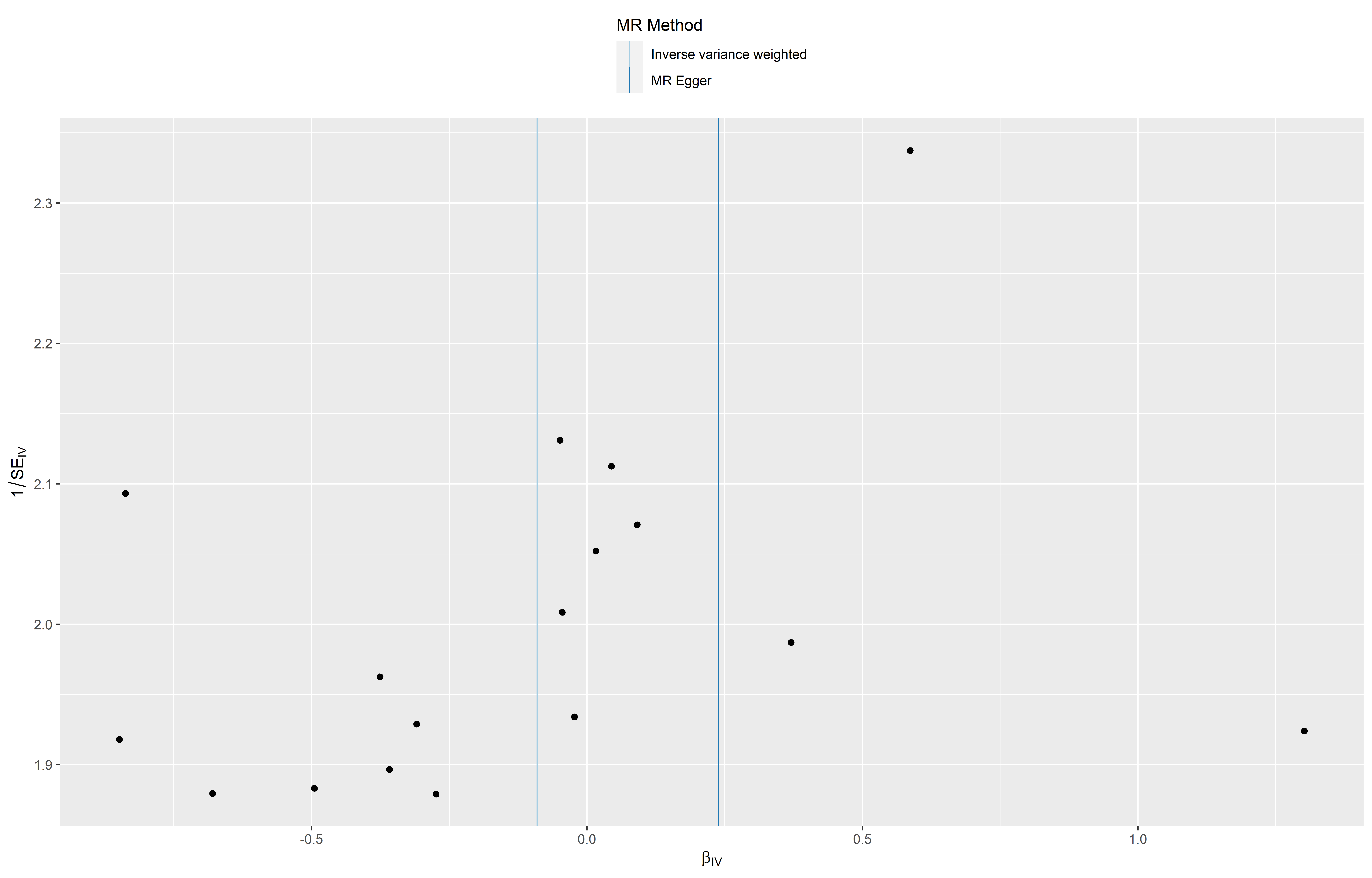

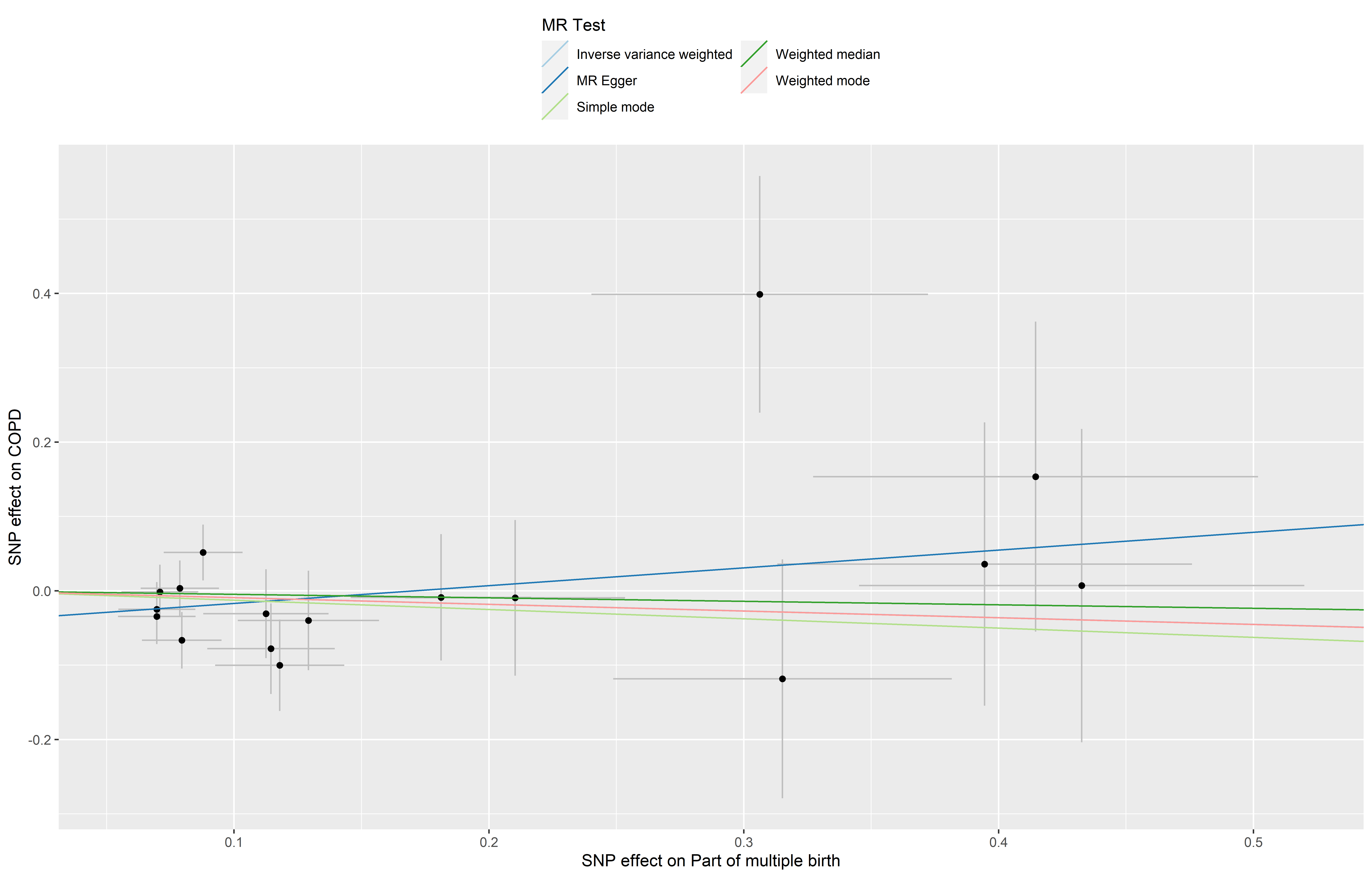


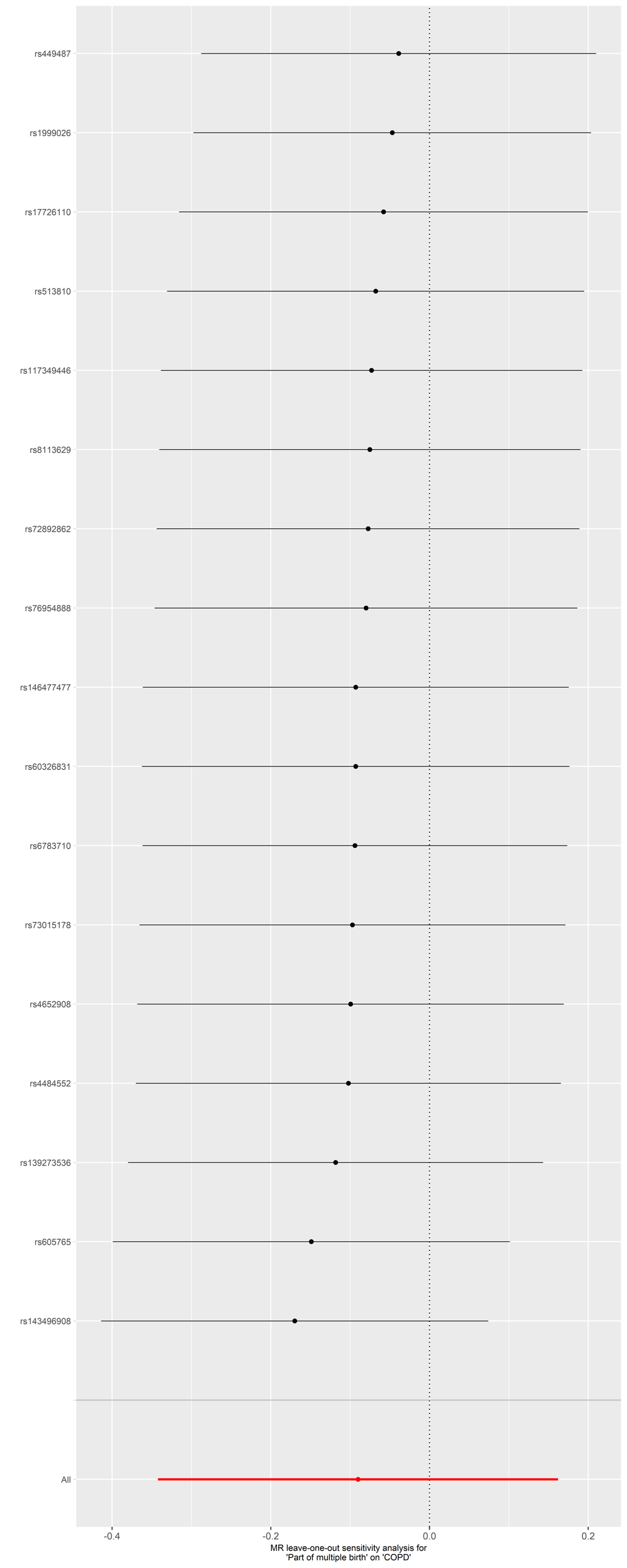


**Asthma – Finngen**


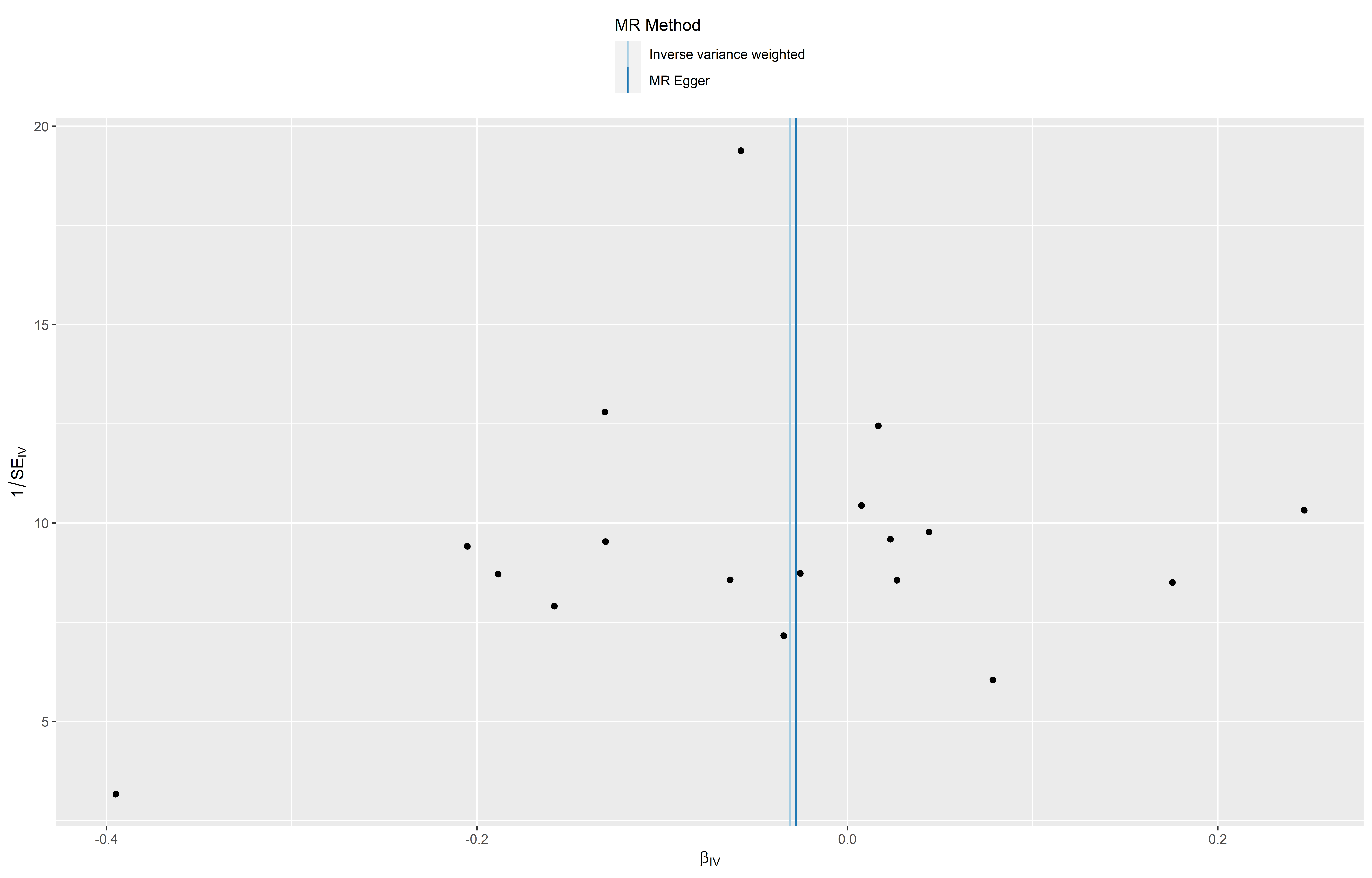

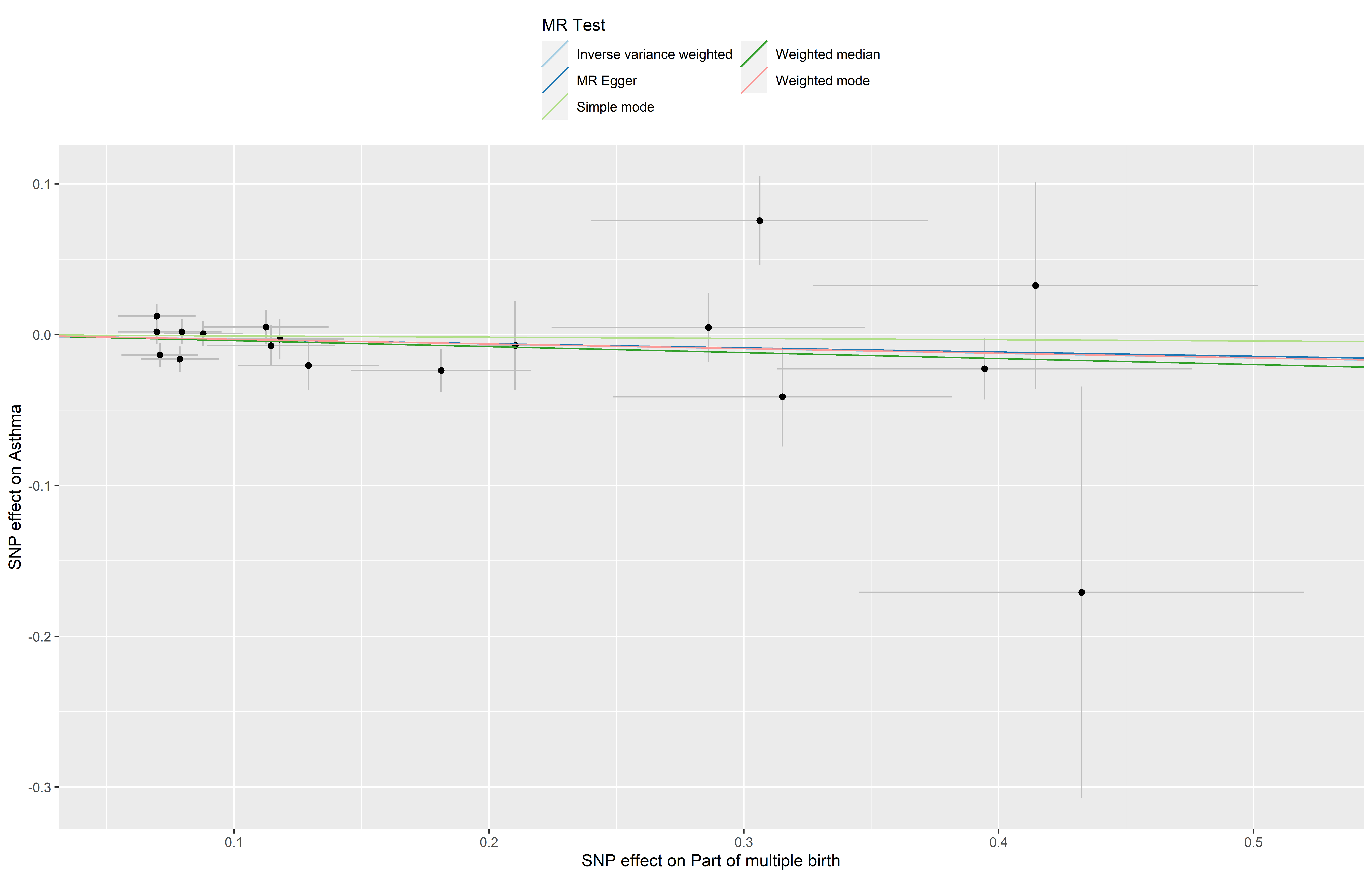


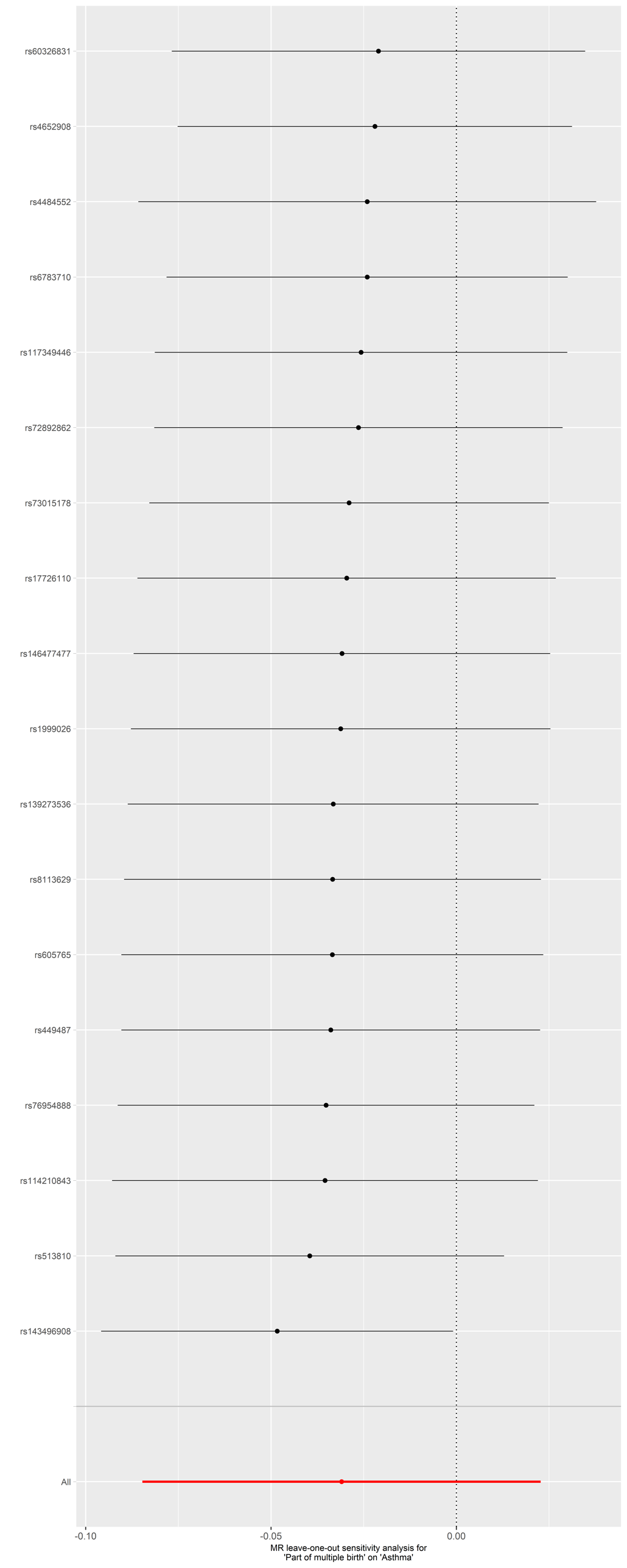


**Asthma – UK Biobank**


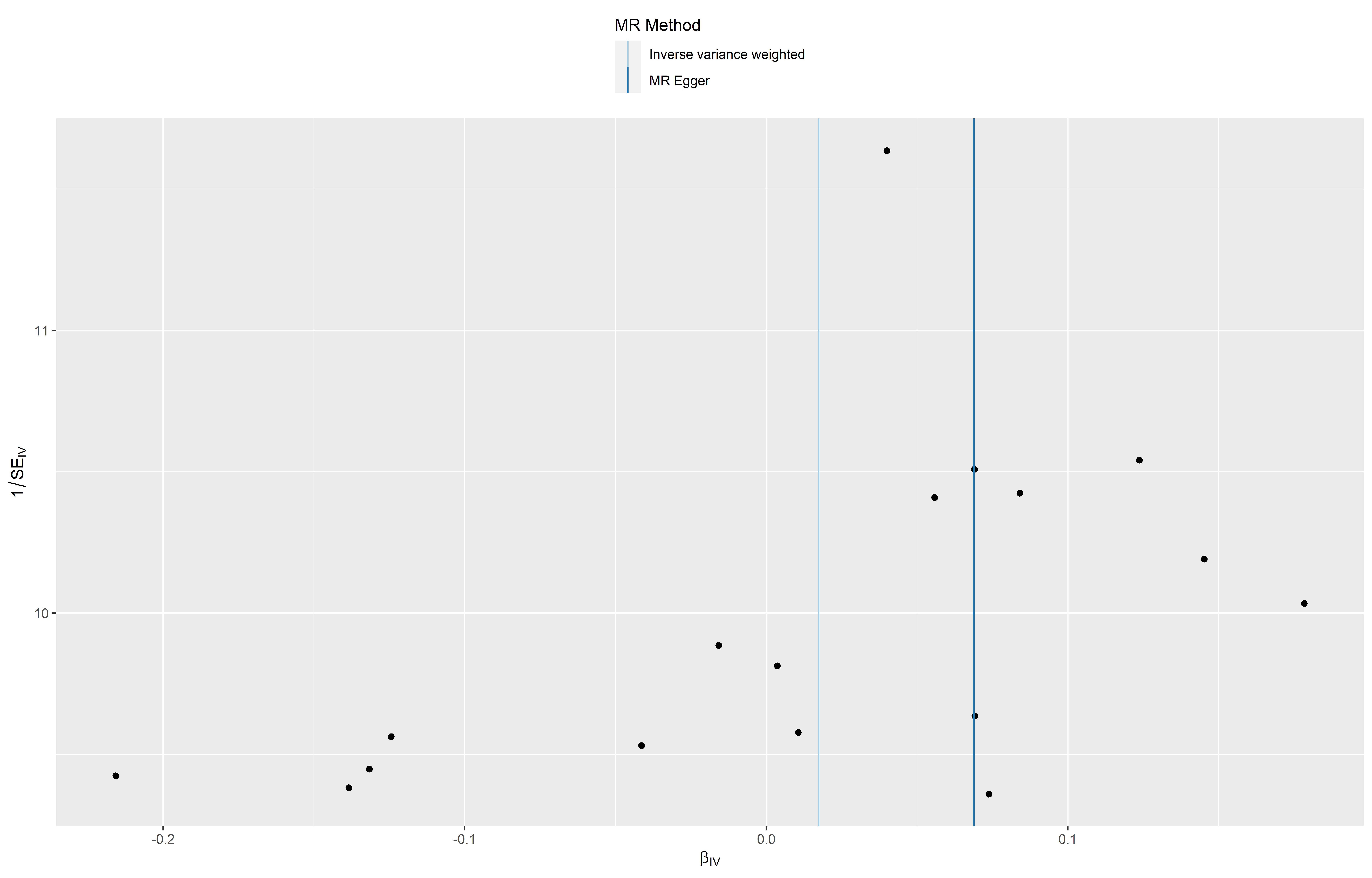

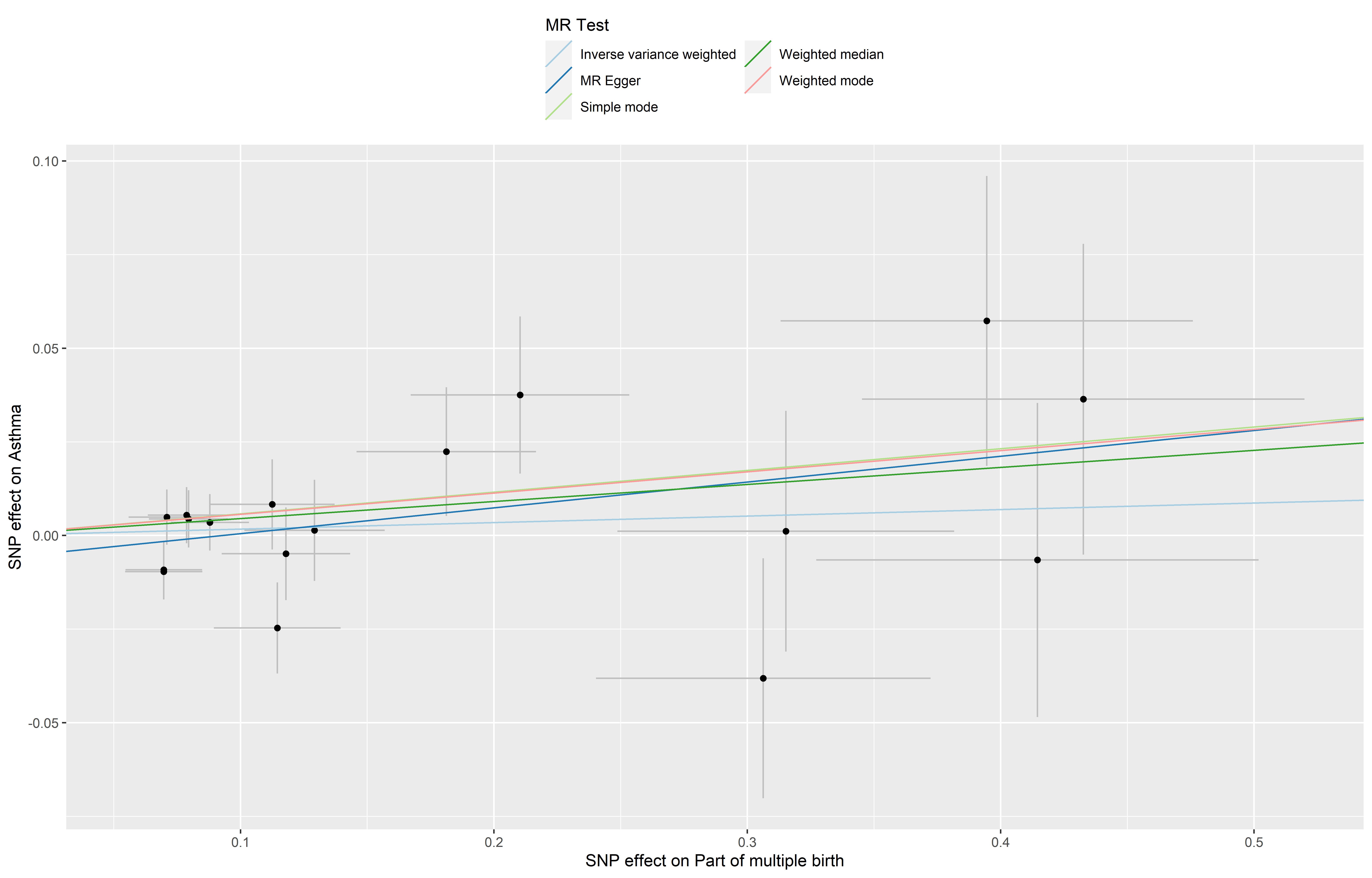


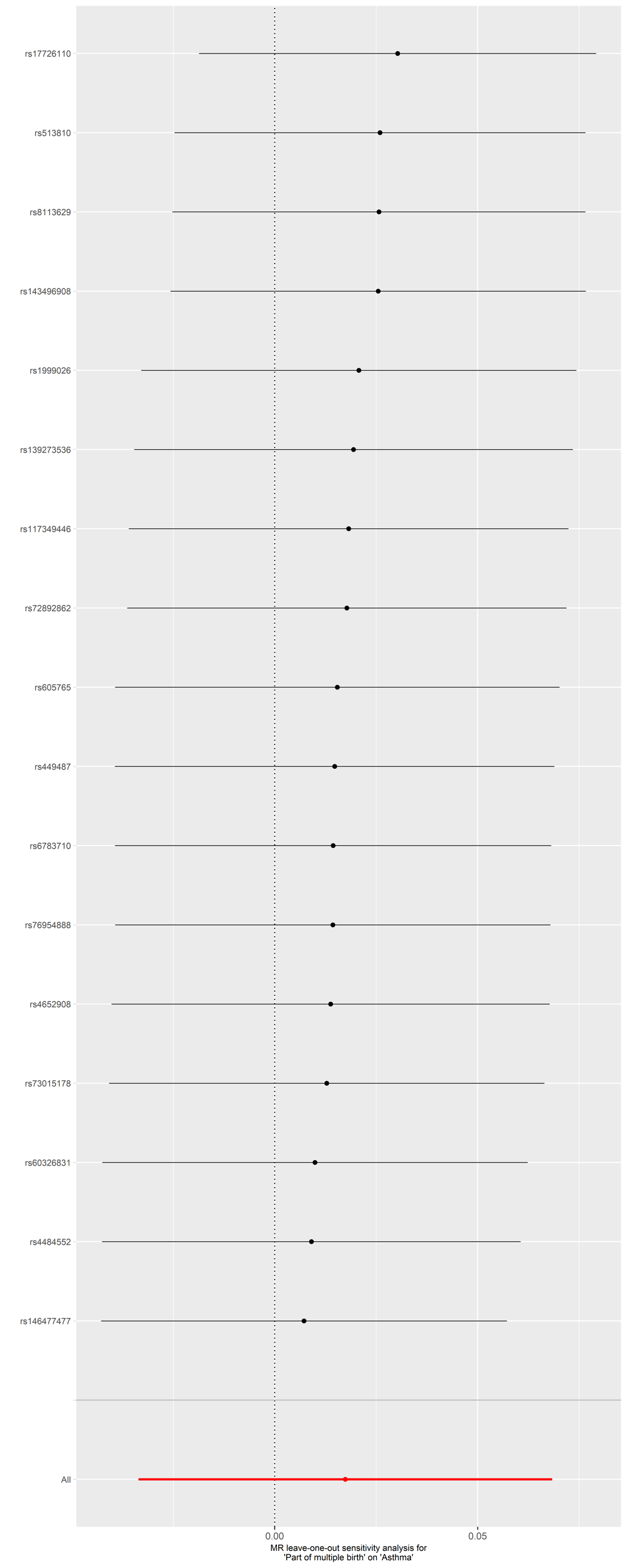


**Bronchitis – Finngen**


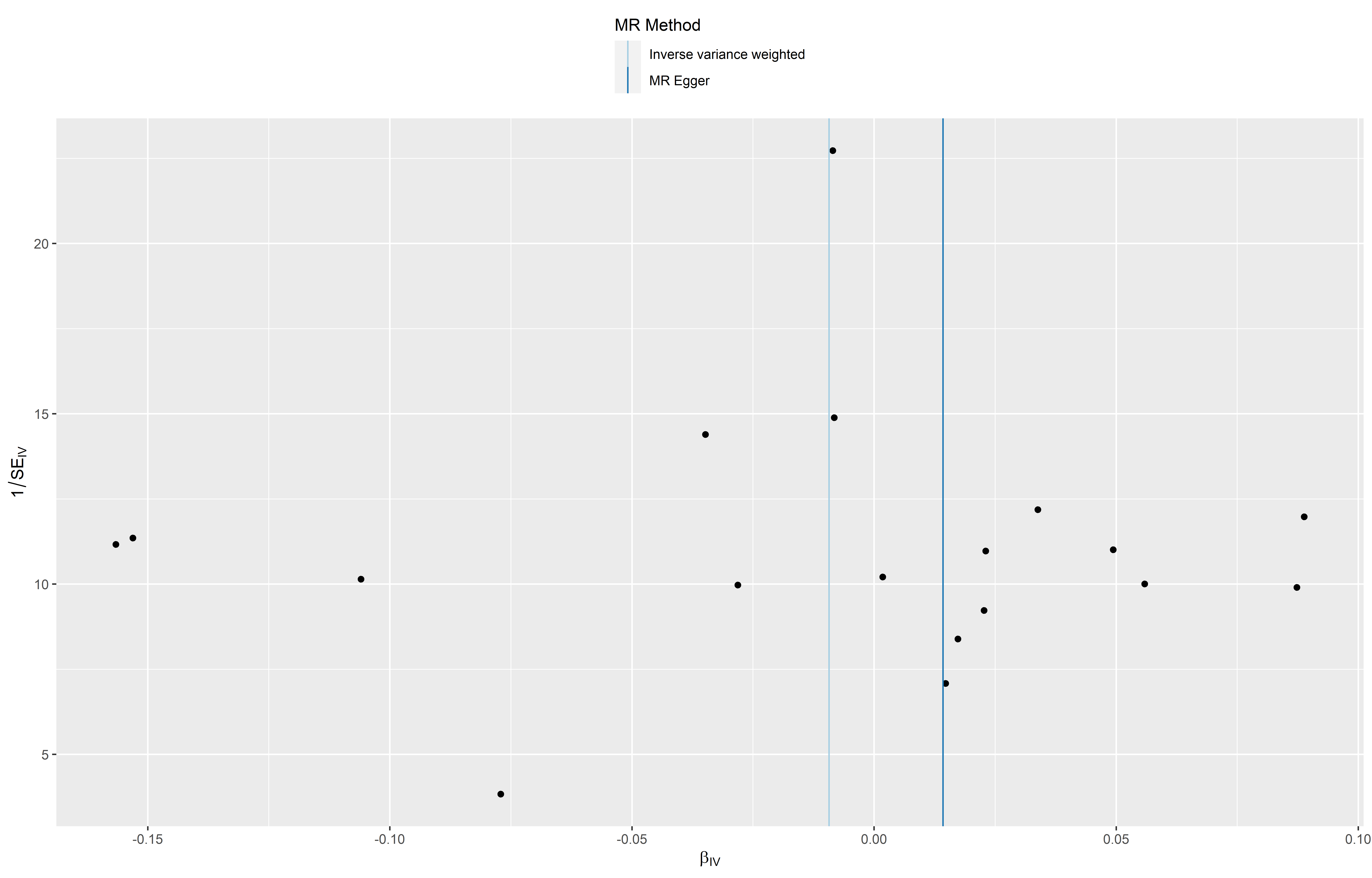

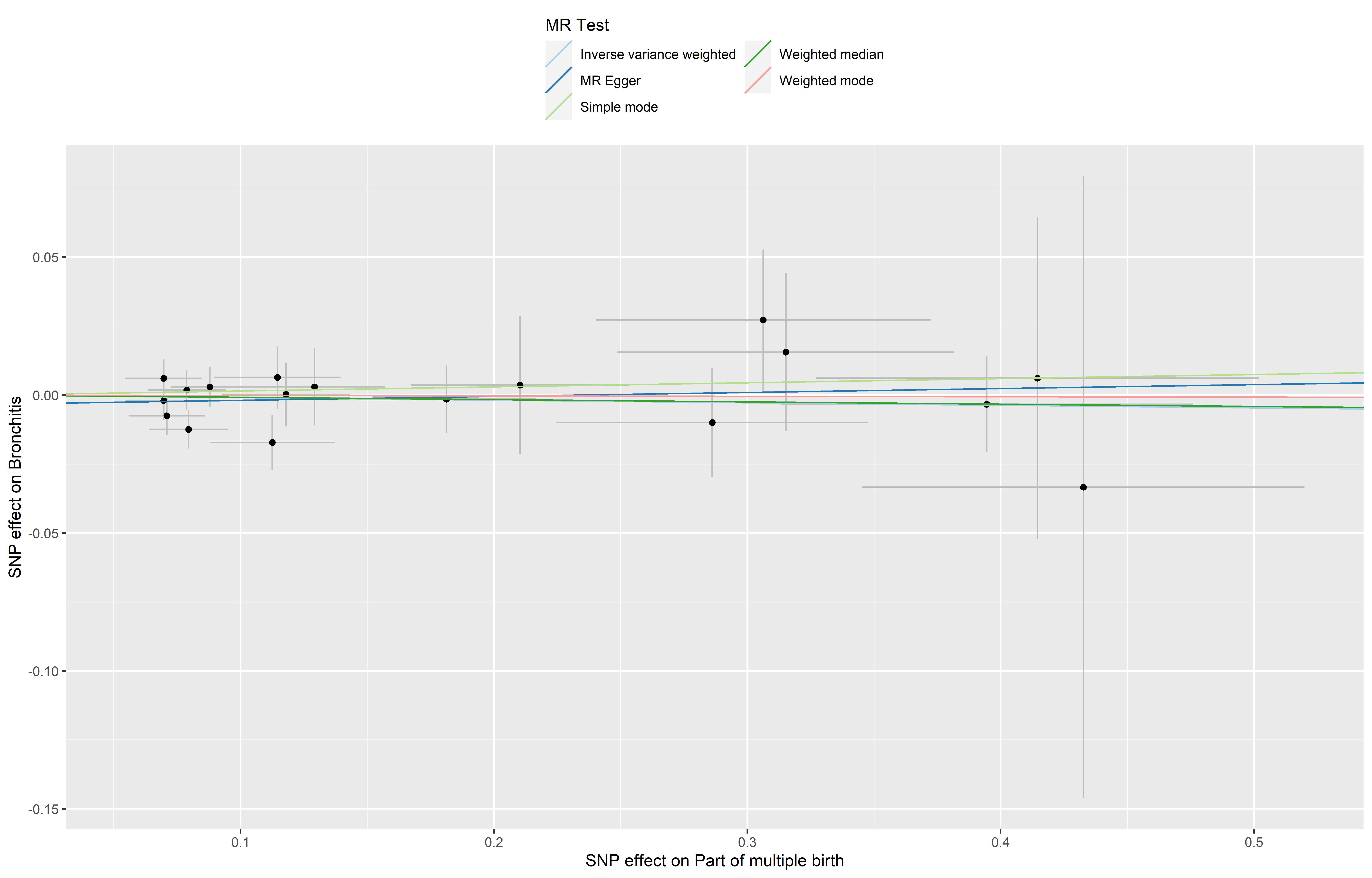


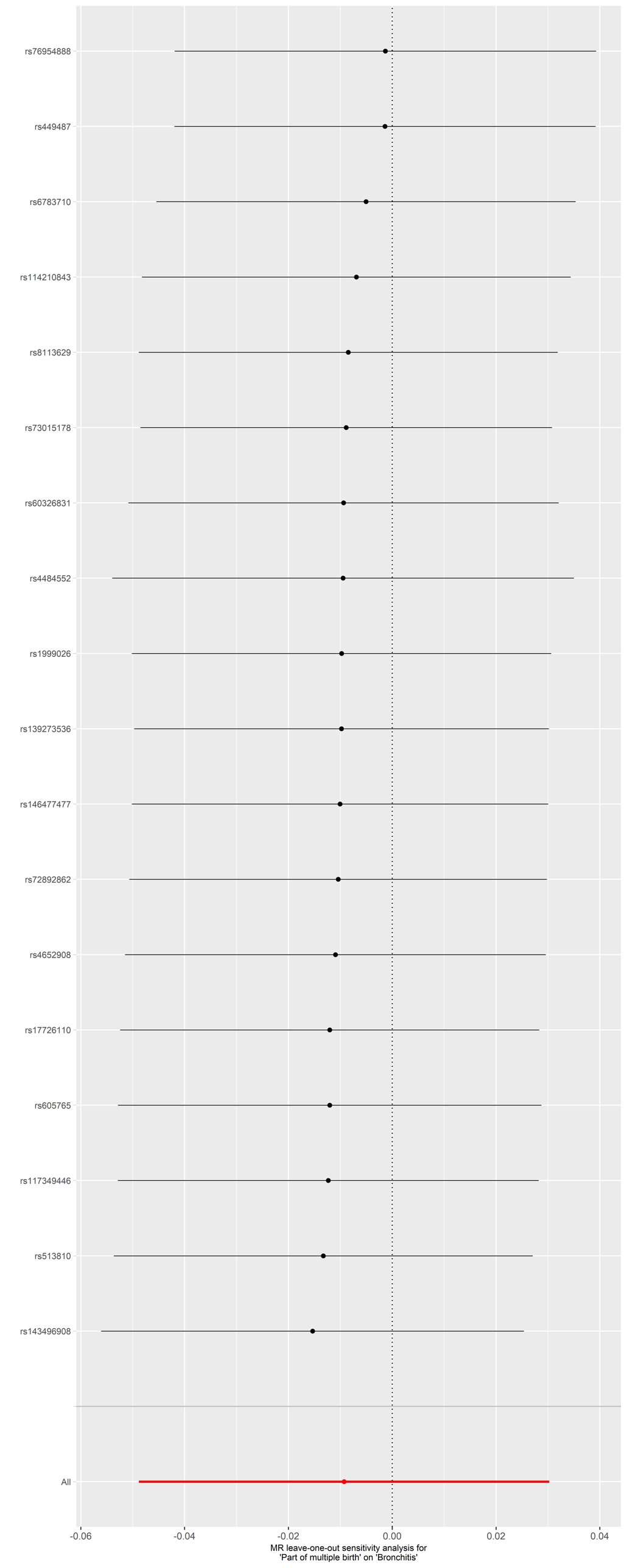


**Bronchitis – UK Biobank**


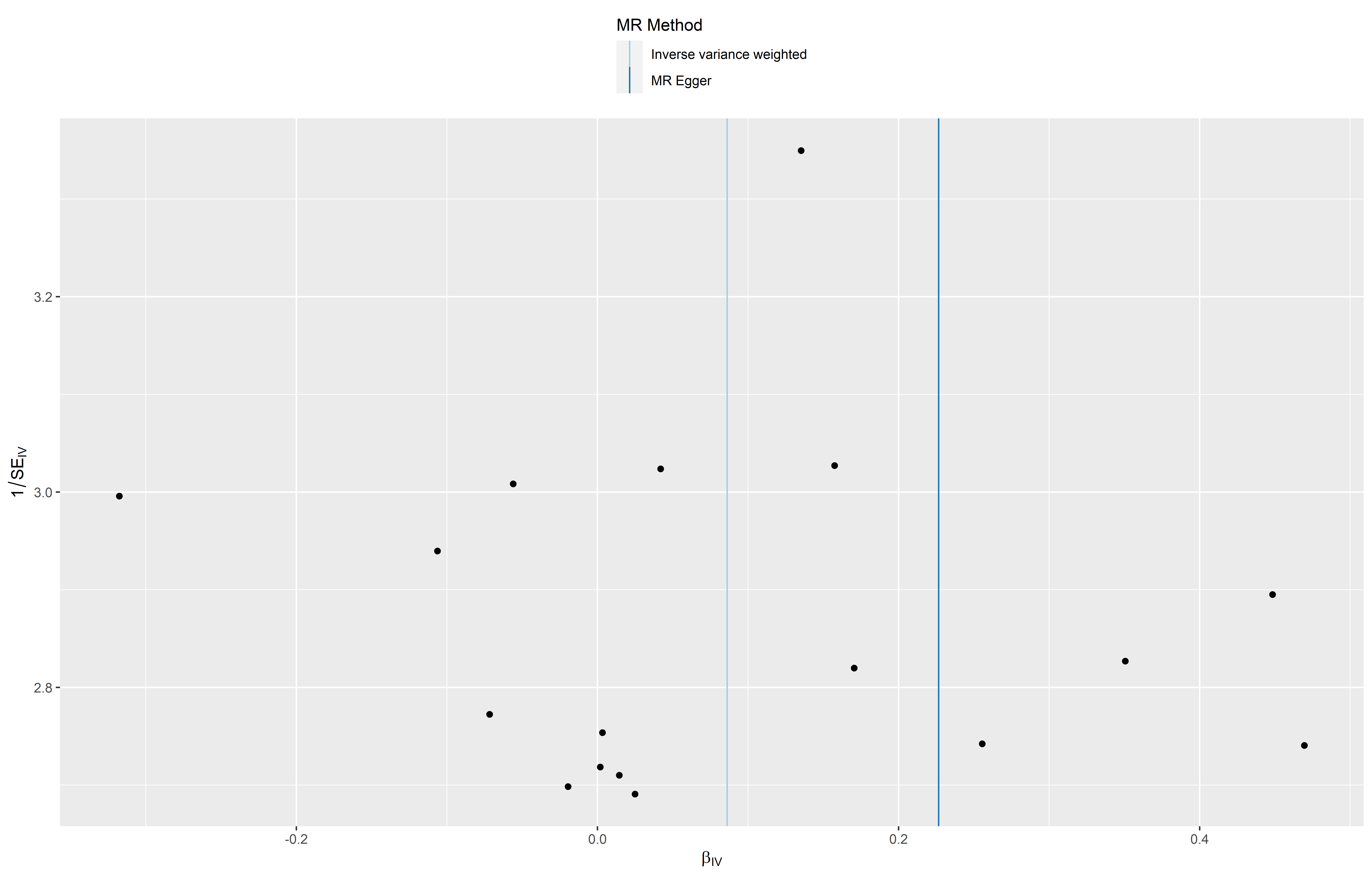

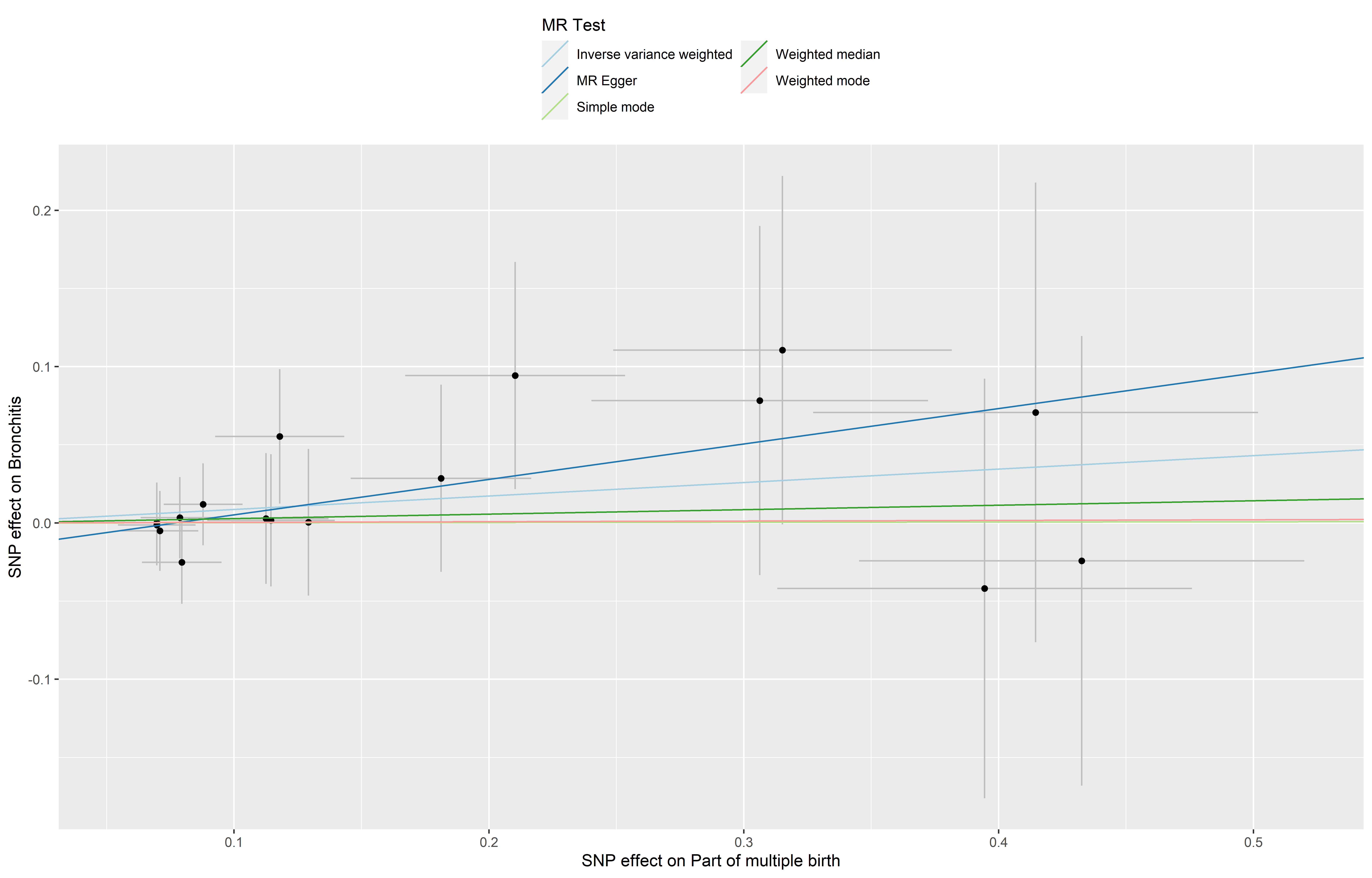


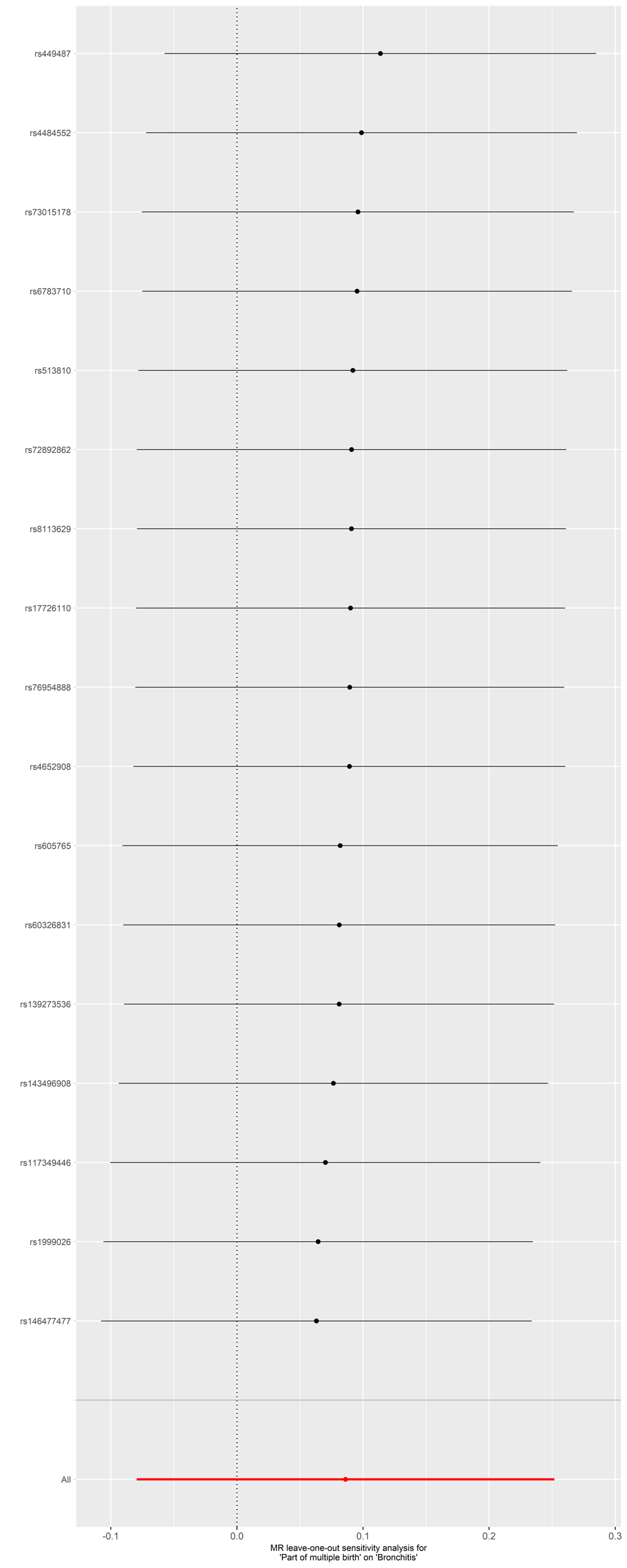


**Tuberculosis - Finngen**


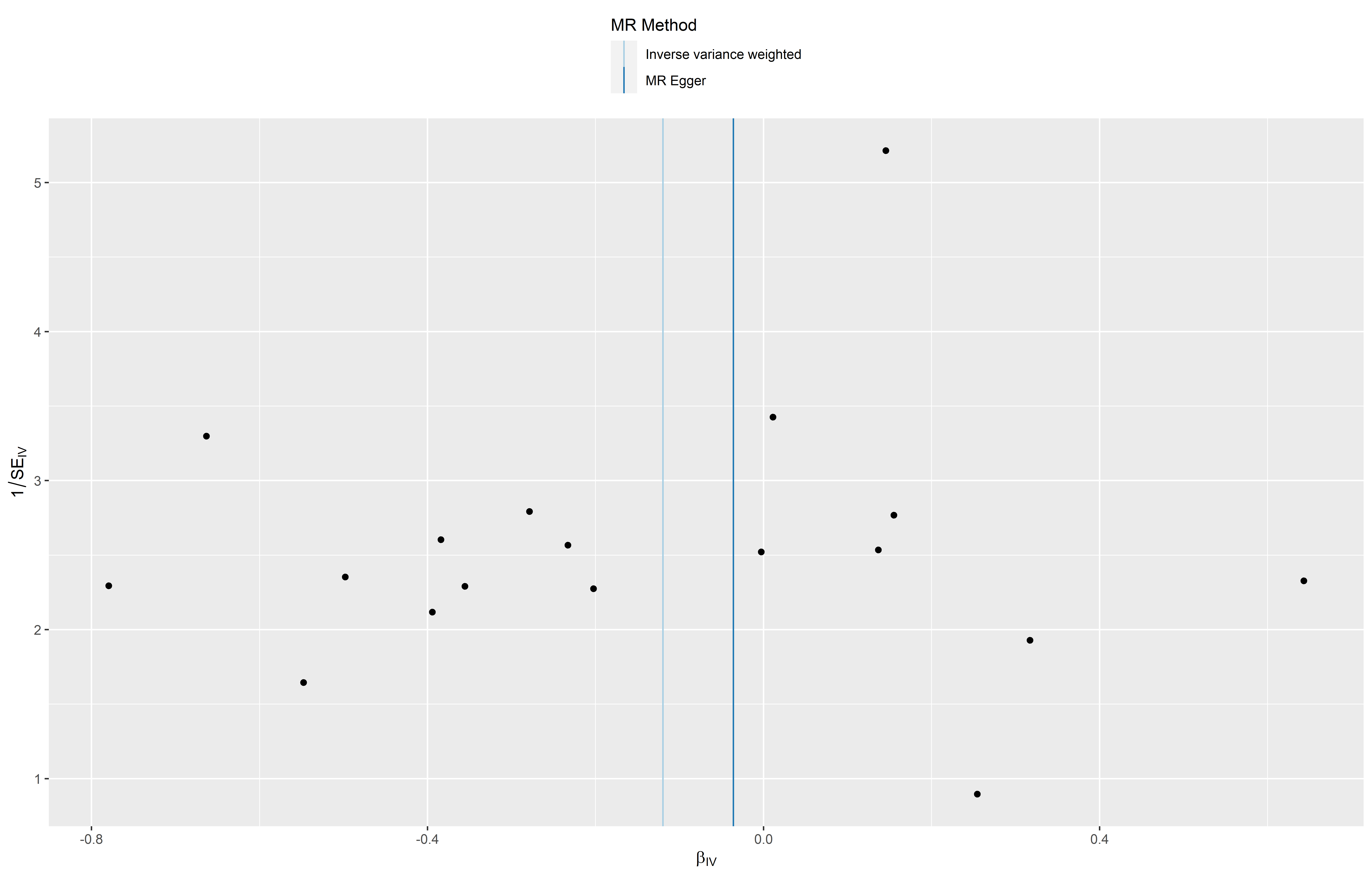

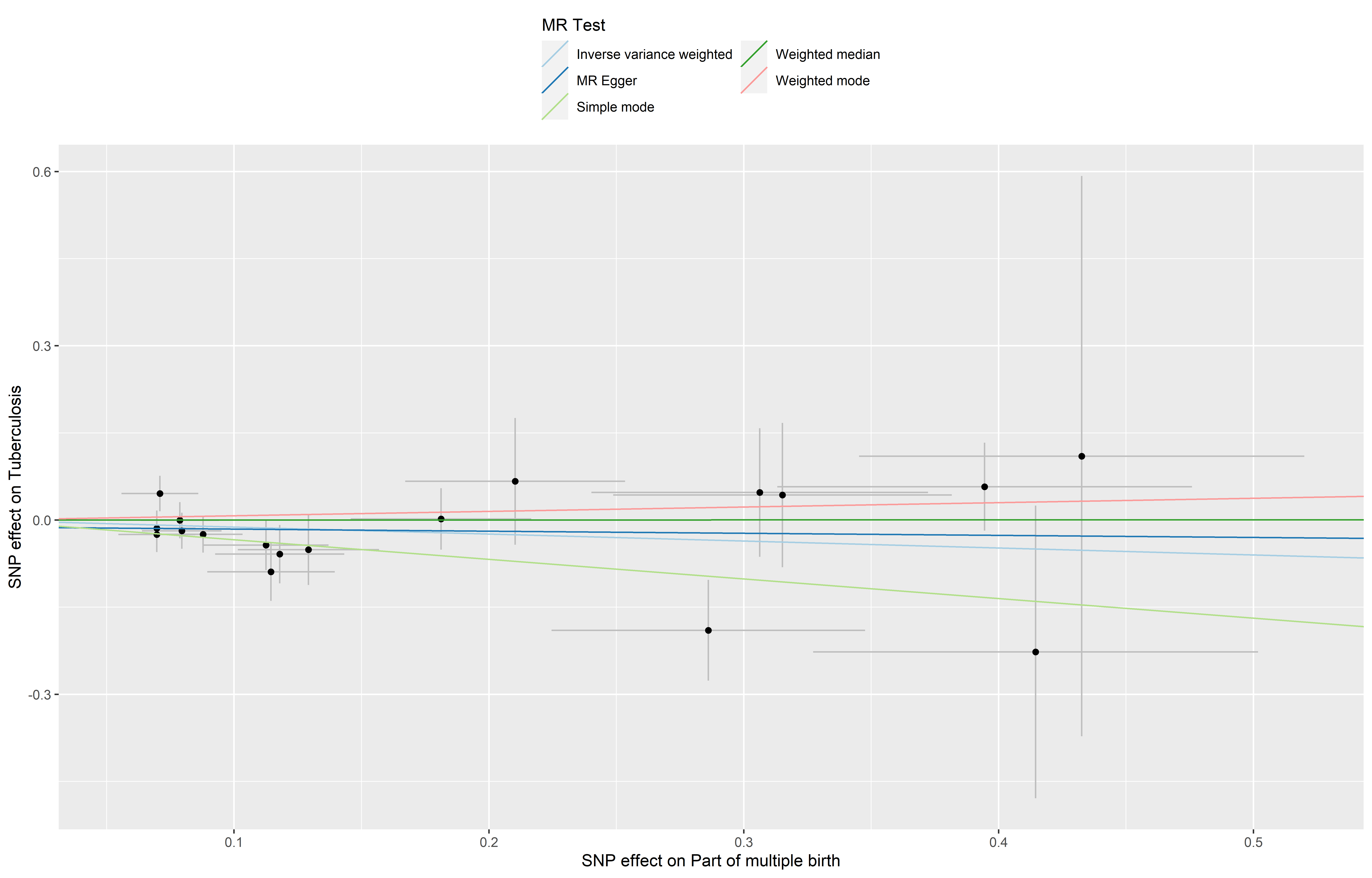


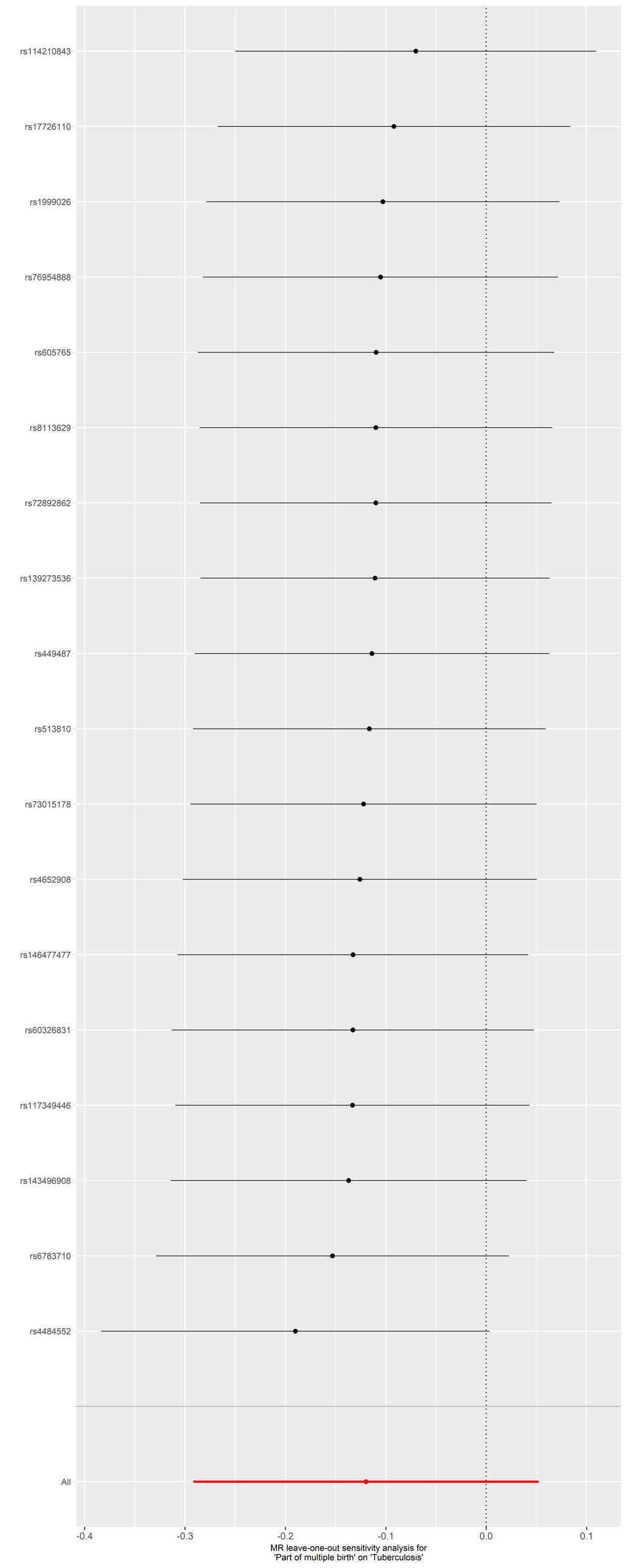


**Tuberculosis – UK Biobank**


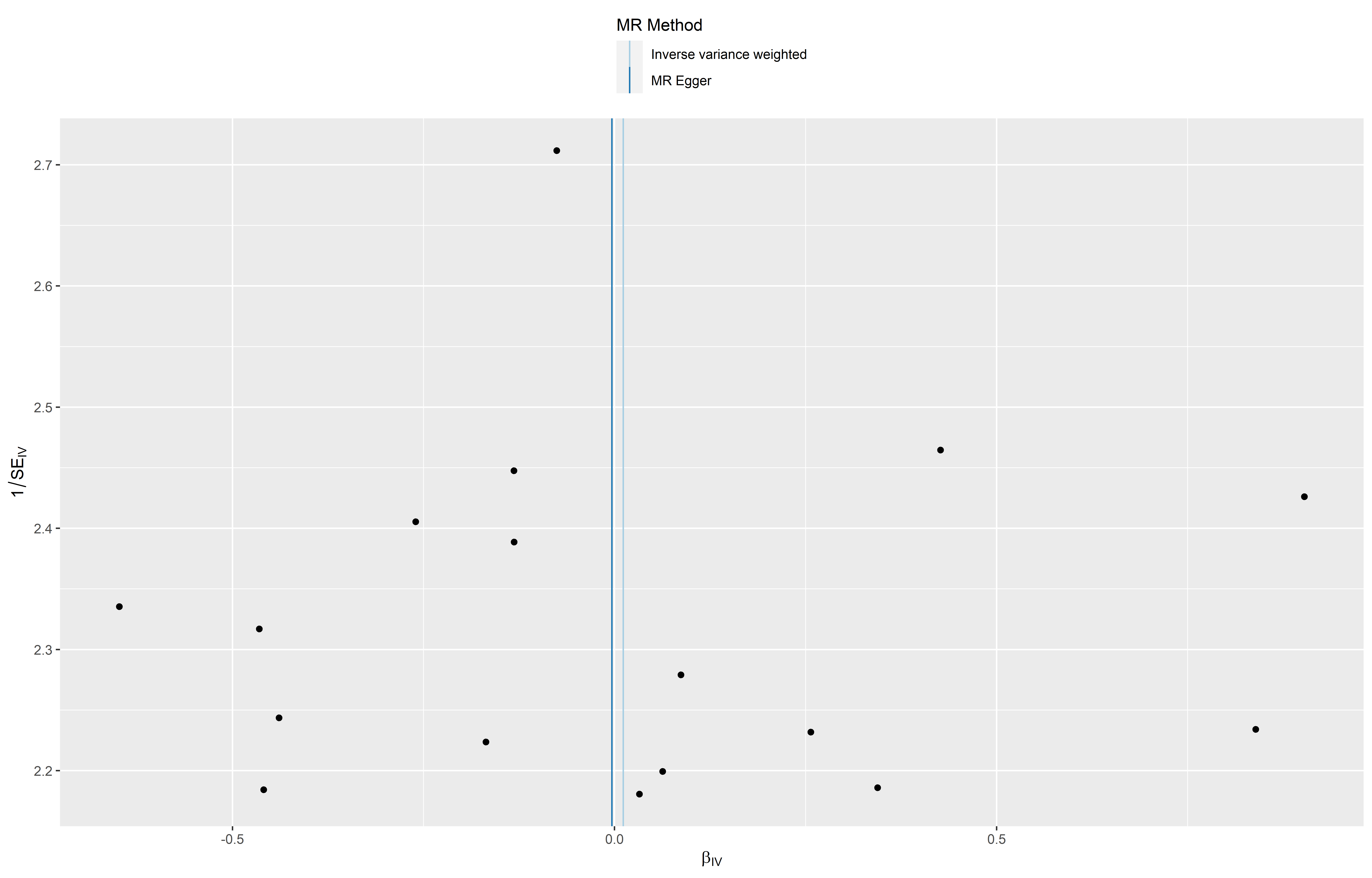

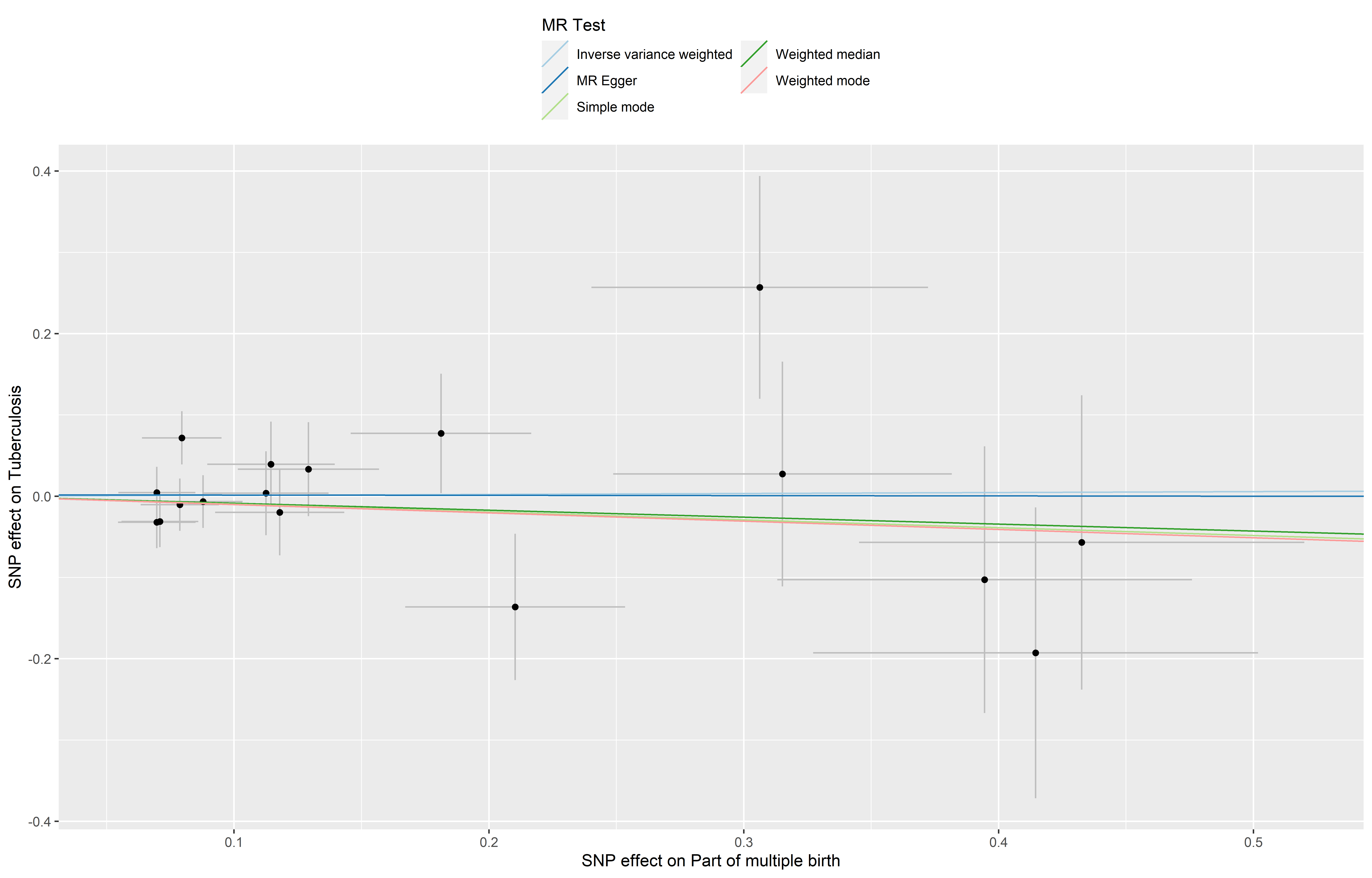


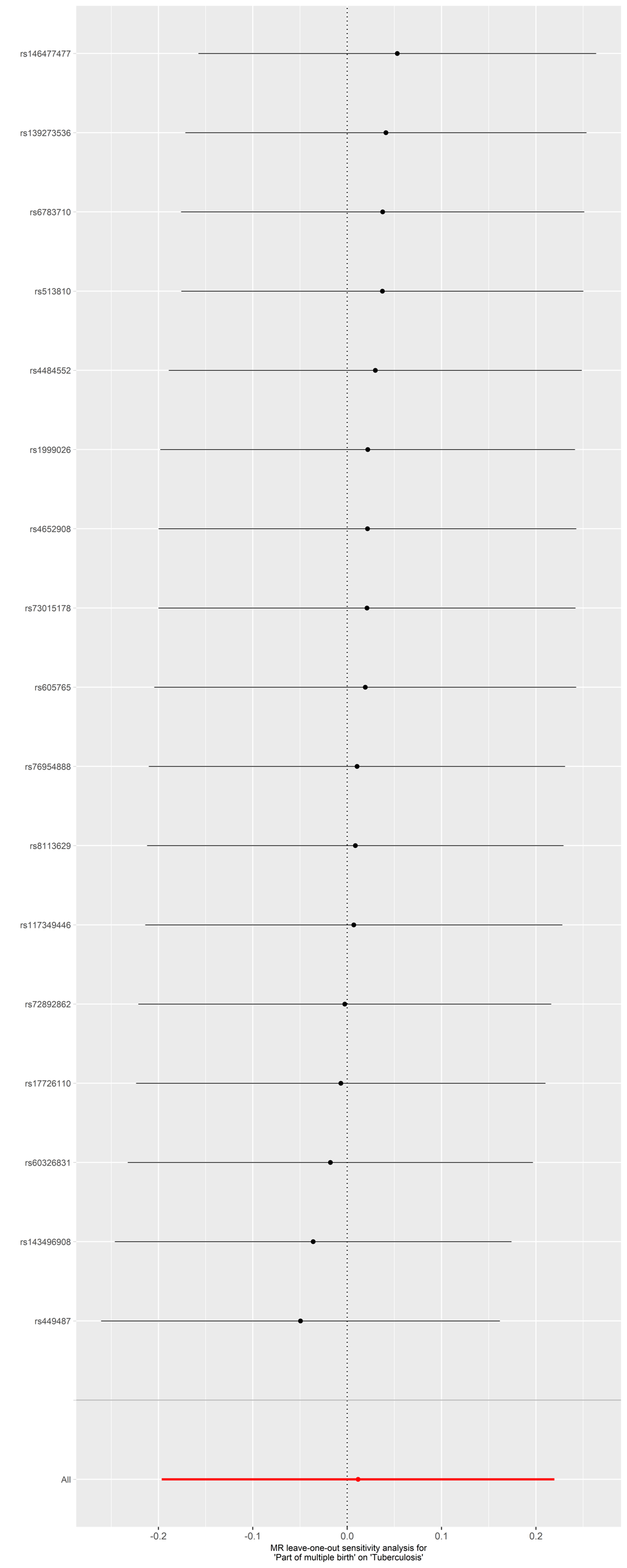

Supplement: Supplementary file 12 — Additional file 12: Material S4. The scatter plot, funnel plot and leave-one-out plot for the MR analysis of multiple birth and respiratory system disease. [file 12967_2023_4423_MOESM12_ESM.docx]
