# Supplementary material for: Long-term outcomes of offspring from multiple gestations: a two-sample Mendelian randomization study on multi-system diseases using UK Biobank and FinnGen databases
Source: J Transl Med. 2023 Sep 8;21:608. doi: 10.1186/s12967-023-04423-w (PMC10492369; doi:10.1186/s12967-023-04423-w)

**Gastric ulcer – Finngen**


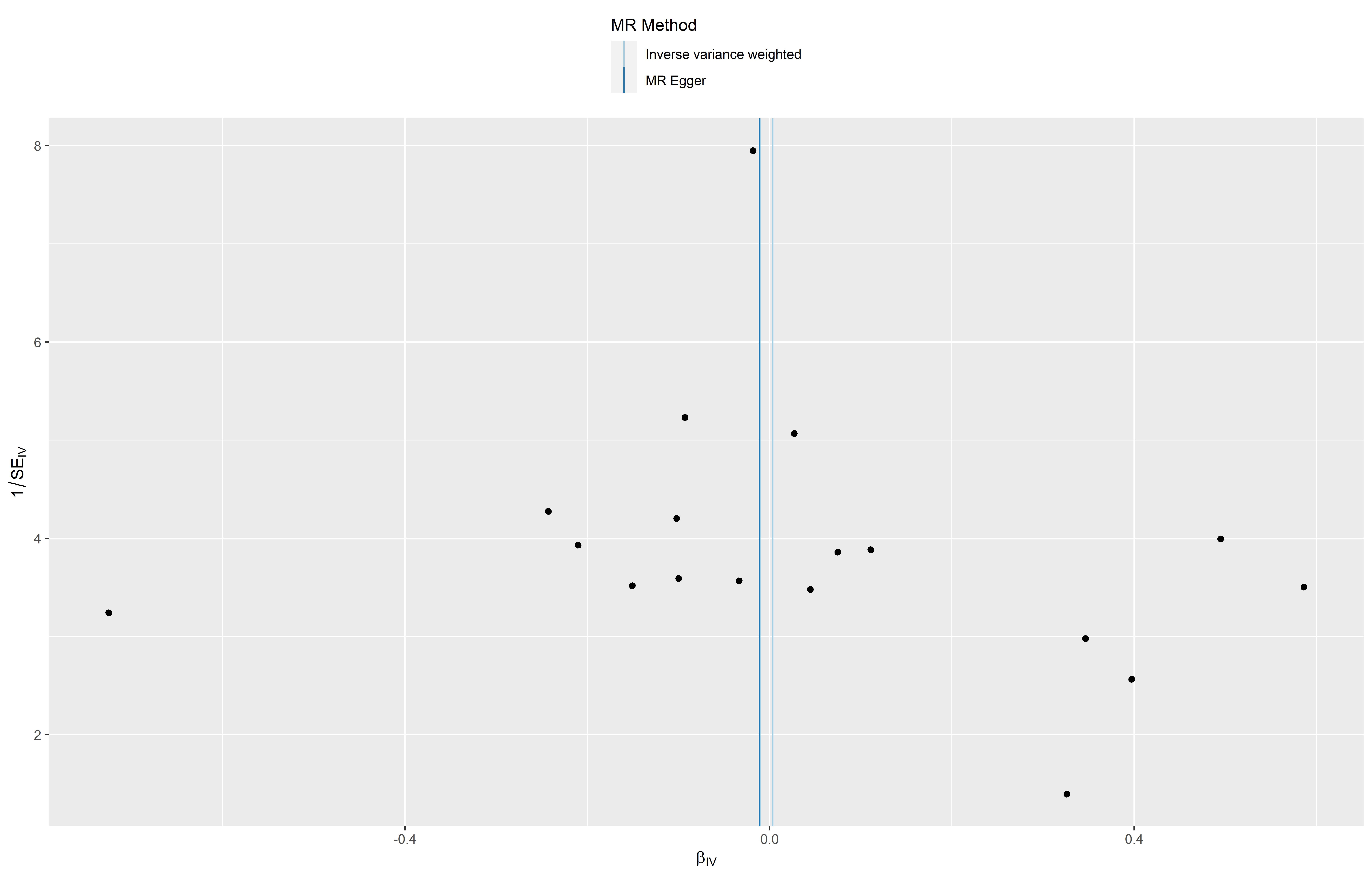

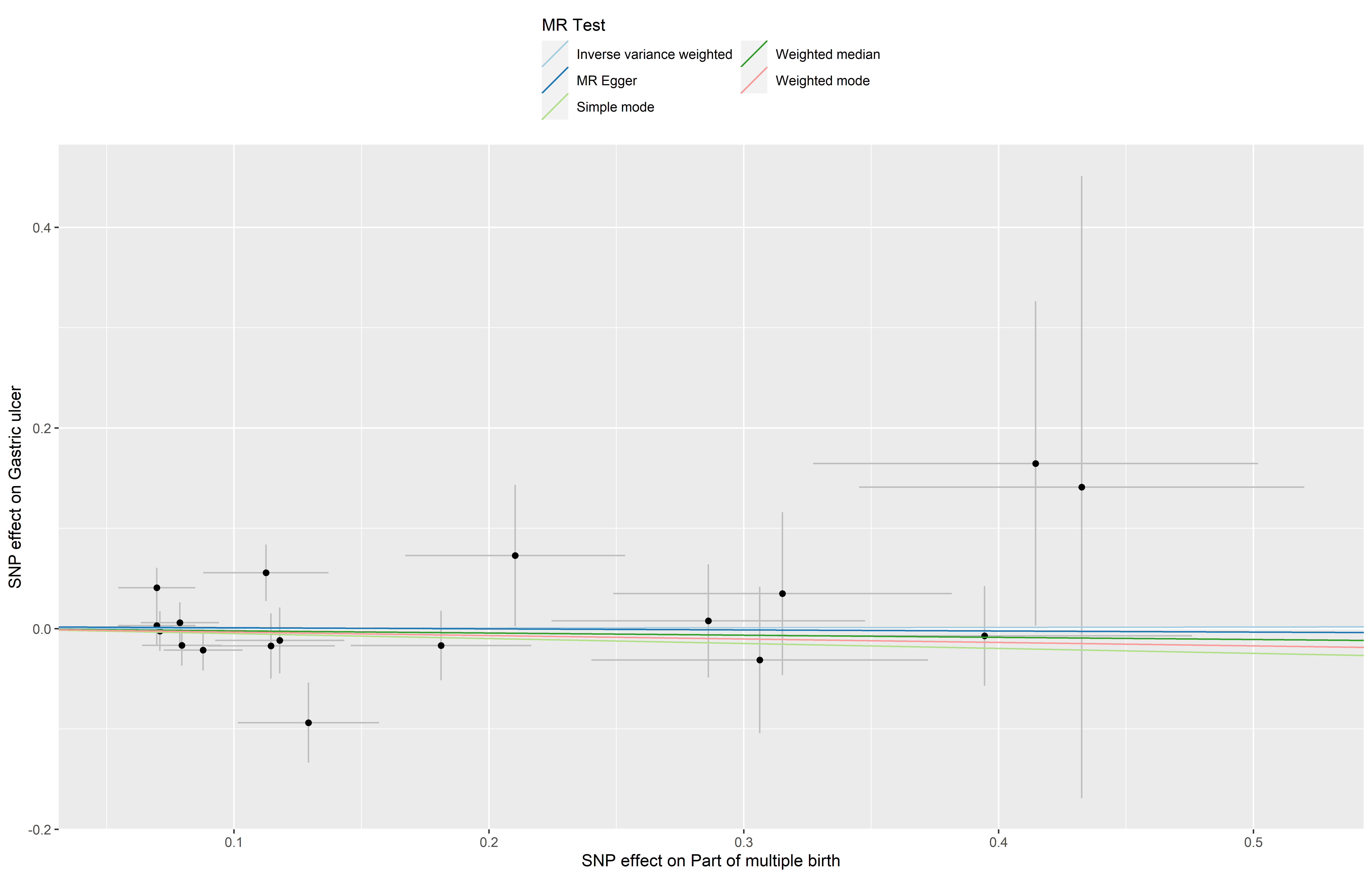


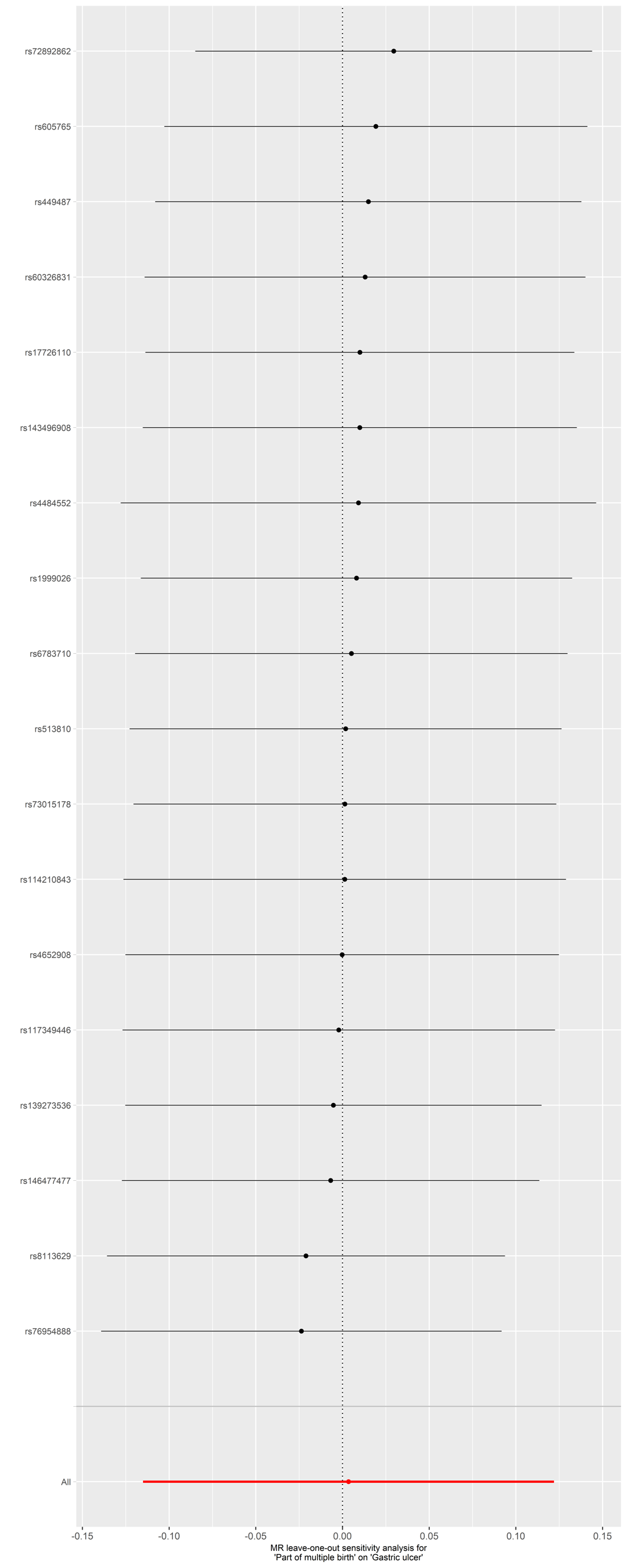


**Gastric ulcer – UK Biobank**


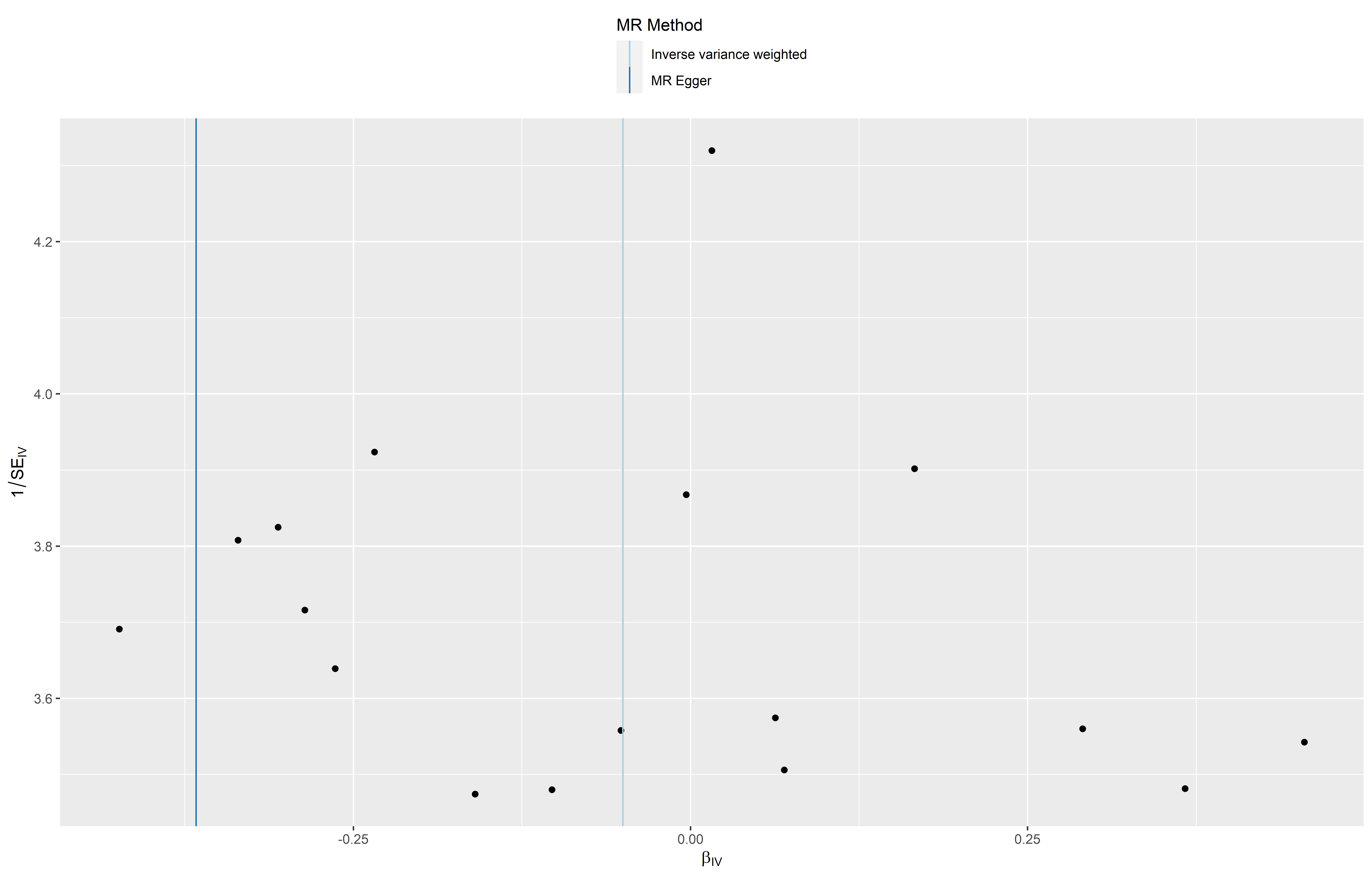

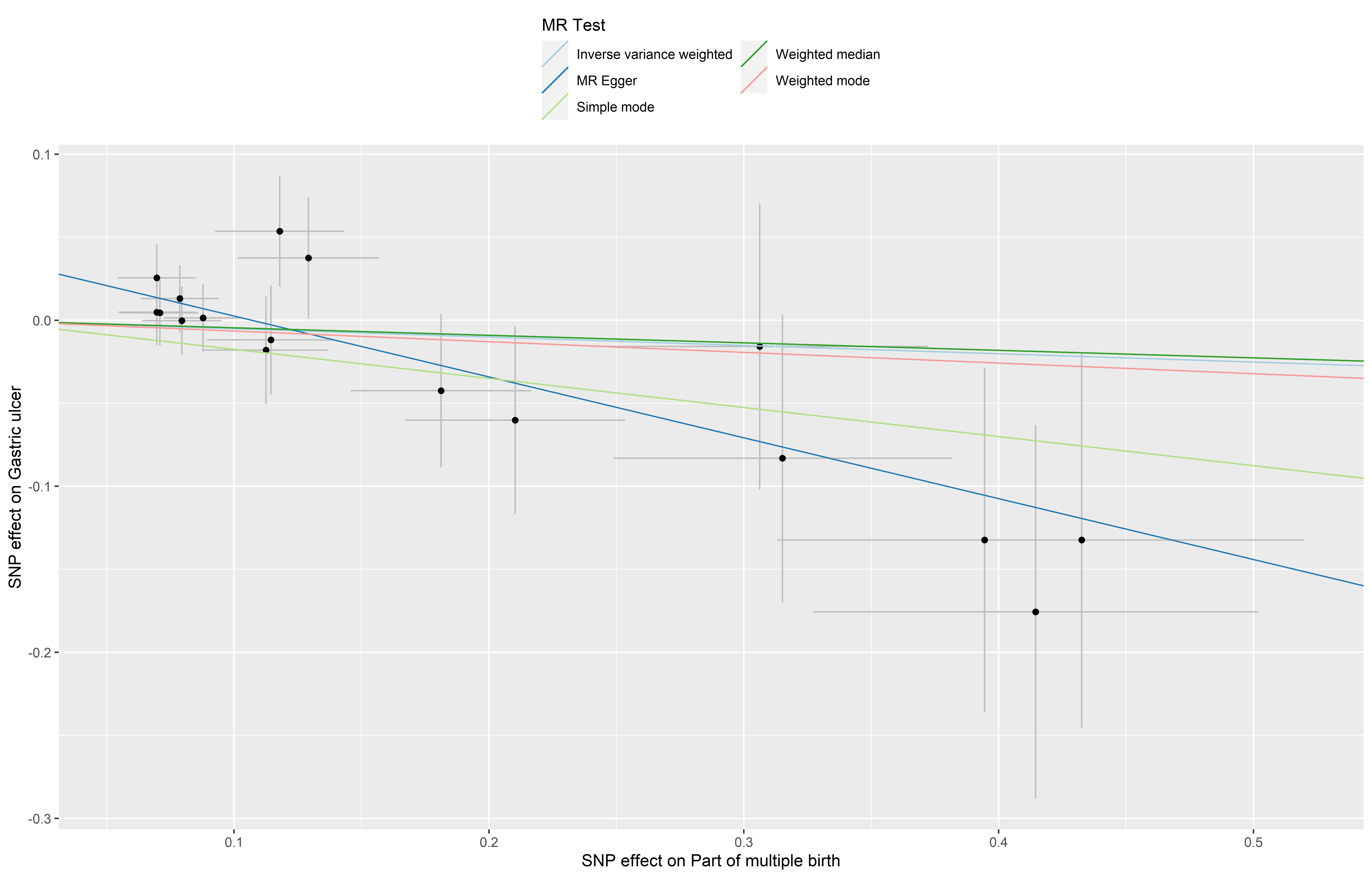


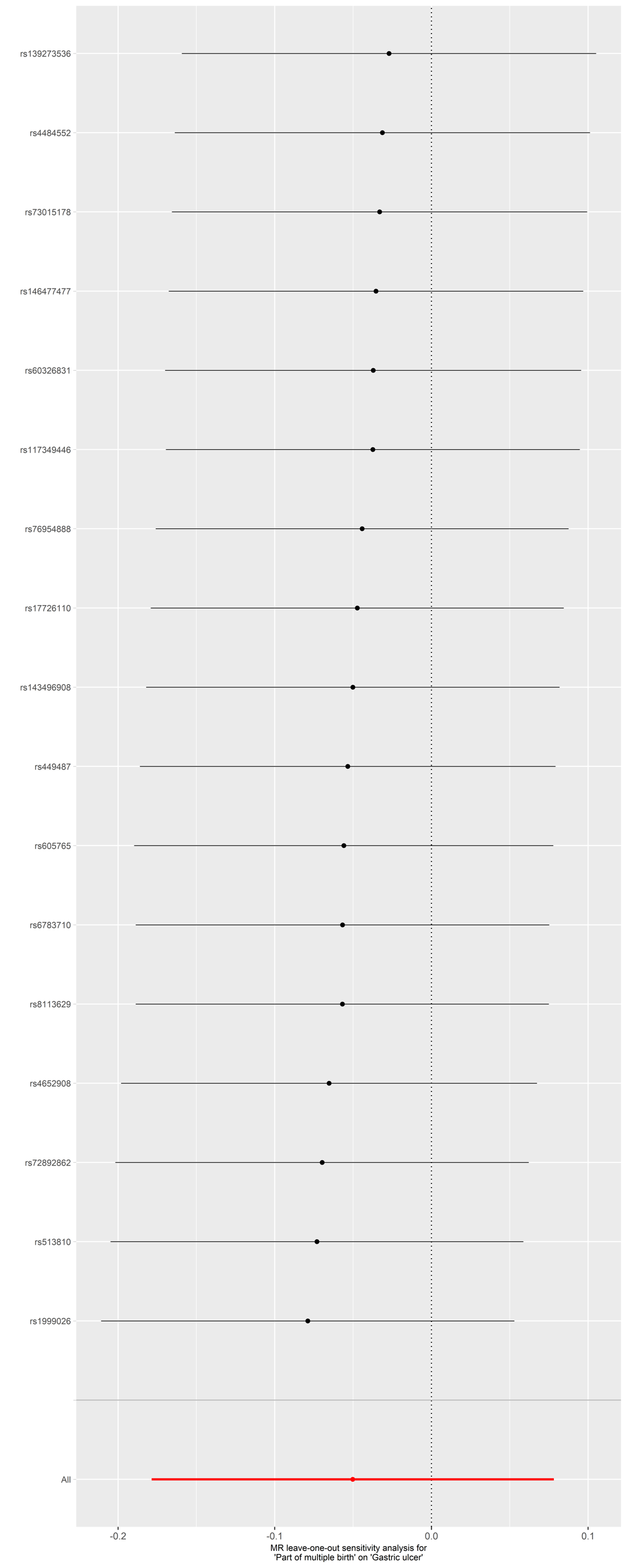


**Duodenal ulcer – Finngen**


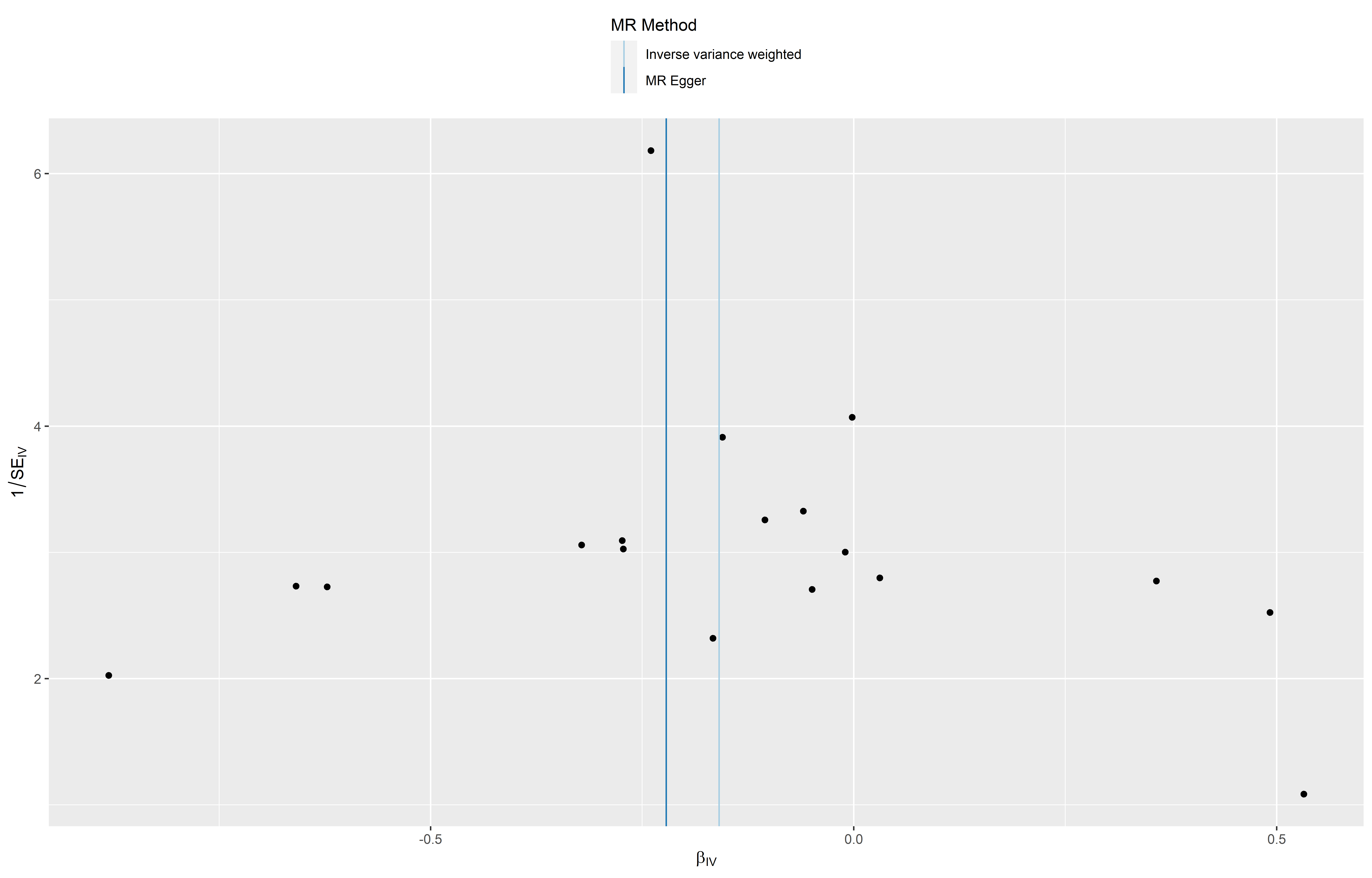

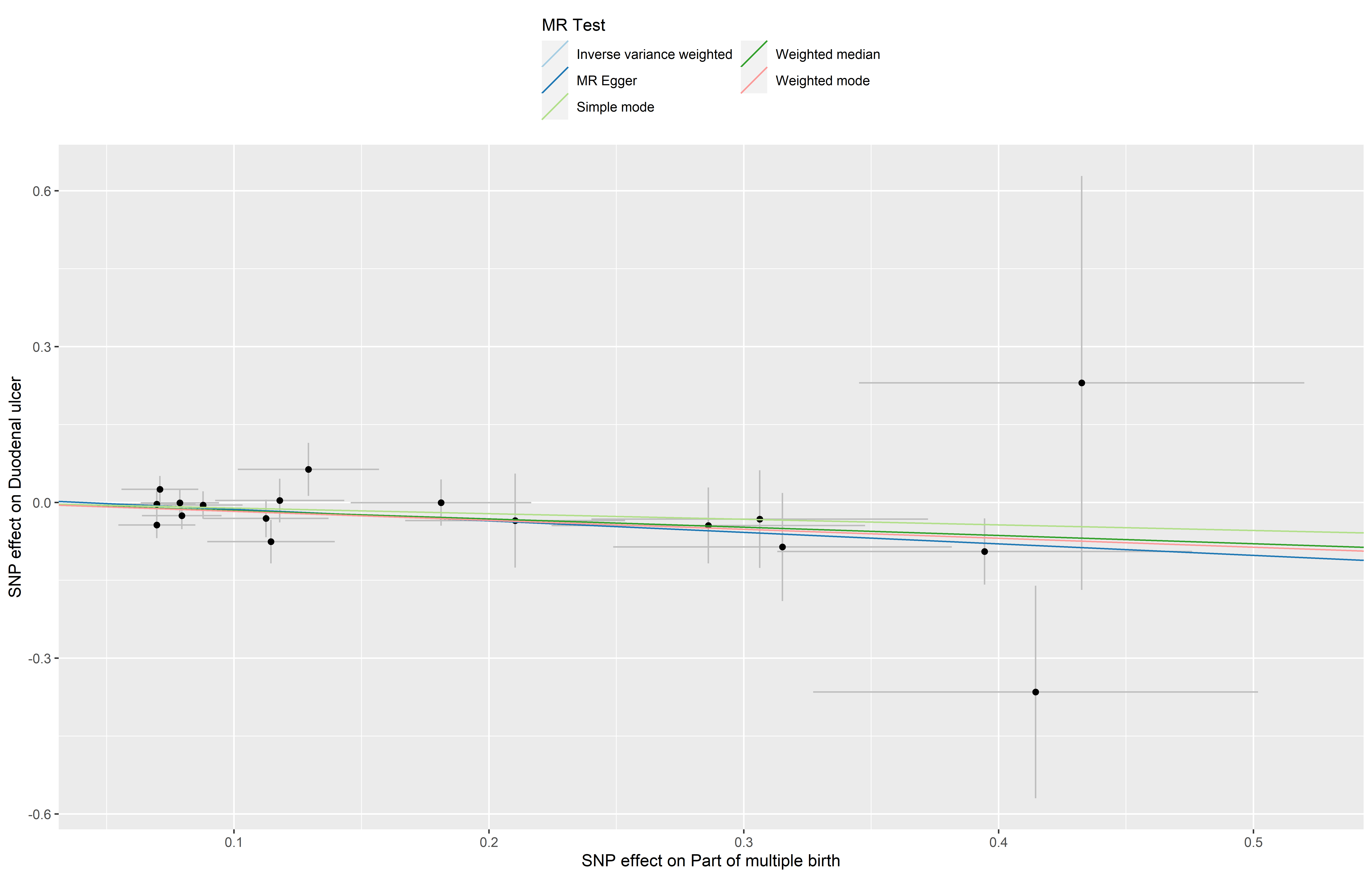


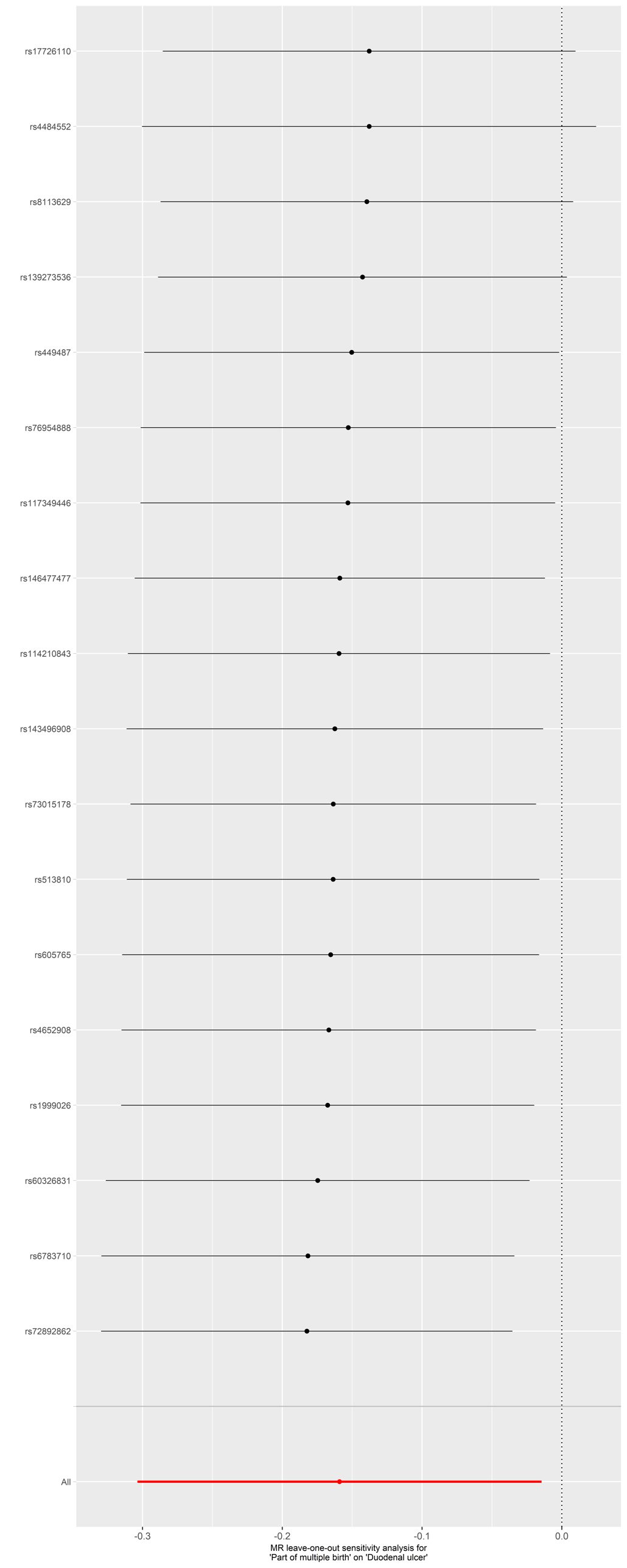


**Duodenal ulcer – UK Biobank**


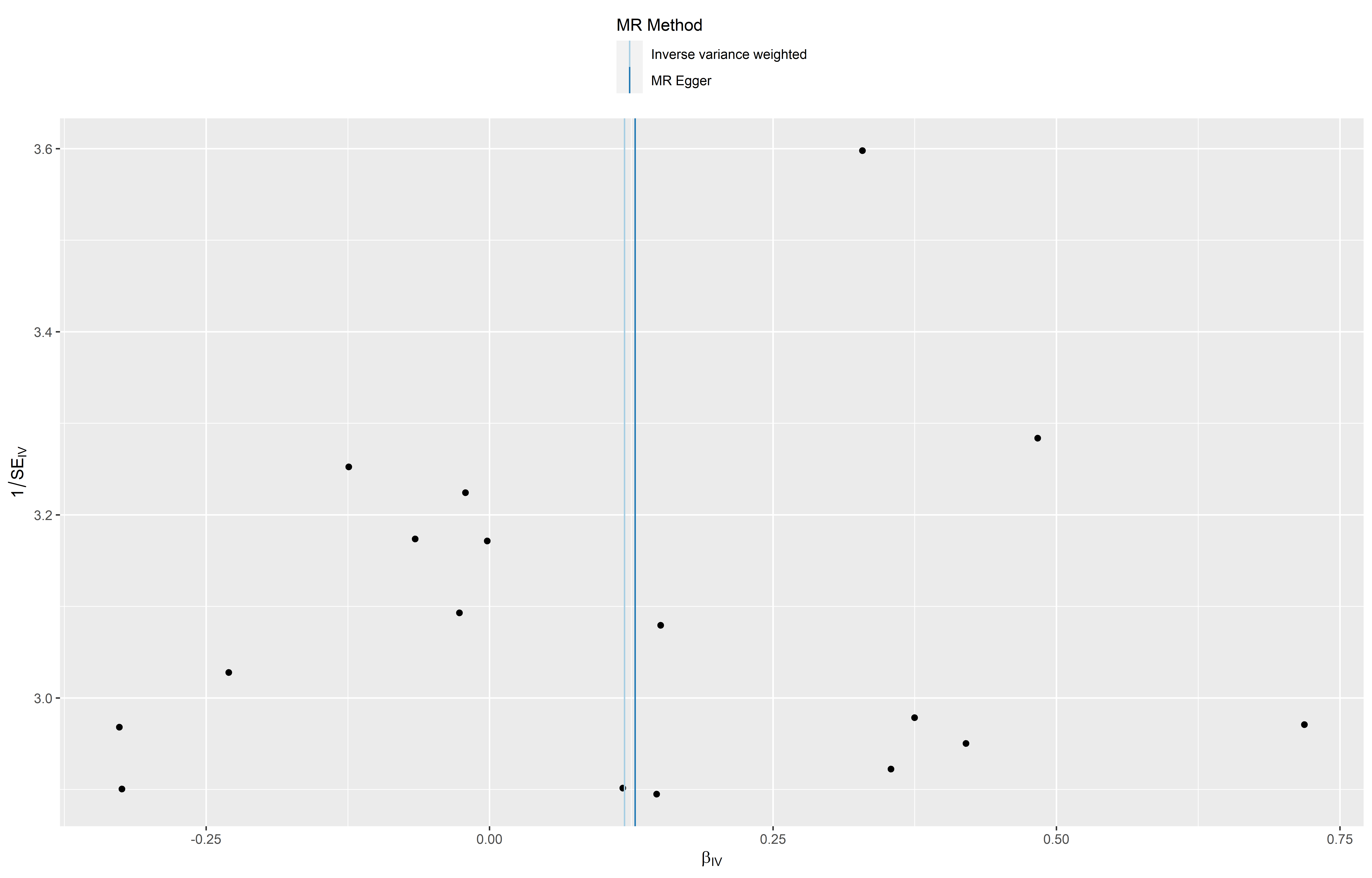

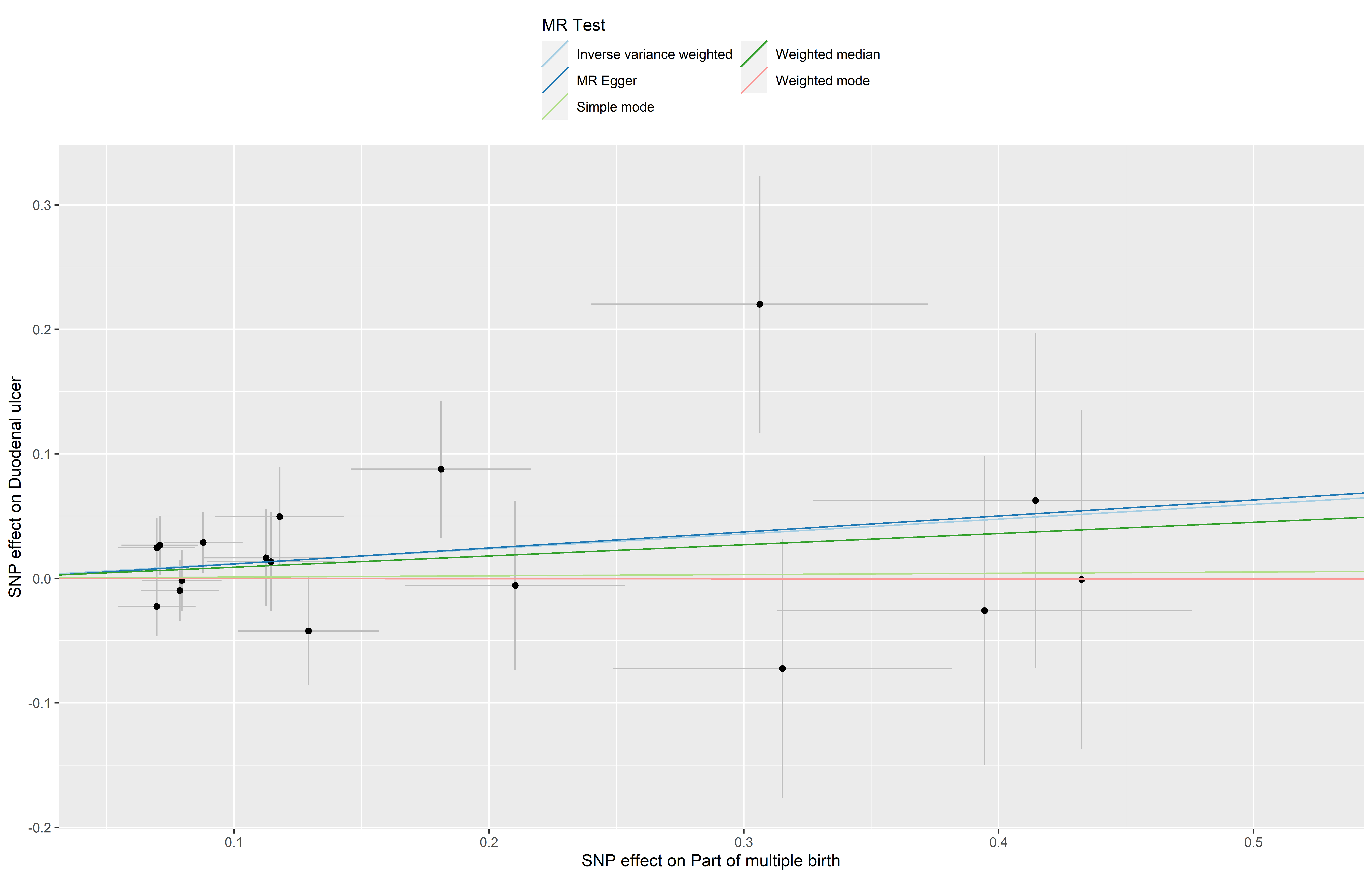


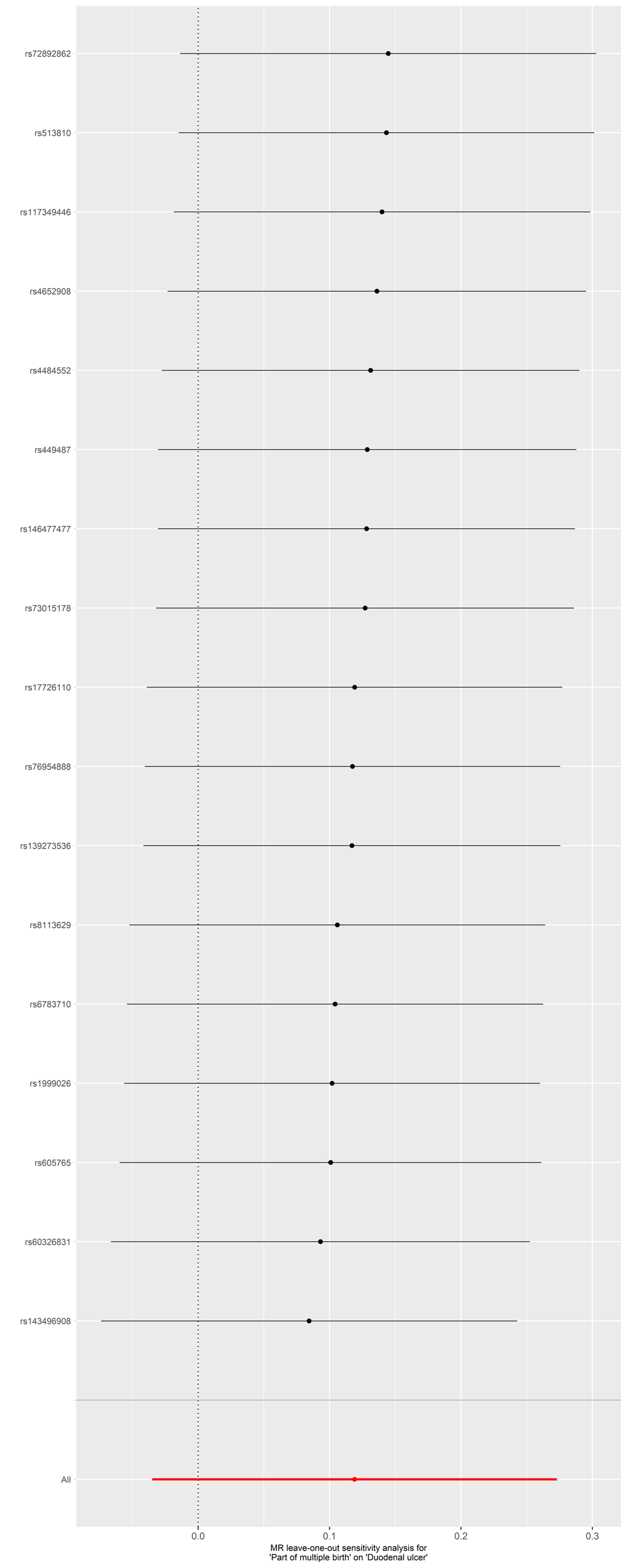


**Crohn disease – Finngen**


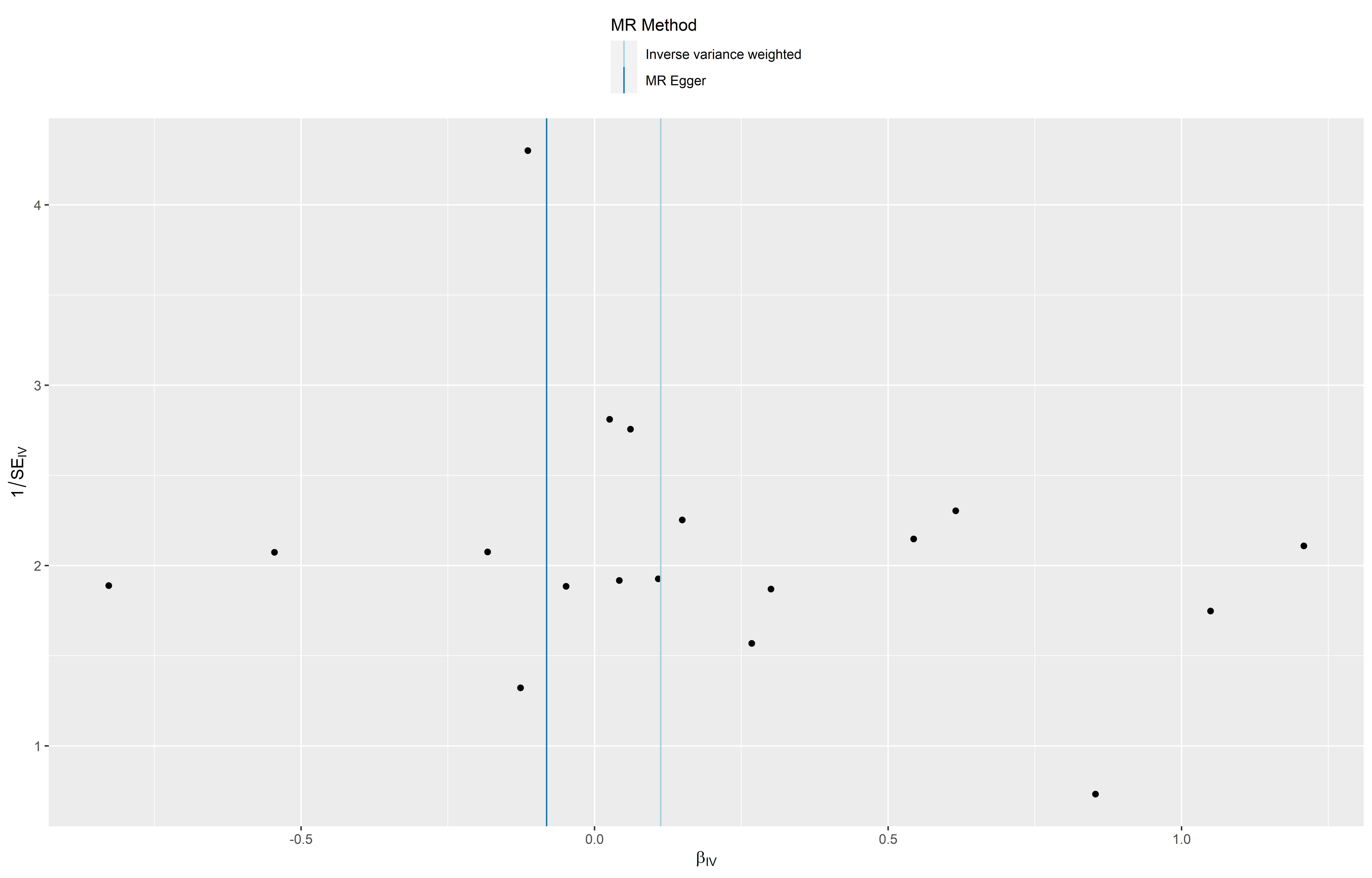

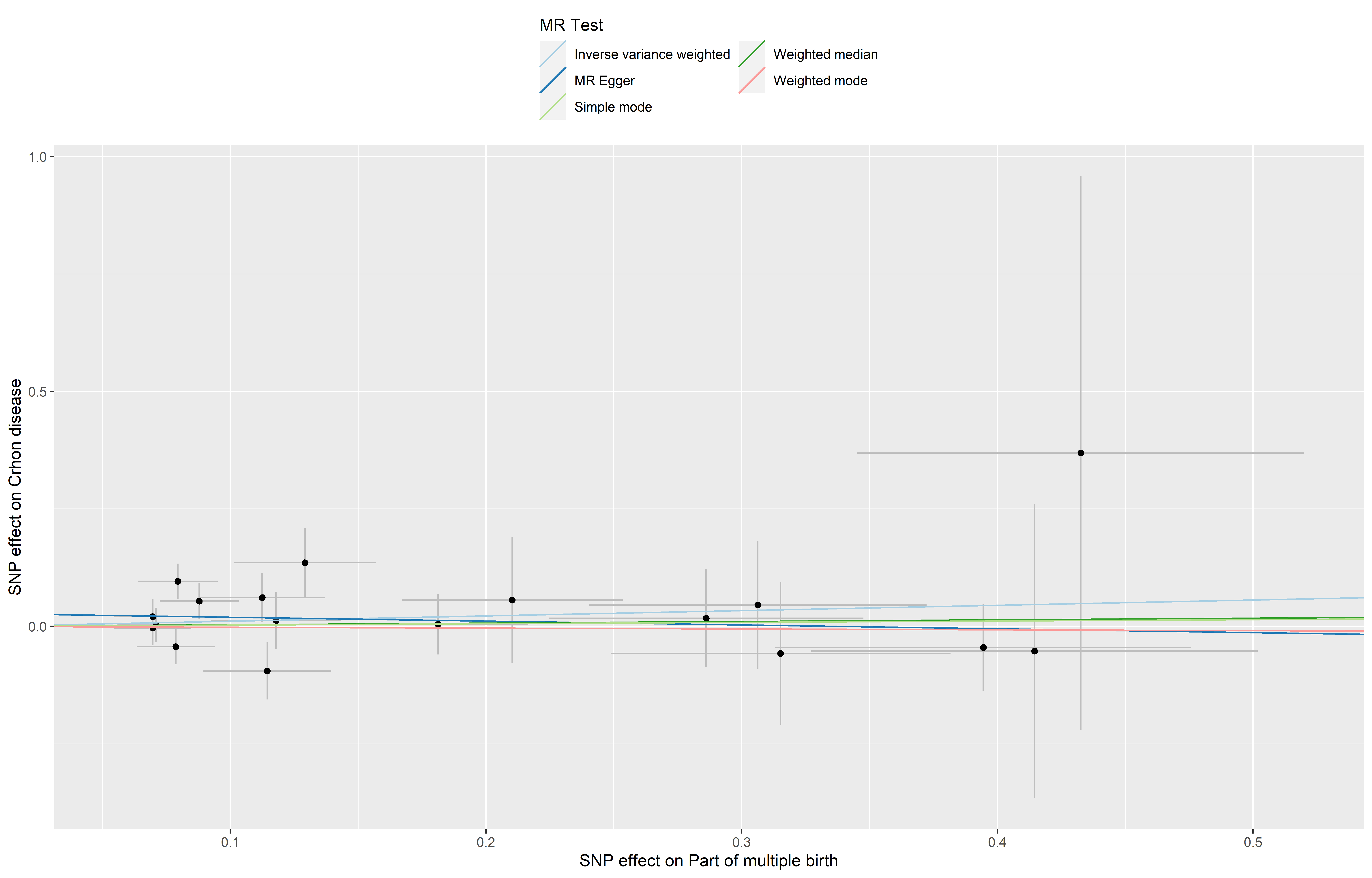


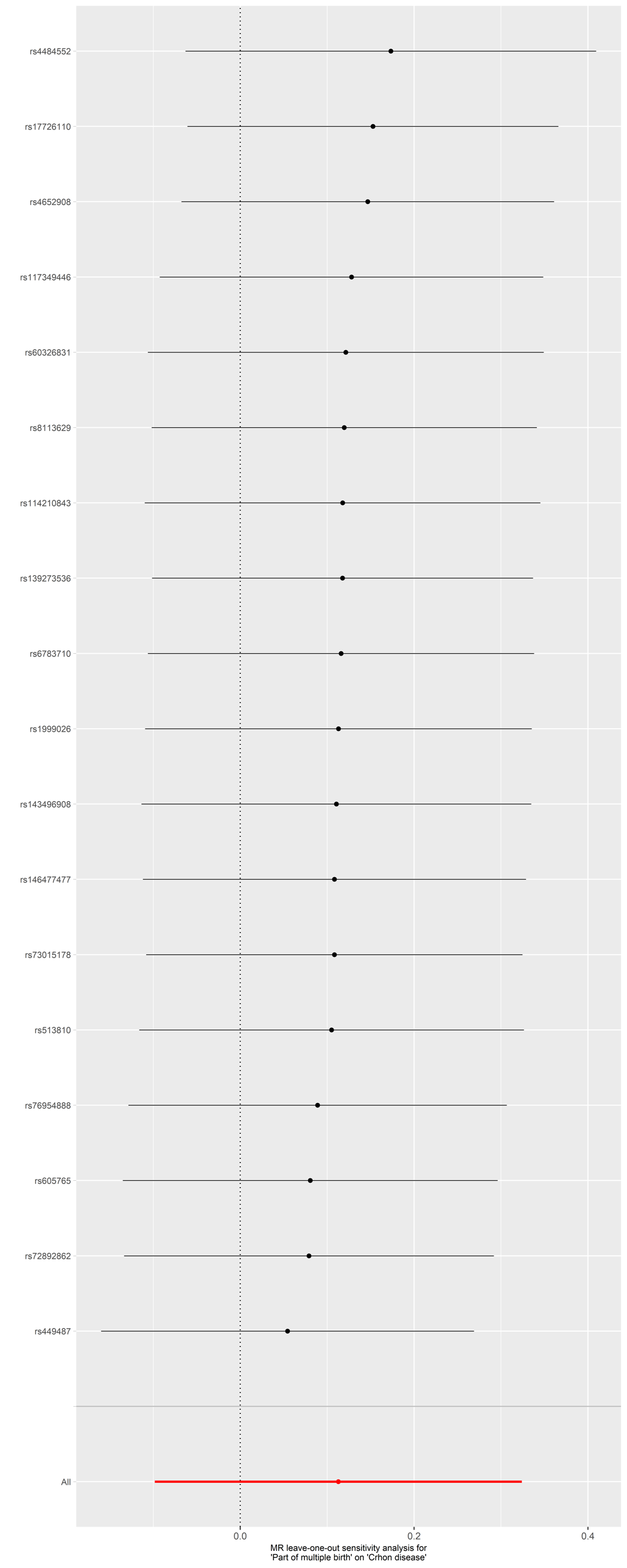


**Crohn disease – UK Biobank**


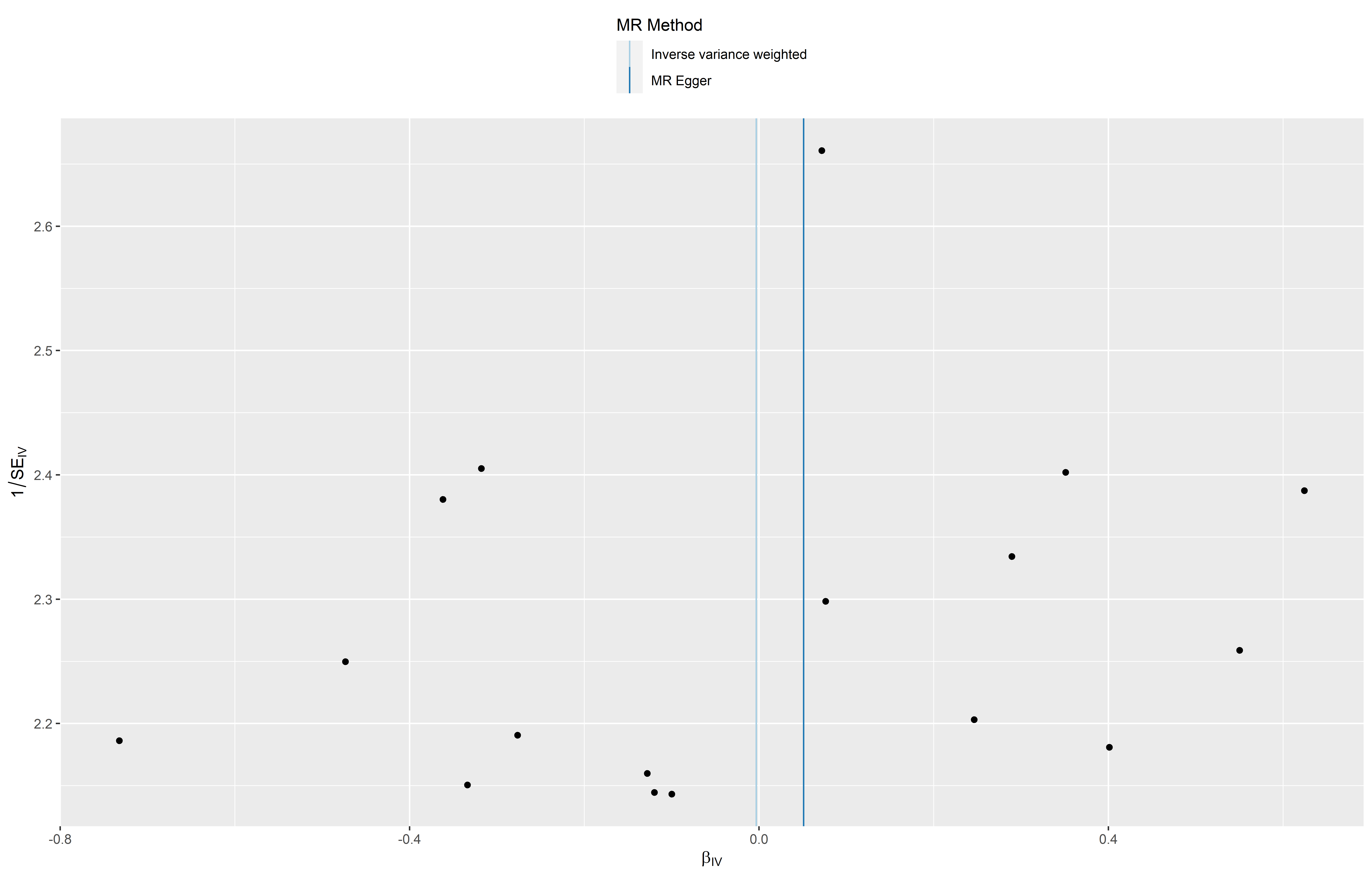

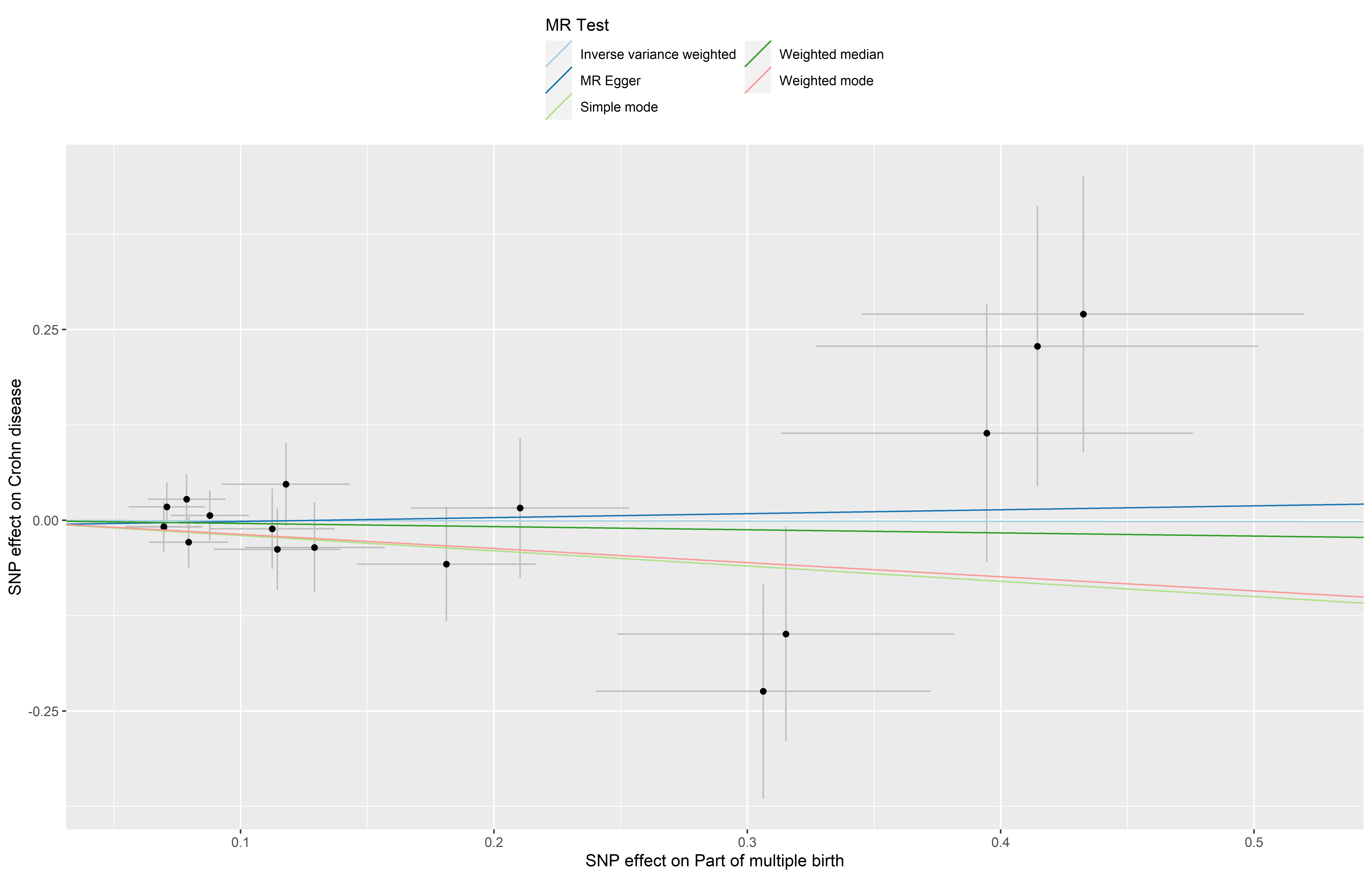


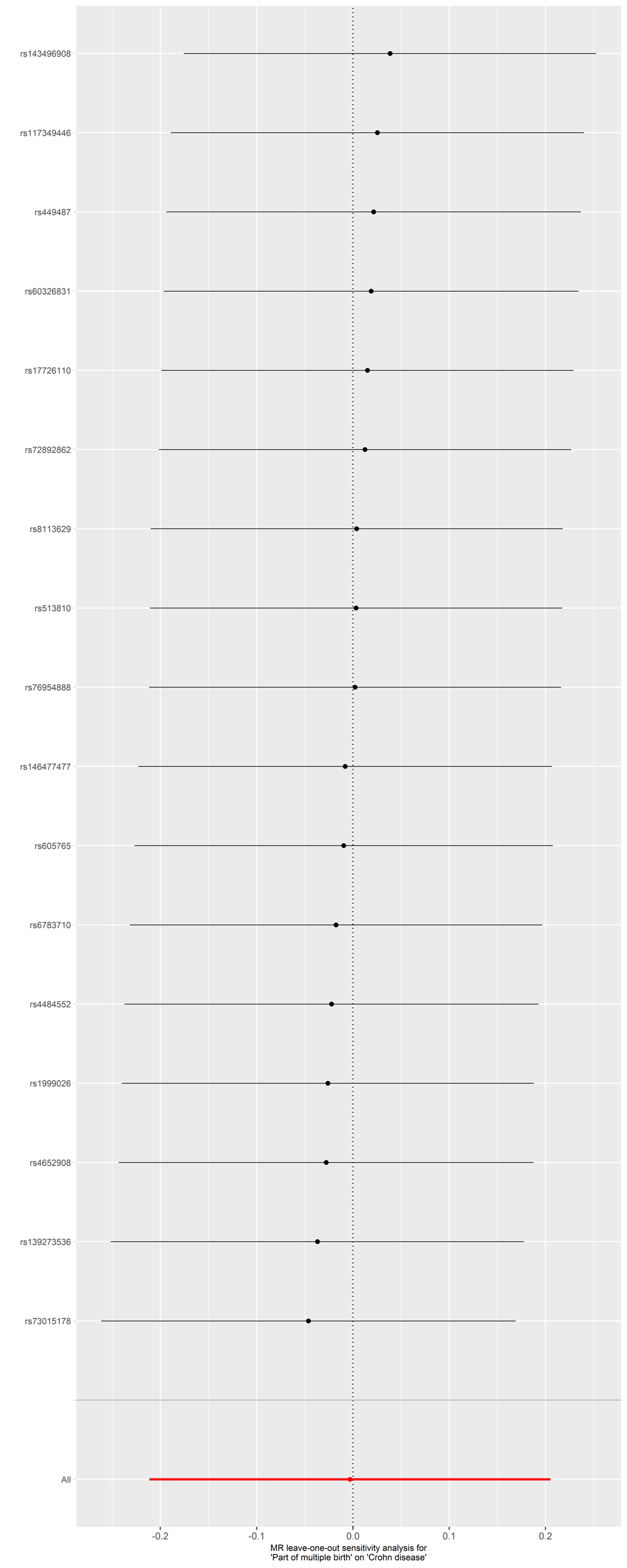


**Fibrosis and cirrhosis of liver – Finngen**


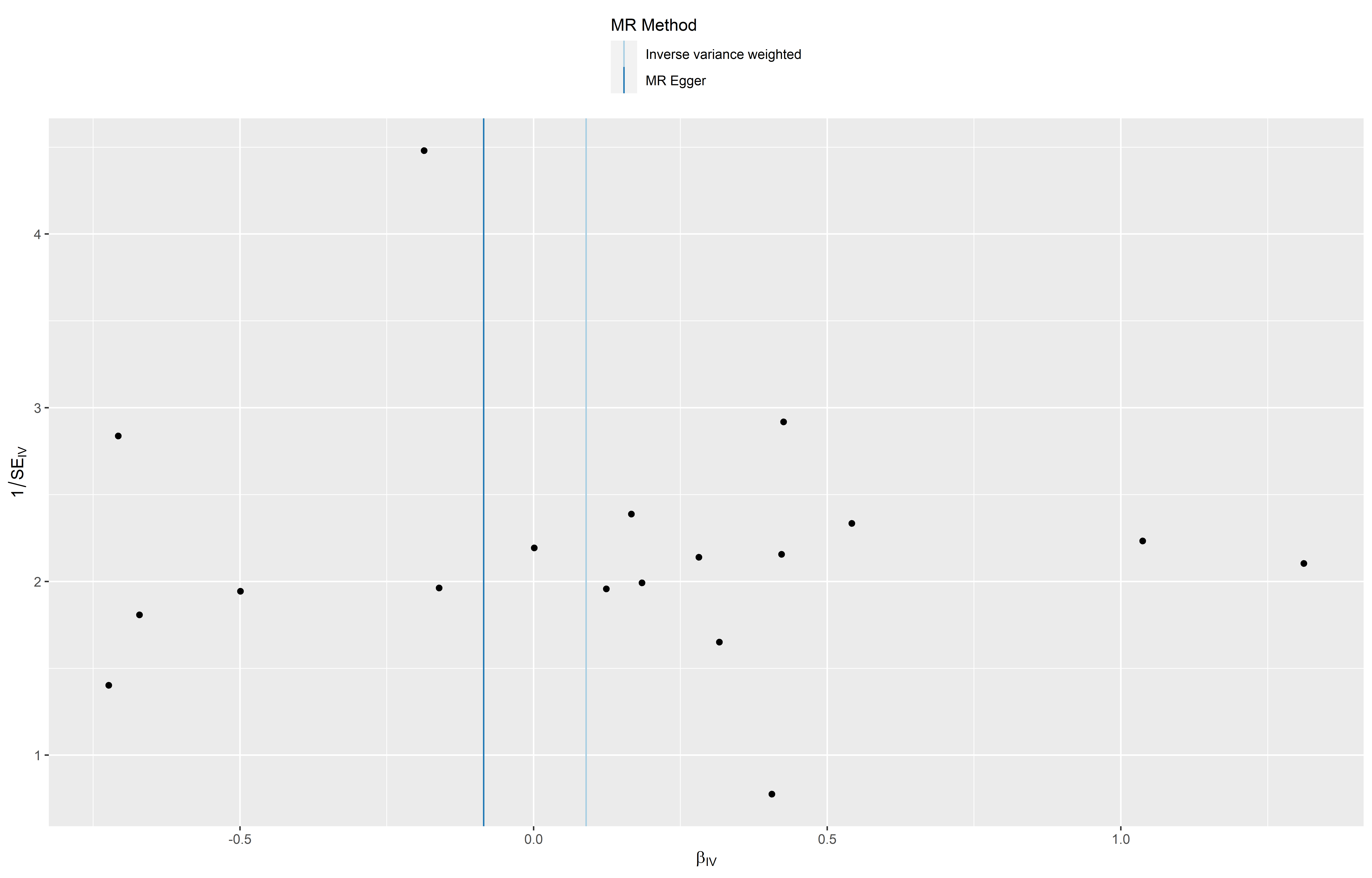

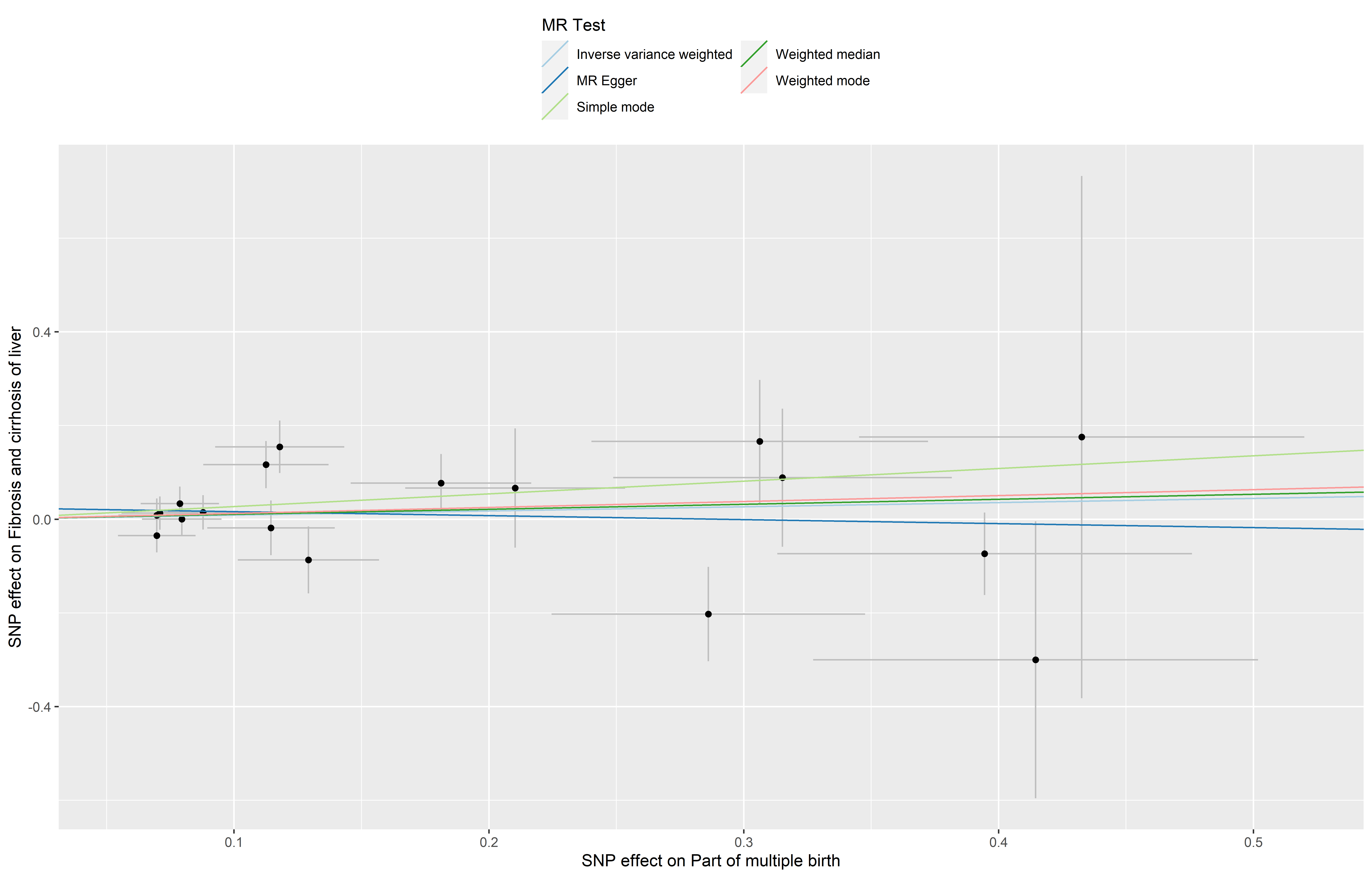


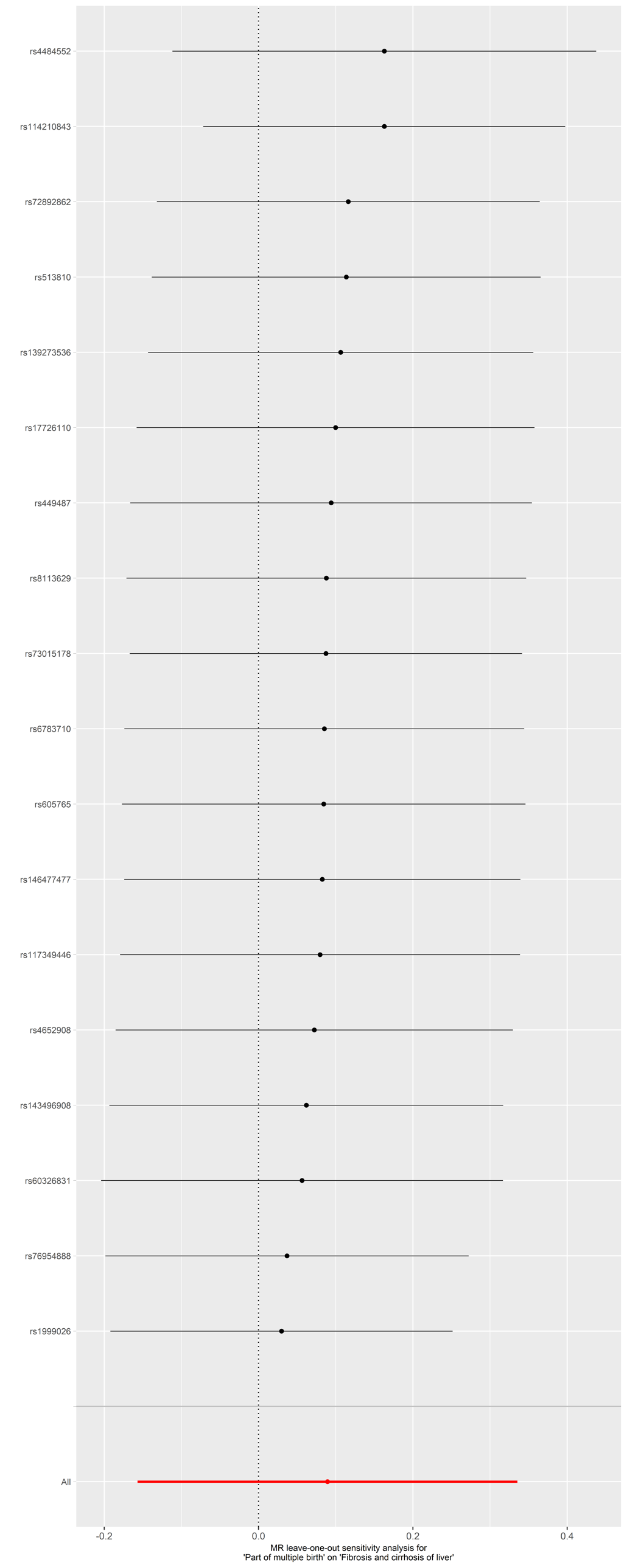


**Fibrosis and cirrhosis of liver – UK Biobank**


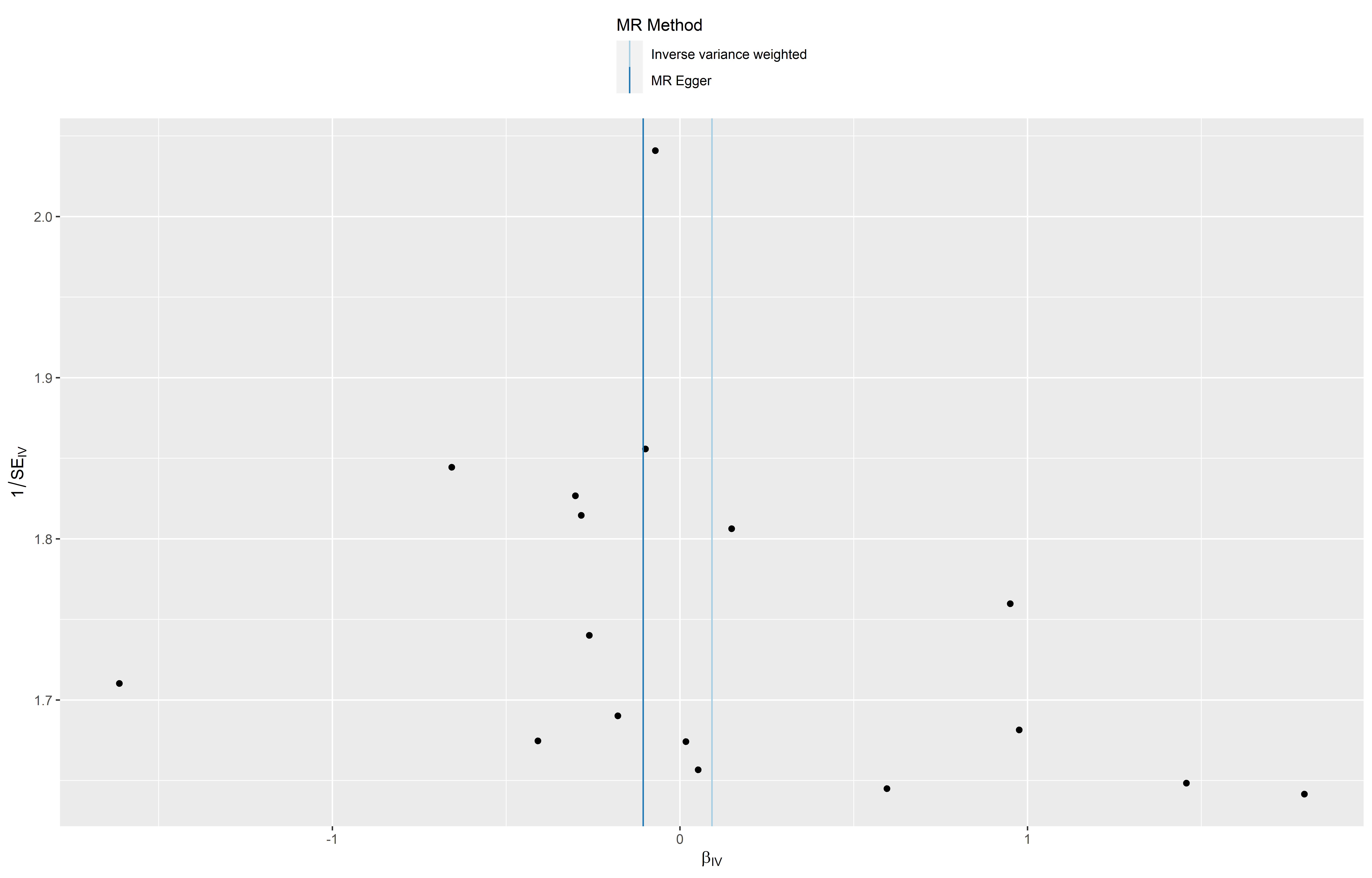

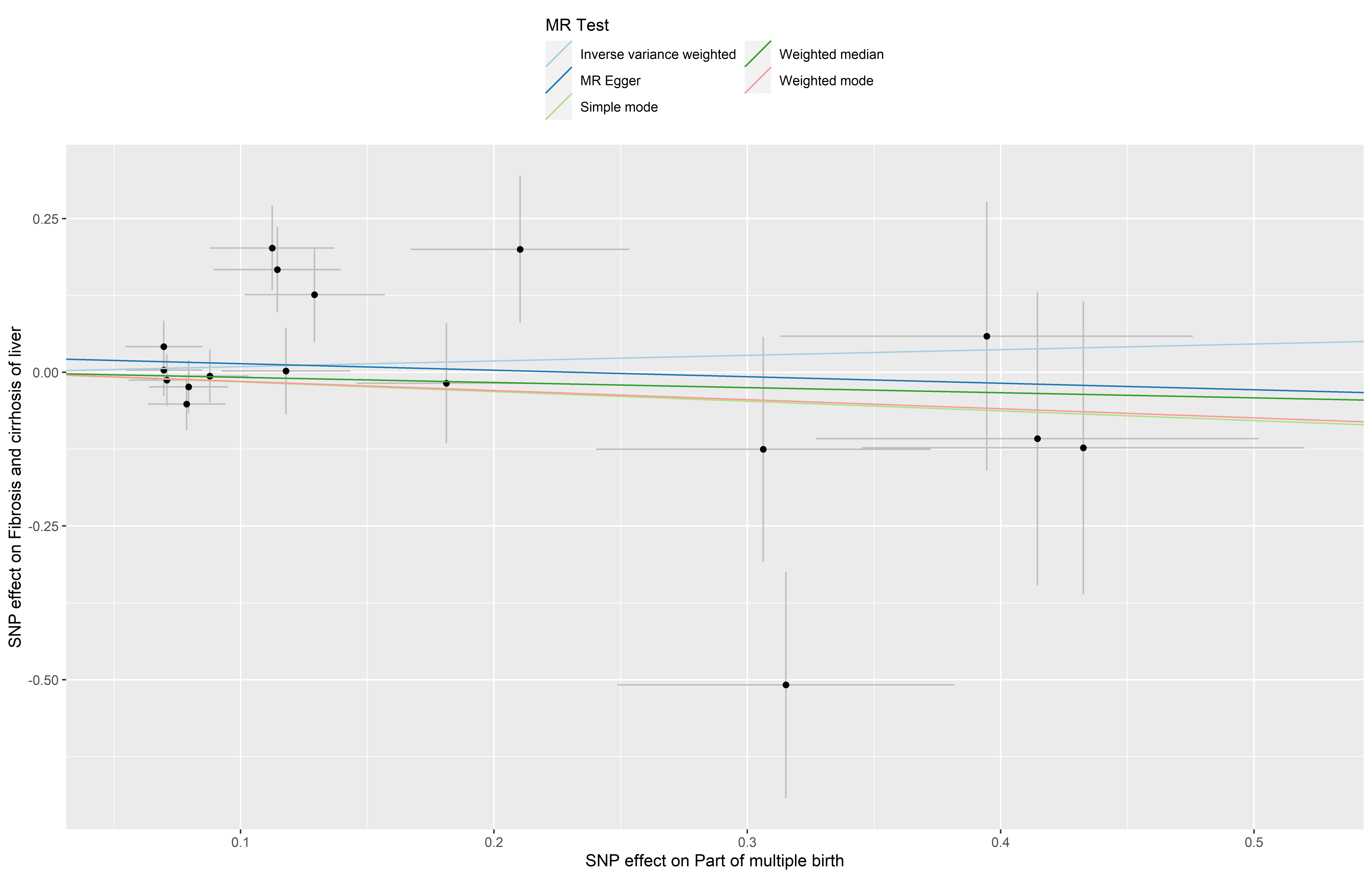


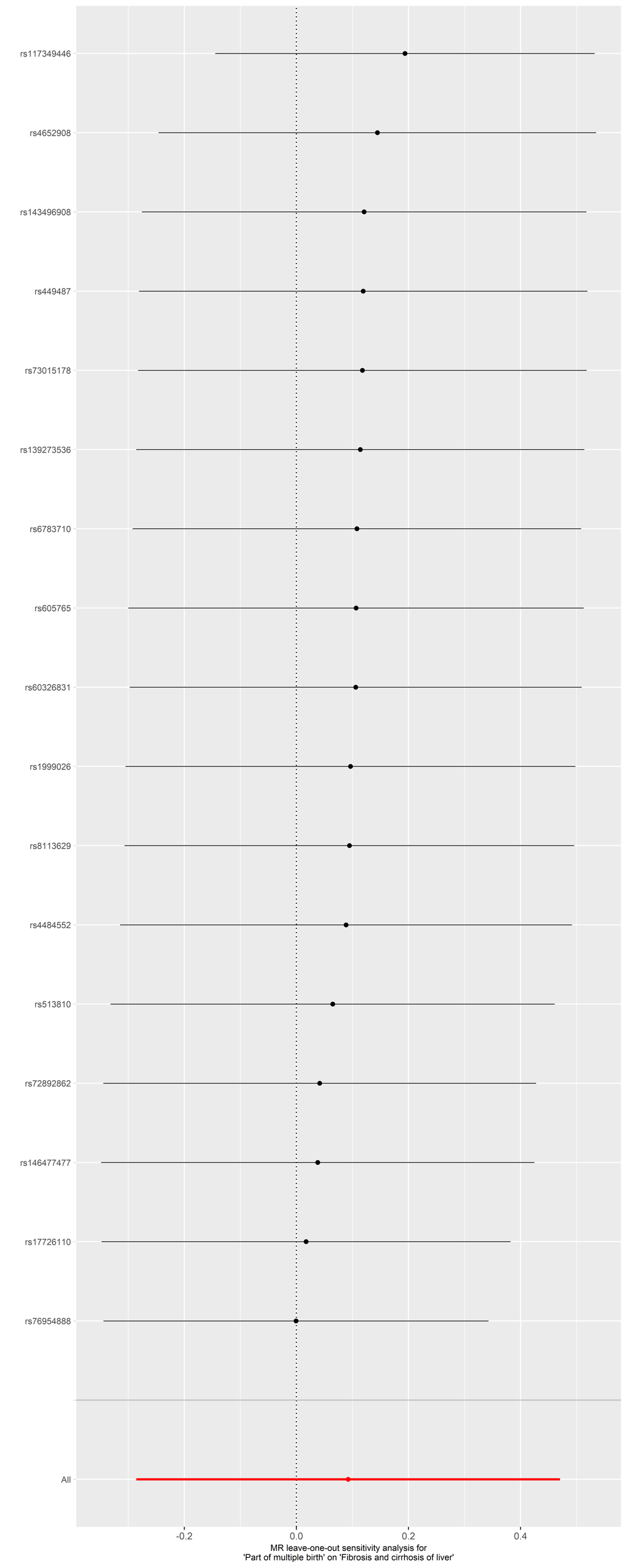


**Gastritis (chronic) – Finngen**


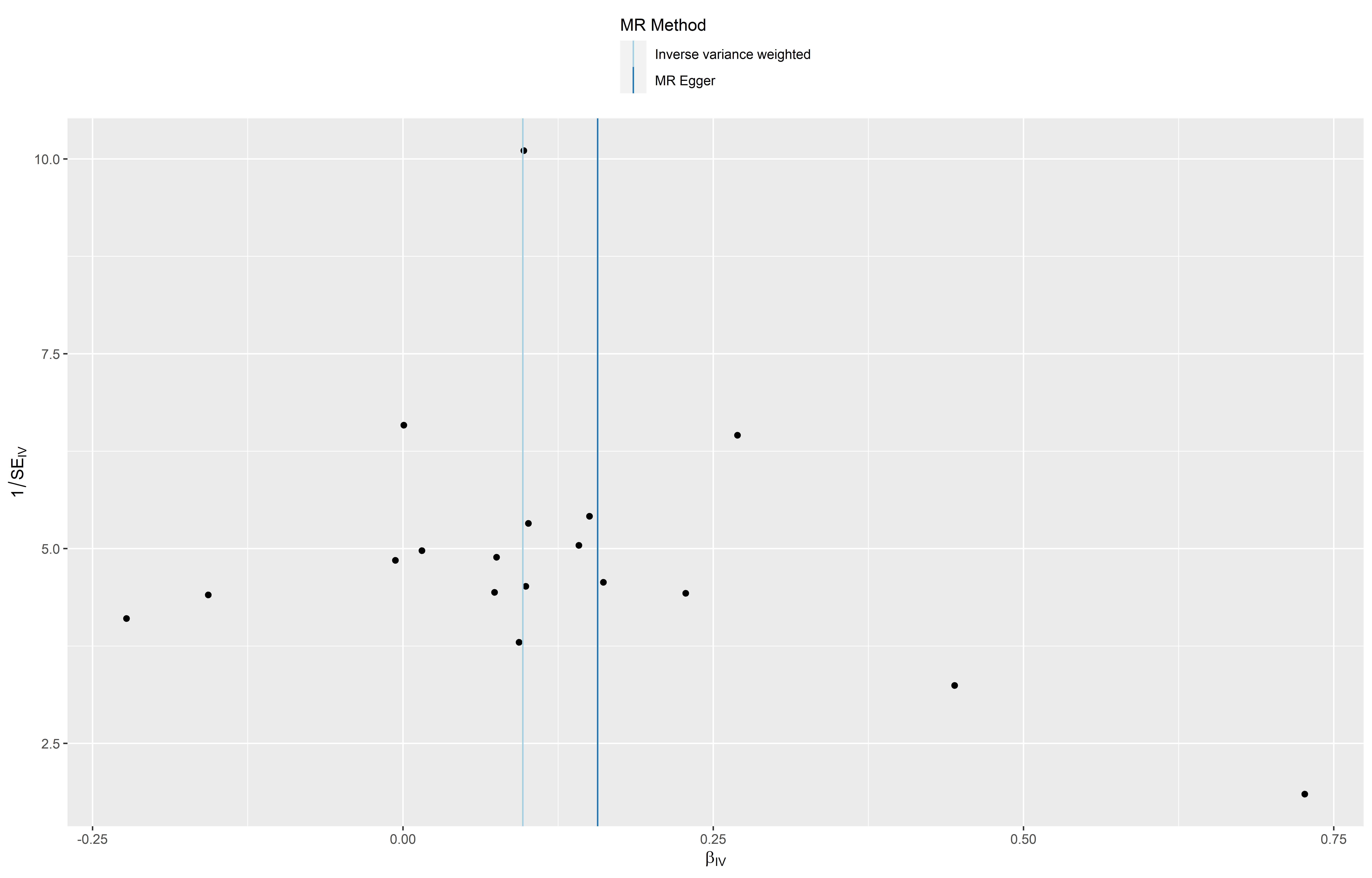

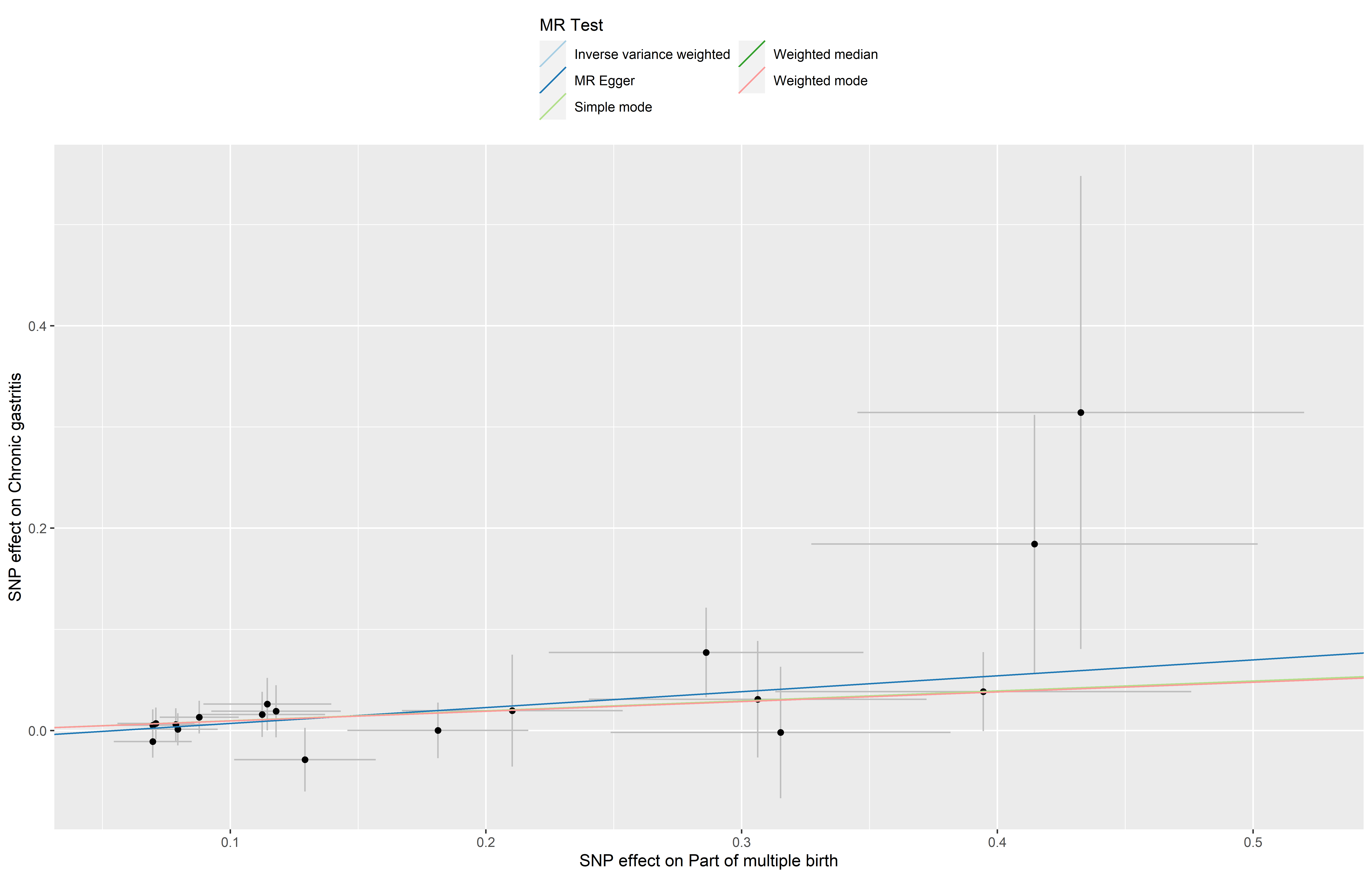


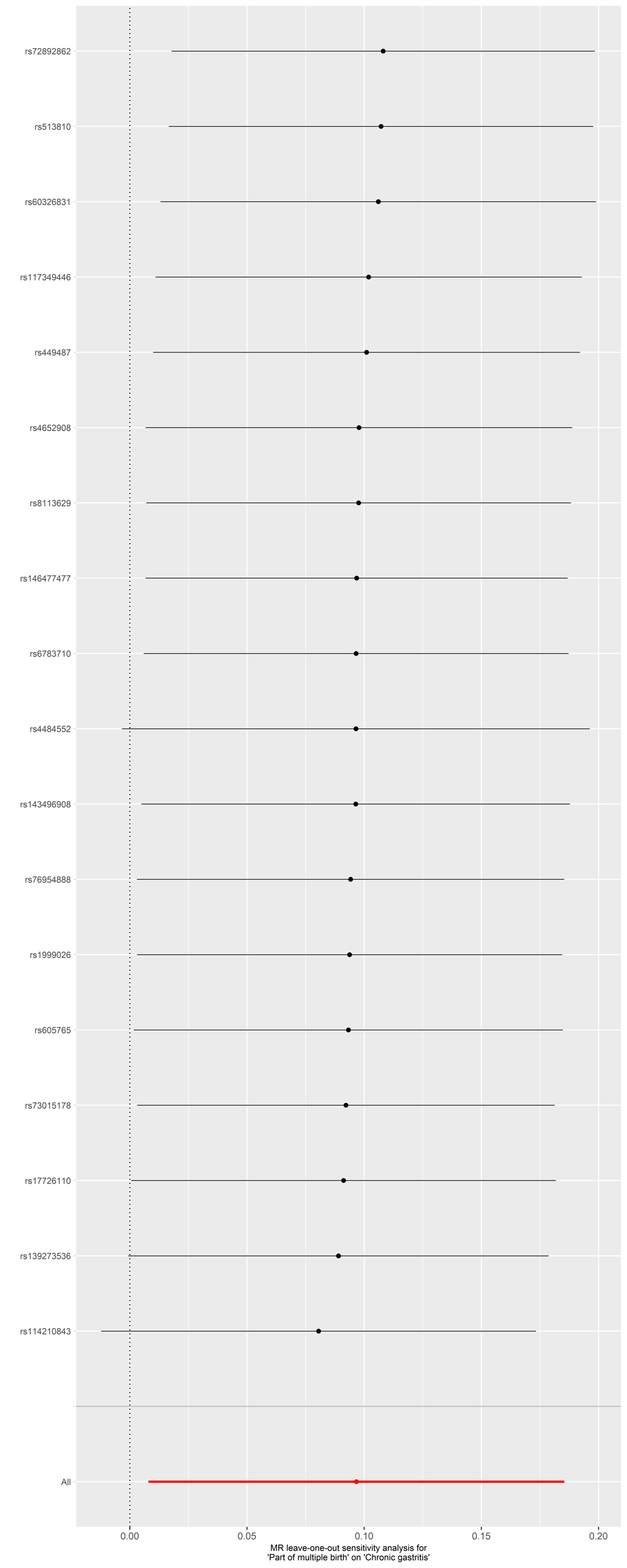


**Gastritis (acute) – Finngen**


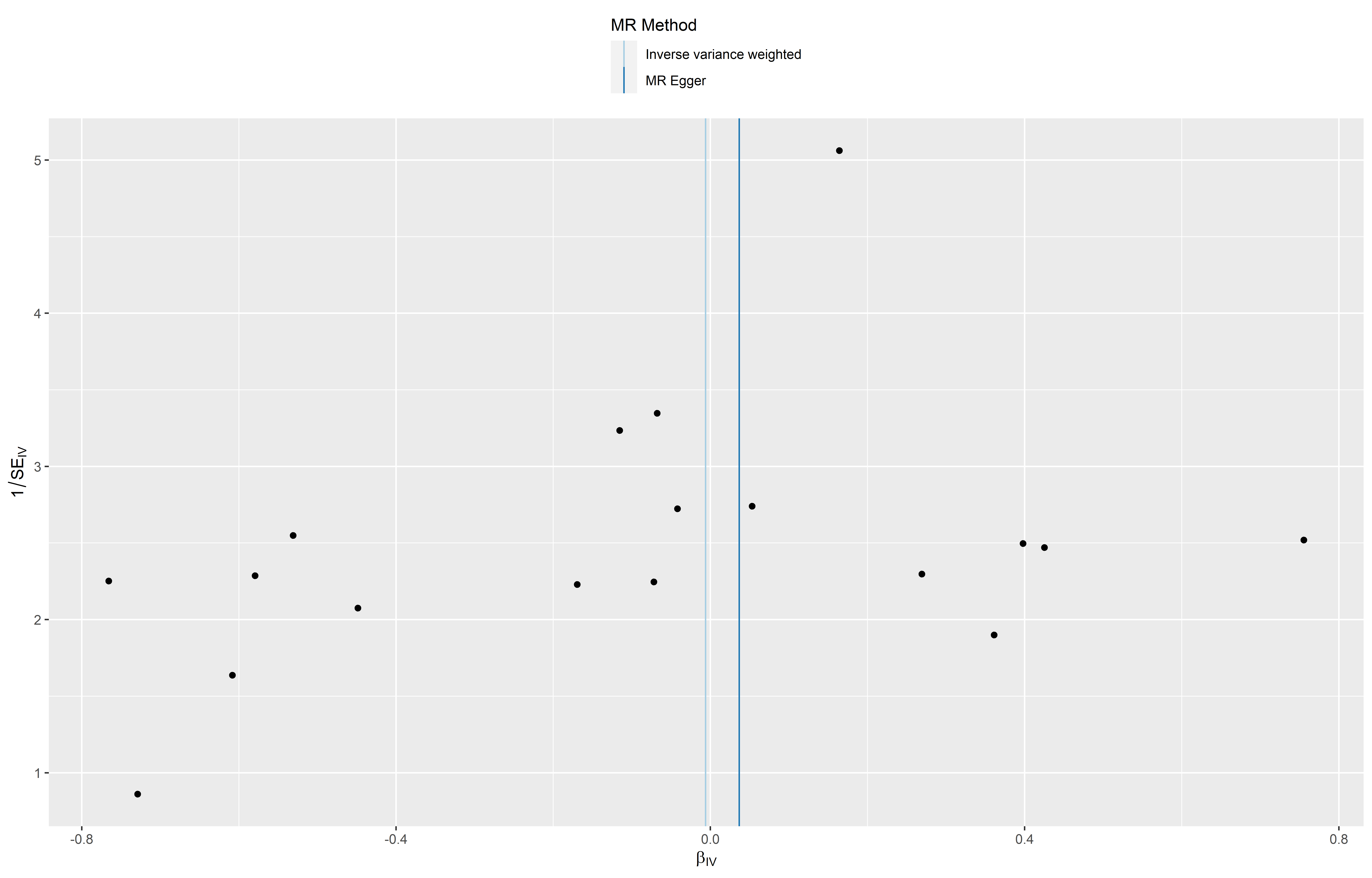

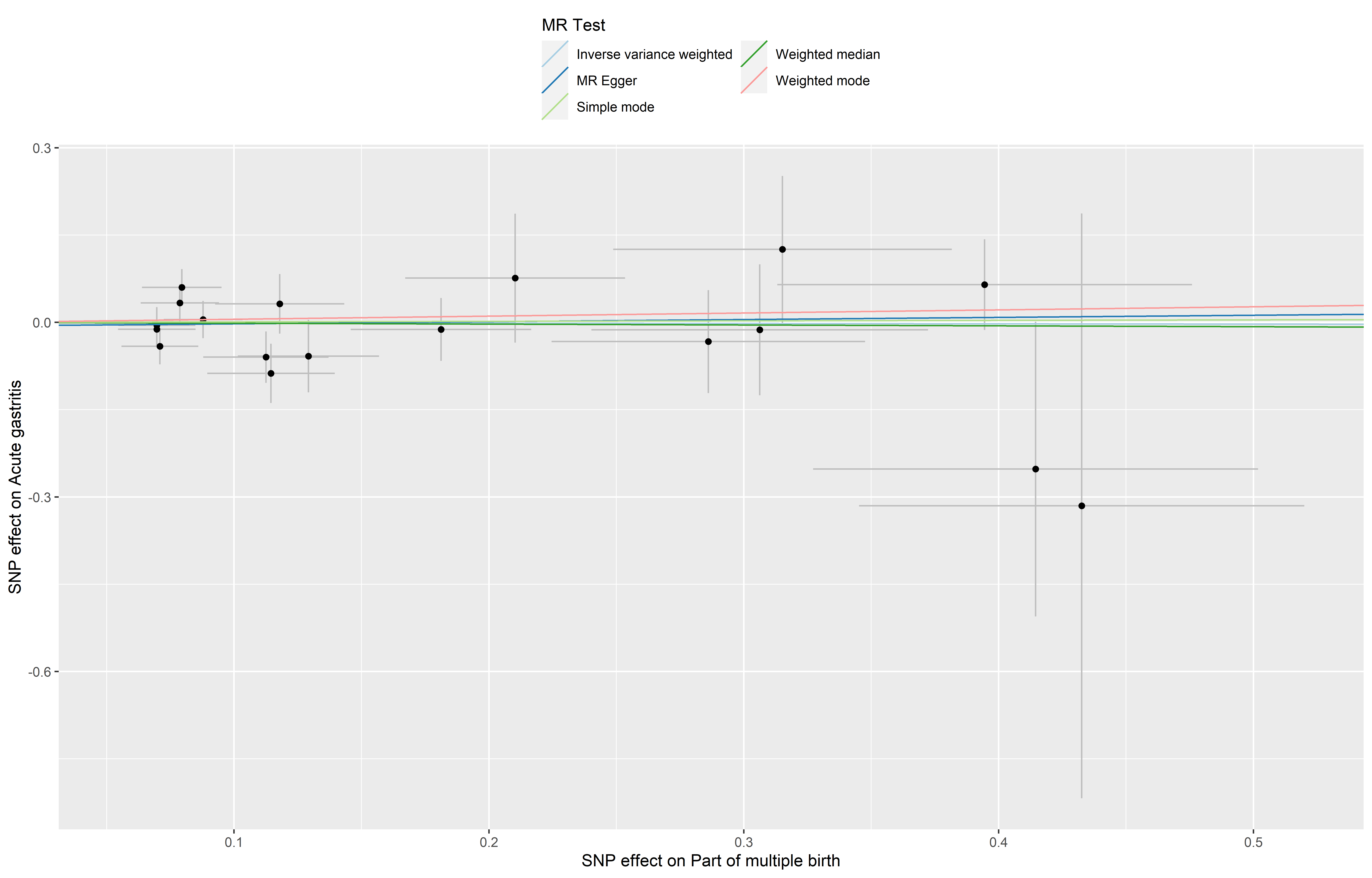


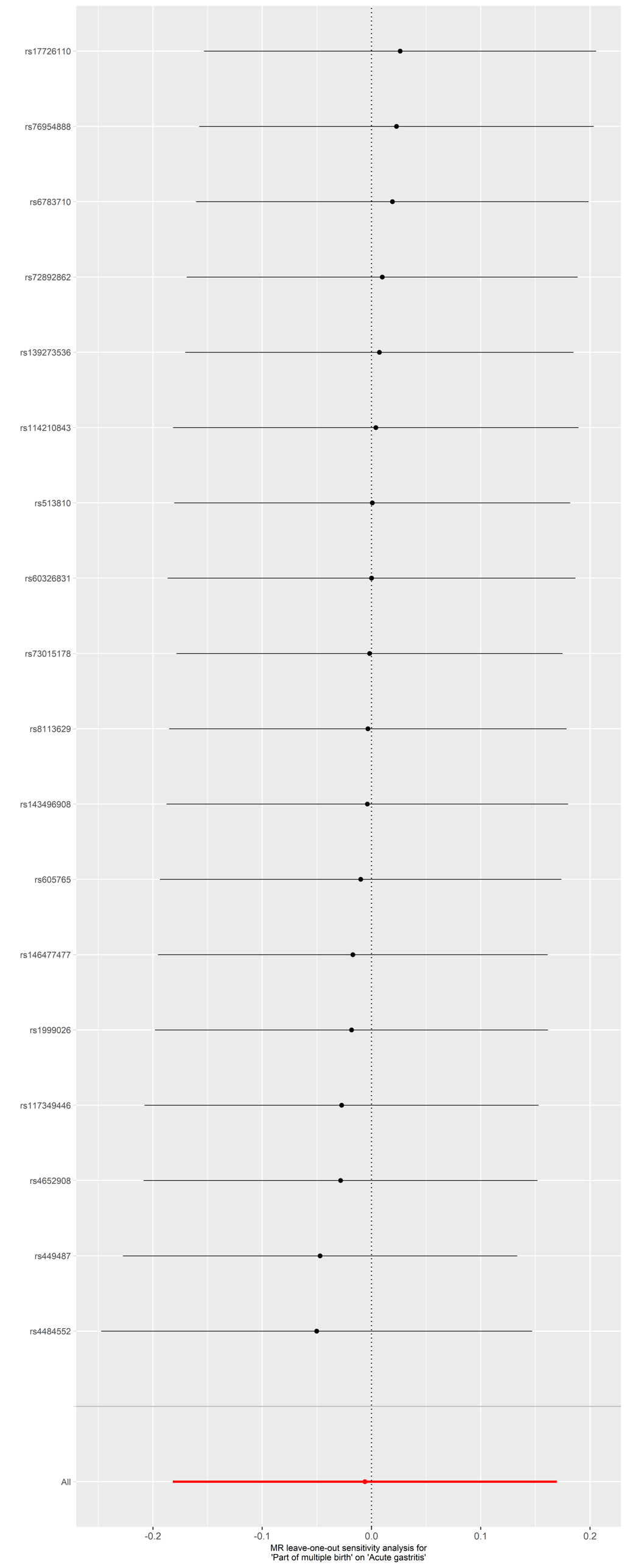


**Gastritis (acute) – UK Biobank**


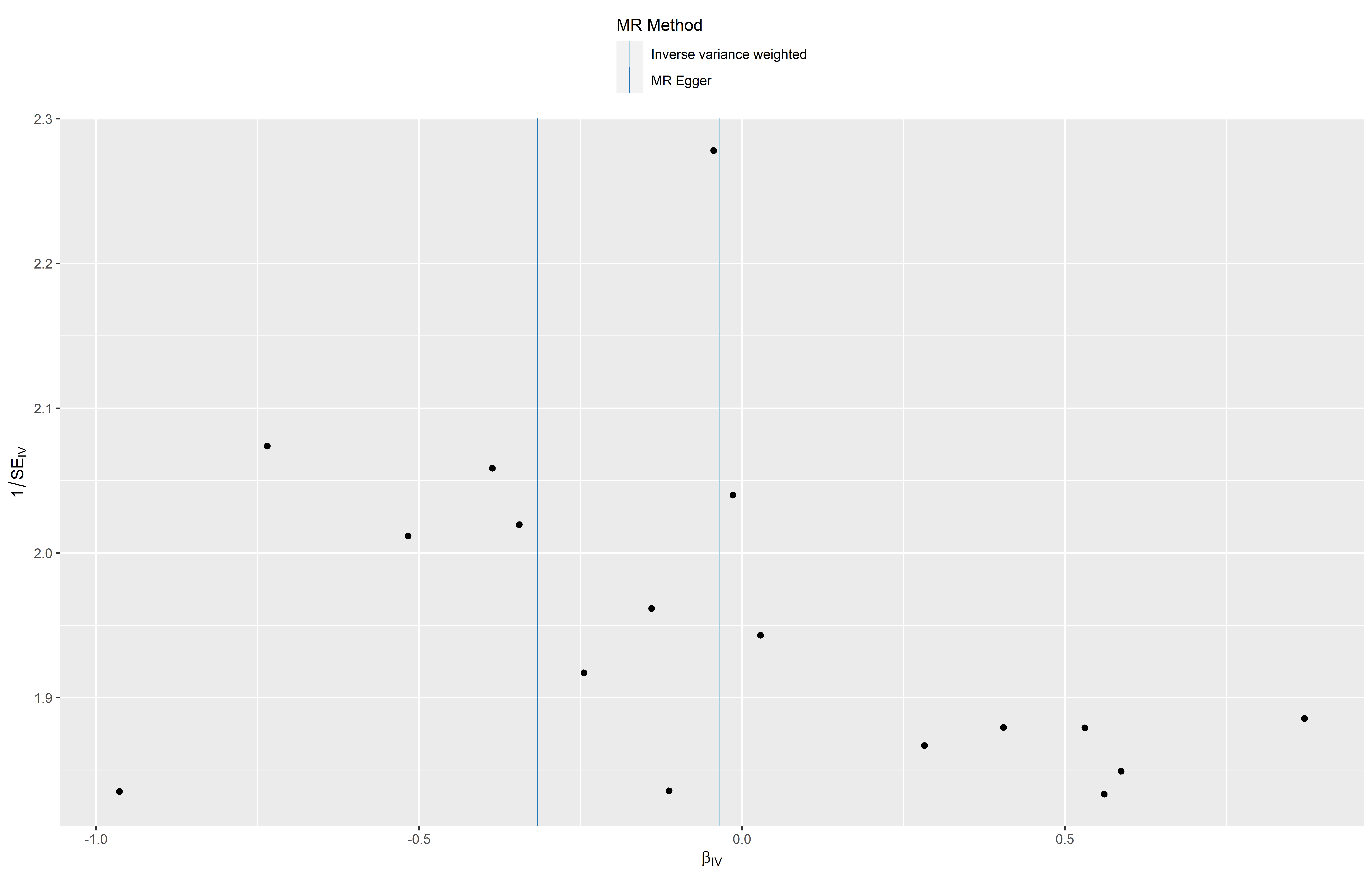

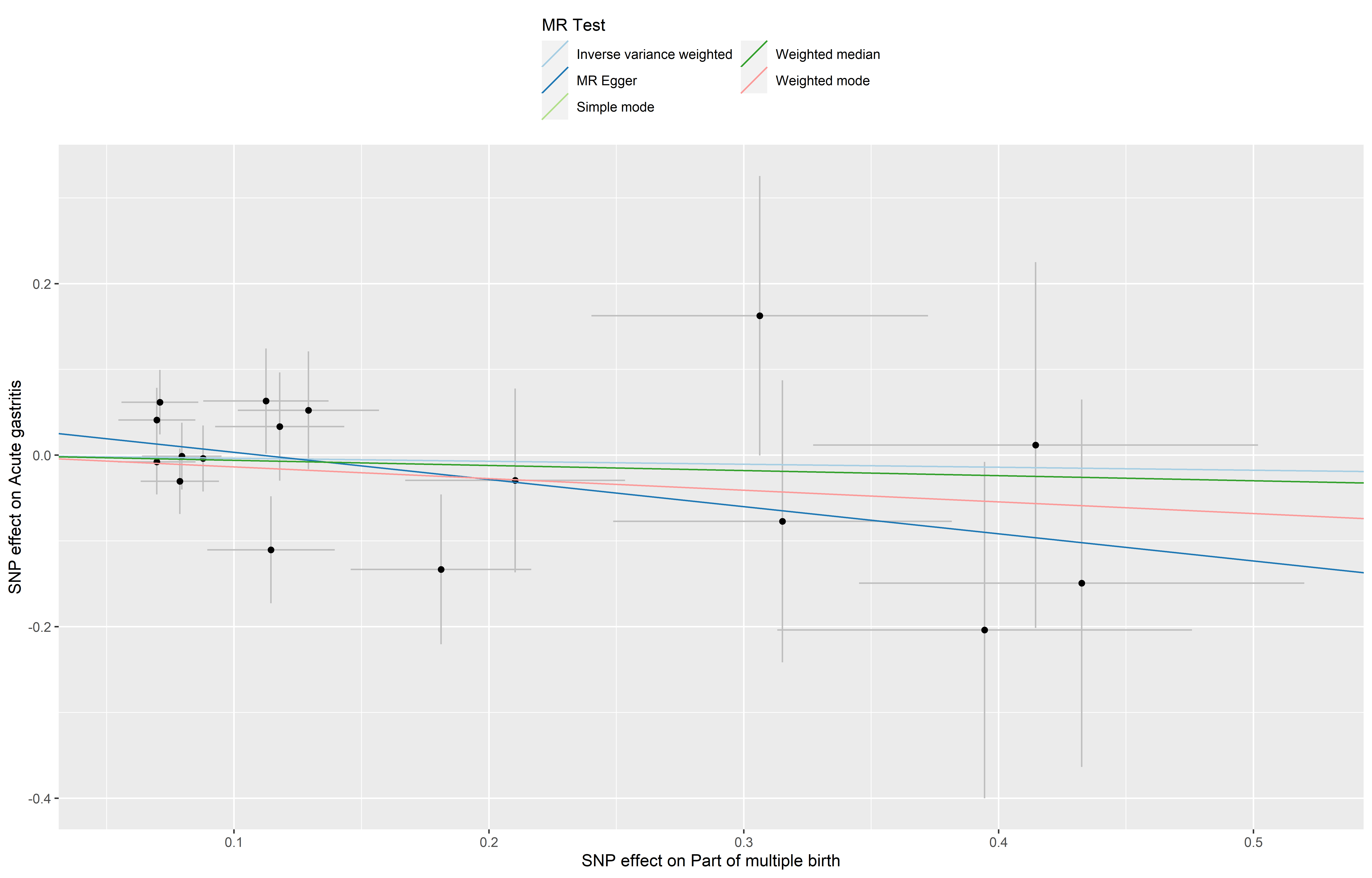


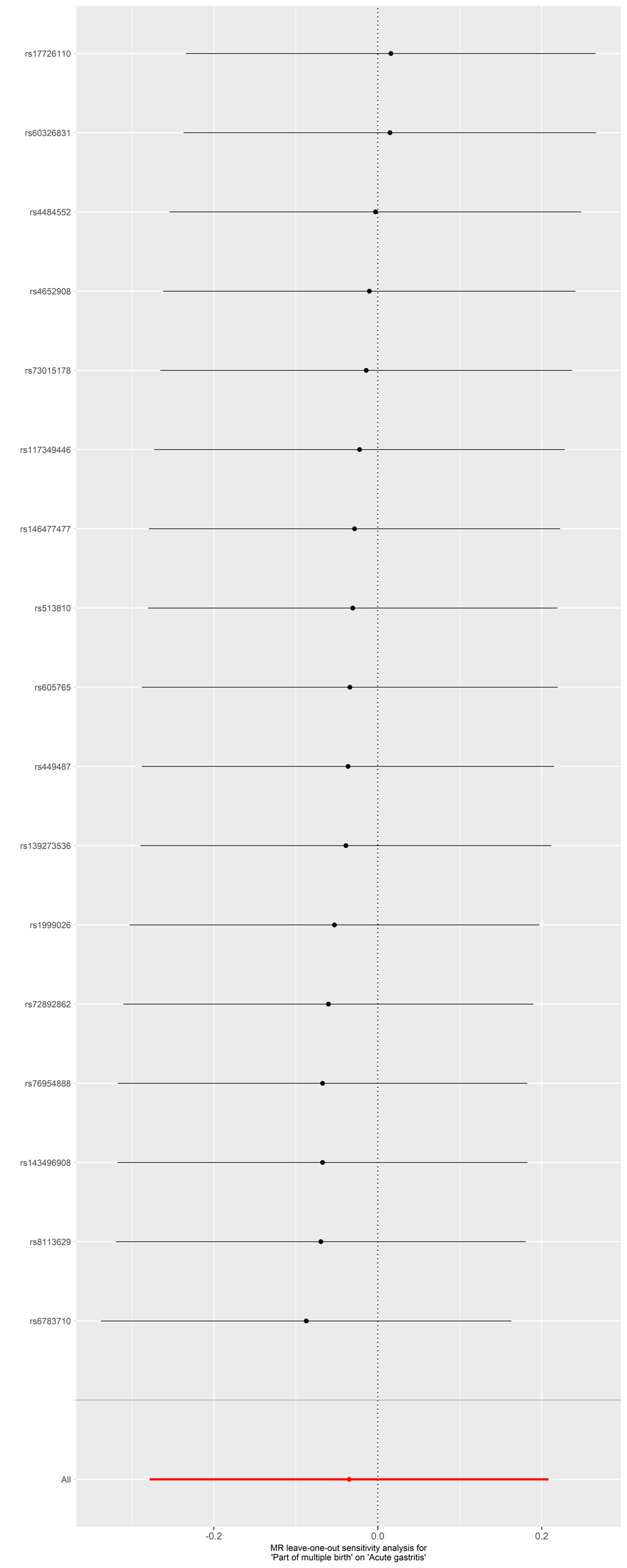

Supplement: Supplementary file 13 — Additional file 13: Material S5. The scatter plot, funnel plot and leave-one-out plot for the MR analysis of multiple birth and digestive system disease. [file 12967_2023_4423_MOESM13_ESM.docx]
