# Supplementary material for: Long-term outcomes of offspring from multiple gestations: a two-sample Mendelian randomization study on multi-system diseases using UK Biobank and FinnGen databases
Source: J Transl Med. 2023 Sep 8;21:608. doi: 10.1186/s12967-023-04423-w (PMC10492369; doi:10.1186/s12967-023-04423-w)

**Thyrotoxicosis – Finngen**


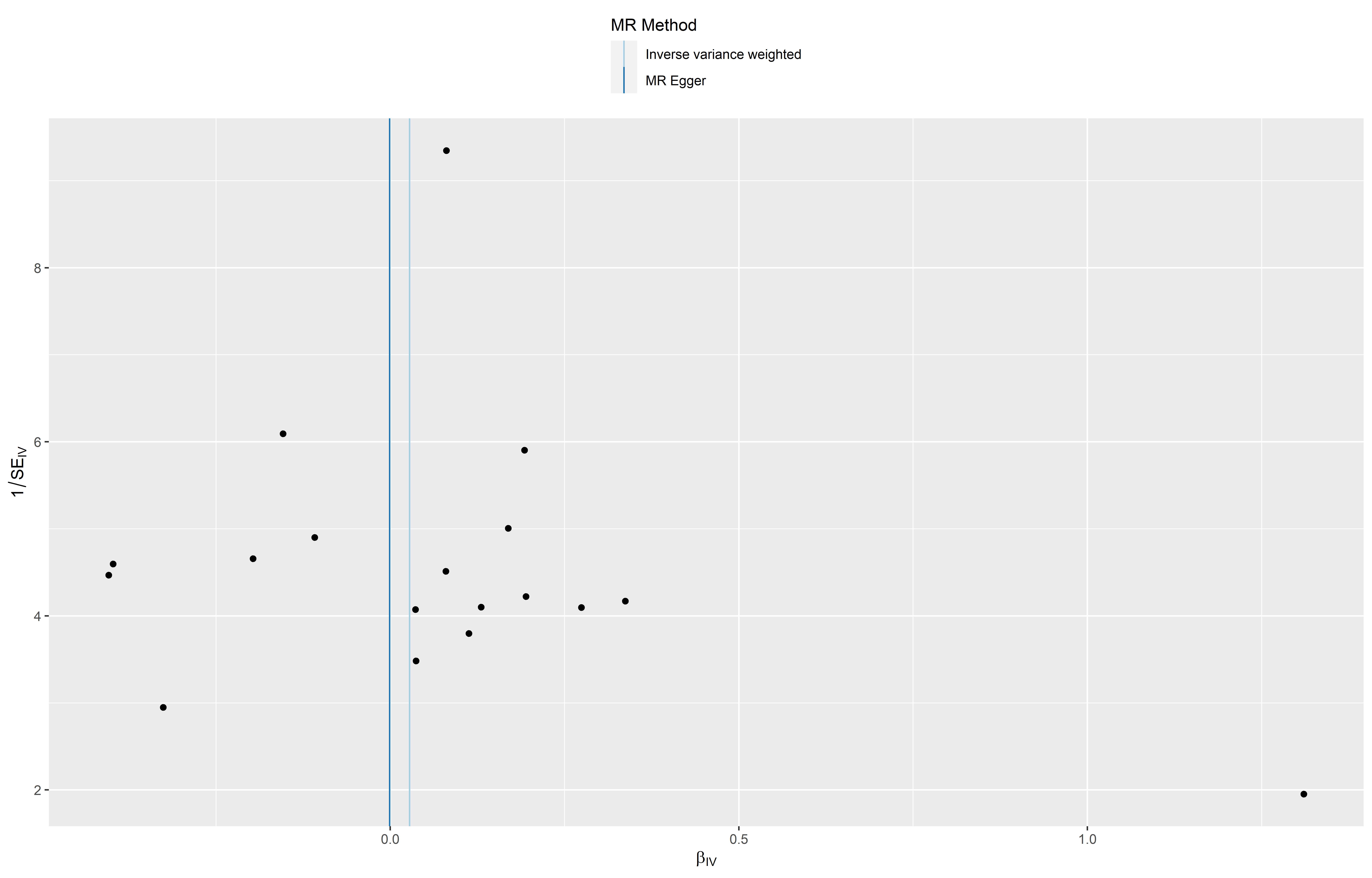

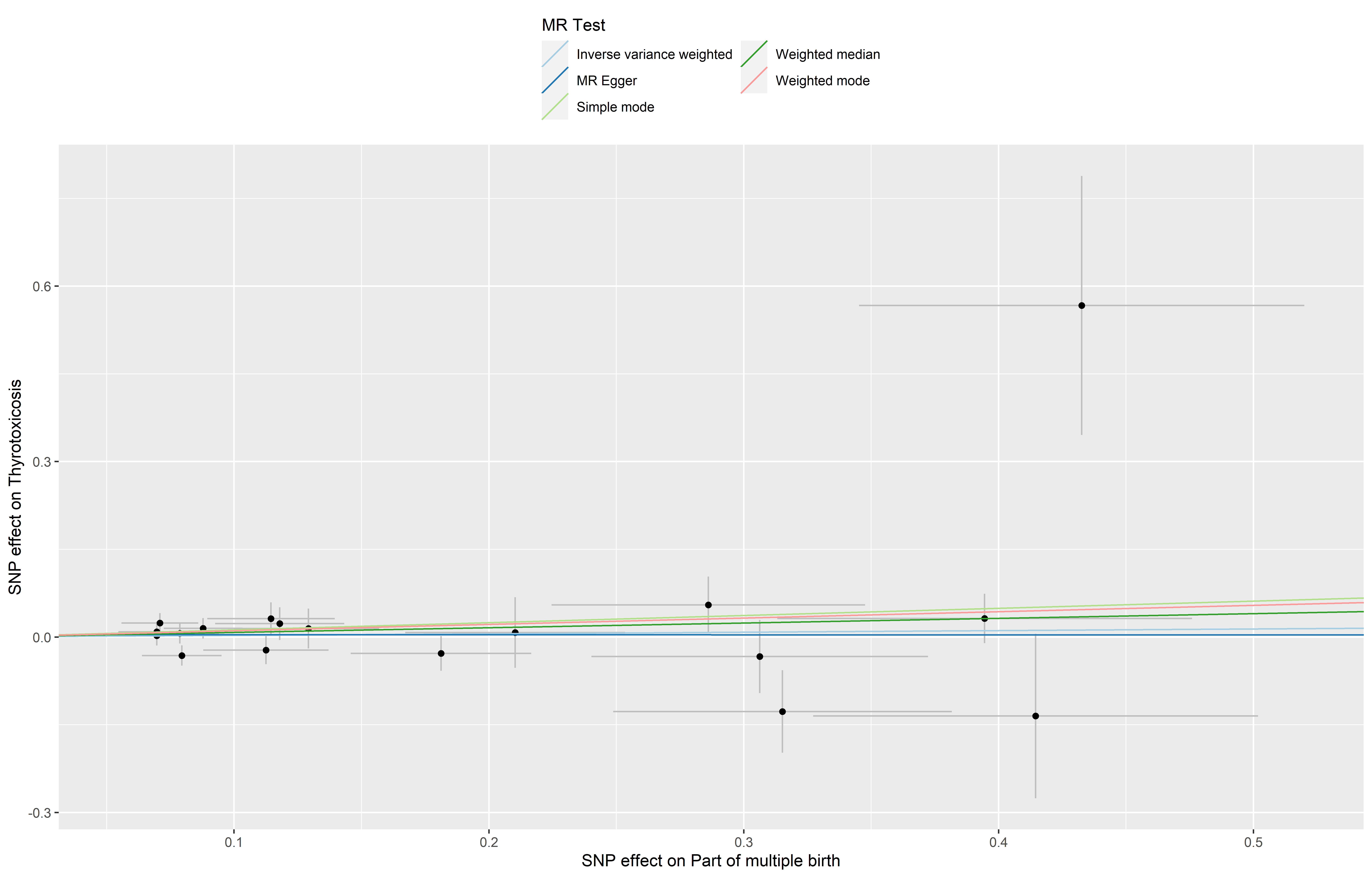


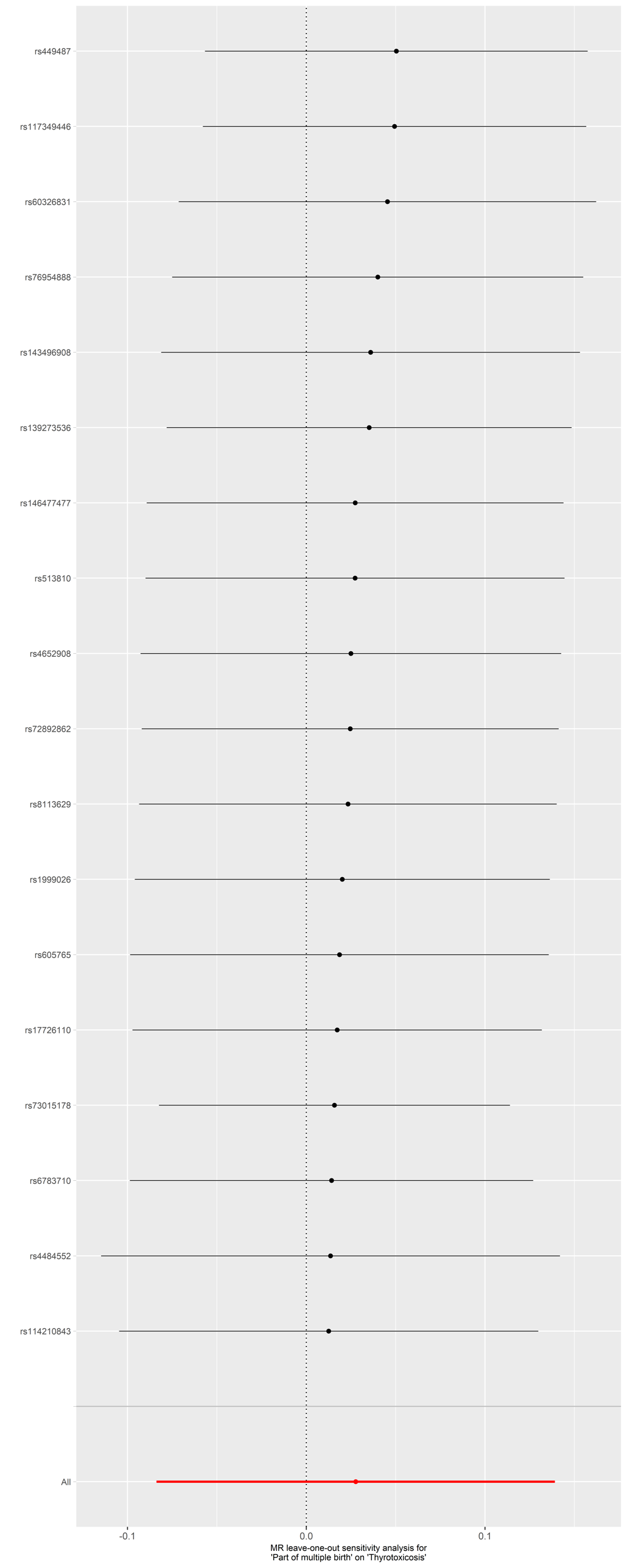


**Thyrotoxicosis – UK Biobank**


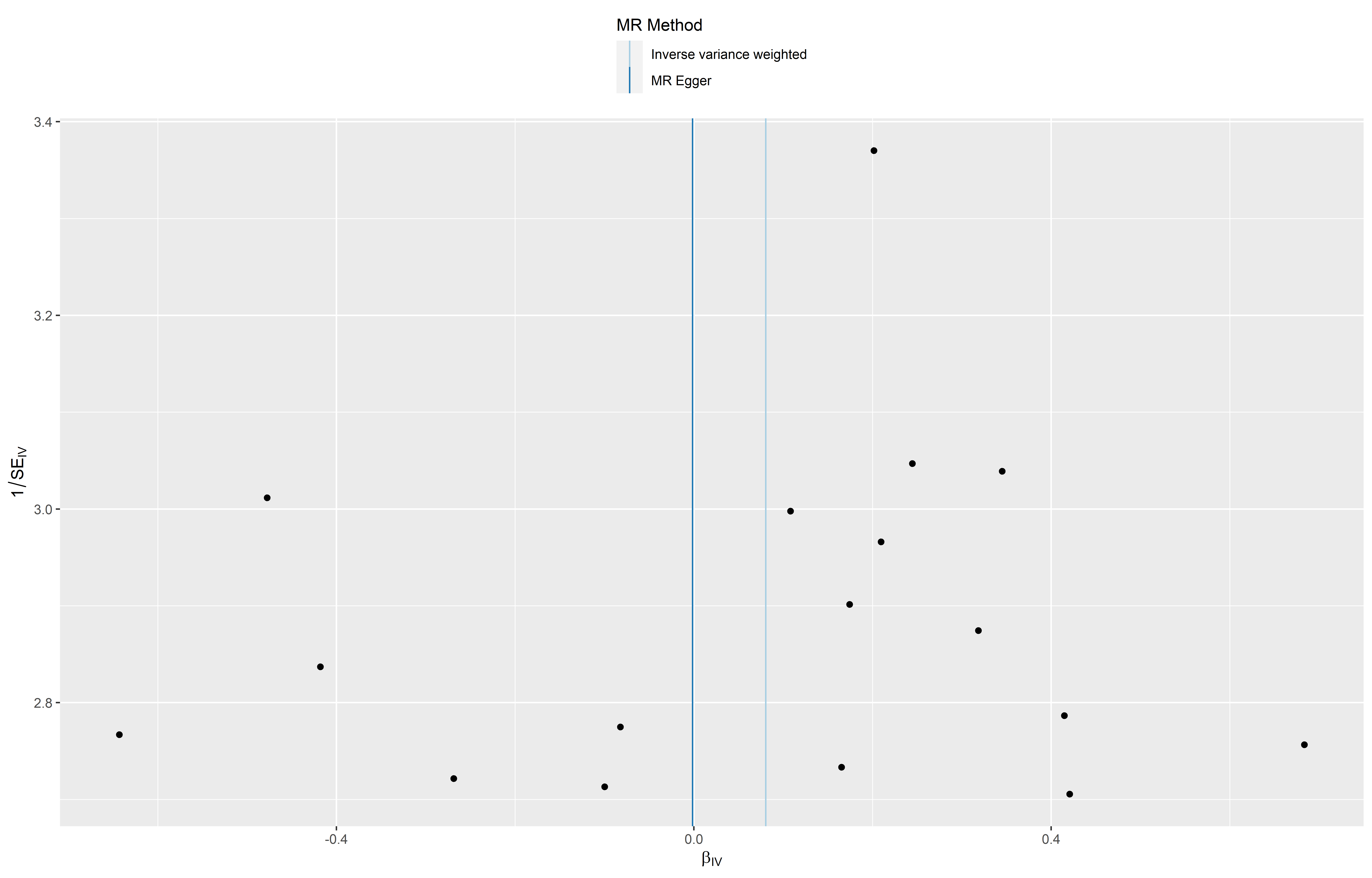

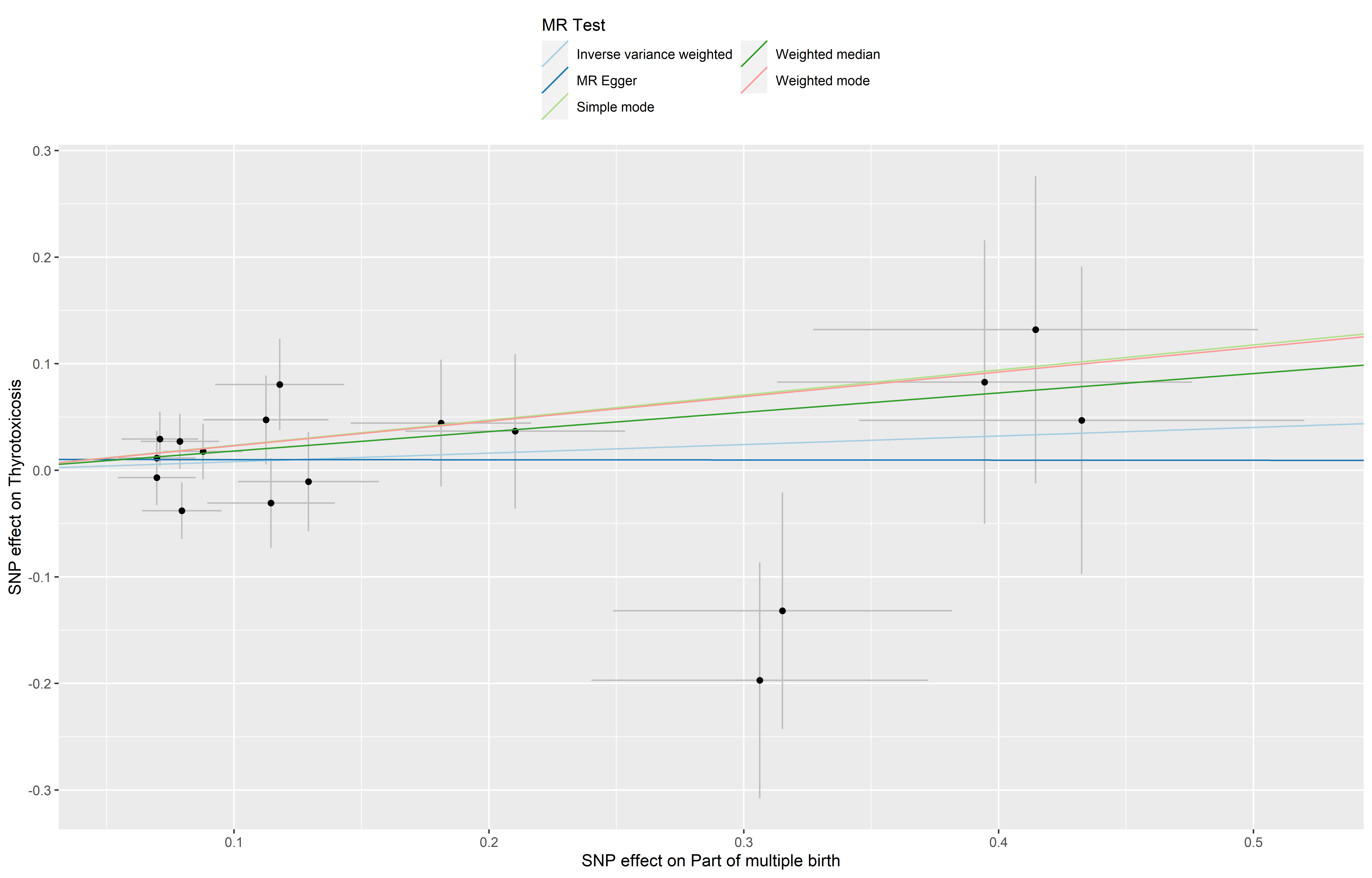


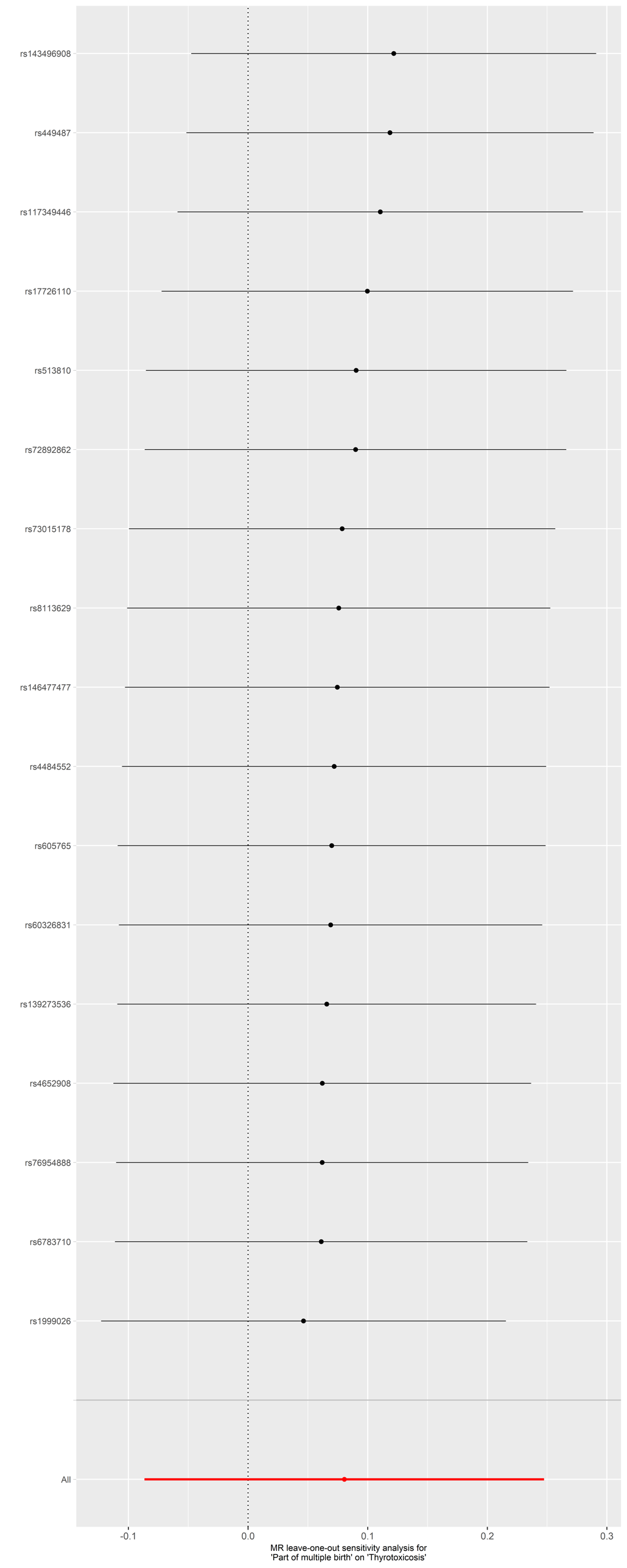


**Hypothyroidism – Finngen**


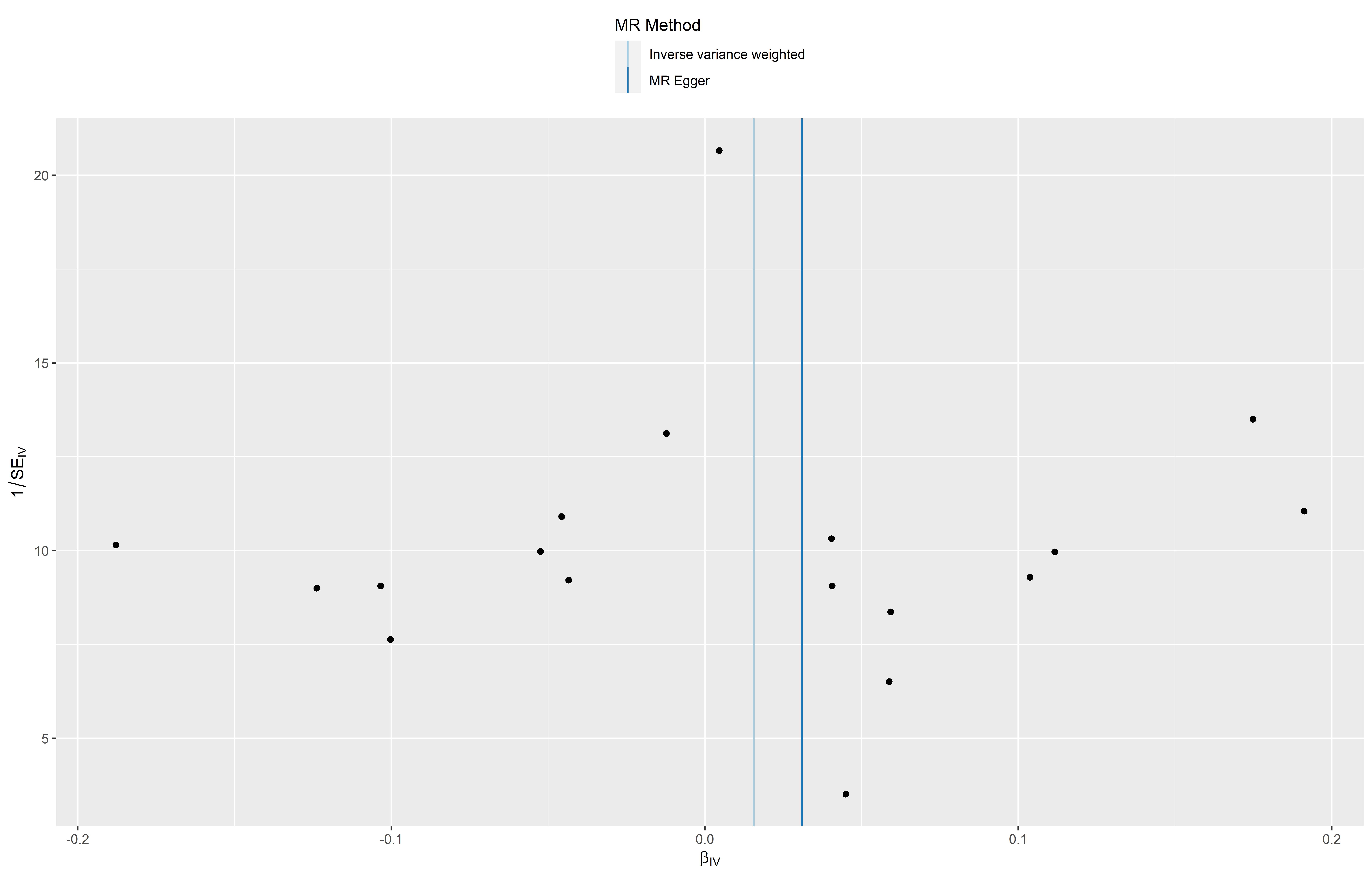

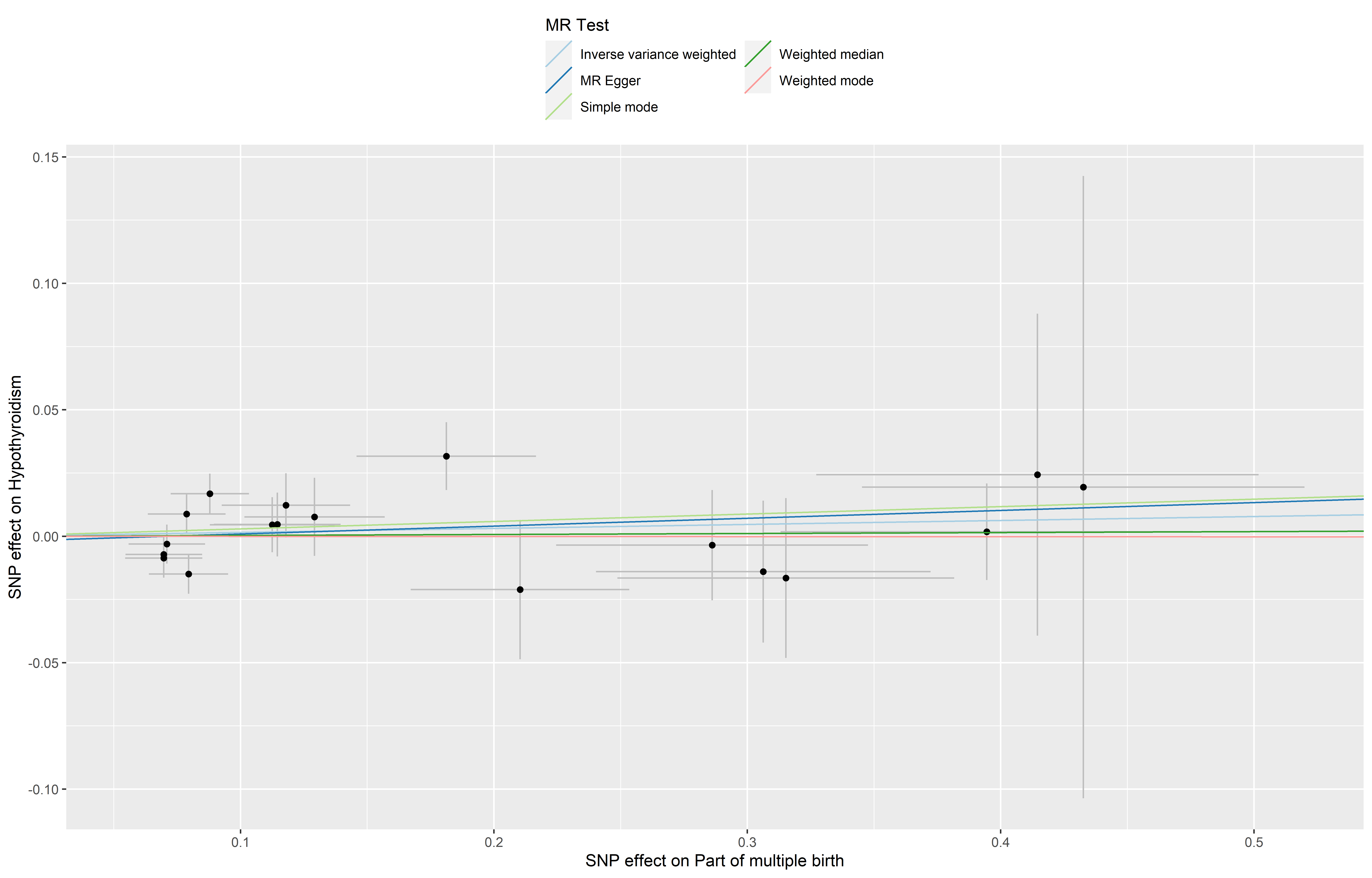


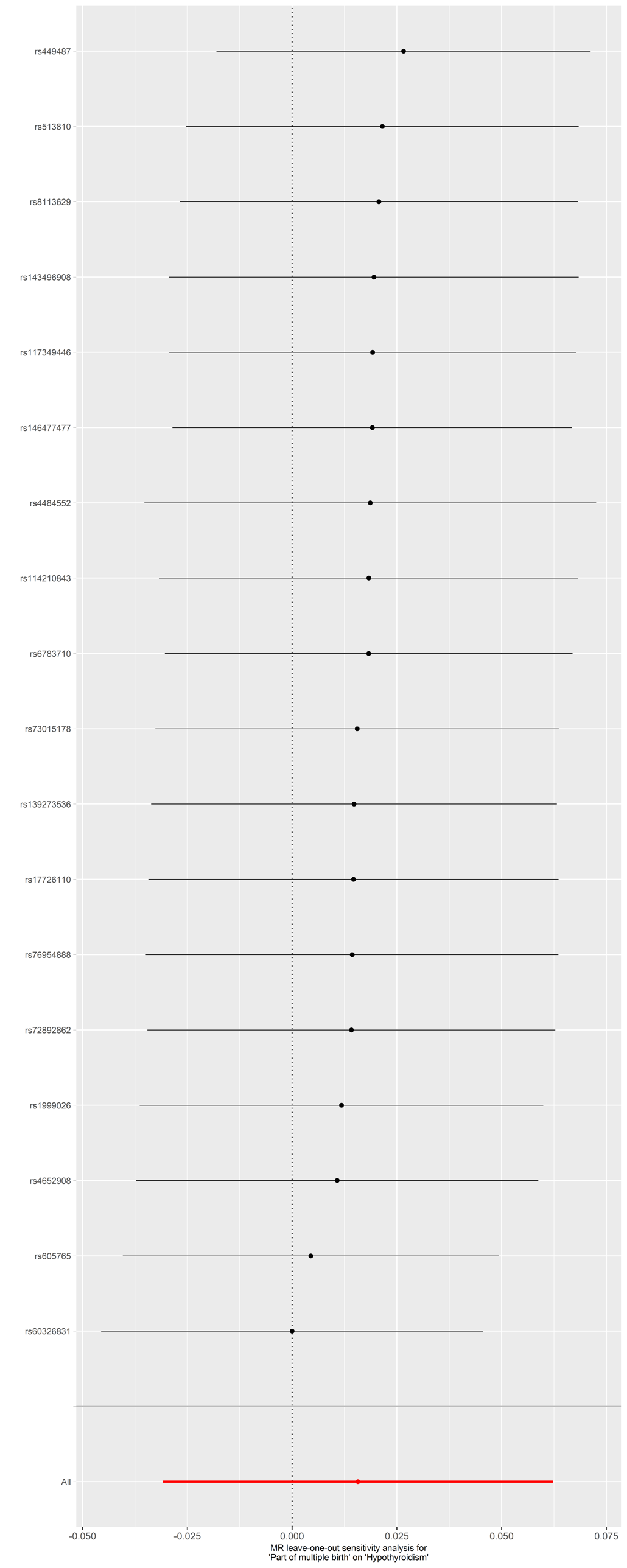


**Hypothyroidism – UK Biobank**


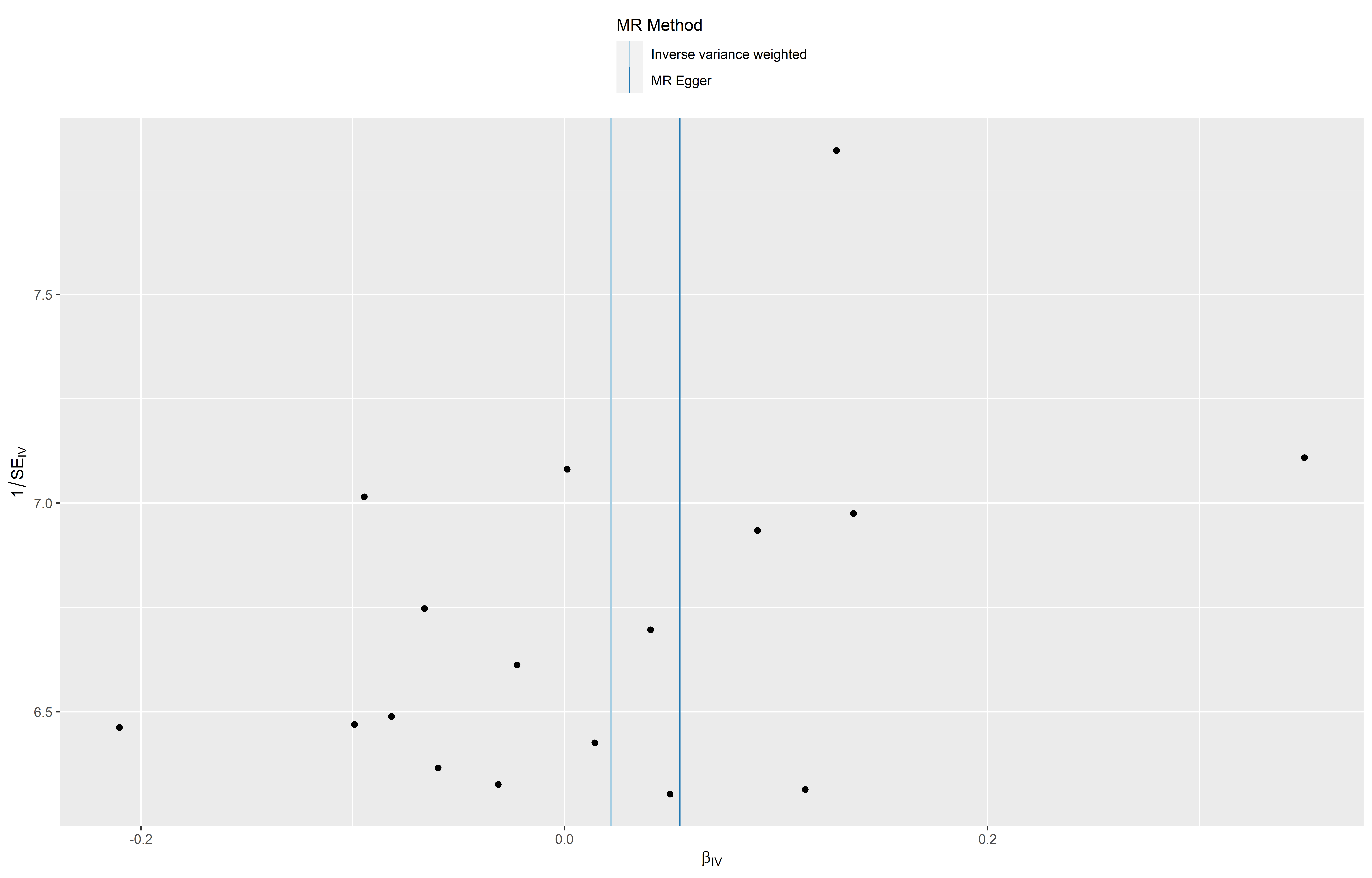

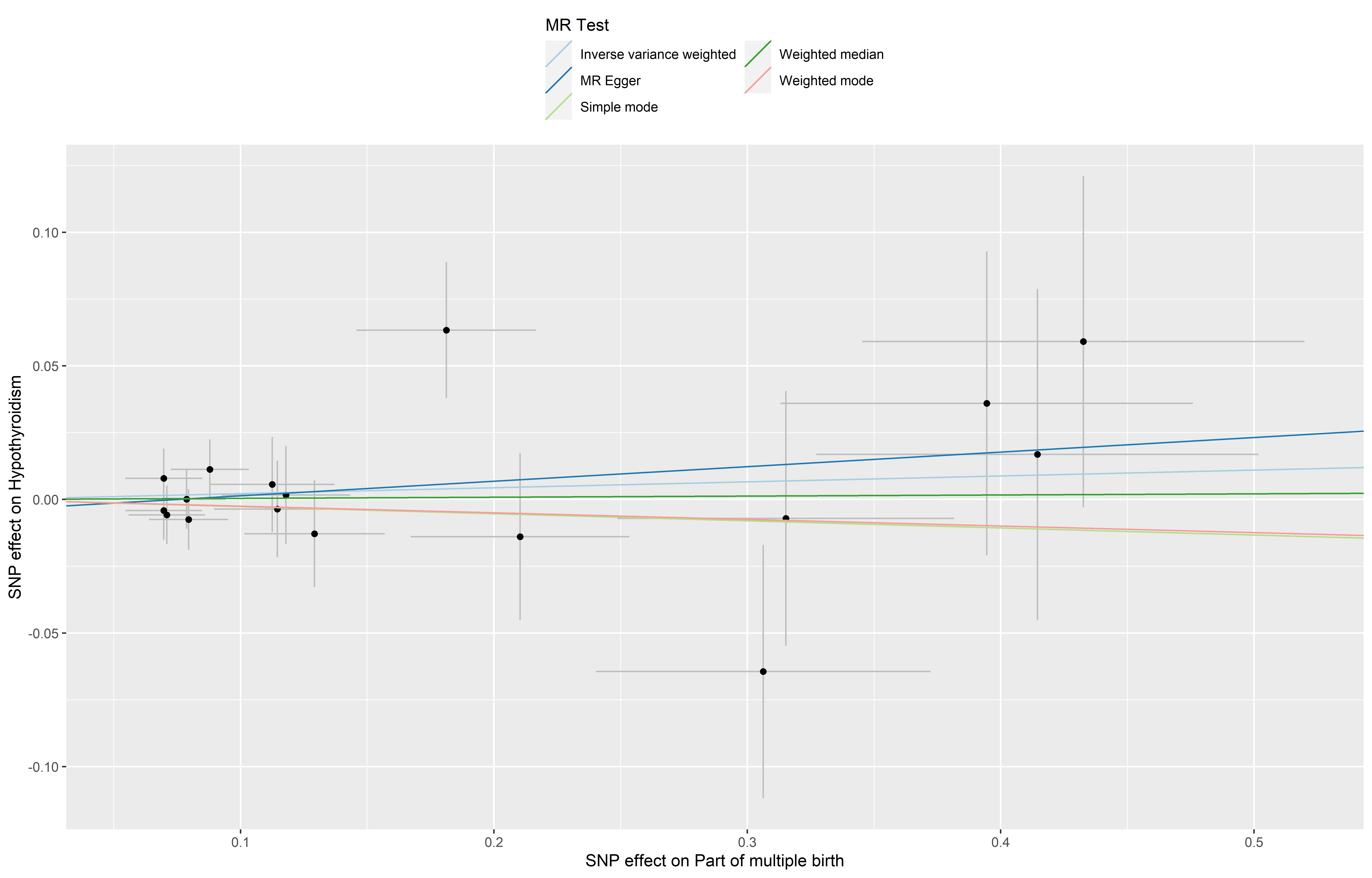


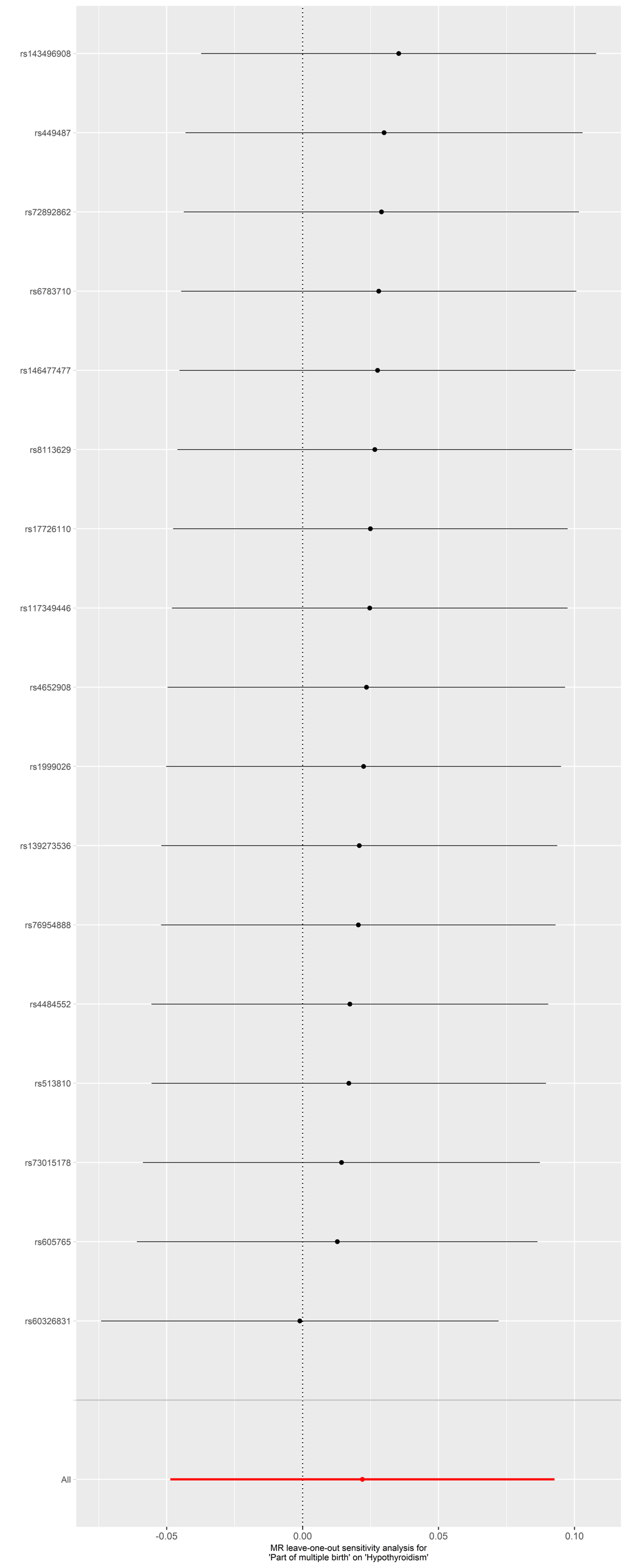


**Thyroiditis – Finngen**


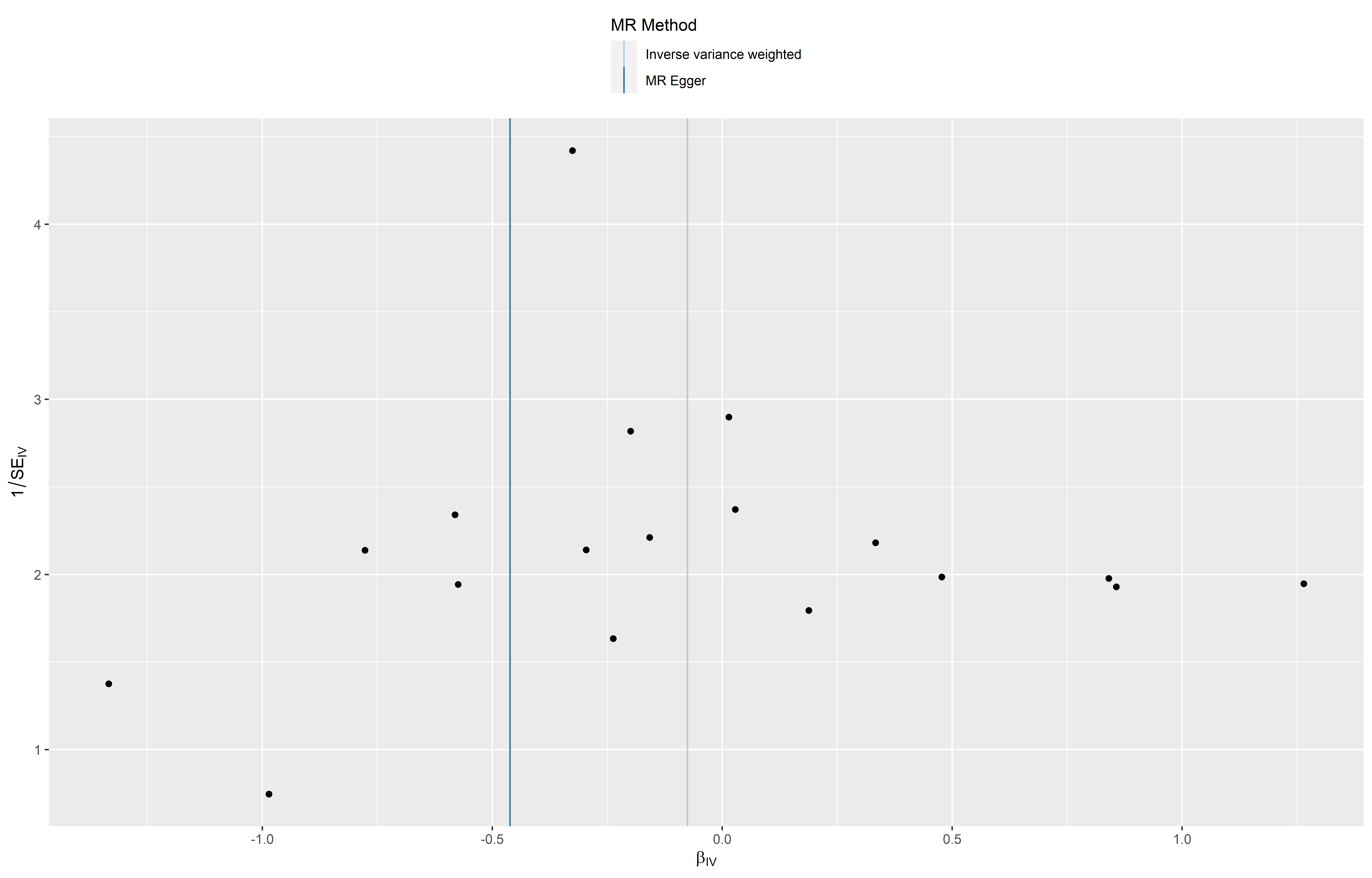

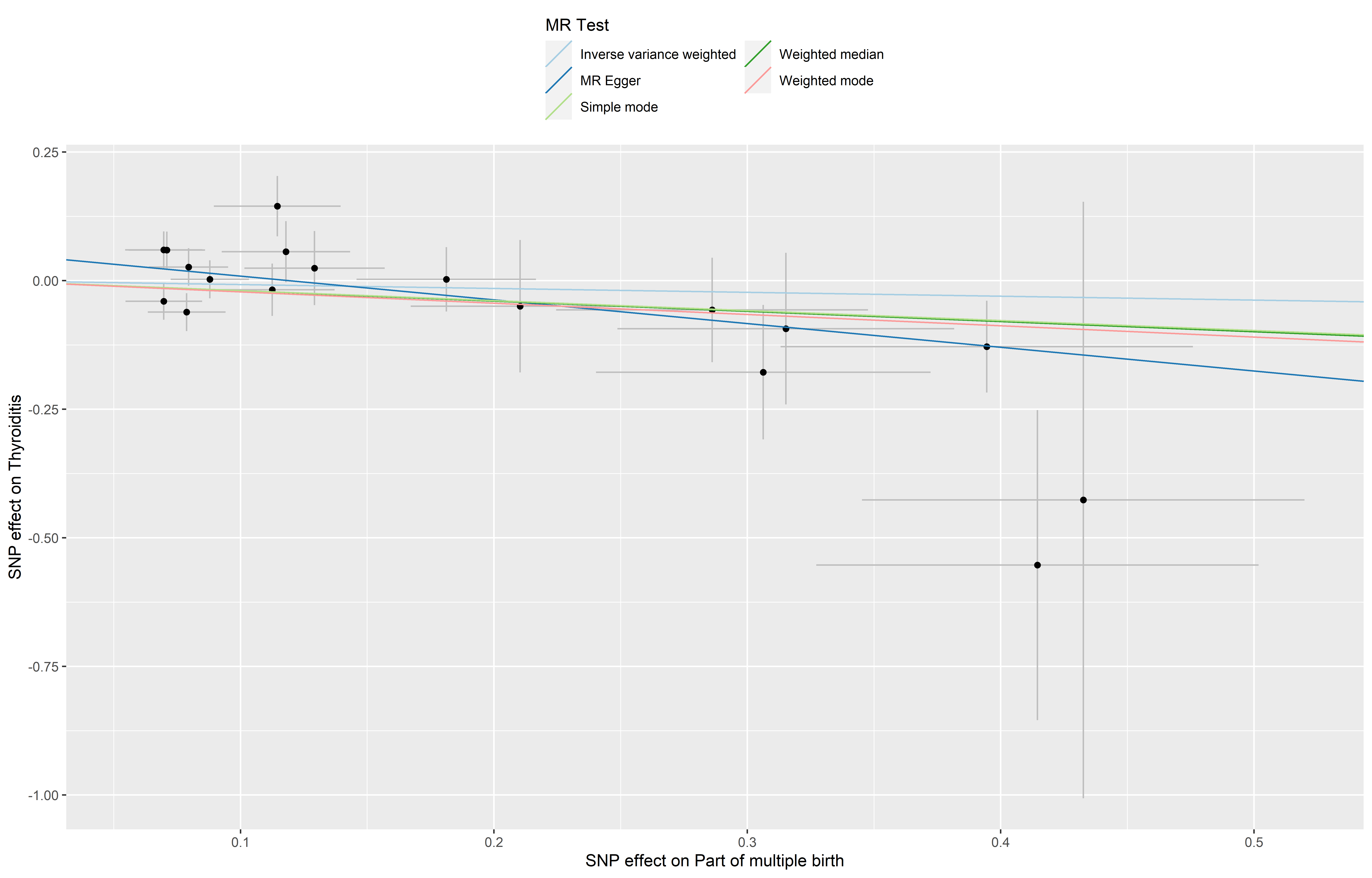


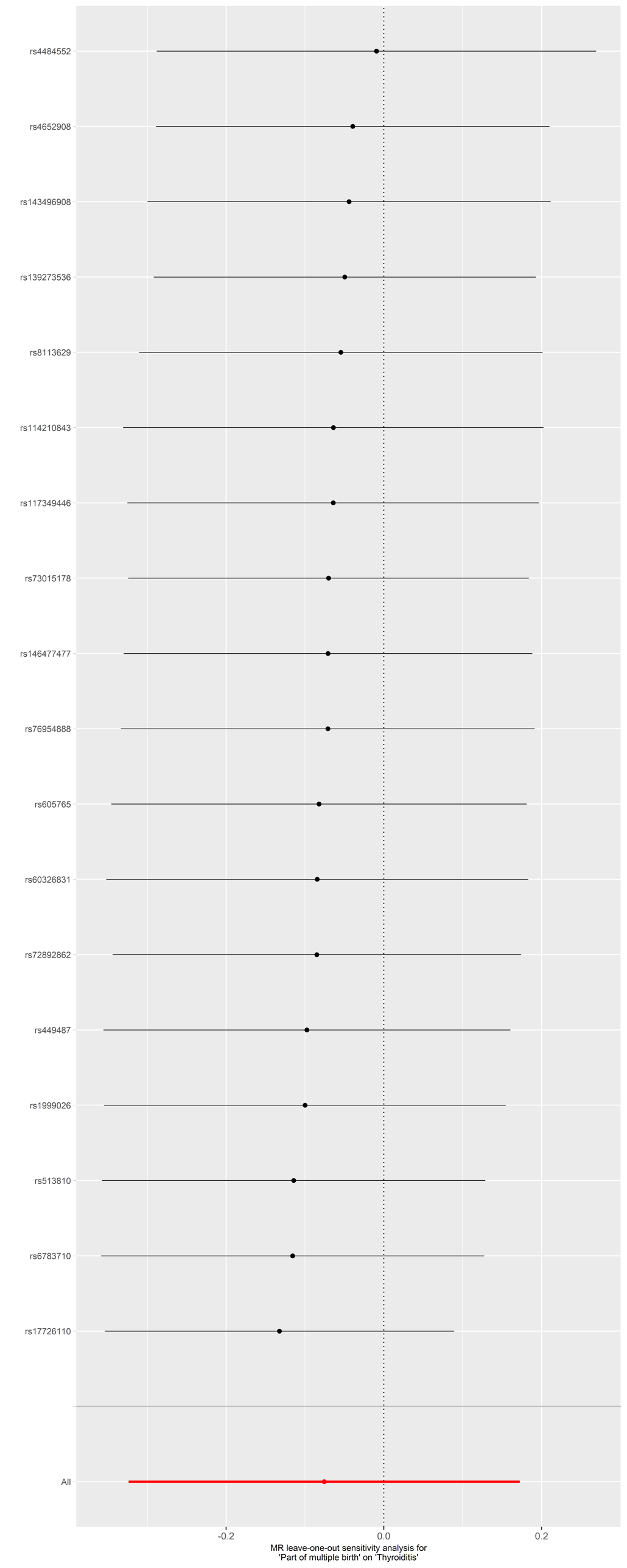


**Thyroiditis – UK Biobank**


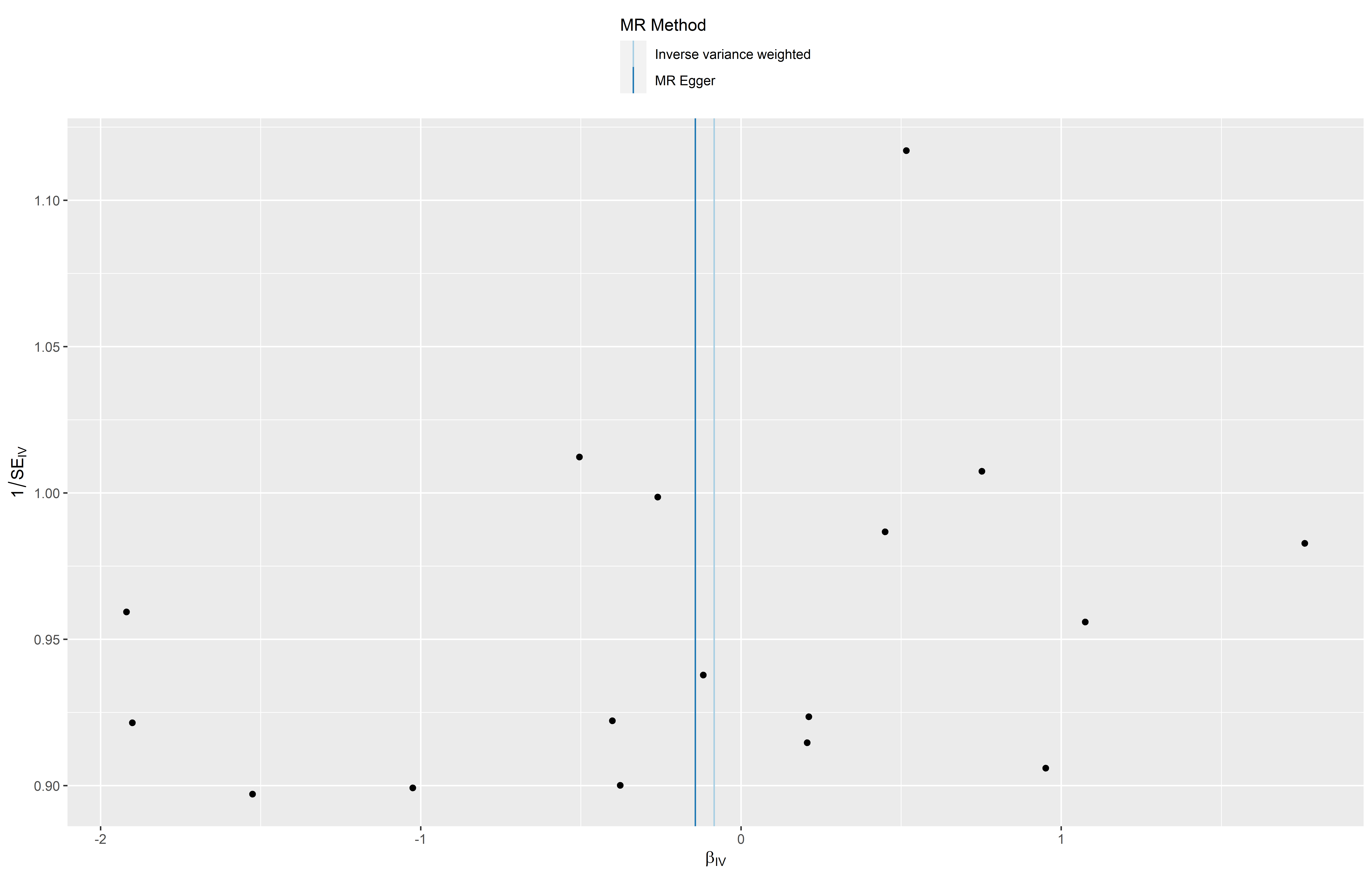

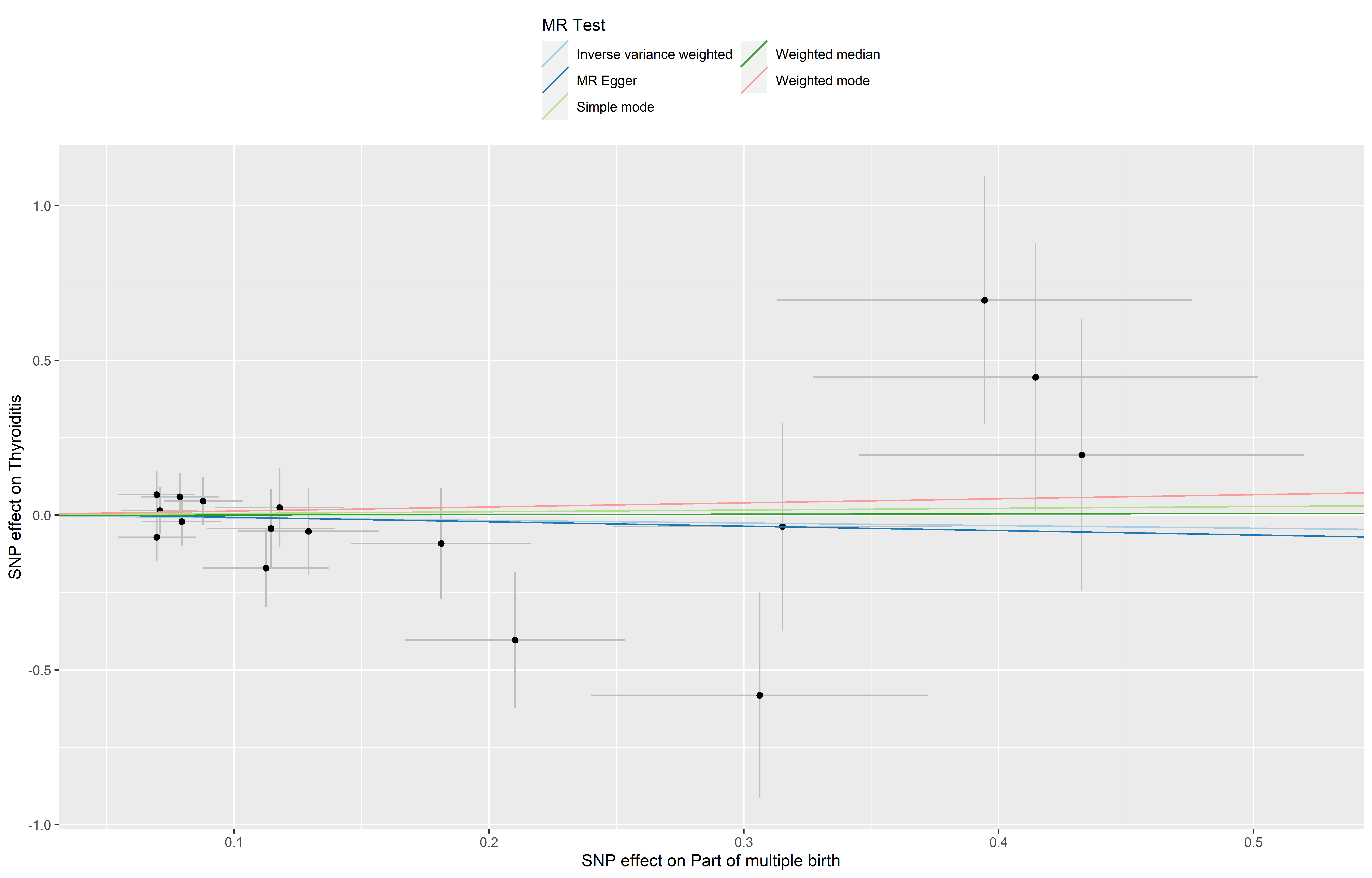


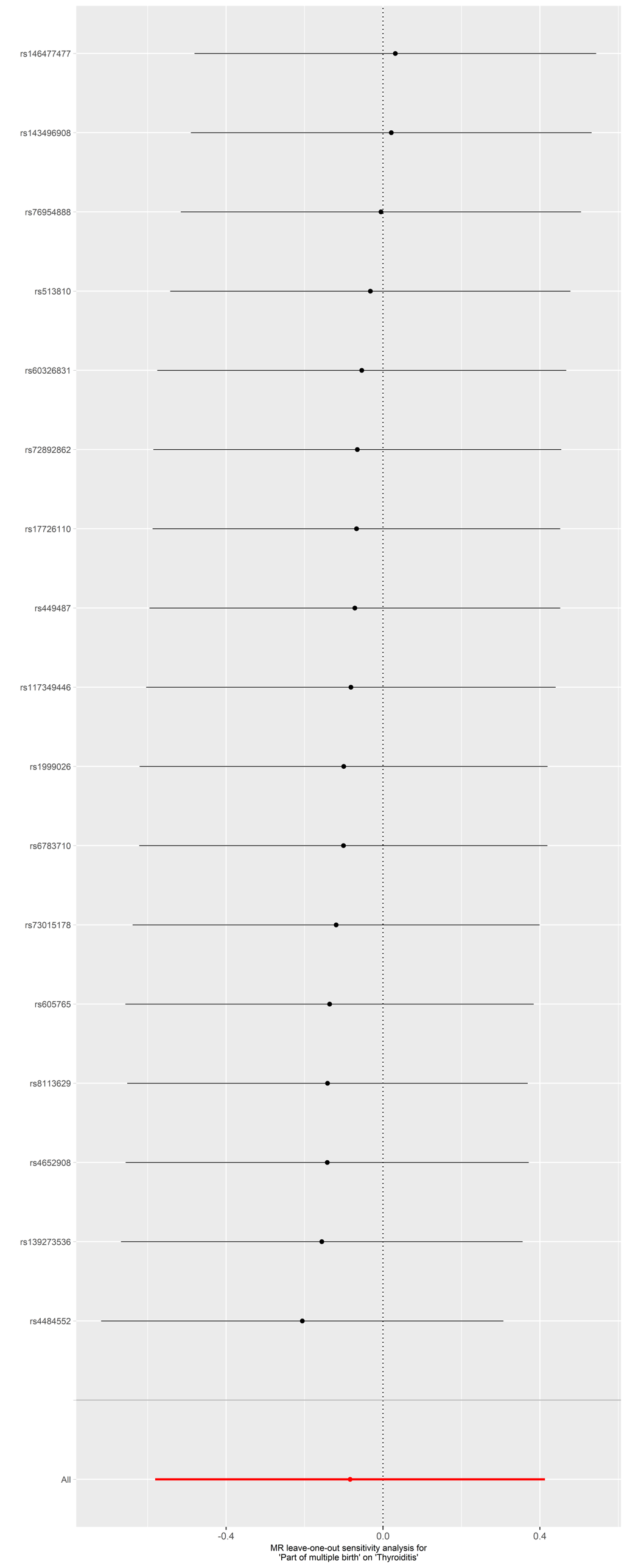


**Type 1 diabetes – Finngen**


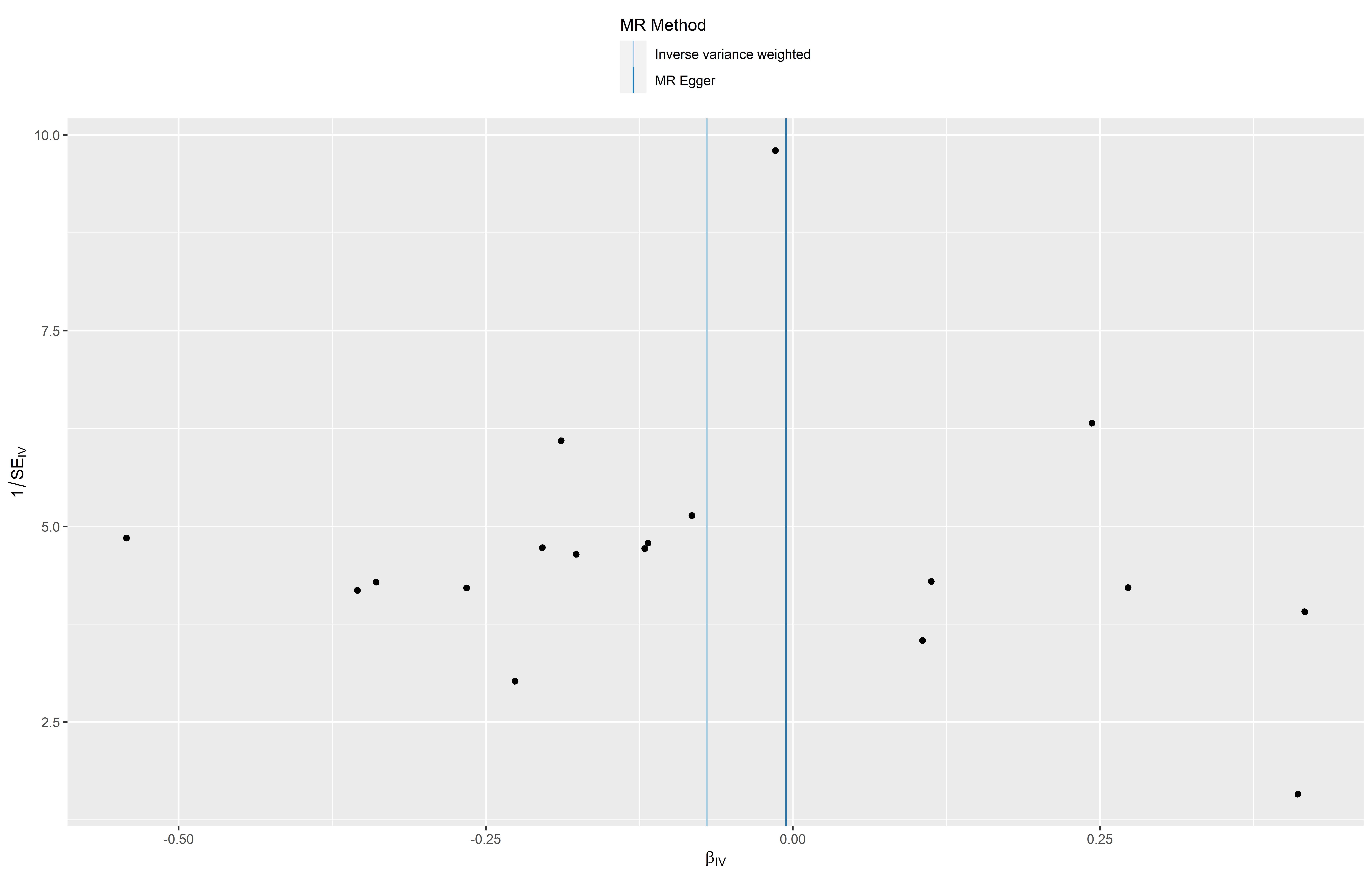

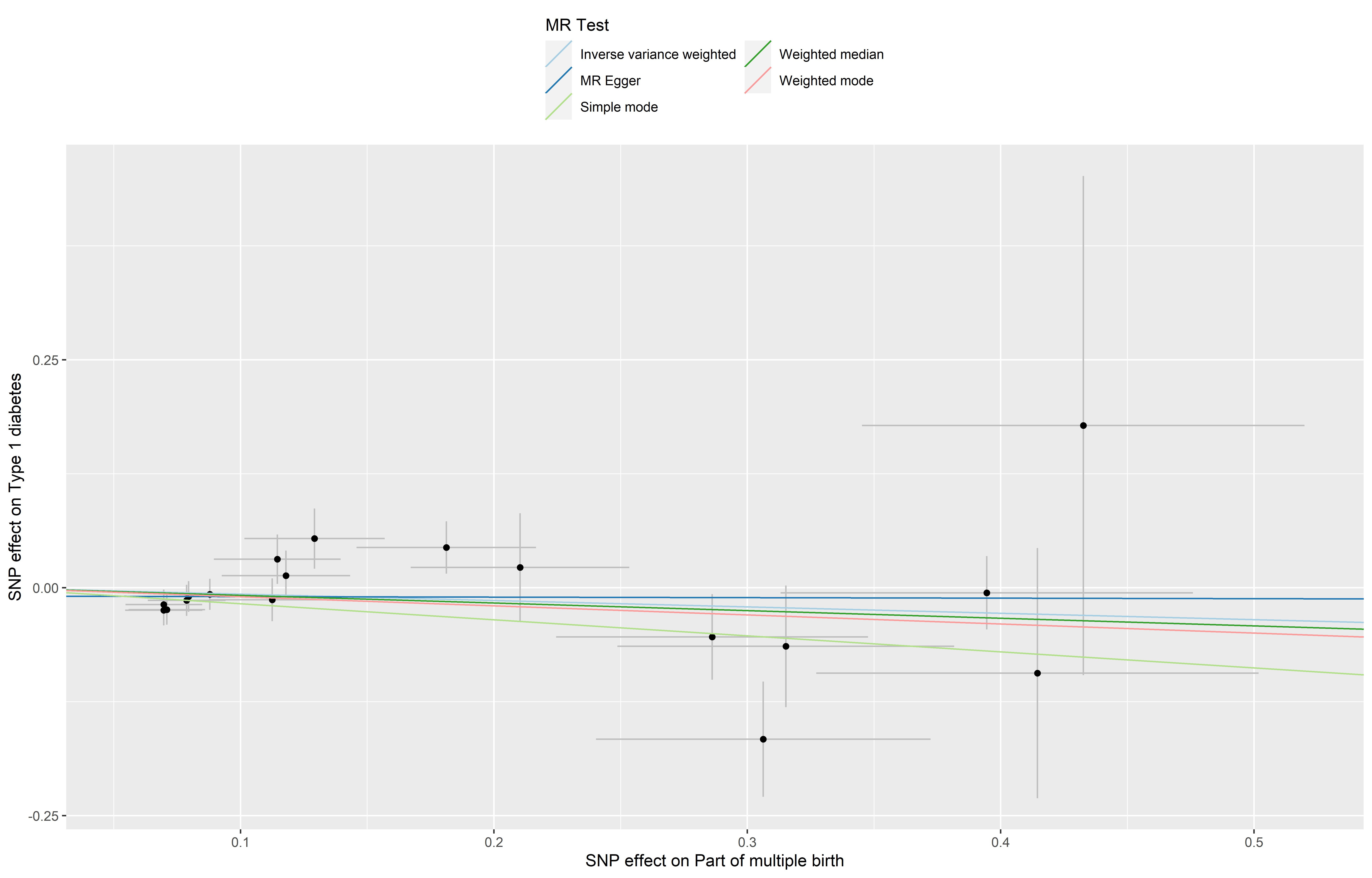


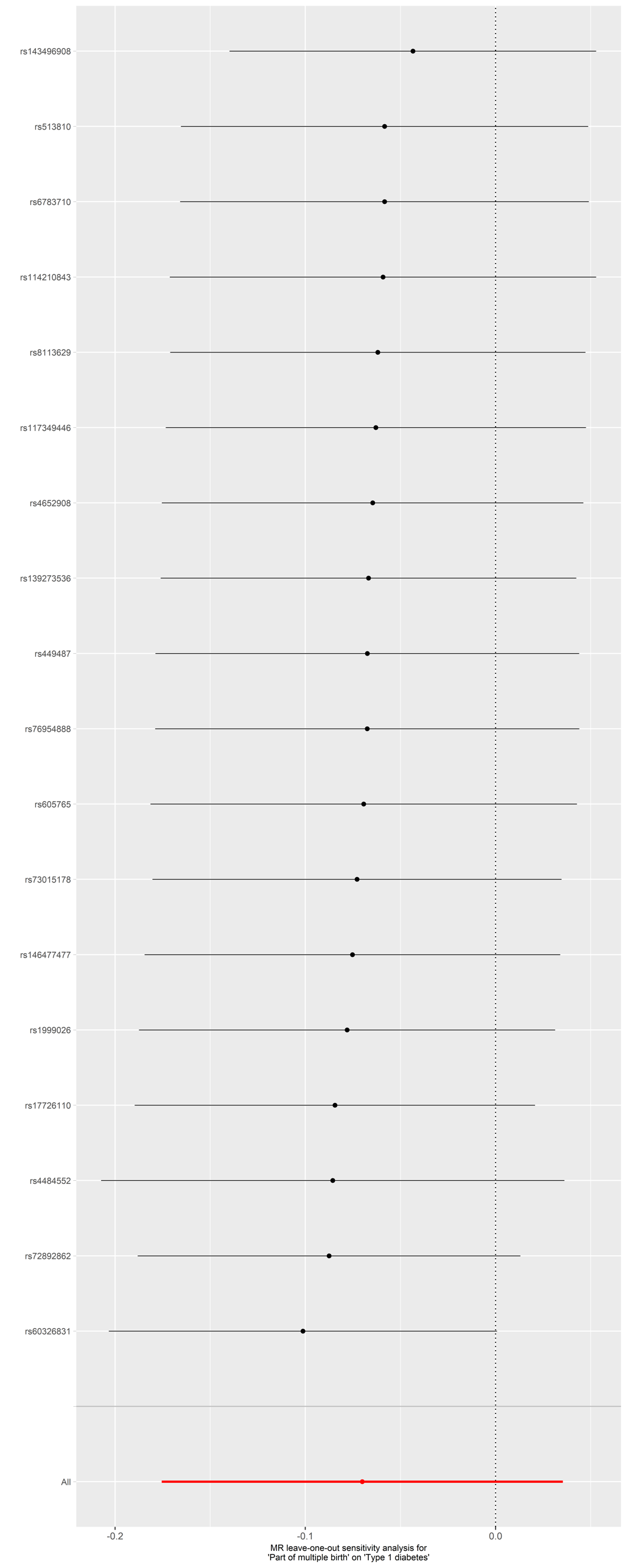


**Type 1 diabetes – UK Biobank**


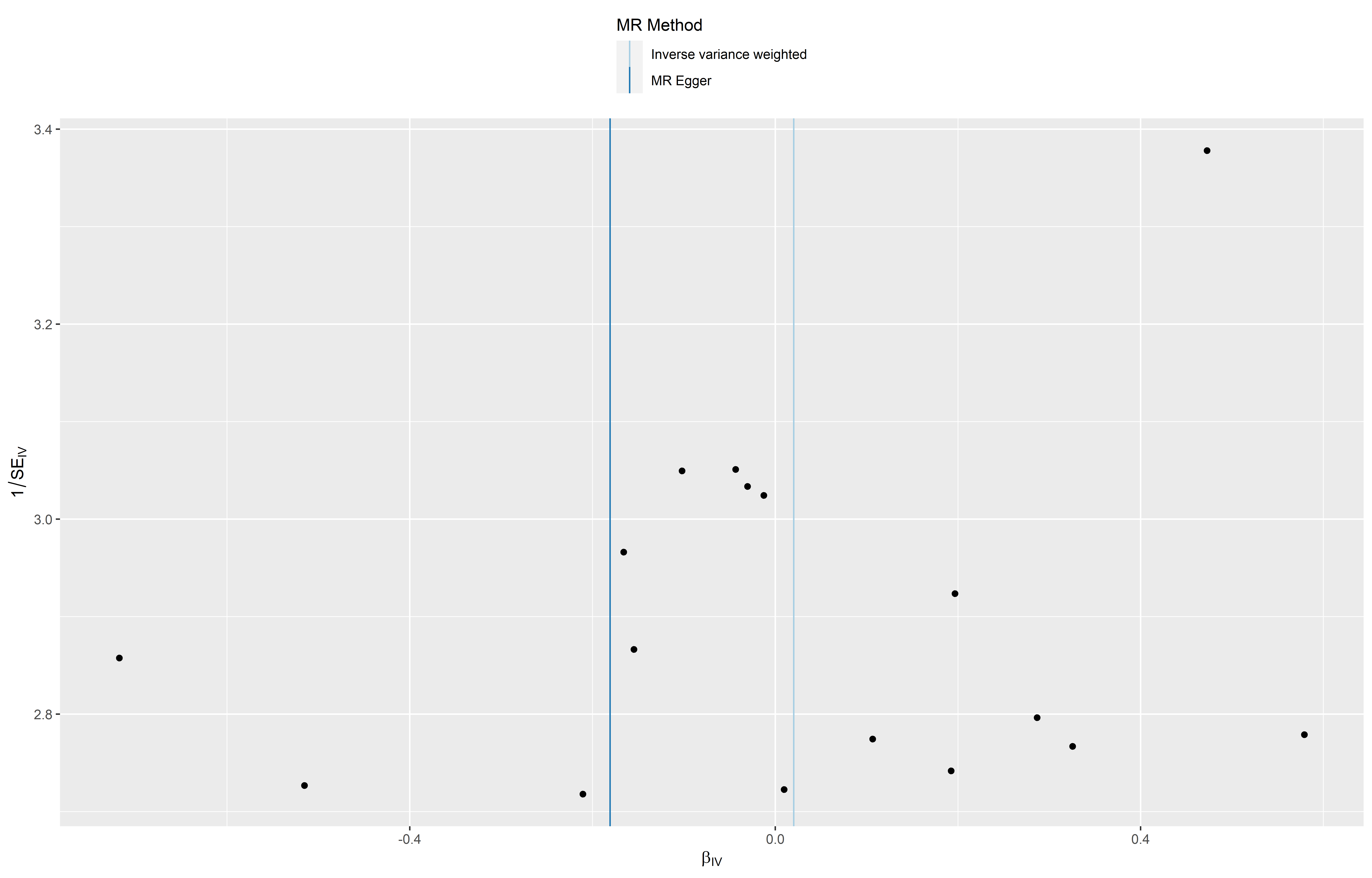

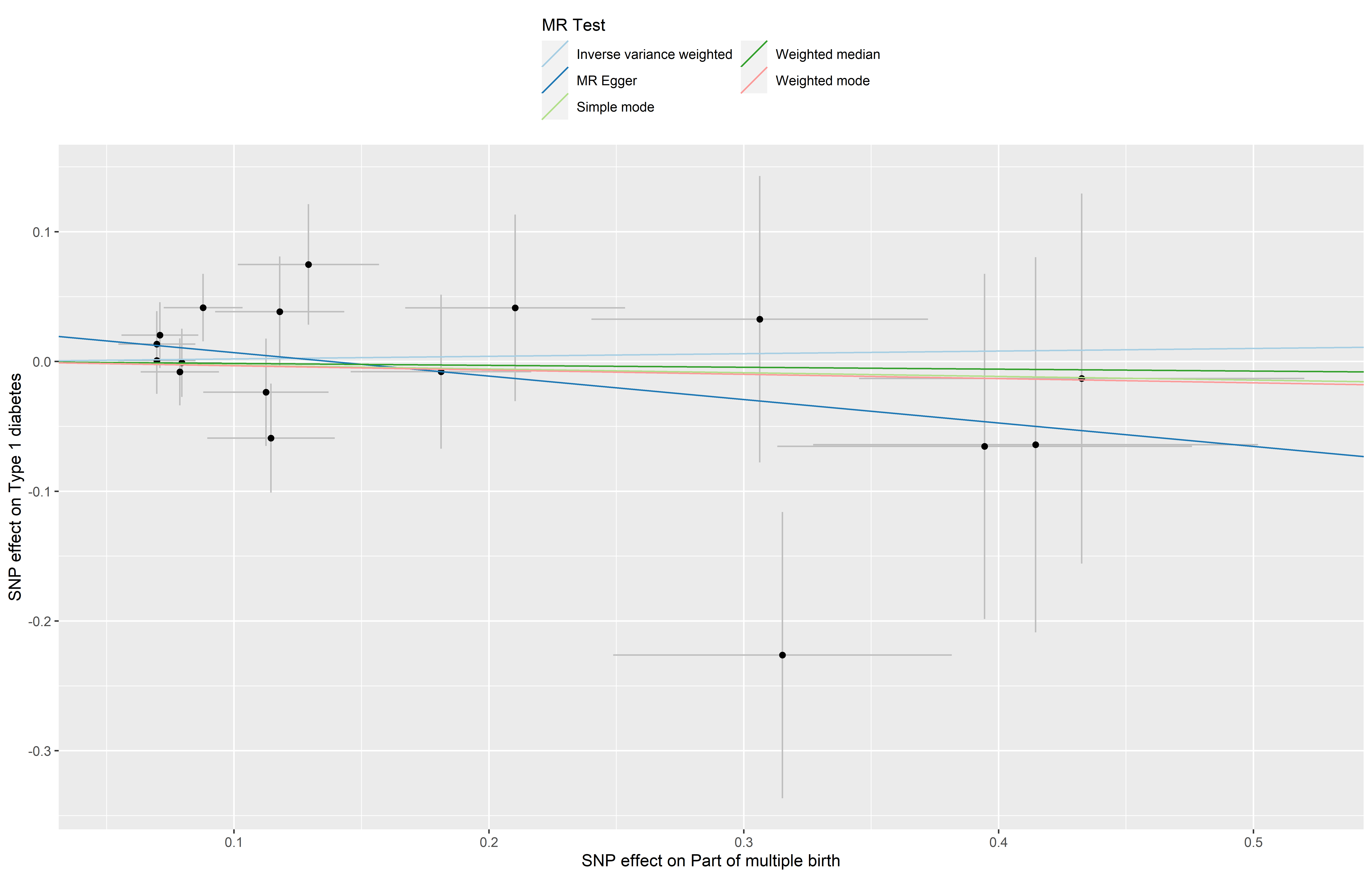


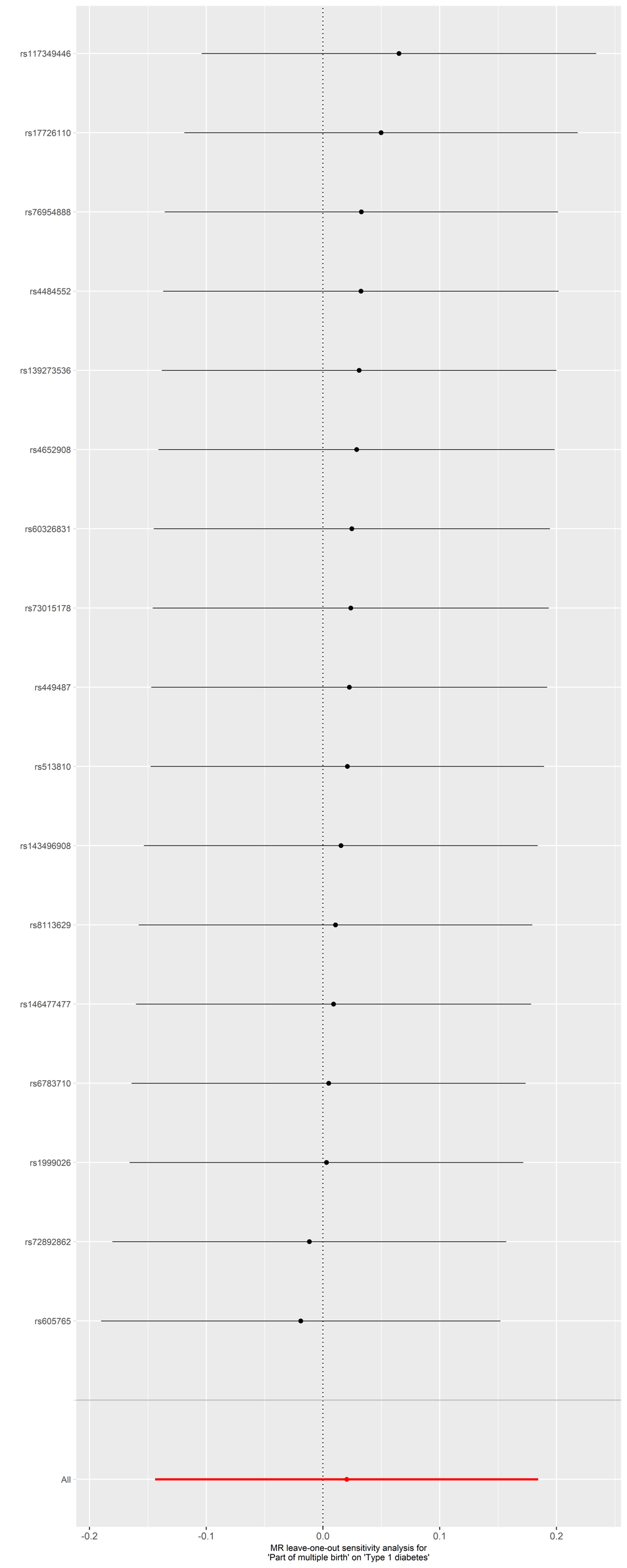


**Type 2 diabetes – Finngen**


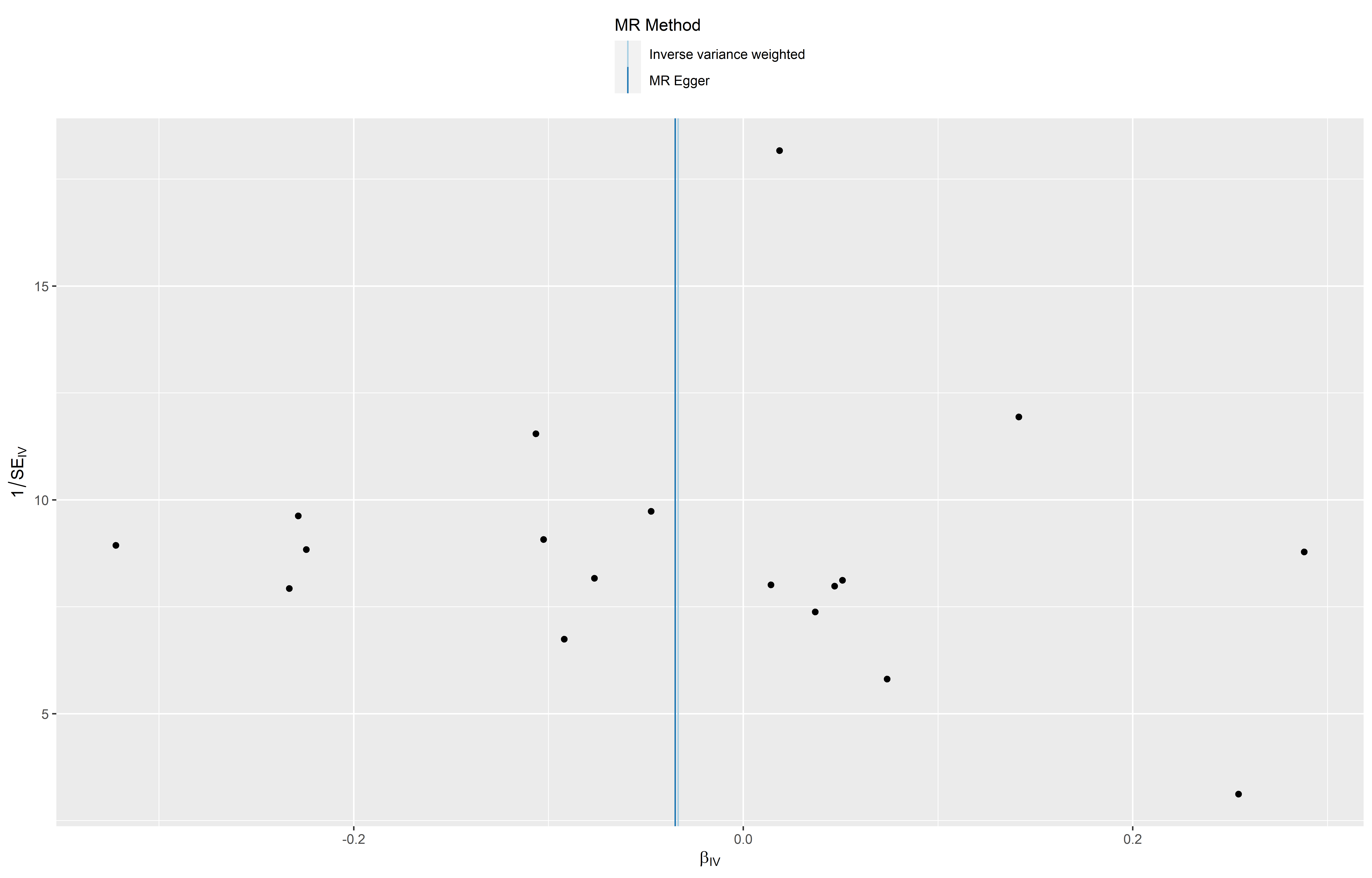

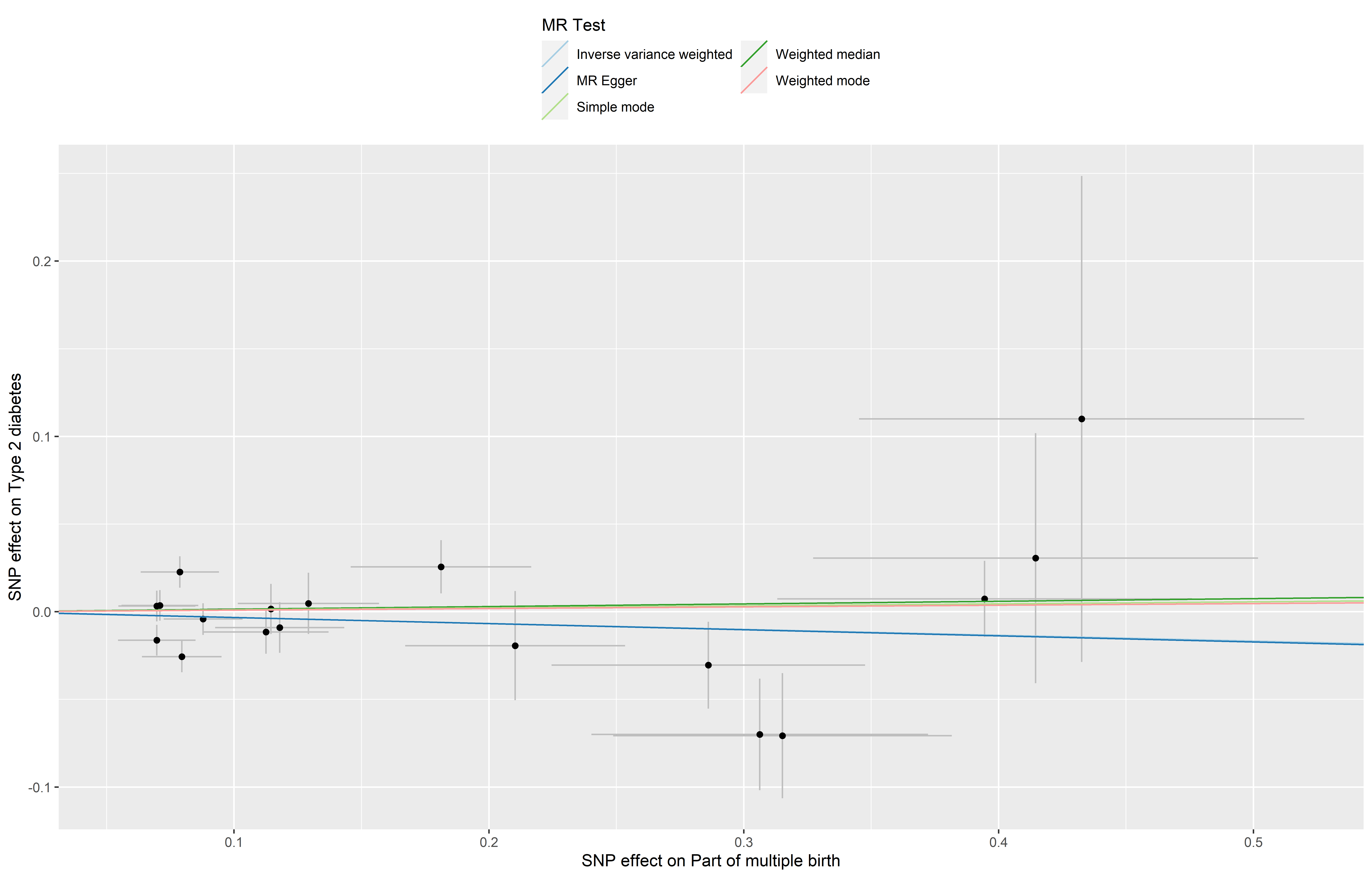


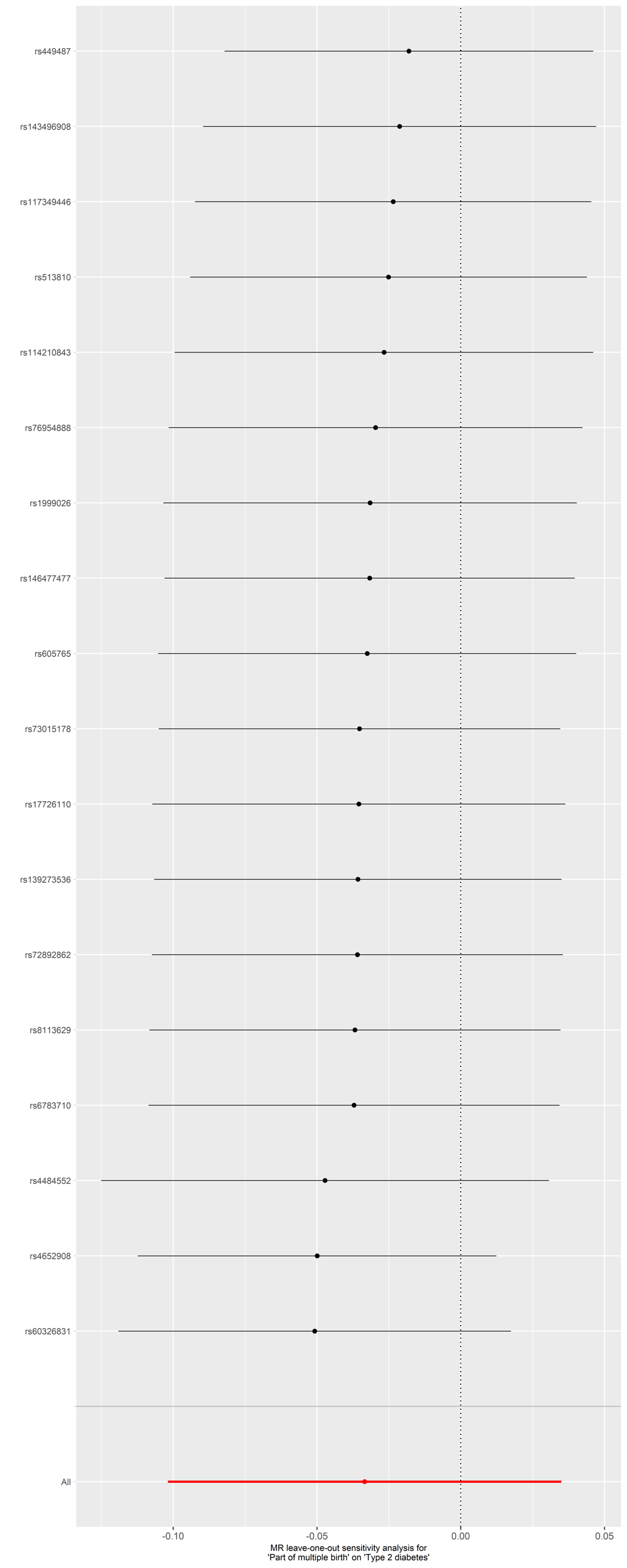


**Type 2 diabetes – UK Biobank**


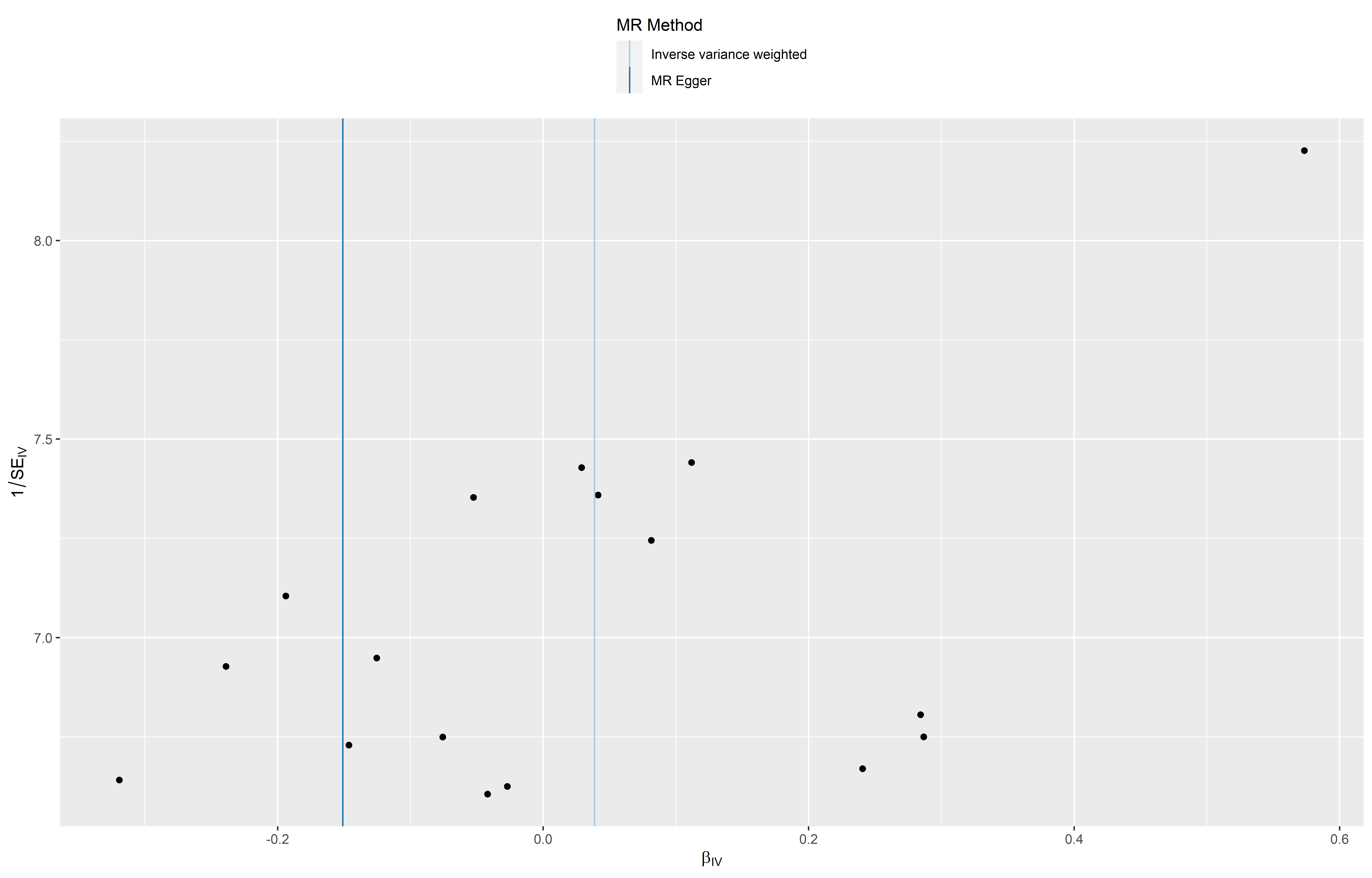

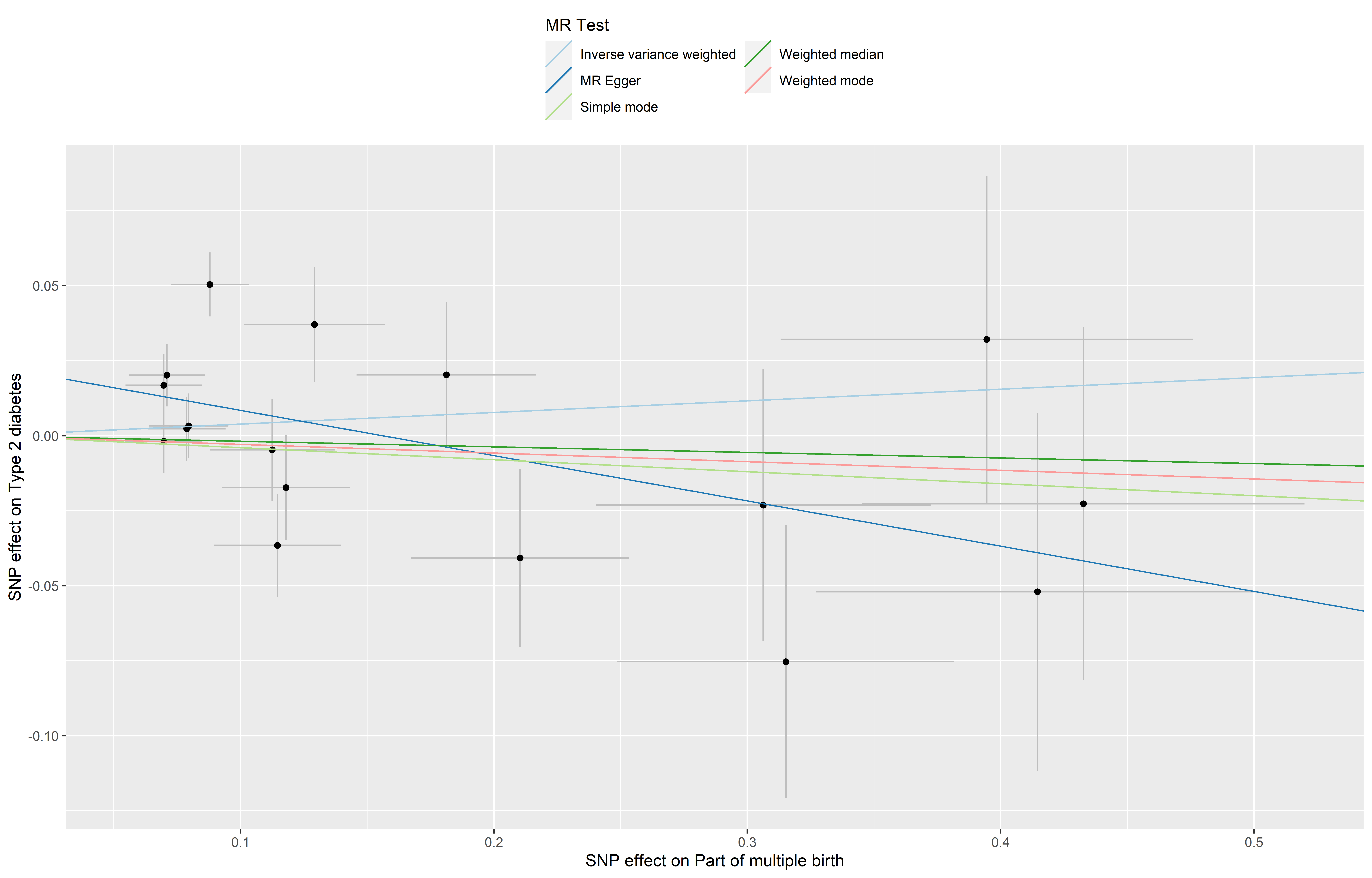


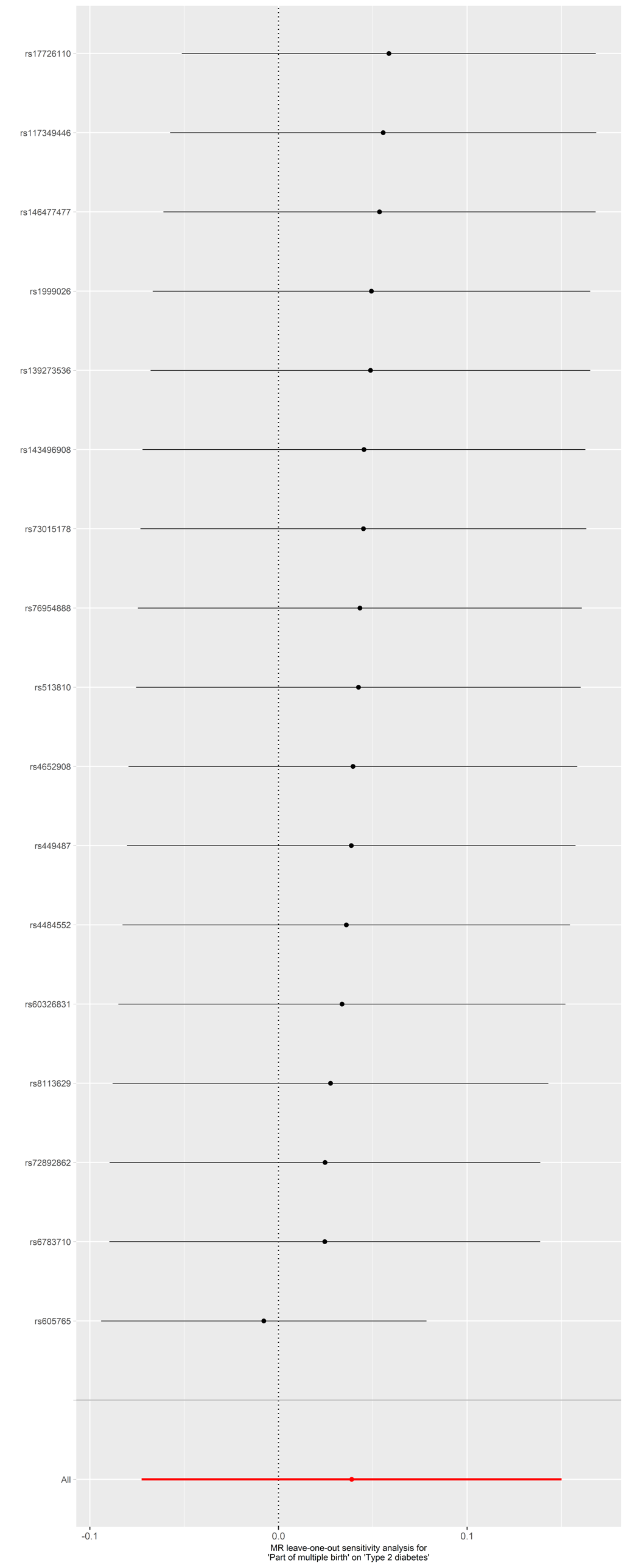


**Obesity – Finngen**


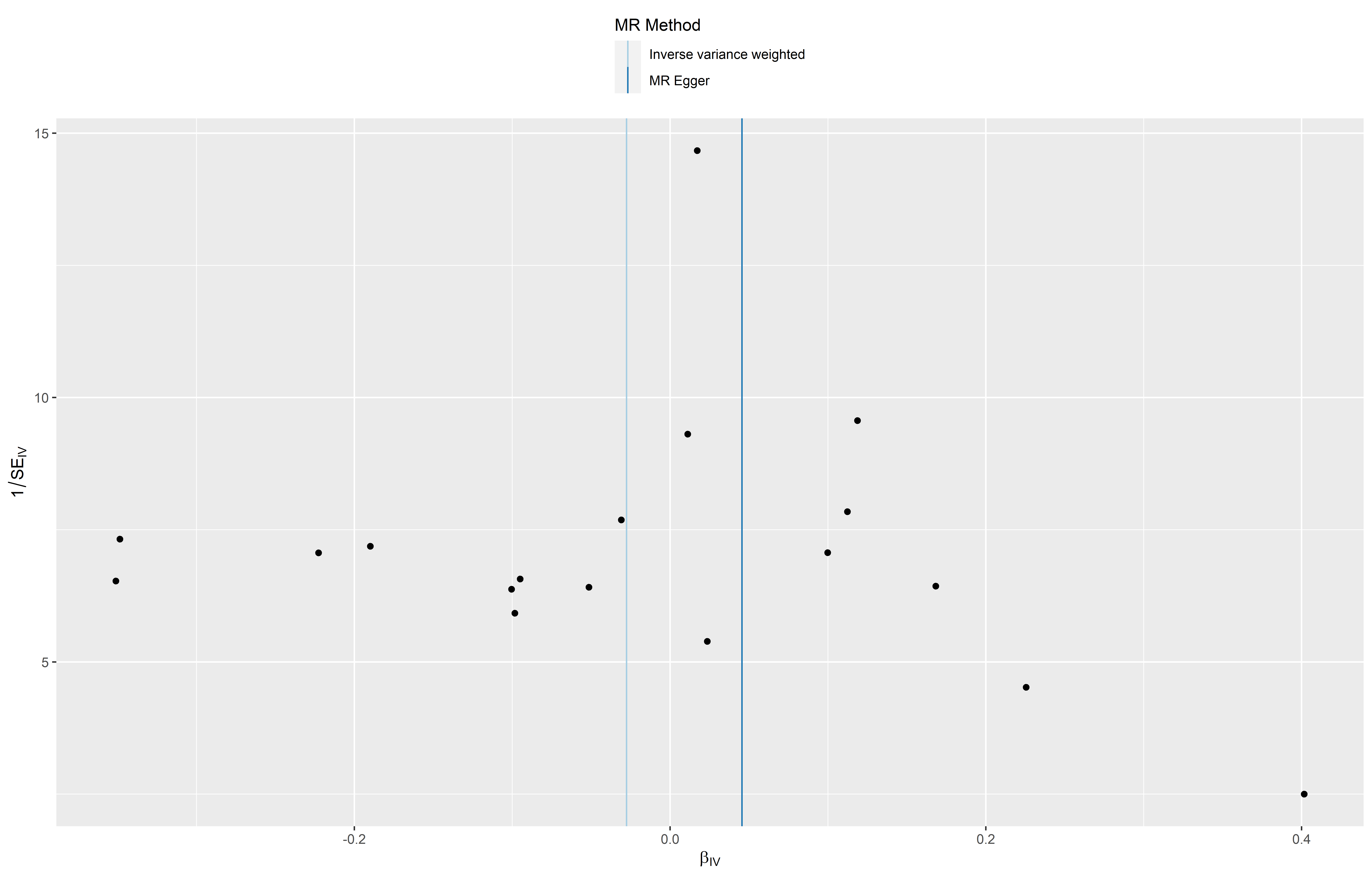

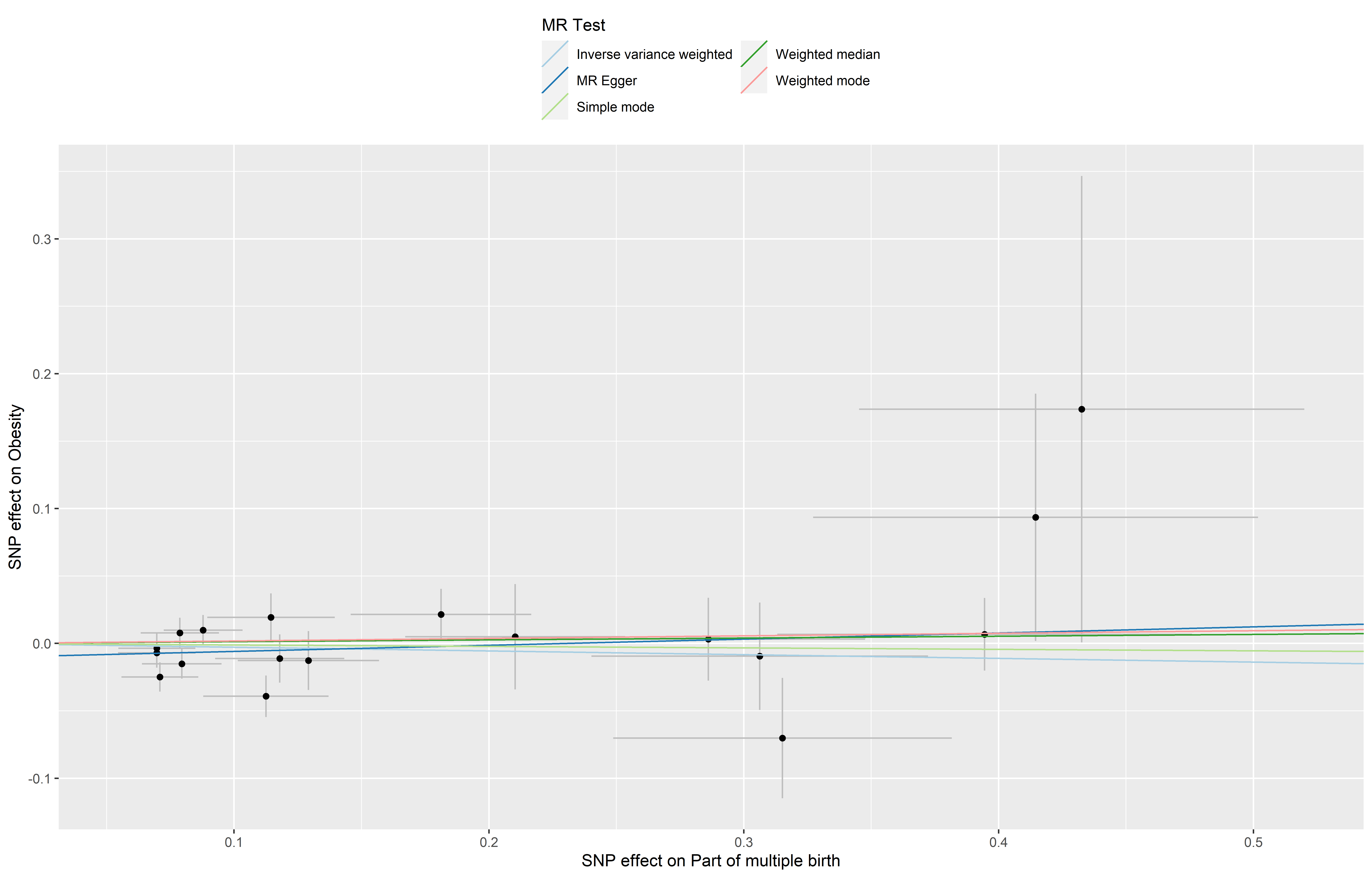


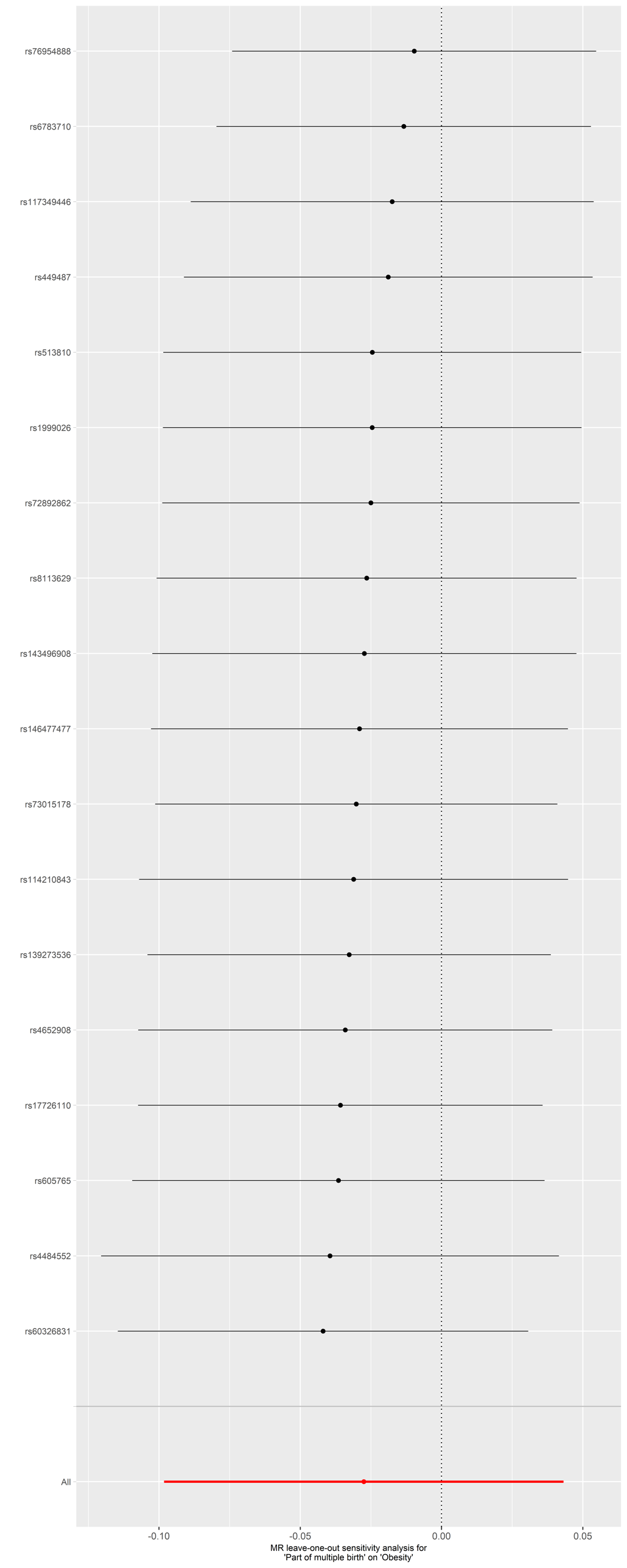


**Obesity – UK Biobank**


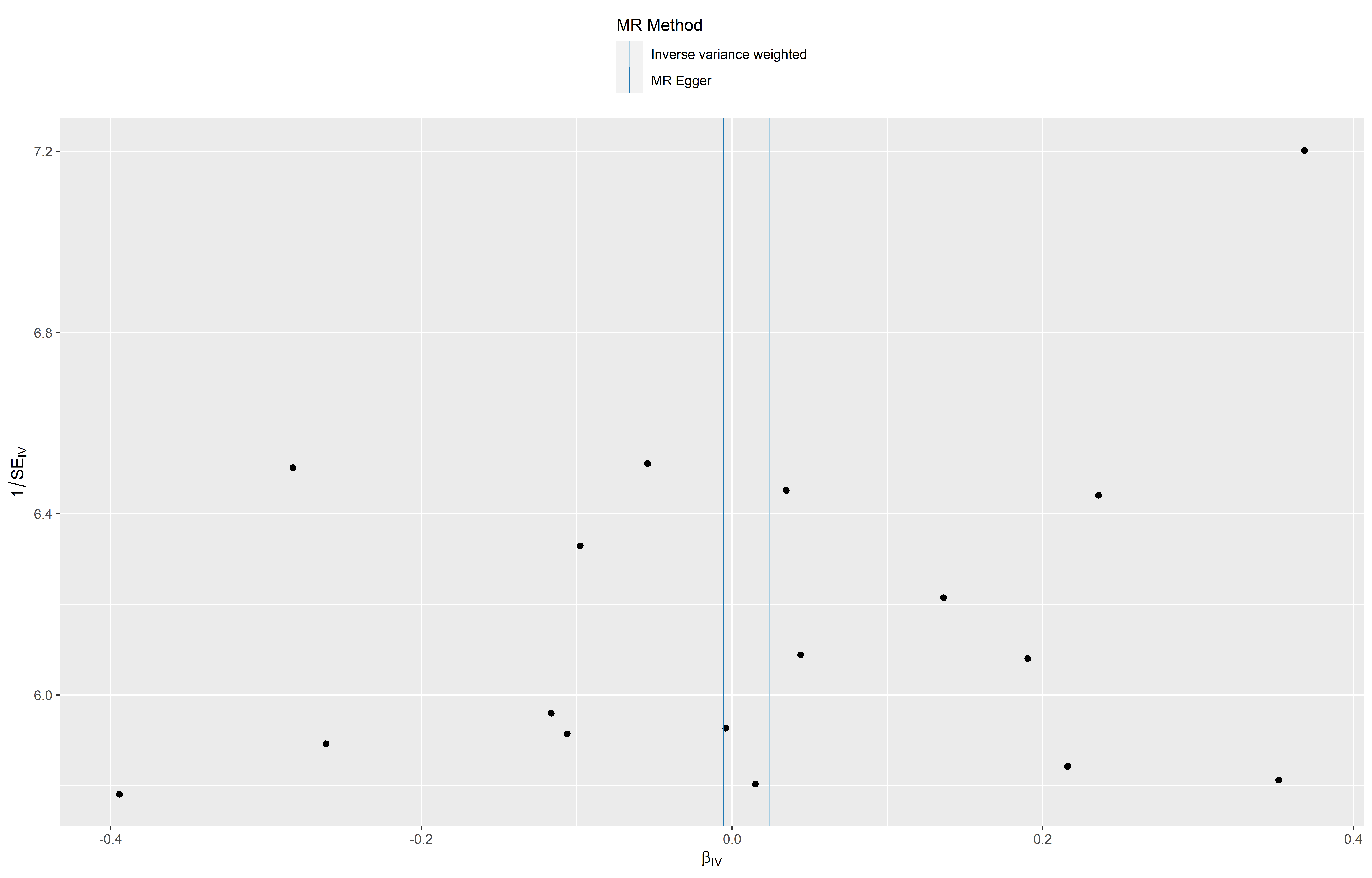

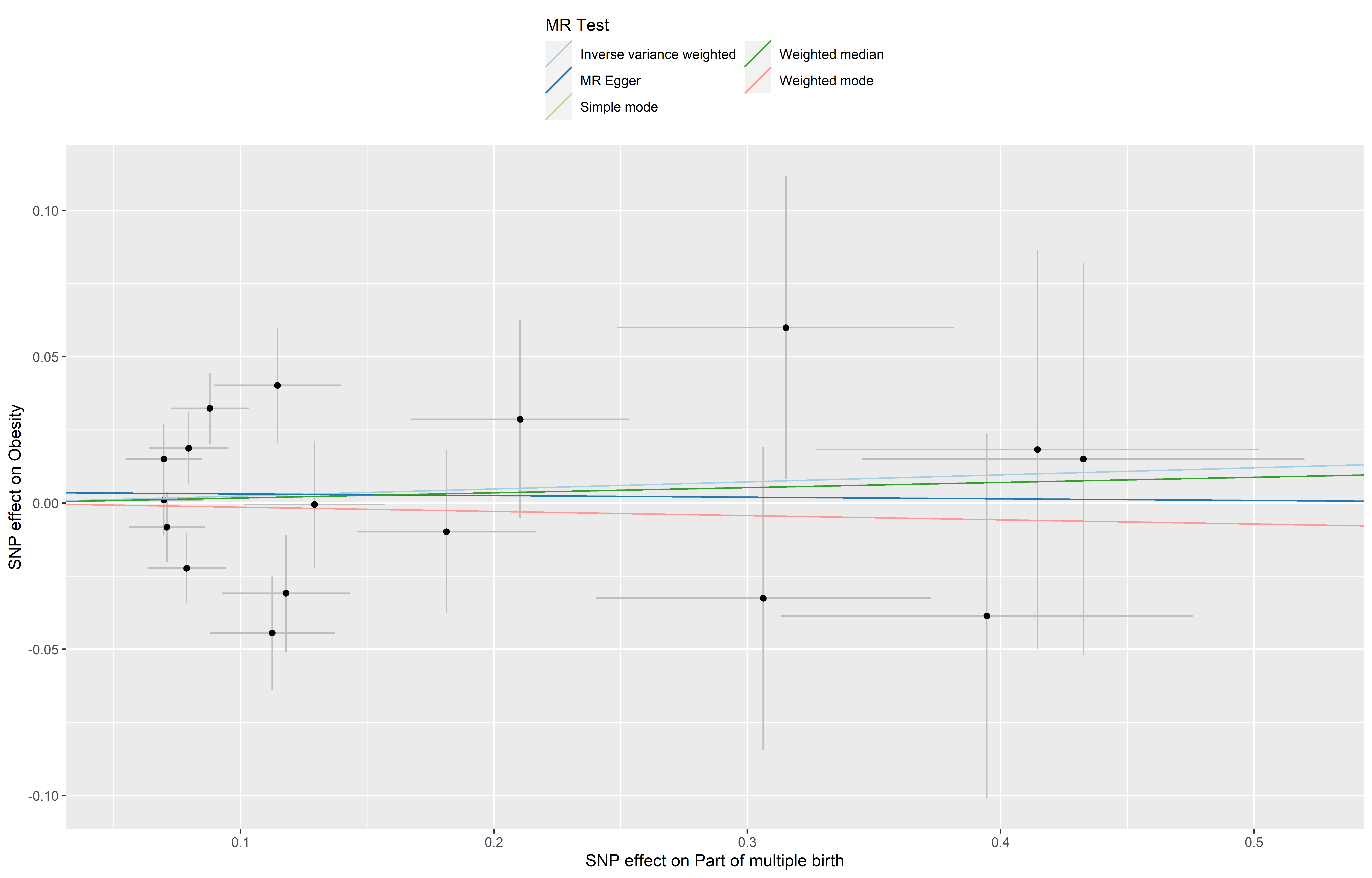


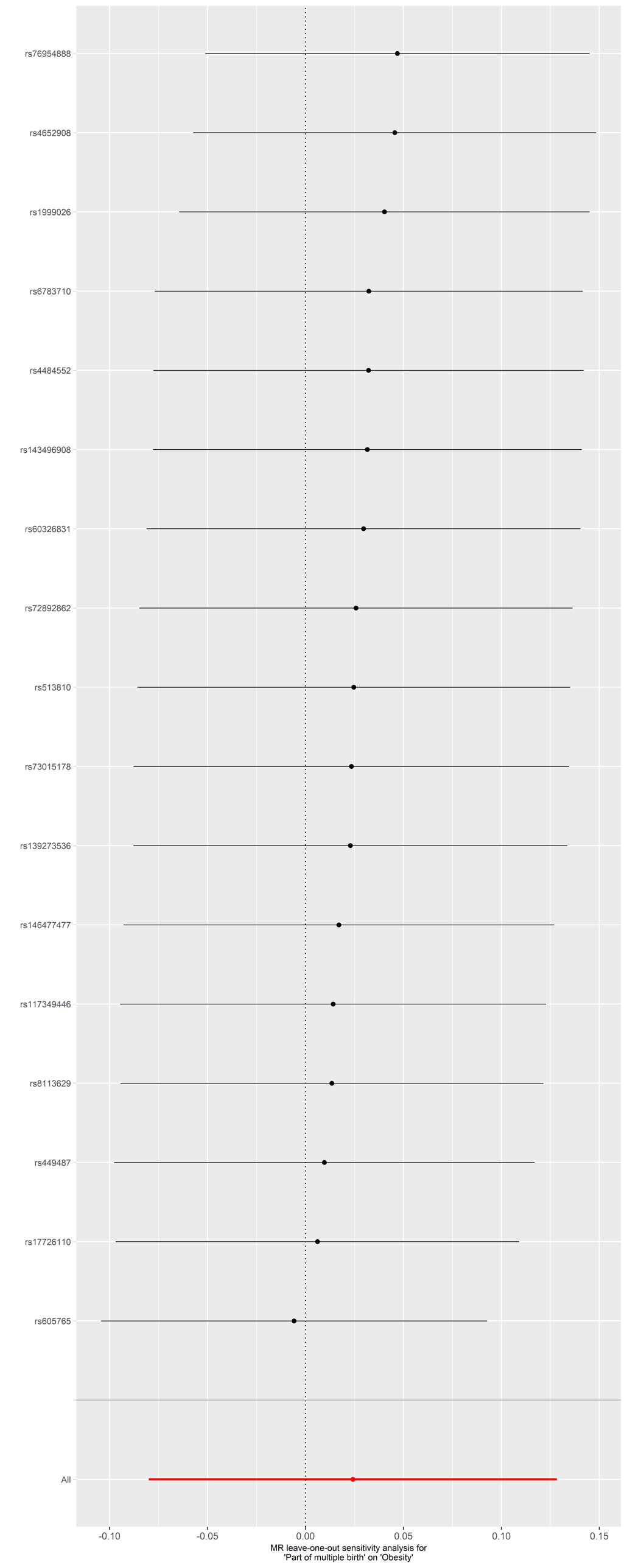


**Gout – Finngen**


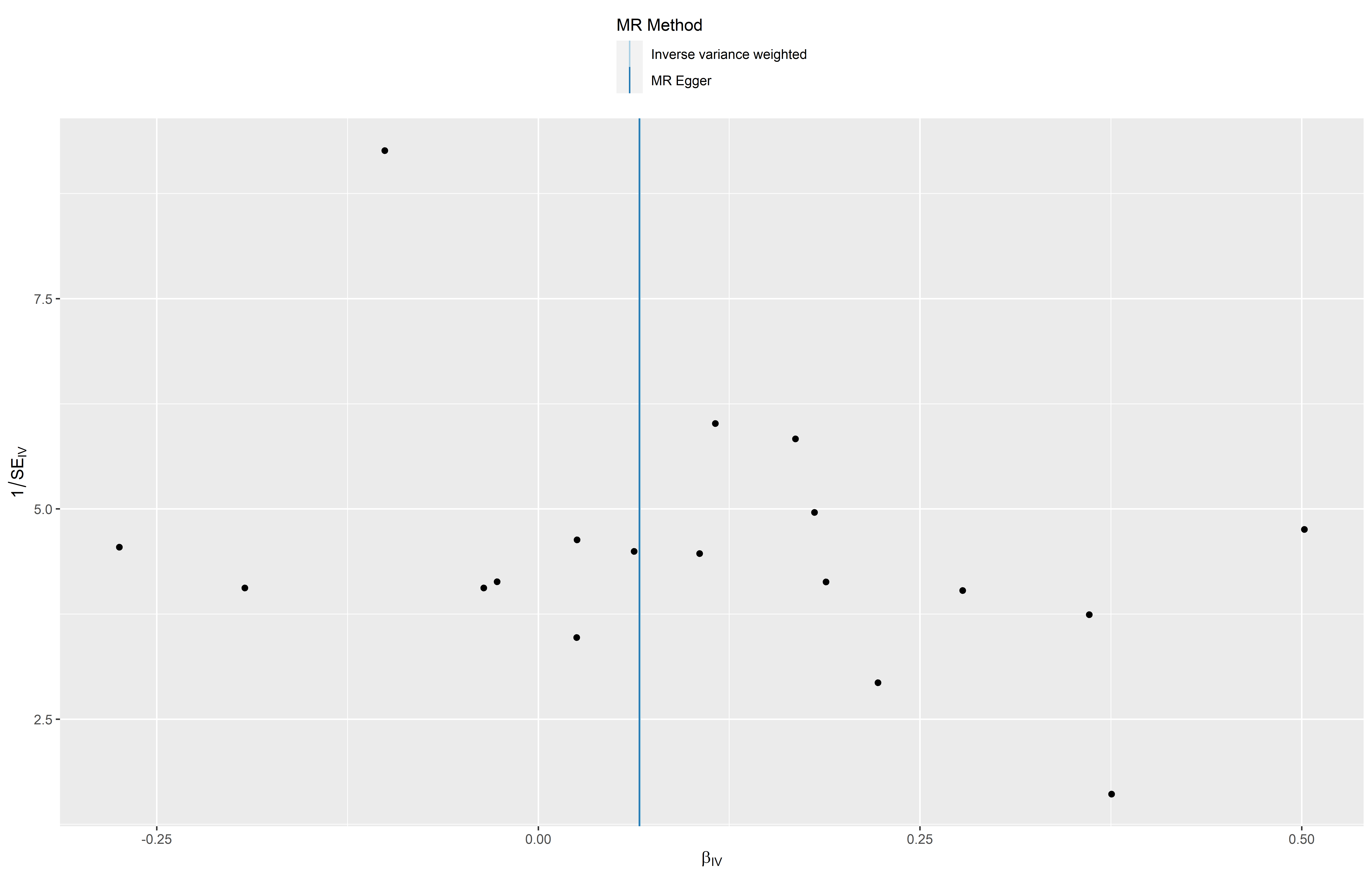

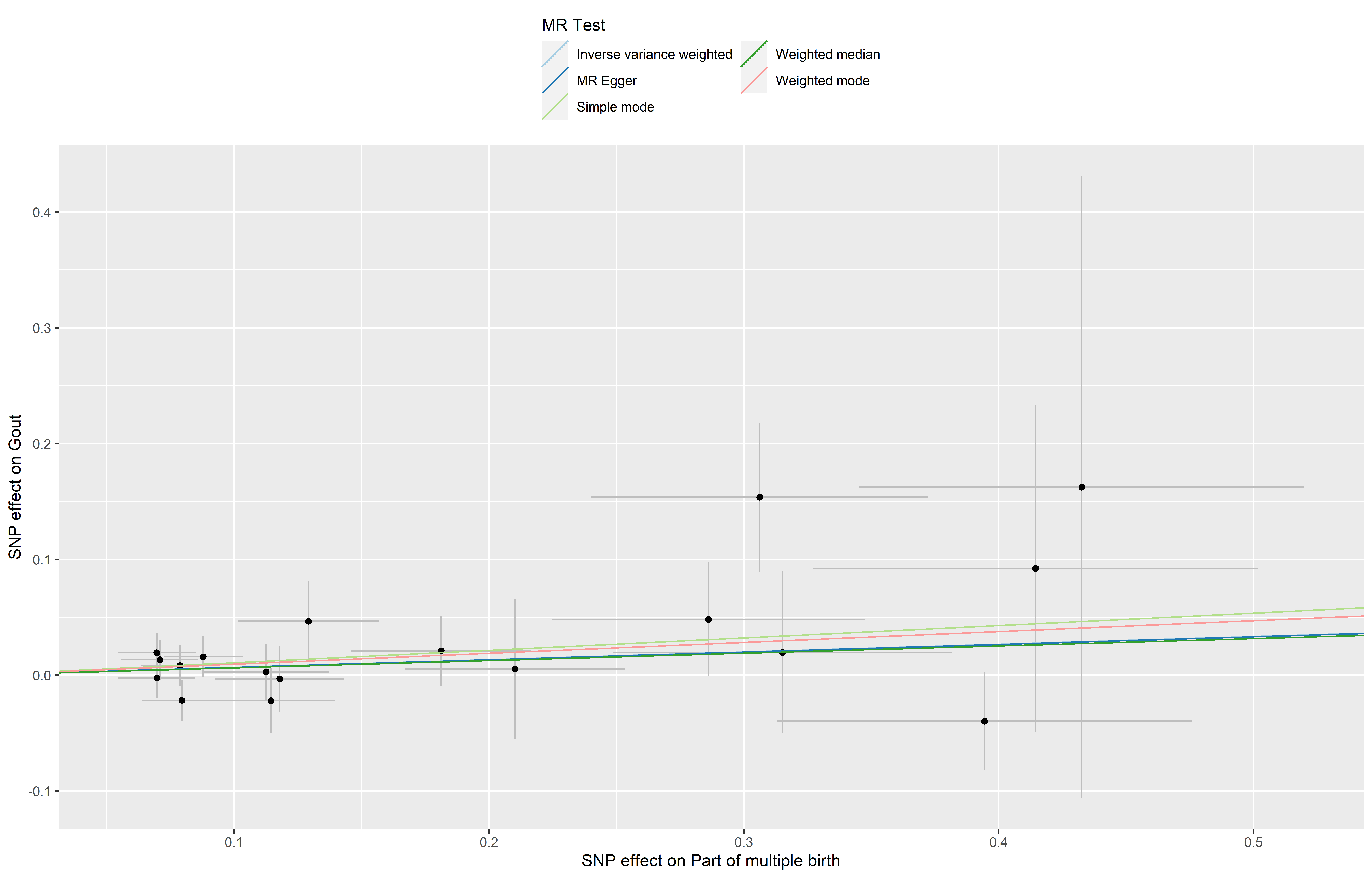


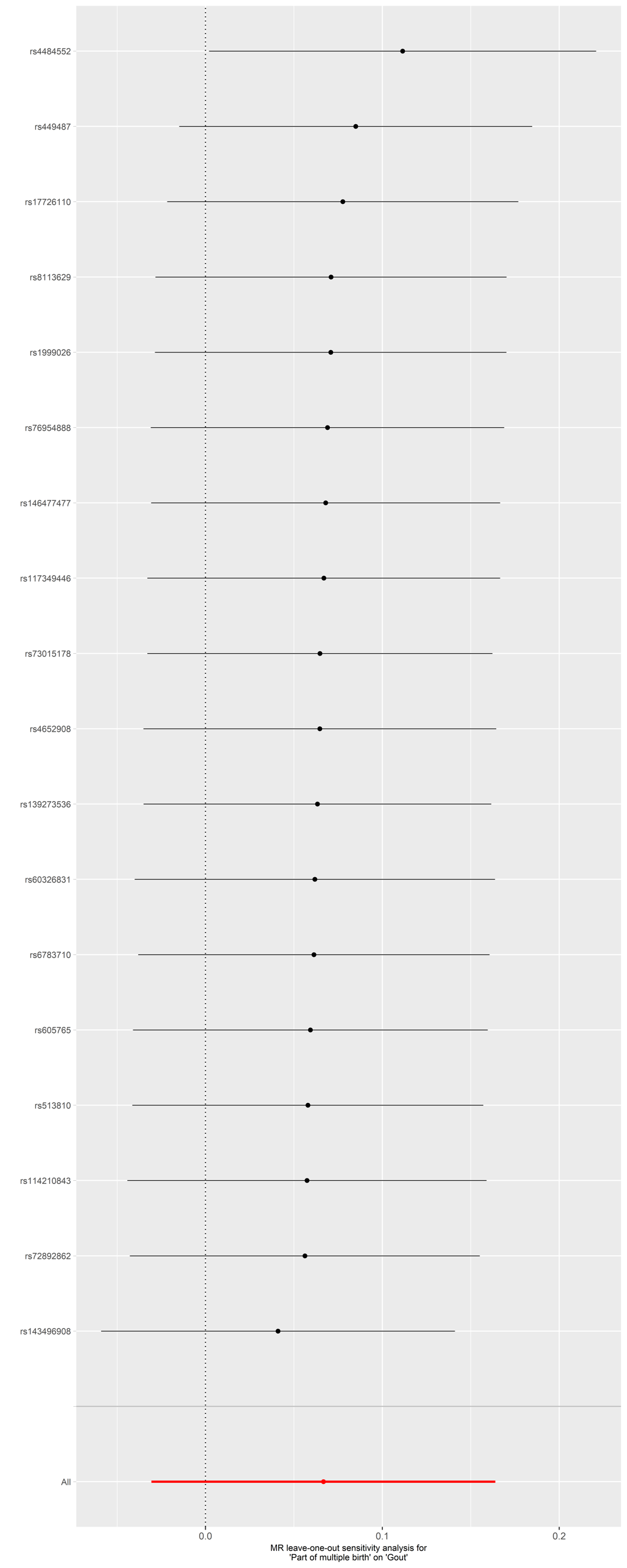


**Gout – UK Biobank**


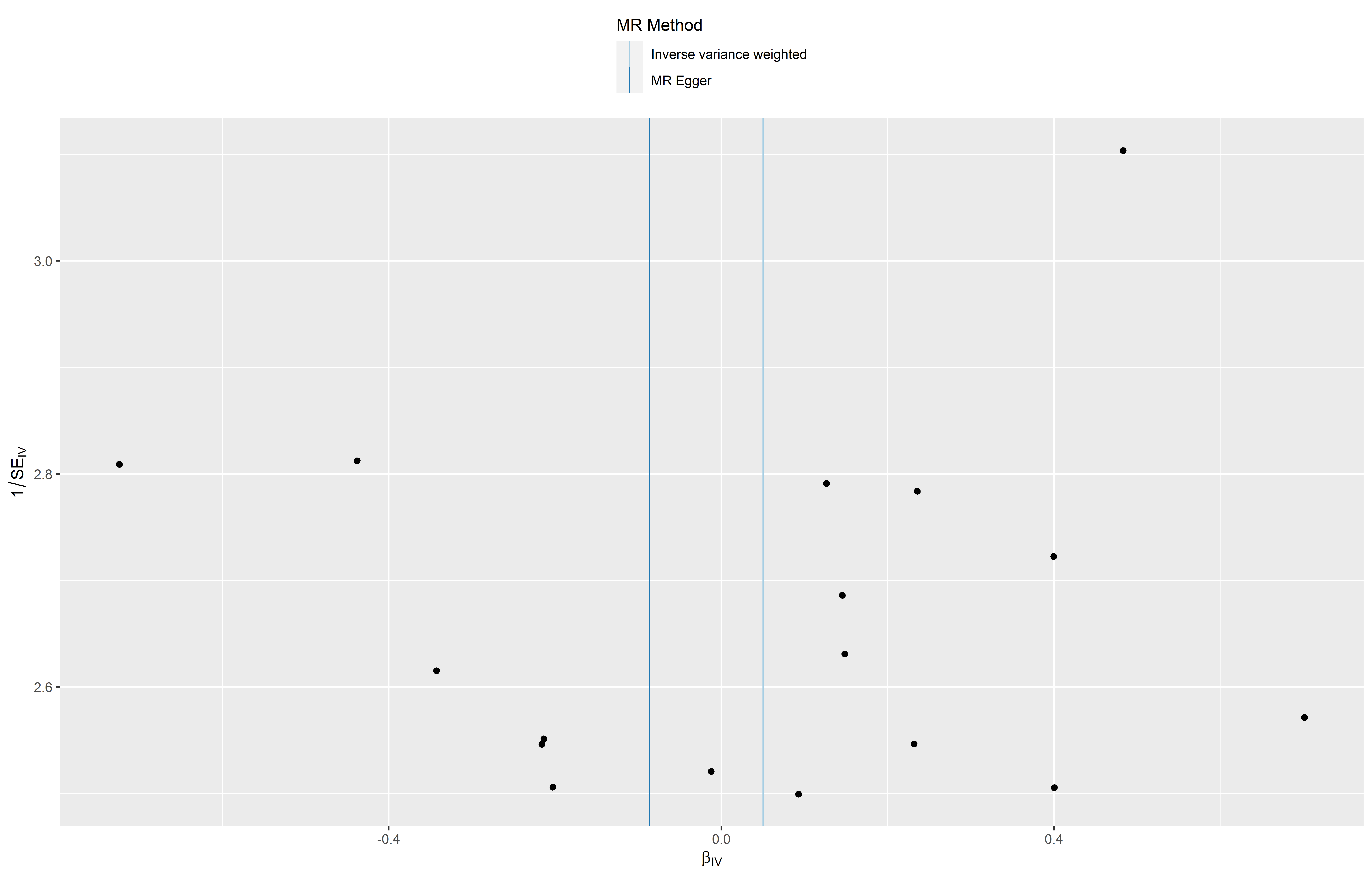

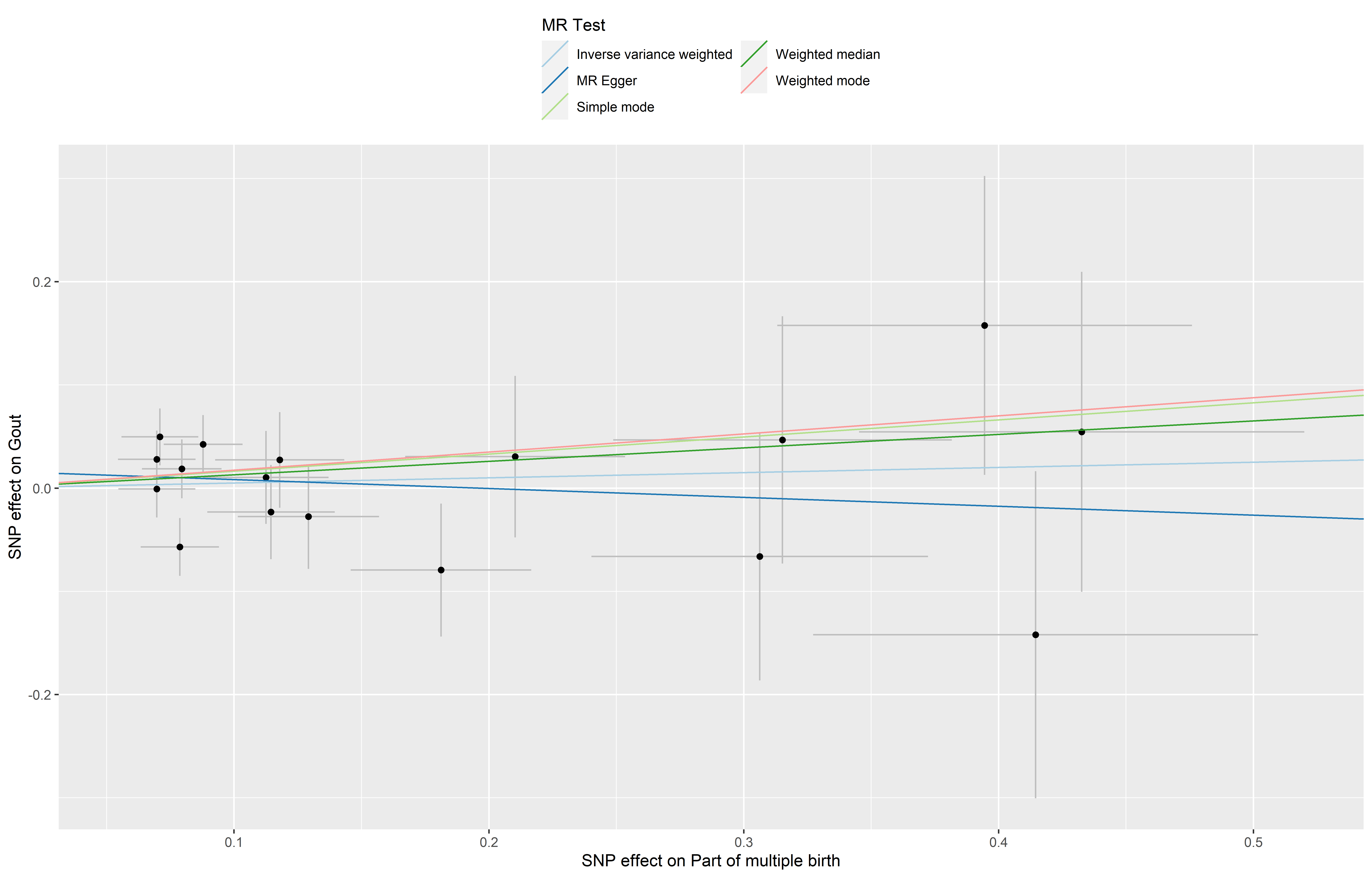


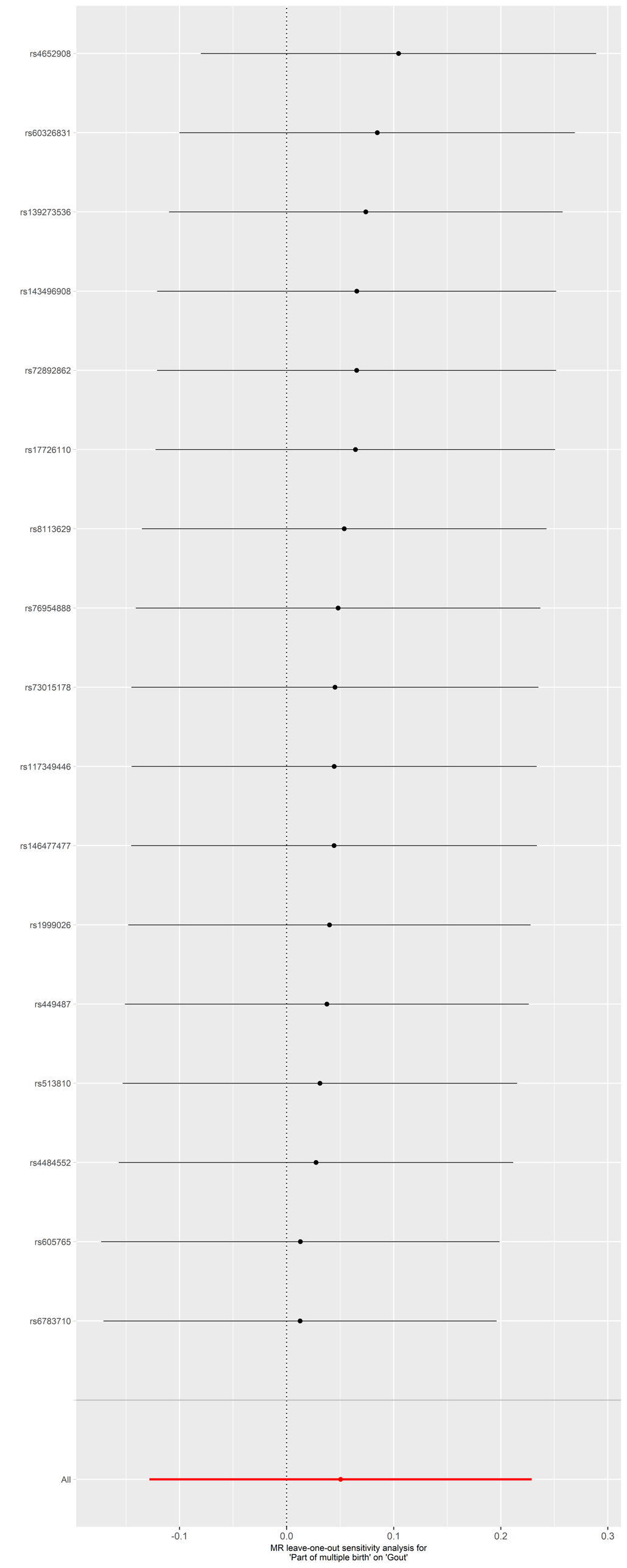

Supplement: Supplementary file 14 — Additional file 14: Material S6. The scatter plot, funnel plot and leave-one-out plot for the MR analysis of multiple birth and endocrine system disease. [file 12967_2023_4423_MOESM14_ESM.docx]
